# Network Pharmacology Approach for Predicting Targets of Zishen Yutai Pills on Premature Ovarian Insufficiency

**DOI:** 10.1155/2021/8215454

**Published:** 2021-08-04

**Authors:** Yihui Feng, Xinyi Chai, Yingyin Chen, Yan Ning, Ying Zhao

**Affiliations:** ^1^Guangzhou University of Chinese Medicine, Guangzhou 510405, China; ^2^Department of Gynecology, First Affiliated Hospital, Guangzhou University of Chinese Medicine, Guangzhou 510405, China; ^3^Lingnan Medical Research Center, Guangzhou University of Chinese Medicine, Guangzhou 510405, China; ^4^Singapore Thong Chai Medical Institution, 169874, Singapore; ^5^Shenzhen Maternity and Child Healthcare Hospital, Shenzhen 518028, China

## Abstract

**Methods:**

A comprehensive strategy based on several Chinese herb databases and chemical compound databases was established to screen active compounds of ZSYTP and predict target genes. For network pharmacological analysis, network construction and gene enrichment analysis were conducted and further verified by molecular docking.

**Results:**

A total of 476 target genes of ZSYTP were obtained from 205 active compounds. 13 herbs of ZSYTP overlapped on 8 active compounds based on the compound-target-disease network (C-T network). 20 biological processes and 9 pathways were strongly connected to the targets of ZSYTP in treating POI, including negative regulation of gene expression, mRNA metabolic process, hypoxia-inducible factor 1 (HIF-1) signaling pathway, and gluconeogenesis. Finally, molecular docking was visualized.

**Conclusion:**

Intriguingly, the signal pathways and biological processes uncovered in this study implicate inflamm-aging and glucose metabolism as potential pathological mechanisms of POI. The therapeutic effect of ZSYTP could be mediated by regulating glucose metabolism and HIF-1 signal pathway. Collectively, this study sheds light on the therapeutic potential of ZSYTP on POI.

## 1. Introduction

Premature ovarian insufficiency (POI) is a common reproductive endocrine disorder in females of childbearing age. POI is characterized by amenorrhea before the age of 40, with high gonadotropin and low estrogen [1]. Endocrine dysfunction can cause atrophy of the ovary, hot flashes, depression, and insomnia, all of which are symptoms of perimenopausal women [2]. Owing to the low levels of estrogen, women with POI may suffer from chronic complications, including osteoporosis and cardiovascular disease. As ovarian functional decline affects ovulation, the pregnancy and live birth rates dramatically decrease [3]. POI not only leads to reproductive disorders but also shortens a patient's life span, which has a negative impact on the quality of life [4, 5]. The conventional treatment for POI is hormone replacement therapy (HRT), which relieves perimenopausal symptoms, but is unable to improve ovulation function. Therefore, patients with POI are in urgent need of a more effective treatment.

Complementary and alternative medicine (CAM) refers to a group of diverse medical and healthcare systems, practices, and products that are not generally considered a part of conventional medicine [6] and include compound Chinese medicine compounds. Previous experimental studies and clinical experiences have suggested that compound Chinese medicine have significant clinical efficacy [7]. ZSYTP is a well-known compound Chinese medicine that is widely used for treating reproductive disorders [8, 9]. ZSYTP can effectively relieve the clinical symptoms caused by low estrogen through lowering serum follicle-stimulating hormone (FSH) levels and elevating serum estrogen levels [10, 11]. Compared with HRT treatment, ZSYTP can induce follicle development and stimulate ovulation [12]. Toxicological experiments have been conducted to prove no perinatal toxicity and observed adverse effects on livers, suggesting the pharmaceutical safety of ZSYTP [13–16]. Thus, ZSYTP might be a novel therapeutic strategy for POI. However, its pharmacological mechanism and molecular targets are unclear yet.

**Figure 1:**
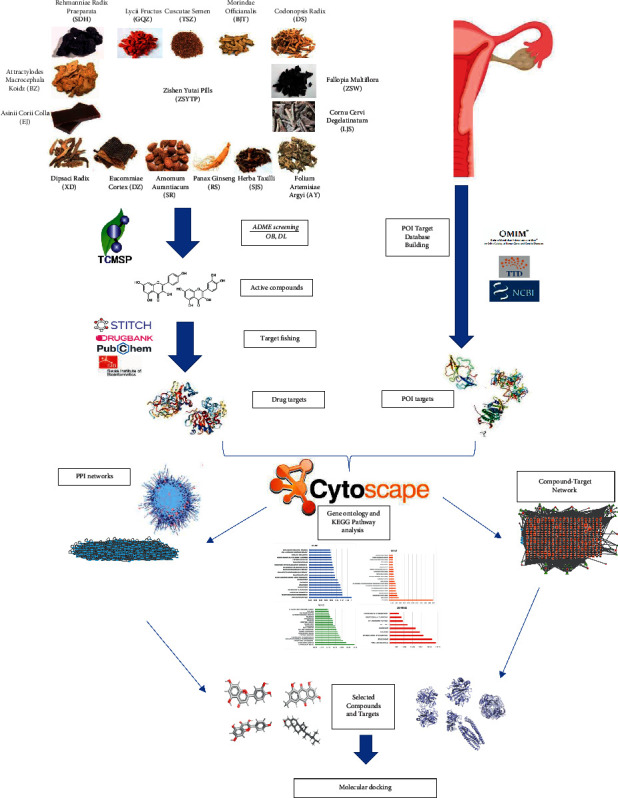
Workflow of the present study.

Network pharmacology is an approach to drug design that improves clinical efficacy and understands side effects and toxicity [17]. It enables effective prediction of a complex interplay among TCM components and targets through integrated network analysis [18]. In this study, we conducted a comprehensive network pharmacology approach to predict the potential pharmacological targets of ZSYTP on POI by molecular docking and network analysis. The workflow of this study on the pharmacological mechanism of ZSYTP on POI based on network pharmacology is presented in Figure 1.

## 2. Methods and Materials

### 2.1. Data Preparation

#### 2.1.1. Screening for Chemical Compounds of Herbs in ZSYTP

Chemical compounds from all 15 herbs in ZSYTP were obtained from the Traditional Chinese Medicine Systems Pharmacology (TCMSP; http://lsp.nwu.edu.cn/tcmsp.php, updated in March 2014) [19], TCM Database@Taiwan (http://tcm.cmu.edu.tw) [20], and BATMAN-TCM (http://bionet.ncpsb.org/batman-tcm/) [21]. The TCMSP is a unique pharmacology platform designed for Chinese herbs, the TCM Database@Taiwan is one of the most comprehensive TCM databases in the world, and BATMAN-TCM is a bioinformatics analysis tool for analyzing the active compounds of Chinese herbs.

The screening filters used in this research to maximize drug discovery included oral bioavailability (OB) ≥ 30% [22] and drug likeness (DL) ≥ 0.18 [23]. Active compounds were selected when both these criteria were met in accordance with absorption, distribution, metabolism, and excretion (ADME) consideration to ensure higher efficiency of the active compounds selected.

OB is the percentage value that measures the fractional extent of an orally administered drug that reaches the systemic circulation after ADME. A higher OB value indicates that a lesser amount of drug must reach the intended pharmacological effect, thereby reducing the risk of drug toxicity and potential side effects. A chemical compound with a low OB value indicates a poor drug effect and a higher intersubject variability. Thus, the OB value is one of the most commonly used pharmacokinetic properties in drug screening.

DL is a network pharmacological concept that indicates the similarity or likeness of the compound in question compared with known compounds. The concept originates from Lipinski's Rule of Five, which is used to estimate the possibility of obtaining a pharmacologically active compound, thereby reducing failure rates and increasing efficacy in pharmacological studies [24].

#### 2.1.2. Screening Target Genes of Each Herb in ZSYTP

The canonical SMILES and IUPAC International Chemical Identifier (InChI) of each of the chemical compounds were collated from PubChem Database (http://pubchem.ncbi.nlm.nih.gov/) [25] to ensure the uniqueness of the molecules in the database.

We input all molecular information of the chemical compounds of ZSYTP into the following: (1) STITCH (http://stitch.embl.de) [26], a database of known and predicted interactions between chemicals and proteins; (2) Swiss Target Prediction (http://www.swisstargetprediction.ch/index.php) [27], an online tool that predicts the most probable protein targets of molecules; (3) PubChem Database (http://pubchem.ncbi.nlm.nih.gov/), an online chemistry database providing drug-target identification; and (4) DrugBank (https://go.drugbank.com) [28], an online database containing information on known drugs and their corresponding target genes.

The canonical SMILES of each compound were input into the “chemical structure(s)” search engine of STITCH, and “Homo sapiens” was selected as the organism. Similarly, the canonical SMILES of each active compound were input into the search engine of Swiss Target Prediction; “Homo sapiens” was selected as the species, and target genes with a probability of >70% [27] were included.

Both the canonical SMILES and InChI key of the compounds were searched through the PubChem database, and target genes were subsequently obtained from the “Biological Test Results” panel, only including target genes with known bioactive outcomes.

The InChI key of each chemical compound was searched at DrugBank and consequent target genes were obtained.

#### 2.1.3. POI Targets Database Building

With reference to the POI guideline published by the *European Society of Human Reproduction and Embryology* (ESHRE) in 2015 [2], it was recommended that the term “premature ovarian insufficiency” be used for standard terminology. The guideline recognized a lack in proper clinical diagnostic definition for the condition and provided the diagnosis of POI with two conditions: (1) oligo/amenorrhea for at least four months and (2) an elevated FSH level >25 IU/L on two occasions more than two weeks apart. This study aims to investigate the pharmacological mechanism of ZSYTP on POI, therefore omitting keywords such as “premature menopause” and “premature ovarian failure” in our search for disease targets. “Premature ovarian insufficiency” was, therefore, the keyword used in the search engines listed as follows.

POI targets were collected through searches using databases such as Online Mendelian Inheritance in Man (OMIM) (https://www.omim.org) [29], NCBI Gene Database (https://www.ncbi.nlm.nih.gov/gene) [30], Therapeutic Target Database (TTD) (http://db.idrblab.net/ttd/) [31], and MalaCards (https://www.malacards.org) [32].

### 2.2. Network Construction Method

The network construction was built as follows: (1) ZSYTP chemical compounds-potential target network (C-T network), (2) PPI network of POI disease targets, (3) PPI network of ZSYTP target genes, and (4) PPI network of interaction between POI disease targets and ZSYTP target genes.

The network analysis software Cytoscape (https://cytoscape.org, version 3.8.2) [33] was used to visualize the networks. The nodes in the C-T network represent targets, compounds, pathways, while edges represent interactions.

The C-T network was constructed with Cytoscape software, and the network analyzer tool was used to evaluate the network of chemical compounds and their corresponding gene targets. PPI networks were visualized using BisoGenet plugin for the Cytoscape software.

### 2.3. Gene Ontology and KEGG Enrichment Analysis

Cytoscape ClueGO [34] application was used for gene ontology (GO) enrichment analysis for biological process, molecular function, cellular components, and KEGG. A *p*-value <0.05 was set as statistically significant.

### 2.4. Verification of Molecular Docking

**Figure 2:**
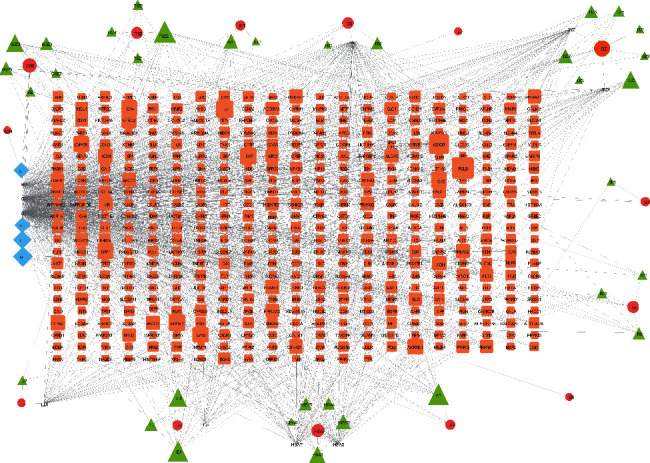
Compound-potential target network. Each red ellipse represents a herb in ZSYTP, and the green triangles represent active compounds. The orange rectangles represent the potential targets, and the blue diamonds represent common active compounds. The size of each node is proportional to the target degree in the network.

Molecular docking simulations were used to verify the binding of the target and the corresponding compound. 8 active compounds of the highest degree and 5 potential targets of the highest degree were obtained from the C-T network (Figure 2). Data on the construction of macromolecular protein target receptors were acquired via the RCSB PDB database (PDB, http://www.rcsb.org/) [35], and data on small molecule compounds were retrieved via the PubChem Database [25] and TCMSP [19]. The expulsion of water and ligand from macromolecular protein was performed by PyMol 2.4 [36], and format conversion was performed using Open Babel software. Molecular docking simulations of the macromolecular protein targets and the corresponding compounds were performed by AutoDockTool 1.5.6 and AutoDock 4.2.6 software [37]. The results were visualized by Pymol 2.4.

## 3. Results

### 3.1. Screened Chemical Compounds of Herbs in ZSYTP

A total of 1364 chemical compounds were found in the process, 76 from *Rehmanniae Radix Praeparata* (SDH), 188 from *Lycii Fructus* (GQZ), 29 from *Cuscutae Semen* (TSZ), 174 from *Morindae Officinalis Radix* (BJT), 134 from *Codonopsis Radix* (DS), 55 from *Atractylodes macrocephala Koidz* (BZ), 30 from *Dipsaci Radix* (XD), 119 from *Eucommiae Cortex* (DZ), 165 from *Amomum aurantiacum* (SR), 190 from *Panax Ginseng* (RS), 46 from *Herba Taxilli* (SJS), 135 from *Folium Artemisiae Argyi* (AY), 17 from *Fallopia multiflora* (HSW), 4 from *Colla Corii Asini* (EJ), and 2 from *Cornu Cervi Degelatinatum* (LJS).

In accordance with the ADME thresholds of OB ≥ 30% and DL ≥ 0.18, 187 chemical compounds were obtained as follows: 2 from SDH, 45 from GQZ, 11 from TSZ, 20 from BJT, 21 from DS, 9 from BZ, 6 from XD, 28 from DZ, 10 from SR, 22 from RS, 2 from SJS, 9 from AY, and 2 from HSW. EJ and LJS were not found in any of the TCM databases. However, a further 18 compounds with lower OB or DL values were consolidated as they hold extensive pharmacological activities: 10 from HSW, 4 from EJ, and 2 from LJS. The final list of 205 active compounds with their parameters and sources are provided in Supplementary Table 1.

### 3.2. Active Target Gene Prediction of ZSYTP

Target gene prediction of the ZSYTP active compounds based on molecular similarity was conducted by entering each unique molecular data into STITCH, PubChem, Swiss Target Prediction, and DrugBank. 481 target genes were obtained from ZSYTP active compounds upon eliminating duplication. The target genes of each herb of ZSYTP are listed in Supplementary Table 2.

### 3.3. POI Targets

The keyword “premature ovarian insufficiency” was used in the search through OMIM, TTD, NCBI Gene Database, and MalaCards as a POI disease target. OMIM and TTD yielded no results, and MalaCards provided potential disease targets including premature ovarian failure (POF), which were not included in this study. A total of 119 disease targets were obtained (Supplementary Table 3).

### 3.4. Constructed ZSYTP Compound-Potential Target Network (C-T Network)

Cytoscape software was used to map out a network illustrating the relationship between each herb, its corresponding active compounds and targets, and the C-T network. The nodes depict herbs, active compounds, and target genes, while the edges indicate the correlation between them (Supplementary Files 4-5). The constructed C-T network of 48 active compounds contained 538 nodes and 1418 edges (Figure 2). Compounds and target genes of the highest degrees were noted for molecular docking verification (Table 1; Supplementary File 6B). The median degree of the 48 active compounds in the network was 9.5 (number of related targets), suggesting that the most active compounds influence multiple targets. Specifically, quercetin, kaempferol, and luteolin acted on 384, 255, and 105 targets, respectively (Supplementary File 6A), indicating that they could be crucial in the therapeutic potential of ZSYTP on POI. 13 herbs of ZSYTP overlapped on 8 active compounds; they are indicated in Table 2 with their corresponding degree.

### 3.5. Core Network Analysis

PPI networks for both potential drug and disease targets were constructed with Cytoscape version 3.8.2 plug-in BisoGenet. Of note, 3938 nodes and 99,199 edges were established in the PPI network for disease targets, while 7834 nodes and 182,596 edges were found in the PPI network for ZSYTP drug targets.

**Figure 3:**
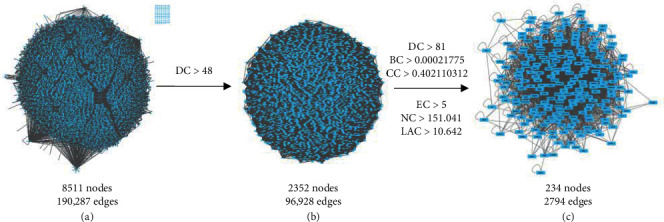
Topological screening process of PPI network.

Topological feature analysis was subsequently performed by intersecting both PPI networks based on “betweenness (BC),” “closeness (CC),” and “eigenvector (EC),” deriving a total of 8511 nodes and 190,297 edges (Figure 3(a)). Based on a previous study by Zhou et al. [16], targets were selected with parameters above twice the median value. The first selection criteria were set as degree >48, and 2352 nodes and 96,928 edges were derived (Figure 3(b)). The 2352 targets were further screened with the second selection criteria of degree, DC > 81, BC > 0.00021775, CC > 0.402110312, EC > 5, neighborhood connectivity (NC) > 151.041, and node (LAC) > 10.642. A final PPI network of 234 nodes and 2,794 edges was identified (Figure 3(c); Supplementary File 7).

### 3.6. Gene Ontology and KEGG Analysis

#### 3.6.1. Gene Ontology Biological Process (GO-BP)

The enrichment analysis was completed with the Cytoscape ClueGO plugin for visualization. 221 biological processes were retrieved; the *p*-value was set at >1.0 × 10^−21^, and 20 processes were selected for further analysis. The full list of enriched GO-BP terms is presented in Supplementary Table S8A.

The 20 biological processes selected were mainly involved in the negative regulation of gene expression (15.46%), telomere maintenance (10.57%), viral process (8.76%), regulation of protein localization of chromosome, telomeric region (5.41%), mRNA metabolic process (5.41%), and establishment of protein localization to organelles (5.15%). Percentage values were calculated based on the number of processes in a group out of the 221 chart records retrieved.

For negative regulation of the gene expression group, biological processes, such as negative regulation of nucleobase-containing compound metabolic process, negative regulation of RNA metabolic process, negative regulation of macromolecule biosynthetic process, negative regulation of biosynthetic process, negative regulation of nucleic acid templated transcription, and negative regulation of gene expression (epigenetic), were identified. For the mRNA metabolic process group, biological processes, such as RNA processing, mRNA metabolic process, RNA splicing, protein-containing complex subunit organization, and mRNA catabolic process, were identified.

The other biological processes selected for further analysis were as follows: chromosome organization, cellular protein-containing complex assembly, ATP-dependent chromatin remodeling, telomere maintenance, CENP-A containing nucleosome assembly, posttranscriptional regulation of gene expression, protein localization to organelles, SRP-dependent cotranslational protein targeting to membrane, cellular response to DNA damage stimulus, chromatin assembly and disassembly, DNA repair, and double-strand break repair.

**Figure 4:**
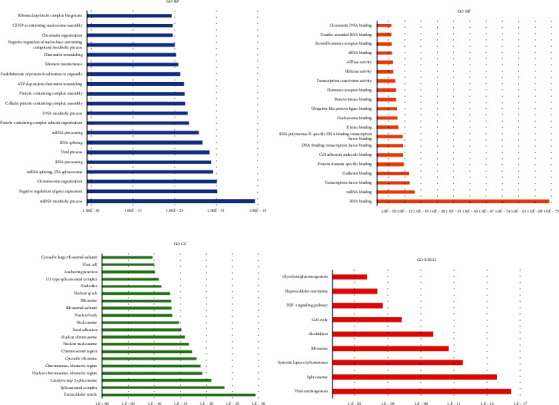
The GO function and KEGG pathway enrichments. (a) Enriched BP functions of active target genes; (b) enriched MF functions of active target genes; (c) enriched CC functions of active target genes; (d) KEGG pathway enrichments. BP: biological process; MF: molecular function; CC: cellular component.

The selected processes of GO-BP were visualized (Figure 4(a)) and ranked according to their corresponding *p* values.

#### 3.6.2. Gene Ontology Molecular Function (GO-MF)

Gene ontology analysis of MF identified 45 functions. The results indicated that the active target genes of ZSYTP that act on POI have functions, including transcription factor binding (15.56%), rRNA-binding (8.89%), helicase activity (8.89%), and nucleosome binding (6.67%). 20 molecular functions with *p* values >1.0 × 10^−7^ were selected and visualized; the results are shown in Figure 4(b) with corresponding *p* values. The list of enriched GO-MF terms is presented in Supplementary Table S8B.

For the transcription factor binding group, the identified functions included transcription factor binding, DNA-binding transcription factor binding, RNA polymerase II-specific DNA-binding transcription factor binding, and transcription coactivator activity.

For the group of rRNA binding, the identified functions included RNA binding, mRNA binding, rRNA binding, and double stranded RNA binding.

Other identified functions included cadherin binding, protein domain-specific binding, cell adhesion molecule binding, kinase binding, nucleosome binding, ubiquitin-like protein binding, protein kinase binding, hormone receptor binding, helicase activity, ATPase activity, steroid hormone receptor binding, and chromatin DNA binding.

#### 3.6.3. Gene Ontology Cellular Component (GO-CC)

Gene ontology analysis of CC identified 82 components, including spliceosomal complex (18.07%), cytosolic ribosomes (10.84%), nuclear chromosome, telomeric region (10.84%), and SWI/SNF superfamily-type complex (7.23%). Twenty cellular components with *p* values >1.0 × 10^−10^ were selected for further analysis. The full list of enriched GO-BP terms is presented in Supplementary Table S8C.

GO-CC analysis identified the following cellular components as enriched, extracellular vesicle, spliceosome complex, catalytic step 2 spliceosome, nuclear chromosome (telomeric region), chromosome (telomeric region), cytosolic ribosome, chromosomal region, nuclear nucleosome, focal adhesion, nucleosome, nuclear body, ribosomal subunit, ribosome, nuclear speck, nucleolus, U2-type spliceosomal complex, anchoring junction, host cell, and cytosolic large ribosomal subunit.

The 20 selected components of GO-CC analysis are visualized in Figure 4(c) with their corresponding *p* values.

#### 3.6.4. KEGG Pathway Analysis

The results of KEGG pathway analysis highlighted nine pathways, in which target genes of ZSYTP acting on POI are enriched significantly; 33.33% of the enriched target genes act on viral carcinogenesis and 22.22% on the hypoxia-inducible factor 1 (HIF-1) signaling pathway; hepatocellular carcinoma, cell cycle, spliceosome, and ribosome pathways were equally divided at 11.11%.

All 9 pathways highlighted by KEGG analysis were presented (Figure 4(d)) and ranked according to their corresponding *p* values (Supplementary Table 8D).

**Figure 5:**
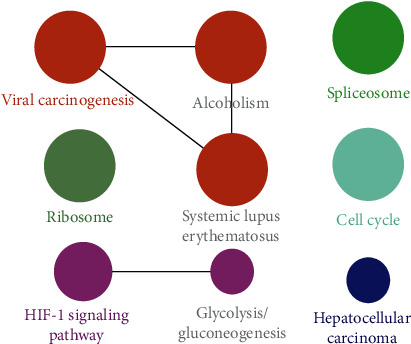
KEGG pathway enrichment analysis visualized by ClueGO.

Cytoscape ClueGO plug-in was utilized to better visualize the KEGG pathway as shown in Figure 5.

### 3.7. Molecular Docking Visualization

**Figure 6:**
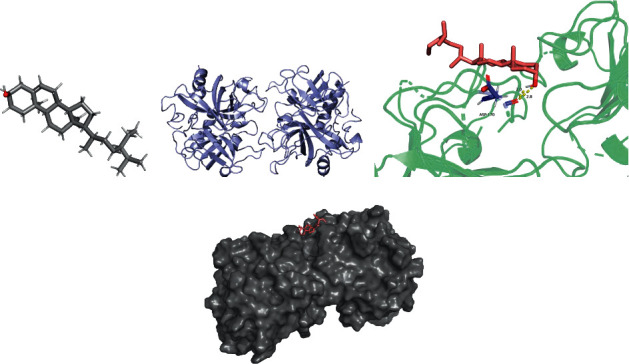
F2 beta-sitosterol molecular docking. 3D structures of (a) beta-sitosterol and (b) F2; (c) molecular docking simulation; (d) display protein surface of molecular docking simulation.

**Figure 7:**
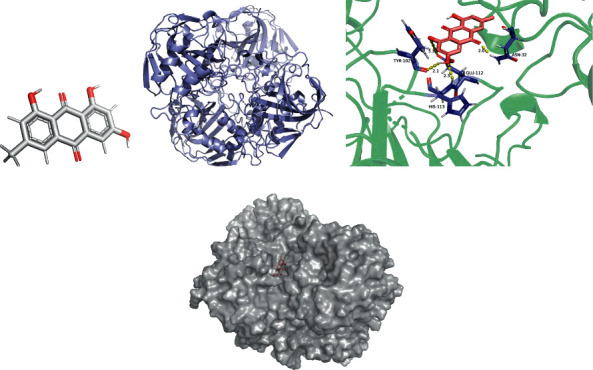
CA4 emodin molecular docking. 3D structures of (a) emodin and (b) CA4; (c) molecular docking simulation; (d) display protein surface of molecular docking simulation.

**Figure 8:**
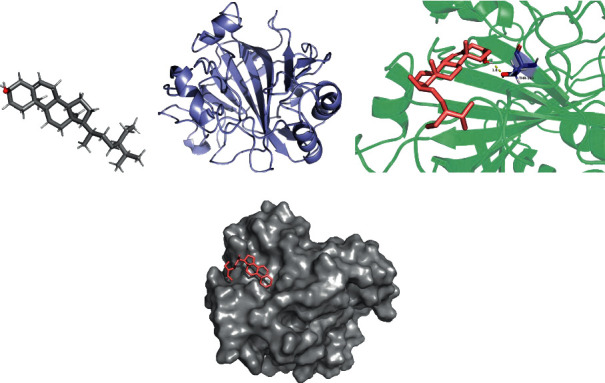
CA7 beta-sitosterol molecular docking. 3D structures of (a) beta-sitosterol and (b) CA7; (c) molecular docking simulation; (d) display protein surface of molecular docking simulation.

**Figure 9:**
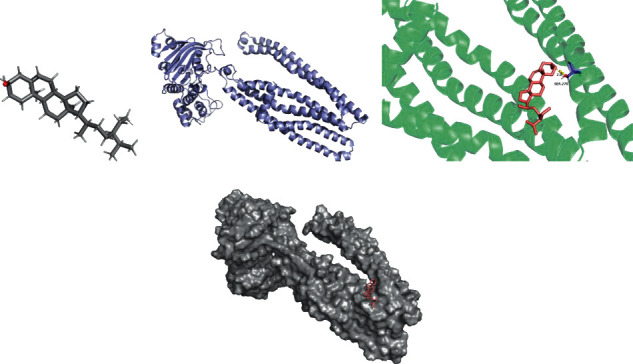
ABCB1 beta-sitosterol molecular docking. 3D structures of (a) beta-sitosterol and (b) ABCB1; (c) molecular docking simulation; (d) display protein surface of molecular docking simulation.

The lowest binding energy of the molecular docking of potential targets and their designated compounds are presented in Table 3. The simulations of the molecular docking of F2-beta-sitosterol, CA4-emodin, CA7-beta-sitosterol, and ABCB1-beta-sitosterol are shown in Figures 6–9, respectively.

## 4. Discussion

ZSYTP is a Traditional Chinese Medicine (TCM) prescription derived from the clinical experience of Professor Luo Yuankai, a nationally acclaimed TCM scholar of Guangzhou University of Chinese Medicine. Luo received a Class 1 award from China's National Health Commission for his contributions with the ZSYTP.

ZSYTP was originally derived as treatment to prevent recurrent and early pregnancy loss. In accordance with the concept of TCM that states that different diseases can be treated with the same therapeutic principle, and on the basis of the TCM theory that kidneys are closely linked with reproduction, it is postulated that ZSYTP could also be used clinically for irregular periods and infertility.

It has been found that ZSYTP can improve ovarian function in patients with POI [11–14]; as a result, we sought to explore the possible etiologies of POI and the pharmacological mechanisms of ZSYTP on POI.

The nine pathways highlighted by KEGG analysis were studied extensively for further corroboration with existing literature that would provide insights and possible hypotheses of the pharmacological mechanisms of ZSYTP in the context of POI. In particular, the HIF-1 signaling pathway was highlighted by KEGG analysis due to its multiple roles in ovarian function.

An overview of the C-T network combined with GO and KEGG analysis led us to postulate that the potential mechanism of ZSYTP on POI is likely connected to the anti-inflammatory and antioxidant properties of ZSYTP, as well as participating in glucose metabolism. GO and KEGG analysis also highlighted a change in cell and gene expression as a possible pathological mechanism of POI.

### 4.1. Inflamm-Aging and POI

A term first coined by Franceschi et al. [38] in 2000, “inflamm-aging” refers to the body undergoing a chronic and progressive inflammatory state [39–41] through the process of aging.

Chronic inflammation has been closely linked with oxidative stress [42], cytokines, and DNA damage. Oxidative stress refers to the imbalance of reactive oxygen species (ROS) and antioxidants in the body. ROS help to fight pathogens while being kept in check by antioxidants, an imbalance between the two leads to ROS damage of proteins, DNA, and fatty tissue. The oxidative stress levels and neutrophil-to-lymphocyte ratio were found to be elevated in subjects with POI, indicating a state of inflammation in these patients [43].

In reference to the C-T network, active compounds, such as quercetin, kaempferol, luteolin, beta-sitosterol, and emodin, were found to be key hub compounds of ZSYTP, in descending degree, respectively.

Quercetin, kaempferol, and luteolin are natural flavonoids that demonstrate anti-inflammatory, antioxidant, anticarcinogenic properties and gene expression-modulating potential [44]. A recent study indicated that quercetin could protect ovarian function in female albino mice with cyclophosphamide-induced premature ovarian failure. Primordial follicles and serum anti-Müllerian hormone (AMH) were increased, while the number of atretic follicles was decreased under quercetin treatment, suggesting a protective effect of quercetin on ovarian function in cyclophosphamide-induced POF [45].

The C-T network also highlighted NFE2L2 and NOX4 as key targets of ZSYTP. NFE2L2 encodes a transcription factor that regulates genes containing antioxidant response element (ARE) in their promoters; these genes encode proteins involved in the production of free radicals. NOX4 encodes a family of enzymes that catalyzes the reduction of molecular oxygen to various ROS.

It is, therefore, possible that the potential anti-inflammatory and antioxidant effect of ZSYTP could be achieved by the combined actions of quercetin, kaempferol, and luteolin via key gene targets; together, these actions could reduce ovarian inflammation and possibly slow ovarian degradation in POI.

### 4.2. Glucose Metabolism in POI

POI describes a hypoestrogenic state in women that is associated with metabolic changes [46]. As estrogen optimizes insulin activity, multiple studies have aimed to understand glucose metabolism and insulin resistance levels in patients with POI [47]. A meta-analysis was conducted to investigate the association of POI with type 2 diabetes (T2DM) [48], the results of which demonstrated that women with POI presented with a higher risk of T2DM compared with women of normal menopausal age (45–55 years) [49–51].

KEGG analysis implicated the HIF-1 signaling pathway and glycolysis as being associated with the underlying therapeutic mechanisms of ZSYTP in POI. HIF-1 is a transcription factor of two subunits, HIF-1a and HIF-1b [52]. The activity of HIF-1 is mainly determined by HIF-1a, which is regulated by hypoxia and hyperglycemia. Many studies have aimed to elucidate the relationship between HIF-1a gene expression in cells and hyperglycemia. Although the molecular mechanism of this relationship is still unclear, researchers have come to an agreement that hyperglycemia is directly linked to a compromised HIF-1a expression level.

**Figure 10:**
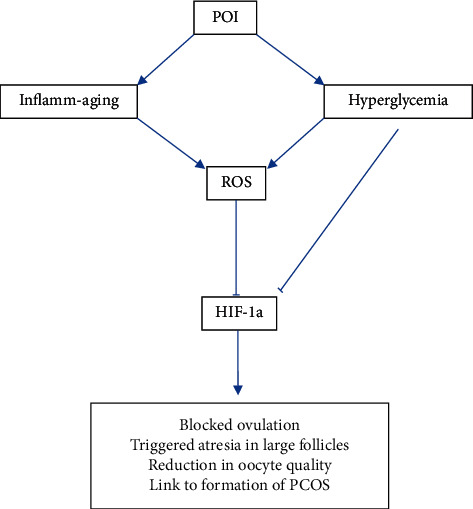
Possible etiology of POI leading to repressed HIF-1a protein levels.

Inhibition of HIF-1a expression was found to have triggered atresia in large follicles of mice with polycystic ovary syndrome (PCOS) and, therefore, prevented ovulation [53]. One study showed that HIF-1a signaling is inhibited in PCOS rat models [54], and after clinical PCOS treatment of dimethyldiguanide, PCOS symptoms were improved by rescuing this pathway, increasing HIF-1a gene expression in the process. Another study exposed human primary granulosa cells of subjects with PCOS to mitochondrial and glycolysis inhibitors and compared mitochondrial activity and glycometabolism with controls [55]. It was found that HIF-1a gene expression decreased, while ROS levels increased upon inhibition, and the researchers concluded that glycolysis and high HIF-1a expression in human primary granulosa cells are required for oocyte competence of PCOS. Therefore, it can be surmised that decreased levels of HIF-1a gene expression have a negative effect on ovarian follicle development; these hypothesized pathways are summarized in Figure 10.

Hyperglycemia augments oxidative stress and contributes to the overproduction of ROS [56], which, in turn, downregulates HIF-1a levels [57] via multiple possible mechanisms [58]. The significance of identifying the potential pathological mechanism of HIF-1 signaling pathway and glycolysis in POI could guide future pharmacological research to focus on possible therapeutic approaches.

In ZSYTP, active compounds that act on glucose metabolism include quercetin, kaempferol, emodin [59], luteolin, and chrysophanol; among them, quercetin is also involved in the inhibition of intestinal glucose absorption, insulin secretion, and insulin-sensitizing activities. This is substantiated by reports that quercetin intake results in a significant decrease in insulin resistance in PCOS cases [60–62].

It is possible that the active compounds of ZSYTP collectively act on regulating glucose metabolism and HIF-1 expression, therefore improving ovarian follicle development, slowing the rate of ovarian degradation in POI.

### 4.3. Change in Gene and Cell Expression

Subsequent GO and KEGG analysis aided in the elucidation of another possible pharmacological mechanism of ZSYTP on POI. The enriched GO term of BP with the smallest *p*-value was “mRNA metabolic process,” which refers to the process of carrying messages transcribed from DNA to the protein assembly at ribosomes. Moreover, “negative regulation of gene expression” was the group of biological processes highlighted in the GO-BP analysis, implying abnormal regulation of gene expression in POI.

The most enriched GO-CC was “extracellular vesicles (EVs).” Recent studies have suggested the ability of EVs to transfer functional RNA from cell to cell [63, 64] and their involvement on immune responses. Indeed, a previous study reported that HIV coreceptors could be transferred between cells with EVs, increasing the body's susceptibility to infection [65, 66]. EVs have been a topic of interest in the treatment for POI. Several studies indicated that EVs derived from human umbilical cord mesenchymal stem cells (HUCMSCs) can restore ovarian function of chemically induced POI [67].

The most significant enriched GO-MF was “RNA binding,” which further implies the importance of RNA in the mechanism of ZSYTP on POI.

KEGG analysis indicated “viral carcinogenesis” as the most enriched process. The mechanisms of viral carcinogenesis include direct transformation through the expression of viral genes and indirect transformational activities in cells. These activities increase translation of modified proteins with altered cell function, which corroborate with the GO-BP analysis of multiple negative regulation of gene expression processes identified.

Multiple studies have shown decreased protein expression in POI [68–72], signifying the significance of a downregulated gene as a possible cause for POI.

### 4.4. Molecular Docking

According to previous research, we considered the binding activity of molecular docking simulations to be practical when the binding energy was < −1.2 kcal/mol (−5.0 kJ/mol) and dynamite when the binding energy was < −5.0 kcal/mol. In our study, all of the binding energies were < −1.2 kcal/mol, and 12.5% of binding energies were < −5.0 kcal/mol. Furthermore, it was found that beta-sitosterol could stably dock to the F2 protein structure, while the H-bond plays a critical role at residue ASP-170. These active compounds may provide a foundation for treating POI, and the therapeutic action could be performed by correlative pathways in ZSYTP.

## 5. Conclusion

POI remains a debilitating disease for women with no fixed treatment protocol currently. ZSYTP has previously been shown to have a clinical effect on POI and warrants further research [11–14]. Our study mapped out the active compounds and corresponding gene targets of ZSYTP and further explored the pharmacological mechanisms underlying the effects of ZSYTP on POI using the method of network pharmacology.

Our results provide future research directions for the therapeutic use of ZSYTP in POI into three aspects: (1) the anti-inflammatory and antioxidant effect, (2) regulation of glucose metabolism, and (3) negative regulation on mRNA metabolic process. Our results also indicated that quercetin and kaempferol, as the two major active compounds found in ZSYTP, have potential pharmacological effects on POI. Future studies should aim to validate the effect of quercetin and kaempferol on POI to elucidate the underlying mechanism. In addition, future studies should also ascertain the status of glucose metabolism in patients with POI.

In conclusion, it is possible that the therapeutic potential of ZSYTP on POI is a multipathway effect, and therefore, more research is warranted to fully elucidate this relationship. The results of this study brought focus to potential pharmacological mechanisms of ZSYTP and its effect on POI. Multiple hypotheses of the pathological mechanism of POI were thereby formulated. Here, we provide a preliminary platform showcasing a comprehensive study of a TCM formula for POI, establishing a protocol enhancing TCM drug discovery to be more systematic and efficient.

## Figures and Tables

**Table 1:** List of top ten active compounds of ZSYTP based on degree (number of related targets).

Abbreviation	Active compound	Degree	Herbs involved
D	Quercetin	384	GQZ, TSZ, DZ, AY, SJS
C	Kaempferol	255	TSZ, DZ, RS
DS1	Luteolin	104	DS
B	Beta-sitosterol	97	GQZ, TSZ, DZ, AY, RS, BJT, SR, HSW, BZ
HSW2	Emodin	58	HSW
DZ7	Helenalin	48	DZ
HSW7	Rhein	47	HSW
LJJ1	Calcium phosphate	44	LJJ
DZ1	Mairin	29	DZ
EJ2	Histidine	28	EJ

**Table 2:** List of overlapping active compounds of ZSYTP.

Active compound	Degree	Herbs involved
Quercetin	384	GQZ, TSZ, DZ, AY, SJS
Kaempferol	255	TSZ, DZ, DS
Beta-sitosterol	97	TSZ, BJT, BZ, SR, RS, AY, HSW
Mandenol	18	GQZ, AY
Glycitein	16	BJT, DS, RS
Diop	12	GQZ, DS
Stigmasterol	12	GQZ, SR, RS, DS, AY
CLR	5	GQZ, TSZ

**Table 3:** Lowest binding energy of compounds-target molecular docking (kcal/mol).

Target	Degree value	*β*-Sitosterol	Emodin	Kaempferol	Luteolin	Quercetin
POLB	20	−4.86	−3.98	−3.5	−3.54	−3.22
GSK3B	18	−4.31	−1.53	−2.5	−2.37	−1.86
F2	14	−6.67	−4.62	−4.82	−4.48	−2.97
ABCB1	13	−5.3	−3.4	−2.17	−2.64	−1.73
CA7	13	−5.94	−4.63	−4.27	−4.16	−4.25
CA4	13	−3.7	−5.34	−2.39	−3.55	−1.91
CA12	12	−4.0	−4.4	−3.01	−2.35	−2.83
CA2	12	−5.08	−3.96	−3.22	−3.02	−2.7

## Data Availability

The data used in this study are available from the corresponding author upon request.

## Supplementary Materials

Supplementary Materials Supplementary Table 1: active compounds of each herb in ZSYTP. Supplementary Table 2: corresponding targets of active compounds: (A) two target genes of SDH; (B) 138 target genes of GQZ; (C) 202 target genes of TSZ; (D) 25 target genes of BJT; (E) 121 target genes of DS; (F) 11 target genes of BZ; (G) 283 target genes of DZ; (H) 13 target genes of SR; (J) 112 target genes of RS; (K) 76 target genes of SJS; (L) 115 target genes of AY; (M) 149 target genes of HSW; (N) 68 target genes of EJ; (P) 46 target genes of LJJ. Supplementary Table 3: POI targets. Supplementary Table 4: compounds and targets attributes of C-T network. Supplementary Table 5: data of compound-target network. Supplementary Table 6: analysed C-T network: (A) active compounds of C-T network and corresponding degrees; (B) targets of C-T network and corresponding degrees. Supplementary Table 7: ZSYTP core PPI network. Supplementary Table 8: gene ontology and KEGG analysis: (A) GO-BP analysis; (B) GO-MF analysis; (C) GO-CC analysis; (D) KEGG pathway enrichment analysis.
