# Supplementary material for: Network Pharmacology Approach for Predicting Targets of Zishen Yutai Pills on Premature Ovarian Insufficiency
Source: Evid Based Complement Alternat Med. 2021 Aug 4;2021:8215454. doi: 10.1155/2021/8215454 (PMC8357500; doi:10.1155/2021/8215454)
Supplement: Supplementary Materials — Supplementary Table 1: active compounds of each herb in ZSYTP. Supplementary Table 2: corresponding targets of active compounds: (A) two target genes of SDH; (B) 138 target genes of GQZ; (C) 202 target genes of TSZ; (D) 25 target genes of BJT; (E) 121 target genes of DS; (F) 11 target genes of BZ; (G) 283 target genes of DZ; (H) 13 target genes of SR; (J) 112 target genes of RS; (K) 76 target genes of SJS; (L) 115 target genes of AY; (M) 149 target genes of HSW; (N) 68 target genes of EJ; (P) 46 target genes of LJJ. Supplementary Table 3: POI targets. Supplementary Table 4: compounds and targets attributes of C-T network. Supplementary Table 5: data of compound-target network. Supplementary Table 6: analysed C-T network: (A) active compounds of C-T network and corresponding degrees; (B) targets of C-T network and corresponding degrees. Supplementary Table 7: ZSYTP core PPI network. Supplementary Table 8: gene ontology and KEGG analysis: (A) GO-BP analysis; (B) GO-MF analysis; (C) GO-CC analysis; (D) KEGG pathway enrichment analysis. [file 8215454.f1.zip › 8215454.f1/ST2A SDH Herb Targets.pdf]

Supplementary Table S2A. Corresponding Targets of SDH

Herb Name	Mol ID	Chemical Name	Protein Name	Symbol	Source
Rehmanniae Radix Praeparata (Shu Di Huang, SDH)	MOL000359	Sitosterol			
Rehmanniae Radix Praeparata (Shu Di Huang, SDH)	MOL000449	Stigmasterol	DNA polymerase beta	POLB	PubChem
