# Supplementary material for: Network Pharmacology Approach for Predicting Targets of Zishen Yutai Pills on Premature Ovarian Insufficiency
Source: Evid Based Complement Alternat Med. 2021 Aug 4;2021:8215454. doi: 10.1155/2021/8215454 (PMC8357500; doi:10.1155/2021/8215454)
Supplement: Supplementary Materials — Supplementary Table 1: active compounds of each herb in ZSYTP. Supplementary Table 2: corresponding targets of active compounds: (A) two target genes of SDH; (B) 138 target genes of GQZ; (C) 202 target genes of TSZ; (D) 25 target genes of BJT; (E) 121 target genes of DS; (F) 11 target genes of BZ; (G) 283 target genes of DZ; (H) 13 target genes of SR; (J) 112 target genes of RS; (K) 76 target genes of SJS; (L) 115 target genes of AY; (M) 149 target genes of HSW; (N) 68 target genes of EJ; (P) 46 target genes of LJJ. Supplementary Table 3: POI targets. Supplementary Table 4: compounds and targets attributes of C-T network. Supplementary Table 5: data of compound-target network. Supplementary Table 6: analysed C-T network: (A) active compounds of C-T network and corresponding degrees; (B) targets of C-T network and corresponding degrees. Supplementary Table 7: ZSYTP core PPI network. Supplementary Table 8: gene ontology and KEGG analysis: (A) GO-BP analysis; (B) GO-MF analysis; (C) GO-CC analysis; (D) KEGG pathway enrichment analysis. [file 8215454.f1.zip › 8215454.f1/ST2B GQZ Herb Targets.pdf]

Supplementary Table S2B. Corresponding Targets of GQZ

Herb Name	Mol ID	Chemical Name	Protein Name	Symbol	Sources	
Lycii Fructus (Gou Qi Zi, GQZ)	MOL000358	Beta-sitosterol	DNA polymerase beta	POLB	PubChem	
Lycii Fructus (Gou Qi Zi, GQZ)	MOL000358	Beta-sitosterol	glycogen synthase kinase 3 beta	GSK3B	PubChem	
Lycii Fructus (Gou Qi Zi, GQZ)	MOL000358	Beta-sitosterol	protein kinase AMP-activated non-catalytic subunit	PRKAB2	PubChem	
Lycii Fructus (Gou Qi Zi, GQZ)	MOL000358	Beta-sitosterol	protein kinase AMP-activated non-catalytic subunit	PRKAG3	PubChem	
Lycii Fructus (Gou Qi Zi, GQZ)	MOL000358	Beta-sitosterol	protein kinase AMP-activated non-catalytic subunit	PRKAG1	PubChem	
Lycii Fructus (Gou Qi Zi, GQZ)	MOL000358	Beta-sitosterol	protein kinase AMP-activated catalytic subunit alph	PRKAA1	PubChem	
Lycii Fructus (Gou Qi Zi, GQZ)	MOL000358	Beta-sitosterol	protein kinase AMP-activated catalytic subunit alph	PRKAA2	PubChem	
Lycii Fructus (Gou Qi Zi, GQZ)	MOL000358	Beta-sitosterol	protein kinase AMP-activated non-catalytic subunit	PRKAB1	PubChem	
Lycii Fructus (Gou Qi Zi, GQZ)	MOL000358	Beta-sitosterol	protein kinase AMP-activated non-catalytic subunit	PRKAG2	PubChem	
Lycii Fructus (Gou Qi Zi, GQZ)	MOL000358	Beta-sitosterol	dipeptidyl peptidase 4	DPP4	PubChem	
Lycii Fructus (Gou Qi Zi, GQZ)	MOL000358	Beta-sitosterol	coagulation factor II, thrombin	F2	PubChem	
Lycii Fructus (Gou Qi Zi, GQZ)	MOL000953	CLR	nuclear factor, erythroid 2 like 2	NFE2L2	PubChem	
Lycii Fructus (Gou Qi Zi, GQZ)	MOL000953	CLR	ATP binding cassette subfamily B member 1	ABCB1	PubChem	
Lycii Fructus (Gou Qi Zi, GQZ)	MOL000953	CLR	RAR related orphan receptor A	RORA	PubChem	
Lycii Fructus (Gou Qi Zi, GQZ)	MOL000953	CLR	RAR related orphan receptor C	RORC	PubChem	
Lycii Fructus (Gou Qi Zi, GQZ)	MOL003578	Cycloartenol	APC membrane recruitment protein 1	FAM123B	STITCH	
Lycii Fructus (Gou Qi Zi, GQZ)	MOL003578	Cycloartenol	Lanosterol 14-alpha demethylase	CYP51A1	STITCH	
Lycii Fructus (Gou Qi Zi, GQZ)	MOL001494	Mandenol	peroxisome proliferator activated receptor gamma	PPARG	PubChem	
Lycii Fructus (Gou Qi Zi, GQZ)	MOL001494	Mandenol	heat shock protein family B (small) member 1	HSPB1	PubChem	
Lycii Fructus (Gou Qi Zi, GQZ)	MOL001494	Mandenol	retinoic acid receptor alpha	RARA	PubChem	
Lycii Fructus (Gou Qi Zi, GQZ)	MOL001494	Mandenol	G protein-coupled receptor 32	GPR32	PubChem	
Lycii Fructus (Gou Qi Zi, GQZ)	MOL001494	Mandenol	nuclear receptor subfamily 1, group I, member 2	NR1I2	PubChem	
Lycii Fructus (Gou Qi Zi, GQZ)	MOL001494	Mandenol	nuclear factor, erythroid 2 like 2	NFE2L2	PubChem	
Lycii Fructus (Gou Qi Zi, GQZ)	MOL001494	Mandenol	peroxisome proliferator activated receptor delta	PPARD	PubChem	
Lycii Fructus (Gou Qi Zi, GQZ)	MOL001494	Mandenol	vitamin D receptor	VDR	PubChem	
Lycii Fructus (Gou Qi Zi, GQZ)	MOL001979	LAN	cytochrome P450 family 51 subfamily A member 1	CYP51A1	PubChem	
Lycii Fructus (Gou Qi Zi, GQZ)	MOL001979	LAN	crystallin alpha A	CRYAA	PubChem	
Lycii Fructus (Gou Qi Zi, GQZ)	MOL001979	LAN	crystallin beta B2	CRYBB2	PubChem	
Lycii Fructus (Gou Qi Zi, GQZ)	MOL001979	LAN	crystallin gamma C	CRYGC	PubChem	
Lycii Fructus (Gou Qi Zi, GQZ)	MOL001979	LAN	crystallin gamma D	CRYGD	PubChem	
Lycii Fructus (Gou Qi Zi, GQZ)	MOL001979	LAN	crystallin alpha B	CRYAB	PubChem	
Lycii Fructus (Gou Qi Zi, GQZ)	MOL001979	LAN	Lanosterol synthase	LSS	PubChem	
Lycii Fructus (Gou Qi Zi, GQZ)	MOL009650	Atropine	Muscarinic acetylcholine receptor M3	CHRM3	STITCH	Swiss Target Prediction
Lycii Fructus (Gou Qi Zi, GQZ)	MOL009650	Atropine	Muscarinic acetylcholine receptor M1	CHRM1	STITCH	Swiss Target Prediction
Lycii Fructus (Gou Qi Zi, GQZ)	MOL009650	Atropine	Muscarinic acetylcholine receptor M4	CHRM4	STITCH	Swiss Target Prediction
Lycii Fructus (Gou Qi Zi, GQZ)	MOL009650	Atropine	Muscarinic acetylcholine receptor M5	CHRM5	STITCH	Swiss Target Prediction
Lycii Fructus (Gou Qi Zi, GQZ)	MOL009650	Atropine	Muscarinic acetylcholine receptor M2	CHRM2	STITCH	Swiss Target Prediction
Lycii Fructus (Gou Qi Zi, GQZ)	MOL009650	Atropine	Armadillo repeat-containing protein 3	ARMC3	STITCH	
Lycii Fructus (Gou Qi Zi, GQZ)	MOL009650	Atropine	Histamine H1 receptor	HRH1	STITCH	
Lycii Fructus (Gou Qi Zi, GQZ)	MOL009650	Atropine	Armadillo repeat-containing X-linked protein 3	ARMCX3	STITCH	
Lycii Fructus (Gou Qi Zi, GQZ)	MOL009650	Atropine	Junction plakoglobin	JUP	STITCH	
Lycii Fructus (Gou Qi Zi, GQZ)	MOL009650	Atropine	Cholinesterase	BCHE	STITCH	
Lycii Fructus (Gou Qi Zi, GQZ)	MOL009650	Atropine	lysine acetyltransferase 2A	KAT2A	PubChem	
Lycii Fructus (Gou Qi Zi, GQZ)	MOL009650	Atropine	Acetylcholinesterase (by homology)	ACHE	Swiss Target Prediction	
Lycii Fructus (Gou Qi Zi, GQZ)	MOL009650	Atropine	Alpha-1d adrenergic receptor	ADRA1D	Swiss Target Prediction	
Lycii Fructus (Gou Qi Zi, GQZ)	MOL009650	Atropine	Serotonin 2c (5-HT2c) receptor	HTR2C	Swiss Target Prediction	
Lycii Fructus (Gou Qi Zi, GQZ)	MOL000449	Stigmasterol	DNA polymerase beta	POLB	PubChem	
Lycii Fructus (Gou Qi Zi, GQZ)	MOL001495	Ethyl linolenate	cytochrome P450 family 3 subfamily A member 4	CYP3A4	PubChem	
Lycii Fructus (Gou Qi Zi, GQZ)	MOL001495	Ethyl linolenate	arachidonate 15-lipoxygenase	ALOX15	PubChem	
Lycii Fructus (Gou Qi Zi, GQZ)	MOL001495	Ethyl linolenate	aldehyde dehydrogenase 1 family member A1	ALDH1A1*	PubChem	
Lycii Fructus (Gou Qi Zi, GQZ)	MOL001495	Ethyl linolenate	nuclear factor, erythroid 2 like 2	NFE2L2	PubChem	
Lycii Fructus (Gou Qi Zi, GQZ)	MOL001495	Ethyl linolenate	nuclear receptor subfamily 3 group C member 1	NR3C1	PubChem	
Lycii Fructus (Gou Qi Zi, GQZ)	MOL001495	Ethyl linolenate	GLI family zinc finger 3	GLI3	PubChem	
Lycii Fructus (Gou Qi Zi, GQZ)	MOL001495	Ethyl linolenate	estrogen receptor 2	ESR2	PubChem	
Lycii Fructus (Gou Qi Zi, GQZ)	MOL001495	Ethyl linolenate	estrogen related receptor alpha	ESRRA	PubChem	
Lycii Fructus (Gou Qi Zi, GQZ)	MOL000098	Quercetin	Induced myeloid leukemia cell differentiation protein M	MCL1	STITCH	
Lycii Fructus (Gou Qi Zi, GQZ)	MOL000098	Quercetin	Tyrosine-protein kinase HCK	HCK	STITCH	
Lycii Fructus (Gou Qi Zi, GQZ)	MOL000098	Quercetin	Solute carrier family 2, facilitated glucose transporter m	SLC2A2	STITCH	
Lycii Fructus (Gou Qi Zi, GQZ)	MOL000098	Quercetin	Cytochrome P450 2C8	CYP2C8	STITCH	
Lycii Fructus (Gou Qi Zi, GQZ)	MOL000098	Quercetin	ATP synthase subunit beta, mitochondrial	ATP5B	STITCH	
Lycii Fructus (Gou Qi Zi, GQZ)	MOL000098	Quercetin	3-hydroxyisobutyryl-CoA hydrolase, mitochondrial	HIBCH	STITCH	
Lycii Fructus (Gou Qi Zi, GQZ)	MOL000098	Quercetin	serine/threonine kinase 17b	STK17B	STITCH	
Lycii Fructus (Gou Qi Zi, GQZ)	MOL000098	Quercetin	NADPH oxidase 4	NOX4	Swiss Target Prediction	
Lycii Fructus (Gou Qi Zi, GQZ)	MOL000098	Quercetin	Vasopressin V2 receptor	AVPR2	Swiss Target Prediction	
Lycii Fructus (Gou Qi Zi, GQZ)	MOL000098	Quercetin	Aldose reductase	AKR1B1	Swiss Target Prediction	
Lycii Fructus (Gou Qi Zi, GQZ)	MOL000098	Quercetin	Xanthine dehydrogenase	XDH	Swiss Target Prediction	
Lycii Fructus (Gou Qi Zi, GQZ)	MOL000098	Quercetin	Monoamine oxidase A	MAOA	Swiss Target Prediction	
Lycii Fructus (Gou Qi Zi, GQZ)	MOL000098	Quercetin	Insulin-like growth factor I receptor	IGF1R	Swiss Target Prediction	
Lycii Fructus (Gou Qi Zi, GQZ)	MOL000098	Quercetin	Tyrosine-protein kinase receptor FLT3	FLT3	Swiss Target Prediction	
Lycii Fructus (Gou Qi Zi, GQZ)	MOL000098	Quercetin	Cytochrome P450 19A1	CYP19A1	Swiss Target Prediction	
Lycii Fructus (Gou Qi Zi, GQZ)	MOL000098	Quercetin	Epidermal growth factor receptor erbB1	EGFR	Swiss Target Prediction	
Lycii Fructus (Gou Qi Zi, GQZ)	MOL000098	Quercetin	Thrombin	F2	Swiss Target Prediction	
Lycii Fructus (Gou Qi Zi, GQZ)	MOL000098	Quercetin	Carbonic anhydrase II	CA2	Swiss Target Prediction	
Lycii Fructus (Gou Qi Zi, GQZ)	MOL000098	Quercetin	Serine/threonine-protein kinase PIM1	PIM1	Swiss Target Prediction	STITCH
Lycii Fructus (Gou Qi Zi, GQZ)	MOL000098	Quercetin	Arachidonate 5-lipoxygenase	ALOX5	Swiss Target Prediction	
Lycii Fructus (Gou Qi Zi, GQZ)	MOL000098	Quercetin	Serine/threonine-protein kinase Aurora-B	AURKB	Swiss Target Prediction	
Lycii Fructus (Gou Qi Zi, GQZ)	MOL000098	Quercetin	Dopamine D4 receptor	DRD4	Swiss Target Prediction	
Lycii Fructus (Gou Qi Zi, GQZ)	MOL000098	Quercetin	Adenosine A1 receptor (by homology)	ADORA1	Swiss Target Prediction	
Lycii Fructus (Gou Qi Zi, GQZ)	MOL000098	Quercetin	Carbonic anhydrase VII	CA7	Swiss Target Prediction	
Lycii Fructus (Gou Qi Zi, GQZ)	MOL000098	Quercetin	Glyoxalase I	GLO1	Swiss Target Prediction	
Lycii Fructus (Gou Qi Zi, GQZ)	MOL000098	Quercetin	Myeloperoxidase	MPO	Swiss Target Prediction	
Lycii Fructus (Gou Qi Zi, GQZ)	MOL000098	Quercetin	PI3-kinase p85-alpha subunit	PIK3R1	Swiss Target Prediction	
Lycii Fructus (Gou Qi Zi, GQZ)	MOL000098	Quercetin	Adenosine A2a receptor (by homology)	ADORA2A	Swiss Target Prediction	
Lycii Fructus (Gou Qi Zi, GQZ)	MOL000098	Quercetin	Death-associated protein kinase 1	DAPK1	Swiss Target Prediction	
Lycii Fructus (Gou Qi Zi, GQZ)	MOL000098	Quercetin	Liver glycogen phosphorylase	PYGL	Swiss Target Prediction	
Lycii Fructus (Gou Qi Zi, GQZ)	MOL000098	Quercetin	Carbonic anhydrase I	CA1	Swiss Target Prediction	
Lycii Fructus (Gou Qi Zi, GQZ)	MOL000098	Quercetin	Glycogen synthase kinase-3 beta	GSK3B	Swiss Target Prediction	
Lycii Fructus (Gou Qi Zi, GQZ)	MOL000098	Quercetin	Tyrosine-protein kinase SRC	SRC	Swiss Target Prediction	
Lycii Fructus (Gou Qi Zi, GQZ)	MOL000098	Quercetin	Focal adhesion kinase 1	PTK2	Swiss Target Prediction	
Lycii Fructus (Gou Qi Zi, GQZ)	MOL000098	Quercetin	Estradiol 17-beta-dehydrogenase 2	HSD17B2	Swiss Target Prediction	
Lycii Fructus (Gou Qi Zi, GQZ)	MOL000098	Quercetin	Vascular endothelial growth factor receptor 2	KDR	Swiss Target Prediction	
Lycii Fructus (Gou Qi Zi, GQZ)	MOL000098	Quercetin	Matrix metalloproteinase 13	MMP13	Swiss Target Prediction	
Lycii Fructus (Gou Qi Zi, GQZ)	MOL000098	Quercetin	Matrix metalloproteinase 3	MMP3	Swiss Target Prediction	
Lycii Fructus (Gou Qi Zi, GQZ)	MOL000098	Quercetin	Carbonic anhydrase III	CA3	Swiss Target Prediction	
Lycii Fructus (Gou Qi Zi, GQZ)	MOL000098	Quercetin	Arachidonate 15-lipoxygenase	ALOX15	Swiss Target Prediction	
Lycii Fructus (Gou Qi Zi, GQZ)	MOL000098	Quercetin	Multidrug resistance-associated protein 1	ABCC1	Swiss Target Prediction	
Lycii Fructus (Gou Qi Zi, GQZ)	MOL000098	Quercetin	Serine/threonine-protein kinase PLK1	PLK1	Swiss Target Prediction	
Lycii Fructus (Gou Qi Zi, GQZ)	MOL000098	Quercetin	Carbonic anhydrase VI	CA6	Swiss Target Prediction	
Lycii Fructus (Gou Qi Zi, GQZ)	MOL000098	Quercetin	Cyclin-dependent kinase 1	CDK1	Swiss Target Prediction	
Lycii Fructus (Gou Qi Zi, GQZ)	MOL000098	Quercetin	Matrix metalloproteinase 9	MMP9	Swiss Target Prediction	
Lycii Fructus (Gou Qi Zi, GQZ)	MOL000098	Quercetin	Carbonic anhydrase XII	CA12	Swiss Target Prediction	
Lycii Fructus (Gou Qi Zi, GQZ)	MOL000098	Quercetin	Matrix metalloproteinase 2	MMP2	Swiss Target Prediction	
Lycii Fructus (Gou Qi Zi, GQZ)	MOL000098	Quercetin	Protein kinase N1	PKN1	Swiss Target Prediction	
Lycii Fructus (Gou Qi Zi, GQZ)	MOL000098	Quercetin	Carbonic anhydrase XIV	CA14	Swiss Target Prediction	
Lycii Fructus (Gou Qi Zi, GQZ)	MOL000098	Quercetin	Carbonic anhydrase IX	CA9	Swiss Target Prediction	
Lycii Fructus (Gou Qi Zi, GQZ)	MOL000098	Quercetin	Casein kinase II alpha	CSNK2A1	Swiss Target Prediction	
Lycii Fructus (Gou Qi Zi, GQZ)	MOL000098	Quercetin	Arachidonate 12-lipoxygenase	ALOX12	Swiss Target Prediction	
Lycii Fructus (Gou Qi Zi, GQZ)	MOL000098	Quercetin	Hepatocyte growth factor receptor	MET	Swiss Target Prediction	
Lycii Fructus (Gou Qi Zi, GQZ)	MOL000098	Quercetin	Carbonic anhydrase IV	CA4	Swiss Target Prediction	
Lycii Fructus (Gou Qi Zi, GQZ)	MOL000098	Quercetin	Serine/threonine-protein kinase NEK2	NEK2	Swiss Target Prediction	
Lycii Fructus (Gou Qi Zi, GQZ)	MOL000098	Quercetin	Interleukin-8 receptor A	CXCR1	Swiss Target Prediction	
Lycii Fructus (Gou Qi Zi, GQZ)	MOL000098	Quercetin	CaM kinase II beta	CAMK2B	Swiss Target Prediction	
Lycii Fructus (Gou Qi Zi, GQZ)	MOL000098	Quercetin	ALK tyrosine kinase receptor	ALK	Swiss Target Prediction	
Lycii Fructus (Gou Qi Zi, GQZ)	MOL000098	Quercetin	Serine/threonine-protein kinase AKT	AKT1	Swiss Target Prediction	
Lycii Fructus (Gou Qi Zi, GQZ)	MOL000098	Quercetin	P-glycoprotein 1	ABCB1	Swiss Target Prediction	
Lycii Fructus (Gou Qi Zi, GQZ)	MOL000098	Quercetin	Serine/threonine-protein kinase NEK6	NEK6	Swiss Target Prediction	
Lycii Fructus (Gou Qi Zi, GQZ)	MOL000098	Quercetin	Phospholipase A2 group 1B	PLA2G1B	Swiss Target Prediction	
Lycii Fructus (Gou Qi Zi, GQZ)	MOL000098	Quercetin	Carbonic anhydrase VA	CA5A	Swiss Target Prediction	
Lycii Fructus (Gou Qi Zi, GQZ)	MOL000098	Quercetin	Beta-secretase 1	BACE1	Swiss Target Prediction	
Lycii Fructus (Gou Qi Zi, GQZ)	MOL000098	Quercetin	Cytochrome P450 1B1	CYP1B1	Swiss Target Prediction	STITCH
Lycii Fructus (Gou Qi Zi, GQZ)	MOL000098	Quercetin	Tyrosine-protein kinase receptor UFO	AXL	Swiss Target Prediction	
Lycii Fructus (Gou Qi Zi, GQZ)	MOL000098	Quercetin	ATP-binding cassette sub-family G member 2	ABCG2	Swiss Target Prediction	
Lycii Fructus (Gou Qi Zi, GQZ)	MOL000098	Quercetin	NUAK family SNF1-like kinase 1	NUAK1	Swiss Target Prediction	
Lycii Fructus (Gou Qi Zi, GQZ)	MOL000098	Quercetin	Aldo-keto reductase family 1 member C2 (by homology)	AKR1C2	Swiss Target Prediction	
Lycii Fructus (Gou Qi Zi, GQZ)	MOL000098	Quercetin	Aldo-keto reductase family 1 member C1 (by homology)	AKR1C1	Swiss Target Prediction	
Lycii Fructus (Gou Qi Zi, GQZ)	MOL000098	Quercetin	Aldo-keto reductase family 1 member C3 (by homology)	AKR1C3	Swiss Target Prediction	
Lycii Fructus (Gou Qi Zi, GQZ)	MOL000098	Quercetin	Aldo-keto reductase family 1 member C4 (by homology)	AKR1C4	Swiss Target Prediction	
Lycii Fructus (Gou Qi Zi, GQZ)	MOL000098	Quercetin	Carbonic anhydrase XIII (by homology)	CA13	Swiss Target Prediction	
Lycii Fructus (Gou Qi Zi, GQZ)	MOL000098	Quercetin	Aldehyde reductase (by homology)	AKR1A1	Swiss Target Prediction	
Lycii Fructus (Gou Qi Zi, GQZ)	MOL000098	Quercetin	G-protein coupled receptor 35	GPR35	Swiss Target Prediction	
Lycii Fructus (Gou Qi Zi, GQZ)	MOL000098	Quercetin	Tyrosine-protein kinase SYK	SYK	Swiss Target Prediction	
Lycii Fructus (Gou Qi Zi, GQZ)	MOL008400	Glycitein	Transcription factor AP-1	JUN	STITCH	
Lycii Fructus (Gou Qi Zi, GQZ)	MOL008400	Glycitein	Mitogen-activated protein kinase 3	MAPK3	STITCH	
Lycii Fructus (Gou Qi Zi, GQZ)	MOL008400	Glycitein	Transcription factor Sp1	SP1	STITCH	
Lycii Fructus (Gou Qi Zi, GQZ)	MOL008400	Glycitein	Collagenase 3	MMP13	STITCH	
Lycii Fructus (Gou Qi Zi, GQZ)	MOL008400	Glycitein	Mitogen-activated protein kinase 1	MAPK1	STITCH	
Lycii Fructus (Gou Qi Zi, GQZ)	MOL008400	Glycitein	Cyclin-dependent kinase 4	CDK4	STITCH	
Lycii Fructus (Gou Qi Zi, GQZ)	MOL008400	Glycitein	Estrogen Receptor 1	ESR1	Pubchem	
