# Supplementary material for: Network Pharmacology Approach for Predicting Targets of Zishen Yutai Pills on Premature Ovarian Insufficiency
Source: Evid Based Complement Alternat Med. 2021 Aug 4;2021:8215454. doi: 10.1155/2021/8215454 (PMC8357500; doi:10.1155/2021/8215454)
Supplement: Supplementary Materials — Supplementary Table 1: active compounds of each herb in ZSYTP. Supplementary Table 2: corresponding targets of active compounds: (A) two target genes of SDH; (B) 138 target genes of GQZ; (C) 202 target genes of TSZ; (D) 25 target genes of BJT; (E) 121 target genes of DS; (F) 11 target genes of BZ; (G) 283 target genes of DZ; (H) 13 target genes of SR; (J) 112 target genes of RS; (K) 76 target genes of SJS; (L) 115 target genes of AY; (M) 149 target genes of HSW; (N) 68 target genes of EJ; (P) 46 target genes of LJJ. Supplementary Table 3: POI targets. Supplementary Table 4: compounds and targets attributes of C-T network. Supplementary Table 5: data of compound-target network. Supplementary Table 6: analysed C-T network: (A) active compounds of C-T network and corresponding degrees; (B) targets of C-T network and corresponding degrees. Supplementary Table 7: ZSYTP core PPI network. Supplementary Table 8: gene ontology and KEGG analysis: (A) GO-BP analysis; (B) GO-MF analysis; (C) GO-CC analysis; (D) KEGG pathway enrichment analysis. [file 8215454.f1.zip › 8215454.f1/ST2D BJT Herb Targets.pdf]

Supplementary Table S2D. Corresponding Targets of BJT

Herb Name	Mol ID	Chemical Name	Protein Name	Symbol	Sources	
Morindae Officinalis Radix (Ba Ji Tian, BJT)	MOL001506	Supraene	farnesyltransferase, CAAX box, alpha	FNTA	STITCH	
Morindae Officinalis Radix (Ba Ji Tian, BJT)	MOL001506	Supraene	GDP dissociation inhibitor 1	GDI1	STITCH	
Morindae Officinalis Radix (Ba Ji Tian, BJT)	MOL001506	Supraene	farnesyltransferase, CAAX box, beta	FNTB	STITCH	
Morindae Officinalis Radix (Ba Ji Tian, BJT)	MOL001506	Supraene	Rho GDP dissociation inhibitor (GDI) alpha	ARHGDIA	STITCH	
Morindae Officinalis Radix (Ba Ji Tian, BJT)	MOL001506	Supraene	Ras-related C3 botulinum toxin substrate 1 (rho fa	RAC1	STITCH	
Morindae Officinalis Radix (Ba Ji Tian, BJT)	MOL001506	Supraene	cell division cycle 42 (GTP binding protein, 25kDa)	CDC42	STITCH	
Morindae Officinalis Radix (Ba Ji Tian, BJT)	MOL001506	Supraene	protein geranylgeranyltransferase type I, beta sul	PGGT1B	STITCH	
Morindae Officinalis Radix (Ba Ji Tian, BJT)	MOL001506	Supraene	Rab geranylgeranyltransferase, beta subunit	RABGGTB	STITCH	
Morindae Officinalis Radix (Ba Ji Tian, BJT)	MOL001506	Supraene	RAB7A, member RAS oncogene family	RAB7A	STITCH	
Morindae Officinalis Radix (Ba Ji Tian, BJT)	MOL001506	Supraene	Rab geranylgeranyltransferase, alpha subunit	RABGGTA	STITCH	
Morindae Officinalis Radix (Ba Ji Tian, BJT)	MOL000358	Beta-sitosterol	DNA polymerase beta	POLB	PubChem	
Morindae Officinalis Radix (Ba Ji Tian, BJT)	MOL000358	Beta-sitosterol	glycogen synthase kinase 3 beta	GSK3B	PubChem	
Morindae Officinalis Radix (Ba Ji Tian, BJT)	MOL000358	Beta-sitosterol	protein kinase AMP-activated non-catalytic subur	PRKAB2	PubChem	
Morindae Officinalis Radix (Ba Ji Tian, BJT)	MOL000358	Beta-sitosterol	protein kinase AMP-activated non-catalytic subur	PRKAG3	PubChem	
Morindae Officinalis Radix (Ba Ji Tian, BJT)	MOL000358	Beta-sitosterol	protein kinase AMP-activated non-catalytic subur	PRKAG1	PubChem	
Morindae Officinalis Radix (Ba Ji Tian, BJT)	MOL000358	Beta-sitosterol	protein kinase AMP-activated catalytic subunit al	PRKAA1	PubChem	
Morindae Officinalis Radix (Ba Ji Tian, BJT)	MOL000358	Beta-sitosterol	protein kinase AMP-activated catalytic subunit al	PRKAA2	PubChem	
Morindae Officinalis Radix (Ba Ji Tian, BJT)	MOL000358	Beta-sitosterol	protein kinase AMP-activated non-catalytic subur	PRKAB1	PubChem	
Morindae Officinalis Radix (Ba Ji Tian, BJT)	MOL000358	Beta-sitosterol	protein kinase AMP-activated non-catalytic subur	PRKAG2	PubChem	
Morindae Officinalis Radix (Ba Ji Tian, BJT)	MOL000358	Beta-sitosterol	dipeptidyl peptidase 4	DPP4	PubChem	
Morindae Officinalis Radix (Ba Ji Tian, BJT)	MOL000358	Beta-sitosterol	coagulation factor II, thrombin	F2	PubChem	
Morindae Officinalis Radix (Ba Ji Tian, BJT)	MOL009562	Ohioensin-A	protein tyrosine phosphatase non-receptor type 1	PTPN1	PubChem	Swiss Target Prediction
Morindae Officinalis Radix (Ba Ji Tian, BJT)	MOL002879	Diop	Androgen Receptor	AR	PubChem	
Morindae Officinalis Radix (Ba Ji Tian, BJT)	MOL002879	Diop	GLI family zinc finger 3	GLI3	PubChem	
Morindae Officinalis Radix (Ba Ji Tian, BJT)	MOL002879	Diop	nuclear receptor subfamily 1, group I, member 2	NR1I2	PubChem	
