# Supplementary material for: Network Pharmacology Approach for Predicting Targets of Zishen Yutai Pills on Premature Ovarian Insufficiency
Source: Evid Based Complement Alternat Med. 2021 Aug 4;2021:8215454. doi: 10.1155/2021/8215454 (PMC8357500; doi:10.1155/2021/8215454)
Supplement: Supplementary Materials — Supplementary Table 1: active compounds of each herb in ZSYTP. Supplementary Table 2: corresponding targets of active compounds: (A) two target genes of SDH; (B) 138 target genes of GQZ; (C) 202 target genes of TSZ; (D) 25 target genes of BJT; (E) 121 target genes of DS; (F) 11 target genes of BZ; (G) 283 target genes of DZ; (H) 13 target genes of SR; (J) 112 target genes of RS; (K) 76 target genes of SJS; (L) 115 target genes of AY; (M) 149 target genes of HSW; (N) 68 target genes of EJ; (P) 46 target genes of LJJ. Supplementary Table 3: POI targets. Supplementary Table 4: compounds and targets attributes of C-T network. Supplementary Table 5: data of compound-target network. Supplementary Table 6: analysed C-T network: (A) active compounds of C-T network and corresponding degrees; (B) targets of C-T network and corresponding degrees. Supplementary Table 7: ZSYTP core PPI network. Supplementary Table 8: gene ontology and KEGG analysis: (A) GO-BP analysis; (B) GO-MF analysis; (C) GO-CC analysis; (D) KEGG pathway enrichment analysis. [file 8215454.f1.zip › 8215454.f1/ST2E DS Herb Targets.pdf]

Supplementary Table S2E. Corresponding Targets of DS

Herb Name	Mol ID	Chemical Name	Protein Name	Symbol		Sources
Codonopsis Radix (Dang Shen, DS)	MOL000006	Luteolin	mitogen-activated protein kinase 8	MAPK8	STITCH	
Codonopsis Radix (Dang Shen, DS)	MOL000006	Luteolin	matrix metallopeptidase 9 (gelatinase B, 92kDa	MMP9	STITCH	Swiss Target Prediction
Codonopsis Radix (Dang Shen, DS)	MOL000006	Luteolin	caspase 3, apoptosis-related cysteine peptidase	CASP3	STITCH	
Codonopsis Radix (Dang Shen, DS)	MOL000006	Luteolin	jun proto-oncogene	JUN	STITCH	
Codonopsis Radix (Dang Shen, DS)	MOL000006	Luteolin	FBJ murine osteosarcoma viral oncogene homol	FOS	STITCH	
Codonopsis Radix (Dang Shen, DS)	MOL000006	Luteolin	cyclin-dependent kinase 2	CDK2	STITCH	
Codonopsis Radix (Dang Shen, DS)	MOL000006	Luteolin	epidermal growth factor receptor (1210 aa)	EGFR	STITCH	
Codonopsis Radix (Dang Shen, DS)	MOL000006	Luteolin	SMAD family member 2	SMAD2	STITCH	
Codonopsis Radix (Dang Shen, DS)	MOL000006	Luteolin	cyclin A2	CCNA2	STITCH	
Codonopsis Radix (Dang Shen, DS)	MOL000006	Luteolin	v-akt murine thymoma viral oncogene homolog	AKT1	STITCH	
Codonopsis Radix (Dang Shen, DS)	MOL000006	Luteolin	15-hydroxyprostaglandin dehydrogenase	HPGD	PubChem	
Codonopsis Radix (Dang Shen, DS)	MOL000006	Luteolin	Cytochrome P450 Family 1 Subfamily A Membe	CYP1A2	PubChem	
Codonopsis Radix (Dang Shen, DS)	MOL000006	Luteolin	casein kinase 1 gamma 2	CSNK1G2	PubChem	
Codonopsis Radix (Dang Shen, DS)	MOL000006	Luteolin	CDC like kinase 3	CLK3	PubChem	
Codonopsis Radix (Dang Shen, DS)	MOL000006	Luteolin	CDC Like Kinase 1	CLK1	PubChem	
Codonopsis Radix (Dang Shen, DS)	MOL000006	Luteolin	serine/threonine kinase 16	STK16	PubChem	
Codonopsis Radix (Dang Shen, DS)	MOL000006	Luteolin	Pim-2 proto-oncogene, serine/threonine kinase	PIM2	PubChem	
Codonopsis Radix (Dang Shen, DS)	MOL000006	Luteolin	carbonyl reductase 3	CBR3	PubChem	
Codonopsis Radix (Dang Shen, DS)	MOL000006	Luteolin	arachidonate 15-lipoxygenase type B	ALOX15B	PubChem	
Codonopsis Radix (Dang Shen, DS)	MOL000006	Luteolin	Cytochrome P450 3A4	CYP3A4	PubChem	
Codonopsis Radix (Dang Shen, DS)	MOL000006	Luteolin	arachidonate 15-lipoxygenase	ALOX15	PubChem	
Codonopsis Radix (Dang Shen, DS)	MOL000006	Luteolin	MCL1 apoptosis regulator, BCL2 family member	MCL1	PubChem	
Codonopsis Radix (Dang Shen, DS)	MOL000006	Luteolin	aldehyde dehydrogenase 1 family member A1	ALDH1A1	PubChem	
Codonopsis Radix (Dang Shen, DS)	MOL000006	Luteolin	survival of motor neuron 2, centromeric	SMN2	PubChem	
Codonopsis Radix (Dang Shen, DS)	MOL000006	Luteolin	Microtubule Associated Protein Tau	MAPT	PubChem	
Codonopsis Radix (Dang Shen, DS)	MOL000006	Luteolin	Cytochrome P450 2D6	CYP2D6	PubChem	
Codonopsis Radix (Dang Shen, DS)	MOL000006	Luteolin	Cytochrome P450 2C19	CYP2C19	PubChem	
Codonopsis Radix (Dang Shen, DS)	MOL000006	Luteolin	Cytochrome P450 family 2 subfamily C member	CYP2C9	PubChem	
Codonopsis Radix (Dang Shen, DS)	MOL000006	Luteolin	glutathione S-transferase omega 1	GSTO1	PubChem	
Codonopsis Radix (Dang Shen, DS)	MOL000006	Luteolin	glucosidase alpha, acid	GAA	PubChem	
Codonopsis Radix (Dang Shen, DS)	MOL000006	Luteolin	BLM RecQ like helicase	BLM	PubChem	
Codonopsis Radix (Dang Shen, DS)	MOL000006	Luteolin	alkaline phosphatase, intestinal	ALPI	PubChem	
Codonopsis Radix (Dang Shen, DS)	MOL000006	Luteolin	tyrosinase	TYR	PubChem	
Codonopsis Radix (Dang Shen, DS)	MOL000006	Luteolin	glycogen synthase kinase 3 alpha	GSK3A	PubChem	
Codonopsis Radix (Dang Shen, DS)	MOL000006	Luteolin	glycogen synthase kinase 3 beta	GSK3B	PubChem	Swiss Target Prediction
Codonopsis Radix (Dang Shen, DS)	MOL000006	Luteolin	cyclin B2	CCNB2	PubChem	
Codonopsis Radix (Dang Shen, DS)	MOL000006	Luteolin	Cyclin-dependent kinase 1	CDK1	PubChem	
Codonopsis Radix (Dang Shen, DS)	MOL000006	Luteolin	cyclin B1	CCNB1	PubChem	
Codonopsis Radix (Dang Shen, DS)	MOL000006	Luteolin	cyclin B3	CCNB3	PubChem	
Codonopsis Radix (Dang Shen, DS)	MOL000006	Luteolin	cyclin dependent kinase 5	CDK5	PubChem	
Codonopsis Radix (Dang Shen, DS)	MOL000006	Luteolin	cyclin dependent kinase 5 regulatory subunit 1	CDK5R1	PubChem	
Codonopsis Radix (Dang Shen, DS)	MOL000006	Luteolin	glyoxalase I	GLO1	PubChem	Swiss Target Prediction
Codonopsis Radix (Dang Shen, DS)	MOL000006	Luteolin	xanthine dehydrogenase	XDH	PubChem	Swiss Target Prediction
Codonopsis Radix (Dang Shen, DS)	MOL000006	Luteolin	nuclear receptor subfamily 3 group C member 1	NR3C1	PubChem	
Codonopsis Radix (Dang Shen, DS)	MOL000006	Luteolin	androgen receptor	AR	PubChem	
Codonopsis Radix (Dang Shen, DS)	MOL000006	Luteolin	monoamine oxidase A	MAOA	PubChem	Swiss Target Prediction
Codonopsis Radix (Dang Shen, DS)	MOL000006	Luteolin	DNA polymerase beta	POLB	PubChem	
Codonopsis Radix (Dang Shen, DS)	MOL000006	Luteolin	platelet activating factor acetylhydrolase 1b cat	PAFAH1B2	PubChem	
Codonopsis Radix (Dang Shen, DS)	MOL000006	Luteolin	platelet activating factor acetylhydrolase 1b cat	PAFAH1B3	PubChem	
Codonopsis Radix (Dang Shen, DS)	MOL000006	Luteolin	cytochrome P450 family 1 subfamily A member	CYP1A1	PubChem	
Codonopsis Radix (Dang Shen, DS)	MOL000006	Luteolin	Cytochrome P450 1B1	CYP1B1	PubChem	Swiss Target Prediction
Codonopsis Radix (Dang Shen, DS)	MOL000006	Luteolin	bromodomain adjacent to zinc finger domain 2B	BAZ2B	PubChem	
Codonopsis Radix (Dang Shen, DS)	MOL000006	Luteolin	NADPH oxidase 4	NOX4	PubChem	Swiss Target Prediction
Codonopsis Radix (Dang Shen, DS)	MOL000006	Luteolin	frataxin	FXN	PubChem	
Codonopsis Radix (Dang Shen, DS)	MOL000006	Luteolin	TNF receptor superfamily member 10b	TNFRSF10B	PubChem	
Codonopsis Radix (Dang Shen, DS)	MOL000006	Luteolin	ATP-binding cassette super-family G member 2	ABCG2	PubChem	Swiss Target Prediction
Codonopsis Radix (Dang Shen, DS)	MOL000006	Luteolin	DNA polymerase kappa	POLK	PubChem	
Codonopsis Radix (Dang Shen, DS)	MOL000006	Luteolin	DNA Polymerase Iota	POLI	PubChem	
Codonopsis Radix (Dang Shen, DS)	MOL000006	Luteolin	DNA Polymerase Eta	POLH	PubChem	
Codonopsis Radix (Dang Shen, DS)	MOL000006	Luteolin	flap structure-specific endonuclease 1	FEN1	PubChem	
Codonopsis Radix (Dang Shen, DS)	MOL000006	Luteolin	CD38 molecule	CD38	PubChem	Swiss Target Prediction
Codonopsis Radix (Dang Shen, DS)	MOL000006	Luteolin	WRN RecQ like helicase	WRN	PubChem	
Codonopsis Radix (Dang Shen, DS)	MOL000006	Luteolin	T cell receptor alpha locus	TRA	PubChem	
Codonopsis Radix (Dang Shen, DS)	MOL000006	Luteolin	dopamine receptor D3	DRD3	PubChem	
Codonopsis Radix (Dang Shen, DS)	MOL000006	Luteolin	phosphatidylinositol-5-phosphate 4-kinase type	PIP4K2A	PubChem	
Codonopsis Radix (Dang Shen, DS)	MOL000006	Luteolin	matrix metallopeptidase 2	MMP2	PubChem	Swiss Target Prediction
Codonopsis Radix (Dang Shen, DS)	MOL000006	Luteolin	matrix metallopeptidase 3	MMP3	PubChem	
Codonopsis Radix (Dang Shen, DS)	MOL000006	Luteolin	matrix metallopeptidase 12	MMP12	PubChem	Swiss Target Prediction
Codonopsis Radix (Dang Shen, DS)	MOL000006	Luteolin	matrix metallopeptidase 13	MMP13	PubChem	
Codonopsis Radix (Dang Shen, DS)	MOL000006	Luteolin	G protein-coupled receptor 35	GPR35	PubChem	
Codonopsis Radix (Dang Shen, DS)	MOL000006	Luteolin	ATP binding cassette subfamily B member 1	ABCB1	PubChem	
Codonopsis Radix (Dang Shen, DS)	MOL000006	Luteolin	spleen associated tyrosine kinase	SYK	PubChem	Swiss Target Prediction
Codonopsis Radix (Dang Shen, DS)	MOL000006	Luteolin	cystic fibrosis transmembrane conductance regu	CFTR	PubChem	
Codonopsis Radix (Dang Shen, DS)	MOL000006	Luteolin	Rap guanine nucleotide exchange factor 3	RAPGEF3	PubChem	
Codonopsis Radix (Dang Shen, DS)	MOL000006	Luteolin	poly(ADP-ribose) polymerase 1	PARP1	PubChem	Swiss Target Prediction
Codonopsis Radix (Dang Shen, DS)	MOL000006	Luteolin	fms related tyrosine kinase 3	FLT3	PubChem	Swiss Target Prediction
Codonopsis Radix (Dang Shen, DS)	MOL000006	Luteolin	tankyrase	TNKS	PubChem	Swiss Target Prediction
Codonopsis Radix (Dang Shen, DS)	MOL000006	Luteolin	tankyrase 2	TNKS2	PubChem	Swiss Target Prediction
Codonopsis Radix (Dang Shen, DS)	MOL000006	Luteolin	PC4 and SFRS1 interacting protein 1	PSIP1	PubChem	
Codonopsis Radix (Dang Shen, DS)	MOL000006	Luteolin	cytochrome b-245 beta chain	CYBB	PubChem	
Codonopsis Radix (Dang Shen, DS)	MOL000006	Luteolin	aldo-keto reductase family 1 member B	AKR1B1	PubChem	Swiss Target Prediction
Codonopsis Radix (Dang Shen, DS)	MOL000006	Luteolin	arachidonate 5-lipoxygenase	ALOX5	PubChem	Swiss Target Prediction
Codonopsis Radix (Dang Shen, DS)	MOL000006	Luteolin	amyloid beta precursor protein	APP	PubChem	Swiss Target Prediction
Codonopsis Radix (Dang Shen, DS)	MOL000006	Luteolin	nuclear factor kappa B subunit 1	NFKB1	PubChem	
Codonopsis Radix (Dang Shen, DS)	MOL000006	Luteolin	nuclear factor kappa B subunit 2	NFKB2	PubChem	
Codonopsis Radix (Dang Shen, DS)	MOL000006	Luteolin	v-rel reticuloendotheliosis viral oncogene homol	RELA	PubChem	
Codonopsis Radix (Dang Shen, DS)	MOL000006	Luteolin	Casein kinase II subunit alpha	CSNK2A2	PubChem	
Codonopsis Radix (Dang Shen, DS)	MOL000006	Luteolin	Casein Kinase 2 Beta	CSNK2B	PubChem	
Codonopsis Radix (Dang Shen, DS)	MOL000006	Luteolin	Casein Kinase 2 Alpha 1	CSNK2A1	PubChem	
Codonopsis Radix (Dang Shen, DS)	MOL000006	Luteolin	death associated protein kinase 1	DAPK1	PubChem	
Codonopsis Radix (Dang Shen, DS)	MOL000006	Luteolin	transthyretin	TTR	PubChem	Swiss Target Prediction
Codonopsis Radix (Dang Shen, DS)	MOL000006	Luteolin	carbonic anhydrase 2	CA2	PubChem	Swiss Target Prediction
Codonopsis Radix (Dang Shen, DS)	MOL000006	Luteolin	carbonic anhydrase 7	CA7	PubChem	Swiss Target Prediction
Codonopsis Radix (Dang Shen, DS)	MOL000006	Luteolin	carbonic anhydrase 12	CA12	PubChem	Swiss Target Prediction
Codonopsis Radix (Dang Shen, DS)	MOL000006	Luteolin	carbonic anhydrase 4	CA4	PubChem	Swiss Target Prediction
Codonopsis Radix (Dang Shen, DS)	MOL000006	Luteolin	aldo-keto reductase family 1 member B10	AKR1B10	PubChem	Swiss Target Prediction
Codonopsis Radix (Dang Shen, DS)	MOL000006	Luteolin	polybromo 1	PRBM1	PubChem	
Codonopsis Radix (Dang Shen, DS)	MOL000006	Luteolin	dipeptidyl peptidase 4	DPP4	PubChem	
Codonopsis Radix (Dang Shen, DS)	MOL000006	Luteolin	interferon beta 1	IFNB1	PubChem	
Codonopsis Radix (Dang Shen, DS)	MOL000006	Luteolin	transcription factor binding to IGHM enhancer 3	TFE3	PubChem	
Codonopsis Radix (Dang Shen, DS)	MOL000006	Luteolin	Cyclin-dependent kinase 5/CDK5 activator 1	CDK5R1 CDK5	Swiss Target Prediction	
Codonopsis Radix (Dang Shen, DS)	MOL000006	Luteolin	Adenosine A1 receptor (by homology)	ADORA1	Swiss Target Prediction	
Codonopsis Radix (Dang Shen, DS)	MOL000006	Luteolin	DNA topoisomerase I (by homology)	TOP1	Swiss Target Prediction	
Codonopsis Radix (Dang Shen, DS)	MOL003896	-Methoxy-2-methyl isoflavor	cytochrome P450 family 3 subfamily A member	CYP3A4	PubChem	
Codonopsis Radix (Dang Shen, DS)	MOL003896	-Methoxy-2-methyl isoflavor	cytochrome P450 family 1 subfamily A member	CYP1A2	PubChem	
Codonopsis Radix (Dang Shen, DS)	MOL003896	-Methoxy-2-methyl isoflavor	cytochrome P450 family 2 subfamily C member	CYP2C19	PubChem	
Codonopsis Radix (Dang Shen, DS)	MOL003896	-Methoxy-2-methyl isoflavor	cytochrome P450 family 2 subfamily C member	CYP2C9	PubChem	
Codonopsis Radix (Dang Shen, DS)	MOL003896	-Methoxy-2-methyl isoflavor	cytochrome P450 family 2 subfamily D member	CYP2D6	PubChem	
Codonopsis Radix (Dang Shen, DS)	MOL003896	-Methoxy-2-methyl isoflavor	TAR DNA binding protein	TARDBP	PubChem	
Codonopsis Radix (Dang Shen, DS)	MOL002879	Diop	Androgen Receptor	AR	PubChem	
Codonopsis Radix (Dang Shen, DS)	MOL002879	Diop	GLI family zinc finger 3	GLI3	PubChem	
Codonopsis Radix (Dang Shen, DS)	MOL002879	Diop	nuclear receptor subfamily 1, group I, member 2	NR1I2	PubChem	
Codonopsis Radix (Dang Shen, DS)	MOL000449	Stigmasterol	DNA polymerase beta	POLB	PubChem	
Codonopsis Radix (Dang Shen, DS)	MOL008400	Glycitein	Transcription factor AP-1	JUN	STITCH	
Codonopsis Radix (Dang Shen, DS)	MOL008400	Glycitein	Mitogen-activated protein kinase 3	MAPK3	STITCH	
Codonopsis Radix (Dang Shen, DS)	MOL008400	Glycitein	Transcription factor Sp1	SP1	STITCH	
Codonopsis Radix (Dang Shen, DS)	MOL008400	Glycitein	Collagenase 3	MMP13	STITCH	
Codonopsis Radix (Dang Shen, DS)	MOL008400	Glycitein	Mitogen-activated protein kinase 1	MAPK1	STITCH	
Codonopsis Radix (Dang Shen, DS)	MOL008400	Glycitein	Cyclin-dependent kinase 4	CDK4	STITCH	
Codonopsis Radix (Dang Shen, DS)	MOL008400	Glycitein	Estrogen Receptor 1	ESR1	Pubchem	
Codonopsis Radix (Dang Shen, DS)	MOL002140	Perlolyrine	phosphodiesterase 5A	PDE5A	Pubchem	
