# Supplementary material for: Network Pharmacology Approach for Predicting Targets of Zishen Yutai Pills on Premature Ovarian Insufficiency
Source: Evid Based Complement Alternat Med. 2021 Aug 4;2021:8215454. doi: 10.1155/2021/8215454 (PMC8357500; doi:10.1155/2021/8215454)
Supplement: Supplementary Materials — Supplementary Table 1: active compounds of each herb in ZSYTP. Supplementary Table 2: corresponding targets of active compounds: (A) two target genes of SDH; (B) 138 target genes of GQZ; (C) 202 target genes of TSZ; (D) 25 target genes of BJT; (E) 121 target genes of DS; (F) 11 target genes of BZ; (G) 283 target genes of DZ; (H) 13 target genes of SR; (J) 112 target genes of RS; (K) 76 target genes of SJS; (L) 115 target genes of AY; (M) 149 target genes of HSW; (N) 68 target genes of EJ; (P) 46 target genes of LJJ. Supplementary Table 3: POI targets. Supplementary Table 4: compounds and targets attributes of C-T network. Supplementary Table 5: data of compound-target network. Supplementary Table 6: analysed C-T network: (A) active compounds of C-T network and corresponding degrees; (B) targets of C-T network and corresponding degrees. Supplementary Table 7: ZSYTP core PPI network. Supplementary Table 8: gene ontology and KEGG analysis: (A) GO-BP analysis; (B) GO-MF analysis; (C) GO-CC analysis; (D) KEGG pathway enrichment analysis. [file 8215454.f1.zip › 8215454.f1/ST2F BZ Herb Targets.pdf]

Supplementary Table S2F. Corresponding Targets of BZ

Herb Name	Mol ID	Chemical Name	Protein Name	Symbol	Sources
Atractylodes Macrocephala Koidz. (Bai Zhu, BZ)	MOL000358	Beta-sitosterol	DNA polymerase beta	POLB	PubChem
Atractylodes Macrocephala Koidz. (Bai Zhu, BZ)	MOL000358	Beta-sitosterol	glycogen synthase kinase 3 beta	GSK3B	PubChem
Atractylodes Macrocephala Koidz. (Bai Zhu, BZ)	MOL000358	Beta-sitosterol	protein kinase AMP-activated non-catalytic subunit be	PRKAB2	PubChem
Atractylodes Macrocephala Koidz. (Bai Zhu, BZ)	MOL000358	Beta-sitosterol	protein kinase AMP-activated non-catalytic subunit ga	PRKAG3	PubChem
Atractylodes Macrocephala Koidz. (Bai Zhu, BZ)	MOL000358	Beta-sitosterol	protein kinase AMP-activated non-catalytic subunit ga	PRKAG1	PubChem
Atractylodes Macrocephala Koidz. (Bai Zhu, BZ)	MOL000358	Beta-sitosterol	protein kinase AMP-activated catalytic subunit alpha 1	PRKAA1	PubChem
Atractylodes Macrocephala Koidz. (Bai Zhu, BZ)	MOL000358	Beta-sitosterol	protein kinase AMP-activated catalytic subunit alpha 2	PRKAA2	PubChem
Atractylodes Macrocephala Koidz. (Bai Zhu, BZ)	MOL000358	Beta-sitosterol	protein kinase AMP-activated non-catalytic subunit be	PRKAB1	PubChem
Atractylodes Macrocephala Koidz. (Bai Zhu, BZ)	MOL000358	Beta-sitosterol	protein kinase AMP-activated non-catalytic subunit ga	PRKAG2	PubChem
Atractylodes Macrocephala Koidz. (Bai Zhu, BZ)	MOL000358	Beta-sitosterol	dipeptidyl peptidase 4	DPP4	PubChem
Atractylodes Macrocephala Koidz. (Bai Zhu, BZ)	MOL000358	Beta-sitosterol	coagulation factor II, thrombin	F2	PubChem
