# Supplementary material for: Network Pharmacology Approach for Predicting Targets of Zishen Yutai Pills on Premature Ovarian Insufficiency
Source: Evid Based Complement Alternat Med. 2021 Aug 4;2021:8215454. doi: 10.1155/2021/8215454 (PMC8357500; doi:10.1155/2021/8215454)
Supplement: Supplementary Materials — Supplementary Table 1: active compounds of each herb in ZSYTP. Supplementary Table 2: corresponding targets of active compounds: (A) two target genes of SDH; (B) 138 target genes of GQZ; (C) 202 target genes of TSZ; (D) 25 target genes of BJT; (E) 121 target genes of DS; (F) 11 target genes of BZ; (G) 283 target genes of DZ; (H) 13 target genes of SR; (J) 112 target genes of RS; (K) 76 target genes of SJS; (L) 115 target genes of AY; (M) 149 target genes of HSW; (N) 68 target genes of EJ; (P) 46 target genes of LJJ. Supplementary Table 3: POI targets. Supplementary Table 4: compounds and targets attributes of C-T network. Supplementary Table 5: data of compound-target network. Supplementary Table 6: analysed C-T network: (A) active compounds of C-T network and corresponding degrees; (B) targets of C-T network and corresponding degrees. Supplementary Table 7: ZSYTP core PPI network. Supplementary Table 8: gene ontology and KEGG analysis: (A) GO-BP analysis; (B) GO-MF analysis; (C) GO-CC analysis; (D) KEGG pathway enrichment analysis. [file 8215454.f1.zip › 8215454.f1/ST2J RS Herb Targets.pdf]

Supplementary Table S2H. Corresponding Targets of RS

Herb Name	Mol ID	Chemical Name	Protein Name	Symbol		Sources
Panax Ginseng (Ren Shen, RS)	MOL000787	Fumarine	Histamine H1 receptor	HRH1	STITCH	
Panax Ginseng (Ren Shen, RS)	MOL000787	Fumarine	KIAA0101	KIAA0101	STITCH	
Panax Ginseng (Ren Shen, RS)	MOL000787	Fumarine	coagulation factor II (thrombin)	F2	STITCH	
Panax Ginseng (Ren Shen, RS)	MOL000787	Fumarine	cytochrome P450 family 1 subfamily	CYP1A2	Pubchem	
Panax Ginseng (Ren Shen, RS)	MOL000787	Fumarine	cytochrome P450 family 2 subfamily	CYP2D6	Pubchem	
Panax Ginseng (Ren Shen, RS)	MOL000787	Fumarine	cytochrome P450 family 2 subfamily	CYP2C19	Pubchem	
Panax Ginseng (Ren Shen, RS)	MOL000449	Stigmasterol	DNA polymerase beta	POLB	PubChem	
Panax Ginseng (Ren Shen, RS)	MOL002879	Diop	Androgen Receptor	AR	PubChem	
Panax Ginseng (Ren Shen, RS)	MOL002879	Diop	GLI family zinc finger 3	GLI3	PubChem	
Panax Ginseng (Ren Shen, RS)	MOL002879	Diop	nuclear receptor subfamily 1, group I, me	NR1I2	PubChem	
Panax Ginseng (Ren Shen, RS)	MOL000422	Kaempferol	cyclin-dependent kinase 1	CDK1	STITCH	
Panax Ginseng (Ren Shen, RS)	MOL000422	Kaempferol	nuclear receptor subfamily 1, group I, me	NR1I2	STITCH	
Panax Ginseng (Ren Shen, RS)	MOL000422	Kaempferol	UDP glycosyltransferase 3 family, polype	UGT3A1	STITCH	
Panax Ginseng (Ren Shen, RS)	MOL000422	Kaempferol	cytochrome P450, family 1, subfamily B, p	CYP1B1	STITCH	Swiss Target Prediction
Panax Ginseng (Ren Shen, RS)	MOL000422	Kaempferol	ribosomal protein S6 kinase, 90kDa, poly	RPS6KA3	STITCH	
Panax Ginseng (Ren Shen, RS)	MOL000422	Kaempferol	aryl hydrocarbon receptor	AHR	STITCH	Swiss Target Prediction
Panax Ginseng (Ren Shen, RS)	MOL000422	Kaempferol	UDP glucuronosyltransferase 1 family, po	UGT1A3	STITCH	
Panax Ginseng (Ren Shen, RS)	MOL000422	Kaempferol	UDP glucuronosyltransferase 1 family, po	UGT1A8	STITCH	
Panax Ginseng (Ren Shen, RS)	MOL000422	Kaempferol	UDP glucuronosyltransferase 1 family, po	UGT1A7	STITCH	
Panax Ginseng (Ren Shen, RS)	MOL000422	Kaempferol	UDP glucuronosyltransferase 1 family, po	UGT1A9	STITCH	
Panax Ginseng (Ren Shen, RS)	MOL000422	Kaempferol	cytochrome P450 family 1 subfamily A m	CYP1A2	PubChem	
Panax Ginseng (Ren Shen, RS)	MOL000422	Kaempferol	CDC like kinase 1	CLK1	PubChem	
Panax Ginseng (Ren Shen, RS)	MOL000422	Kaempferol	serine/threonine kinase 16	STK16	PubChem	
Panax Ginseng (Ren Shen, RS)	MOL000422	Kaempferol	Pim-2 proto-oncogene, serine/threonine k	PIM2	PubChem	
Panax Ginseng (Ren Shen, RS)	MOL000422	Kaempferol	microtubule associated protein tau	MAPT	PubChem	
Panax Ginseng (Ren Shen, RS)	MOL000422	Kaempferol	cytochrome P450 family 2 subfamily D m	CYP2D6	PubChem	
Panax Ginseng (Ren Shen, RS)	MOL000422	Kaempferol	cytochrome P450 family 3 subfamily A m	CYP3A4	PubChem	
Panax Ginseng (Ren Shen, RS)	MOL000422	Kaempferol	cytochrome P450 family 2 subfamily C m	CYP2C9	PubChem	
Panax Ginseng (Ren Shen, RS)	MOL000422	Kaempferol	15-hydroxyprostaglandin dehydrogenase	HPGD	PubChem	
Panax Ginseng (Ren Shen, RS)	MOL000422	Kaempferol	hypoxia inducible factor 1 subunit alpha	HIF1A	PubChem	
Panax Ginseng (Ren Shen, RS)	MOL000422	Kaempferol	Cytochrome P450 2C19	CYP2C19	PubChem	
Panax Ginseng (Ren Shen, RS)	MOL000422	Kaempferol	glycogen synthase kinase 3 alpha	GSK3A	PubChem	
Panax Ginseng (Ren Shen, RS)	MOL000422	Kaempferol	glycogen synthase kinase 3 Beta	GSK3B	PubChem	
Panax Ginseng (Ren Shen, RS)	MOL000422	Kaempferol	cyclin B2	CCNB2	PubChem	
Panax Ginseng (Ren Shen, RS)	MOL000422	Kaempferol	cyclin B1	CCNB1	PubChem	
Panax Ginseng (Ren Shen, RS)	MOL000422	Kaempferol	cyclin B3	CCNB3	PubChem	
Panax Ginseng (Ren Shen, RS)	MOL000422	Kaempferol	cyclin dependent kinase 6	CDK6	PubChem	
Panax Ginseng (Ren Shen, RS)	MOL000422	Kaempferol	arachidonate 5-lipoxygenase	ALOX5	PubChem	Swiss Target Prediction
Panax Ginseng (Ren Shen, RS)	MOL000422	Kaempferol	glyoxalase I	GLO1	PubChem	
Panax Ginseng (Ren Shen, RS)	MOL000422	Kaempferol	xanthine dehydrogenase	XDH	PubChem	Swiss Target Prediction
Panax Ginseng (Ren Shen, RS)	MOL000422	Kaempferol	hydroxysteroid 17-Beta dehydrogenase 1	HSD17B1	PubChem	
Panax Ginseng (Ren Shen, RS)	MOL000422	Kaempferol	hydroxysteroid 17-Beta dehydrogenase 2	HSD17B2	PubChem	Swiss Target Prediction
Panax Ginseng (Ren Shen, RS)	MOL000422	Kaempferol	ATP binding cassette subfamily C membe	ABCC1	PubChem	
Panax Ginseng (Ren Shen, RS)	MOL000422	Kaempferol	androgen receptor	AR	PubChem	
Panax Ginseng (Ren Shen, RS)	MOL000422	Kaempferol	butyrylcholinesterase	BCHE	PubChem	
Panax Ginseng (Ren Shen, RS)	MOL000422	Kaempferol	platelet activating factor acetylhydrolase	PAFAH1B3	PubChem	
Panax Ginseng (Ren Shen, RS)	MOL000422	Kaempferol	CTD small phosphatase 1	CTDSP1	PubChem	
Panax Ginseng (Ren Shen, RS)	MOL000422	Kaempferol	cytochrome P450 family 1 subfamily A m	CYP1A1	PubChem	
Panax Ginseng (Ren Shen, RS)	MOL000422	Kaempferol	NADPH oxidase 4	NOX4	PubChem	Swiss Target Prediction
Panax Ginseng (Ren Shen, RS)	MOL000422	Kaempferol	ATP binding cassette subfamily G membe	ABCG2	PubChem	
Panax Ginseng (Ren Shen, RS)	MOL000422	Kaempferol	estrogen receptor 1	ESR1	PubChem	
Panax Ginseng (Ren Shen, RS)	MOL000422	Kaempferol	DNA polymerase iota	POLI	PubChem	
Panax Ginseng (Ren Shen, RS)	MOL000422	Kaempferol	DNA polymerase eta	POLH	PubChem	
Panax Ginseng (Ren Shen, RS)	MOL000422	Kaempferol	cholinergic receptor muscarinic 1	CHRM	PubChem	
Panax Ginseng (Ren Shen, RS)	MOL000422	Kaempferol	ATP binding cassette subfamily B membe	ABCB1	PubChem	Swiss Target Prediction
Panax Ginseng (Ren Shen, RS)	MOL000422	Kaempferol	Rac GTPase activating protein 1	RACGAP1	PubChem	
Panax Ginseng (Ren Shen, RS)	MOL000422	Kaempferol	aldo-keto reductase family 1 member B	AKR1B1	PubChem	Swiss Target Prediction
Panax Ginseng (Ren Shen, RS)	MOL000422	Kaempferol	nuclear factor, erythroid 2 like 2	NFE2L2	PubChem	
Panax Ginseng (Ren Shen, RS)	MOL000422	Kaempferol	phosphatidylinositol-5-phosphate 4-kinas	PIP4K2A	PubChem	
Panax Ginseng (Ren Shen, RS)	MOL000422	Kaempferol	ADAM metallopeptidase domain 10	ADAM10	PubChem	
Panax Ginseng (Ren Shen, RS)	MOL000422	Kaempferol	ADAM metallopeptidase domain 17	ADAM17	PubChem	
Panax Ginseng (Ren Shen, RS)	MOL000422	Kaempferol	nuclear receptor subfamily 3 group C mer	NR3C1	PubChem	
Panax Ginseng (Ren Shen, RS)	MOL000422	Kaempferol	fms related tyrosine kinase 3	FLT3	PubChem	Swiss Target Prediction
Panax Ginseng (Ren Shen, RS)	MOL000422	Kaempferol	peroxisome proliferator activated recepto	PPARG	PubChem	
Panax Ginseng (Ren Shen, RS)	MOL000422	Kaempferol	cytochrome P450 family 19 subfamily A r	CYP19A1	PubChem	
Panax Ginseng (Ren Shen, RS)	MOL000422	Kaempferol	estrogen related receptor alpha	ESRRA	PubChem	Swiss Target Prediction
Panax Ginseng (Ren Shen, RS)	MOL000422	Kaempferol	sialic acid acetylesterase	SIAE	PubChem	
Panax Ginseng (Ren Shen, RS)	MOL000422	Kaempferol	Beta-secretase 1	BACE1	PubChem	
Panax Ginseng (Ren Shen, RS)	MOL000422	Kaempferol	retinoic acid receptor alpha	RARA	PubChem	
Panax Ginseng (Ren Shen, RS)	MOL000422	Kaempferol	nuclear receptor subfamily 1 group I mer	NR1I3	PubChem	
Panax Ginseng (Ren Shen, RS)	MOL000422	Kaempferol	nuclear factor kappa B subunit 1	NFKB1	PubChem	
Panax Ginseng (Ren Shen, RS)	MOL000422	Kaempferol	nuclear factor kappa B subunit 2	NFKB2	PubChem	
Panax Ginseng (Ren Shen, RS)	MOL000422	Kaempferol	RELA proto-oncogene, NF-kB subunit	RELA	PubChem	
Panax Ginseng (Ren Shen, RS)	MOL000422	Kaempferol	casein kinase 2 alpha 2	CSNK2A2	PubChem	
Panax Ginseng (Ren Shen, RS)	MOL000422	Kaempferol	casein kinase 2 Beta	CSNK2B	PubChem	
Panax Ginseng (Ren Shen, RS)	MOL000422	Kaempferol	casein kinase 2 alpha 1	CSNK2A1	PubChem	
Panax Ginseng (Ren Shen, RS)	MOL000422	Kaempferol	death associated protein kinase 1	DAPK1	PubChem	
Panax Ginseng (Ren Shen, RS)	MOL000422	Kaempferol	carbonic anhydrase 2	CA2	PubChem	Swiss Target Prediction
Panax Ginseng (Ren Shen, RS)	MOL000422	Kaempferol	carbonic anhydrase 7	CA7	PubChem	Swiss Target Prediction
Panax Ginseng (Ren Shen, RS)	MOL000422	Kaempferol	carbonic anhydrase 12	CA12	PubChem	Swiss Target Prediction
Panax Ginseng (Ren Shen, RS)	MOL000422	Kaempferol	histone deacetylase 9	HDAC9	PubChem	
Panax Ginseng (Ren Shen, RS)	MOL000422	Kaempferol	GLI family zinc finger 3	GLI3	PubChem	
Panax Ginseng (Ren Shen, RS)	MOL000422	Kaempferol	estrogen receptor 2	ESR2	PubChem	
Panax Ginseng (Ren Shen, RS)	MOL000422	Kaempferol	amyloid Beta precursor protein	APP	PubChem	
Panax Ginseng (Ren Shen, RS)	MOL000422	Kaempferol	CDGSH iron sulfur domain 1	CISD1	PubChem	
Panax Ginseng (Ren Shen, RS)	MOL000422	Kaempferol	Tyrosinase	TYR	PubChem	Swiss Target Prediction
Panax Ginseng (Ren Shen, RS)	MOL000422	Kaempferol	Progesterone receptor	PGR	PubChem	
Panax Ginseng (Ren Shen, RS)	MOL000422	Kaempferol	SMAD family member 2	SMAD2	PubChem	
Panax Ginseng (Ren Shen, RS)	MOL000422	Kaempferol	SMAD family member 3	SMAD3	PubChem	
Panax Ginseng (Ren Shen, RS)	MOL000422	Kaempferol	solute carrier family 2 member 1	SLC2A1	PubChem	
Panax Ginseng (Ren Shen, RS)	MOL000422	Kaempferol	dipeptidyl peptidase 4	DPP4	PubChem	
Panax Ginseng (Ren Shen, RS)	MOL000422	Kaempferol	myeloperoxidase	MPO	PubChem	
Panax Ginseng (Ren Shen, RS)	MOL000422	Kaempferol	Adenosine A1 receptor (by homology)	ADORA1	Swiss Target Prediction	
Panax Ginseng (Ren Shen, RS)	MOL000422	Kaempferol	Carbonic anhydrase IV	CA4	Swiss Target Prediction	
Panax Ginseng (Ren Shen, RS)	MOL000358	Beta-sitosterol	DNA polymerase beta	POLB	PubChem	
Panax Ginseng (Ren Shen, RS)	MOL000358	Beta-sitosterol	glycogen synthase kinase 3 beta	GSK3B	PubChem	
Panax Ginseng (Ren Shen, RS)	MOL000358	Beta-sitosterol	protein kinase AMP-activated non-cataly	PRKAB2	PubChem	
Panax Ginseng (Ren Shen, RS)	MOL000358	Beta-sitosterol	protein kinase AMP-activated non-cataly	PRKAG3	PubChem	
Panax Ginseng (Ren Shen, RS)	MOL000358	Beta-sitosterol	protein kinase AMP-activated non-cataly	PRKAG1	PubChem	
Panax Ginseng (Ren Shen, RS)	MOL000358	Beta-sitosterol	protein kinase AMP-activated catalytic su	PRKAA1	PubChem	
Panax Ginseng (Ren Shen, RS)	MOL000358	Beta-sitosterol	protein kinase AMP-activated catalytic su	PRKAA2	PubChem	
Panax Ginseng (Ren Shen, RS)	MOL000358	Beta-sitosterol	protein kinase AMP-activated non-cataly	PRKAB1	PubChem	
Panax Ginseng (Ren Shen, RS)	MOL000358	Beta-sitosterol	protein kinase AMP-activated non-cataly	PRKAG2	PubChem	
Panax Ginseng (Ren Shen, RS)	MOL000358	Beta-sitosterol	dipeptidyl peptidase 4	DPP4	PubChem	
Panax Ginseng (Ren Shen, RS)	MOL000358	Beta-sitosterol	coagulation factor II, thrombin	F2	PubChem	
Panax Ginseng (Ren Shen, RS)	MOL005344	Ginsenoside rh2	BCL2-related protein A1	BCL2A1	STITCH	
Panax Ginseng (Ren Shen, RS)	MOL005344	Ginsenoside rh2	Rh-associated glycoprotein	RHAG	STITCH	
Panax Ginseng (Ren Shen, RS)	MOL005344	Ginsenoside rh2	sirtuin 1	SIRT1	Pubchem	
Panax Ginseng (Ren Shen, RS)	MOL005320	Arachidonate	Fatty acid binding protein adipocyte	FABP4	Swiss Target Prediction	
Panax Ginseng (Ren Shen, RS)	MOL005320	Arachidonate	Peroxisome proliferator-activated re	PPARG	Swiss Target Prediction	
Panax Ginseng (Ren Shen, RS)	MOL005320	Arachidonate	Peroxisome proliferator-activated re	PPARA	Swiss Target Prediction	
Panax Ginseng (Ren Shen, RS)	MOL005320	Arachidonate	Peroxisome proliferator-activated re	PPARD	Swiss Target Prediction	
