# Supplementary material for: Network Pharmacology Approach for Predicting Targets of Zishen Yutai Pills on Premature Ovarian Insufficiency
Source: Evid Based Complement Alternat Med. 2021 Aug 4;2021:8215454. doi: 10.1155/2021/8215454 (PMC8357500; doi:10.1155/2021/8215454)
Supplement: Supplementary Materials — Supplementary Table 1: active compounds of each herb in ZSYTP. Supplementary Table 2: corresponding targets of active compounds: (A) two target genes of SDH; (B) 138 target genes of GQZ; (C) 202 target genes of TSZ; (D) 25 target genes of BJT; (E) 121 target genes of DS; (F) 11 target genes of BZ; (G) 283 target genes of DZ; (H) 13 target genes of SR; (J) 112 target genes of RS; (K) 76 target genes of SJS; (L) 115 target genes of AY; (M) 149 target genes of HSW; (N) 68 target genes of EJ; (P) 46 target genes of LJJ. Supplementary Table 3: POI targets. Supplementary Table 4: compounds and targets attributes of C-T network. Supplementary Table 5: data of compound-target network. Supplementary Table 6: analysed C-T network: (A) active compounds of C-T network and corresponding degrees; (B) targets of C-T network and corresponding degrees. Supplementary Table 7: ZSYTP core PPI network. Supplementary Table 8: gene ontology and KEGG analysis: (A) GO-BP analysis; (B) GO-MF analysis; (C) GO-CC analysis; (D) KEGG pathway enrichment analysis. [file 8215454.f1.zip › 8215454.f1/ST2K SJS Herb Targets.pdf]

**Supplementary Table S2K. Corresponding Targets of SJS**

| Herb Name | Mol ID | Chemical Name | Protein Name | Symbol | Sources | |
|---|---|---|---|---|---|---|
| *Herba Taxili (Sang Ji Sheng, SJS)* | MOL000098 | Quercetin | Induced myeloid leukemia cel | MCL1 | STITCH | |
| *Herba Taxili (Sang Ji Sheng, SJS)* | MOL000098 | Quercetin | Tyrosine-protein kinase HCK | HCK | STITCH | |
| *Herba Taxili (Sang Ji Sheng, SJS)* | MOL000098 | Quercetin | Solute carrier family 2, facilitat | SLC2A2 | STITCH | |
| *Herba Taxili (Sang Ji Sheng, SJS)* | MOL000098 | Quercetin | Cytochrome P450 2C8 | CYP2C8 | STITCH | |
| *Herba Taxili (Sang Ji Sheng, SJS)* | MOL000098 | Quercetin | ATP synthase subunit Beta, mi | ATP5B | STITCH | |
| *Herba Taxili (Sang Ji Sheng, SJS)* | MOL000098 | Quercetin | 3-hydroxyisobutyryl-CoA hydr | HIBCH | STITCH | |
| *Herba Taxili (Sang Ji Sheng, SJS)* | MOL000098 | Quercetin | serine/threonine kinase 17b | STK17B | STITCH | |
| *Herba Taxili (Sang Ji Sheng, SJS)* | MOL000098 | Quercetin | NADPH oxidase 4 | NOX4 | Swiss Target Prediction | |
| *Herba Taxili (Sang Ji Sheng, SJS)* | MOL000098 | Quercetin | Vasopressin V2 receptor | AVPR2 | Swiss Target Prediction | |
| *Herba Taxili (Sang Ji Sheng, SJS)* | MOL000098 | Quercetin | Aldose reductase | AKR1B1 | Swiss Target Prediction | |
| *Herba Taxili (Sang Ji Sheng, SJS)* | MOL000098 | Quercetin | Xanthine dehydrogenase | XDH | Swiss Target Prediction | |
| *Herba Taxili (Sang Ji Sheng, SJS)* | MOL000098 | Quercetin | Monoamine oxidase A | MAOA | Swiss Target Prediction | |
| *Herba Taxili (Sang Ji Sheng, SJS)* | MOL000098 | Quercetin | Insulin-like growth factor I rec | IGF1R | Swiss Target Prediction | |
| *Herba Taxili (Sang Ji Sheng, SJS)* | MOL000098 | Quercetin | Tyrosine-protein kinase recept | FLT3 | Swiss Target Prediction | |
| *Herba Taxili (Sang Ji Sheng, SJS)* | MOL000098 | Quercetin | Cytochrome P450 19A1 | CYP19A1 | Swiss Target Prediction | |
| *Herba Taxili (Sang Ji Sheng, SJS)* | MOL000098 | Quercetin | Epidermal growth factor recep | EGFR | Swiss Target Prediction | |
| *Herba Taxili (Sang Ji Sheng, SJS)* | MOL000098 | Quercetin | Thrombin | F2 | Swiss Target Prediction | |
| *Herba Taxili (Sang Ji Sheng, SJS)* | MOL000098 | Quercetin | Carbonic anhydrase II | CA2 | Swiss Target Prediction | |
| *Herba Taxili (Sang Ji Sheng, SJS)* | MOL000098 | Quercetin | Serine/threonine-protein kinas | PIM1 | Swiss Target Prediction | STITCH |
| *Herba Taxili (Sang Ji Sheng, SJS)* | MOL000098 | Quercetin | Arachidonate 5-lipoxygenase | ALOX5 | Swiss Target Prediction | |
| *Herba Taxili (Sang Ji Sheng, SJS)* | MOL000098 | Quercetin | Serine/threonine-protein kinas | AURKB | Swiss Target Prediction | |
| *Herba Taxili (Sang Ji Sheng, SJS)* | MOL000098 | Quercetin | Dopamine D4 receptor | DRD4 | Swiss Target Prediction | |
| *Herba Taxili (Sang Ji Sheng, SJS)* | MOL000098 | Quercetin | Adenosine A1 receptor (by ho | ADORA1 | Swiss Target Prediction | |
| *Herba Taxili (Sang Ji Sheng, SJS)* | MOL000098 | Quercetin | Carbonic anhydrase VII | CA7 | Swiss Target Prediction | |
| *Herba Taxili (Sang Ji Sheng, SJS)* | MOL000098 | Quercetin | Glyoxalase I | GLO1 | Swiss Target Prediction | |
| *Herba Taxili (Sang Ji Sheng, SJS)* | MOL000098 | Quercetin | Myeloperoxidase | MPO | Swiss Target Prediction | |
| *Herba Taxili (Sang Ji Sheng, SJS)* | MOL000098 | Quercetin | PI3-kinase p85-alpha subunit | PIK3R1 | Swiss Target Prediction | |
| *Herba Taxili (Sang Ji Sheng, SJS)* | MOL000098 | Quercetin | Adenosine A2a receptor (by h | ADORA2A | Swiss Target Prediction | |
| *Herba Taxili (Sang Ji Sheng, SJS)* | MOL000098 | Quercetin | Death-associated protein kina | DAPK1 | Swiss Target Prediction | |
| *Herba Taxili (Sang Ji Sheng, SJS)* | MOL000098 | Quercetin | Liver glycogen phosphorylase | PYGL | Swiss Target Prediction | |
| *Herba Taxili (Sang Ji Sheng, SJS)* | MOL000098 | Quercetin | Carbonic anhydrase I | CA1 | Swiss Target Prediction | |
| *Herba Taxili (Sang Ji Sheng, SJS)* | MOL000098 | Quercetin | Glycogen synthase kinase-3 B | GSK3B | Swiss Target Prediction | |
| *Herba Taxili (Sang Ji Sheng, SJS)* | MOL000098 | Quercetin | Tyrosine-protein kinase SRC | SRC | Swiss Target Prediction | |
| *Herba Taxili (Sang Ji Sheng, SJS)* | MOL000098 | Quercetin | Focal adhesion kinase 1 | PTK2 | Swiss Target Prediction | |
| *Herba Taxili (Sang Ji Sheng, SJS)* | MOL000098 | Quercetin | Estradiol 17-Beta-dehydrogen | HSD17B2 | Swiss Target Prediction | |
| *Herba Taxili (Sang Ji Sheng, SJS)* | MOL000098 | Quercetin | Vascular endothelial growth fa | KDR | Swiss Target Prediction | |
| *Herba Taxili (Sang Ji Sheng, SJS)* | MOL000098 | Quercetin | Matrix metalloproteinase 13 | MMP13 | Swiss Target Prediction | |
| *Herba Taxili (Sang Ji Sheng, SJS)* | MOL000098 | Quercetin | Matrix metalloproteinase 3 | MMP3 | Swiss Target Prediction | |
| *Herba Taxili (Sang Ji Sheng, SJS)* | MOL000098 | Quercetin | Carbonic anhydrase III | CA3 | Swiss Target Prediction | |
| *Herba Taxili (Sang Ji Sheng, SJS)* | MOL000098 | Quercetin | Arachidonate 15-lipoxygenase | ALOX15 | Swiss Target Prediction | |
| *Herba Taxili (Sang Ji Sheng, SJS)* | MOL000098 | Quercetin | Multidrug resistance-associate | ABCC1 | Swiss Target Prediction | |
| *Herba Taxili (Sang Ji Sheng, SJS)* | MOL000098 | Quercetin | Serine/threonine-protein kinas | PLK1 | Swiss Target Prediction | |
| *Herba Taxili (Sang Ji Sheng, SJS)* | MOL000098 | Quercetin | Carbonic anhydrase VI | CA6 | Swiss Target Prediction | |
| *Herba Taxili (Sang Ji Sheng, SJS)* | MOL000098 | Quercetin | Cyclin-dependent kinase 1 | CDK1 | Swiss Target Prediction | |
| *Herba Taxili (Sang Ji Sheng, SJS)* | MOL000098 | Quercetin | Matrix metalloproteinase 9 | MMP9 | Swiss Target Prediction | |
| *Herba Taxili (Sang Ji Sheng, SJS)* | MOL000098 | Quercetin | Carbonic anhydrase XII | CA12 | Swiss Target Prediction | |
| *Herba Taxili (Sang Ji Sheng, SJS)* | MOL000098 | Quercetin | Matrix metalloproteinase 2 | MMP2 | Swiss Target Prediction | |
| *Herba Taxili (Sang Ji Sheng, SJS)* | MOL000098 | Quercetin | Protein kinase N1 | PKN1 | Swiss Target Prediction | |
| *Herba Taxili (Sang Ji Sheng, SJS)* | MOL000098 | Quercetin | Carbonic anhydrase XIV | CA14 | Swiss Target Prediction | |
| *Herba Taxili (Sang Ji Sheng, SJS)* | MOL000098 | Quercetin | Carbonic anhydrase IX | CA9 | Swiss Target Prediction | |
| *Herba Taxili (Sang Ji Sheng, SJS)* | MOL000098 | Quercetin | Casein kinase II alpha | CSNK2A1 | Swiss Target Prediction | |
| *Herba Taxili (Sang Ji Sheng, SJS)* | MOL000098 | Quercetin | Arachidonate 12-lipoxygenase | ALOX12 | Swiss Target Prediction | |
| *Herba Taxili (Sang Ji Sheng, SJS)* | MOL000098 | Quercetin | Hepatocyte growth factor rece | MET | Swiss Target Prediction | |
| *Herba Taxili (Sang Ji Sheng, SJS)* | MOL000098 | Quercetin | Carbonic anhydrase IV | CA4 | Swiss Target Prediction | |
| *Herba Taxili (Sang Ji Sheng, SJS)* | MOL000098 | Quercetin | Serine/threonine-protein kinas | NEK2 | Swiss Target Prediction | |
| *Herba Taxili (Sang Ji Sheng, SJS)* | MOL000098 | Quercetin | Interleukin-8 receptor A | CXCR1 | Swiss Target Prediction | |
| *Herba Taxili (Sang Ji Sheng, SJS)* | MOL000098 | Quercetin | CaM kinase II Beta | CAMK2B | Swiss Target Prediction | |
| *Herba Taxili (Sang Ji Sheng, SJS)* | MOL000098 | Quercetin | ALK tyrosine kinase receptor | ALK | Swiss Target Prediction | |
| *Herba Taxili (Sang Ji Sheng, SJS)* | MOL000098 | Quercetin | Serine/threonine-protein kinas | AKT1 | Swiss Target Prediction | |
| *Herba Taxili (Sang Ji Sheng, SJS)* | MOL000098 | Quercetin | P-glycoprotein 1 | ABCB1 | Swiss Target Prediction | |
| *Herba Taxili (Sang Ji Sheng, SJS)* | MOL000098 | Quercetin | Serine/threonine-protein kinas | NEK6 | Swiss Target Prediction | |
| *Herba Taxili (Sang Ji Sheng, SJS)* | MOL000098 | Quercetin | Phospholipase A2 group 1B | PLA2G1B | Swiss Target Prediction | |
| *Herba Taxili (Sang Ji Sheng, SJS)* | MOL000098 | Quercetin | Carbonic anhydrase VA | CA5A | Swiss Target Prediction | |
| *Herba Taxili (Sang Ji Sheng, SJS)* | MOL000098 | Quercetin | Beta-secretase 1 | BACE1 | Swiss Target Prediction | |
| *Herba Taxili (Sang Ji Sheng, SJS)* | MOL000098 | Quercetin | Cytochrome P450 1B1 | CYP1B1 | Swiss Target Prediction | STITCH |
| *Herba Taxili (Sang Ji Sheng, SJS)* | MOL000098 | Quercetin | Tyrosine-protein kinase recept | AXL | Swiss Target Prediction | |
| *Herba Taxili (Sang Ji Sheng, SJS)* | MOL000098 | Quercetin | ATP-binding cassette sub-fam | ABCG2 | Swiss Target Prediction | |
| *Herba Taxili (Sang Ji Sheng, SJS)* | MOL000098 | Quercetin | NUAK family SNF1-like kinase | NUAK1 | Swiss Target Prediction | |
| *Herba Taxili (Sang Ji Sheng, SJS)* | MOL000098 | Quercetin | Aldo-keto reductase family 1 r | AKR1C2 | Swiss Target Prediction | |
| *Herba Taxili (Sang Ji Sheng, SJS)* | MOL000098 | Quercetin | Aldo-keto reductase family 1 r | AKR1C1 | Swiss Target Prediction | |
| *Herba Taxili (Sang Ji Sheng, SJS)* | MOL000098 | Quercetin | Aldo-keto-reductase family 1 r | AKR1C3 | Swiss Target Prediction | |
| *Herba Taxili (Sang Ji Sheng, SJS)* | MOL000098 | Quercetin | Aldo-keto reductase family 1 r | AKR1C4 | Swiss Target Prediction | |
| *Herba Taxili (Sang Ji Sheng, SJS)* | MOL000098 | Quercetin | Carbonic anhydrase XIII (by h | CA13 | Swiss Target Prediction | |
| *Herba Taxili (Sang Ji Sheng, SJS)* | MOL000098 | Quercetin | Aldehyde reductase (by hom | AKR1A1 | Swiss Target Prediction | |
| *Herba Taxili (Sang Ji Sheng, SJS)* | MOL000098 | Quercetin | G-protein coupled receptor 35 | GPR35 | Swiss Target Prediction | |
| *Herba Taxili (Sang Ji Sheng, SJS)* | MOL000098 | Quercetin | Tyrosine-protein kinase SYK | SYK | Swiss Target Prediction | |
