# Supplementary material for: Network Pharmacology Approach for Predicting Targets of Zishen Yutai Pills on Premature Ovarian Insufficiency
Source: Evid Based Complement Alternat Med. 2021 Aug 4;2021:8215454. doi: 10.1155/2021/8215454 (PMC8357500; doi:10.1155/2021/8215454)
Supplement: Supplementary Materials — Supplementary Table 1: active compounds of each herb in ZSYTP. Supplementary Table 2: corresponding targets of active compounds: (A) two target genes of SDH; (B) 138 target genes of GQZ; (C) 202 target genes of TSZ; (D) 25 target genes of BJT; (E) 121 target genes of DS; (F) 11 target genes of BZ; (G) 283 target genes of DZ; (H) 13 target genes of SR; (J) 112 target genes of RS; (K) 76 target genes of SJS; (L) 115 target genes of AY; (M) 149 target genes of HSW; (N) 68 target genes of EJ; (P) 46 target genes of LJJ. Supplementary Table 3: POI targets. Supplementary Table 4: compounds and targets attributes of C-T network. Supplementary Table 5: data of compound-target network. Supplementary Table 6: analysed C-T network: (A) active compounds of C-T network and corresponding degrees; (B) targets of C-T network and corresponding degrees. Supplementary Table 7: ZSYTP core PPI network. Supplementary Table 8: gene ontology and KEGG analysis: (A) GO-BP analysis; (B) GO-MF analysis; (C) GO-CC analysis; (D) KEGG pathway enrichment analysis. [file 8215454.f1.zip › 8215454.f1/ST2L AY Herb Targets.pdf]

Supplementary Table S2L. Corresponding Targets of AY

Herb Name	Mol ID	Chemical Name	Protein Name	Symbol	Sources	
Folium Artemisiae Argyi (Ai Ye, AY)	MOL000358	Beta-sitosterol	DNA polymerase beta	POLB	PubChem	
Folium Artemisiae Argyi (Ai Ye, AY)	MOL000358	Beta-sitosterol	glycogen synthase kinase 3 beta	GSK3B	PubChem	
Folium Artemisiae Argyi (Ai Ye, AY)	MOL000358	Beta-sitosterol	protein kinase AMP-activated non-catalytic s	PRKAB2	PubChem	
Folium Artemisiae Argyi (Ai Ye, AY)	MOL000358	Beta-sitosterol	protein kinase AMP-activated non-catalytic s	PRKAG3	PubChem	
Folium Artemisiae Argyi (Ai Ye, AY)	MOL000358	Beta-sitosterol	protein kinase AMP-activated non-catalytic s	PRKAG1	PubChem	
Folium Artemisiae Argyi (Ai Ye, AY)	MOL000358	Beta-sitosterol	protein kinase AMP-activated catalytic subur	PRKAA1	PubChem	
Folium Artemisiae Argyi (Ai Ye, AY)	MOL000358	Beta-sitosterol	protein kinase AMP-activated catalytic subur	PRKAA2	PubChem	
Folium Artemisiae Argyi (Ai Ye, AY)	MOL000358	Beta-sitosterol	protein kinase AMP-activated non-catalytic s	PRKAB1	PubChem	
Folium Artemisiae Argyi (Ai Ye, AY)	MOL000358	Beta-sitosterol	protein kinase AMP-activated non-catalytic s	PRKAG2	PubChem	
Folium Artemisiae Argyi (Ai Ye, AY)	MOL000358	Beta-sitosterol	dipeptidyl peptidase 4	DPP4	PubChem	
Folium Artemisiae Argyi (Ai Ye, AY)	MOL000358	Beta-sitosterol	coagulation factor II, thrombin	F2	PubChem	
Folium Artemisiae Argyi (Ai Ye, AY)	MOL001494	Mandenol	peroxisome proliferator activated recep	PPARG	PubChem	
Folium Artemisiae Argyi (Ai Ye, AY)	MOL001494	Mandenol	heat shock protein family B (small) mem	HSPB1	PubChem	
Folium Artemisiae Argyi (Ai Ye, AY)	MOL001494	Mandenol	retinoic acid receptor alpha	RARA	PubChem	
Folium Artemisiae Argyi (Ai Ye, AY)	MOL001494	Mandenol	G protein-coupled receptor 32	GPR32	PubChem	
Folium Artemisiae Argyi (Ai Ye, AY)	MOL001494	Mandenol	nuclear receptor subfamily 1, group I, m	NR1I2	PubChem	
Folium Artemisiae Argyi (Ai Ye, AY)	MOL001494	Mandenol	nuclear factor, erythroid 2 like 2	NFE2L2	PubChem	
Folium Artemisiae Argyi (Ai Ye, AY)	MOL001494	Mandenol	peroxisome proliferator activated recep	PPARD	PubChem	
Folium Artemisiae Argyi (Ai Ye, AY)	MOL001494	Mandenol	vitamin D receptor	VDR	PubChem	
Folium Artemisiae Argyi (Ai Ye, AY)	MOL001040	xy-2-(4-hydroxyphen	peroxisome proliferator-activated recep	PPARA	STITCH	
Folium Artemisiae Argyi (Ai Ye, AY)	MOL001040	xy-2-(4-hydroxyphen	ATP-binding cassette, sub-family B (MDI	ABCB1	STITCH	
Folium Artemisiae Argyi (Ai Ye, AY)	MOL001040	xy-2-(4-hydroxyphen	cytochrome P450, family 1, subfamily A	CYP1A2	STITCH	
Folium Artemisiae Argyi (Ai Ye, AY)	MOL001040	xy-2-(4-hydroxyphen	apolipoprotein B (including Ag(x) antige	APOB	STITCH	
Folium Artemisiae Argyi (Ai Ye, AY)	MOL001040	xy-2-(4-hydroxyphen	chemokine (C-C motif) ligand 2	CCL2	STITCH	
Folium Artemisiae Argyi (Ai Ye, AY)	MOL001040	xy-2-(4-hydroxyphen	heme oxygenase (decycling) 1	HMOX1	STITCH	
Folium Artemisiae Argyi (Ai Ye, AY)	MOL001040	xy-2-(4-hydroxyphen	low density lipoprotein receptor (860 a	LDLR	STITCH	
Folium Artemisiae Argyi (Ai Ye, AY)	MOL001040	xy-2-(4-hydroxyphen	brain-derived neurotrophic factor (329	BDNF	STITCH	
Folium Artemisiae Argyi (Ai Ye, AY)	MOL001040	xy-2-(4-hydroxyphen	Rap guanine nucleotide exchange factor	RAPGEF1	STITCH	
Folium Artemisiae Argyi (Ai Ye, AY)	MOL001040	xy-2-(4-hydroxyphen	stearoyl-CoA desaturase (delta-9-desatu	SCD	STITCH	
Folium Artemisiae Argyi (Ai Ye, AY)	MOL001040	xy-2-(4-hydroxyphen	Cytochrome P450 19A1	CYP19A1	Swiss Target Prediction	
Folium Artemisiae Argyi (Ai Ye, AY)	MOL001040	xy-2-(4-hydroxyphen	Carbonic anhydrase VII	CA7	Swiss Target Prediction	
Folium Artemisiae Argyi (Ai Ye, AY)	MOL001040	xy-2-(4-hydroxyphen	Multidrug resistance-associated protein	ABCC1	Swiss Target Prediction	
Folium Artemisiae Argyi (Ai Ye, AY)	MOL001040	xy-2-(4-hydroxyphen	Estradiol 17-beta-dehydrogenase 1	HSD17B1	Swiss Target Prediction	
Folium Artemisiae Argyi (Ai Ye, AY)	MOL001040	xy-2-(4-hydroxyphen	Carbonic anhydrase XII	CA12	Swiss Target Prediction	
Folium Artemisiae Argyi (Ai Ye, AY)	MOL001040	xy-2-(4-hydroxyphen	Testis-specific androgen-binding protein	SHBG	Swiss Target Prediction	
Folium Artemisiae Argyi (Ai Ye, AY)	MOL001040	xy-2-(4-hydroxyphen	Carbonic anhydrase IV	CA4	Swiss Target Prediction	
Folium Artemisiae Argyi (Ai Ye, AY)	MOL001040	xy-2-(4-hydroxyphen	Cytochrome P450 1B1	CYP1B1	Swiss Target Prediction	
Folium Artemisiae Argyi (Ai Ye, AY)	MOL001040	xy-2-(4-hydroxyphen	Carbonyl reductase [NADPH] 1	CBR1	Swiss Target Prediction	
Folium Artemisiae Argyi (Ai Ye, AY)	MOL000449	Stigmasterol	DNA polymerase beta	POLB	PubChem	
Folium Artemisiae Argyi (Ai Ye, AY)	MOL000098	Quercetin	Induced myeloid leukemia cell differentiation	MCL1	STITCH	
Folium Artemisiae Argyi (Ai Ye, AY)	MOL000098	Quercetin	Tyrosine-protein kinase HCK	HCK	STITCH	
Folium Artemisiae Argyi (Ai Ye, AY)	MOL000098	Quercetin	Solute carrier family 2, facilitated glucose trar	SLC2A2	STITCH	
Folium Artemisiae Argyi (Ai Ye, AY)	MOL000098	Quercetin	Cytochrome P450 2C8	CYP2C8	STITCH	
Folium Artemisiae Argyi (Ai Ye, AY)	MOL000098	Quercetin	ATP synthase subunit Beta, mitochondrial	ATP5B	STITCH	
Folium Artemisiae Argyi (Ai Ye, AY)	MOL000098	Quercetin	3-hydroxyisobutyryl-CoA hydrolase, mitochor	HIBCH	STITCH	
Folium Artemisiae Argyi (Ai Ye, AY)	MOL000098	Quercetin	serine/threonine kinase 17b	STK17B	STITCH	
Folium Artemisiae Argyi (Ai Ye, AY)	MOL000098	Quercetin	NADPH oxidase 4	NOX4	Swiss Target Prediction	
Folium Artemisiae Argyi (Ai Ye, AY)	MOL000098	Quercetin	Vasopressin V2 receptor	AVPR2	Swiss Target Prediction	
Folium Artemisiae Argyi (Ai Ye, AY)	MOL000098	Quercetin	Aldose reductase	AKR1B1	Swiss Target Prediction	
Folium Artemisiae Argyi (Ai Ye, AY)	MOL000098	Quercetin	Xanthine dehydrogenase	XDH	Swiss Target Prediction	
Folium Artemisiae Argyi (Ai Ye, AY)	MOL000098	Quercetin	Monoamine oxidase A	MAOA	Swiss Target Prediction	
Folium Artemisiae Argyi (Ai Ye, AY)	MOL000098	Quercetin	Insulin-like growth factor I receptor	IGF1R	Swiss Target Prediction	
Folium Artemisiae Argyi (Ai Ye, AY)	MOL000098	Quercetin	Tyrosine-protein kinase receptor FLT3	FLT3	Swiss Target Prediction	
Folium Artemisiae Argyi (Ai Ye, AY)	MOL000098	Quercetin	Cytochrome P450 19A1	CYP19A1	Swiss Target Prediction	
Folium Artemisiae Argyi (Ai Ye, AY)	MOL000098	Quercetin	Epidermal growth factor receptor erbB1	EGFR	Swiss Target Prediction	
Folium Artemisiae Argyi (Ai Ye, AY)	MOL000098	Quercetin	Thrombin	F2	Swiss Target Prediction	
Folium Artemisiae Argyi (Ai Ye, AY)	MOL000098	Quercetin	Carbonic anhydrase II	CA2	Swiss Target Prediction	
Folium Artemisiae Argyi (Ai Ye, AY)	MOL000098	Quercetin	Serine/threonine-protein kinase PIM1	PIM1	Swiss Target Prediction	STITCH
Folium Artemisiae Argyi (Ai Ye, AY)	MOL000098	Quercetin	Arachidonate 5-lipoxygenase	ALOX5	Swiss Target Prediction	
Folium Artemisiae Argyi (Ai Ye, AY)	MOL000098	Quercetin	Serine/threonine-protein kinase Aurora-B	AURKB	Swiss Target Prediction	
Folium Artemisiae Argyi (Ai Ye, AY)	MOL000098	Quercetin	Dopamine D4 receptor	DRD4	Swiss Target Prediction	
Folium Artemisiae Argyi (Ai Ye, AY)	MOL000098	Quercetin	Adenosine A1 receptor (by homology)	ADORA1	Swiss Target Prediction	
Folium Artemisiae Argyi (Ai Ye, AY)	MOL000098	Quercetin	Carbonic anhydrase VII	CA7	Swiss Target Prediction	
Folium Artemisiae Argyi (Ai Ye, AY)	MOL000098	Quercetin	Glyoxalase I	GLO1	Swiss Target Prediction	
Folium Artemisiae Argyi (Ai Ye, AY)	MOL000098	Quercetin	Myeloperoxidase	MPO	Swiss Target Prediction	
Folium Artemisiae Argyi (Ai Ye, AY)	MOL000098	Quercetin	PI3-kinase p85-alpha subunit	PIK3R1	Swiss Target Prediction	
Folium Artemisiae Argyi (Ai Ye, AY)	MOL000098	Quercetin	Adenosine A2a receptor (by homology)	ADORA2A	Swiss Target Prediction	
Folium Artemisiae Argyi (Ai Ye, AY)	MOL000098	Quercetin	Death-associated protein kinase 1	DAPK1	Swiss Target Prediction	
Folium Artemisiae Argyi (Ai Ye, AY)	MOL000098	Quercetin	Liver glycogen phosphorylase	PYGL	Swiss Target Prediction	
Folium Artemisiae Argyi (Ai Ye, AY)	MOL000098	Quercetin	Carbonic anhydrase I	CA1	Swiss Target Prediction	
Folium Artemisiae Argyi (Ai Ye, AY)	MOL000098	Quercetin	Glycogen synthase kinase-3 Beta	GSK3B	Swiss Target Prediction	
Folium Artemisiae Argyi (Ai Ye, AY)	MOL000098	Quercetin	Tyrosine-protein kinase SRC	SRC	Swiss Target Prediction	
Folium Artemisiae Argyi (Ai Ye, AY)	MOL000098	Quercetin	Focal adhesion kinase 1	PTK2	Swiss Target Prediction	
Folium Artemisiae Argyi (Ai Ye, AY)	MOL000098	Quercetin	Estradiol 17-Beta-dehydrogenase 2	HSD17B2	Swiss Target Prediction	
Folium Artemisiae Argyi (Ai Ye, AY)	MOL000098	Quercetin	Vascular endothelial growth factor receptor 2	KDR	Swiss Target Prediction	
Folium Artemisiae Argyi (Ai Ye, AY)	MOL000098	Quercetin	Matrix metalloproteinase 13	MMP13	Swiss Target Prediction	
Folium Artemisiae Argyi (Ai Ye, AY)	MOL000098	Quercetin	Matrix metalloproteinase 3	MMP3	Swiss Target Prediction	
Folium Artemisiae Argyi (Ai Ye, AY)	MOL000098	Quercetin	Carbonic anhydrase III	CA3	Swiss Target Prediction	
Folium Artemisiae Argyi (Ai Ye, AY)	MOL000098	Quercetin	Arachidonate 15-lipoxygenase	ALOX15	Swiss Target Prediction	
Folium Artemisiae Argyi (Ai Ye, AY)	MOL000098	Quercetin	Multidrug resistance-associated protein 1	ABCC1	Swiss Target Prediction	
Folium Artemisiae Argyi (Ai Ye, AY)	MOL000098	Quercetin	Serine/threonine-protein kinase PLK1	PLK1	Swiss Target Prediction	
Folium Artemisiae Argyi (Ai Ye, AY)	MOL000098	Quercetin	Carbonic anhydrase VI	CA6	Swiss Target Prediction	
Folium Artemisiae Argyi (Ai Ye, AY)	MOL000098	Quercetin	Cyclin-dependent kinase 1	CDK1	Swiss Target Prediction	
Folium Artemisiae Argyi (Ai Ye, AY)	MOL000098	Quercetin	Matrix metalloproteinase 9	MMP9	Swiss Target Prediction	
Folium Artemisiae Argyi (Ai Ye, AY)	MOL000098	Quercetin	Carbonic anhydrase XII	CA12	Swiss Target Prediction	
Folium Artemisiae Argyi (Ai Ye, AY)	MOL000098	Quercetin	Matrix metalloproteinase 2	MMP2	Swiss Target Prediction	
Folium Artemisiae Argyi (Ai Ye, AY)	MOL000098	Quercetin	Protein kinase N1	PKN1	Swiss Target Prediction	
Folium Artemisiae Argyi (Ai Ye, AY)	MOL000098	Quercetin	Carbonic anhydrase XIV	CA14	Swiss Target Prediction	
Folium Artemisiae Argyi (Ai Ye, AY)	MOL000098	Quercetin	Carbonic anhydrase IX	CA9	Swiss Target Prediction	
Folium Artemisiae Argyi (Ai Ye, AY)	MOL000098	Quercetin	Casein kinase II alpha	CSNK2A1	Swiss Target Prediction	
Folium Artemisiae Argyi (Ai Ye, AY)	MOL000098	Quercetin	Arachidonate 12-lipoxygenase	ALOX12	Swiss Target Prediction	
Folium Artemisiae Argyi (Ai Ye, AY)	MOL000098	Quercetin	Hepatocyte growth factor receptor	MET	Swiss Target Prediction	
Folium Artemisiae Argyi (Ai Ye, AY)	MOL000098	Quercetin	Carbonic anhydrase IV	CA4	Swiss Target Prediction	
Folium Artemisiae Argyi (Ai Ye, AY)	MOL000098	Quercetin	Serine/threonine-protein kinase NEK2	NEK2	Swiss Target Prediction	
Folium Artemisiae Argyi (Ai Ye, AY)	MOL000098	Quercetin	Interleukin-8 receptor A	CXCR1	Swiss Target Prediction	
Folium Artemisiae Argyi (Ai Ye, AY)	MOL000098	Quercetin	CaM kinase II Beta	CAMK2B	Swiss Target Prediction	
Folium Artemisiae Argyi (Ai Ye, AY)	MOL000098	Quercetin	ALK tyrosine kinase receptor	ALK	Swiss Target Prediction	
Folium Artemisiae Argyi (Ai Ye, AY)	MOL000098	Quercetin	Serine/threonine-protein kinase AKT	AKT1	Swiss Target Prediction	
Folium Artemisiae Argyi (Ai Ye, AY)	MOL000098	Quercetin	P-glycoprotein 1	ABCB1	Swiss Target Prediction	
Folium Artemisiae Argyi (Ai Ye, AY)	MOL000098	Quercetin	Serine/threonine-protein kinase NEK6	NEK6	Swiss Target Prediction	
Folium Artemisiae Argyi (Ai Ye, AY)	MOL000098	Quercetin	Phospholipase A2 group 1B	PLA2G1B	Swiss Target Prediction	
Folium Artemisiae Argyi (Ai Ye, AY)	MOL000098	Quercetin	Carbonic anhydrase VA	CA5A	Swiss Target Prediction	
Folium Artemisiae Argyi (Ai Ye, AY)	MOL000098	Quercetin	Beta-secretase 1	BACE1	Swiss Target Prediction	
Folium Artemisiae Argyi (Ai Ye, AY)	MOL000098	Quercetin	Cytochrome P450 1B1	CYP1B1	Swiss Target Prediction	STITCH
Folium Artemisiae Argyi (Ai Ye, AY)	MOL000098	Quercetin	Tyrosine-protein kinase receptor UFO	AXL	Swiss Target Prediction	
Folium Artemisiae Argyi (Ai Ye, AY)	MOL000098	Quercetin	ATP-binding cassette sub-family G member 2	ABCG2	Swiss Target Prediction	
Folium Artemisiae Argyi (Ai Ye, AY)	MOL000098	Quercetin	NUAK family SNF1-like kinase 1	NUAK1	Swiss Target Prediction	
Folium Artemisiae Argyi (Ai Ye, AY)	MOL000098	Quercetin	Aldo-keto reductase family 1 member C2 (b	AKR1C2	Swiss Target Prediction	
Folium Artemisiae Argyi (Ai Ye, AY)	MOL000098	Quercetin	Aldo-keto reductase family 1 member C1 (b	AKR1C1	Swiss Target Prediction	
Folium Artemisiae Argyi (Ai Ye, AY)	MOL000098	Quercetin	Aldo-keto-reductase family 1 member C3 (b	AKR1C3	Swiss Target Prediction	
Folium Artemisiae Argyi (Ai Ye, AY)	MOL000098	Quercetin	Aldo-keto reductase family 1 member C4 (b	AKR1C4	Swiss Target Prediction	
Folium Artemisiae Argyi (Ai Ye, AY)	MOL000098	Quercetin	Carbonic anhydrase XIII (by homology)	CA13	Swiss Target Prediction	
Folium Artemisiae Argyi (Ai Ye, AY)	MOL000098	Quercetin	Aldehyde reductase (by homology)	AKR1A1	Swiss Target Prediction	
Folium Artemisiae Argyi (Ai Ye, AY)	MOL000098	Quercetin	G-protein coupled receptor 35	GPR35	Swiss Target Prediction	
Folium Artemisiae Argyi (Ai Ye, AY)	MOL000098	Quercetin	Tyrosine-protein kinase SYK	SYK	Swiss Target Prediction	
