# Supplementary material for: Network Pharmacology Approach for Predicting Targets of Zishen Yutai Pills on Premature Ovarian Insufficiency
Source: Evid Based Complement Alternat Med. 2021 Aug 4;2021:8215454. doi: 10.1155/2021/8215454 (PMC8357500; doi:10.1155/2021/8215454)
Supplement: Supplementary Materials — Supplementary Table 1: active compounds of each herb in ZSYTP. Supplementary Table 2: corresponding targets of active compounds: (A) two target genes of SDH; (B) 138 target genes of GQZ; (C) 202 target genes of TSZ; (D) 25 target genes of BJT; (E) 121 target genes of DS; (F) 11 target genes of BZ; (G) 283 target genes of DZ; (H) 13 target genes of SR; (J) 112 target genes of RS; (K) 76 target genes of SJS; (L) 115 target genes of AY; (M) 149 target genes of HSW; (N) 68 target genes of EJ; (P) 46 target genes of LJJ. Supplementary Table 3: POI targets. Supplementary Table 4: compounds and targets attributes of C-T network. Supplementary Table 5: data of compound-target network. Supplementary Table 6: analysed C-T network: (A) active compounds of C-T network and corresponding degrees; (B) targets of C-T network and corresponding degrees. Supplementary Table 7: ZSYTP core PPI network. Supplementary Table 8: gene ontology and KEGG analysis: (A) GO-BP analysis; (B) GO-MF analysis; (C) GO-CC analysis; (D) KEGG pathway enrichment analysis. [file 8215454.f1.zip › 8215454.f1/ST2M HSW Herb Targets.pdf]

**Supplementary Table S2M. Corresponding Targets of HSW**

| Herb Name | Mol ID | Chemical Name | Protein Name | Symbol | | Sources |
|---|---|---|---|---|---|---|
| *Polygonum Multiflorum (He Shou Wu, HSW)* | MOL000358 | Beta-sitosterol | DNA polymerase beta | POLB | PubChem | |
| *Polygonum Multiflorum (He Shou Wu, HSW)* | MOL000358 | Beta-sitosterol | glycogen synthase kinase 3 beta | GSK3B | PubChem | |
| *Polygonum Multiflorum (He Shou Wu, HSW)* | MOL000358 | Beta-sitosterol | protein kinase AMP-activated non-catal | PRKAB2 | PubChem | |
| *Polygonum Multiflorum (He Shou Wu, HSW)* | MOL000358 | Beta-sitosterol | protein kinase AMP-activated non-catal | PRKAG3 | PubChem | |
| *Polygonum Multiflorum (He Shou Wu, HSW)* | MOL000358 | Beta-sitosterol | protein kinase AMP-activated non-catal | PRKAG1 | PubChem | |
| *Polygonum Multiflorum (He Shou Wu, HSW)* | MOL000358 | Beta-sitosterol | protein kinase AMP-activated catalytic s | PRKAA1 | PubChem | |
| *Polygonum Multiflorum (He Shou Wu, HSW)* | MOL000358 | Beta-sitosterol | protein kinase AMP-activated catalytic s | PRKAA2 | PubChem | |
| *Polygonum Multiflorum (He Shou Wu, HSW)* | MOL000358 | Beta-sitosterol | protein kinase AMP-activated non-catal | PRKAB1 | PubChem | |
| *Polygonum Multiflorum (He Shou Wu, HSW)* | MOL000358 | Beta-sitosterol | protein kinase AMP-activated non-catal | PRKAG2 | PubChem | |
| *Polygonum Multiflorum (He Shou Wu, HSW)* | MOL000358 | Beta-sitosterol | dipeptidyl peptidase 4 | DPP4 | PubChem | |
| *Polygonum Multiflorum (He Shou Wu, HSW)* | MOL000358 | Beta-sitosterol | coagulation factor II, thrombin | F2 | PubChem | |
| *Polygonum Multiflorum (He Shou Wu, HSW)* | MOL001729 | Crysophanol | NLR family, pyrin domain containing 3 (1 | NLRP3 | STITCH | |
| *Polygonum Multiflorum (He Shou Wu, HSW)* | MOL001729 | Crysophanol | UDP glucuronosyltransferase 1 family, p | UGT1A9 | STITCH | |
| *Polygonum Multiflorum (He Shou Wu, HSW)* | MOL001729 | Crysophanol | aldehyde dehydrogenase 1 family mem | ALDH1A1 | Pubchem | |
| *Polygonum Multiflorum (He Shou Wu, HSW)* | MOL001729 | Crysophanol | elastase, neutrophil expressed | ELANE | Pubchem | |
| *Polygonum Multiflorum (He Shou Wu, HSW)* | MOL001729 | Crysophanol | vitamin D receptor | VDR | Pubchem | |
| *Polygonum Multiflorum (He Shou Wu, HSW)* | MOL001729 | Crysophanol | nuclear factor, erythroid 2 like 2 | NFE2L2 | Pubchem | |
| *Polygonum Multiflorum (He Shou Wu, HSW)* | MOL001729 | Crysophanol | aryl hydrocarbon receptor | AHR | Pubchem | |
| *Polygonum Multiflorum (He Shou Wu, HSW)* | MOL001729 | Crysophanol | nuclear receptor subfamily 1 group I me | NR1I3 | Pubchem | |
| *Polygonum Multiflorum (He Shou Wu, HSW)* | MOL001729 | Crysophanol | estrogen related receptor alpha | ESRRA | Pubchem | |
| *Polygonum Multiflorum (He Shou Wu, HSW)* | MOL001729 | Crysophanol | androgen receptor | AR | Pubchem | |
| *Polygonum Multiflorum (He Shou Wu, HSW)* | MOL001729 | Crysophanol | histone deacetylase 9 | HDAC9 | Pubchem | |
| *Polygonum Multiflorum (He Shou Wu, HSW)* | MOL001729 | Crysophanol | progesterone receptor | PGR | Pubchem | |
| *Polygonum Multiflorum (He Shou Wu, HSW)* | MOL000472 | Emodin | casein kinase 2, alpha 1 polypeptide | CSNK2A1 | STITCH | |
| *Polygonum Multiflorum (He Shou Wu, HSW)* | MOL000472 | Emodin | caspase 3, apoptosis-related cysteine pe | CASP3 | STITCH | |
| *Polygonum Multiflorum (He Shou Wu, HSW)* | MOL000472 | Emodin | vascular endothelial growth factor A (41 | VEGFA | STITCH | |
| *Polygonum Multiflorum (He Shou Wu, HSW)* | MOL000472 | Emodin | cytochrome P450, family 1, subfamily B, | CYP1B1 | STITCH | |
| *Polygonum Multiflorum (He Shou Wu, HSW)* | MOL000472 | Emodin | v-erb-b2 erythroblastic leukemia viral or | ERBB2 | STITCH | |
| *Polygonum Multiflorum (He Shou Wu, HSW)* | MOL000472 | Emodin | tumor necrosis factor (233 aa) | TP53 | STITCH | |
| *Polygonum Multiflorum (He Shou Wu, HSW)* | MOL000472 | Emodin | lymphocyte-specific protein tyrosine kin | LCK | STITCH | |
| *Polygonum Multiflorum (He Shou Wu, HSW)* | MOL000472 | Emodin | myeloid cell leukemia sequence 1 (BCL2- | MCL1 | STITCH | |
| *Polygonum Multiflorum (He Shou Wu, HSW)* | MOL000472 | Emodin | chemokine (C-X-C motif) receptor 4 | CXCR4 | STITCH | |
| *Polygonum Multiflorum (He Shou Wu, HSW)* | MOL000472 | Emodin | Pim-1 proto-oncogene, serine/threonine | PIM1 | Pubchem | Swiss Target Prediction |
| *Polygonum Multiflorum (He Shou Wu, HSW)* | MOL000472 | Emodin | cytochrome P450 family 1 subfamily A n | CYP1A2 | Pubchem | |
| *Polygonum Multiflorum (He Shou Wu, HSW)* | MOL000472 | Emodin | CDC like kinase 1 | CLK1 | Pubchem | |
| *Polygonum Multiflorum (He Shou Wu, HSW)* | MOL000472 | Emodin | Pim-2 proto-oncogene, serine/threonine | PIM2 | Pubchem | |
| *Polygonum Multiflorum (He Shou Wu, HSW)* | MOL000472 | Emodin | microtubule associated protein tau | MAPT | Pubchem | |
| *Polygonum Multiflorum (He Shou Wu, HSW)* | MOL000472 | Emodin | cytochrome P450 family 2 subfamily D n | CYP2D6 | Pubchem | |
| *Polygonum Multiflorum (He Shou Wu, HSW)* | MOL000472 | Emodin | cytochrome P450 family 3 subfamily A n | CYP3A4 | Pubchem | |
| *Polygonum Multiflorum (He Shou Wu, HSW)* | MOL000472 | Emodin | cytochrome P450 family 2 subfamily C m | CYP2C9 | Pubchem | |
| *Polygonum Multiflorum (He Shou Wu, HSW)* | MOL000472 | Emodin | cytochrome P450 family 2 subfamily C m | CYP2C19 | Pubchem | |
| *Polygonum Multiflorum (He Shou Wu, HSW)* | MOL000472 | Emodin | aldehyde dehydrogenase 1 family mem | ALDH1A1 | Pubchem | |
| *Polygonum Multiflorum (He Shou Wu, HSW)* | MOL000472 | Emodin | thrombopoietin | THPO | Pubchem | |
| *Polygonum Multiflorum (He Shou Wu, HSW)* | MOL000472 | Emodin | SUMO peptidase family member, NEDD | SENP8 | Pubchem | |
| *Polygonum Multiflorum (He Shou Wu, HSW)* | MOL000472 | Emodin | potassium voltage-gated channel subfa | KCNQ1 | Pubchem | |
| *Polygonum Multiflorum (He Shou Wu, HSW)* | MOL000472 | Emodin | SUMO specific peptidase 6 | SENP6 | Pubchem | |
| *Polygonum Multiflorum (He Shou Wu, HSW)* | MOL000472 | Emodin | estrogen receptor 1 | ESR1 | Pubchem | Swiss Target Prediction |
| *Polygonum Multiflorum (He Shou Wu, HSW)* | MOL000472 | Emodin | estrogen receptor 2 | ESR2 | Pubchem | Swiss Target Prediction |
| *Polygonum Multiflorum (He Shou Wu, HSW)* | MOL000472 | Emodin | SUMO specific peptidase 7 | SENP7 | Pubchem | |
| *Polygonum Multiflorum (He Shou Wu, HSW)* | MOL000472 | Emodin | phospholipase A2 group VII | PLA2G7 | Pubchem | |
| *Polygonum Multiflorum (He Shou Wu, HSW)* | MOL000472 | Emodin | DNA polymerase beta | POLB | Pubchem | |
| *Polygonum Multiflorum (He Shou Wu, HSW)* | MOL000472 | Emodin | flap structure-specific endonuclease 1 | FEN1 | Pubchem | |
| *Polygonum Multiflorum (He Shou Wu, HSW)* | MOL000472 | Emodin | chromobox 1 | CBX1 | Pubchem | |
| *Polygonum Multiflorum (He Shou Wu, HSW)* | MOL000472 | Emodin | M-phase phosphoprotein 8 | MPHOSPH8 | Pubchem | |
| *Polygonum Multiflorum (He Shou Wu, HSW)* | MOL000472 | Emodin | protein tyrosine phosphatase 4A3 | PTP4A3 | Pubchem | Swiss Target Prediction |
| *Polygonum Multiflorum (He Shou Wu, HSW)* | MOL000472 | Emodin | casein kinase 2 beta | CSNK2B | Pubchem | Swiss Target Prediction |
| *Polygonum Multiflorum (He Shou Wu, HSW)* | MOL000472 | Emodin | nuclear factor, erythroid 2 like 2 | NFE2L2 | Pubchem | |
| *Polygonum Multiflorum (He Shou Wu, HSW)* | MOL000472 | Emodin | C-X-C motif chemokine ligand 8 | CXCL8 | Pubchem | |
| *Polygonum Multiflorum (He Shou Wu, HSW)* | MOL000472 | Emodin | aryl hydrocarbon receptor | AHR | Pubchem | |
| *Polygonum Multiflorum (He Shou Wu, HSW)* | MOL000472 | Emodin | RAR related orphan receptor C | RORC | Pubchem | |
| *Polygonum Multiflorum (He Shou Wu, HSW)* | MOL000472 | Emodin | phosphatidylinositol-5-phosphate 4-kina | PIP4K2A | Pubchem | |
| *Polygonum Multiflorum (He Shou Wu, HSW)* | MOL000472 | Emodin | tyrosyl-DNA phosphodiesterase 1 | TDP1 | Pubchem | |
| *Polygonum Multiflorum (He Shou Wu, HSW)* | MOL000472 | Emodin | nuclear receptor subfamily 3 group C me | NR3C1 | Pubchem | |
| *Polygonum Multiflorum (He Shou Wu, HSW)* | MOL000472 | Emodin | androgen receptor | AR | Pubchem | |
| *Polygonum Multiflorum (He Shou Wu, HSW)* | MOL000472 | Emodin | cytochrome P450 family 19 subfamily A | CYP19A1 | Pubchem | |
| *Polygonum Multiflorum (He Shou Wu, HSW)* | MOL000472 | Emodin | peroxisome proliferator activated recept | PPARG | Pubchem | |
| *Polygonum Multiflorum (He Shou Wu, HSW)* | MOL000472 | Emodin | nuclear receptor subfamily 1 group H me | NR1H4 | Pubchem | |
| *Polygonum Multiflorum (He Shou Wu, HSW)* | MOL000472 | Emodin | casein kinase 2 alpha 2 | CSNK2A2 | Pubchem | |
| *Polygonum Multiflorum (He Shou Wu, HSW)* | MOL000472 | Emodin | retinoic acid receptor alpha | RARA | Pubchem | |
| *Polygonum Multiflorum (He Shou Wu, HSW)* | MOL000472 | Emodin | nuclear receptor subfamily 1 group I me | NR1I3 | Pubchem | |
| *Polygonum Multiflorum (He Shou Wu, HSW)* | MOL000472 | Emodin | epoxide hydrolase 2 | EPHX2 | Pubchem | |
| *Polygonum Multiflorum (He Shou Wu, HSW)* | MOL000472 | Emodin | histone deacetylase 9 | HDAC9 | Pubchem | |
| *Polygonum Multiflorum (He Shou Wu, HSW)* | MOL000472 | Emodin | GLI family zinc finger 3 | GLI3 | Pubchem | |
| *Polygonum Multiflorum (He Shou Wu, HSW)* | MOL000472 | Emodin | aldo-keto reductase family 1 member B | AKR1B1 | Pubchem | |
| *Polygonum Multiflorum (He Shou Wu, HSW)* | MOL000472 | Emodin | progesterone receptor | PGR | Pubchem | |
| *Polygonum Multiflorum (He Shou Wu, HSW)* | MOL000472 | Emodin | nuclear receptor subfamily 1, group I, me | NR1I2 | Pubchem | |
| *Polygonum Multiflorum (He Shou Wu, HSW)* | MOL000472 | Emodin | natriuretic peptide receptor 1 | NPR1 | Pubchem | |
| *Polygonum Multiflorum (He Shou Wu, HSW)* | MOL000472 | Emodin | N-terminal Xaa-Pro-Lys N-methyltransfe | NTMT1 | Pubchem | |
| *Polygonum Multiflorum (He Shou Wu, HSW)* | MOL000472 | Emodin | acetylcholinesterase (Cartwright blood g | ACHE | Pubchem | |
| *Polygonum Multiflorum (He Shou Wu, HSW)* | MOL000472 | Emodin | cytochrome P450 family 1 subfamily A n | CYP1A1 | Pubchem | |
| *Polygonum Multiflorum (He Shou Wu, HSW)* | MOL000702 | Guajavarin | enhancer of zeste 2 polycomb repressive | EZH2 | Pubchem | |
| *Polygonum Multiflorum (He Shou Wu, HSW)* | MOL000702 | Guajavarin | Aldose reductase (by homology) | AKR1B1 | Swiss Target Prediction | |
| *Polygonum Multiflorum (He Shou Wu, HSW)* | MOL000702 | Guajavarin | Carbonic anhydrase II | CA2 | Swiss Target Prediction | |
| *Polygonum Multiflorum (He Shou Wu, HSW)* | MOL000702 | Guajavarin | Carbonic anhydrase VII | CA7 | Swiss Target Prediction | |
| *Polygonum Multiflorum (He Shou Wu, HSW)* | MOL000702 | Guajavarin | Carbonic anhydrase XII | CA12 | Swiss Target Prediction | |
| *Polygonum Multiflorum (He Shou Wu, HSW)* | MOL000702 | Guajavarin | Carbonic anhydrase IV | CA4 | Swiss Target Prediction | |
| *Polygonum Multiflorum (He Shou Wu, HSW)* | MOL000702 | Guajavarin | NADPH oxidase 4 | NOX4 | Swiss Target Prediction | |
| *Polygonum Multiflorum (He Shou Wu, HSW)* | MOL000702 | Guajavarin | Adrenergic receptor alpha-2 | ADRA2C | Swiss Target Prediction | |
| *Polygonum Multiflorum (He Shou Wu, HSW)* | MOL000702 | Guajavarin | Acetylcholinesterase | ACHE | Swiss Target Prediction | |
| *Polygonum Multiflorum (He Shou Wu, HSW)* | MOL000476 | Physcion | UDP glucuronosyltransferase 1 family, p | UGT1A9 | STITCH | |
| *Polygonum Multiflorum (He Shou Wu, HSW)* | MOL000476 | Physcion | acetyl-CoA carboxylase alpha (2383 aa) | ACACA | STITCH | |
| *Polygonum Multiflorum (He Shou Wu, HSW)* | MOL000476 | Physcion | maltase-glucoamylase | MGAM | Pubchem | |
| *Polygonum Multiflorum (He Shou Wu, HSW)* | MOL000476 | Physcion | elastase, neutrophil expressed | ELANE | Pubchem | |
| *Polygonum Multiflorum (He Shou Wu, HSW)* | MOL000476 | Physcion | vitamin D receptor | VDR | Pubchem | |
| *Polygonum Multiflorum (He Shou Wu, HSW)* | MOL000476 | Physcion | phosphogluconate dehydrogenas | PGD | Pubchem | |
| *Polygonum Multiflorum (He Shou Wu, HSW)* | MOL013289 | Piceid | growth factor, augmenter of liver regene | GFER | Pubchem | |
| *Polygonum Multiflorum (He Shou Wu, HSW)* | MOL013289 | Piceid | DNA polymerase beta | POLB | Pubchem | |
| *Polygonum Multiflorum (He Shou Wu, HSW)* | MOL013289 | Piceid | potassium calcium-activated channel sul | KCNM4 | Pubchem | |
| *Polygonum Multiflorum (He Shou Wu, HSW)* | MOL013289 | Piceid | leucine rich repeat containing 26 | LRRC26 | Pubchem | |
| *Polygonum Multiflorum (He Shou Wu, HSW)* | MOL013289 | Piceid | potassium calcium-activated channel sul | KCNMA1 | Pubchem | |
| *Polygonum Multiflorum (He Shou Wu, HSW)* | MOL013289 | Piceid | potassium calcium-activated channel sul | KCNMB1 | Pubchem | |
| *Polygonum Multiflorum (He Shou Wu, HSW)* | MOL013289 | Piceid | potassium calcium-activated channel sul | KCNMB2 | Pubchem | |
| *Polygonum Multiflorum (He Shou Wu, HSW)* | | Procyanidin B1 3'-O-gallate | squalene epoxidase | SQLE | STITCH | |
| *Polygonum Multiflorum (He Shou Wu, HSW)* | MOL002268 | Rhien | retinoid X receptor, alpha | RXRA | STITCH | |
| *Polygonum Multiflorum (He Shou Wu, HSW)* | MOL002268 | Rhien | fat mass and obesity associated | FTO | STITCH | |
| *Polygonum Multiflorum (He Shou Wu, HSW)* | MOL002268 | Rhien | peroxisome proliferator-activated recept | PPARG | STITCH | |
| *Polygonum Multiflorum (He Shou Wu, HSW)* | MOL002268 | Rhien | uncoupling protein 1 (mitochondrial, prot | UCP1 | STITCH | |
| *Polygonum Multiflorum (He Shou Wu, HSW)* | MOL002268 | Rhien | peroxisome proliferator-activated recept | PPARA | STITCH | |
| *Polygonum Multiflorum (He Shou Wu, HSW)* | MOL002268 | Rhien | dynamin 1-like (736 aa) | DNM1L | STITCH | |
| *Polygonum Multiflorum (He Shou Wu, HSW)* | MOL002268 | Rhien | v-rel reticuloendotheliosis viral oncogene | RELA | STITCH | |
| *Polygonum Multiflorum (He Shou Wu, HSW)* | MOL002268 | Rhien | vascular endothelial growth factor A (41 | VEGFA | STITCH | |
| *Polygonum Multiflorum (He Shou Wu, HSW)* | MOL002268 | Rhien | nuclear factor of kappa light polypeptide | NFKB1 | STITCH | |
| *Polygonum Multiflorum (He Shou Wu, HSW)* | MOL002268 | Rhien | dynamin 1 | DNM1 | STITCH | |
| *Polygonum Multiflorum (He Shou Wu, HSW)* | MOL002268 | Rhien | heat shock protein 90 alpha family class | HSP90AB1 | Pubchem | |
| *Polygonum Multiflorum (He Shou Wu, HSW)* | MOL002268 | Rhien | protein tyrosine phosphatase non-recep | PTPN7 | Pubchem | |
| *Polygonum Multiflorum (He Shou Wu, HSW)* | MOL002268 | Rhien | arachidonate 15-lipoxygenase | ALOX15 | Pubchem | |
| *Polygonum Multiflorum (He Shou Wu, HSW)* | MOL002268 | Rhien | MCL1 apoptosis regulator, BCL2 family m | MCL1 | Pubchem | |
| *Polygonum Multiflorum (He Shou Wu, HSW)* | MOL002268 | Rhien | microtubule associated protein tau | MAPT | Pubchem | |
| *Polygonum Multiflorum (He Shou Wu, HSW)* | MOL002268 | Rhien | RB binding protein 9, serine hydrolase | RBBP9 | Pubchem | |
| *Polygonum Multiflorum (He Shou Wu, HSW)* | MOL002268 | Rhien | glutathione S-transferase omega 1 | GSTO1 | Pubchem | |
| *Polygonum Multiflorum (He Shou Wu, HSW)* | MOL002268 | Rhien | BCL2 like 1 | BCL2L1 | Pubchem | |
| *Polygonum Multiflorum (He Shou Wu, HSW)* | MOL002268 | Rhien | BLM RecQ like helicase | BLM | Pubchem | |
| *Polygonum Multiflorum (He Shou Wu, HSW)* | MOL002268 | Rhien | phospholipase A2 group VI | PLA2G7 | Pubchem | |
| *Polygonum Multiflorum (He Shou Wu, HSW)* | MOL002268 | Rhien | DNA polymerase beta | POLB | Pubchem | |
| *Polygonum Multiflorum (He Shou Wu, HSW)* | MOL002268 | Rhien | platelet activating factor acetylhydrolase | PAFAH2 | Pubchem | |
| *Polygonum Multiflorum (He Shou Wu, HSW)* | MOL002268 | Rhien | CTD small phosphatase 1 | CTDSP1 | Pubchem | |
| *Polygonum Multiflorum (He Shou Wu, HSW)* | MOL002268 | Rhien | C-C motif chemokine receptor 6 | CCR6 | Pubchem | |
| *Polygonum Multiflorum (He Shou Wu, HSW)* | MOL002268 | Rhien | hexokinase domain containing 1 | HKDC1 | Pubchem | |
| *Polygonum Multiflorum (He Shou Wu, HSW)* | MOL002268 | Rhien | bromodomain adjacent to zinc finger do | BAZ2B | Pubchem | |
| *Polygonum Multiflorum (He Shou Wu, HSW)* | MOL002268 | Rhien | DNA methyltransferase 1 | DNMT1 | Pubchem | |
| *Polygonum Multiflorum (He Shou Wu, HSW)* | MOL002268 | Rhien | DNA polymerase kappa | POLK | Pubchem | |
| *Polygonum Multiflorum (He Shou Wu, HSW)* | MOL002268 | Rhien | DNA polymerase iota | POLI | Pubchem | |
| *Polygonum Multiflorum (He Shou Wu, HSW)* | MOL002268 | Rhien | DNA polymerase eta | POLH | Pubchem | |
| *Polygonum Multiflorum (He Shou Wu, HSW)* | MOL002268 | Rhien | C-X-C motif chemokine receptor 6 | CXCR6 | Pubchem | |
| *Polygonum Multiflorum (He Shou Wu, HSW)* | MOL002268 | Rhien | glutaminase | GLS | Pubchem | |
| *Polygonum Multiflorum (He Shou Wu, HSW)* | MOL002268 | Rhien | tumor protein p53 | TP53 | Pubchem | |
| *Polygonum Multiflorum (He Shou Wu, HSW)* | MOL002268 | Rhien | RB binding protein 8, endonuclease | RBBP8 | Pubchem | |
| *Polygonum Multiflorum (He Shou Wu, HSW)* | MOL002268 | Rhien | SIX homeobox 1 | SIX1 | Pubchem | |
| *Polygonum Multiflorum (He Shou Wu, HSW)* | MOL002268 | Rhien | WRN RecQ like helicase | WRN | Pubchem | |
| *Polygonum Multiflorum (He Shou Wu, HSW)* | MOL002268 | Rhien | RAR related orphan receptor C | RORC | Pubchem | |
| *Polygonum Multiflorum (He Shou Wu, HSW)* | MOL002268 | Rhien | phosphatidylinositol-5-phosphate 4-kina | PIP4K2A | Pubchem | |
| *Polygonum Multiflorum (He Shou Wu, HSW)* | MOL002268 | Rhien | sirtuin 5 | SIRT5 | Pubchem | |
| *Polygonum Multiflorum (He Shou Wu, HSW)* | MOL002268 | Rhien | protein arginine methyltransferase 1 | PRMT1 | Pubchem | |
| *Polygonum Multiflorum (He Shou Wu, HSW)* | MOL002268 | Rhien | caspase 6 | CASP6 | Pubchem | |
| *Polygonum Multiflorum (He Shou Wu, HSW)* | MOL002268 | Rhien | flap structure-specific endonuclease 1 | FEN1 | Pubchem | |
| *Polygonum Multiflorum (He Shou Wu, HSW)* | MOL002268 | Rhien | estrogen receptor 1 | ESR1 | Pubchem | |
| *Polygonum Multiflorum (He Shou Wu, HSW)* | MOL002268 | Rhien | nuclear factor, erythroid 2 like 2 | NFE2L2 | Pubchem | |
| *Polygonum Multiflorum (He Shou Wu, HSW)* | MOL002268 | Rhien | butyrylcholinesterase | BCHE | Pubchem | |
| *Polygonum Multiflorum (He Shou Wu, HSW)* | MOL002268 | Rhien | histone deacetylase 9 | HDAC9 | Pubchem | |
