# Supplementary material for: Network Pharmacology Approach for Predicting Targets of Zishen Yutai Pills on Premature Ovarian Insufficiency
Source: Evid Based Complement Alternat Med. 2021 Aug 4;2021:8215454. doi: 10.1155/2021/8215454 (PMC8357500; doi:10.1155/2021/8215454)
Supplement: Supplementary Materials — Supplementary Table 1: active compounds of each herb in ZSYTP. Supplementary Table 2: corresponding targets of active compounds: (A) two target genes of SDH; (B) 138 target genes of GQZ; (C) 202 target genes of TSZ; (D) 25 target genes of BJT; (E) 121 target genes of DS; (F) 11 target genes of BZ; (G) 283 target genes of DZ; (H) 13 target genes of SR; (J) 112 target genes of RS; (K) 76 target genes of SJS; (L) 115 target genes of AY; (M) 149 target genes of HSW; (N) 68 target genes of EJ; (P) 46 target genes of LJJ. Supplementary Table 3: POI targets. Supplementary Table 4: compounds and targets attributes of C-T network. Supplementary Table 5: data of compound-target network. Supplementary Table 6: analysed C-T network: (A) active compounds of C-T network and corresponding degrees; (B) targets of C-T network and corresponding degrees. Supplementary Table 7: ZSYTP core PPI network. Supplementary Table 8: gene ontology and KEGG analysis: (A) GO-BP analysis; (B) GO-MF analysis; (C) GO-CC analysis; (D) KEGG pathway enrichment analysis. [file 8215454.f1.zip › 8215454.f1/ST2N EJ Herb Targets.pdf]

Supplementary Table S2N. Corresponding Targets of EJ

Herb Name	Mol ID	Chemical Name	Protein Name	Symbol	Sources		
Colla Corrii Asini (EJiao, EJ)	MOL000054	Arginine	cytochrome P450 family 1 subfamily	CYP1A2	PubChem		
Colla Corrii Asini (EJiao, EJ)	MOL000054	Arginine	nitric oxide synthase 3	NOS3	PubChem	DrugBank	STITCH
Colla Corrii Asini (EJiao, EJ)	MOL000054	Arginine	nitric oxide synthase 2	NOS2	PubChem	DrugBank	STITCH
Colla Corrii Asini (EJiao, EJ)	MOL000054	Arginine	nitric oxide synthase 1	NOS1	PubChem	STITCH	
Colla Corrii Asini (EJiao, EJ)	MOL000054	Arginine	Solute Carrier Family 7 Member 3	SLC7A3	DrugBank		
Colla Corrii Asini (EJiao, EJ)	MOL000054	Arginine	Antizyme Inhibitor 2	AZIN2	DrugBank		
Colla Corrii Asini (EJiao, EJ)	MOL000054	Arginine	Arginine decarboxylase	ASL	DrugBank		
Colla Corrii Asini (EJiao, EJ)	MOL000054	Arginine	Argininosuccinate lyase	SLC7A1	DrugBank		
Colla Corrii Asini (EJiao, EJ)	MOL000054	Arginine	Arginase-2, mitochondrial	ARG2	DrugBank		
Colla Corrii Asini (EJiao, EJ)	MOL000054	Arginine	Solute Carrier Family 7 Member 4	SLC7A4	DrugBank		
Colla Corrii Asini (EJiao, EJ)	MOL000054	Arginine	Argininosuccinate synthase	ASS1	DrugBank	STITCH	
Colla Corrii Asini (EJiao, EJ)	MOL000054	Arginine	protein arginine methyltransferase 1	PRMT1	STITCH		
Colla Corrii Asini (EJiao, EJ)	MOL000054	Arginine	serum response factor (c-fos serum r	SRF	STITCH		
Colla Corrii Asini (EJiao, EJ)	MOL000054	Arginine	coactivator-associated arginine meth	CARM1	STITCH		
Colla Corrii Asini (EJiao, EJ)	MOL000054	Arginine	protein arginine methyltransferase	PRMT5	STITCH		
Colla Corrii Asini (EJiao, EJ)	MOL000054	Arginine	adenosylmethionine decarboxylase 1	AMD1	STITCH		
Colla Corrii Asini (EJiao, EJ)	MOL000054	Arginine	ornithine decarboxylase 1	ODC1	STITCH		
Colla Corrii Asini (EJiao, EJ)	MOL000054	Arginine	Glucagon	GCG	STITCH		
Colla Corrii Asini (EJiao, EJ)	MOL000071	Histidine	Carbonic Anhydrase 1	CA1	Pubchem		
Colla Corrii Asini (EJiao, EJ)	MOL000071	Histidine	Carbonic Anhydrase 2	CA2	Pubchem		
Colla Corrii Asini (EJiao, EJ)	MOL000071	Histidine	Carbonic Anhydrase 4	CA4	Pubchem		
Colla Corrii Asini (EJiao, EJ)	MOL000071	Histidine	Carbonic Anhydrase 9	CA9	Pubchem		
Colla Corrii Asini (EJiao, EJ)	MOL000071	Histidine	Carbonic Anhydrase 7	CA7	Pubchem		
Colla Corrii Asini (EJiao, EJ)	MOL000071	Histidine	Carbonic Anhydrase 14	CA14	Pubchem		
Colla Corrii Asini (EJiao, EJ)	MOL000071	Histidine	Carbonic Anhydrase 5A	CA5A	Pubchem		
Colla Corrii Asini (EJiao, EJ)	MOL000071	Histidine	Carbonic Anhydrase 5B	CA5B	Pubchem		
Colla Corrii Asini (EJiao, EJ)	MOL000071	Histidine	Carbonic Anhydrase 6	CA6	Pubchem		
Colla Corrii Asini (EJiao, EJ)	MOL000071	Histidine	solute carrier family 15 member 4	SLC15A4	Pubchem		
Colla Corrii Asini (EJiao, EJ)	MOL000071	Histidine	solute carrier family 15 member 3	SLC15A3	Pubchem		
Colla Corrii Asini (EJiao, EJ)	MOL000071	Histidine	signal regulatory protein alpha	SIRPA	Pubchem		
Colla Corrii Asini (EJiao, EJ)	MOL000071	Histidine	CD47 molecule	CD47	Pubchem		
Colla Corrii Asini (EJiao, EJ)	MOL000071	Histidine	solute carrier family 7 member 5	SLC7A5	Pubchem		
Colla Corrii Asini (EJiao, EJ)	MOL000071	Histidine	Histidine decarboxylase	HDC	DrugBank	STITCH	
Colla Corrii Asini (EJiao, EJ)	MOL000071	Histidine	Histidine--tRNA ligase, cytoplasmic	HARS	DrugBank		
Colla Corrii Asini (EJiao, EJ)	MOL000071	Histidine	Sodium-coupled neutral amino acid t	SLC38A3	DrugBank		
Colla Corrii Asini (EJiao, EJ)	MOL000071	Histidine	Histidine ammonia-lyase	HAL	DrugBank		
Colla Corrii Asini (EJiao, EJ)	MOL000071	Histidine	eukaryotic translation elongation fact	EEF2	STITCH		
Colla Corrii Asini (EJiao, EJ)	MOL000071	Histidine	catalase	CAT	STITCH		
Colla Corrii Asini (EJiao, EJ)	MOL000071	Histidine	Myoglobin	MB	STITCH		
Colla Corrii Asini (EJiao, EJ)	MOL000071	Histidine	Tyrosinase	TYR	STITCH		
Colla Corrii Asini (EJiao, EJ)	MOL000071	Histidine	PR domain containing 10 (1160 aa)	PRDM10	STITCH		
Colla Corrii Asini (EJiao, EJ)	MOL000071	Histidine	glutamate-ammonia ligase	GLUL	STITCH		
Colla Corrii Asini (EJiao, EJ)	MOL000071	Histidine	solute carrier family 39 (zinc transpor	SLC39A6	STITCH		
Colla Corrii Asini (EJiao, EJ)	MOL000071	Histidine	heme oxygenase (decycling) 2	HMOX2	STITCH		
Colla Corrii Asini (EJiao, EJ)	MOL000055	Lysine	solute carrier family 7 member 1	SLC7A1	DrugBank		
Colla Corrii Asini (EJiao, EJ)	MOL000055	Lysine	Solute Carrier Family 7 Member 4	SLC7A4	DrugBank		
Colla Corrii Asini (EJiao, EJ)	MOL000055	Lysine	Solute Carrier Family 7 Member 3	SLC7A3	DrugBank		
Colla Corrii Asini (EJiao, EJ)	MOL000055	Lysine	Solute Carrier Family 7 Member 2	SLC7A2	DrugBank		
Colla Corrii Asini (EJiao, EJ)	MOL000055	Lysine	Lysine--tRNA ligase	KARS	DrugBank		
Colla Corrii Asini (EJiao, EJ)	MOL000055	Lysine	H3 histone, family 3A	H3F3A	STITCH		
Colla Corrii Asini (EJiao, EJ)	MOL000055	Lysine	H3 histone, family 3B	H3F3B	STITCH		
Colla Corrii Asini (EJiao, EJ)	MOL000055	Lysine	histone cluster 1, H4a	HIST1H4A	STITCH		
Colla Corrii Asini (EJiao, EJ)	MOL000055	Lysine	histone cluster 1, H4f	HIST1H4F	STITCH		
Colla Corrii Asini (EJiao, EJ)	MOL000055	Lysine	histone cluster 2, H4a	HIST2H4A	STITCH		
Colla Corrii Asini (EJiao, EJ)	MOL000055	Lysine	histone cluster 1, H4c	HIST1H4C	STITCH		
Colla Corrii Asini (EJiao, EJ)	MOL000055	Lysine	histone cluster 1, H4b	HIST1H4B	STITCH		
Colla Corrii Asini (EJiao, EJ)	MOL000055	Lysine	histone cluster 2, H4b	HIST2H4B	STITCH		
Colla Corrii Asini (EJiao, EJ)	MOL000055	Lysine	histone cluster 1, H4e	HIST1H4E	STITCH		
Colla Corrii Asini (EJiao, EJ)	MOL000055	Lysine	histone cluster 4, H4	HIST4H4	STITCH		
Colla Corrii Asini (EJiao, EJ)	MOL003971	Threonine	Threonine--tRNA ligase, cytoplasmic	TARS	DrugBank		
Colla Corrii Asini (EJiao, EJ)	MOL003971	Threonine	Threonine--tRNA ligase, mitochondria	TARS2	DrugBank		
Colla Corrii Asini (EJiao, EJ)	MOL003971	Threonine	neurotensin receptor 2	NTSR2	STITCH		
Colla Corrii Asini (EJiao, EJ)	MOL003971	Threonine	neurotensin receptor 1 (high affinity	NTSR1	STITCH		
Colla Corrii Asini (EJiao, EJ)	MOL003971	Threonine	taspase, threonine aspartase, 1	TASP1	STITCH		
Colla Corrii Asini (EJiao, EJ)	MOL003971	Threonine	protein phosphatase 1, catalytic subu	PPP1CB	STITCH		
Colla Corrii Asini (EJiao, EJ)	MOL003971	Threonine	Neurotensin	NTS	STITCH		
