# Supplementary material for: Network Pharmacology Approach for Predicting Targets of Zishen Yutai Pills on Premature Ovarian Insufficiency
Source: Evid Based Complement Alternat Med. 2021 Aug 4;2021:8215454. doi: 10.1155/2021/8215454 (PMC8357500; doi:10.1155/2021/8215454)
Supplement: Supplementary Materials — Supplementary Table 1: active compounds of each herb in ZSYTP. Supplementary Table 2: corresponding targets of active compounds: (A) two target genes of SDH; (B) 138 target genes of GQZ; (C) 202 target genes of TSZ; (D) 25 target genes of BJT; (E) 121 target genes of DS; (F) 11 target genes of BZ; (G) 283 target genes of DZ; (H) 13 target genes of SR; (J) 112 target genes of RS; (K) 76 target genes of SJS; (L) 115 target genes of AY; (M) 149 target genes of HSW; (N) 68 target genes of EJ; (P) 46 target genes of LJJ. Supplementary Table 3: POI targets. Supplementary Table 4: compounds and targets attributes of C-T network. Supplementary Table 5: data of compound-target network. Supplementary Table 6: analysed C-T network: (A) active compounds of C-T network and corresponding degrees; (B) targets of C-T network and corresponding degrees. Supplementary Table 7: ZSYTP core PPI network. Supplementary Table 8: gene ontology and KEGG analysis: (A) GO-BP analysis; (B) GO-MF analysis; (C) GO-CC analysis; (D) KEGG pathway enrichment analysis. [file 8215454.f1.zip › 8215454.f1/ST2P LJJ Herb Targets.pdf]

Supplementary Table S2P. Corresponding Targets of LJJ

Herb Name	Mol ID	Chemical Name	Protein Name	Symbol	Sources
Colla Cornus Cervi (Lu Jiao Jiao, LJJ)		Calcium Phosphate	alpha-2-HS-glycoprotein	AHSG	STITCH
Colla Cornus Cervi (Lu Jiao Jiao, LJJ)		Calcium Phosphate	carbonic anhydrase X;	CA10	STITCH
Colla Cornus Cervi (Lu Jiao Jiao, LJJ)		Calcium Phosphate	matrix metallopeptidase 20	MMP20	STITCH
Colla Cornus Cervi (Lu Jiao Jiao, LJJ)		Calcium Phosphate	integrin-binding sialoprotein;	IBSP	STITCH
Colla Cornus Cervi (Lu Jiao Jiao, LJJ)		Calcium Phosphate	secreted phosphoprotein 1	SSP1	STITCH
Colla Cornus Cervi (Lu Jiao Jiao, LJJ)		Calcium Phosphate	ameloblastin (enamel matrix protein)	AMBN	STITCH
Colla Cornus Cervi (Lu Jiao Jiao, LJJ)		Calcium Phosphate	enamelin	ENAM	STITCH
Colla Cornus Cervi (Lu Jiao Jiao, LJJ)		Calcium Phosphate	dentin sialophosphoprotein	DSPP	STITCH
Colla Cornus Cervi (Lu Jiao Jiao, LJJ)		Calcium Phosphate	matrix metallopeptidase 25;	MMP25	STITCH
Colla Cornus Cervi (Lu Jiao Jiao, LJJ)		Calcium Phosphate	mitochondrially encoded cytochrome c ox	MT-CO3	STITCH
Colla Cornus Cervi (Lu Jiao Jiao, LJJ)		Calcium Phosphate	Extracellular calcium-sensing receptor	CASR	DrugBank
Colla Cornus Cervi (Lu Jiao Jiao, LJJ)		Calcium Phosphate	Calreticulin	CALR	DrugBank
Colla Cornus Cervi (Lu Jiao Jiao, LJJ)		Calcium Phosphate	Calcium and integrin-binding protein 1	CIB1	DrugBank
Colla Cornus Cervi (Lu Jiao Jiao, LJJ)		Calcium Phosphate	Programmed cell death protein 6	PDCD6	DrugBank
Colla Cornus Cervi (Lu Jiao Jiao, LJJ)		Calcium Phosphate	Sorcin	SRI	DrugBank
Colla Cornus Cervi (Lu Jiao Jiao, LJJ)		Calcium Phosphate	Calcineurin B homologous protein 1	CHP1	DrugBank
Colla Cornus Cervi (Lu Jiao Jiao, LJJ)		Calcium Phosphate	SPARC	SPARC	DrugBank
Colla Cornus Cervi (Lu Jiao Jiao, LJJ)		Calcium Phosphate	Calnexin	CANX	DrugBank
Colla Cornus Cervi (Lu Jiao Jiao, LJJ)		Calcium Phosphate	Fibrillin-2	FBN2	DrugBank
Colla Cornus Cervi (Lu Jiao Jiao, LJJ)		Calcium Phosphate	Protein S100-B	S100B	DrugBank
Colla Cornus Cervi (Lu Jiao Jiao, LJJ)		Calcium Phosphate	Calsequestrin-2	CASQ2	DrugBank
Colla Cornus Cervi (Lu Jiao Jiao, LJJ)		Calcium Phosphate	Regucalcin	RGN	DrugBank
Colla Cornus Cervi (Lu Jiao Jiao, LJJ)		Calcium Phosphate	Peflin	PEF1	DrugBank
Colla Cornus Cervi (Lu Jiao Jiao, LJJ)		Calcium Phosphate	Protein S100-A6	S100A6	DrugBank
Colla Cornus Cervi (Lu Jiao Jiao, LJJ)		Calcium Phosphate	Translationally-controlled tumor protein	TPT1	DrugBank
Colla Cornus Cervi (Lu Jiao Jiao, LJJ)		Calcium Phosphate	Calcium and integrin-binding family mem	CIB2	DrugBank
Colla Cornus Cervi (Lu Jiao Jiao, LJJ)		Calcium Phosphate	Protein S100-A13	S100A13	DrugBank
Colla Cornus Cervi (Lu Jiao Jiao, LJJ)		Calcium Phosphate	Calsequestrin-1	CASQ1	DrugBank
Colla Cornus Cervi (Lu Jiao Jiao, LJJ)		Calcium Phosphate	Nucleobindin-1	NUCB1	DrugBank
Colla Cornus Cervi (Lu Jiao Jiao, LJJ)		Calcium Phosphate	Nucleobindin-2	NUCB2	DrugBank
Colla Cornus Cervi (Lu Jiao Jiao, LJJ)		Calcium Phosphate	Calmodulin	CALM1	DrugBank
Colla Cornus Cervi (Lu Jiao Jiao, LJJ)		Calcium Phosphate	Fibrillin-3	FBN3	DrugBank
Colla Cornus Cervi (Lu Jiao Jiao, LJJ)		Calcium Phosphate	Grancalcin	GCA	DrugBank
Colla Cornus Cervi (Lu Jiao Jiao, LJJ)		Calcium Phosphate	Calcium-dependent secretion activator 1	GADPS	DrugBank
Colla Cornus Cervi (Lu Jiao Jiao, LJJ)		Calcium Phosphate	Calmodulin-2	CALM2	DrugBank
Colla Cornus Cervi (Lu Jiao Jiao, LJJ)		Calcium Phosphate	Calmodulin-3	CALM3	DrugBank
Colla Cornus Cervi (Lu Jiao Jiao, LJJ)		Calcium Phosphate	Protein S100-A16	S100A16	DrugBank
Colla Cornus Cervi (Lu Jiao Jiao, LJJ)		Calcium Phosphate	Calretinin	CALB2	DrugBank
Colla Cornus Cervi (Lu Jiao Jiao, LJJ)		Calcium Phosphate	Calcyphosin	CAPS	DrugBank
Colla Cornus Cervi (Lu Jiao Jiao, LJJ)		Calcium Phosphate	Calcium-dependent secretion activator 2	CADPS2	DrugBank
Colla Cornus Cervi (Lu Jiao Jiao, LJJ)		Calcium Phosphate	Calreticulin-3	CALR3	DrugBank
Colla Cornus Cervi (Lu Jiao Jiao, LJJ)		Calcium Phosphate	Neurexin-1	NRXN1	DrugBank
Colla Cornus Cervi (Lu Jiao Jiao, LJJ)		Calcium Phosphate	Neuronal calcium sensor 1	NCS1	DrugBank
Colla Cornus Cervi (Lu Jiao Jiao, LJJ)		Calcium Carbonate	solute carrier family 4, sodium bicarbonat	SLC4A4	STITCH
Colla Cornus Cervi (Lu Jiao Jiao, LJJ)		Calcium Carbonate	solute carrier family 4, sodium bicarbonat	SLC4A5	STITCH
Colla Cornus Cervi (Lu Jiao Jiao, LJJ)		Calcium Carbonate	solute carrier family 4, sodium bicarbonat	SLC4A9	STITCH
