# Supplementary material for: Network Pharmacology Approach for Predicting Targets of Zishen Yutai Pills on Premature Ovarian Insufficiency
Source: Evid Based Complement Alternat Med. 2021 Aug 4;2021:8215454. doi: 10.1155/2021/8215454 (PMC8357500; doi:10.1155/2021/8215454)
Supplement: Supplementary Materials — Supplementary Table 1: active compounds of each herb in ZSYTP. Supplementary Table 2: corresponding targets of active compounds: (A) two target genes of SDH; (B) 138 target genes of GQZ; (C) 202 target genes of TSZ; (D) 25 target genes of BJT; (E) 121 target genes of DS; (F) 11 target genes of BZ; (G) 283 target genes of DZ; (H) 13 target genes of SR; (J) 112 target genes of RS; (K) 76 target genes of SJS; (L) 115 target genes of AY; (M) 149 target genes of HSW; (N) 68 target genes of EJ; (P) 46 target genes of LJJ. Supplementary Table 3: POI targets. Supplementary Table 4: compounds and targets attributes of C-T network. Supplementary Table 5: data of compound-target network. Supplementary Table 6: analysed C-T network: (A) active compounds of C-T network and corresponding degrees; (B) targets of C-T network and corresponding degrees. Supplementary Table 7: ZSYTP core PPI network. Supplementary Table 8: gene ontology and KEGG analysis: (A) GO-BP analysis; (B) GO-MF analysis; (C) GO-CC analysis; (D) KEGG pathway enrichment analysis. [file 8215454.f1.zip › 8215454.f1/ST3 POI Targets.pdf]

Table S3 POI targets

Protein Name	Gene Symbol
BRCA2 DNA repair associated	BRCA2
FMRP translational regulator 1	FMR1
tumour protein p53	TP53
forkhead box L2	FOXL2
follicle stimulating hormone receptor	FSHR
nuclear receptor subfamily 5 group A member 1	NR5A1
Tumour necrosis Factor	TNF
methylenetetrahydrofolate reductase	MTHER
interleukin 6	IL6
vascular endothelial growth factor A	VEGFA
Sirtuin 1	SIRT1
Estrogen Receptor 2	ESR2
Growth differentiation factor 9	GDF9
Transforming Growth factor beta 1	TGFB1
Androgen receptor	AR
adiponectin, C1Q and collagen domain containing	ADIPOQ
matrix metallopeptidase 9	MMP9
X-ray repair cross complementing 2	XRCC2
Leptin	LEP
C-reactive Protein	CRP
Angiotensin I converting enzyme	ACE
insulin like growth factor 1	IGF1
CF transmembrane conductance regulator	CFTR
interleukin 10	IL10
Toll like receptor 4	TLR4
bone morphogenetic protein 15	BMP15
brain derived neurotrophic factor	BDNF
anti-Mullerian Hormone	AMH
matrix metallopeptidase 2	MMP2
cyclin dependent kinase inhibitor 1B	CDKN1B
catechol-O-methyltransferase	COMT
GC vitamin D binding protein	GC
poly(ADP-ribose) polymerase 1	PARP1
tumour protein p53	TP63
NOBOX oogenesis homeobox	NOBOX
FA complementation group M	FANCM
lamin A/C	LMNA
minichromosome maintenance 8 homologous recombination repair factor	MCM8
major histocompatibility complex, class I, G	HLA-G
intercellular adhesion molecule 1	ICAM1
Nitric Oxide Synthase 3	NOS3
Inhibin subunit alpha	INHA
endothelin 1	EDN1
advanced glycosylation end-product specific receptor	AGER
glutathione S-transferase theta 1	GSTT1
Galectin 3	LGALS3
WT1 Transcription factor	WT1
Forkhead box O3	FOXO3
mutS homolog 5	MSH5
basonuclin 1	BNC1
fms related receptor tyrosine kinase 1	FLT1
angiotensin II receptor type 1	AGTR1
BUB1 mitotic checkpoint serine/threonine kinase B	BUB1B
interleukin 17A	IL17A
natriuretic peptide B	NPPB
synuclein alpha	SNCA
cytochrome P450 family 11 subfamily A member 1	CYP11A1
mannose binding lectin 2	MBL2
matrix metallopeptidase 3	MMP3
Stromal antigen 3	STAG3
diaphanous related formin 2	DIAPH2
collagen type I alpha 1 chain	COL1A1
TIMP metallopeptidase inhibitor 1	TIMP1
folliculogenesis specific bHLH transcription factor	FIGLA
TNF receptor superfamily member 11b	TNFRSF11B
nuclear receptor subfamily 3 group C member 1	NR3C1
Fibrillin 1	FBN1
FTO alpha-ketoglutarate dependent dioxygenase	FTO
alkaline phosphatase, biomineralization associated	ALPL
fragile site, folic acid type, rare, fra(X)(q27.3) A (macroorchidism, mental retardation)	FRAXA
C-C motif chemokine ligand 5	CCL5
bone morphogenetic protein 4	BMP4
synaptonemal complex central element protein 1	SYCE1
aldehyde dehydrogenase 2 family member	ALDH2
fibroblast growth factor receptor 3	FGFR3
von Hippel-Lindau tumor suppressor	VHL
TNF superfamily member 11	TNFSF11
helicase for meiosis 1	HFM1
DNA polymerase gamma, catalytic subunit	POLG
galactose-1-phosphate uridylyltransferase	GALT
Prolactin	PRL
insulin like growth factor binding protein 1	IGFBP1
exportin 1	XBO1
Y-box binding protein 1	YBX1
vascular cell adhesion molecule 1	VCAM1
Retinol binding protein 4	RBP4
beta-2-microglobulin	B2M
S100 calcium binding protein A9	S100A9
matrix metallopeptidase 13	MMP13
serine peptidase inhibitor Kazal type 1	SPINK1
X-ray repair cross complementing 6	XRCC6
minichromosome maintenance 9 homologous recombination repair factor	MCM9
spalt like transcription factor 4	SALL4
FA complementation group A	FANCA
PSMC3 interacting protein	PSMC3IP
notch receptor 2	NOTCH2
paired like homeodomain 2	PITX2
alanyl-tRNA synthetase 2, mitochondrial	AARS2
nuclear receptor interacting protein 1	NRIP1
phosphomannomutase 2	PMM2
hydroxysteroid 17-beta dehydrogenase 4	HSD17B4
ADAM metallopeptidase with thrombospondin type 1 motif 4	ADAMTS4
DNA meiotic recombinase 1	DMC1
Leptin	LEPQTL1
leucyl-tRNA synthetase 2, mitochondrial	LARS2
mitochondrially encoded cytochrome c oxidase I	MT-CO1
propionyl-CoA carboxylase subunit alpha	PCCA
microtubule actin crosslinking factor 1	MACF1
propionyl-CoA carboxylase subunit beta	PCCB
WD repeat domain 62	WDR62
cytoplasmic polyadenylation element binding protein 1	CPEB1
FKBP prolyl isomerase 14	FKBP14
HLA complex P5	HCP5
tousled like kinase 1	TLK1
abraxas 1, BRCA1 A complex subunit	ABRAXAS1
nanos C2HC-type zinc finger 3	NANOS3
microRNA 449b	MIR449B
microRNA 379	MIR379
transmembrane protein 150B	TMEM150B
