# Supplementary material for: Network Pharmacology Approach for Predicting Targets of Zishen Yutai Pills on Premature Ovarian Insufficiency
Source: Evid Based Complement Alternat Med. 2021 Aug 4;2021:8215454. doi: 10.1155/2021/8215454 (PMC8357500; doi:10.1155/2021/8215454)
Supplement: Supplementary Materials — Supplementary Table 1: active compounds of each herb in ZSYTP. Supplementary Table 2: corresponding targets of active compounds: (A) two target genes of SDH; (B) 138 target genes of GQZ; (C) 202 target genes of TSZ; (D) 25 target genes of BJT; (E) 121 target genes of DS; (F) 11 target genes of BZ; (G) 283 target genes of DZ; (H) 13 target genes of SR; (J) 112 target genes of RS; (K) 76 target genes of SJS; (L) 115 target genes of AY; (M) 149 target genes of HSW; (N) 68 target genes of EJ; (P) 46 target genes of LJJ. Supplementary Table 3: POI targets. Supplementary Table 4: compounds and targets attributes of C-T network. Supplementary Table 5: data of compound-target network. Supplementary Table 6: analysed C-T network: (A) active compounds of C-T network and corresponding degrees; (B) targets of C-T network and corresponding degrees. Supplementary Table 7: ZSYTP core PPI network. Supplementary Table 8: gene ontology and KEGG analysis: (A) GO-BP analysis; (B) GO-MF analysis; (C) GO-CC analysis; (D) KEGG pathway enrichment analysis. [file 8215454.f1.zip › 8215454.f1/ST4 Compounds and Targets Attributes of C-T network.pdf]

Node	Type	SDH	GQZ	TSZ	BJT	DS	BZ	DZ	SR	RS	SJS	AY	HSW	EJ	LJJ
POLB	gene	0	0	0	0	0	0	0	0	0	0	0	0	0	0
POLB	gene	0	0	0	0	0	0	0	0	0	0	0	0	0	0
POLB	gene	0	0	0	0	0	0	0	0	0	0	0	0	0	0
POLB	gene	0	0	0	0	0	0	0	0	0	0	0	0	0	0
POLB	gene	0	0	0	0	0	0	0	0	0	0	0	0	0	0
POLB	gene	0	0	0	0	0	0	0	0	0	0	0	0	0	0
PPARA	gene	0	0	0	0	0	0	0	0	0	0	0	0	0	0
ABCB1	gene	0	0	0	0	0	0	0	0	0	0	0	0	0	0
CYP1A2	gene	0	0	0	0	0	0	0	0	0	0	0	0	0	0
APOB	gene	0	0	0	0	0	0	0	0	0	0	0	0	0	0
CCL2	gene	0	0	0	0	0	0	0	0	0	0	0	0	0	0
HMOX1	gene	0	0	0	0	0	0	0	0	0	0	0	0	0	0
LDLR	gene	0	0	0	0	0	0	0	0	0	0	0	0	0	0
BDNF	gene	0	0	0	0	0	0	0	0	0	0	0	0	0	0
RAPGEF1	gene	0	0	0	0	0	0	0	0	0	0	0	0	0	0
SCD	gene	0	0	0	0	0	0	0	0	0	0	0	0	0	0
CYP19A1	gene	0	0	0	0	0	0	0	0	0	0	0	0	0	0
CA7	gene	0	0	0	0	0	0	0	0	0	0	0	0	0	0
ABCC1	gene	0	0	0	0	0	0	0	0	0	0	0	0	0	0
HSD17B1	gene	0	0	0	0	0	0	0	0	0	0	0	0	0	0
CA12	gene	0	0	0	0	0	0	0	0	0	0	0	0	0	0
SHBG	gene	0	0	0	0	0	0	0	0	0	0	0	0	0	0
CA4	gene	0	0	0	0	0	0	0	0	0	0	0	0	0	0
CYP1B1	gene	0	0	0	0	0	0	0	0	0	0	0	0	0	0
CBR1	gene	0	0	0	0	0	0	0	0	0	0	0	0	0	0
POLB	gene	0	0	0	0	0	0	0	0	0	0	0	0	0	0
GSK3B	gene	0	0	0	0	0	0	0	0	0	0	0	0	0	0
PRKAB2	gene	0	0	0	0	0	0	0	0	0	0	0	0	0	0
PRKAG3	gene	0	0	0	0	0	0	0	0	0	0	0	0	0	0
PRKAG1	gene	0	0	0	0	0	0	0	0	0	0	0	0	0	0
PRKAA1	gene	0	0	0	0	0	0	0	0	0	0	0	0	0	0
PRKAA2	gene	0	0	0	0	0	0	0	0	0	0	0	0	0	0
PRKAB1	gene	0	0	0	0	0	0	0	0	0	0	0	0	0	0
PRKAG2	gene	0	0	0	0	0	0	0	0	0	0	0	0	0	0
DPP4	gene	0	0	0	0	0	0	0	0	0	0	0	0	0	0
F2	gene	0	0	0	0	0	0	0	0	0	0	0	0	0	0
POLB	gene	0	0	0	0	0	0	0	0	0	0	0	0	0	0
GSK3B	gene	0	0	0	0	0	0	0	0	0	0	0	0	0	0
PRKAB2	gene	0	0	0	0	0	0	0	0	0	0	0	0	0	0
PRKAG3	gene	0	0	0	0	0	0	0	0	0	0	0	0	0	0
PRKAG1	gene	0	0	0	0	0	0	0	0	0	0	0	0	0	0
PRKAA1	gene	0	0	0	0	0	0	0	0	0	0	0	0	0	0
PRKAA2	gene	0	0	0	0	0	0	0	0	0	0	0	0	0	0
PRKAB1	gene	0	0	0	0	0	0	0	0	0	0	0	0	0	0
PRKAG2	gene	0	0	0	0	0	0	0	0	0	0	0	0	0	0
DPP4	gene	0	0	0	0	0	0	0	0	0	0	0	0	0	0
F2	gene	0	0	0	0	0	0	0	0	0	0	0	0	0	0
POLB	gene	0	0	0	0	0	0	0	0	0	0	0	0	0	0
GSK3B	gene	0	0	0	0	0	0	0	0	0	0	0	0	0	0
PRKAB2	gene	0	0	0	0	0	0	0	0	0	0	0	0	0	0
PRKAG3	gene	0	0	0	0	0	0	0	0	0	0	0	0	0	0
PRKAG1	gene	0	0	0	0	0	0	0	0	0	0	0	0	0	0
PRKAA1	gene	0	0	0	0	0	0	0	0	0	0	0	0	0	0
PRKAA2	gene	0	0	0	0	0	0	0	0	0	0	0	0	0	0
PRKAB1	gene	0	0	0	0	0	0	0	0	0	0	0	0	0	0
PRKAG2	gene	0	0	0	0	0	0	0	0	0	0	0	0	0	0
DPP4	gene	0	0	0	0	0	0	0	0	0	0	0	0	0	0
F2	gene	0	0	0	0	0	0	0	0	0	0	0	0	0	0
POLB	gene	0	0	0	0	0	0	0	0	0	0	0	0	0	0
GSK3B	gene	0	0	0	0	0	0	0	0	0	0	0	0	0	0
PRKAB2	gene	0	0	0	0	0	0	0	0	0	0	0	0	0	0
PRKAG3	gene	0	0	0	0	0	0	0	0	0	0	0	0	0	0
PRKAG1	gene	0	0	0	0	0	0	0	0	0	0	0	0	0	0
PRKAA1	gene	0	0	0	0	0	0	0	0	0	0	0	0	0	0
PRKAA2	gene	0	0	0	0	0	0	0	0	0	0	0	0	0	0
PRKAB1	gene	0	0	0	0	0	0	0	0	0	0	0	0	0	0
PRKAG2	gene	0	0	0	0	0	0	0	0	0	0	0	0	0	0
DPP4	gene	0	0	0	0	0	0	0	0	0	0	0	0	0	0
F2	gene	0	0	0	0	0	0	0	0	0	0	0	0	0	0
POLB	gene	0	0	0	0	0	0	0	0	0	0	0	0	0	0
GSK3B	gene	0	0	0	0	0	0	0	0	0	0	0	0	0	0
PRKAB2	gene	0	0	0	0	0	0	0	0	0	0	0	0	0	0
PRKAG3	gene	0	0	0	0	0	0	0	0	0	0	0	0	0	0
PRKAG1	gene	0	0	0	0	0	0	0	0	0	0	0	0	0	0
PRKAA1	gene	0	0	0	0	0	0	0	0	0	0	0	0	0	0
PRKAA2	gene	0	0	0	0	0	0	0	0	0	0	0	0	0	0
PRKAB1	gene	0	0	0	0	0	0	0	0	0	0	0	0	0	0
PRKAG2	gene	0	0	0	0	0	0	0	0	0	0	0	0	0	0
DPP4	gene	0	0	0	0	0	0	0	0	0	0	0	0	0	0
F2	gene	0	0	0	0	0	0	0	0	0	0	0	0	0	0
POLB	gene	0	0	0	0	0	0	0	0	0	0	0	0	0	0
GSK3B	gene	0	0	0	0	0	0	0	0	0	0	0	0	0	0
PRKAB2	gene	0	0	0	0	0	0	0	0	0	0	0	0	0	0
PRKAG3	gene	0	0	0	0	0	0	0	0	0	0	0	0	0	0
PRKAG1	gene	0	0	0	0	0	0	0	0	0	0	0	0	0	0
PRKAA1	gene	0	0	0	0	0	0	0	0	0	0	0	0	0	0
PRKAA2	gene	0	0	0	0	0	0	0	0	0	0	0	0	0	0
PRKAB1	gene	0	0	0	0	0	0	0	0	0	0	0	0	0	0
PRKAG2	gene	0	0	0	0	0	0	0	0	0	0	0	0	0	0
DPP4	gene	0	0	0	0	0	0	0	0	0	0	0	0	0	0
F2	gene	0	0	0	0	0	0	0	0	0	0	0	0	0	0
POLB	gene	0	0	0	0	0	0	0	0	0	0	0	0	0	0
GSK3B	gene	0	0	0	0	0	0	0	0	0	0	0	0	0	0
PRKAB2	gene	0	0	0	0	0	0	0	0	0	0	0	0	0	0
PRKAG3	gene	0	0	0	0	0	0	0	0	0	0	0	0	0	0
PRKAG1	gene	0	0	0	0	0	0	0	0	0	0	0	0	0	0
PRKAA1	gene	0	0	0	0	0	0	0	0	0	0	0	0	0	0
PRKAA2	gene	0	0	0	0	0	0	0	0	0	0	0	0	0	0
PRKAB1	gene	0	0	0	0	0	0	0	0	0	0	0	0	0	0
PRKAG2	gene	0	0	0	0	0	0	0	0	0	0	0	0	0	0
DPP4	gene	0	0	0	0	0	0	0	0	0	0	0	0	0	0
F2	gene	0	0	0	0	0	0	0	0	0	0	0	0	0	0
POLB	gene	0	0	0	0	0	0	0	0	0	0	0	0	0	0
GSK3B	gene	0	0	0	0	0	0	0	0	0	0	0	0	0	0
PRKAB2	gene	0	0	0	0	0	0	0	0	0	0	0	0	0	0
PRKAG3	gene	0	0	0	0	0	0	0	0	0	0	0	0	0	0
PRKAG1	gene	0	0	0	0	0	0	0	0	0	0	0	0	0	0
PRKAA1	gene	0	0	0	0	0	0	0	0	0	0	0	0	0	0
PRKAA2	gene	0	0	0	0	0	0	0	0	0	0	0	0	0	0
PRKAB1	gene	0	0	0	0	0	0	0	0	0	0	0	0	0	0
PRKAG2	gene	0	0	0	0	0	0	0	0	0	0	0	0	0	0
DPP4	gene	0	0	0	0	0	0	0	0	0	0	0	0	0	0
F2	gene	0	0	0	0	0	0	0	0	0	0	0	0	0	0
FNTA	gene	0	0	0	0	0	0	0	0	0	0	0	0	0	0
GDI1	gene	0	0	0	0	0	0	0	0	0	0	0	0	0	0
FNTB	gene	0	0	0	0	0	0	0	0	0	0	0	0	0	0
ARHGDIA	gene	0	0	0	0	0	0	0	0	0	0	0	0	0	0
RAC1	gene	0	0	0	0	0	0	0	0	0	0	0	0	0	0
CDC42	gene	0	0	0	0	0	0	0	0	0	0	0	0	0	0
PGGT1B	gene	0	0	0	0	0	0	0	0	0	0	0	0	0	0
RABGGTB	gene	0	0	0	0	0	0	0	0	0	0	0	0	0	0
RAB7A	gene	0	0	0	0	0	0	0	0	0	0	0	0	0	0
RABGGTA	gene	0	0	0	0	0	0	0	0	0	0	0	0	0	0
PTPN1	gene	0	0	0	0	0	0	0	0	0	0	0	0	0	0
CDK1	gene	0	0	0	0	0	0	0	0	0	0	0	0	0	0
NR1I2	gene	0	0	0	0	0	0	0	0	0	0	0	0	0	0
UGT3A1	gene	0	0	0	0	0	0	0	0	0	0	0	0	0	0
CYP1B1	gene	0	0	0	0	0	0	0	0	0	0	0	0	0	0
RPS6KA3	gene	0	0	0	0	0	0	0	0	0	0	0	0	0	0
AHR	gene	0	0	0	0	0	0	0	0	0	0	0	0	0	0
UGT1A3	gene	0	0	0	0	0	0	0	0	0	0	0	0	0	0
UGT1A8	gene	0	0	0	0	0	0	0	0	0	0	0	0	0	0
UGT1A7	gene	0	0	0	0	0	0	0	0	0	0	0	0	0	0
UGT1A9	gene	0	0	0	0	0	0	0	0	0	0	0	0	0	0
CYP1A2	gene	0	0	0	0	0	0	0	0	0	0	0	0	0	0
CLK1	gene	0	0	0	0	0	0	0	0	0	0	0	0	0	0
STK16	gene	0	0	0	0	0	0	0	0	0	0	0	0	0	0
PIM2	gene	0	0	0	0	0	0	0	0	0	0	0	0	0	0
MAPT	gene	0	0	0	0	0	0	0	0	0	0	0	0	0	0
CYP2D6	gene	0	0	0	0	0	0	0	0	0	0	0	0	0	0
CYP3A4	gene	0	0	0	0	0	0	0	0	0	0	0	0	0	0
CYP2C9	gene	0	0	0	0	0	0	0	0	0	0	0	0	0	0
HPGD	gene	0	0	0	0	0	0	0	0	0	0	0	0	0	0
HIF1A	gene	0	0	0	0	0	0	0	0	0	0	0	0	0	0
CYP2C19	gene	0	0	0	0	0	0	0	0	0	0	0	0	0	0
GSK3A	gene	0	0	0	0	0	0	0	0	0	0	0	0	0	0
GSK3B	gene	0	0	0	0	0	0	0	0	0	0	0	0	0	0
CCNB2	gene	0	0	0	0	0	0	0	0	0	0	0	0	0	0
CCNB1	gene	0	0	0	0	0	0	0	0	0	0	0	0	0	0
CCNB3	gene	0	0	0	0	0	0	0	0	0	0	0	0	0	0
CDK6	gene	0	0	0	0	0	0	0	0	0	0	0	0	0	0
ALOX5	gene	0	0	0	0	0	0	0	0	0	0	0	0	0	0
GLO1	gene	0	0	0	0	0	0	0	0	0	0	0	0	0	0
XDH	gene	0	0	0	0	0	0	0	0	0	0	0	0	0	0
HSD17B1	gene	0	0	0	0	0	0	0	0	0	0	0	0	0	0
HSD17B2	gene	0	0	0	0	0	0	0	0	0	0	0	0	0	0
ABCC1	gene	0	0	0	0	0	0	0	0	0	0	0	0	0	0
AR	gene	0	0	0	0	0	0	0	0	0	0	0	0	0	0
BCHE	gene	0	0	0	0	0	0	0	0	0	0	0	0	0	0
PAFAH1B3	gene	0	0	0	0	0	0	0	0	0	0	0	0	0	0
CTDSP1	gene	0	0	0	0	0	0	0	0	0	0	0	0	0	0
CYP1A1	gene	0	0	0	0	0	0	0	0	0	0	0	0	0	0
NOX4	gene	0	0	0	0	0	0	0	0	0	0	0	0	0	0
ABCG2	gene	0	0	0	0	0	0	0	0	0	0	0	0	0	0
ESR1	gene	0	0	0	0	0	0	0	0	0	0	0	0	0	0
POLI	gene	0	0	0	0	0	0	0	0	0	0	0	0	0	0
POLH	gene	0	0	0	0	0	0	0	0	0	0	0	0	0	0
CHRM	gene	0	0	0	0	0	0	0	0	0	0	0	0	0	0
ABCB1	gene	0	0	0	0	0	0	0	0	0	0	0	0	0	0
RACGAP1	gene	0	0	0	0	0	0	0	0	0	0	0	0	0	0
AKR1B1	gene	0	0	0	0	0	0	0	0	0	0	0	0	0	0
NFE2L2	gene	0	0	0	0	0	0	0	0	0	0	0	0	0	0
PIP4K2A	gene	0	0	0	0	0	0	0	0	0	0	0	0	0	0
ADAM10	gene	0	0	0	0	0	0	0	0	0	0	0	0	0	0
ADAM17	gene	0	0	0	0	0	0	0	0	0	0	0	0	0	0
NR3C1	gene	0	0	0	0	0	0	0	0	0	0	0	0	0	0
FLT3	gene	0	0	0	0	0	0	0	0	0	0	0	0	0	0
PPARG	gene	0	0	0	0	0	0	0	0	0	0	0	0	0	0
CYP19A1	gene	0	0	0	0	0	0	0	0	0	0	0	0	0	0
ESRRA	gene	0	0	0	0	0	0	0	0	0	0	0	0	0	0
SIAE	gene	0	0	0	0	0	0	0	0	0	0	0	0	0	0
BACE1	gene	0	0	0	0	0	0	0	0	0	0	0	0	0	0
RARA	gene	0	0	0	0	0	0	0	0	0	0	0	0	0	0
NR1I3	gene	0	0	0	0	0	0	0	0	0	0	0	0	0	0
NFKB1	gene	0	0	0	0	0	0	0	0	0	0	0	0	0	0
NFKB2	gene	0	0	0	0	0	0	0	0	0	0	0	0	0	0
RELA	gene	0	0	0	0	0	0	0	0	0	0	0	0	0	0
CSNK2A2	gene	0	0	0	0	0	0	0	0	0	0	0	0	0	0
CSNK2B	gene	0	0	0	0	0	0	0	0	0	0	0	0	0	0
CSNK2A1	gene	0	0	0	0	0	0	0	0	0	0	0	0	0	0
DAPK1	gene	0	0	0	0	0	0	0	0	0	0	0	0	0	0
CA2	gene	0	0	0	0	0	0	0	0	0	0	0	0	0	0
CA7	gene	0	0	0	0	0	0	0	0	0	0	0	0	0	0
CA12	gene	0	0	0	0	0	0	0	0	0	0	0	0	0	0
HDAC9	gene	0	0	0	0	0	0	0	0	0	0	0	0	0	0
GLI3	gene	0	0	0	0	0	0	0	0	0	0	0	0	0	0
ESR2	gene	0	0	0	0	0	0	0	0	0	0	0	0	0	0
APP	gene	0	0	0	0	0	0	0	0	0	0	0	0	0	0
CISD1	gene	0	0	0	0	0	0	0	0	0	0	0	0	0	0
TYR	gene	0	0	0	0	0	0	0	0	0	0	0	0	0	0
PGR	gene	0	0	0	0	0	0	0	0	0	0	0	0	0	0
SMAD2	gene	0	0	0	0	0	0	0	0	0	0	0	0	0	0
SMAD3	gene	0	0	0	0	0	0	0	0	0	0	0	0	0	0
SLC2A1	gene	0	0	0	0	0	0	0	0	0	0	0	0	0	0
DPP4	gene	0	0	0	0	0	0	0	0	0	0	0	0	0	0
MPO	gene	0	0	0	0	0	0	0	0	0	0	0	0	0	0
ADORA1	gene	0	0	0	0	0	0	0	0	0	0	0	0	0	0
CA4	gene	0	0	0	0	0	0	0	0	0	0	0	0	0	0
CDK1	gene	0	0	0	0	0	0	0	0	0	0	0	0	0	0
NR1I2	gene	0	0	0	0	0	0	0	0	0	0	0	0	0	0
UGT3A1	gene	0	0	0	0	0	0	0	0	0	0	0	0	0	0
CYP1B1	gene	0	0	0	0	0	0	0	0	0	0	0	0	0	0
RPS6KA3	gene	0	0	0	0	0	0	0	0	0	0	0	0	0	0
AHR	gene	0	0	0	0	0	0	0	0	0	0	0	0	0	0
UGT1A3	gene	0	0	0	0	0	0	0	0	0	0	0	0	0	0
UGT1A8	gene	0	0	0	0	0	0	0	0	0	0	0	0	0	0
UGT1A7	gene	0	0	0	0	0	0	0	0	0	0	0	0	0	0
UGT1A9	gene	0	0	0	0	0	0	0	0	0	0	0	0	0	0
CYP1A2	gene	0	0	0	0	0	0	0	0	0	0	0	0	0	0
CLK1	gene	0	0	0	0	0	0	0	0	0	0	0	0	0	0
STK16	gene	0	0	0	0	0	0	0	0	0	0	0	0	0	0
PIM2	gene	0	0	0	0	0	0	0	0	0	0	0	0	0	0
MAPT	gene	0	0	0	0	0	0	0	0	0	0	0	0	0	0
CYP2D6	gene	0	0	0	0	0	0	0	0	0	0	0	0	0	0
CYP3A4	gene	0	0	0	0	0	0	0	0	0	0	0	0	0	0
CYP2C9	gene	0	0	0	0	0	0	0	0	0	0	0	0	0	0
HPGD	gene	0	0	0	0	0	0	0	0	0	0	0	0	0	0
HIF1A	gene	0	0	0	0	0	0	0	0	0	0	0	0	0	0
CYP2C19	gene	0	0	0	0	0	0	0	0	0	0	0	0	0	0
GSK3A	gene	0	0	0	0	0	0	0	0	0	0	0	0	0	0
GSK3B	gene	0	0	0	0	0	0	0	0	0	0	0	0	0	0
CCNB2	gene	0	0	0	0	0	0	0	0	0	0	0	0	0	0
CCNB1	gene	0	0	0	0	0	0	0	0	0	0	0	0	0	0
CCNB3	gene	0	0	0	0	0	0	0	0	0	0	0	0	0	0
CDK6	gene	0	0	0	0	0	0	0	0	0	0	0	0	0	0
ALOX5	gene	0	0	0	0	0	0	0	0	0	0	0	0	0	0
GLO1	gene	0	0	0	0	0	0	0	0	0	0	0	0	0	0
XDH	gene	0	0	0	0	0	0	0	0	0	0	0	0	0	0
HSD17B1	gene	0	0	0	0	0	0	0	0	0	0	0	0	0	0
HSD17B2	gene	0	0	0	0	0	0	0	0	0	0	0	0	0	0
ABCC1	gene	0	0	0	0	0	0	0	0	0	0	0	0	0	0
AR	gene	0	0	0	0	0	0	0	0	0	0	0	0	0	0
BCHE	gene	0	0	0	0	0	0	0	0	0	0	0	0	0	0
PAFAH1B3	gene	0	0	0	0	0	0	0	0	0	0	0	0	0	0
CTDSP1	gene	0	0	0	0	0	0	0	0	0	0	0	0	0	0
CYP1A1	gene	0	0	0	0	0	0	0	0	0	0	0	0	0	0
NOX4	gene	0	0	0	0	0	0	0	0	0	0	0	0	0	0
ABCG2	gene	0	0	0	0	0	0	0	0	0	0	0	0	0	0
ESR1	gene	0	0	0	0	0	0	0	0	0	0	0	0	0	0
POLI	gene	0	0	0	0	0	0	0	0	0	0	0	0	0	0
POLH	gene	0	0	0	0	0	0	0	0	0	0	0	0	0	0
CHRM	gene	0	0	0	0	0	0	0	0	0	0	0	0	0	0
ABCB1	gene	0	0	0	0	0	0	0	0	0	0	0	0	0	0
RACGAP1	gene	0	0	0	0	0	0	0	0	0	0	0	0	0	0
AKR1B1	gene	0	0	0	0	0	0	0	0	0	0	0	0	0	0
NFE2L2	gene	0	0	0	0	0	0	0	0	0	0	0	0	0	0
PIP4K2A	gene	0	0	0	0	0	0	0	0	0	0	0	0	0	0
ADAM10	gene	0	0	0	0	0	0	0	0	0	0	0	0	0	0
ADAM17	gene	0	0	0	0	0	0	0	0	0	0	0	0	0	0
NR3C1	gene	0	0	0	0	0	0	0	0	0	0	0	0	0	0
FLT3	gene	0	0	0	0	0	0	0	0	0	0	0	0	0	0
PPARG	gene	0	0	0	0	0	0	0	0	0	0	0	0	0	0
CYP19A1	gene	0	0	0	0	0	0	0	0	0	0	0	0	0	0
ESRRA	gene	0	0	0	0	0	0	0	0	0	0	0	0	0	0
SIAE	gene	0	0	0	0	0	0	0	0	0	0	0	0	0	0
BACE1	gene	0	0	0	0	0	0	0	0	0	0	0	0	0	0
RARA	gene	0	0	0	0	0	0	0	0	0	0	0	0	0	0
NR1I3	gene	0	0	0	0	0	0	0	0	0	0	0	0	0	0
NFKB1	gene	0	0	0	0	0	0	0	0	0	0	0	0	0	0
NFKB2	gene	0	0	0	0	0	0	0	0	0	0	0	0	0	0
RELA	gene	0	0	0	0	0	0	0	0	0	0	0	0	0	0
CSNK2A2	gene	0	0	0	0	0	0	0	0	0	0	0	0	0	0
CSNK2B	gene	0	0	0	0	0	0	0	0	0	0	0	0	0	0
CSNK2A1	gene	0	0	0	0	0	0	0	0	0	0	0	0	0	0
DAPK1	gene	0	0	0	0	0	0	0	0	0	0	0	0	0	0
CA2	gene	0	0	0	0	0	0	0	0	0	0	0	0	0	0
CA7	gene	0	0	0	0	0	0	0	0	0	0	0	0	0	0
CA12	gene	0	0	0	0	0	0	0	0	0	0	0	0	0	0
HDAC9	gene	0	0	0	0	0	0	0	0	0	0	0	0	0	0
GLI3	gene	0	0	0	0	0	0	0	0	0	0	0	0	0	0
ESR2	gene	0	0	0	0	0	0	0	0	0	0	0	0	0	0
APP	gene	0	0	0	0	0	0	0	0	0	0	0	0	0	0
CISD1	gene	0	0	0	0	0	0	0	0	0	0	0	0	0	0
TYR	gene	0	0	0	0	0	0	0	0	0	0	0	0	0	0
PGR	gene	0	0	0	0	0	0	0	0	0	0	0	0	0	0
SMAD2	gene	0	0	0	0	0	0	0	0	0	0	0	0	0	0
SMAD3	gene	0	0	0	0	0	0	0	0	0	0	0	0	0	0
SLC2A1	gene	0	0	0	0	0	0	0	0	0	0	0	0	0	0
DPP4	gene	0	0	0	0	0	0	0	0	0	0	0	0	0	0
MPO	gene	0	0	0	0	0	0	0	0	0	0	0	0	0	0
ADORA1	gene	0	0	0	0	0	0	0	0	0	0	0	0	0	0
CA4	gene	0	0	0	0	0	0	0	0	0	0	0	0	0	0
CDK1	gene	0	0	0	0	0	0	0	0	0	0	0	0	0	0
NR1I2	gene	0	0	0	0	0	0	0	0	0	0	0	0	0	0
UGT3A1	gene	0	0	0	0	0	0	0	0	0	0	0	0	0	0
CYP1B1	gene	0	0	0	0	0	0	0	0	0	0	0	0	0	0
RPS6KA3	gene	0	0	0	0	0	0	0	0	0	0	0	0	0	0
AHR	gene	0	0	0	0	0	0	0	0	0	0	0	0	0	0
UGT1A3	gene	0	0	0	0	0	0	0	0	0	0	0	0	0	0
UGT1A8	gene	0	0	0	0	0	0	0	0	0	0	0	0	0	0
UGT1A7	gene	0	0	0	0	0	0	0	0	0	0	0	0	0	0
UGT1A9	gene	0	0	0	0	0	0	0	0	0	0	0	0	0	0
CYP1A2	gene	0	0	0	0	0	0	0	0	0	0	0	0	0	0
CLK1	gene	0	0	0	0	0	0	0	0	0	0	0	0	0	0
STK16	gene	0	0	0	0	0	0	0	0	0	0	0	0	0	0
PIM2	gene	0	0	0	0	0	0	0	0	0	0	0	0	0	0
MAPT	gene	0	0	0	0	0	0	0	0	0	0	0	0	0	0
CYP2D6	gene	0	0	0	0	0	0	0	0	0	0	0	0	0	0
CYP3A4	gene	0	0	0	0	0	0	0	0	0	0	0	0	0	0
CYP2C9	gene	0	0	0	0	0	0	0	0	0	0	0	0	0	0
HPGD	gene	0	0	0	0	0	0	0	0	0	0	0	0	0	0
HIF1A	gene	0	0	0	0	0	0	0	0	0	0	0	0	0	0
CYP2C19	gene	0	0	0	0	0	0	0	0	0	0	0	0	0	0
GSK3A	gene	0	0	0	0	0	0	0	0	0	0	0	0	0	0
GSK3B	gene	0	0	0	0	0	0	0	0	0	0	0	0	0	0
CCNB2	gene	0	0	0	0	0	0	0	0	0	0	0	0	0	0
CCNB1	gene	0	0	0	0	0	0	0	0	0	0	0	0	0	0
CCNB3	gene	0	0	0	0	0	0	0	0	0	0	0	0	0	0
CDK6	gene	0	0	0	0	0	0	0	0	0	0	0	0	0	0
ALOX5	gene	0	0	0	0	0	0	0	0	0	0	0	0	0	0
GLO1	gene	0	0	0	0	0	0	0	0	0	0	0	0	0	0
XDH	gene	0	0	0	0	0	0	0	0	0	0	0	0	0	0
HSD17B1	gene	0	0	0	0	0	0	0	0	0	0	0	0	0	0
HSD17B2	gene	0	0	0	0	0	0	0	0	0	0	0	0	0	0
ABCC1	gene	0	0	0	0	0	0	0	0	0	0	0	0	0	0
AR	gene	0	0	0	0	0	0	0	0	0	0	0	0	0	0
BCHE	gene	0	0	0	0	0	0	0	0	0	0	0	0	0	0
PAFAH1B3	gene	0	0	0	0	0	0	0	0	0	0	0	0	0	0
CTDSP1	gene	0	0	0	0	0	0	0	0	0	0	0	0	0	0
CYP1A1	gene	0	0	0	0	0	0	0	0	0	0	0	0	0	0
NOX4	gene	0	0	0	0	0	0	0	0	0	0	0	0	0	0
ABCG2	gene	0	0	0	0	0	0	0	0	0	0	0	0	0	0
ESR1	gene	0	0	0	0	0	0	0	0	0	0	0	0	0	0
POLI	gene	0	0	0	0	0	0	0	0	0	0	0	0	0	0
POLH	gene	0	0	0	0	0	0	0	0	0	0	0	0	0	0
CHRM	gene	0	0	0	0	0	0	0	0	0	0	0	0	0	0
ABCB1	gene	0	0	0	0	0	0	0	0	0	0	0	0	0	0
RACGAP1	gene	0	0	0	0	0	0	0	0	0	0	0	0	0	0
AKR1B1	gene	0	0	0	0	0	0	0	0	0	0	0	0	0	0
NFE2L2	gene	0	0	0	0	0	0	0	0	0	0	0	0	0	0
PIP4K2A	gene	0	0	0	0	0	0	0	0	0	0	0	0	0	0
ADAM10	gene	0	0	0	0	0	0	0	0	0	0	0	0	0	0
ADAM17	gene	0	0	0	0	0	0	0	0	0	0	0	0	0	0
NR3C1	gene	0	0	0	0	0	0	0	0	0	0	0	0	0	0
FLT3	gene	0	0	0	0	0	0	0	0	0	0	0	0	0	0
PPARG	gene	0	0	0	0	0	0	0	0	0	0	0	0	0	0
CYP19A1	gene	0	0	0	0	0	0	0	0	0	0	0	0	0	0
ESRRA	gene	0	0	0	0	0	0	0	0	0	0	0	0	0	0
SIAE	gene	0	0	0	0	0	0	0	0	0	0	0	0	0	0
BACE1	gene	0	0	0	0	0	0	0	0	0	0	0	0	0	0
RARA	gene	0	0	0	0	0	0	0	0	0	0	0	0	0	0
NR1I3	gene	0	0	0	0	0	0	0	0	0	0	0	0	0	0
NFKB1	gene	0	0	0	0	0	0	0	0	0	0	0	0	0	0
NFKB2	gene	0	0	0	0	0	0	0	0	0	0	0	0	0	0
RELA	gene	0	0	0	0	0	0	0	0	0	0	0	0	0	0
CSNK2A2	gene	0	0	0	0	0	0	0	0	0	0	0	0	0	0
CSNK2B	gene	0	0	0	0	0	0	0	0	0	0	0	0	0	0
CSNK2A1	gene	0	0	0	0	0	0	0	0	0	0	0	0	0	0
DAPK1	gene	0	0	0	0	0	0	0	0	0	0	0	0	0	0
CA2	gene	0	0	0	0	0	0	0	0	0	0	0	0	0	0
CA7	gene	0	0	0	0	0	0	0	0	0	0	0	0	0	0
CA12	gene	0	0	0	0	0	0	0	0	0	0	0	0	0	0
HDAC9	gene	0	0	0	0	0	0	0	0	0	0	0	0	0	0
GLI3	gene	0	0	0	0	0	0	0	0	0	0	0	0	0	0
ESR2	gene	0	0	0	0	0	0	0	0	0	0	0	0	0	0
APP	gene	0	0	0	0	0	0	0	0	0	0	0	0	0	0
CISD1	gene	0	0	0	0	0	0	0	0	0	0	0	0	0	0
TYR	gene	0	0	0	0	0	0	0	0	0	0	0	0	0	0
PGR	gene	0	0	0	0	0	0	0	0	0	0	0	0	0	0
SMAD2	gene	0	0	0	0	0	0	0	0	0	0	0	0	0	0
SMAD3	gene	0	0	0	0	0	0	0	0	0	0	0	0	0	0
SLC2A1	gene	0	0	0	0	0	0	0	0	0	0	0	0	0	0
DPP4	gene	0	0	0	0	0	0	0	0	0	0	0	0	0	0
MPO	gene	0	0	0	0	0	0	0	0	0	0	0	0	0	0
ADORA1	gene	0	0	0	0	0	0	0	0	0	0	0	0	0	0
CA4	gene	0	0	0	0	0	0	0	0	0	0	0	0	0	0
MCL1	gene	0	0	0	0	0	0	0	0	0	0	0	0	0	0
HCK	gene	0	0	0	0	0	0	0	0	0	0	0	0	0	0
SLC2A2	gene	0	0	0	0	0	0	0	0	0	0	0	0	0	0
CYP2C8	gene	0	0	0	0	0	0	0	0	0	0	0	0	0	0
ATP5B	gene	0	0	0	0	0	0	0	0	0	0	0	0	0	0
HIBCH	gene	0	0	0	0	0	0	0	0	0	0	0	0	0	0
STK17B	gene	0	0	0	0	0	0	0	0	0	0	0	0	0	0
NOX4	gene	0	0	0	0	0	0	0	0	0	0	0	0	0	0
AVPR2	gene	0	0	0	0	0	0	0	0	0	0	0	0	0	0
AKR1B1	gene	0	0	0	0	0	0	0	0	0	0	0	0	0	0
XDH	gene	0	0	0	0	0	0	0	0	0	0	0	0	0	0
MAOA	gene	0	0	0	0	0	0	0	0	0	0	0	0	0	0
IGF1R	gene	0	0	0	0	0	0	0	0	0	0	0	0	0	0
FLT3	gene	0	0	0	0	0	0	0	0	0	0	0	0	0	0
CYP19A1	gene	0	0	0	0	0	0	0	0	0	0	0	0	0	0
EGFR	gene	0	0	0	0	0	0	0	0	0	0	0	0	0	0
F2	gene	0	0	0	0	0	0	0	0	0	0	0	0	0	0
CA2	gene	0	0	0	0	0	0	0	0	0	0	0	0	0	0
PIM1	gene	0	0	0	0	0	0	0	0	0	0	0	0	0	0
ALOX5	gene	0	0	0	0	0	0	0	0	0	0	0	0	0	0
AURKB	gene	0	0	0	0	0	0	0	0	0	0	0	0	0	0
DRD4	gene	0	0	0	0	0	0	0	0	0	0	0	0	0	0
ADORA1	gene	0	0	0	0	0	0	0	0	0	0	0	0	0	0
CA7	gene	0	0	0	0	0	0	0	0	0	0	0	0	0	0
GLO1	gene	0	0	0	0	0	0	0	0	0	0	0	0	0	0
MPO	gene	0	0	0	0	0	0	0	0	0	0	0	0	0	0
PIK3R1	gene	0	0	0	0	0	0	0	0	0	0	0	0	0	0
ADORA2A	gene	0	0	0	0	0	0	0	0	0	0	0	0	0	0
DAPK1	gene	0	0	0	0	0	0	0	0	0	0	0	0	0	0
PYGL	gene	0	0	0	0	0	0	0	0	0	0	0	0	0	0
CA1	gene	0	0	0	0	0	0	0	0	0	0	0	0	0	0
GSK3B	gene	0	0	0	0	0	0	0	0	0	0	0	0	0	0
SRC	gene	0	0	0	0	0	0	0	0	0	0	0	0	0	0
PTK2	gene	0	0	0	0	0	0	0	0	0	0	0	0	0	0
HSD17B2	gene	0	0	0	0	0	0	0	0	0	0	0	0	0	0
KDR	gene	0	0	0	0	0	0	0	0	0	0	0	0	0	0
MMP13	gene	0	0	0	0	0	0	0	0	0	0	0	0	0	0
MMP3	gene	0	0	0	0	0	0	0	0	0	0	0	0	0	0
CA3	gene	0	0	0	0	0	0	0	0	0	0	0	0	0	0
ALOX15	gene	0	0	0	0	0	0	0	0	0	0	0	0	0	0
ABCC1	gene	0	0	0	0	0	0	0	0	0	0	0	0	0	0
PLK1	gene	0	0	0	0	0	0	0	0	0	0	0	0	0	0
CA6	gene	0	0	0	0	0	0	0	0	0	0	0	0	0	0
CDK1	gene	0	0	0	0	0	0	0	0	0	0	0	0	0	0
MMP9	gene	0	0	0	0	0	0	0	0	0	0	0	0	0	0
CA12	gene	0	0	0	0	0	0	0	0	0	0	0	0	0	0
MMP2	gene	0	0	0	0	0	0	0	0	0	0	0	0	0	0
PKN1	gene	0	0	0	0	0	0	0	0	0	0	0	0	0	0
CA14	gene	0	0	0	0	0	0	0	0	0	0	0	0	0	0
CA9	gene	0	0	0	0	0	0	0	0	0	0	0	0	0	0
CSNK2A1	gene	0	0	0	0	0	0	0	0	0	0	0	0	0	0
ALOX12	gene	0	0	0	0	0	0	0	0	0	0	0	0	0	0
MET	gene	0	0	0	0	0	0	0	0	0	0	0	0	0	0
CA4	gene	0	0	0	0	0	0	0	0	0	0	0	0	0	0
NEK2	gene	0	0	0	0	0	0	0	0	0	0	0	0	0	0
CXCR1	gene	0	0	0	0	0	0	0	0	0	0	0	0	0	0
CAMK2B	gene	0	0	0	0	0	0	0	0	0	0	0	0	0	0
ALK	gene	0	0	0	0	0	0	0	0	0	0	0	0	0	0
AKT1	gene	0	0	0	0	0	0	0	0	0	0	0	0	0	0
ABCB1	gene	0	0	0	0	0	0	0	0	0	0	0	0	0	0
NEK6	gene	0	0	0	0	0	0	0	0	0	0	0	0	0	0
PLA2G1B	gene	0	0	0	0	0	0	0	0	0	0	0	0	0	0
CASA	gene	0	0	0	0	0	0	0	0	0	0	0	0	0	0
BACE1	gene	0	0	0	0	0	0	0	0	0	0	0	0	0	0
CYP1B1	gene	0	0	0	0	0	0	0	0	0	0	0	0	0	0
AXL	gene	0	0	0	0	0	0	0	0	0	0	0	0	0	0
ABCG2	gene	0	0	0	0	0	0	0	0	0	0	0	0	0	0
NUAK1	gene	0	0	0	0	0	0	0	0	0	0	0	0	0	0
AKR1C2	gene	0	0	0	0	0	0	0	0	0	0	0	0	0	0
AKR1C1	gene	0	0	0	0	0	0	0	0	0	0	0	0	0	0
AKR1C3	gene	0	0	0	0	0	0	0	0	0	0	0	0	0	0
AKR1C4	gene	0	0	0	0	0	0	0	0	0	0	0	0	0	0
CA13	gene	0	0	0	0	0	0	0	0	0	0	0	0	0	0
AKR1A1	gene	0	0	0	0	0	0	0	0	0	0	0	0	0	0
GPR35	gene	0	0	0	0	0	0	0	0	0	0	0	0	0	0
SYK	gene	0	0	0	0	0	0	0	0	0	0	0	0	0	0
MCL1	gene	0	0	0	0	0	0	0	0	0	0	0	0	0	0
HCK	gene	0	0	0	0	0	0	0	0	0	0	0	0	0	0
SLC2A2	gene	0	0	0	0	0	0	0	0	0	0	0	0	0	0
CYP2C8	gene	0	0	0	0	0	0	0	0	0	0	0	0	0	0
ATP5B	gene	0	0	0	0	0	0	0	0	0	0	0	0	0	0
HIBCH	gene	0	0	0	0	0	0	0	0	0	0	0	0	0	0
STK17B	gene	0	0	0	0	0	0	0	0	0	0	0	0	0	0
NOX4	gene	0	0	0	0	0	0	0	0	0	0	0	0	0	0
AVPR2	gene	0	0	0	0	0	0	0	0	0	0	0	0	0	0
AKR1B1	gene	0	0	0	0	0	0	0	0	0	0	0	0	0	0
XDH	gene	0	0	0	0	0	0	0	0	0	0	0	0	0	0
MAOA	gene	0	0	0	0	0	0	0	0	0	0	0	0	0	0
IGF1R	gene	0	0	0	0	0	0	0	0	0	0	0	0	0	0
FLT3	gene	0	0	0	0	0	0	0	0	0	0	0	0	0	0
CYP19A1	gene	0	0	0	0	0	0	0	0	0	0	0	0	0	0
EGFR	gene	0	0	0	0	0	0	0	0	0	0	0	0	0	0
F2	gene	0	0	0	0	0	0	0	0	0	0	0	0	0	0
CA2	gene	0	0	0	0	0	0	0	0	0	0	0	0	0	0
PIM1	gene	0	0	0	0	0	0	0	0	0	0	0	0	0	0
ALOX5	gene	0	0	0	0	0	0	0	0	0	0	0	0	0	0
AURKB	gene	0	0	0	0	0	0	0	0	0	0	0	0	0	0
DRD4	gene	0	0	0	0	0	0	0	0	0	0	0	0	0	0
ADORA1	gene	0	0	0	0	0	0	0	0	0	0	0	0	0	0
CA7	gene	0	0	0	0	0	0	0	0	0	0	0	0	0	0
GLO1	gene	0	0	0	0	0	0	0	0	0	0	0	0	0	0
MPO	gene	0	0	0	0	0	0	0	0	0	0	0	0	0	0
PIK3R1	gene	0	0	0	0	0	0	0	0	0	0	0	0	0	0
ADORA2A	gene	0	0	0	0	0	0	0	0	0	0	0	0	0	0
DAPK1	gene	0	0	0	0	0	0	0	0	0	0	0	0	0	0
PYGL	gene	0	0	0	0	0	0	0	0	0	0	0	0	0	0
CA1	gene	0	0	0	0	0	0	0	0	0	0	0	0	0	0
GSK3B	gene	0	0	0	0	0	0	0	0	0	0	0	0	0	0
SRC	gene	0	0	0	0	0	0	0	0	0	0	0	0	0	0
PTK2	gene	0	0	0	0	0	0	0	0	0	0	0	0	0	0
HSD17B2	gene	0	0	0	0	0	0	0	0	0	0	0	0	0	0
KDR	gene	0	0	0	0	0	0	0	0	0	0	0	0	0	0

MMP13	gene	0	0	0	0	0	0	0	0	0	0	0	0	0	0
MMP3	gene	0	0	0	0	0	0	0	0	0	0	0	0	0	0
CA3	gene	0	0	0	0	0	0	0	0	0	0	0	0	0	0
ALOX15	gene	0	0	0	0	0	0	0	0	0	0	0	0	0	0
ABCC1	gene	0	0	0	0	0	0	0	0	0	0	0	0	0	0
PLK1	gene	0	0	0	0	0	0	0	0	0	0	0	0	0	0
CA6	gene	0	0	0	0	0	0	0	0	0	0	0	0	0	0
CDK1	gene	0	0	0	0	0	0	0	0	0	0	0	0	0	0
MMP9	gene	0	0	0	0	0	0	0	0	0	0	0	0	0	0
CA12	gene	0	0	0	0	0	0	0	0	0	0	0	0	0	0
MMP2	gene	0	0	0	0	0	0	0	0	0	0	0	0	0	0
PKN1	gene	0	0	0	0	0	0	0	0	0	0	0	0	0	0
CA14	gene	0	0	0	0	0	0	0	0	0	0	0	0	0	0
CA9	gene	0	0	0	0	0	0	0	0	0	0	0	0	0	0
CSNK2A1	gene	0	0	0	0	0	0	0	0	0	0	0	0	0	0
ALOX12	gene	0	0	0	0	0	0	0	0	0	0	0	0	0	0
MET	gene	0	0	0	0	0	0	0	0	0	0	0	0	0	0
CA4	gene	0	0	0	0	0	0	0	0	0	0	0	0	0	0
NEK2	gene	0	0	0	0	0	0	0	0	0	0	0	0	0	0
CXCR1	gene	0	0	0	0	0	0	0	0	0	0	0	0	0	0
CAMK2B	gene	0	0	0	0	0	0	0	0	0	0	0	0	0	0
ALK	gene	0	0	0	0	0	0	0	0	0	0	0	0	0	0
AKT1	gene	0	0	0	0	0	0	0	0	0	0	0	0	0	0
ABCB1	gene	0	0	0	0	0	0	0	0	0	0	0	0	0	0
NEK6	gene	0	0	0	0	0	0	0	0	0	0	0	0	0	0
PLA2G1B	gene	0	0	0	0	0	0	0	0	0	0	0	0	0	0
CASA	gene	0	0	0	0	0	0	0	0	0	0	0	0	0	0
BACE1	gene	0	0	0	0	0	0	0	0	0	0	0	0	0	0
CYP1B1	gene	0	0	0	0	0	0	0	0	0	0	0	0	0	0
AXL	gene	0	0	0	0	0	0	0	0	0	0	0	0	0	0
ABCG2	gene	0	0	0	0	0	0	0	0	0	0	0	0	0	0
NUAK1	gene	0	0	0	0	0	0	0	0	0	0	0	0	0	0
AKR1C2	gene	0	0	0	0	0	0	0	0	0	0	0	0	0	0
AKR1C1	gene	0	0	0	0	0	0	0	0	0	0	0	0	0	0
AKR1C3	gene	0	0	0	0	0	0	0	0	0	0	0	0	0	0
AKR1C4	gene	0	0	0	0	0	0	0	0	0	0	0	0	0	0
CA13	gene	0	0	0	0	0	0	0	0	0	0	0	0	0	0
AKR1A1	gene	0	0	0	0	0	0	0	0	0	0	0	0	0	0
GPR35	gene	0	0	0	0	0	0	0	0	0	0	0	0	0	0
SYK	gene	0	0	0	0	0	0	0	0	0	0	0	0	0	0
MCL1	gene	0	0	0	0	0	0	0	0	0	0	0	0	0	0
HCK	gene	0	0	0	0	0	0	0	0	0	0	0	0	0	0
SLC2A2	gene	0	0	0	0	0	0	0	0	0	0	0	0	0	0
CYP2C8	gene	0	0	0	0	0	0	0	0	0	0	0	0	0	0
ATP5B	gene	0	0	0	0	0	0	0	0	0	0	0	0	0	0
HIBCH	gene	0	0	0	0	0	0	0	0	0	0	0	0	0	0
STK17B	gene	0	0	0	0	0	0	0	0	0	0	0	0	0	0
NOX4	gene	0	0	0	0	0	0	0	0	0	0	0	0	0	0
AVPR2	gene	0	0	0	0	0	0	0	0	0	0	0	0	0	0
AKR1B1	gene	0	0	0	0	0	0	0	0	0	0	0	0	0	0
XDH	gene	0	0	0	0	0	0	0	0	0	0	0	0	0	0
MAOA	gene	0	0	0	0	0	0	0	0	0	0	0	0	0	0
IGF1R	gene	0	0	0	0	0	0	0	0	0	0	0	0	0	0
FLT3	gene	0	0	0	0	0	0	0	0	0	0	0	0	0	0
CYP19A1	gene	0	0	0	0	0	0	0	0	0	0	0	0	0	0
EGFR	gene	0	0	0	0	0	0	0	0	0	0	0	0	0	0
F2	gene	0	0	0	0	0	0	0	0	0	0	0	0	0	0
CA2	gene	0	0	0	0	0	0	0	0	0	0	0	0	0	0
PIM1	gene	0	0	0	0	0	0	0	0	0	0	0	0	0	0
ALOX5	gene	0	0	0	0	0	0	0	0	0	0	0	0	0	0
AURKB	gene	0	0	0	0	0	0	0	0	0	0	0	0	0	0
DRD4	gene	0	0	0	0	0	0	0	0	0	0	0	0	0	0
ADORA1	gene	0	0	0	0	0	0	0	0	0	0	0	0	0	0
CA7	gene	0	0	0	0	0	0	0	0	0	0	0	0	0	0
GLO1	gene	0	0	0	0	0	0	0	0	0	0	0	0	0	0
MPO	gene	0	0	0	0	0	0	0	0	0	0	0	0	0	0
PIK3R1	gene	0	0	0	0	0	0	0	0	0	0	0	0	0	0
ADORA2A	gene	0	0	0	0	0	0	0	0	0	0	0	0	0	0
DAPK1	gene	0	0	0	0	0	0	0	0	0	0	0	0	0	0
PYGL	gene	0	0	0	0	0	0	0	0	0	0	0	0	0	0
CA1	gene	0	0	0	0	0	0	0	0	0	0	0	0	0	0
GSK3B	gene	0	0	0	0	0	0	0	0	0	0	0	0	0	0
SRC	gene	0	0	0	0	0	0	0	0	0	0	0	0	0	0
PTK2	gene	0	0	0	0	0	0	0	0	0	0	0	0	0	0
HSD17B2	gene	0	0	0	0	0	0	0	0	0	0	0	0	0	0
KDR	gene	0	0	0	0	0	0	0	0	0	0	0	0	0	0
MMP13	gene	0	0	0	0	0	0	0	0	0	0	0	0	0	0
MMP3	gene	0	0	0	0	0	0	0	0	0	0	0	0	0	0
CA3	gene	0	0	0	0	0	0	0	0	0	0	0	0	0	0
ALOX15	gene	0	0	0	0	0	0	0	0	0	0	0	0	0	0
ABCC1	gene	0	0	0	0	0	0	0	0	0	0	0	0	0	0
PLK1	gene	0	0	0	0	0	0	0	0	0	0	0	0	0	0
CA6	gene	0	0	0	0	0	0	0	0	0	0	0	0	0	0
CDK1	gene	0	0	0	0	0	0	0	0	0	0	0	0	0	0
MMP9	gene	0	0	0	0	0	0	0	0	0	0	0	0	0	0
CA12	gene	0	0	0	0	0	0	0	0	0	0	0	0	0	0
MMP2	gene	0	0	0	0	0	0	0	0	0	0	0	0	0	0
PKN1	gene	0	0	0	0	0	0	0	0	0	0	0	0	0	0
CA14	gene	0	0	0	0	0	0	0	0	0	0	0	0	0	0
CA9	gene	0	0	0	0	0	0	0	0	0	0	0	0	0	0
CSNK2A1	gene	0	0	0	0	0	0	0	0	0	0	0	0	0	0
ALOX12	gene	0	0	0	0	0	0	0	0	0	0	0	0	0	0
MET	gene	0	0	0	0	0	0	0	0	0	0	0	0	0	0
CA4	gene	0	0	0	0	0	0	0	0	0	0	0	0	0	0
NEK2	gene	0	0	0	0	0	0	0	0	0	0	0	0	0	0
CXCR1	gene	0	0	0	0	0	0	0	0	0	0	0	0	0	0
CAMK2B	gene	0	0	0	0	0	0	0	0	0	0	0	0	0	0
ALK	gene	0	0	0	0	0	0	0	0	0	0	0	0	0	0
AKT1	gene	0	0	0	0	0	0	0	0	0	0	0	0	0	0
ABCB1	gene	0	0	0	0	0	0	0	0	0	0	0	0	0	0
NEK6	gene	0	0	0	0	0	0	0	0	0	0	0	0	0	0
PLA2G1B	gene	0	0	0	0	0	0	0	0	0	0	0	0	0	0
CASA	gene	0	0	0	0	0	0	0	0	0	0	0	0	0	0
BACE1	gene	0	0	0	0	0	0	0	0	0	0	0	0	0	0
CYP1B1	gene	0	0	0	0	0	0	0	0	0	0	0	0	0	0
AXL	gene	0	0	0	0	0	0	0	0	0	0	0	0	0	0
ABCG2	gene	0	0	0	0	0	0	0	0	0	0	0	0	0	0
NUAK1	gene	0	0	0	0	0	0	0	0	0	0	0	0	0	0
AKR1C2	gene	0	0	0	0	0	0	0	0	0	0	0	0	0	0
AKR1C1	gene	0	0	0	0	0	0	0	0	0	0	0	0	0	0
AKR1C3	gene	0	0	0	0	0	0	0	0	0	0	0	0	0	0
AKR1C4	gene	0	0	0	0	0	0	0	0	0	0	0	0	0	0
CA13	gene	0	0	0	0	0	0	0	0	0	0	0	0	0	0
AKR1A1	gene	0	0	0	0	0	0	0	0	0	0	0	0	0	0
GPR35	gene	0	0	0	0	0	0	0	0	0	0	0	0	0	0
SYK	gene	0	0	0	0	0	0	0	0	0	0	0	0	0	0
MCL1	gene	0	0	0	0	0	0	0	0	0	0	0	0	0	0
HCK	gene	0	0	0	0	0	0	0	0	0	0	0	0	0	0
SLC2A2	gene	0	0	0	0	0	0	0	0	0	0	0	0	0	0
CYP2C8	gene	0	0	0	0	0	0	0	0	0	0	0	0	0	0
ATP5B	gene	0	0	0	0	0	0	0	0	0	0	0	0	0	0
HIBCH	gene	0	0	0	0	0	0	0	0	0	0	0	0	0	0
STK17B	gene	0	0	0	0	0	0	0	0	0	0	0	0	0	0
NOX4	gene	0	0	0	0	0	0	0	0	0	0	0	0	0	0
AVPR2	gene	0	0	0	0	0	0	0	0	0	0	0	0	0	0
AKR1B1	gene	0	0	0	0	0	0	0	0	0	0	0	0	0	0
XDH	gene	0	0	0	0	0	0	0	0	0	0	0	0	0	0
MAOA	gene	0	0	0	0	0	0	0	0	0	0	0	0	0	0
IGF1R	gene	0	0	0	0	0	0	0	0	0	0	0	0	0	0
FLT3	gene	0	0	0	0	0	0	0	0	0	0	0	0	0	0
CYP19A1	gene	0	0	0	0	0	0	0	0	0	0	0	0	0	0
EGFR	gene	0	0	0	0	0	0	0	0	0	0	0	0	0	0
F2	gene	0	0	0	0	0	0	0	0	0	0	0	0	0	0
CA2	gene	0	0	0	0	0	0	0	0	0	0	0	0	0	0
PIM1	gene	0	0	0	0	0	0	0	0	0	0	0	0	0	0
ALOX5	gene	0	0	0	0	0	0	0	0	0	0	0	0	0	0
AURKB	gene	0	0	0	0	0	0	0	0	0	0	0	0	0	0
DRD4	gene	0	0	0	0	0	0	0	0	0	0	0	0	0	0
ADORA1	gene	0	0	0	0	0	0	0	0	0	0	0	0	0	0
CA7	gene	0	0	0	0	0	0	0	0	0	0	0	0	0	0
GLO1	gene	0	0	0	0	0	0	0	0	0	0	0	0	0	0
MPO	gene	0	0	0	0	0	0	0	0	0	0	0	0	0	0
PIK3R1	gene	0	0	0	0	0	0	0	0	0	0	0	0	0	0
ADORA2A	gene	0	0	0	0	0	0	0	0	0	0	0	0	0	0
DAPK1	gene	0	0	0	0	0	0	0	0	0	0	0	0	0	0
PYGL	gene	0	0	0	0	0	0	0	0	0	0	0	0	0	0
CA1	gene	0	0	0	0	0	0	0	0	0	0	0	0	0	0
GSK3B	gene	0	0	0	0	0	0	0	0	0	0	0	0	0	0
SRC	gene	0	0	0	0	0	0	0	0	0	0	0	0	0	0
PTK2	gene	0	0	0	0	0	0	0	0	0	0	0	0	0	0
HSD17B2	gene	0	0	0	0	0	0	0	0	0	0	0	0	0	0
KDR	gene	0	0	0	0	0	0	0	0	0	0	0	0	0	0
MMP13	gene	0	0	0	0	0	0	0	0	0	0	0	0	0	0
MMP3	gene	0	0	0	0	0	0	0	0	0	0	0	0	0	0
CA3	gene	0	0	0	0	0	0	0	0	0	0	0	0	0	0
ALOX15	gene	0	0	0	0	0	0	0	0	0	0	0	0	0	0
ABCC1	gene	0	0	0	0	0	0	0	0	0	0	0	0	0	0
PLK1	gene	0	0	0	0	0	0	0	0	0	0	0	0	0	0
CA6	gene	0	0	0	0	0	0	0	0	0	0	0	0	0	0
CDK1	gene	0	0	0	0	0	0	0	0	0	0	0	0	0	0
MMP9	gene	0	0	0	0	0	0	0	0	0	0	0	0	0	0
CA12	gene	0	0	0	0	0	0	0	0	0	0	0	0	0	0
MMP2	gene	0	0	0	0	0	0	0	0	0	0	0	0	0	0
PKN1	gene	0	0	0	0	0	0	0	0	0	0	0	0	0	0
CA14	gene	0	0	0	0	0	0	0	0	0	0	0	0	0	0
CA9	gene	0	0	0	0	0	0	0	0	0	0	0	0	0	0
CSNK2A1	gene	0	0	0	0	0	0	0	0	0	0	0	0	0	0
ALOX12	gene	0	0	0	0	0	0	0	0	0	0	0	0	0	0
MET	gene	0	0	0	0	0	0	0	0	0	0	0	0	0	0
CA4	gene	0	0	0	0	0	0	0	0	0	0	0	0	0	0
NEK2	gene	0	0	0	0	0	0	0	0	0	0	0	0	0	0
CXCR1	gene	0	0	0	0	0	0	0	0	0	0	0	0	0	0
CAMK2B	gene	0	0	0	0	0	0	0	0	0	0	0	0	0	0
ALK	gene	0	0	0	0	0	0	0	0	0	0	0	0	0	0
AKT1	gene	0	0	0	0	0	0	0	0	0	0	0	0	0	0
ABCB1	gene	0	0	0	0	0	0	0	0	0	0	0	0	0	0
NEK6	gene	0	0	0	0	0	0	0	0	0	0	0	0	0	0
PLA2G1B	gene	0	0	0	0	0	0	0	0	0	0	0	0	0	0
CASA	gene	0	0	0	0	0	0	0	0	0	0	0	0	0	0
BACE1	gene	0	0	0	0	0	0	0	0	0	0	0	0	0	0
CYP1B1	gene	0	0	0	0	0	0	0	0	0	0	0	0	0	0
AXL	gene	0	0	0	0	0	0	0	0	0	0	0	0	0	0
ABCG2	gene	0	0	0	0	0	0	0	0	0	0	0	0	0	0
NUAK1	gene	0	0	0	0	0	0	0	0	0	0	0	0	0	0
AKR1C2	gene	0	0	0	0	0	0	0	0	0	0	0	0	0	0
AKR1C1	gene	0	0	0	0	0	0	0	0	0	0	0	0	0	0
AKR1C3	gene	0	0	0	0	0	0	0	0	0	0	0	0	0	0
AKR1C4	gene	0	0	0	0	0	0	0	0	0	0	0	0	0	0
CA13	gene	0	0	0	0	0	0	0	0	0	0	0	0	0	0
AKR1A1	gene	0	0	0	0	0	0	0	0	0	0	0	0	0	0
GPR35	gene	0	0	0	0	0	0	0	0	0	0	0	0	0	0
SYK	gene	0	0	0	0	0	0	0	0	0	0	0	0	0	0
MCL1	gene	0	0	0	0	0	0	0	0	0	0	0	0	0	0
HCK	gene	0	0	0	0	0	0	0	0	0	0	0	0	0	0
SLC2A2	gene	0	0	0	0	0	0	0	0	0	0	0	0	0	0
CYP2C8	gene	0	0	0	0	0	0	0	0	0	0	0	0	0	0
ATP5B	gene	0	0	0	0	0	0	0	0	0	0	0	0	0	0
HIBCH	gene	0	0	0	0	0	0	0	0	0	0	0	0	0	0
STK17B	gene	0	0	0	0	0	0	0	0	0	0	0	0	0	0
NOX4	gene	0	0	0	0	0	0	0	0	0	0	0	0	0	0
AVPR2	gene	0	0	0	0	0	0	0	0	0	0	0	0	0	0
AKR1B1	gene	0	0	0	0	0	0	0	0	0	0	0	0	0	0
XDH	gene	0	0	0	0	0	0	0	0	0	0	0	0	0	0
MAOA	gene	0	0	0	0	0	0	0	0	0	0	0	0	0	0
IGF1R	gene	0	0	0	0	0	0	0	0	0	0	0	0	0	0
FLT3	gene	0	0	0	0	0	0	0	0	0	0	0	0	0	0
CYP19A1	gene	0	0	0	0	0	0	0	0	0	0	0	0	0	0
EGFR	gene	0	0	0	0	0	0	0	0	0	0	0	0	0	0
F2	gene	0	0	0	0	0	0	0	0	0	0	0	0	0	0
CA2	gene	0	0	0	0	0	0	0	0	0	0	0	0	0	0
PIM1	gene	0	0	0	0	0	0	0	0	0	0	0	0	0	0
ALOX5	gene	0	0	0	0	0	0	0	0	0	0	0	0	0	0
AURKB	gene	0	0	0	0	0	0	0	0	0	0	0	0	0	0
DRD4	gene	0	0	0	0	0	0	0	0	0	0	0	0	0	0
ADORA1	gene	0	0	0	0	0	0	0	0	0	0	0	0	0	0
CA7	gene	0	0	0	0	0	0	0	0	0	0	0	0	0	0
GLO1	gene	0	0	0	0	0	0	0	0	0	0	0	0	0	0
MPO	gene	0	0	0	0	0	0	0	0	0	0	0	0	0	0
PIK3R1	gene	0	0	0	0	0	0	0	0	0	0	0	0	0	0
ADORA2A	gene	0	0	0	0	0	0	0	0	0	0	0	0	0	0
DAPK1	gene	0	0	0	0	0	0	0	0	0	0	0	0	0	0
PYGL	gene	0	0	0	0	0	0	0	0	0	0	0	0	0	0
CA1	gene	0	0	0	0	0	0	0	0	0	0	0	0	0	0
GSK3B	gene	0	0	0	0	0	0	0	0	0	0	0	0	0	0
SRC	gene	0	0	0	0	0	0	0	0	0	0	0	0	0	0
PTK2	gene	0	0	0	0	0	0	0	0	0	0	0	0	0	0
HSD17B2	gene	0	0	0	0	0	0	0	0	0	0	0	0	0	0
KDR	gene	0	0	0	0	0	0	0	0	0	0	0	0	0	0
MMP13	gene	0	0	0	0	0	0	0	0	0	0	0	0	0	0
MMP3	gene	0	0	0	0	0	0	0	0	0	0	0	0	0	0
CA3	gene	0	0	0	0	0	0	0	0	0	0	0	0	0	0
ALOX15	gene	0	0	0	0	0	0	0	0	0	0	0	0	0	0
ABCC1	gene	0	0	0	0	0	0	0	0	0	0	0	0	0	0
PLK1	gene	0	0	0	0	0	0	0	0	0	0	0	0	0	0
CA6	gene	0	0	0	0	0	0	0	0	0	0	0	0	0	0
CDK1	gene	0	0	0	0	0	0	0	0	0	0	0	0	0	0
MMP9	gene	0	0	0	0	0	0	0	0	0	0	0	0	0	0
CA12	gene	0	0	0	0	0	0	0	0	0	0	0	0	0	0
MMP2	gene	0	0	0	0	0	0	0	0	0	0	0	0	0	0
PKN1	gene	0	0	0	0	0	0	0	0	0	0	0	0	0	0
CA14	gene	0	0	0	0	0	0	0	0	0	0	0	0	0	0
CA9	gene	0	0	0	0	0	0	0	0	0	0	0	0	0	0
CSNK2A1	gene	0	0	0	0	0	0	0	0	0	0	0	0	0	0
ALOX12	gene	0	0	0	0	0	0	0	0	0	0	0	0	0	0
MET	gene	0	0	0	0	0	0	0	0	0	0	0	0	0	0
CA4	gene	0	0	0	0	0	0	0	0	0	0	0	0	0	0
NEK2	gene	0	0	0	0	0	0	0	0	0	0	0	0	0	0
CXCR1	gene	0	0	0	0	0	0	0	0	0	0	0	0	0	0
CAMK2B	gene	0	0	0	0	0	0	0	0	0	0	0	0	0	0
ALK	gene	0	0	0	0	0	0	0	0	0	0	0	0	0	0
AKT1	gene	0	0	0	0	0	0	0	0	0	0	0	0	0	0
ABCB1	gene	0	0	0	0	0	0	0	0	0	0	0	0	0	0
NEK6	gene	0	0	0	0	0	0	0	0	0	0	0	0	0	0
PLA2G1B	gene	0	0	0	0	0	0	0	0	0	0	0	0	0	0
CASA	gene	0	0	0	0	0	0	0	0	0	0	0	0	0	0
BACE1	gene	0	0	0	0	0	0	0	0	0	0	0	0	0	0
CYP1B1	gene	0	0	0	0	0	0	0	0	0	0	0	0	0	0
AXL	gene	0	0	0	0	0	0	0	0	0	0	0	0	0	0
ABCG2	gene	0	0	0	0	0	0	0	0	0	0	0	0	0	0
NUAK1	gene	0	0	0	0	0	0	0	0	0	0	0	0	0	0
AKR1C2	gene	0	0	0	0	0	0	0	0	0	0	0	0	0	0
AKR1C1	gene	0	0	0	0	0	0	0	0	0	0	0	0	0	0
AKR1C3	gene	0	0	0	0	0	0	0	0	0	0	0	0	0	0
AKR1C4	gene	0	0	0	0	0	0	0	0	0	0	0	0	0	0
CA13	gene	0	0	0	0	0	0	0	0	0	0	0	0	0	0
AKR1A1	gene	0	0	0	0	0	0	0	0	0	0	0	0	0	0
GPR35	gene	0	0	0	0	0	0	0	0	0	0	0	0	0	0
SYK	gene	0	0	0	0	0	0	0	0	0	0	0	0	0	0
MAPK8	gene	0	0	0	0	0	0	0	0	0	0	0	0	0	0
MMP9	gene	0	0	0	0	0	0	0	0	0	0	0	0	0	0
CASP3	gene	0	0	0	0	0	0	0	0	0	0	0	0	0	0
JUN	gene	0	0	0	0	0	0	0	0	0	0	0	0	0	0
FOS	gene	0	0	0	0	0	0	0	0	0	0	0	0	0	0
CDK2	gene	0	0	0	0	0	0	0	0	0	0	0	0	0	0
EGFR	gene	0	0	0	0	0	0	0	0	0	0	0	0	0	0
SMAD2	gene	0	0	0	0	0	0	0	0	0	0	0	0	0	0
CCNA2	gene	0	0	0	0	0	0	0	0	0	0	0	0	0	0
AKT1	gene	0	0	0	0	0	0	0	0	0	0	0	0	0	0
HPGD	gene	0	0	0	0	0	0	0	0	0	0	0	0	0	0
CYP1A2	gene	0	0	0	0	0	0	0	0	0	0	0	0	0	0
CSNK1G2	gene	0	0	0	0	0	0	0	0	0	0	0	0	0	0
CLK3	gene	0	0	0	0	0	0	0	0	0	0	0	0	0	0
CLK1	gene	0	0	0	0	0	0	0	0	0	0	0	0	0	0
STK16	gene	0	0	0	0	0	0	0	0	0	0	0	0	0	0
PIM2	gene	0	0	0	0	0	0	0	0	0	0	0	0	0	0
CBR3	gene	0	0	0	0	0	0	0	0	0	0	0	0	0	0
ALOX15B	gene	0	0	0	0	0	0	0	0	0	0	0	0	0	0
CYP3A4	gene	0	0	0	0	0	0	0	0	0	0	0	0	0	0
ALOX15	gene	0	0	0	0	0	0	0	0	0	0	0	0	0	0
MCL1	gene	0	0	0	0	0	0	0	0	0	0	0	0	0	0
ALDH1A1	gene	0	0	0	0	0	0	0	0	0	0	0	0	0	0
SMN2	gene	0	0	0	0	0	0	0	0	0	0	0	0	0	0
MAPT	gene	0	0	0	0	0	0	0	0	0	0	0	0	0	0
CYP2D6	gene	0	0	0	0	0	0	0	0	0	0	0	0	0	0
CYP2C19	gene	0	0	0	0	0	0	0	0	0	0	0	0	0	0
CYP2C9	gene	0	0	0	0	0	0	0	0	0	0	0	0	0	0
GSTO1	gene	0	0	0	0	0	0	0	0	0	0	0	0	0	0
GAA	gene	0	0	0	0	0	0	0	0	0	0	0	0	0	0
BLM	gene	0	0	0	0	0	0	0	0	0	0	0	0	0	0
ALPI	gene	0	0	0	0	0	0	0	0	0	0	0	0	0	0
TYR	gene	0	0	0	0	0	0	0	0	0	0	0	0	0	0
GSK3A	gene	0	0	0	0	0	0	0	0	0	0	0	0	0	0
GSK3B	gene	0	0	0	0	0	0	0	0	0	0	0	0	0	0
CCNB2	gene	0	0	0	0	0	0	0	0	0	0	0	0	0	0
CDK1	gene	0	0	0	0	0	0	0	0	0	0	0	0	0	0
CCNB1	gene	0	0	0	0	0	0	0	0	0	0	0	0	0	0
CCNB3	gene	0	0	0	0	0	0	0	0	0	0	0	0	0	0
CDK5	gene	0	0	0	0	0	0	0	0	0	0	0	0	0	0
CDK5R1	gene	0	0	0	0	0	0	0	0	0	0	0	0	0	0
GLO1	gene	0	0	0	0	0	0	0	0	0	0	0	0	0	0
XDH	gene	0	0	0	0	0	0	0	0	0	0	0	0	0	0
NR3C1	gene	0	0	0	0	0	0	0	0	0	0	0	0	0	0
AR	gene	0	0	0	0	0	0	0	0	0	0	0	0	0	0
MAOA	gene	0	0	0	0	0	0	0	0	0	0	0	0	0	0
POLB	gene	0	0	0	0	0	0	0	0	0	0	0	0	0	0
PAFAH1B2	gene	0	0	0	0	0	0	0	0	0	0	0	0	0	0
PAFAH1B3	gene	0	0	0	0	0	0	0	0	0	0	0	0	0	0
CYP1A1	gene	0	0	0	0	0	0	0	0	0	0	0	0	0	0
CYP1B1	gene	0	0	0	0	0	0	0	0	0	0	0	0	0	0
BAZ2B	gene	0	0	0	0	0	0	0	0	0	0	0	0	0	0
NOX4	gene	0	0	0	0	0	0	0	0	0	0	0	0	0	0
FXN	gene	0	0	0	0	0	0	0	0	0	0	0	0	0	0
TNFRSF10B	gene	0	0	0	0	0	0	0	0	0	0	0	0	0	0
ABCG2	gene	0	0	0	0	0	0	0	0	0	0	0	0	0	0
POLK	gene	0	0	0	0	0	0	0	0	0	0	0	0	0	0
POLI	gene	0	0	0	0	0	0	0	0	0	0	0	0	0	0
POLH	gene	0	0	0	0	0	0	0	0	0	0	0	0	0	0
FEN1	gene	0	0	0	0	0	0	0	0	0	0	0	0	0	0
CD38	gene	0	0	0	0	0	0	0	0	0	0	0	0	0	0
WRN	gene	0	0	0	0	0	0	0	0	0	0	0	0	0	0
TRA	gene	0	0	0	0	0	0	0	0	0	0	0	0	0	0
DRD3	gene	0	0	0	0	0	0	0	0	0	0	0	0	0	0
PIP4K2A	gene	0	0	0	0	0	0	0	0	0	0	0	0	0	0
MMP2	gene	0	0	0	0	0	0	0	0	0	0	0	0	0	0
MMP3	gene	0	0	0	0	0	0	0	0	0	0	0	0	0	0
MMP12	gene	0	0	0	0	0	0	0	0	0	0	0	0	0	0
MMP13	gene	0	0	0	0	0	0	0	0	0	0	0	0	0	0
GPR35	gene	0	0	0	0	0	0	0	0	0	0	0	0	0	0
ABCB1	gene	0	0	0	0	0	0	0	0	0	0	0	0	0	0
SYK	gene	0	0	0	0	0	0	0	0	0	0	0	0	0	0
CFTR	gene	0	0	0	0	0	0	0	0	0	0	0	0	0	0
RAPGEF3	gene	0	0	0	0	0	0	0	0	0	0	0	0	0	0
PARP1	gene	0	0	0	0	0	0	0	0	0	0	0	0	0	0
FLT3	gene	0	0	0	0	0	0	0	0	0	0	0	0	0	0
TNKS	gene	0	0	0	0	0	0	0	0	0	0	0	0	0	0
TNKS2	gene	0	0	0	0	0	0	0	0	0	0	0	0	0	0
PSIP1	gene	0	0	0	0	0	0	0	0	0	0	0	0	0	0
CYBB	gene	0	0	0	0	0	0	0	0	0	0	0	0	0	0
AKR1B1	gene	0	0	0	0	0	0	0	0	0	0	0	0	0	0
ALOX5	gene	0	0	0	0	0	0	0	0	0	0	0	0	0	0
APP	gene	0	0	0	0	0	0	0	0	0	0	0	0	0	0
NFKB1	gene	0	0	0	0	0	0	0	0	0	0	0	0	0	0
NFKB2	gene	0	0	0	0	0	0	0	0	0	0	0	0	0	0
RELA	gene	0	0	0	0	0	0	0	0	0	0	0	0	0	0
CSNK2A2	gene	0	0	0	0	0	0	0	0	0	0	0	0	0	0
CSNK2B	gene	0	0	0	0	0	0	0	0	0	0	0	0	0	0
CSNK2A1	gene	0	0	0	0	0	0	0	0	0	0	0	0	0	0
DAPK1	gene	0	0	0	0	0	0	0	0	0	0	0	0	0	0
TTR	gene	0	0	0	0	0	0	0	0	0	0	0	0	0	0
CA2	gene	0	0	0	0	0	0	0	0	0	0	0	0	0	0
CA7	gene	0	0	0	0	0	0	0	0	0	0	0	0	0	0
CA12	gene	0	0	0	0	0	0	0	0	0	0	0	0	0	0
CA4	gene	0	0	0	0	0	0	0	0	0	0	0	0	0	0
AKR1B10	gene	0	0	0	0	0	0	0	0	0	0	0	0	0	0
PRBM1	gene	0	0	0	0	0	0	0	0	0	0	0	0	0	0
DPP4	gene	0	0	0	0	0	0	0	0	0	0	0	0	0	0
IFNB1	gene	0	0	0	0	0	0	0	0	0	0	0	0	0	0
TFE3	gene	0	0	0	0	0	0	0	0	0	0	0	0	0	0
CDK5R1	gene	0	0	0	0	0	0	0	0	0	0	0	0	0	0
ADORA1	gene	0	0	0	0	0	0	0	0	0	0	0	0	0	0
TOP1	gene	0	0	0	0	0	0	0	0	0	0	0	0	0	0
CYP3A4	gene	0	0	0	0	0	0	0	0	0	0	0	0	0	0
CYP1A2	gene	0	0	0	0	0	0	0	0	0	0	0	0	0	0
CYP2C19	gene	0	0	0	0	0	0	0	0	0	0	0	0	0	0
CYP2C9	gene	0	0	0	0	0	0	0	0	0	0	0	0	0	0
CYP2D6	gene	0	0	0	0	0	0	0	0	0	0	0	0	0	0
TARDBP	gene	0	0	0	0	0	0	0	0	0	0	0	0	0	0
PDE5A	gene	0	0	0	0	0	0	0	0	0	0	0	0	0	0
UBA2	gene	0	0	0	0	0	0	0	0	0	0	0	0	0	0
SAE1	gene	0	0	0	0	0	0	0	0	0	0	0	0	0	0
UBE2I	gene	0	0	0	0	0	0	0	0	0	0	0	0	0	0
STK33	gene	0	0	0	0	0	0	0	0	0	0	0	0	0	0
POLB	gene	0	0	0	0	0	0	0	0	0	0	0	0	0	0
NR1H3	gene	0	0	0	0	0	0	0	0	0	0	0	0	0	0
GSK3B	gene	0	0	0	0	0	0	0	0	0	0	0	0	0	0
GPBAR1	gene	0	0	0	0	0	0	0	0	0	0	0	0	0	0
PRKAB2	gene	0	0	0	0	0	0	0	0	0	0	0	0	0	0
PRKAG3	gene	0	0	0	0	0	0	0	0	0	0	0	0	0	0
PRKAG1	gene	0	0	0	0	0	0	0	0	0	0	0	0	0	0
PRKAA1	gene	0	0	0	0	0	0	0	0	0	0	0	0	0	0
PRKAA2	gene	0	0	0	0	0	0	0	0	0	0	0	0	0	0
PRKAB1	gene	0	0	0	0	0	0	0	0	0	0	0	0	0	0
PRKAG2	gene	0	0	0	0	0	0	0	0	0	0	0	0	0	0
MDM2	gene	0	0	0	0	0	0	0	0	0	0	0	0	0	0
MDM4	gene	0	0	0	0	0	0	0	0	0	0	0	0	0	0
DRD2	gene	0	0	0	0	0	0	0	0	0	0	0	0	0	0
DNMT1	gene	0	0	0	0	0	0	0	0	0	0	0	0	0	0
CFTR	gene	0	0	0	0	0	0	0	0	0	0	0	0	0	0
TDP1	gene	0	0	0	0	0	0	0	0	0	0	0	0	0	0
AKR1B10	gene	0	0	0	0	0	0	0	0	0	0	0	0	0	0
AKR1B1	gene	0	0	0	0	0	0	0	0	0	0	0	0	0	0
ALB	gene	0	0	0	0	0	0	0	0	0	0	0	0	0	0
IFNG	gene	0	0	0	0	0	0	0	0	0	0	0	0	0	0
POLB	gene	0	0	0	0	0	0	0	0	0	0	0	0	0	0
AKR1B10	gene	0	0	0	0	0	0	0	0	0	0	0	0	0	0
SAE1 UBA2	gene	0	0	0	0	0	0	0	0	0	0	0	0	0	0
HMOX1	gene	0	0	0	0	0	0	0	0	0	0	0	0	0	0
DNMT1	gene	0	0	0	0	0	0	0	0	0	0	0	0	0	0
PTGS2	gene	0	0	0	0	0	0	0	0	0	0	0	0	0	0
SLC47A1	gene	0	0	0	0	0	0	0	0	0	0	0	0	0	0
APOB	gene	0	0	0	0	0	0	0	0	0	0	0	0	0	0
PON1	gene	0	0	0	0	0	0	0	0	0	0	0	0	0	0
BACE1	gene	0	0	0	0	0	0	0	0	0	0	0	0	0	0
SLC22A11	gene	0	0	0	0	0	0	0	0	0	0	0	0	0	0
CSF2	gene	0	0	0	0	0	0	0	0	0	0	0	0	0	0
KIAA1149	gene	0	0	0	0	0	0	0	0	0	0	0	0	0	0
OPRK1	gene	0	0	0	0	0	0	0	0	0	0	0	0	0	0
OPRM1	gene	0	0	0	0	0	0	0	0	0	0	0	0	0	0
OPRD1	gene	0	0	0	0	0	0	0	0	0	0	0	0	0	0
PTPN1	gene	0	0	0	0	0	0	0	0	0	0	0	0	0	0
PKD1	gene	0	0	0	0	0	0	0	0	0	0	0	0	0	0
SMO	gene	0	0	0	0	0	0	0	0	0	0	0	0	0	0
SHH	gene	0	0	0	0	0	0	0	0	0	0	0	0	0	0
NR1H4	gene	0	0	0	0	0	0	0	0	0	0	0	0	0	0
ESRRA	gene	0	0	0	0	0	0	0	0	0	0	0	0	0	0
GLI3	gene	0	0	0	0	0	0	0	0	0	0	0	0	0	0
CHRM2	gene	0	0	0	0	0	0	0	0	0	0	0	0	0	0
ADRB3	gene	0	0	0	0	0	0	0	0	0	0	0	0	0	0
BCHE	gene	0	0	0	0	0	0	0	0	0	0	0	0	0	0
SCN5A	gene	0	0	0	0	0	0	0	0	0	0	0	0	0	0
KCNH2	gene	0	0	0	0	0	0	0	0	0	0	0	0	0	0
ABCB1	gene	0	0	0	0	0	0	0	0	0	0	0	0	0	0
KCNA5	gene	0	0	0	0	0	0	0	0	0	0	0	0	0	0
RELA	gene	0	0	0	0	0	0	0	0	0	0	0	0	0	0
NFKBIA	gene	0	0	0	0	0	0	0	0	0	0	0	0	0	0
MAPK8	gene	0	0	0	0	0	0	0	0	0	0	0	0	0	0
ATG12	gene	0	0	0	0	0	0	0	0	0	0	0	0	0	0
TBK1	gene	0	0	0	0	0	0	0	0	0	0	0	0	0	0
HSPA6	gene	0	0	0	0	0	0	0	0	0	0	0	0	0	0
VCAM1	gene	0	0	0	0	0	0	0	0	0	0	0	0	0	0
STAR	gene	0	0	0	0	0	0	0	0	0	0	0	0	0	0
CASP9	gene	0	0	0	0	0	0	0	0	0	0	0	0	0	0
MDM2	gene	0	0	0	0	0	0	0	0	0	0	0	0	0	0
TNFSF10	gene	0	0	0	0	0	0	0	0	0	0	0	0	0	0
MAPT	gene	0	0	0	0	0	0	0	0	0	0	0	0	0	0
HTT	gene	0	0	0	0	0	0	0	0	0	0	0	0	0	0
TNF	gene	0	0	0	0	0	0	0	0	0	0	0	0	0	0
GPR55	gene	0	0	0	0	0	0	0	0	0	0	0	0	0	0
APLNR	gene	0	0	0	0	0	0	0	0	0	0	0	0	0	0
NFKB1	gene	0	0	0	0	0	0	0	0	0	0	0	0	0	0
NFKB2	gene	0	0	0	0	0	0	0	0	0	0	0	0	0	0
RGS4	gene	0	0	0	0	0	0	0	0	0	0	0	0	0	0
AGTR1	gene	0	0	0	0	0	0	0	0	0	0	0	0	0	0
MITF	gene	0	0	0	0	0	0	0	0	0	0	0	0	0	0
MDM4	gene	0	0	0	0	0	0	0	0	0	0	0	0	0	0
XBP1	gene	0	0	0	0	0	0	0	0	0	0	0	0	0	0
NFE2L2	gene	0	0	0	0	0	0	0	0	0	0	0	0	0	0
ATAD5	gene	0	0	0	0	0	0	0	0	0	0	0	0	0	0
BRCA1	gene	0	0	0	0	0	0	0	0	0	0	0	0	0	0
BARD1	gene	0	0	0	0	0	0	0	0	0	0	0	0	0	0
CFTR	gene	0	0	0	0	0	0	0	0	0	0	0	0	0	0
SMAD3	gene	0	0	0	0	0	0	0	0	0	0	0	0	0	0
NR2E3	gene	0	0	0	0	0	0	0	0	0	0	0	0	0	0
PLIN5	gene	0	0	0	0	0	0	0	0	0	0	0	0	0	0
ABHD5	gene	0	0	0	0	0	0	0	0	0	0	0	0	0	0
GMNN	gene	0	0	0	0	0	0	0	0	0	0	0	0	0	0
EPAS1	gene	0	0	0	0	0	0	0	0	0	0	0	0	0	0
TNFRSF10B	gene	0	0	0	0	0	0	0	0	0	0	0	0	0	0
GLP1R	gene	0	0	0	0	0	0	0	0	0	0	0	0	0	0
GPR183	gene	0	0	0	0	0	0	0	0	0	0	0	0	0	0
ATXN2	gene	0	0	0	0	0	0	0	0	0	0	0	0	0	0
HNF4A	gene	0	0	0	0	0	0	0	0	0	0	0	0	0	0
NCOA2	gene	0	0	0	0	0	0	0	0	0	0	0	0	0	0
DRD3	gene	0	0	0	0	0	0	0	0	0	0	0	0	0	0
PAX8	gene	0	0	0	0	0	0	0	0	0	0	0	0	0	0
NR2F3	gene	0	0	0	0	0	0	0	0	0	0	0	0	0	0
IDH1	gene	0	0	0	0	0	0	0	0	0	0	0	0	0	0
PRNP	gene	0	0	0	0	0	0	0	0	0	0	0	0	0	0
IL1B	gene	0	0	0	0	0	0	0	0	0	0	0	0	0	0
TERT	gene	0	0	0	0	0	0	0	0	0	0	0	0	0	0
SLCO1B1	gene	0	0	0	0	0	0	0	0	0	0	0	0	0	0
SLCO1B3	gene	0	0	0	0	0	0	0	0	0	0	0	0	0	0
ABCB11	gene	0	0	0	0	0	0	0	0	0	0	0	0	0	0
VLDLR	gene	0	0	0	0	0	0	0	0	0	0	0	0	0	0
RBP1	gene	0	0	0	0	0	0	0	0	0	0	0	0	0	0
RBP2	gene	0	0	0	0	0	0	0	0	0	0	0	0	0	0
RBP3	gene	0	0	0	0	0	0	0	0	0	0	0	0	0	0
RBP4	gene	0	0	0	0	0	0	0	0	0	0	0	0	0	0
RBP5	gene	0	0	0	0	0	0	0	0	0	0	0	0	0	0

BMP2	gene	0	0	0	0	0	0	0	0	0	0	0	0	0	0
AR	gene	0	0	0	0	0	0	0	0	0	0	0	0	0	0
GLI3	gene	0	0	0	0	0	0	0	0	0	0	0	0	0	0
NR1I2	gene	0	0	0	0	0	0	0	0	0	0	0	0	0	0
AR	gene	0	0	0	0	0	0	0	0	0	0	0	0	0	0
GLI3	gene	0	0	0	0	0	0	0	0	0	0	0	0	0	0
NR1I2	gene	0	0	0	0	0	0	0	0	0	0	0	0	0	0
AR	gene	0	0	0	0	0	0	0	0	0	0	0	0	0	0
GLI3	gene	0	0	0	0	0	0	0	0	0	0	0	0	0	0
NR1I2	gene	0	0	0	0	0	0	0	0	0	0	0	0	0	0
CYP1A2	gene	0	0	0	0	0	0	0	0	0	0	0	0	0	0
NOS3	gene	0	0	0	0	0	0	0	0	0	0	0	0	0	0
NOS2	gene	0	0	0	0	0	0	0	0	0	0	0	0	0	0
NOS1	gene	0	0	0	0	0	0	0	0	0	0	0	0	0	0
SLC7A3	gene	0	0	0	0	0	0	0	0	0	0	0	0	0	0
AZIN2	gene	0	0	0	0	0	0	0	0	0	0	0	0	0	0
ASL	gene	0	0	0	0	0	0	0	0	0	0	0	0	0	0
SLC7A1	gene	0	0	0	0	0	0	0	0	0	0	0	0	0	0
ARG2	gene	0	0	0	0	0	0	0	0	0	0	0	0	0	0
SLC7A4	gene	0	0	0	0	0	0	0	0	0	0	0	0	0	0
ASS1	gene	0	0	0	0	0	0	0	0	0	0	0	0	0	0
PRMT1	gene	0	0	0	0	0	0	0	0	0	0	0	0	0	0
SRF	gene	0	0	0	0	0	0	0	0	0	0	0	0	0	0
CARM1	gene	0	0	0	0	0	0	0	0	0	0	0	0	0	0
PRMT5	gene	0	0	0	0	0	0	0	0	0	0	0	0	0	0
AMD1	gene	0	0	0	0	0	0	0	0	0	0	0	0	0	0
ODC1	gene	0	0	0	0	0	0	0	0	0	0	0	0	0	0
GCG	gene	0	0	0	0	0	0	0	0	0	0	0	0	0	0
CA1	gene	0	0	0	0	0	0	0	0	0	0	0	0	0	0
CA2	gene	0	0	0	0	0	0	0	0	0	0	0	0	0	0
CA4	gene	0	0	0	0	0	0	0	0	0	0	0	0	0	0
CA9	gene	0	0	0	0	0	0	0	0	0	0	0	0	0	0
CA7	gene	0	0	0	0	0	0	0	0	0	0	0	0	0	0
CA14	gene	0	0	0	0	0	0	0	0	0	0	0	0	0	0
CA5A	gene	0	0	0	0	0	0	0	0	0	0	0	0	0	0
CA5B	gene	0	0	0	0	0	0	0	0	0	0	0	0	0	0
CA6	gene	0	0	0	0	0	0	0	0	0	0	0	0	0	0
SLC15A4	gene	0	0	0	0	0	0	0	0	0	0	0	0	0	0
SLC15A3	gene	0	0	0	0	0	0	0	0	0	0	0	0	0	0
SIRPA	gene	0	0	0	0	0	0	0	0	0	0	0	0	0	0
CD47	gene	0	0	0	0	0	0	0	0	0	0	0	0	0	0
SLC7A5	gene	0	0	0	0	0	0	0	0	0	0	0	0	0	0
HDC	gene	0	0	0	0	0	0	0	0	0	0	0	0	0	0
HARS	gene	0	0	0	0	0	0	0	0	0	0	0	0	0	0
SLC38A3	gene	0	0	0	0	0	0	0	0	0	0	0	0	0	0
HAL	gene	0	0	0	0	0	0	0	0	0	0	0	0	0	0
EEF2	gene	0	0	0	0	0	0	0	0	0	0	0	0	0	0
CAT	gene	0	0	0	0	0	0	0	0	0	0	0	0	0	0
MB	gene	0	0	0	0	0	0	0	0	0	0	0	0	0	0
TYR	gene	0	0	0	0	0	0	0	0	0	0	0	0	0	0
PRDM10	gene	0	0	0	0	0	0	0	0	0	0	0	0	0	0
GLUL	gene	0	0	0	0	0	0	0	0	0	0	0	0	0	0
SLC39A6	gene	0	0	0	0	0	0	0	0	0	0	0	0	0	0
HMOX2	gene	0	0	0	0	0	0	0	0	0	0	0	0	0	0
BCHE	gene	0	0	0	0	0	0	0	0	0	0	0	0	0	0
SLC7A1	gene	0	0	0	0	0	0	0	0	0	0	0	0	0	0
SLC7A4	gene	0	0	0	0	0	0	0	0	0	0	0	0	0	0
SLC7A3	gene	0	0	0	0	0	0	0	0	0	0	0	0	0	0
SLC7A2	gene	0	0	0	0	0	0	0	0	0	0	0	0	0	0
KARS	gene	0	0	0	0	0	0	0	0	0	0	0	0	0	0
H3F3A	gene	0	0	0	0	0	0	0	0	0	0	0	0	0	0
H3F3B	gene	0	0	0	0	0	0	0	0	0	0	0	0	0	0
HIST1H4A	gene	0	0	0	0	0	0	0	0	0	0	0	0	0	0
HIST1H4F	gene	0	0	0	0	0	0	0	0	0	0	0	0	0	0
HIST2H4A	gene	0	0	0	0	0	0	0	0	0	0	0	0	0	0
HIST1H4C	gene	0	0	0	0	0	0	0	0	0	0	0	0	0	0
HIST1H4B	gene	0	0	0	0	0	0	0	0	0	0	0	0	0	0
HIST2H4B	gene	0	0	0	0	0	0	0	0	0	0	0	0	0	0
HIST1H4E	gene	0	0	0	0	0	0	0	0	0	0	0	0	0	0
HIST4H4	gene	0	0	0	0	0	0	0	0	0	0	0	0	0	0
TARS	gene	0	0	0	0	0	0	0	0	0	0	0	0	0	0
TARS2	gene	0	0	0	0	0	0	0	0	0	0	0	0	0	0
NTSR2	gene	0	0	0	0	0	0	0	0	0	0	0	0	0	0
NTSR1	gene	0	0	0	0	0	0	0	0	0	0	0	0	0	0
TASP1	gene	0	0	0	0	0	0	0	0	0	0	0	0	0	0
PPP1CB	gene	0	0	0	0	0	0	0	0	0	0	0	0	0	0
NTS	gene	0	0	0	0	0	0	0	0	0	0	0	0	0	0
JUN	gene	0	0	0	0	0	0	0	0	0	0	0	0	0	0
MAPK3	gene	0	0	0	0	0	0	0	0	0	0	0	0	0	0
SP1	gene	0	0	0	0	0	0	0	0	0	0	0	0	0	0
MMP13	gene	0	0	0	0	0	0	0	0	0	0	0	0	0	0
MAPK1	gene	0	0	0	0	0	0	0	0	0	0	0	0	0	0
CDK4	gene	0	0	0	0	0	0	0	0	0	0	0	0	0	0
ESR1	gene	0	0	0	0	0	0	0	0	0	0	0	0	0	0
JUN	gene	0	0	0	0	0	0	0	0	0	0	0	0	0	0
MAPK3	gene	0	0	0	0	0	0	0	0	0	0	0	0	0	0
SP1	gene	0	0	0	0	0	0	0	0	0	0	0	0	0	0
MMP13	gene	0	0	0	0	0	0	0	0	0	0	0	0	0	0
MAPK1	gene	0	0	0	0	0	0	0	0	0	0	0	0	0	0
CDK4	gene	0	0	0	0	0	0	0	0	0	0	0	0	0	0
ESR1	gene	0	0	0	0	0	0	0	0	0	0	0	0	0	0
PPARG	gene	0	0	0	0	0	0	0	0	0	0	0	0	0	0
HSPB1	gene	0	0	0	0	0	0	0	0	0	0	0	0	0	0
RARA	gene	0	0	0	0	0	0	0	0	0	0	0	0	0	0
GPR32	gene	0	0	0	0	0	0	0	0	0	0	0	0	0	0
NR1I2	gene	0	0	0	0	0	0	0	0	0	0	0	0	0	0
NFE2L2	gene	0	0	0	0	0	0	0	0	0	0	0	0	0	0
PPARD	gene	0	0	0	0	0	0	0	0	0	0	0	0	0	0
VDR	gene	0	0	0	0	0	0	0	0	0	0	0	0	0	0
PPARG	gene	0	0	0	0	0	0	0	0	0	0	0	0	0	0
HSPB1	gene	0	0	0	0	0	0	0	0	0	0	0	0	0	0
RARA	gene	0	0	0	0	0	0	0	0	0	0	0	0	0	0
GPR32	gene	0	0	0	0	0	0	0	0	0	0	0	0	0	0
NR1I2	gene	0	0	0	0	0	0	0	0	0	0	0	0	0	0
NFE2L2	gene	0	0	0	0	0	0	0	0	0	0	0	0	0	0
PPARD	gene	0	0	0	0	0	0	0	0	0	0	0	0	0	0
VDR	gene	0	0	0	0	0	0	0	0	0	0	0	0	0	0
NFE2L2	gene	0	0	0	0	0	0	0	0	0	0	0	0	0	0
ABCB1	gene	0	0	0	0	0	0	0	0	0	0	0	0	0	0
RORA	gene	0	0	0	0	0	0	0	0	0	0	0	0	0	0
RORC	gene	0	0	0	0	0	0	0	0	0	0	0	0	0	0
FAM123B	gene	0	0	0	0	0	0	0	0	0	0	0	0	0	0
CYP51A1	gene	0	0	0	0	0	0	0	0	0	0	0	0	0	0
CYP51A1	gene	0	0	0	0	0	0	0	0	0	0	0	0	0	0
CRYAA	gene	0	0	0	0	0	0	0	0	0	0	0	0	0	0
CRYBB2	gene	0	0	0	0	0	0	0	0	0	0	0	0	0	0
CRYGC	gene	0	0	0	0	0	0	0	0	0	0	0	0	0	0
CRYGD	gene	0	0	0	0	0	0	0	0	0	0	0	0	0	0
CRYAB	gene	0	0	0	0	0	0	0	0	0	0	0	0	0	0
LSS	gene	0	0	0	0	0	0	0	0	0	0	0	0	0	0
CHRM3	gene	0	0	0	0	0	0	0	0	0	0	0	0	0	0
CHRM1	gene	0	0	0	0	0	0	0	0	0	0	0	0	0	0
CHRM4	gene	0	0	0	0	0	0	0	0	0	0	0	0	0	0
CHRM5	gene	0	0	0	0	0	0	0	0	0	0	0	0	0	0
CHRM2	gene	0	0	0	0	0	0	0	0	0	0	0	0	0	0
ARMC3	gene	0	0	0	0	0	0	0	0	0	0	0	0	0	0
HRH1	gene	0	0	0	0	0	0	0	0	0	0	0	0	0	0
ARMCX3	gene	0	0	0	0	0	0	0	0	0	0	0	0	0	0
JUP	gene	0	0	0	0	0	0	0	0	0	0	0	0	0	0
BCHE	gene	0	0	0	0	0	0	0	0	0	0	0	0	0	0
KAT2A	gene	0	0	0	0	0	0	0	0	0	0	0	0	0	0
ACHE	gene	0	0	0	0	0	0	0	0	0	0	0	0	0	0
ADRA1D	gene	0	0	0	0	0	0	0	0	0	0	0	0	0	0
HTR2C	gene	0	0	0	0	0	0	0	0	0	0	0	0	0	0
CYP3A4	gene	0	0	0	0	0	0	0	0	0	0	0	0	0	0
ALOX15	gene	0	0	0	0	0	0	0	0	0	0	0	0	0	0
ALDH1A1	gene	0	0	0	0	0	0	0	0	0	0	0	0	0	0
NFE2L2	gene	0	0	0	0	0	0	0	0	0	0	0	0	0	0
NR3C1	gene	0	0	0	0	0	0	0	0	0	0	0	0	0	0
GLI3	gene	0	0	0	0	0	0	0	0	0	0	0	0	0	0
ESR2	gene	0	0	0	0	0	0	0	0	0	0	0	0	0	0
ESRRA	gene	0	0	0	0	0	0	0	0	0	0	0	0	0	0
NLRP3	gene	0	0	0	0	0	0	0	0	0	0	0	0	0	0
UGT1A9	gene	0	0	0	0	0	0	0	0	0	0	0	0	0	0
ALDH1A1	gene	0	0	0	0	0	0	0	0	0	0	0	0	0	0
ELANE	gene	0	0	0	0	0	0	0	0	0	0	0	0	0	0
VDR	gene	0	0	0	0	0	0	0	0	0	0	0	0	0	0
NFE2L2	gene	0	0	0	0	0	0	0	0	0	0	0	0	0	0
AHR	gene	0	0	0	0	0	0	0	0	0	0	0	0	0	0
NR1I3	gene	0	0	0	0	0	0	0	0	0	0	0	0	0	0
ESRRA	gene	0	0	0	0	0	0	0	0	0	0	0	0	0	0
AR	gene	0	0	0	0	0	0	0	0	0	0	0	0	0	0
HDAC9	gene	0	0	0	0	0	0	0	0	0	0	0	0	0	0
PGR	gene	0	0	0	0	0	0	0	0	0	0	0	0	0	0
CSNK2A1	gene	0	0	0	0	0	0	0	0	0	0	0	0	0	0
CASP3	gene	0	0	0	0	0	0	0	0	0	0	0	0	0	0
VEGFA	gene	0	0	0	0	0	0	0	0	0	0	0	0	0	0
CYP1B1	gene	0	0	0	0	0	0	0	0	0	0	0	0	0	0
ERBB2	gene	0	0	0	0	0	0	0	0	0	0	0	0	0	0
TP53	gene	0	0	0	0	0	0	0	0	0	0	0	0	0	0
LCK	gene	0	0	0	0	0	0	0	0	0	0	0	0	0	0
MCL1	gene	0	0	0	0	0	0	0	0	0	0	0	0	0	0
CXCR4	gene	0	0	0	0	0	0	0	0	0	0	0	0	0	0
PIM1	gene	0	0	0	0	0	0	0	0	0	0	0	0	0	0
CYP1A2	gene	0	0	0	0	0	0	0	0	0	0	0	0	0	0
CLK1	gene	0	0	0	0	0	0	0	0	0	0	0	0	0	0
PIM2	gene	0	0	0	0	0	0	0	0	0	0	0	0	0	0
MAPT	gene	0	0	0	0	0	0	0	0	0	0	0	0	0	0
CYP2D6	gene	0	0	0	0	0	0	0	0	0	0	0	0	0	0
CYP3A4	gene	0	0	0	0	0	0	0	0	0	0	0	0	0	0
CYP2C9	gene	0	0	0	0	0	0	0	0	0	0	0	0	0	0
CYP2C19	gene	0	0	0	0	0	0	0	0	0	0	0	0	0	0
ALDH1A1	gene	0	0	0	0	0	0	0	0	0	0	0	0	0	0
THPO	gene	0	0	0	0	0	0	0	0	0	0	0	0	0	0
SENP8	gene	0	0	0	0	0	0	0	0	0	0	0	0	0	0
KCNQ1	gene	0	0	0	0	0	0	0	0	0	0	0	0	0	0
SENP6	gene	0	0	0	0	0	0	0	0	0	0	0	0	0	0
ESR1	gene	0	0	0	0	0	0	0	0	0	0	0	0	0	0
ESR2	gene	0	0	0	0	0	0	0	0	0	0	0	0	0	0
SENP7	gene	0	0	0	0	0	0	0	0	0	0	0	0	0	0
PLA2G7	gene	0	0	0	0	0	0	0	0	0	0	0	0	0	0
POLB	gene	0	0	0	0	0	0	0	0	0	0	0	0	0	0
FEN1	gene	0	0	0	0	0	0	0	0	0	0	0	0	0	0
CBX1	gene	0	0	0	0	0	0	0	0	0	0	0	0	0	0
MPHOSPH8	gene	0	0	0	0	0	0	0	0	0	0	0	0	0	0
PTP4A3	gene	0	0	0	0	0	0	0	0	0	0	0	0	0	0
CSNK2B	gene	0	0	0	0	0	0	0	0	0	0	0	0	0	0
NFE2L2	gene	0	0	0	0	0	0	0	0	0	0	0	0	0	0
CXCL8	gene	0	0	0	0	0	0	0	0	0	0	0	0	0	0
AHR	gene	0	0	0	0	0	0	0	0	0	0	0	0	0	0
RORC	gene	0	0	0	0	0	0	0	0	0	0	0	0	0	0
PIP4K2A	gene	0	0	0	0	0	0	0	0	0	0	0	0	0	0
TDP1	gene	0	0	0	0	0	0	0	0	0	0	0	0	0	0
NR3C1	gene	0	0	0	0	0	0	0	0	0	0	0	0	0	0
AR	gene	0	0	0	0	0	0	0	0	0	0	0	0	0	0
CYP19A1	gene	0	0	0	0	0	0	0	0	0	0	0	0	0	0
PPARG	gene	0	0	0	0	0	0	0	0	0	0	0	0	0	0
NR1H4	gene	0	0	0	0	0	0	0	0	0	0	0	0	0	0
CSNK2A2	gene	0	0	0	0	0	0	0	0	0	0	0	0	0	0
RARA	gene	0	0	0	0	0	0	0	0	0	0	0	0	0	0
NR1I3	gene	0	0	0	0	0	0	0	0	0	0	0	0	0	0
EPHX2	gene	0	0	0	0	0	0	0	0	0	0	0	0	0	0
HDAC9	gene	0	0	0	0	0	0	0	0	0	0	0	0	0	0
GLI3	gene	0	0	0	0	0	0	0	0	0	0	0	0	0	0
AKR1B1	gene	0	0	0	0	0	0	0	0	0	0	0	0	0	0
PGR	gene	0	0	0	0	0	0	0	0	0	0	0	0	0	0
NR1I2	gene	0	0	0	0	0	0	0	0	0	0	0	0	0	0
NPR1	gene	0	0	0	0	0	0	0	0	0	0	0	0	0	0
NTMT1	gene	0	0	0	0	0	0	0	0	0	0	0	0	0	0
ACHE	gene	0	0	0	0	0	0	0	0	0	0	0	0	0	0
CYP1A1	gene	0	0	0	0	0	0	0	0	0	0	0	0	0	0
EZH2	gene	0	0	0	0	0	0	0	0	0	0	0	0	0	0
AKR1B1	gene	0	0	0	0	0	0	0	0	0	0	0	0	0	0
CA2	gene	0	0	0	0	0	0	0	0	0	0	0	0	0	0
CA7	gene	0	0	0	0	0	0	0	0	0	0	0	0	0	0
CA12	gene	0	0	0	0	0	0	0	0	0	0	0	0	0	0
CA4	gene	0	0	0	0	0	0	0	0	0	0	0	0	0	0
NOX4	gene	0	0	0	0	0	0	0	0	0	0	0	0	0	0
ADRA2C	gene	0	0	0	0	0	0	0	0	0	0	0	0	0	0
ACHE	gene	0	0	0	0	0	0	0	0	0	0	0	0	0	0
UGT1A9	gene	0	0	0	0	0	0	0	0	0	0	0	0	0	0
ACACA	gene	0	0	0	0	0	0	0	0	0	0	0	0	0	0
MGAM	gene	0	0	0	0	0	0	0	0	0	0	0	0	0	0
ELANE	gene	0	0	0	0	0	0	0	0	0	0	0	0	0	0
VDR	gene	0	0	0	0	0	0	0	0	0	0	0	0	0	0
PGD	gene	0	0	0	0	0	0	0	0	0	0	0	0	0	0
GFER	gene	0	0	0	0	0	0	0	0	0	0	0	0	0	0
POLB	gene	0	0	0	0	0	0	0	0	0	0	0	0	0	0
KCNN4	gene	0	0	0	0	0	0	0	0	0	0	0	0	0	0
LRRC26	gene	0	0	0	0	0	0	0	0	0	0	0	0	0	0
KCNMA1	gene	0	0	0	0	0	0	0	0	0	0	0	0	0	0
KCNMB1	gene	0	0	0	0	0	0	0	0	0	0	0	0	0	0
KCNMB2	gene	0	0	0	0	0	0	0	0	0	0	0	0	0	0
UGT1A9	gene	0	0	0	0	0	0	0	0	0	0	0	0	0	0
RXRA	gene	0	0	0	0	0	0	0	0	0	0	0	0	0	0
FTO	gene	0	0	0	0	0	0	0	0	0	0	0	0	0	0
PPARG	gene	0	0	0	0	0	0	0	0	0	0	0	0	0	0
UCP1	gene	0	0	0	0	0	0	0	0	0	0	0	0	0	0
PPARA	gene	0	0	0	0	0	0	0	0	0	0	0	0	0	0
DNM1L	gene	0	0	0	0	0	0	0	0	0	0	0	0	0	0
RELA	gene	0	0	0	0	0	0	0	0	0	0	0	0	0	0
VEGFA	gene	0	0	0	0	0	0	0	0	0	0	0	0	0	0
NFKB1	gene	0	0	0	0	0	0	0	0	0	0	0	0	0	0
DNM1	gene	0	0	0	0	0	0	0	0	0	0	0	0	0	0
HSP90AB1	gene	0	0	0	0	0	0	0	0	0	0	0	0	0	0
PTPN7	gene	0	0	0	0	0	0	0	0	0	0	0	0	0	0
ALOX15	gene	0	0	0	0	0	0	0	0	0	0	0	0	0	0
MCL1	gene	0	0	0	0	0	0	0	0	0	0	0	0	0	0
MAPT	gene	0	0	0	0	0	0	0	0	0	0	0	0	0	0
RBBP9	gene	0	0	0	0	0	0	0	0	0	0	0	0	0	0
GSTO1	gene	0	0	0	0	0	0	0	0	0	0	0	0	0	0
BCL2L1	gene	0	0	0	0	0	0	0	0	0	0	0	0	0	0
BLM	gene	0	0	0	0	0	0	0	0	0	0	0	0	0	0
PLA2G7	gene	0	0	0	0	0	0	0	0	0	0	0	0	0	0
POLB	gene	0	0	0	0	0	0	0	0	0	0	0	0	0	0
PAFAH2	gene	0	0	0	0	0	0	0	0	0	0	0	0	0	0
CTDSP1	gene	0	0	0	0	0	0	0	0	0	0	0	0	0	0
CCR6	gene	0	0	0	0	0	0	0	0	0	0	0	0	0	0
HKDC1	gene	0	0	0	0	0	0	0	0	0	0	0	0	0	0
BAZ2B	gene	0	0	0	0	0	0	0	0	0	0	0	0	0	0
DNMT1	gene	0	0	0	0	0	0	0	0	0	0	0	0	0	0
POLK	gene	0	0	0	0	0	0	0	0	0	0	0	0	0	0
POLI	gene	0	0	0	0	0	0	0	0	0	0	0	0	0	0
POLH	gene	0	0	0	0	0	0	0	0	0	0	0	0	0	0
CXCR6	gene	0	0	0	0	0	0	0	0	0	0	0	0	0	0
GLS	gene	0	0	0	0	0	0	0	0	0	0	0	0	0	0
TP53	gene	0	0	0	0	0	0	0	0	0	0	0	0	0	0
RBBP8	gene	0	0	0	0	0	0	0	0	0	0	0	0	0	0
SIX1	gene	0	0	0	0	0	0	0	0	0	0	0	0	0	0
WRN	gene	0	0	0	0	0	0	0	0	0	0	0	0	0	0
RORC	gene	0	0	0	0	0	0	0	0	0	0	0	0	0	0
PIP4K2A	gene	0	0	0	0	0	0	0	0	0	0	0	0	0	0
SIRT5	gene	0	0	0	0	0	0	0	0	0	0	0	0	0	0
PRMT1	gene	0	0	0	0	0	0	0	0	0	0	0	0	0	0
CASP6	gene	0	0	0	0	0	0	0	0	0	0	0	0	0	0
FEN1	gene	0	0	0	0	0	0	0	0	0	0	0	0	0	0
ESR1	gene	0	0	0	0	0	0	0	0	0	0	0	0	0	0
NFE2L2	gene	0	0	0	0	0	0	0	0	0	0	0	0	0	0
BCHE	gene	0	0	0	0	0	0	0	0	0	0	0	0	0	0
HDAC9	gene	0	0	0	0	0	0	0	0	0	0	0	0	0	0
AHSG	gene	0	0	0	0	0	0	0	0	0	0	0	0	0	0
CA10	gene	0	0	0	0	0	0	0	0	0	0	0	0	0	0
MMP20	gene	0	0	0	0	0	0	0	0	0	0	0	0	0	0
IBSP	gene	0	0	0	0	0	0	0	0	0	0	0	0	0	0
SSP1	gene	0	0	0	0	0	0	0	0	0	0	0	0	0	0
AMBN	gene	0	0	0	0	0	0	0	0	0	0	0	0	0	0
ENAM	gene	0	0	0	0	0	0	0	0	0	0	0	0	0	0
DSPP	gene	0	0	0	0	0	0	0	0	0	0	0	0	0	0
MMP25	gene	0	0	0	0	0	0	0	0	0	0	0	0	0	0
MT-CO3	gene	0	0	0	0	0	0	0	0	0	0	0	0	0	0
CASR	gene	0	0	0	0	0	0	0	0	0	0	0	0	0	0
CALR	gene	0	0	0	0	0	0	0	0	0	0	0	0	0	0
CIB1	gene	0	0	0	0	0	0	0	0	0	0	0	0	0	0
PDCD6	gene	0	0	0	0	0	0	0	0	0	0	0	0	0	0
SRI	gene	0	0	0	0	0	0	0	0	0	0	0	0	0	0
CHP1	gene	0	0	0	0	0	0	0	0	0	0	0	0	0	0
SPARC	gene	0	0	0	0	0	0	0	0	0	0	0	0	0	0
CANX	gene	0	0	0	0	0	0	0	0	0	0	0	0	0	0
FBN2	gene	0	0	0	0	0	0	0	0	0	0	0	0	0	0
S100B	gene	0	0	0	0	0	0	0	0	0	0	0	0	0	0
CASQ2	gene	0	0	0	0	0	0	0	0	0	0	0	0	0	0
RGN	gene	0	0	0	0	0	0	0	0	0	0	0	0	0	0
PEF1	gene	0	0	0	0	0	0	0	0	0	0	0	0	0	0
S100A6	gene	0	0	0	0	0	0	0	0	0	0	0	0	0	0
TPT1	gene	0	0	0	0	0	0	0	0	0	0	0	0	0	0
CIB2	gene	0	0	0	0	0	0	0	0	0	0	0	0	0	0
S100A13	gene	0	0	0	0	0	0	0	0	0	0	0	0	0	0
CASQ1	gene	0	0	0	0	0	0	0	0	0	0	0	0	0	0
NUCB1	gene	0	0	0	0	0	0	0	0	0	0	0	0	0	0
NUCB2	gene	0	0	0	0	0	0	0	0	0	0	0	0	0	0
CALM1	gene	0	0	0	0	0	0	0	0	0	0	0	0	0	0
FBN3	gene	0	0	0	0	0	0	0	0	0	0	0	0	0	0
GCA	gene	0	0	0	0	0	0	0	0	0	0	0	0	0	0
CADPS	gene	0	0	0	0	0	0	0	0	0	0	0	0	0	0
CALM2	gene	0	0	0	0	0	0	0	0	0	0	0	0	0	0
CALM3	gene	0	0	0	0	0	0	0	0	0	0	0	0	0	0
S100A16	gene	0	0	0	0	0	0	0	0	0	0	0	0	0	0
CALB2	gene	0	0	0	0	0	0	0	0	0	0	0	0	0	0
CAPS	gene	0	0	0	0	0	0	0	0	0	0	0	0	0	0
CADPS2	gene	0	0	0	0	0	0	0	0	0	0	0	0	0	0
CALR3	gene	0	0	0	0	0	0	0	0	0	0	0	0	0	0
NRXN1	gene	0	0	0	0	0	0	0	0	0	0	0	0	0	0
NCS1	gene	0	0	0	0	0	0	0	0	0	0	0	0	0	0
SLC4A4	gene	0	0	0	0	0	0	0	0	0	0	0	0	0	0
SLC4A5	gene	0	0	0	0	0	0	0	0	0	0	0	0	0	0
SLC4A9	gene	0	0	0	0	0	0	0	0	0	0	0	0	0	0
HRH1	gene	0	0	0	0	0	0	0	0	0	0	0	0	0	0
KIAA0101	gene	0	0	0	0	0	0	0	0	0	0	0	0	0	0
F2	gene	0	0	0	0	0	0	0	0	0	0	0	0	0	0
CYP1A2	gene	0	0	0	0	0	0	0	0	0	0	0	0	0	0
CYP2D6	gene	0	0	0	0	0	0	0	0	0	0	0	0	0	0
CYP2C19	gene	0	0	0	0	0	0	0	0	0	0	0	0	0	0
BCL2A1	gene	0	0	0	0	0	0	0	0	0	0	0	0	0	0
RHAG	gene	0	0	0	0	0	0	0	0	0	0	0	0	0	0
SIRT1	gene	0	0	0	0	0	0	0	0	0	0	0	0	0	0
FABP4	gene	0	0	0	0	0	0	0	0	0	0	0	0	0	0
PPARG	gene	0	0	0	0	0	0	0	0	0	0	0	0	0	0
PPARA	gene	0	0	0	0	0	0	0	0	0	0	0	0	0	0
PPARD	gene	0	0	0	0	0	0	0	0	0	0	0	0	0	0
SRD5A1	gene	0	0	0	0	0	0	0	0	0	0	0	0	0	0
NFE2L2	gene	0	0	0	0	0	0	0	0	0	0	0	0	0	0
ABCB1	gene	0	0	0	0	0	0	0	0	0	0	0	0	0	0
RORA	gene	0	0	0	0	0	0	0	0	0	0	0	0	0	0
RORC	gene	0	0	0	0	0	0	0	0	0	0	0	0	0	0
CYP1B1	gene	0	0	0	0	0	0	0	0	0	0	0	0	0	0
CYP1A1	gene	0	0	0	0	0	0	0	0	0	0	0	0	0	0
MAPK8	gene	0	0	0	0	0	0	0	0	0	0	0	0	0	0
AKT1	gene	0	0	0	0	0	0	0	0	0	0	0	0	0	0
PON2	gene	0	0	0	0	0	0	0	0	0	0	0	0	0	0
HMOX1	gene	0	0	0	0	0	0	0	0	0	0	0	0	0	0
MAPK9	gene	0	0	0	0	0	0	0	0	0	0	0	0	0	0
NOS2	gene	0	0	0	0	0	0	0	0	0	0	0	0	0	0
ABCC1	gene	0	0	0	0	0	0	0	0	0	0	0	0	0	0
PRKCD	gene	0	0	0	0	0	0	0	0	0	0	0	0	0	0
XDH	gene	0	0	0	0	0	0	0	0	0	0	0	0	0	0
CYP1A2	gene	0	0	0	0	0	0	0	0	0	0	0	0	0	0
NFKB1	gene	0	0	0	0	0	0	0	0	0	0	0	0	0	0
NFKB2	gene	0	0	0	0	0	0	0	0	0	0	0	0	0	0
RELA	gene	0	0	0	0	0	0	0	0	0	0	0	0	0	0
CA2	gene	0	0	0	0	0	0	0	0	0	0	0	0	0	0
CA7	gene	0	0	0	0	0	0	0	0	0	0	0	0	0	0
CA4	gene	0	0	0	0	0	0	0	0	0	0	0	0	0	0
CA12	gene	0	0	0	0	0	0	0	0	0	0	0	0	0	0
TARDBP	gene	0	0	0	0	0	0	0	0	0	0	0	0	0	0
CYP2C9	gene	0	0	0	0	0	0	0	0	0	0	0	0	0	0
CYP1A2	gene	0	0	0	0	0	0	0	0	0	0	0	0	0	0
MCL1	gene	0	0	0	0	0	0	0	0	0	0	0	0	0	0
PTEN	gene	0	0	0	0	0	0	0	0	0	0	0	0	0	0
KCNH2	gene	0	0	0	0	0	0	0	0	0	0	0	0	0	0
MMP9	gene	0	0	0	0	0	0	0	0	0	0	0	0	0	0
CCND1	gene	0	0	0	0	0	0	0	0	0	0	0	0	0	0
Stigmasterol	MultiDrug	1	1	0	0	1	0	0	1	0	0	1	0	0	0
Beta-sitosterol	MultiDrug	0	1	1	1	0	1	1	1	1	0	1	0	0	0
Kaempferol	MultiDrug	0	0	0	0	0	0	1	0	1	0	0	0	0	0
Quercetin	MultiDrug	0	1	1	0	0	0	1	0	0	1	1	0	0	0
Diop	MultiDrug	0	0	0	1	1	0	0	0	1	0	0	0	0	0
Glycitein	MultiDrug	0	1	0	0	1	0	0	0	0	0	0	0	0	0
Mandenol	MultiDrug	0	1	0	0	0	0	0	0	0	0	1	0	0	0
CLR	MultiDrug	0	1	1	0	0	0	0	0	0	0	0	0	0	0
Cycloartenol	GQZ	0	1	0	0	0	0	0	0	0	0	0	0	0	0
LAN	GQZ	0	1	0	0	0	0	0	0	0	0	0	0	0	0
Atropine	GQZ	0	1	0	0	0	0	0	0	0	0	0	0	0	0
Ethyl linolenate	GQZ	0	1	0	0	0	0	0	0	0	0	0	0	0	0
Isorhamnetin	TSZ	0	0	1	0	0	0	0	0	0	0	0	0	0	0
Sesamin	TSZ	0	0	1	0	0	0	0	0	0	0	0	0	0	0
Matrine	TSZ	0	0	1	0	0	0	0	0	0	0	0	0	0	0
Supraene	BJT	0	0	0	1	0	0	0	0	0	0	0	0	0	0
Ohioensin-A	BJT	0	0	0	1	0	0	0	0	0	0	0	0	0	0
Luteolin	DS	0	0	0	0	1	0	0	0	0	0	0	0	0	0
7-Methoxy-2-methyl is	DS	0	0	0	0	1	0	0	0	0	0	0	0	0	0
Perlolyrine	DS	0	0	0	0	1	0	0	0	0	0	0	0	0	0
Mairin	DZ	0	0	0	0	0	0	1	0	0	0	0	0	0	0
ent-Epicatechin	DZ	0	0	0	0	0	0	1	0	0	0	0	0	0	0
Yangambin	DZ	0	0	0	0	0	0	1	0	0	0	0	0	0	0
(-)-Tabernemontanin	DZ	0	0	0	0	0	0	1	0	0	0	0	0	0	0
Cyclopamine	DZ	0	0	0	0	0	0	1	0	0	0	0	0	0	0
Cinchonan-9-al, 6'-met	DZ	0	0	0	0	0	0	1	0	0	0	0	0	0	0
Helenalin	DZ	0	0	0	0	0	0	1	0	0	0	0	0	0	0
Beta-Carotene	DZ	0	0	0	0	0	0	1	0	0	0	0	0	0	0
Syringetin	DZ	0	0	0	0	0	0	1	0	0	0	0	0	0	0
24-Ethylcholest-4-en-3	SR	0	0	0	0	0	0	0	1	0	0	0	0	0	0
Fumarine	RS	0	0	0	0	0	0	0	0	1	0	0	0	0	0
Ginsenoside rh2	RS	0	0	0	0	0	0	0	0	1	0	0	0	0	0
Arachidonate	RS	0	0	0	0	0	0	0	0	1	0	0	0	0	0
(2R)-5,7-dihydroxy-2	AY	0	0	0	0	0	0	0	0	0	0	1	0	0	0
Chrysophanol	HSW	0	0	0	0	0	0	0	0	0	0	0	1	0	0
Emodin	HSW	0	0	0	0	0	0	0	0	0	0	0	1	0	0
Guajavarin	HSW	0	0	0	0	0	0	0	0	0	0	0	1	0	0
Physcion	HSW	0	0	0	0	0	0	0	0	0	0	0	1	0	0
Piceid	HSW	0	0	0	0	0	0	0	0	0	0	0	1	0	0
Procyanidin B1 3'-O-ga	HSW	0	0	0	0	0	0	0	0	0	0	0	1	0	0
Rhein	HSW	0	0	0	0	0	0	0	0	0	0	0	1	0	0
Arginine	EJ	0	0	0	0	0	0	0	0	0	0	0	0	1	0
Histidine	EJ	0	0	0	0	0	0	0	0	0	0	0	0	1	0
Lysine	EJ	0	0	0	0	0	0	0	0	0	0	0	0	1	0
Threonine	EJ	0	0	0	0	0	0	0	0	0	0	0	0	1	0
Calcium Carbonate	LJJ	0	0	0	0	0	0	0	0	0	0	0	0	0	1
Calcium Phosphate	LJJ	0	0	0	0	0	0	0	0	0	0	0	0	0	1
