# Supplementary material for: Network Pharmacology Approach for Predicting Targets of Zishen Yutai Pills on Premature Ovarian Insufficiency
Source: Evid Based Complement Alternat Med. 2021 Aug 4;2021:8215454. doi: 10.1155/2021/8215454 (PMC8357500; doi:10.1155/2021/8215454)
Supplement: Supplementary Materials — Supplementary Table 1: active compounds of each herb in ZSYTP. Supplementary Table 2: corresponding targets of active compounds: (A) two target genes of SDH; (B) 138 target genes of GQZ; (C) 202 target genes of TSZ; (D) 25 target genes of BJT; (E) 121 target genes of DS; (F) 11 target genes of BZ; (G) 283 target genes of DZ; (H) 13 target genes of SR; (J) 112 target genes of RS; (K) 76 target genes of SJS; (L) 115 target genes of AY; (M) 149 target genes of HSW; (N) 68 target genes of EJ; (P) 46 target genes of LJJ. Supplementary Table 3: POI targets. Supplementary Table 4: compounds and targets attributes of C-T network. Supplementary Table 5: data of compound-target network. Supplementary Table 6: analysed C-T network: (A) active compounds of C-T network and corresponding degrees; (B) targets of C-T network and corresponding degrees. Supplementary Table 7: ZSYTP core PPI network. Supplementary Table 8: gene ontology and KEGG analysis: (A) GO-BP analysis; (B) GO-MF analysis; (C) GO-CC analysis; (D) KEGG pathway enrichment analysis. [file 8215454.f1.zip › 8215454.f1/ST6A Analysed C-T Network active compounds.pdf]

Supplementary Table 6A: Analysed Compound-Target Network

ID	Name	Type	Degree	AverageShortestPathLength	BetweennessCentrality	ClosenessCentrality	ClusteringCoefficient	Eccentricity	NeighborhoodConnectivity	NumberOfUndirectedEdges	PartnerOfMultiEdgedNodePairs	Radiality	Stress	TopologicalCoefficient
D	Quercetin	Herb	384	2.891	0.226	0.346	0.000	5.000	2.600	384	76	0.982	1,431,752	0.053
C	Kaempferol	Herb	255	2.773	0.236	0.361	0.000	5.000	3.563	255	84	0.983	2,000,366	0.071
DS1	Luteolin	Herb	104	2.895	0.243	0.345	0.000	5.000	2.854	104	1	0.982	2,090,456	0.071
B	Beta-sitosterol	Herb	97	3.154	0.091	0.317	0.000	7.000	4.400	97	11	0.979	445,282	0.087
HSW2	Emodin	Herb	58	3.092	0.114	0.323	0.000	5.000	3.310	58	0	0.980	903,626	0.083
DZ7	Helenalin	Herb	48	3.367	0.148	0.297	0.000	7.000	1.896	48	0	0.977	878,370	0.045
HSW7	Rhein	Herb	47	3.031	0.114	0.330	0.000	5.000	2.702	47	0	0.980	497,304	0.063
LJJ1	Calcium Phosphate	Herb	44	1.146	0.991	0.873	0.000	3.000	1.023	44	0	0.997	2,236	0.023
DZ1	Mairin	Herb	29	3.473	0.062	0.288	0.000	7.000	2.519	29	2	0.976	340,100	0.089
EJ2	Histidine	Herb	28	3.584	0.123	0.279	0.000	7.000	2.107	28	0	0.975	1,100,752	0.092
AY1	(2R)-5,7-dihydroxy-2-(4-hydr	Herb	20	3.461	0.040	0.289	0.000	5.000	3.550	20	0	0.976	262,928	0.128
TSZ2	Isorhamnetin	Herb	20	3.355	0.030	0.298	0.000	5.000	4.400	20	0	0.977	298,758	0.200
EJ1	Arginine	Herb	19	3.805	0.090	0.263	0.000	7.000	1.842	19	0	0.973	609,996	0.070
G	Mandenol	Herb	18	3.670	0.018	0.272	0.000	7.000	4.200	18	8	0.974	138,138	0.168
EJ3	Lysine	Herb	16	5.260	0.049	0.190	0.000	9.000	1.375	16	0	0.959	536,370	0.125
F	Glycitein	Herb	16	3.773	0.019	0.265	0.000	7.000	3.222	16	7	0.973	72,418	0.139
GQZ4	Atropine	Herb	15	3.703	0.045	0.270	0.000	5.000	2.133	15	0	0.974	172,156	0.071
DZ2	ent-Epicatechin	Herb	14	3.736	0.039	0.268	0.000	7.000	2.286	14	0	0.973	246,400	0.092
HSW1	Chrysophanol	Herb	13	3.822	0.012	0.262	0.000	7.000	4.077	13	0	0.973	136,062	0.192
A	Stigmasterol	Herb	12	3.617	0.011	0.276	0.000	7.000	5.429	12	1	0.975	50,344	0.193
E	Diop	Herb	12	4.031	0.012	0.248	0.000	7.000	5.167	12	3	0.971	79,644	0.245
BJT1	Supraene	Herb	11	5.023	0.041	0.199	0.000	9.000	1.273	11	0	0.961	188,790	0.091
DZ8	Beta-Carotene	Herb	10	3.932	0.037	0.254	0.000	7.000	2.100	10	0	0.972	134,424	0.100
HSW3	Guajavarin	Herb	10	3.535	0.013	0.283	0.000	5.000	4.900	10	0	0.975	94,070	0.260
GQZ5	Ethyl linolenate	Herb	9	3.596	0.006	0.278	0.000	7.000	5.333	9	0	0.975	50,918	0.228
DZ6	Cinchonan-9-al, 6'-methoxy-, (9	Herb	8	3.395	0.021	0.295	0.000	5.000	3.875	8	0	0.977	63,222	0.151
EJ4	Threonine	Herb	8	5.293	0.029	0.189	0.000	9.000	1.375	8	0	0.958	193,088	0.125
GQZ3	LAN	Herb	8	4.412	0.026	0.227	0.000	7.000	2.250	8	0	0.967	153,418	0.139
HSW5	Piceid	Herb	8	4.072	0.025	0.246	0.000	7.000	2.625	8	0	0.970	118,000	0.163
DS2	7-Methoxy-2-methyl isoflavone	Herb	7	3.961	0.003	0.252	0.000	5.000	5.286	7	0	0.971	28,768	0.330
HSW4	Physcion	Herb	7	4.227	0.013	0.237	0.000	7.000	2.857	7	0	0.969	71,598	0.206
RS1	Fumarine	Herb	7	3.707	0.010	0.270	0.000	5.000	4.571	7	0	0.974	56,474	0.238
DZ5	Cyclopamine	Herb	6	3.814	0.010	0.262	0.000	7.000	4.167	6	0	0.973	64,376	0.211
GQZ1	CLR	Herb	5	3.576	0.005	0.280	0.000	7.000	6.400	5	0	0.975	28,222	0.300
RS3	Arachidonate	Herb	5	4.207	0.006	0.238	0.000	7.000	3.600	5	0	0.969	47,920	0.260
TSZ1	CLR	Herb	5	3.654	0.003	0.274	0.000	7.000	5.800	5	0	0.974	15,590	0.300
TSZ3	Sesamin	Herb	5	3.699	0.003	0.270	0.000	5.000	5.600	5	0	0.974	25,114	0.354
TSZ4	Matrine	Herb	5	4.068	0.009	0.246	0.000	7.000	2.800	5	0	0.970	32,442	0.225
LJJ2	Calcium Carbonate	Herb	4	2.813	0.122	0.356	0.000	3.000	1.250	4	0	0.959	276	0.250
RS2	Ginsenoside rh2	Herb	4	4.494	0.012	0.223	0.000	7.000	2.500	4	0	0.966	50,190	0.250
GQZ2	Cycloartenol	Herb	3	4.432	0.006	0.226	0.000	7.000	4.333	3	0	0.967	43,838	0.370
BJT2	Ohioensin-A	Herb	2	5.055	0.000	0.198	0.000	9.000	3.000	2	0	0.961	1,170	0.500
DS3	Perlolyrine	Herb	2	4.605	0.004	0.217	0.000	7.000	3.500	2	0	0.965	24,206	0.500
DZ3	Yangambin	Herb	2	3.961	0.004	0.252	0.000	7.000	7.000	2	0	0.971	15,996	0.500
DZ4	(-)-Tabernemontanine	Herb	2	3.965	0.004	0.252	0.000	7.000	6.500	2	0	0.971	14,944	0.500
DZ9	Syringetin	Herb	2	3.965	0.004	0.252	0.000	7.000	6.500	2	0	0.971	14,944	0.500
HSW6	Procyanidin B1 3'-O-gallate	Herb	2	4.264	0.000	0.235	0.000	7.000	6.000	2	0	0.968	608	0.625
SR1	24-Ethylcholest-4-en-3-one	Herb	2	5.113	0.004	0.196	0.000	9.000	2.000	2	0	0.960	17,970	0.500

median degree 9.5
