# Supplementary material for: Network Pharmacology Approach for Predicting Targets of Zishen Yutai Pills on Premature Ovarian Insufficiency
Source: Evid Based Complement Alternat Med. 2021 Aug 4;2021:8215454. doi: 10.1155/2021/8215454 (PMC8357500; doi:10.1155/2021/8215454)
Supplement: Supplementary Materials — Supplementary Table 1: active compounds of each herb in ZSYTP. Supplementary Table 2: corresponding targets of active compounds: (A) two target genes of SDH; (B) 138 target genes of GQZ; (C) 202 target genes of TSZ; (D) 25 target genes of BJT; (E) 121 target genes of DS; (F) 11 target genes of BZ; (G) 283 target genes of DZ; (H) 13 target genes of SR; (J) 112 target genes of RS; (K) 76 target genes of SJS; (L) 115 target genes of AY; (M) 149 target genes of HSW; (N) 68 target genes of EJ; (P) 46 target genes of LJJ. Supplementary Table 3: POI targets. Supplementary Table 4: compounds and targets attributes of C-T network. Supplementary Table 5: data of compound-target network. Supplementary Table 6: analysed C-T network: (A) active compounds of C-T network and corresponding degrees; (B) targets of C-T network and corresponding degrees. Supplementary Table 7: ZSYTP core PPI network. Supplementary Table 8: gene ontology and KEGG analysis: (A) GO-BP analysis; (B) GO-MF analysis; (C) GO-CC analysis; (D) KEGG pathway enrichment analysis. [file 8215454.f1.zip › 8215454.f1/ST7 ZSYT Core PPI Network.pdf]

annotation.T	bg.annotatio	bg.annotatio	bg.annotatio	bg.annotatio	bg.annotatio	bg.annotation.ID	bg.annotatio	bg.annotatio	bg.annotatio	bg.annotatio	bg.annotatio	bg.annotatio	name	selected	shared name
taxon:9606	176130	1902	nucleolar pro	gene	biological_pr	G:9606:NOL3				NOL3	Homo sapier	Gene Symbo	NOL3	FALSE	NOL3
taxon:9606	167939	1902	ring finger pr	gene	biological_pr	G:9606:RNF34				RNF34	Homo sapier	Gene Symbo	RNF34	FALSE	RNF34
taxon:9606	180225	1902	integrin subu	gene	biological_pr	G:9606:ITGAV			KEGG-&-1&-	ITGAV	Homo sapier	Gene Symbo	ITGAV	FALSE	ITGAV
taxon:9606	176135	1902	selenium bin	gene	biological_pr	G:9606:SELENBP1				SELENBP1	Homo sapier	Gene Symbo	SELENBP1	FALSE	SELENBP1
taxon:9606	180229	1902	integrin subu	gene	biological_pr	G:9606:ITGAD			KEGG-&-1&-	ITGAD	Homo sapier	Gene Symbo	ITGAD	FALSE	ITGAD
taxon:9606	180234	1902	heterogeneo	gene	biological_pr	G:9606:HNRNPH3				HNRNPH3	Homo sapier	Gene Symbo	HNRNPH3	FALSE	HNRNPH3
taxon:9606	180233	1902	heterogeneo	gene	biological_pr	G:9606:HNRNPK			KEGG-&-1&-	HNRNPK	Homo sapier	Gene Symbo	HNRNPK	FALSE	HNRNPK
taxon:9606	180238	1902	heterogeneo	gene	biological_pr	G:9606:HNRNPF				HNRNPF	Homo sapier	Gene Symbo	HNRNPF	FALSE	HNRNPF
taxon:9606	180239	1902	heterogeneo	gene	biological_pr	G:9606:HNRNPD				HNRNPD	Homo sapier	Gene Symbo	HNRNPD	FALSE	HNRNPD
taxon:9606	167948	1902	splA/ryanodi	gene	biological_pr	G:9606:SPSB1				SPSB1	Homo sapier	Gene Symbo	SPSB1	FALSE	SPSB1
taxon:9606	180236	1902	heterogeneo	gene	biological_pr	G:9606:HNRNPH1				HNRNPH1	Homo sapier	Gene Symbo	HNRNPH1	FALSE	HNRNPH1
taxon:9606	180242	1902	heterogeneo	gene	biological_pr	G:9606:HNRNPA2B1				HNRNPA2B1	Homo sapier	Gene Symbo	HNRNPA2B1	FALSE	HNRNPA2B1
taxon:9606	180243	1902	heterogeneo	gene	biological_pr	G:9606:HNRNPA1			KEGG-&-1&-	HNRNPA1	Homo sapier	Gene Symbo	HNRNPA1	FALSE	HNRNPA1
taxon:9606	176147	1902	histone clust	gene	biological_pr	G:9606:HIST1H2BJ			KEGG-&-1&-	HIST1H2BJ	Homo sapier	Gene Symbo	HIST1H2BJ	FALSE	HIST1H2BJ
taxon:9606	172048	1902	copine 7	gene	biological_pr	G:9606:CPNE7				CPNE7	Homo sapier	Gene Symbo	CPNE7	FALSE	CPNE7
taxon:9606	180240	1902	heterogeneo	gene	biological_pr	G:9606:HNRNPC			KEGG-&-1&-	HNRNPC	Homo sapier	Gene Symbo	HNRNPC	FALSE	HNRNPC
taxon:9606	176150	1902	beta-transdu	gene	biological_pr	G:9606:BTRC			KEGG-&-1&-	BTRC	Homo sapier	Gene Symbo	BTRC	FALSE	BTRC
taxon:9606	180246	1902	one cut hom	gene	biological_pr	G:9606:ONECUT1			KEGG-&-1&-	ONECUT1	Homo sapier	Gene Symbo	ONECUT1	FALSE	ONECUT1
taxon:9606	167958	1902	N(alpha)-ace	gene	biological_pr	G:9606:NAA15				NAA15	Homo sapier	Gene Symbo	NAA15	FALSE	NAA15
taxon:9606	176149	1902	histone clust	gene	biological_pr	G:9606:HIST1H3F			KEGG-&-1&-	HIST1H3F	Homo sapier	Gene Symbo	HIST1H3F	FALSE	HIST1H3F
taxon:9606	180251	1902	forkhead box	gene	biological_pr	G:9606:FOXA1				FOXA1	Homo sapier	Gene Symbo	FOXA1	FALSE	FOXA1
taxon:9606	180248	1902	hepatocyte n	gene	biological_pr	G:9606:HNF4A			KEGG-&-1&-	HNF4A	Homo sapier	Gene Symbo	HNF4A	FALSE	HNF4A
taxon:9606	159768	1902	small ubiquit	gene	biological_pr	G:9606:SUMO4			KEGG-&-1&-	SUMO4	Homo sapier	Gene Symbo	SUMO4	FALSE	SUMO4
taxon:9606	180249	1902	forkhead box	gene	biological_pr	G:9606:FOXA3			KEGG-&-1&-	FOXA3	Homo sapier	Gene Symbo	FOXA3	FALSE	FOXA3
taxon:9606	176153	1902	kynureninase	gene	biological_pr	G:9606:KYNU			KEGG-&-1&-	KYNU	Homo sapier	Gene Symbo	KYNU	FALSE	KYNU
taxon:9606	172057	1902	dickkopf WN	gene	biological_pr	G:9606:DKK3				DKK3	Homo sapier	Gene Symbo	DKK3	FALSE	DKK3
taxon:9606	180254	1902	nuclear rece	gene	biological_pr	G:9606:NR4A1			KEGG-&-1&-	NR4A1	Homo sapier	Gene Symbo	NR4A1	FALSE	NR4A1
taxon:9606	167966	1902	THO comple	gene	biological_pr	G:9606:THOC7			KEGG-&-1&-	THOC7	Homo sapier	Gene Symbo	THOC7	FALSE	THOC7
taxon:9606	180255	1902	heme oxyge	gene	biological_pr	G:9606:HMOX2			KEGG-&-1&-	HMOX2	Homo sapier	Gene Symbo	HMOX2	FALSE	HMOX2
taxon:9606	176156	1902	far upstream	gene	biological_pr	G:9606:FUBP3				FUBP3	Homo sapier	Gene Symbo	FUBP3	FALSE	FUBP3
taxon:9606	180258	1902	high mobility	gene	biological_pr	G:9606:HMGA1				HMGA1	Homo sapier	Gene Symbo	HMGA1	FALSE	HMGA1
taxon:9606	176167	1902	SNRPN upstr	gene	biological_pr	G:9606:SNURF				SNURF	Homo sapier	Gene Symbo	SNURF	FALSE	SNURF
taxon:9606	172071	1902	TATA-box bin	gene	biological_pr	G:9606:TAF5L			KEGG-&-1&-	TAF5L	Homo sapier	Gene Symbo	TAF5L	FALSE	TAF5L
taxon:9606	127012	1902	BOLA2-SMG:	gene		G:9606:BOLA2-SMG1P6				BOLA2-SMG:	Homo sapier	Gene Symbo	BOLA2-SMG:	FALSE	BOLA2-SMG1P6
taxon:9606	172068	1902	calcyclin bin	gene	biological_pr	G:9606:CACYBP			KEGG-&-1&-	CACYBP	Homo sapier	Gene Symbo	CACYBP	FALSE	CACYBP
taxon:9606	176165	1902	forkhead box	gene	biological_pr	G:9606:FOXH1				FOXH1	Homo sapier	Gene Symbo	FOXH1	FALSE	FOXH1
taxon:9606	172072	1902	trafficking pr	gene	biological_pr	G:9606:TRAPPC3				TRAPPC3	Homo sapier	Gene Symbo	TRAPPC3	FALSE	TRAPPC3
taxon:9606	176169	1902	HECT and RL	gene	biological_pr	G:9606:HERC2			KEGG-&-1&-	HERC2	Homo sapier	Gene Symbo	HERC2	FALSE	HERC2
taxon:9606	180268	1902	high mobility	gene	biological_pr	G:9606:HMGB2				HMGB2	Homo sapier	Gene Symbo	HMGB2	FALSE	HMGB2
taxon:9606	180269	1902	high mobility	gene	biological_pr	G:9606:HMGB1			KEGG-&-1&-	HMGB1	Homo sapier	Gene Symbo	HMGB1	FALSE	HMGB1
taxon:9606	172080	1902	forkhead box	gene	biological_pr	G:9606:FOXP1			KEGG-&-1&-	FOXP1	Homo sapier	Gene Symbo	FOXP1	FALSE	FOXP1
taxon:9606	167988	1902	mitotic spind	gene	cellular_com	G:9606:MZT2B				MZT2B	Homo sapier	Gene Symbo	MZT2B	FALSE	MZT2B
taxon:9606	172094	1902	staphylococ	gene	biological_pr	G:9606:SND1			KEGG-&-1&-	SND1	Homo sapier	Gene Symbo	SND1	FALSE	SND1
taxon:9606	176191	1902	eukaryotic tr	gene	biological_pr	G:9606:EIF2S2			KEGG-&-1&-	EIF2S2	Homo sapier	Gene Symbo	EIF2S2	FALSE	EIF2S2
taxon:9606	172095	1902	proline, gluta	gene	biological_pr	G:9606:PELP1				PELP1	Homo sapier	Gene Symbo	PELP1	FALSE	PELP1
taxon:9606	172107	1902	mutL homolo	gene	biological_pr	G:9606:MLH3			KEGG-&-1&-	MLH3	Homo sapier	Gene Symbo	MLH3	FALSE	MLH3
taxon:9606	176203	1902	far upstream	gene	biological_pr	G:9606:FUBP1				FUBP1	Homo sapier	Gene Symbo	FUBP1	FALSE	FUBP1
taxon:9606	172104	1902	zinc finger ar	gene	biological_pr	G:9606:ZBTB32				ZBTB32	Homo sapier	Gene Symbo	ZBTB32	FALSE	ZBTB32
taxon:9606	172108	1902	forkhead box	gene	biological_pr	G:9606:FOXB1				FOXB1	Homo sapier	Gene Symbo	FOXB1	FALSE	FOXB1
taxon:9606	176205	1902	sequestosom	gene	biological_pr	G:9606:SQSTM1			KEGG-&-1&-	SQSTM1	Homo sapier	Gene Symbo	SQSTM1	FALSE	SQSTM1
taxon:9606	176215	1902	period circad	gene	biological_pr	G:9606:PER2			KEGG-&-1&-	PER2	Homo sapier	Gene Symbo	PER2	FALSE	PER2
taxon:9606	163930	1902	PDLIM1 inter	gene	biological_pr	G:9606:PDIK1L				PDIK1L	Homo sapier	Gene Symbo	PDIK1L	FALSE	PDIK1L
taxon:9606	180315	1902	ubiquitin con	gene	biological_pr	G:9606:UBE2K			KEGG-&-1&-	UBE2K	Homo sapier	Gene Symbo	UBE2K	FALSE	UBE2K
taxon:9606	176222	1902	nuclear rece	gene	biological_pr	G:9606:NR1I2				NR1I2	Homo sapier	Gene Symbo	NR1I2	FALSE	NR1I2
taxon:9606	180318	1902	HIC ZBTB tra	gene	biological_pr	G:9606:HIC1				HIC1	Homo sapier	Gene Symbo	HIC1	FALSE	HIC1
taxon:9606	172127	1902	poly(A) bindi	gene	biological_pr	G:9606:PABPC1			KEGG-&-1&-	PABPC1	Homo sapier	Gene Symbo	PABPC1	FALSE	PABPC1
taxon:9606	180317	1902	hypoxia indu	gene	biological_pr	G:9606:HIF1A			KEGG-&-1&-	HIF1A	Homo sapier	Gene Symbo	HIF1A	FALSE	HIF1A
taxon:9606	176227	1902	lysine acetylt	gene	biological_pr	G:9606:KAT2B			KEGG-&-1&-	KAT2B	Homo sapier	Gene Symbo	KAT2B	FALSE	KAT2B
taxon:9606	176228	1902	TSC22 domai	gene	biological_pr	G:9606:TSC22D1				TSC22D1	Homo sapier	Gene Symbo	TSC22D1	FALSE	TSC22D1
taxon:9606	176234	1902	histone deac	gene	biological_pr	G:9606:HDAC3			KEGG-&-1&-	HDAC3	Homo sapier	Gene Symbo	HDAC3	FALSE	HDAC3
taxon:9606	176238	1902	CASP8 and F	gene	biological_pr	G:9606:CFLAR			KEGG-&-1&-	CFLAR	Homo sapier	Gene Symbo	CFLAR	FALSE	CFLAR
taxon:9606	180335	1902	histone deac	gene	biological_pr	G:9606:HDAC2			KEGG-&-1&-	HDAC2	Homo sapier	Gene Symbo	HDAC2	FALSE	HDAC2
taxon:9606	180333	1902	hepatoma de	gene	biological_pr	G:9606:HDGF				HDGF	Homo sapier	Gene Symbo	HDGF	FALSE	HDGF
taxon:9606	163949	1902	ring finger ar	gene	biological_pr	G:9606:RC3H1				RC3H1	Homo sapier	Gene Symbo	RC3H1	FALSE	RC3H1
taxon:9606	176242	1902	guanine mon	gene	biological_pr	G:9606:GMPS			KEGG-&-1&-	GMPS	Homo sapier	Gene Symbo	GMPS	FALSE	GMPS
taxon:9606	180336	1902	histone deac	gene	biological_pr	G:9606:HDAC1			KEGG-&-1&-	HDAC1	Homo sapier	Gene Symbo	HDAC1	FALSE	HDAC1
taxon:9606	180337	1902	huntingtin	gene	biological_pr	G:9606:HTT			KEGG-&-1&-	HTT	Homo sapier	Gene Symbo	HTT	FALSE	HTT
taxon:9606	176241	1902	transmembr	gene	biological_pr	G:9606:TMEM11				TMEM11	Homo sapier	Gene Symbo	TMEM11	FALSE	TMEM11
taxon:9606	168054	1902	mitochondri	gene	biological_pr	G:9606:MRPL24			KEGG-&-1&-	MRPL24	Homo sapier	Gene Symbo	MRPL24	FALSE	MRPL24
taxon:9606	176246	1902	neuropilin 1	gene	biological_pr	G:9606:NRP1			KEGG-&-1&-	NRP1	Homo sapier	Gene Symbo	NRP1	FALSE	NRP1
taxon:9606	168055	1902	ring finger pr	gene	biological_pr	G:9606:RNF128				RNF128	Homo sapier	Gene Symbo	RNF128	FALSE	RNF128
taxon:9606	176247	1902	neuropilin 2	gene	biological_pr	G:9606:NRP2				NRP2	Homo sapier	Gene Symbo	NRP2	FALSE	NRP2
taxon:9606	168052	1902	mitochondri	gene	biological_pr	G:9606:MUL1				MUL1	Homo sapier	Gene Symbo	MUL1	FALSE	MUL1
taxon:9606	180346	1902	host cell fact	gene	biological_pr	G:9606:HCFC1			KEGG-&-1&-	HCFC1	Homo sapier	Gene Symbo	HCFC1	FALSE	HCFC1
taxon:9606	176248	1902	IQ motif cont	gene	biological_pr	G:9606:IQGAP1			KEGG-&-1&-	IQGAP1	Homo sapier	Gene Symbo	IQGAP1	FALSE	IQGAP1
taxon:9606	180345	1902	HCK proto-on	gene	biological_pr	G:9606:HCK			KEGG-&-1&-	HCK	Homo sapier	Gene Symbo	HCK	FALSE	HCK
taxon:9606	180350	1902	hemoglobin	gene	biological_pr	G:9606:HBZ				HBZ	Homo sapier	Gene Symbo	HBZ	FALSE	HBZ
taxon:9606	180354	1902	hemoglobin	gene	biological_pr	G:9606:HBE1				HBE1	Homo sapier	Gene Symbo	HBE1	FALSE	HBE1
taxon:9606	180355	1902	hemoglobin	gene	biological_pr	G:9606:HBD				HBD	Homo sapier	Gene Symbo	HBD	FALSE	HBD
taxon:9606	176259	1902	barrier to aut	gene	biological_pr	G:9606:BANF1				BANF1	Homo sapier	Gene Symbo	BANF1	FALSE	BANF1
taxon:9606	180352	1902	hemoglobin	gene	biological_pr	G:9606:HBG2				HBG2	Homo sapier	Gene Symbo	HBG2	FALSE	HBG2
taxon:9606	180353	1902	hemoglobin	gene	biological_pr	G:9606:HBG1				HBG1	Homo sapier	Gene Symbo	HBG1	FALSE	HBG1
taxon:9606	176260	1902	cyclin depen	gene	biological_pr	G:9606:CDKL1				CDKL1	Homo sapier	Gene Symbo	CDKL1	FALSE	CDKL1
taxon:9606	139396	1902	U2 small nuc	gene	biological_pr	G:9606:U2AF1L5			KEGG-&-1&-	U2AF1L5	Homo sapier	Gene Symbo	U2AF1L5	FALSE	U2AF1L5
taxon:9606	180360	1902	hemoglobin	gene	biological_pr	G:9606:HBA2			KEGG-&-1&-	HBA2	Homo sapier	Gene Symbo	HBA2	FALSE	HBA2
taxon:9606	180361	1902	hemoglobin	gene	biological_pr	G:9606:HBA1			KEGG-&-1&-	HBA1	Homo sapier	Gene Symbo	HBA1	FALSE	HBA1
taxon:9606	176268	1902	tripartite mo	gene	biological_pr	G:9606:TRIM24				TRIM24	Homo sapier	Gene Symbo	TRIM24	FALSE	TRIM24
taxon:9606	180368	1902	hydroxyacyl-C	gene	biological_pr	G:9606:HADHB			KEGG-&-1&-	HADHB	Homo sapier	Gene Symbo	HADHB	FALSE	HADHB
taxon:9606	180374	1902	histone clust	gene	biological_pr	G:9606:HIST1H1A				HIST1H1A	Homo sapier	Gene Symbo	HIST1H1A	FALSE	HIST1H1A
taxon:9606	180375	1902	H3 histone fa	gene	biological_pr	G:9606:H3F3B			KEGG-&-1&-	H3F3B	Homo sapier	Gene Symbo	H3F3B	FALSE	H3F3B
taxon:9606	180378	1902	histone clust	gene	biological_pr	G:9606:HIST1H2BD			KEGG-&-1&-	HIST1H2BD	Homo sapier	Gene Symbo	HIST1H2BD	FALSE	HIST1H2BD
taxon:9606	180376	1902	H3 histone fa	gene	biological_pr	G:9606:H3F3A			KEGG-&-1&-	H3F3A	Homo sapier	Gene Symbo	H3F3A	FALSE	H3F3A
taxon:9606	180380	1902	H2A histone	gene	biological_pr	G:9606:H2AFX			KEGG-&-1&-	H2AFX	Homo sapier	Gene Symbo	H2AFX	FALSE	H2AFX
taxon:9606	180381	1902	histone clust	gene	biological_pr	G:9606:HIST1H2AD			KEGG-&-1&-	HIST1H2AD	Homo sapier	Gene Symbo	HIST1H2AD	FALSE	HIST1H2AD
taxon:9606	180386	1902	histone clust	gene	biological_pr	G:9606:HIST1H1D				HIST1H1D	Homo sapier	Gene Symbo	HIST1H1D	FALSE	HIST1H1D
taxon:9606	180387	1902	histone clust	gene	biological_pr	G:9606:HIST1H1C				HIST1H1C	Homo sapier	Gene Symbo	HIST1H1C	FALSE	HIST1H1C
taxon:9606	180384	1902	histone clust	gene	biological_pr	G:9606:HIST1H1B				HIST1H1B	Homo sapier	Gene Symbo	HIST1H1B	FALSE	HIST1H1B
taxon:9606	180391	1902	granzyme B	gene	biological_pr	G:9606:GZMB			KEGG-&-1&-	GZMB	Homo sapier	Gene Symbo	GZMB	FALSE	GZMB
taxon:9606	176295	1902	NSF attachm	gene	biological_pr	G:9606:NAPA			KEGG-&-1&-	NAPA	Homo sapier	Gene Symbo	NAPA	FALSE	NAPA
taxon:9606	176293	1902	multiple PDZ	gene	biological_pr	G:9606:MPDZ			KEGG-&-1&-	MPDZ	Homo sapier	Gene Symbo	MPDZ	FALSE	MPDZ
taxon:9606	180389	1902	granzyme M	gene	biological_pr	G:9606:GZMM				GZMM	Homo sapier	Gene Symbo	GZMM	FALSE	GZMM
taxon:9606	168113	1902	PAXIP1 asso	gene	biological_pr	G:9606:PAGR1				PAGR1	Homo sapier	Gene Symbo	PAGR1	FALSE	PAGR1
taxon:9606	164023	1902	zinc finger pr	gene	biological_pr	G:9606:ZNF420				ZNF420	Homo sapier	Gene Symbo	ZNF420	FALSE	ZNF420
taxon:9606	180408	1902	guanylate cy	gene	biological_pr	G:9606:GUCY1B3			KEGG-&-1&-	GUCY1B3	Homo sapier	Gene Symbo	GUCY1B3	FALSE	GUCY1B3
taxon:9606	176313	1902	axin 1	gene	biological_pr	G:9606:AXIN1			KEGG-&-1&-	AXIN1	Homo sapier	Gene Symbo	AXIN1	FALSE	AXIN1
taxon:9606	180409	1902	guanylate cy	gene	biological_pr	G:9606:GUCY1A3			KEGG-&-1&-	GUCY1A3	Homo sapier	Gene Symbo	GUCY1A3	FALSE	GUCY1A3
taxon:9606	176318	1902	phosphatidyl	gene	biological_pr	G:9606:PICALM				PICALM	Homo sapier	Gene Symbo	PICALM	FALSE	PICALM
taxon:9606	176319	1902	transformati	gene	biological_pr	G:9606:TRRAP			KEGG-&-1&-	TRRAP	Homo sapier	Gene Symbo	TRRAP	FALSE	TRRAP
taxon:9606	168130	1902	basic helix-lo	gene	biological_pr	G:9606:BHLHE41			KEGG-&-1&-	BHLHE41	Homo sapier	Gene Symbo	BHLHE41	FALSE	BHLHE41
taxon:9606	176320	1902	histone clust	gene	biological_pr	G:9606:HIST1H4I			KEGG-&-1&-	HIST1H4I	Homo sapier	Gene Symbo	HIST1H4I	FALSE	HIST1H4I
taxon:9606	176327	1902	ubiquitin spe	gene	biological_pr	G:9606:USP9Y				USP9Y	Homo sapier	Gene Symbo	USP9Y	FALSE	USP9Y
taxon:9606	176324	1902	histone clust	gene	biological_pr	G:9606:HIST3H3			KEGG-&-1&-	HIST3H3	Homo sapier	Gene Symbo	HIST3H3	FALSE	HIST3H3
taxon:9606	176325	1902	AT-rich inter	gene	biological_pr	G:9606:ARID1A			KEGG-&-1&-	ARID1A	Homo sapier	Gene Symbo	ARID1A	FALSE	ARID1A
taxon:9606	180446	1902	glypican 1	gene	biological_pr	G:9606:GPC1			KEGG-&-1&-	GPC1	Homo sapier	Gene Symbo	GPC1	FALSE	GPC1
taxon:9606	176348	1902	ubiquitin spe	gene	biological_pr	G:9606:USP9X				USP9X	Homo sapier	Gene Symbo	USP9X	FALSE	USP9X
taxon:9606	176349	1902	ubiquitin spe	gene	biological_pr	G:9606:USP11				USP11	Homo sapier	Gene Symbo	USP11	FALSE	USP11
taxon:9606	172256	1902	G protein nu	gene	biological_pr	G:9606:GNL3			KEGG-&-1&-	GNL3	Homo sapier	Gene Symbo	GNL3	FALSE	GNL3
taxon:9606	176358	1902	DiGeorge syr	gene	biological_pr	G:9606:DGCR14				DGCR14	Homo sapier	Gene Symbo	DGCR14	FALSE	DGCR14
taxon:9606	176359	1902	clathrin heav	gene	biological_pr	G:9606:CLTCL1			KEGG-&-1&-	CLTCL1	Homo sapier	Gene Symbo	CLTCL1	FALSE	CLTCL1
taxon:9606	176361	1902	leucine zippe	gene	biological_pr	G:9606:LZTR1				LZTR1	Homo sapier	Gene Symbo	LZTR1	FALSE	LZTR1
taxon:9606	176370	1902	nuclear rece	gene	biological_pr	G:9606:NCOA3			KEGG-&-1&-	NCOA3	Homo sapier	Gene Symbo	NCOA3	FALSE	NCOA3
taxon:9606	180466	1902	isoleucyl-tRN	gene	biological_pr	G:9606:IARS			KEGG-&-1&-	IARS	Homo sapier	Gene Symbo	IARS	FALSE	IARS
taxon:9606	176369	1902	nuclear rece	gene	biological_pr	G:9606:NRIP1				NRIP1	Homo sapier	Gene Symbo	NRIP1	FALSE	NRIP1
taxon:9606	168182	1902	BRCA1/BRCA	gene	biological_pr	G:9606:BRCC3			KEGG-&-1&-	BRCC3	Homo sapier	Gene Symbo	BRCC3	FALSE	BRCC3
taxon:9606	168180	1902	transmembr	gene	biological_pr	G:9606:TMEM43				TMEM43	Homo sapier	Gene Symbo	TMEM43	FALSE	TMEM43
taxon:9606	176382	1902	elongation fa	gene	biological_pr	G:9606:ELL				ELL	Homo sapier	Gene Symbo	ELL	FALSE	ELL
taxon:9606	180479	1902	5-hydroxytryp	gene	biological_pr	G:9606:HTR2B			KEGG-&-1&-	HTR2B	Homo sapier	Gene Symbo	HTR2B	FALSE	HTR2B
taxon:9606	127233	1902	bolA-like pro	gene		G:9606:LOC107984053				LOC10798405	Homo sapier	Gene Symbo	LOC10798405	FALSE	LOC107984053
taxon:9606	164096	1902	spindle and c	gene	biological_pr	G:9606:SPICE1				SPICE1	Homo sapier	Gene Symbo	SPICE1	FALSE	SPICE1
taxon:9606	164110	1902	ring finger pr	gene	biological_pr	G:9606:RNF38				RNF38	Homo sapier	Gene Symbo	RNF38	FALSE	RNF38
taxon:9606	180499	1902	heat shock pr	gene	biological_pr	G:9606:HSPD1			KEGG-&-1&-	HSPD1	Homo sapier	Gene Symbo	HSPD1	FALSE	HSPD1
taxon:9606	180496	1902	DnaJ heat sh	gene	biological_pr	G:9606:DNAJB1			KEGG-&-1&-	DNAJB1	Homo sapier	Gene Symbo	DNAJB1	FALSE	DNAJB1
taxon:9606	180497	1902	heat shock pr	gene	biological_pr	G:9606:HSPE1				HSPE1	Homo sapier	Gene Symbo	HSPE1	FALSE	HSPE1
taxon:9606	176404	1902	NPR3 like, GA	gene	biological_pr	G:9606:NPRL3			KEGG-&-1&-	NPRL3	Homo sapier	Gene Symbo	NPRL3	FALSE	NPRL3
taxon:9606	180501	1902	heat shock pr	gene	biological_pr	G:9606:HSP90AB1			KEGG-&-1&-	HSP90AB1	Homo sapier	Gene Symbo	HSP90AB1	FALSE	HSP90AB1
taxon:9606	180506	1902	heat shock pr	gene	biological_pr	G:9606:HSP90AA1			KEGG-&-1&-	HSP90AA1	Homo sapier	Gene Symbo	HSP90AA1	FALSE	HSP90AA1
taxon:9606	180507	1902	heat shock pr	gene	biological_pr	G:9606:HSPB2			KEGG-&-1&-	HSPB2	Homo sapier	Gene Symbo	HSPB2	FALSE	HSPB2
taxon:9606	176409	1902	acidic nuclea	gene	biological_pr	G:9606:ANP32A				ANP32A	Homo sapier	Gene Symbo	ANP32A	FALSE	ANP32A
taxon:9606	180510	1902	heat shock pr	gene	biological_pr	G:9606:HSPA9			KEGG-&-1&-	HSPA9	Homo sapier	Gene Symbo	HSPA9	FALSE	HSPA9
taxon:9606	180511	1902	heat shock pr	gene	biological_pr	G:9606:HSPA8			KEGG-&-1&-	HSPA8	Homo sapier	Gene Symbo	HSPA8	FALSE	HSPA8
taxon:9606	168223	1902	small ArfGA	gene	biological_pr	G:9606:SMAP2			KEGG-&-1&-	SMAP2	Homo sapier	Gene Symbo	SMAP2	FALSE	SMAP2
taxon:9606	180508	1902	heat shock pr	gene	biological_pr	G:9606:HSPB1			KEGG-&-1&-	HSPB1	Homo sapier	Gene Symbo	HSPB1	FALSE	HSPB1
taxon:9606	180514	1902	heat shock pr	gene	biological_pr	G:9606:HSPA5			KEGG-&-1&-	HSPA5	Homo sapier	Gene Symbo	HSPA5	FALSE	HSPA5
taxon:9606	180515	1902	heat shock pr	gene	biological_pr	G:9606:HSPA4			KEGG-&-1&-	HSPA4	Homo sapier	Gene Symbo	HSPA4	FALSE	HSPA4
taxon:9606	180513	1902	heat shock pr	gene	biological_pr	G:9606:HSPA6			KEGG-&-1&-	HSPA6	Homo sapier	Gene Symbo	HSPA6	FALSE	HSPA6
taxon:9606	180518	1902	heat shock pr	gene	biological_pr	G:9606:HSPA1B			KEGG-&-1&-	HSPA1B	Homo sapier	Gene Symbo	HSPA1B	FALSE	HSPA1B
taxon:9606	180519	1902	heat shock pr	gene	biological_pr	G:9606:HSPA1A			KEGG-&-1&-	HSPA1A	Homo sapier	Gene Symbo	HSPA1A	FALSE	HSPA1A
taxon:9606	160039	1902	natural killer	gene	biological_pr	G:9606:NCR3LG1				NCR3LG1	Homo sapier	Gene Symbo	NCR3LG1	FALSE	NCR3LG1
taxon:9606	180516	1902	heat shock pr	gene	biological_pr	G:9606:HSPA2			KEGG-&-1&-	HSPA2	Homo sapier	Gene Symbo	HSPA2	FALSE	HSPA2
taxon:9606	180517	1902	heat shock pr	gene	biological_pr	G:9606:HSPA1L			KEGG-&-1&-	HSPA1L	Homo sapier	Gene Symbo	HSPA1L	FALSE	HSPA1L
taxon:9606	164133	1902	deltex E3 ubi	gene	biological_pr	G:9606:DTX3L			KEGG-&-1&-	DTX3L	Homo sapier	Gene Symbo	DTX3L	FALSE	DTX3L
taxon:9606	180520	1902	DnaJ heat sh	gene	biological_pr	G:9606:DNAJA1			KEGG-&-1&-	DNAJA1	Homo sapier	Gene Symbo	DNAJA1	FALSE	DNAJA1
taxon:9606	180524	1902	heat shock tr	gene	biological_pr	G:9606:HSF1			KEGG-&-1&-	HSF1	Homo sapier	Gene Symbo	HSF1	FALSE	HSF1
taxon:9606	176434	1902	lysine methyl	gene	biological_pr	G:9606:KMT2D			KEGG-&-1&-	KMT2D	Homo sapier	Gene Symbo	KMT2D	FALSE	KMT2D
taxon:9606	180534	1902	hes family b	gene	biological_pr	G:9606:HES1			KEGG-&-1&-	HES1	Homo sapier	Gene Symbo	HES1	FALSE	HES1
taxon:9606	180535	1902	protein argin	gene	biological_pr	G:9606:PRMT1			KEGG-&-1&-	PRMT1	Homo sapier	Gene Symbo	PRMT1	FALSE	PRMT1
taxon:9606	180536	1902	protein argin	gene	biological_pr	G:9606:PRMT2				PRMT2	Homo sapier	Gene Symbo	PRMT2	FALSE	PRMT2
taxon:9606	168249	1902	transducin be	gene	biological_pr	G:9606:TBL1XR1			KEGG-&-1&-	TBL1XR1	Homo sapier	Gene Symbo	TBL1XR1	FALSE	TBL1XR1
taxon:9606	180543	1902	ArfGAP with	gene	biological_pr	G:9606:AGFG1			KEGG-&-1&-	AGFG1	Homo sapier	Gene Symbo	AGFG1	FALSE	AGFG1
taxon:9606	168252	1902	coiled-coil do	gene	cellular_com	G:9606:CCDC51				CCDC51	Homo sapier	Gene Symbo	CCDC51	FALSE	CCDC51
taxon:9606	176459	1902	nuclear rece	gene	biological_pr	G:9606:NCOA4			KEGG-&-1&-	NCOA4	Homo sapier	Gene Symbo	NCOA4	FALSE	NCOA4
taxon:9606	176460	1902	coiled-coil do	gene	biological_pr	G:9606:CCDC6			KEGG-&-1&-	CCDC6	Homo sapier	Gene Symbo	CCDC6	FALSE	CCDC6
taxon:9606	176469	1902	bromodomai	gene	biological_pr	G:9606:BRD3				BRD3	Homo sapier	Gene Symbo	BRD3	FALSE	BRD3
taxon:9606	176475	1902	lysine acetylt	gene	biological_pr	G:9606:KAT6A			KEGG-&-1&-	KAT6A	Homo sapier	Gene Symbo	KAT6A	FALSE	KAT6A
taxon:9606	164194	1902	copper metal	gene	biological_pr	G:9606:COMMD1				COMMD1	Homo sapier	Gene Symbo	COMMD1	FALSE	COMMD1
taxon:9606	160096	1902	lysine methyl	gene	biological_pr	G:9606:KMT5A			KEGG-&-1&-	KMT5A	Homo sapier	Gene Symbo	KMT5A	FALSE	KMT5A
taxon:9606	180582	1902	stratifin	gene	biological_pr	G:9606:SFN			KEGG-&-1&-	SFN	Homo sapier	Gene Symbo	SFN	FALSE	SFN
taxon:9606	168299	1902	testis specifi	gene	biological_pr	G:9606:TSPY2				TSPY2	Homo sapier	Gene Symbo	TSPY2	FALSE	TSPY2
taxon:9606	180588	1902	golgin A2	gene	biological_pr	G:9606:GOLGA2				GOLGA2	Homo sapier	Gene Symbo	GOLGA2	FALSE	GOLGA2
taxon:9606	176493	1902	zinc finger pr	gene	biological_pr	G:9606:ZNF24				ZNF24	Homo sapier	Gene Symbo	ZNF24	FALSE	ZNF24
taxon:9606	176506	1902	CCHC-type zi	gene	biological_pr	G:9606:CNBP				CNBP	Homo sapier	Gene Symbo	CNBP	FALSE	CNBP
taxon:9606	168313	1902	mitochondri	gene	biological_pr	G:9606:MRPS25				MRPS25	Homo sapier	Gene Symbo	MRPS25	FALSE	MRPS25
taxon:9606	180606	1902	G protein su	gene	biological_pr	G:9606:GNB1			KEGG-&-1&-	GNB1	Homo sapier	Gene Symbo	GNB1	FALSE	GNB1
taxon:9606	180605	1902	G protein su	gene	biological_pr	G:9606:GNB2			KEGG-&-1&-	GNB2	Homo sapier	Gene Symbo	GNB2	FALSE	GNB2
taxon:9606	176514	1902	ribosomal pr	gene	biological_pr	G:9606:RPL14			KEGG-&-1&-	RPL14	Homo sapier	Gene Symbo	RPL14	FALSE	RPL14
taxon:9606	176512	1902	Zic family m	gene	biological_pr	G:9606:ZIC3			KEGG-&-1&-	ZIC3	Homo sapier	Gene Symbo	ZIC3	FALSE	ZIC3
taxon:9606	180612	1902	G protein su	gene	biological_pr	G:9606:GNAQ			KEGG-&-1&-	GNAQ	Homo sapier	Gene Symbo	GNAQ	FALSE	GNAQ
taxon:9606	176517	1902	ubiquitin con	gene	biological_pr	G:9606:UBE2M			KEGG-&-1&-	UBE2M	Homo sapier	Gene Symbo	UBE2M	FALSE	UBE2M
taxon:9606	180617	1902	G protein su	gene	biological_pr	G:9606:GNAI2			KEGG-&-1&-	GNAI2	Homo sapier	Gene Symbo	GNAI2	FALSE	GNAI2
taxon:9606	176527	1902	phosphoinosi	gene	biological_pr	G:9606:PIK3R3			KEGG-&-1&-	PIK3R3	Homo sapier	Gene Symbo	PIK3R3	FALSE	PIK3R3
taxon:9606	180621	1902	G protein su	gene	biological_pr	G:9606:GNA11			KEGG-&-1&-	GNA11	Homo sapier	Gene Symbo	GNA11	FALSE	GNA11
taxon:9606	176525	1902	poly(ADP-rib	gene	biological_pr	G:9606:PARG				PARG	Homo sapier	Gene Symbo	PARG	FALSE	PARG
taxon:9606	176534	1902	PPFIA bindin	gene	biological_pr	G:9606:PPFIBP1				PPFIBP1	Homo sapier	Gene Symbo	PPFIBP1	FALSE	PPFIBP1
taxon:9606	168343	1902	matrix metal	gene	biological_pr	G:9606:MMP25			KEGG-&-1&-	MMP25	Homo sapier	Gene Symbo	MMP25	FALSE	MMP25
taxon:9606	168341	1902	zinc finger m	gene	biological_pr	G:9606:ZMAT3			KEGG-&-1&-	ZMAT3	Homo sapier	Gene Symbo	ZMAT3	FALSE	ZMAT3
taxon:9606	176536	1902	protein phos	gene	biological_pr	G:9606:PPM1D			KEGG-&-1&-	PPM1D	Homo sapier	Gene Symbo	PPM1D	FALSE	PPM1D
taxon:9606	176546	1902	OFD1, centri	gene	biological_pr	G:9606:OFD1				OFD1	Homo sapier	Gene Symbo	OFD1	FALSE	OFD1
taxon:9606	176547	1902	ribonucleic a	gene	biological_pr	G:9606:RAE1			KEGG-&-1&-	RAE1	Homo sapier	Gene Symbo	RAE1	FALSE	RAE1
taxon:9606	180640	1902	glutaminase	gene	biological_pr	G:9606:GLS			KEGG-&-1&-	GLS	Homo sapier	Gene Symbo	GLS	FALSE	GLS
taxon:9606	168356	1902	ring finger ar	gene	biological_pr	G:9606:RFWD2			KEGG-&-1&-	RFWD2	Homo sapier	Gene Symbo	RFWD2	FALSE	RFWD2
taxon:9606	168357	1902	nuclear rece	gene	biological_pr	G:9606:NSD1			KEGG-&-1&-	NSD1	Homo sapier	Gene Symbo	NSD1	FALSE	NSD1
taxon:9606	176555	1902	SWI/SNF rel	gene	biological_pr	G:9606:SMARCA5				SMARCA5	Homo sapier	Gene Symbo	SMARCA5	FALSE	SMARCA5
taxon:9606	176552	1902	insulin recep	gene	biological_pr	G:9606:IRS4			KEGG-&-1&-	IRS4	Homo sapier	Gene Symbo	IRS4	FALSE	IRS4
taxon:9606	180649	1902	GLI family zi	gene	biological_pr	G:9606:GLI1			KEGG-&-1&-	GLI1	Homo sapier	Gene Symbo	GLI1	FALSE	GLI1
taxon:9606	176558	1902	TEA domain	gene	biological_pr	G:9606:TEAD2			KEGG-&-1&-	TEAD2	Homo sapier	Gene Symbo	TEAD2	FALSE	TEAD2
taxon:9606	176557	1902	SPT3 homolo	gene	biological_pr	G:9606:SUPT3H			KEGG-&-1&-	SUPT3H	Homo sapier	Gene Symbo	SUPT3H	FALSE	SUPT3H
taxon:9606	176563	1902	forkhead box	gene	biological_pr	G:9606:FOXN1				FOXN1	Homo sapier	Gene Symbo	FOXN1	FALSE	FOXN1
taxon:9606	168371	1902	membrane s	gene	cellular_com	G:9606:MS4A5				MS4A5	Homo sapier	Gene Symbo	MS4A5	FALSE	MS4A5
taxon:9606	176566	1902	cullin 2	gene	biological_pr	G:9606:CUL2			KEGG-&-1&-	CUL2	Homo sapier	Gene Symbo	CUL2	FALSE	CUL2
taxon:9606	164279	1902	centrosomal	gene	cellular_com	G:9606:CEP128				CEP128	Homo sapier	Gene Symbo	CEP128	FALSE	CEP128
taxon:9606	180667	1902	glycerol kina	gene	biological_pr	G:9606:GK			KEGG-&-1&-	GK	Homo sapier	Gene Symbo	GK	FALSE	GK
taxon:9606	176568	1902	cullin 4A	gene	biological_pr	G:9606:CUL4A			KEGG-&-1&-	CUL4A	Homo sapier	Gene Symbo	CUL4A	FALSE	CUL4A
taxon:9606	176569	1902	cullin 4B	gene	biological_pr	G:9606:CUL4B			KEGG-&-1&-	CUL4B	Homo sapier	Gene Symbo	CUL4B	FALSE	CUL4B
taxon:9606	176575	1902	dual specifici	gene	biological_pr	G:9606:DYRK3				DYRK3	Homo sapier	Gene Symbo	DYRK3	FALSE	DYRK3
taxon:9606	168384	1902	MMS19 hom	gene	biological_pr	G:9606:MMS19				MMS19	Homo sapier	Gene Symbo	MMS19	FALSE	MMS19
taxon:9606	176583	1902	reversion ind	gene	biological_pr	G:9606:RECK			KEGG-&-1&-	RECK	Homo sapier	Gene Symbo	RECK	FALSE	RECK
taxon:9606	168395	1902	non-SMC con	gene	biological_pr	G:9606:NCAPG				NCAPG	Homo sapier	Gene Symbo	NCAPG	FALSE	NCAPG
taxon:9606	176585	1902	nuclear rece	gene	biological_pr	G:9606:NR0B2			KEGG-&-1&-	NR0B2	Homo sapier	Gene Symbo	NR0B2	FALSE	NR0B2
taxon:9606	164307	1902	heterogeneo	gene	biological_pr	G:9606:HNRNPA1L2			KEGG-&-1&-	HNRNPA1L2	Homo sapier	Gene Symbo	HNRNPA1L2	FALSE	HNRNPA1L2
taxon:9606	180689	1902	beta-1,4-gala	gene	biological_pr	G:9606:B4GALT1			KEGG-&-1&-	B4GALT1	Homo sapier	Gene Symbo	B4GALT1	FALSE	B4GALT1
taxon:9606	176599	1902	ubiquitously	gene	biological_pr	G:9606:UXT				UXT	Homo sapier	Gene Symbo	UXT	FALSE	UXT
taxon:9606	176597	1902	early endoso	gene	biological_pr	G:9606:EEA1			KEGG-&-1&-	EEA1	Homo sapier	Gene Symbo	EEA1	FALSE	EEA1
taxon:9606	180698	1902	growth facto	gene	biological_pr	G:9606:GFI1				GFI1	Homo sapier	Gene Symbo	GFI1	FALSE	GFI1
taxon:9606	176603	1902	speckle type	gene	biological_pr	G:9606:SPOP			KEGG-&-1&-	SPOP	Homo sapier	Gene Symbo	SPOP	FALSE	SPOP
taxon:9606	176601	1902	transgelin 2	gene	biological_pr	G:9606:TAGLN2				TAGLN2	Homo sapier	Gene Symbo	TAGLN2	FALSE	TAGLN2
taxon:9606	176604	1902	SPARC like 1	gene	biological_pr	G:9606:SPARCL1				SPARCL1	Homo sapier	Gene Symbo	SPARCL1	FALSE	SPARCL1
taxon:9606	176613	1902	phosphatidyl	gene	biological_pr	G:9606:PIP5K1A			KEGG-&-1&-	PIP5K1A	Homo sapier	Gene Symbo	PIP5K1A	FALSE	PIP5K1A
taxon:9606	180717	1902	lysine acetylt	gene	biological_pr	G:9606:KAT2A			KEGG-&-1&-	KAT2A	Homo sapier	Gene Symbo	KAT2A	FALSE	KAT2A
taxon:9606	176631	1902	histone clust	gene	biological_pr	G:9606:HIST1H4L			KEGG-&-1&-	HIST1H4L	Homo sapier	Gene Symbo	HIST1H4L	FALSE	HIST1H4L
taxon:9606	176629	1902	histone clust	gene	biological_pr	G:9606:HIST2H4A			KEGG-&-1&-	HIST2H4A	Homo sapier	Gene Symbo	HIST2H4A	FALSE	HIST2H4A
taxon:9606	180725	1902	glutaryl-CoA	gene	biological_pr	G:9606:GCDH			KEGG-&-1&-	GCDH	Homo sapier	Gene Symbo	GCDH	FALSE	GCDH
taxon:9606	176634	1902	histone clust	gene	biological_pr	G:9606:HIST1H4H			KEGG-&-1&-	HIST1H4H	Homo sapier	Gene Symbo	HIST1H4H	FALSE	HIST1H4H
taxon:9606	176635	1902	histone clust	gene	biological_pr	G:9606:HIST1H4C			KEGG-&-1&-	HIST1H4C	Homo sapier	Gene Symbo	HIST1H4C	FALSE	HIST1H4C
taxon:9606	176632	1902	histone clust	gene	biological_pr	G:9606:HIST1H4E			KEGG-&-1&-	HIST1H4E	Homo sapier	Gene Symbo	HIST1H4E	FALSE	HIST1H4E
taxon:9606	176633	1902	histone clust	gene	biological_pr	G:9606:HIST1H4B			KEGG-&-1&-	HIST1H4B	Homo sapier	Gene Symbo	HIST1H4B	FALSE	HIST1H4B
taxon:9606	168444	1902	neuronal PAS	gene	biological_pr	G:9606:NPAS3				NPAS3	Homo sapier	Gene Symbo	NPAS3	FALSE	NPAS3
taxon:9606	180733	1902	glioblastoma	gene	biological_pr	G:9606:GBAS				GBAS	Homo sapier	Gene Symbo	GBAS	FALSE	GBAS
taxon:9606	168450	1902	TSPY like 2	gene	biological_pr	G:9606:TSPYL2				TSPYL2	Homo sapier	Gene Symbo	TSPYL2	FALSE	TSPYL2
taxon:9606	176646	1902	tyrosine 3-m	gene	biological_pr	G:9606:YWHAZ			KEGG-&-1&-	YWHAZ	Homo sapier	Gene Symbo	YWHAZ	FALSE	YWHAZ
taxon:9606	176647	1902	tyrosine 3-m	gene	biological_pr	G:9606:YWHAH			KEGG-&-1&-	YWHAH	Homo sapier	Gene Symbo	YWHAH	FALSE	YWHAH
taxon:9606	176644	1902	splicing fact	gene	biological_pr	G:9606:SF1				SF1	Homo sapier	Gene Symbo	SF1	FALSE	SF1
taxon:9606	176650	1902	tyrosine 3-m	gene	biological_pr	G:9606:YWHAB			KEGG-&-1&-	YWHAB	Homo sapier	Gene Symbo	YWHAB	FALSE	YWHAB
taxon:9606	176651	1902	YY1 transcrip	gene	biological_pr	G:9606:YY1				YY1	Homo sapier	Gene Symbo	YY1	FALSE	YY1
taxon:9606	176648	1902	tyrosine 3-m	gene	biological_pr	G:9606:YWHAG			KEGG-&-1&-	YWHAG	Homo sapier	Gene Symbo	YWHAG	FALSE	YWHAG
taxon:9606	176649	1902	tyrosine 3-m	gene	biological_pr	G:9606:YWHAE			KEGG-&-1&-	YWHAE	Homo sapier	Gene Symbo	YWHAE	FALSE	YWHAE
taxon:9606	176653	1902	YES proto-on	gene	biological_pr	G:9606:YES1			KEGG-&-1&-	YES1	Homo sapier	Gene Symbo	YES1	FALSE	YES1
taxon:9606	180754	1902	glyceraldehy	gene	biological_pr	G:9606:GAPDH			KEGG-&-1&-	GAPDH	Homo sapier	Gene Symbo	GAPDH	FALSE	GAPDH
taxon:9606	172560	1902	RNA binding	gene	biological_pr	G:9606:RBMX			KEGG-&-1&-	RBMX	Homo sapier	Gene Symbo	RBMX	FALSE	RBMX
taxon:9606	176657	1902	X-ray repair c	gene	biological_pr	G:9606:XRCC5			KEGG-&-1&-	XRCC5	Homo sapier	Gene Symbo	XRCC5	FALSE	XRCC5
taxon:9606	176662	1902	exportin 1	gene	biological_pr	G:9606:XPO1			KEGG-&-1&-	XPO1	Homo sapier	Gene Symbo	XPO1	FALSE	XPO1
taxon:9606	176661	1902	X-ray repair c	gene	biological_pr	G:9606:XRCC1			KEGG-&-1&-	XRCC1	Homo sapier	Gene Symbo	XRCC1	FALSE	XRCC1
taxon:9606	176666	1902	XPA, DNA da	gene	biological_pr	G:9606:XPA			KEGG-&-1&-	XPA	Homo sapier	Gene Symbo	XPA	FALSE	XPA
taxon:9606	176665	1902	XPC complex	gene	biological_pr	G:9606:XPC			KEGG-&-1&-	XPC	Homo sapier	Gene Symbo	XPC	FALSE	XPC
taxon:9606	168479	1902	tRNA methyl	gene	biological_pr	G:9606:TRMT11				TRMT11	Homo sapier	Gene Symbo	TRMT11	FALSE	TRMT11
taxon:9606	180771	1902	ferritin heavy	gene	biological_pr	G:9606:FTH1			KEGG-&-1&-	FTH1	Homo sapier	Gene Symbo	FTH1	FALSE	FTH1
taxon:9606	172576	1902	DIM1 dimeth	gene	biological_pr	G:9606:DIMT1				DIMT1	Homo sapier	Gene Symbo	DIMT1	FALSE	DIMT1
taxon:9606	176679	1902	Wilms tumor	gene	biological_pr	G:9606:WT1			KEGG-&-1&-	WT1	Homo sapier	Gene Symbo	WT1	FALSE	WT1
taxon:9606	176682	1902	Werner synd	gene	biological_pr	G:9606:WRN				WRN	Homo sapier	Gene Symbo	WRN	FALSE	WRN
taxon:9606	180785	1902	mechanistic	gene	biological_pr	G:9606:MTOR			KEGG-&-1&-	MTOR	Homo sapier	Gene Symbo	MTOR	FALSE	MTOR
taxon:9606	176699	1902	nuclear rece	gene	biological_pr	G:9606:NSD2			KEGG-&-1&-	NSD2	Homo sapier	Gene Symbo	NSD2	FALSE	NSD2
taxon:9606	180798	1902	BRF1, RNA p	gene	biological_pr	G:9606:BRF1				BRF1	Homo sapier	Gene Symbo	BRF1	FALSE	BRF1
taxon:9606	164415	1902	NIMA relate	gene	biological_pr	G:9606:NEK7			KEGG-&-1&-	NEK7	Homo sapier	Gene Symbo	NEK7	FALSE	NEK7
taxon:9606	180802	1902	general trans	gene	biological_pr	G:9606:GTF2H4			KEGG-&-1&-	GTF2H4	Homo sapier	Gene Symbo	GTF2H4	FALSE	GTF2H4
taxon:9606	168514	1902	derlin 1	gene	biological_pr	G:9606:DERL1			KEGG-&-1&-	DERL1	Homo sapier	Gene Symbo	DERL1	FALSE	DERL1
taxon:9606	180803	1902	general trans	gene	biological_pr	G:9606:GTF2H3			KEGG-&-1&-	GTF2H3	Homo sapier	Gene Symbo	GTF2H3	FALSE	GTF2H3
taxon:9606	180801	1902	general trans	gene	biological_pr	G:9606:GTF2I			KEGG-&-1&-	GTF2I	Homo sapier	Gene Symbo	GTF2I	FALSE	GTF2I
taxon:9606	180807	1902	general trans	gene	biological_pr	G:9606:GTF2F2			KEGG-&-1&-	GTF2F2	Homo sapier	Gene Symbo	GTF2F2	FALSE	GTF2F2
taxon:9606	180804	1902	general trans	gene	biological_pr	G:9606:GTF2H2			KEGG-&-1&-	GTF2H2	Homo sapier	Gene Symbo	GTF2H2	FALSE	GTF2H2
taxon:9606	180805	1902	general trans	gene	biological_pr	G:9606:GTF2H1			KEGG-&-1&-	GTF2H1	Homo sapier	Gene Symbo	GTF2H1	FALSE	GTF2H1
taxon:9606	164421	1902	transcription	gene	biological_pr	G:9606:TCEAL2				TCEAL2	Homo sapier	Gene Symbo	TCEAL2	FALSE	TCEAL2
taxon:9606	164427	1902	ring finger pr	gene	cellular_com	G:9606:RNF32				RNF32	Homo sapier	Gene Symbo	RNF32	FALSE	RNF32
taxon:9606	176715	1902	vitronectin	gene	biological_pr	G:9606:VTN			KEGG-&-1&-	VTN	Homo sapier	Gene Symbo	VTN	FALSE	VTN
taxon:9606	180808	1902	general trans	gene	biological_pr	G:9606:GTF2F1			KEGG-&-1&-	GTF2F1	Homo sapier	Gene Symbo	GTF2F1	FALSE	GTF2F1
taxon:9606	180814	1902	mutS homolo	gene	biological_pr	G:9606:MSH6			KEGG-&-1&-	MSH6	Homo sapier	Gene Symbo	MSH6	FALSE	MSH6
taxon:9606	176718	1902	vaccinia relat	gene	biological_pr	G:9606:VRK2				VRK2	Homo sapier	Gene Symbo	VRK2	FALSE	VRK2
taxon:9606	176719	1902	vaccinia relat	gene	biological_pr	G:9606:VRK1				VRK1	Homo sapier	Gene Symbo	VRK1	FALSE	VRK1
taxon:9606	180813	1902	general trans	gene	biological_pr	G:9606:GTF2A1			KEGG-&-1&-	GTF2A1	Homo sapier	Gene Symbo	GTF2A1	FALSE	GTF2A1
taxon:9606	180819	1902	glutathione S	gene	biological_pr	G:9606:GSTP1			KEGG-&-1&-	GSTP1	Homo sapier	Gene Symbo	GSTP1	FALSE	GSTP1
taxon:9606	176727	1902	vasoactive in	gene	biological_pr	G:9606:VIPR1			KEGG-&-1&-	VIPR1	Homo sapier	Gene Symbo	VIPR1	FALSE	VIPR1
taxon:9606	180821	1902	glutathione S	gene	biological_pr	G:9606:GSTM4			KEGG-&-1&-	GSTM4	Homo sapier	Gene Symbo	GSTM4	FALSE	GSTM4
taxon:9606	176730	1902	ezrin	gene	biological_pr	G:9606:EZR			KEGG-&-1&-	EZR	Homo sapier	Gene Symbo	EZR	FALSE	EZR
taxon:9606	168539	1902	WD repeat d	gene	biological_pr	G:9606:WDR77				WDR77	Homo sapier	Gene Symbo	WDR77	FALSE	WDR77
taxon:9606	176729	1902	vimentin	gene	biological_pr	G:9606:VIM			KEGG-&-1&-	VIM	Homo sapier	Gene Symbo	VIM	FALSE	VIM
taxon:9606	172639	1902	ankyrin repe	gene	biological_pr	G:9606:ANKRD2				ANKRD2	Homo sapier	Gene Symbo	ANKRD2	FALSE	ANKRD2
taxon:9606	176735	1902	vascular end	gene	biological_pr	G:9606:VEGFB			KEGG-&-1&-	VEGFB	Homo sapier	Gene Symbo	VEGFB	FALSE	VEGFB
taxon:9606	176732	1902	von Hippel-Li	gene	biological_pr	G:9606:VHL			KEGG-&-1&-	VHL	Homo sapier	Gene Symbo	VHL	FALSE	VHL
taxon:9606	180835	1902	glycogen syn	gene	biological_pr	G:9606:GSK3B			KEGG-&-1&-	GSK3B	Homo sapier	Gene Symbo	GSK3B	FALSE	GSK3B
taxon:9606	176736	1902	vascular end	gene	biological_pr	G:9606:VEGFA	VEGFA	TRUE	KEGG-&-1&-	VEGFA	Homo sapier	Gene Symbo	VEGFA	FALSE	VEGFA
taxon:9606	176737	1902	vitamin D (1,	gene	biological_pr	G:9606:VDR			KEGG-&-1&-	VDR	Homo sapier	Gene Symbo	VDR	FALSE	VDR
taxon:9606	180833	1902	gelsolin	gene	biological_pr	G:9606:GSN			KEGG-&-1&-	GSN	Homo sapier	Gene Symbo	GSN	FALSE	GSN
taxon:9606	176742	1902	voltage depe	gene	biological_pr	G:9606:VDAC1			KEGG-&-1&-	VDAC1	Homo sapier	Gene Symbo	VDAC1	FALSE	VDAC1
taxon:9606	168551	1902	FTO, alpha-k	gene	biological_pr	G:9606:FTO	FTO	TRUE		FTO	Homo sapier	Gene Symbo	FTO	FALSE	FTO
taxon:9606	176743	1902	valosin conta	gene	biological_pr	G:9606:VCP			KEGG-&-1&-	VCP	Homo sapier	Gene Symbo	VCP	FALSE	VCP
taxon:9606	180836	1902	glycogen syn	gene	biological_pr	G:9606:GSK3A			KEGG-&-1&-	GSK3A	Homo sapier	Gene Symbo	GSK3A	FALSE	GSK3A
taxon:9606	176741	1902	voltage depe	gene	biological_pr	G:9606:VDAC2			KEGG-&-1&-	VDAC2	Homo sapier	Gene Symbo	VDAC2	FALSE	VDAC2
taxon:9606	172651	1902	F-box protein	gene	biological_pr	G:9606:FBXO4			KEGG-&-1&-	FBXO4	Homo sapier	Gene Symbo	FBXO4	FALSE	FBXO4
taxon:9606	176747	1902	vav guanine	gene	biological_pr	G:9606:VAV2			KEGG-&-1&-	VAV2	Homo sapier	Gene Symbo	VAV2	FALSE	VAV2
taxon:9606	176744	1902	vinculin	gene	biological_pr	G:9606:VCL			KEGG-&-1&-	VCL	Homo sapier	Gene Symbo	VCL	FALSE	VCL
taxon:9606	176745	1902	vascular cell	gene	biological_pr	G:9606:VCAM1	VCAM1	TRUE	KEGG-&-1&-	VCAM1	Homo sapier	Gene Symbo	VCAM1	FALSE	VCAM1
taxon:9606	176750	1902	valyl-tRNA sy	gene	biological_pr	G:9606:VARS			KEGG-&-1&-	VARS	Homo sapier	Gene Symbo	VARS	FALSE	VARS
taxon:9606	176748	1902	vav guanine	gene	biological_pr	G:9606:VAV1			KEGG-&-1&-	VAV1	Homo sapier	Gene Symbo	VAV1	FALSE	VAV1
taxon:9606	180844	1902	C-X-C motif c	gene	biological_pr	G:9606:CXCL1			KEGG-&-1&-	CXCL1	Homo sapier	Gene Symbo	CXCL1	FALSE	CXCL1
taxon:9606	176749	1902	vasodilator-s	gene	biological_pr	G:9606:VASP			KEGG-&-1&-	VASP	Homo sapier	Gene Symbo	VASP	FALSE	VASP
taxon:9606	168557	1902	proline rich a	gene	biological_pr	G:9606:PRRG4				PRRG4	Homo sapier	Gene Symbo	PRRG4	FALSE	PRRG4
taxon:9606	168563	1902	nucleolar co	gene	biological_pr	G:9606:NOC4L				NOC4L	Homo sapier	Gene Symbo	NOC4L	FALSE	NOC4L
taxon:9606	176753	1902	lysine demet	gene	biological_pr	G:9606:KDM6A			KEGG-&-1&-	KDM6A	Homo sapier	Gene Symbo	KDM6A	FALSE	KDM6A
taxon:9606	172662	1902	F-box and W	gene	biological_pr	G:9606:FBXW8			KEGG-&-1&-	FBXW8	Homo sapier	Gene Symbo	FBXW8	FALSE	FBXW8
taxon:9606	180855	1902	nuclear rece	gene	biological_pr	G:9606:NR3C1	NR3C1	TRUE	KEGG-&-1&-	NR3C1	Homo sapier	Gene Symbo	NR3C1	FALSE	NR3C1
taxon:9606	168568	1902	DEAD-box he	gene	biological_pr	G:9606:DDX54				DDX54	Homo sapier	Gene Symbo	DDX54	FALSE	DDX54
taxon:9606	176760	1902	upstream tra	gene	biological_pr	G:9606:USF1				USF1	Homo sapier	Gene Symbo	USF1	FALSE	USF1
taxon:9606	168572	1902	nucleic acid b	gene	biological_pr	G:9606:NABP2				NABP2	Homo sapier	Gene Symbo	NABP2	FALSE	NABP2
taxon:9606	176771	1902	nuclear rece	gene	biological_pr	G:9606:NR1H2			KEGG-&-1&-	NR1H2	Homo sapier	Gene Symbo	NR1H2	FALSE	NR1H2
taxon:9606	180867	1902	granulin prec	gene	biological_pr	G:9606:GRN				GRN	Homo sapier	Gene Symbo	GRN	FALSE	GRN
taxon:9606	176775	1902	uridine mon	gene	biological_pr	G:9606:UMPS			KEGG-&-1&-	UMPS	Homo sapier	Gene Symbo	UMPS	FALSE	UMPS
taxon:9606	180878	1902	growth facto	gene	biological_pr	G:9606:GRB2			KEGG-&-1&-	GRB2	Homo sapier	Gene Symbo	GRB2	FALSE	GRB2
taxon:9606	172686	1902	signal transd	gene	biological_pr	G:9606:STAP1				STAP1	Homo sapier	Gene Symbo	STAP1	FALSE	STAP1
taxon:9606	180877	1902	growth facto	gene	biological_pr	G:9606:GRB7				GRB7	Homo sapier	Gene Symbo	GRB7	FALSE	GRB7
taxon:9606	176786	1902	UDP-glucose	gene	biological_pr	G:9606:UGDH			KEGG-&-1&-	UGDH	Homo sapier	Gene Symbo	UGDH	FALSE	UGDH
taxon:9606	168593	1902	sodium chan	gene	biological_pr	G:9606:SCNM1				SCNM1	Homo sapier	Gene Symbo	SCNM1	FALSE	SCNM1
taxon:9606	176790	1902	ubiquitin fus	gene	biological_pr	G:9606:UFD1L			KEGG-&-1&-	UFD1L	Homo sapier	Gene Symbo	UFD1L	FALSE	UFD1L
taxon:9606	180887	1902	G protein pat	gene	biological_pr	G:9606:GPS2			KEGG-&-1&-	GPS2	Homo sapier	Gene Symbo	GPS2	FALSE	GPS2
taxon:9606	180884	1902	glutathione p	gene	biological_pr	G:9606:GPX2			KEGG-&-1&-	GPX2	Homo sapier	Gene Symbo	GPX2	FALSE	GPX2
taxon:9606	180888	1902	G protein pat	gene	biological_pr	G:9606:GPS1				GPS1	Homo sapier	Gene Symbo	GPS1	FALSE	GPS1
taxon:9606	180889	1902	MAP kinase i	gene	biological_pr	G:9606:MKNK2			KEGG-&-1&-	MKNK2	Homo sapier	Gene Symbo	MKNK2	FALSE	MKNK2
taxon:9606	180892	1902	G protein-co	gene	biological_pr	G:9606:GRK5			KEGG-&-1&-	GRK5	Homo sapier	Gene Symbo	GRK5	FALSE	GRK5
taxon:9606	176797	1902	ubiquitin C-t	gene	biological_pr	G:9606:UCHL1			KEGG-&-1&-	UCHL1	Homo sapier	Gene Symbo	UCHL1	FALSE	UCHL1
taxon:9606	172707	1902	SUMO1/sent	gene	biological_pr	G:9606:SENP3				SENP3	Homo sapier	Gene Symbo	SENP3	FALSE	SENP3
taxon:9606	168611	1902	dual specifici	gene	biological_pr	G:9606:DUSP26				DUSP26	Homo sapier	Gene Symbo	DUSP26	FALSE	DUSP26
taxon:9606	176803	1902	ubiquitin pro	gene	biological_pr	G:9606:UBE3A			KEGG-&-1&-	UBE3A	Homo sapier	Gene Symbo	UBE3A	FALSE	UBE3A
taxon:9606	176800	1902	small ubiquit	gene	biological_pr	G:9606:SUMO1			KEGG-&-1&-	SUMO1	Homo sapier	Gene Symbo	SUMO1	FALSE	SUMO1
taxon:9606	176806	1902	ubiquitin con	gene	biological_pr	G:9606:UBE2N			KEGG-&-1&-	UBE2N	Homo sapier	Gene Symbo	UBE2N	FALSE	UBE2N
taxon:9606	172715	1902	NOC2 like nu	gene	biological_pr	G:9606:NOC2L				NOC2L	Homo sapier	Gene Symbo	NOC2L	FALSE	NOC2L
taxon:9606	176808	1902	ubiquitin con	gene	biological_pr	G:9606:UBE2L3			KEGG-&-1&-	UBE2L3	Homo sapier	Gene Symbo	UBE2L3	FALSE	UBE2L3
taxon:9606	176809	1902	ubiquitin con	gene	biological_pr	G:9606:UBE2I			KEGG-&-1&-	UBE2I	Homo sapier	Gene Symbo	UBE2I	FALSE	UBE2I
taxon:9606	176818	1902	ubiquitin con	gene	biological_pr	G:9606:UBE2B			KEGG-&-1&-	UBE2B	Homo sapier	Gene Symbo	UBE2B	FALSE	UBE2B
taxon:9606	172727	1902	zinc finger ar	gene	biological_pr	G:9606:ZBTB20				ZBTB20	Homo sapier	Gene Symbo	ZBTB20	FALSE	ZBTB20
taxon:9606	168628	1902	family with s	gene	cellular_com	G:9606:FAM173A				FAM173A	Homo sapier	Gene Symbo	FAM173A	FALSE	FAM173A
taxon:9606	176820	1902	ubiquitin B	gene	biological_pr	G:9606:UBB			KEGG-&-1&-	UBB	Homo sapier	Gene Symbo	UBB	FALSE	UBB
taxon:9606	172729	1902	SERPINE1 ml	gene	biological_pr	G:9606:SERBP1				SERBP1	Homo sapier	Gene Symbo	SERBP1	FALSE	SERBP1
taxon:9606	176825	1902	U2 small nuc	gene	biological_pr	G:9606:U2AF1			KEGG-&-1&-	U2AF1	Homo sapier	Gene Symbo	U2AF1	FALSE	U2AF1
taxon:9606	172732	1902	GTPase activ	gene	biological_pr	G:9606:GAPVD1				GAPVD1	Homo sapier	Gene Symbo	GAPVD1	FALSE	GAPVD1
taxon:9606	176835	1902	thioredoxin r	gene	biological_pr	G:9606:TXNRD1			KEGG-&-1&-	TXNRD1	Homo sapier	Gene Symbo	TXNRD1	FALSE	TXNRD1
taxon:9606	176833	1902	thymidylate s	gene	biological_pr	G:9606:TYMS			KEGG-&-1&-	TYMS	Homo sapier	Gene Symbo	TYMS	FALSE	TYMS
taxon:9606	176836	1902	thioredoxin	gene	biological_pr	G:9606:TXN			KEGG-&-1&-	TXN	Homo sapier	Gene Symbo	TXN	FALSE	TXN
taxon:9606	176842	1902	tubby like pro	gene	biological_pr	G:9606:TULP3				TULP3	Homo sapier	Gene Symbo	TULP3	FALSE	TULP3
taxon:9606	176840	1902	twist family	gene	biological_pr	G:9606:TWIST1			KEGG-&-1&-	TWIST1	Homo sapier	Gene Symbo	TWIST1	FALSE	TWIST1
taxon:9606	176846	1902	Tu translatio	gene	biological_pr	G:9606:TUFM				TUFM	Homo sapier	Gene Symbo	TUFM	FALSE	TUFM
taxon:9606	176847	1902	tubulin gam	gene	biological_pr	G:9606:TUBG1			KEGG-&-1&-	TUBG1	Homo sapier	Gene Symbo	TUBG1	FALSE	TUBG1
taxon:9606	176850	1902	tubulin alpha	gene	biological_pr	G:9606:TUBA4A			KEGG-&-1&-	TUBA4A	Homo sapier	Gene Symbo	TUBA4A	FALSE	TUBA4A
taxon:9606	176851	1902	transthyretin	gene	biological_pr	G:9606:TTR			KEGG-&-1&-	TTR	Homo sapier	Gene Symbo	TTR	FALSE	TTR
taxon:9606	176848	1902	tubulin beta	gene	biological_pr	G:9606:TUBB2A			KEGG-&-1&-	TUBB2A	Homo sapier	Gene Symbo	TUBB2A	FALSE	TUBB2A
taxon:9606	127707	1902	BUB1B-PAK6	gene		G:9606:BUB1B-PAK6			KEGG-&-1&-	BUB1B-PAK6	Homo sapier	Gene Symbo	BUB1B-PAK6	FALSE	BUB1B-PAK6
taxon:9606	168671	1902	transmembr	gene	biological_pr	G:9606:TMEM135				TMEM135	Homo sapier	Gene Symbo	TMEM135	FALSE	TMEM135
taxon:9606	168668	1902	MARCKS like	gene	biological_pr	G:9606:MARCKSL1			KEGG-&-1&-	MARCKSL1	Homo sapier	Gene Symbo	MARCKSL1	FALSE	MARCKSL1
taxon:9606	172769	1902	DNA polyme	gene	biological_pr	G:9606:POLDIP2				POLDIP2	Homo sapier	Gene Symbo	POLDIP2	FALSE	POLDIP2
taxon:9606	172773	1902	LSM14A, mR	gene	biological_pr	G:9606:LSM14A				LSM14A	Homo sapier	Gene Symbo	LSM14A	FALSE	LSM14A
taxon:9606	172778	1902	adaptor prot	gene	biological_pr	G:9606:APPL1			KEGG-&-1&-	APPL1	Homo sapier	Gene Symbo	APPL1	FALSE	APPL1
taxon:9606	172780	1902	GRB10 intera	gene	biological_pr	G:9606:GIGYF2				GIGYF2	Homo sapier	Gene Symbo	GIGYF2	FALSE	GIGYF2
taxon:9606	176882	1902	BCL2 associa	gene	biological_pr	G:9606:BAG6				BAG6	Homo sapier	Gene Symbo	BAG6	FALSE	BAG6
taxon:9606	176883	1902	proline rich c	gene	biological_pr	G:9606:PRRC2A				PRRC2A	Homo sapier	Gene Symbo	PRRC2A	FALSE	PRRC2A
taxon:9606	176880	1902	DExD-box hel	gene	biological_pr	G:9606:DDX39B			KEGG-&-1&-	DDX39B	Homo sapier	Gene Symbo	DDX39B	FALSE	DDX39B
taxon:9606	176884	1902	aldehyde deh	gene	biological_pr	G:9606:ALDH5A1			KEGG-&-1&-	ALDH5A1	Homo sapier	Gene Symbo	ALDH5A1	FALSE	ALDH5A1
taxon:9606	168699	1902	mitochondri	gene	biological_pr	G:9606:MRPL41				MRPL41	Homo sapier	Gene Symbo	MRPL41	FALSE	MRPL41
taxon:9606	168697	1902	mitochondri	gene	biological_pr	G:9606:MRPL38				MRPL38	Homo sapier	Gene Symbo	MRPL38	FALSE	MRPL38
taxon:9606	168702	1902	mitochondri	gene	biological_pr	G:9606:MRPS9			KEGG-&-1&-	MRPS9	Homo sapier	Gene Symbo	MRPS9	FALSE	MRPS9
taxon:9606	172798	1902	zinc finger pr	gene	biological_pr	G:9606:ZNF451				ZNF451	Homo sapier	Gene Symbo	ZNF451	FALSE	ZNF451
taxon:9606	168700	1902	mitochondri	gene	biological_pr	G:9606:MRPS5			KEGG-&-1&-	MRPS5	Homo sapier	Gene Symbo	MRPS5	FALSE	MRPS5
taxon:9606	168704	1902	mitochondri	gene	biological_pr	G:9606:MRPS15			KEGG-&-1&-	MRPS15	Homo sapier	Gene Symbo	MRPS15	FALSE	MRPS15
taxon:9606	164615	1902	junction med	gene	biological_pr	G:9606:JMY				JMY	Homo sapier	Gene Symbo	JMY	FALSE	JMY
taxon:9606	176903	1902	ubiquitin spe	gene	biological_pr	G:9606:USP7			KEGG-&-1&-	USP7	Homo sapier	Gene Symbo	USP7	FALSE	USP7
taxon:9606	172812	1902	F-box protein	gene	biological_pr	G:9606:FBXO7				FBXO7	Homo sapier	Gene Symbo	FBXO7	FALSE	FBXO7
taxon:9606	176912	1902	secretogranin	gene	biological_pr	G:9606:SCG2				SCG2	Homo sapier	Gene Symbo	SCG2	FALSE	SCG2
taxon:9606	176918	1902	tubulin alpha	gene	biological_pr	G:9606:TUBA1A			KEGG-&-1&-	TUBA1A	Homo sapier	Gene Symbo	TUBA1A	FALSE	TUBA1A
taxon:9606	176922	1902	mannosyl-oli	gene	biological_pr	G:9606:MOGS			KEGG-&-1&-	MOGS	Homo sapier	Gene Symbo	MOGS	FALSE	MOGS
taxon:9606	168729	1902	nucleic acid b	gene	biological_pr	G:9606:NABP1				NABP1	Homo sapier	Gene Symbo	NABP1	FALSE	NABP1
taxon:9606	181017	1902	Fos proto-on	gene	biological_pr	G:9606:FOS			KEGG-&-1&-	FOS	Homo sapier	Gene Symbo	FOS	FALSE	FOS
taxon:9606	176927	1902	BTG anti-pro	gene	biological_pr	G:9606:BTG2			KEGG-&-1&-	BTG2	Homo sapier	Gene Symbo	BTG2	FALSE	BTG2
taxon:9606	176931	1902	death associ	gene	biological_pr	G:9606:DAP3				DAP3	Homo sapier	Gene Symbo	DAP3	FALSE	DAP3
taxon:9606	181025	1902	fibronectin 1	gene	biological_pr	G:9606:FN1			KEGG-&-1&-	FN1	Homo sapier	Gene Symbo	FN1	FALSE	FN1
taxon:9606	172838	1902	pre-mRNA p	gene	biological_pr	G:9606:PRPF6			KEGG-&-1&-	PRPF6	Homo sapier	Gene Symbo	PRPF6	FALSE	PRPF6
taxon:9606	172837	1902	zinc finger pr	gene	biological_pr	G:9606:ZNF318				ZNF318	Homo sapier	Gene Symbo	ZNF318	FALSE	ZNF318
taxon:9606	172842	1902	tuftelin inter	gene	biological_pr	G:9606:TFIP11				TFIP11	Homo sapier	Gene Symbo	TFIP11	FALSE	TFIP11
taxon:9606	181034	1902	fms related t	gene	biological_pr	G:9606:FLT4			KEGG-&-1&-	FLT4	Homo sapier	Gene Symbo	FLT4	FALSE	FLT4
taxon:9606	181038	1902	flotillin 2	gene	biological_pr	G:9606:FLOT2			KEGG-&-1&-	FLOT2	Homo sapier	Gene Symbo	FLOT2	FALSE	FLOT2
taxon:9606	181039	1902	filamin C	gene	biological_pr	G:9606:FLNC			KEGG-&-1&-	FLNC	Homo sapier	Gene Symbo	FLNC	FALSE	FLNC
taxon:9606	176940	1902	PR/SET dom	gene	biological_pr	G:9606:PRDM2			KEGG-&-1&-	PRDM2	Homo sapier	Gene Symbo	PRDM2	FALSE	PRDM2
taxon:9606	181037	1902	fms related t	gene	biological_pr	G:9606:FLT1			KEGG-&-1&-	FLT1	Homo sapier	Gene Symbo	FLT1	FALSE	FLT1
taxon:9606	168755	1902	EF-hand calci	gene	biological_pr	G:9606:EFCAB6				EFCAB6	Homo sapier	Gene Symbo	EFCAB6	FALSE	EFCAB6
taxon:9606	176944	1902	zyxin	gene	biological_pr	G:9606:ZYX			KEGG-&-1&-	ZYX	Homo sapier	Gene Symbo	ZYX	FALSE	ZYX
taxon:9606	181040	1902	filamin B	gene	biological_pr	G:9606:FLNB			KEGG-&-1&-	FLNB	Homo sapier	Gene Symbo	FLNB	FALSE	FLNB
taxon:9606	181041	1902	filamin A	gene	biological_pr	G:9606:FLNA			KEGG-&-1&-	FLNA	Homo sapier	Gene Symbo	FLNA	FALSE	FLNA
taxon:9606	168753	1902	nicotinamide	gene	biological_pr	G:9606:NMNAT1			KEGG-&-1&-	NMNAT1	Homo sapier	Gene Symbo	NMNAT1	FALSE	NMNAT1
taxon:9606	181050	1902	forkhead box	gene	biological_pr	G:9606:FOXS1				FOXS1	Homo sapier	Gene Symbo	FOXS1	FALSE	FOXS1
taxon:9606	181048	1902	forkhead box	gene	biological_pr	G:9606:FOXO3			KEGG-&-1&-	FOXO3	Homo sapier	Gene Symbo	FOXO3	FALSE	FOXO3
taxon:9606	181049	1902	forkhead box	gene	biological_pr	G:9606:FOXO1			KEGG-&-1&-	FOXO1	Homo sapier	Gene Symbo	FOXO1	FALSE	FOXO1
taxon:9606	168770	1902	ceramide kin	gene	biological_pr	G:9606:CERK			KEGG-&-1&-	CERK	Homo sapier	Gene Symbo	CERK	FALSE	CERK
taxon:9606	164672	1902	splicing regu	gene	biological_pr	G:9606:SREK1				SREK1	Homo sapier	Gene Symbo	SREK1	FALSE	SREK1
taxon:9606	164673	1902	poly(A) bindi	gene	cellular_com	G:9606:PABPC5			KEGG-&-1&-	PABPC5	Homo sapier	Gene Symbo	PABPC5	FALSE	PABPC5
taxon:9606	181057	1902	forkhead box	gene	biological_pr	G:9606:FOXL1				FOXL1	Homo sapier	Gene Symbo	FOXL1	FALSE	FOXL1
taxon:9606	168772	1902	methenyltetr	gene	biological_pr	G:9606:MTHFSD				MTHFSD	Homo sapier	Gene Symbo	MTHFSD	FALSE	MTHFSD
taxon:9606	181061	1902	forkhead box	gene	biological_pr	G:9606:FOXC1				FOXC1	Homo sapier	Gene Symbo	FOXC1	FALSE	FOXC1
taxon:9606	181066	1902	FK506 bindin	gene	biological_pr	G:9606:FKBP4			KEGG-&-1&-	FKBP4	Homo sapier	Gene Symbo	FKBP4	FALSE	FKBP4
taxon:9606	181067	1902	FK506 bindin	gene	biological_pr	G:9606:FKBP3				FKBP3	Homo sapier	Gene Symbo	FKBP3	FALSE	FKBP3
taxon:9606	181064	1902	forkhead box	gene	biological_pr	G:9606:FOXG1			KEGG-&-1&-	FOXG1	Homo sapier	Gene Symbo	FOXG1	FALSE	FOXG1
taxon:9606	181065	1902	FK506 bindin	gene	biological_pr	G:9606:FKBP5			KEGG-&-1&-	FKBP5	Homo sapier	Gene Symbo	FKBP5	FALSE	FKBP5
taxon:9606	181079	1902	four and a ha	gene	biological_pr	G:9606:FHL2			KEGG-&-1&-	FHL2	Homo sapier	Gene Symbo	FHL2	FALSE	FHL2
taxon:9606	160603	1902	metaxin 3	gene	biological_pr	G:9606:MTX3				MTX3	Homo sapier	Gene Symbo	MTX3	FALSE	MTX3
taxon:9606	181084	1902	FGR proto-or	gene	biological_pr	G:9606:FGR			KEGG-&-1&-	FGR	Homo sapier	Gene Symbo	FGR	FALSE	FGR
taxon:9606	160605	1902	actin, beta lil	gene	biological_pr	G:9606:ACTBL2				ACTBL2	Homo sapier	Gene Symbo	ACTBL2	FALSE	ACTBL2
taxon:9606	176994	1902	zinc finger ar	gene	biological_pr	G:9606:ZBTB17			KEGG-&-1&-	ZBTB17	Homo sapier	Gene Symbo	ZBTB17	FALSE	ZBTB17
taxon:9606	164707	1902	ribosomal pr	gene	biological_pr	G:9606:RPL10L			KEGG-&-1&-	RPL10L	Homo sapier	Gene Symbo	RPL10L	FALSE	RPL10L
taxon:9606	176995	1902	zinc finger pr	gene	biological_pr	G:9606:ZNF148				ZNF148	Homo sapier	Gene Symbo	ZNF148	FALSE	ZNF148
taxon:9606	181091	1902	fibroblast gr	gene	biological_pr	G:9606:FGFR1			KEGG-&-1&-	FGFR1	Homo sapier	Gene Symbo	FGFR1	FALSE	FGFR1
taxon:9606	176998	1902	zinc finger ar	gene	biological_pr	G:9606:ZBTB16			KEGG-&-1&-	ZBTB16	Homo sapier	Gene Symbo	ZBTB16	FALSE	ZBTB16
taxon:9606	181097	1902	EGF contain	gene	biological_pr	G:9606:EFEMP1				EFEMP1	Homo sapier	Gene Symbo	EFEMP1	FALSE	EFEMP1
taxon:9606	181101	1902	FAU, ubiquiti	gene	biological_pr	G:9606:FAU			KEGG-&-1&-	FAU	Homo sapier	Gene Symbo	FAU	FALSE	FAU
taxon:9606	181104	1902	fatty acid syn	gene	biological_pr	G:9606:FASN			KEGG-&-1&-	FASN	Homo sapier	Gene Symbo	FASN	FALSE	FASN
taxon:9606	156529	1902	nuclear pore	gene		G:9606:NPIPB13				NPIPB13	Homo sapier	Gene Symbo	NPIPB13	FALSE	NPIPB13
taxon:9606	181105	1902	phenylalanyl-	gene	biological_pr	G:9606:FARSA			KEGG-&-1&-	FARSA	Homo sapier	Gene Symbo	FARSA	FALSE	FARSA
taxon:9606	181114	1902	acyl-CoA syn	gene	biological_pr	G:9606:ACSL4			KEGG-&-1&-	ACSL4	Homo sapier	Gene Symbo	ACSL4	FALSE	ACSL4
taxon:9606	168828	1902	RNA binding	gene	biological_pr	G:9606:RBM25			KEGG-&-1&-	RBM25	Homo sapier	Gene Symbo	RBM25	FALSE	RBM25
taxon:9606	168836	1902	lysine methyl	gene	biological_pr	G:9606:KMT2C			KEGG-&-1&-	KMT2C	Homo sapier	Gene Symbo	KMT2C	FALSE	KMT2C
taxon:9606	181132	1902	cyclin G asso	gene	biological_pr	G:9606:GAK				GAK	Homo sapier	Gene Symbo	GAK	FALSE	GAK
taxon:9606	168845	1902	lymphocyte a	gene	biological_pr	G:9606:LY6G5B				LY6G5B	Homo sapier	Gene Symbo	LY6G5B	FALSE	LY6G5B
taxon:9606	168854	1902	CREB/ATF b	gene	biological_pr	G:9606:CREBZF				CREBZF	Homo sapier	Gene Symbo	CREBZF	FALSE	CREBZF
taxon:9606	177045	1902	TTK protein k	gene	biological_pr	G:9606:TTK			KEGG-&-1&-	TTK	Homo sapier	Gene Symbo	TTK	FALSE	TTK
taxon:9606	164757	1902	lin-54 DREA	gene	biological_pr	G:9606:LIN54			KEGG-&-1&-	LIN54	Homo sapier	Gene Symbo	LIN54	FALSE	LIN54
taxon:9606	177050	1902	DnaJ heat sh	gene	biological_pr	G:9606:DNAJC7				DNAJC7	Homo sapier	Gene Symbo	DNAJC7	FALSE	DNAJC7
taxon:9606	168859	1902	ras homolog	gene	biological_pr	G:9606:RHOU				RHOU	Homo sapier	Gene Symbo	RHOU	FALSE	RHOU
taxon:9606	177049	1902	tetratricope	gene	biological_pr	G:9606:TTC3				TTC3	Homo sapier	Gene Symbo	TTC3	FALSE	TTC3
taxon:9606	177056	1902	TSPY like 1	gene	biological_pr	G:9606:TSPYL1				TSPYL1	Homo sapier	Gene Symbo	TSPYL1	FALSE	TSPYL1
taxon:9606	177057	1902	testis specifi	gene	biological_pr	G:9606:TSPY1				TSPY1	Homo sapier	Gene Symbo	TSPY1	FALSE	TSPY1
taxon:9606	168865	1902	sulfide quino	gene	biological_pr	G:9606:SQRDL			KEGG-&-1&-	SQRDL	Homo sapier	Gene Symbo	SQRDL	FALSE	SQRDL
taxon:9606	181163	1902	X-ray repair c	gene	biological_pr	G:9606:XRCC6			KEGG-&-1&-	XRCC6	Homo sapier	Gene Symbo	XRCC6	FALSE	XRCC6
taxon:9606	177064	1902	tumor susce	gene	biological_pr	G:9606:TSG101			KEGG-&-1&-	TSG101	Homo sapier	Gene Symbo	TSG101	FALSE	TSG101
taxon:9606	177065	1902	tuberous scle	gene	biological_pr	G:9606:TSC2			KEGG-&-1&-	TSC2	Homo sapier	Gene Symbo	TSC2	FALSE	TSC2
taxon:9606	156588	1902	dihydrofolate	gene	biological_pr	G:9606:DHFRP1				DHFRP1	Homo sapier	Gene Symbo	DHFRP1	FALSE	DHFRP1
taxon:9606	164780	1902	TAM41 mito	gene	biological_pr	G:9606:TAMM41				TAMM41	Homo sapier	Gene Symbo	TAMM41	FALSE	TAMM41
taxon:9606	156594	1902	histone clust	gene	biological_pr	G:9606:HIST2H4B			KEGG-&-1&-	HIST2H4B	Homo sapier	Gene Symbo	HIST2H4B	FALSE	HIST2H4B
taxon:9606	181171	1902	FYN proto-or	gene	biological_pr	G:9606:FYN			KEGG-&-1&-	FYN	Homo sapier	Gene Symbo	FYN	FALSE	FYN
taxon:9606	168895	1902	cell cycle an	gene	biological_pr	G:9606:CCAR2				CCAR2	Homo sapier	Gene Symbo	CCAR2	FALSE	CCAR2
taxon:9606	181184	1902	FUS RNA bin	gene	biological_pr	G:9606:FUS			KEGG-&-1&-	FUS	Homo sapier	Gene Symbo	FUS	FALSE	FUS
taxon:9606	181189	1902	nuclear rece	gene	biological_pr	G:9606:NR5A1			KEGG-&-1&-	NR5A1	Homo sapier	Gene Symbo	NR5A1	FALSE	NR5A1
taxon:9606	173003	1902	apoptosis an	gene	biological_pr	G:9606:AATF				AATF	Homo sapier	Gene Symbo	AATF	FALSE	AATF
taxon:9606	144329	1902	putative inac	gene		G:9606:LOC100653061				LOC10065306	Homo sapier	Gene Symbo	LOC10065306	FALSE	LOC100653061
taxon:9606	156622	1902	POU class 5	gene		G:9606:POU5F1P3				POU5F1P3	Homo sapier	Gene Symbo	POU5F1P3	FALSE	POU5F1P3
taxon:9606	168909	1902	RB associate	gene	biological_pr	G:9606:RBAK				RBAK	Homo sapier	Gene Symbo	RBAK	FALSE	RBAK
taxon:9606	181202	1902	coagulation f	gene	biological_pr	G:9606:F12			KEGG-&-1&-	F12	Homo sapier	Gene Symbo	F12	FALSE	F12
taxon:9606	177105	1902	chaperonin c	gene	biological_pr	G:9606:CCT3				CCT3	Homo sapier	Gene Symbo	CCT3	FALSE	CCT3
taxon:9606	173019	1902	large tumor s	gene	biological_pr	G:9606:LATS2			KEGG-&-1&-	LATS2	Homo sapier	Gene Symbo	LATS2	FALSE	LATS2
taxon:9606	173016	1902	DAZ associat	gene	biological_pr	G:9606:DAZAP1			KEGG-&-1&-	DAZAP1	Homo sapier	Gene Symbo	DAZAP1	FALSE	DAZAP1
taxon:9606	177119	1902	TNF receptor	gene	biological_pr	G:9606:TRAF2			KEGG-&-1&-	TRAF2	Homo sapier	Gene Symbo	TRAF2	FALSE	TRAF2
taxon:9606	181215	1902	enhancer of z	gene	biological_pr	G:9606:EZH2			KEGG-&-1&-	EZH2	Homo sapier	Gene Symbo	EZH2	FALSE	EZH2
taxon:9606	173020	1902	argonaute 1,	gene	biological_pr	G:9606:AGO1				AGO1	Homo sapier	Gene Symbo	AGO1	FALSE	AGO1
taxon:9606	177116	1902	TNF receptor	gene	biological_pr	G:9606:TRAF6			KEGG-&-1&-	TRAF6	Homo sapier	Gene Symbo	TRAF6	FALSE	TRAF6
taxon:9606	177123	1902	nuclear rece	gene	biological_pr	G:9606:NR2C2				NR2C2	Homo sapier	Gene Symbo	NR2C2	FALSE	NR2C2
taxon:9606	164832	1902	zinc finger AN	gene	biological_pr	G:9606:ZFAND2B				ZFAND2B	Homo sapier	Gene Symbo	ZFAND2B	FALSE	ZFAND2B
taxon:9606	177121	1902	heat shock pr	gene	biological_pr	G:9606:HSP90B1			KEGG-&-1&-	HSP90B1	Homo sapier	Gene Symbo	HSP90B1	FALSE	HSP90B1
taxon:9606	177127	1902	tumor protei	gene	biological_pr	G:9606:TPT1				TPT1	Homo sapier	Gene Symbo	TPT1	FALSE	TPT1
taxon:9606	177124	1902	nuclear rece	gene	biological_pr	G:9606:NR2C1				NR2C1	Homo sapier	Gene Symbo	NR2C1	FALSE	NR2C1
taxon:9606	181227	1902	EWS RNA bin	gene	biological_pr	G:9606:EWSR1			KEGG-&-1&-	EWSR1	Homo sapier	Gene Symbo	EWSR1	FALSE	EWSR1
taxon:9606	177134	1902	tropomyosin	gene	biological_pr	G:9606:TPM3			KEGG-&-1&-	TPM3	Homo sapier	Gene Symbo	TPM3	FALSE	TPM3
taxon:9606	168943	1902	shootin 1	gene	biological_pr	G:9606:SHTN1				SHTN1	Homo sapier	Gene Symbo	SHTN1	FALSE	SHTN1
taxon:9606	177133	1902	tropomyosin	gene	biological_pr	G:9606:TPM4			KEGG-&-1&-	TPM4	Homo sapier	Gene Symbo	TPM4	FALSE	TPM4
taxon:9606	168946	1902	ubiquitin spe	gene	biological_pr	G:9606:USP37				USP37	Homo sapier	Gene Symbo	USP37	FALSE	USP37
taxon:9606	181235	1902	ETS variant 5	gene	biological_pr	G:9606:ETV5			KEGG-&-1&-	ETV5	Homo sapier	Gene Symbo	ETV5	FALSE	ETV5
taxon:9606	177136	1902	tropomyosin	gene	biological_pr	G:9606:TPM1			KEGG-&-1&-	TPM1	Homo sapier	Gene Symbo	TPM1	FALSE	TPM1
taxon:9606	181232	1902	MDS1 and E	gene	biological_pr	G:9606:MECOM			KEGG-&-1&-	MECOM	Homo sapier	Gene Symbo	MECOM	FALSE	MECOM
taxon:9606	177143	1902	tumor protei	gene	biological_pr	G:9606:TP73			KEGG-&-1&-	TP73	Homo sapier	Gene Symbo	TP73	FALSE	TP73
taxon:9606	173044	1902	apolipoprotei	gene	biological_pr	G:9606:APOL2				APOL2	Homo sapier	Gene Symbo	APOL2	FALSE	APOL2
taxon:9606	177146	1902	tumor protei	gene	biological_pr	G:9606:TP53	TP53	TRUE	KEGG-&-1&-	TP53	Homo sapier	Gene Symbo	TP53	FALSE	TP53
taxon:9606	177144	1902	tumor protei	gene	biological_pr	G:9606:TP53BP2			KEGG-&-1&-	TP53BP2	Homo sapier	Gene Symbo	TP53BP2	FALSE	TP53BP2
taxon:9606	181240	1902	ETS proto-on	gene	biological_pr	G:9606:ETS2			KEGG-&-1&-	ETS2	Homo sapier	Gene Symbo	ETS2	FALSE	ETS2
taxon:9606	177145	1902	tumor protei	gene	biological_pr	G:9606:TP53BP1			KEGG-&-1&-	TP53BP1	Homo sapier	Gene Symbo	TP53BP1	FALSE	TP53BP1
taxon:9606	181241	1902	ETS proto-on	gene	biological_pr	G:9606:ETS1			KEGG-&-1&-	ETS1	Homo sapier	Gene Symbo	ETS1	FALSE	ETS1
taxon:9606	177150	1902	topoisomera	gene	biological_pr	G:9606:TOP2A			KEGG-&-1&-	TOP2A	Homo sapier	Gene Symbo	TOP2A	FALSE	TOP2A
taxon:9606	173055	1902	MAF bZIP tra	gene	biological_pr	G:9606:MAFF				MAFF	Homo sapier	Gene Symbo	MAFF	FALSE	MAFF
taxon:9606	177148	1902	topoisomera	gene	biological_pr	G:9606:TOP2B			KEGG-&-1&-	TOP2B	Homo sapier	Gene Symbo	TOP2B	FALSE	TOP2B
taxon:9606	168957	1902	chromodoma	gene	biological_pr	G:9606:CHD8			KEGG-&-1&-	CHD8	Homo sapier	Gene Symbo	CHD8	FALSE	CHD8
taxon:9606	181250	1902	estrogen rec	gene	biological_pr	G:9606:ESR2	ESR2	TRUE	KEGG-&-1&-	ESR2	Homo sapier	Gene Symbo	ESR2	FALSE	ESR2
taxon:9606	177154	1902	tenascin XB	gene	biological_pr	G:9606:TNXB			KEGG-&-1&-	TNXB	Homo sapier	Gene Symbo	TNXB	FALSE	TNXB
taxon:9606	181251	1902	estrogen rec	gene	biological_pr	G:9606:ESR1			KEGG-&-1&-	ESR1	Homo sapier	Gene Symbo	ESR1	FALSE	ESR1
taxon:9606	177153	1902	topoisomera	gene	biological_pr	G:9606:TOP1				TOP1	Homo sapier	Gene Symbo	TOP1	FALSE	TOP1
taxon:9606	181249	1902	estrogen rela	gene	biological_pr	G:9606:ESRRA				ESRRA	Homo sapier	Gene Symbo	ESRRA	FALSE	ESRRA
taxon:9606	181255	1902	fibrillarin	gene	biological_pr	G:9606:FBL			KEGG-&-1&-	FBL	Homo sapier	Gene Symbo	FBL	FALSE	FBL
taxon:9606	177156	1902	tensin 1	gene	biological_pr	G:9606:TNS1				TNS1	Homo sapier	Gene Symbo	TNS1	FALSE	TNS1
taxon:9606	181258	1902	endoplasmic	gene	biological_pr	G:9606:ERN1			KEGG-&-1&-	ERN1	Homo sapier	Gene Symbo	ERN1	FALSE	ERN1
taxon:9606	181259	1902	enhancer of r	gene	biological_pr	G:9606:ERH				ERH	Homo sapier	Gene Symbo	ERH	FALSE	ERH
taxon:9606	181263	1902	ERCC excisio	gene	biological_pr	G:9606:ERCC6			KEGG-&-1&-	ERCC6	Homo sapier	Gene Symbo	ERCC6	FALSE	ERCC6
taxon:9606	177167	1902	TNF receptor	gene	biological_pr	G:9606:TNFRSF1B			KEGG-&-1&-	TNFRSF1B	Homo sapier	Gene Symbo	TNFRSF1B	FALSE	TNFRSF1B
taxon:9606	181260	1902	ERG, ETS tra	gene	biological_pr	G:9606:ERG			KEGG-&-1&-	ERG	Homo sapier	Gene Symbo	ERG	FALSE	ERG
taxon:9606	177170	1902	TNF alpha in	gene	biological_pr	G:9606:TNFAIP3			KEGG-&-1&-	TNFAIP3	Homo sapier	Gene Symbo	TNFAIP3	FALSE	TNFAIP3
taxon:9606	181266	1902	ERCC excisio	gene	biological_pr	G:9606:ERCC3			KEGG-&-1&-	ERCC3	Homo sapier	Gene Symbo	ERCC3	FALSE	ERCC3
taxon:9606	177168	1902	TNF receptor	gene	biological_pr	G:9606:TNFRSF1A			KEGG-&-1&-	TNFRSF1A	Homo sapier	Gene Symbo	TNFRSF1A	FALSE	TNFRSF1A
taxon:9606	177174	1902	tumor necro	gene	biological_pr	G:9606:TNF	TNF	TRUE	KEGG-&-1&-	TNF	Homo sapier	Gene Symbo	TNF	FALSE	TNF
taxon:9606	181271	1902	erb-b2 recep	gene	biological_pr	G:9606:ERBB4			KEGG-&-1&-	ERBB4	Homo sapier	Gene Symbo	ERBB4	FALSE	ERBB4
taxon:9606	181269	1902	ERCC excisio	gene	biological_pr	G:9606:ERCC2			KEGG-&-1&-	ERCC2	Homo sapier	Gene Symbo	ERCC2	FALSE	ERCC2
taxon:9606	181274	1902	nuclear rece	gene	biological_pr	G:9606:NR2F6				NR2F6	Homo sapier	Gene Symbo	NR2F6	FALSE	NR2F6

taxon:9606	181275	1902	epidermal gr	gene	biological_pr	G:9606:EPS15			KEGG-&-1&-l	EPS15	Homo sapier Gene Symbol	EPS15	FALSE	EPS15
taxon:9606	140313	1902	40S ribosom	gene		G:9606:LOC101929876				LOC1019298	Homo sapier Gene Symbol	LOC1019298	FALSE	LOC101929876
taxon:9606	173086	1902	GABA type A	gene	biological_pr	G:9606:GABARAPL1			KEGG-&-1&-l	GABARAPL1	Homo sapier Gene Symbol	GABARAPL1	FALSE	GABARAPL1
taxon:9606	181276	1902	epidermal gr	gene	biological_pr	G:9606:EPS8				EPS8	Homo sapier Gene Symbol	EPS8	FALSE	EPS8
taxon:9606	181277	1902	glutamyl-pro	gene	biological_pr	G:9606:EPRS			KEGG-&-1&-l	EPRS	Homo sapier Gene Symbol	EPRS	FALSE	EPRS
taxon:9606	177184	1902	thymosin bet	gene	biological_pr	G:9606:TMSB4X			KEGG-&-1&-l	TMSB4X	Homo sapier Gene Symbol	TMSB4X	FALSE	TMSB4X
taxon:9606	156704	1902	bolA family r	gene	biological_pr	G:9606:BOLA2				BOLA2	Homo sapier Gene Symbol	BOLA2	FALSE	BOLA2
taxon:9606	164902	1902	phosphatidyl	gene	biological_pr	G:9606:PIGU			KEGG-&-1&-l	PIGU	Homo sapier Gene Symbol	PIGU	FALSE	PIGU
taxon:9606	164903	1902	charged mult	gene	biological_pr	G:9606:CHMP4B			KEGG-&-1&-l	CHMP4B	Homo sapier Gene Symbol	CHMP4B	FALSE	CHMP4B
taxon:9606	181287	1902	EPH receptor	gene	biological_pr	G:9606:EPHB2			KEGG-&-1&-l	EPHB2	Homo sapier Gene Symbol	EPHB2	FALSE	EPHB2
taxon:9606	177188	1902	TATA elemer	gene	biological_pr	G:9606:TMF1				TMF1	Homo sapier Gene Symbol	TMF1	FALSE	TMF1
taxon:9606	160808	1902	keratin 79	gene	biological_pr	G:9606:KRT79				KRT79	Homo sapier Gene Symbol	KRT79	FALSE	KRT79
taxon:9606	181295	1902	stomatin	gene	biological_pr	G:9606:STOM				STOM	Homo sapier Gene Symbol	STOM	FALSE	STOM
taxon:9606	181293	1902	EPH receptor	gene	biological_pr	G:9606:EPHA3			KEGG-&-1&-l	EPHA3	Homo sapier Gene Symbol	EPHA3	FALSE	EPHA3
taxon:9606	169010	1902	mitochondria	gene	biological_pr	G:9606:MRPS14			KEGG-&-1&-l	MRPS14	Homo sapier Gene Symbol	MRPS14	FALSE	MRPS14
taxon:9606	181298	1902	erythrocyte n	gene	biological_pr	G:9606:EPB41L2				EPB41L2	Homo sapier Gene Symbol	EPB41L2	FALSE	EPB41L2
taxon:9606	181302	1902	E1A binding p	gene	biological_pr	G:9606:EP300			KEGG-&-1&-l	EP300	Homo sapier Gene Symbol	EP300	FALSE	EP300
taxon:9606	169012	1902	X-prolyl amir	gene	biological_pr	G:9606:XPNPEP3				XPNPEP3	Homo sapier Gene Symbol	XPNPEP3	FALSE	XPNPEP3
taxon:9606	177204	1902	talin 1	gene	biological_pr	G:9606:TLN1			KEGG-&-1&-l	TLN1	Homo sapier Gene Symbol	TLN1	FALSE	TLN1
taxon:9606	181301	1902	endothelial P	gene	biological_pr	G:9606:EPAS1			KEGG-&-1&-l	EPAS1	Homo sapier Gene Symbol	EPAS1	FALSE	EPAS1
taxon:9606	177210	1902	transducin lik	gene	biological_pr	G:9606:TLE1				TLE1	Homo sapier Gene Symbol	TLE1	FALSE	TLE1
taxon:9606	177215	1902	thymidine kir	gene	biological_pr	G:9606:TK1			KEGG-&-1&-l	TK1	Homo sapier Gene Symbol	TK1	FALSE	TK1
taxon:9606	181311	1902	enolase 1	gene	biological_pr	G:9606:ENO1			KEGG-&-1&-l	ENO1	Homo sapier Gene Symbol	ENO1	FALSE	ENO1
taxon:9606	173116	1902	tripartite mo	gene	biological_pr	G:9606:TRIM29				TRIM29	Homo sapier Gene Symbol	TRIM29	FALSE	TRIM29
taxon:9606	173117	1902	DNA polymer	gene	biological_pr	G:9606:POLA2			KEGG-&-1&-l	POLA2	Homo sapier Gene Symbol	POLA2	FALSE	POLA2
taxon:9606	177218	1902	TIMP metallo	gene	biological_pr	G:9606:TIMP4				TIMP4	Homo sapier Gene Symbol	TIMP4	FALSE	TIMP4
taxon:9606	177219	1902	TIMP metallo	gene	biological_pr	G:9606:TIMP3			KEGG-&-1&-l	TIMP3	Homo sapier Gene Symbol	TIMP3	FALSE	TIMP3
taxon:9606	177223	1902	T-cell lymph	gene	biological_pr	G:9606:TIAM1			KEGG-&-1&-l	TIAM1	Homo sapier Gene Symbol	TIAM1	FALSE	TIAM1
taxon:9606	177220	1902	TIMP metallo	gene	biological_pr	G:9606:TIMP2				TIMP2	Homo sapier Gene Symbol	TIMP2	FALSE	TIMP2
taxon:9606	181317	1902	cortactin	gene	biological_pr	G:9606:CTTN			KEGG-&-1&-l	CTTN	Homo sapier Gene Symbol	CTTN	FALSE	CTTN
taxon:9606	177221	1902	TIMP metallo	gene	biological_pr	G:9606:TIMP1			KEGG-&-1&-l	TIMP1	Homo sapier Gene Symbol	TIMP1	FALSE	TIMP1
taxon:9606	177226	1902	thimet oligo	gene	biological_pr	G:9606:THOP1			KEGG-&-1&-l	THOP1	Homo sapier Gene Symbol	THOP1	FALSE	THOP1
taxon:9606	177224	1902	TIA1 cytotoxi	gene	biological_pr	G:9606:TIAL1				TIAL1	Homo sapier Gene Symbol	TIAL1	FALSE	TIAL1
taxon:9606	177231	1902	thrombospor	gene	biological_pr	G:9606:THBS2			KEGG-&-1&-l	THBS2	Homo sapier Gene Symbol	THBS2	FALSE	THBS2
taxon:9606	181324	1902	emerin	gene	biological_pr	G:9606:EMD			KEGG-&-1&-l	EMD	Homo sapier Gene Symbol	EMD	FALSE	EMD
taxon:9606	177232	1902	thrombospor	gene	biological_pr	G:9606:THBS1			KEGG-&-1&-l	THBS1	Homo sapier Gene Symbol	THBS1	FALSE	THBS1
taxon:9606	177239	1902	TGFB induce	gene	biological_pr	G:9606:TGIF1			KEGG-&-1&-l	TGIF1	Homo sapier Gene Symbol	TGIF1	FALSE	TGIF1
taxon:9606	173140	1902	beta-secreta	gene	biological_pr	G:9606:BACE1			KEGG-&-1&-l	BACE1	Homo sapier Gene Symbol	BACE1	FALSE	BACE1
taxon:9606	169044	1902	brevican	gene	biological_pr	G:9606:BCAN				BCAN	Homo sapier Gene Symbol	BCAN	FALSE	BCAN
taxon:9606	177237	1902	transglutami	gene	biological_pr	G:9606:TGM2			KEGG-&-1&-l	TGM2	Homo sapier Gene Symbol	TGM2	FALSE	TGM2
taxon:9606	181333	1902	E74 like ETS	gene	biological_pr	G:9606:ELF3				ELF3	Homo sapier Gene Symbol	ELF3	FALSE	ELF3
taxon:9606	181338	1902	ELAV like RN	gene	biological_pr	G:9606:ELAVL1			KEGG-&-1&-l	ELAVL1	Homo sapier Gene Symbol	ELAVL1	FALSE	ELAVL1
taxon:9606	169051	1902	trafficking pr	gene	biological_pr	G:9606:TRAPPC11				TRAPPC11	Homo sapier Gene Symbol	TRAPPC11	FALSE	TRAPPC11
taxon:9606	181337	1902	ELAV like RN	gene	biological_pr	G:9606:ELAVL3				ELAVL3	Homo sapier Gene Symbol	ELAVL3	FALSE	ELAVL3
taxon:9606	173151	1902	makorin ring	gene	biological_pr	G:9606:MKRN1				MKRN1	Homo sapier Gene Symbol	MKRN1	FALSE	MKRN1
taxon:9606	164956	1902	nuclear recep	gene	biological_pr	G:9606:NCOA7				NCOA7	Homo sapier Gene Symbol	NCOA7	FALSE	NCOA7
taxon:9606	181341	1902	elastase, neu	gene	biological_pr	G:9606:ELANE			KEGG-&-1&-l	ELANE	Homo sapier Gene Symbol	ELANE	FALSE	ELANE
taxon:9606	177248	1902	transforming	gene	biological_pr	G:9606:TGFB1I1				TGFB1I1	Homo sapier Gene Symbol	TGFB1I1	FALSE	TGFB1I1
taxon:9606	173152	1902	CD2 associat	gene	biological_pr	G:9606:CD2AP			KEGG-&-1&-l	CD2AP	Homo sapier Gene Symbol	CD2AP	FALSE	CD2AP
taxon:9606	177249	1902	transforming	gene	biological_pr	G:9606:TGFB1			KEGG-&-1&-l	TGFB1	Homo sapier Gene Symbol	TGFB1	FALSE	TGFB1
taxon:9606	177254	1902	tissue factor	gene	biological_pr	G:9606:TFPI			KEGG-&-1&-l	TFPI	Homo sapier Gene Symbol	TFPI	FALSE	TFPI
taxon:9606	173159	1902	POZ/BTB anc	gene	biological_pr	G:9606:PATZ1				PATZ1	Homo sapier Gene Symbol	PATZ1	FALSE	PATZ1
taxon:9606	173156	1902	coronin 1C	gene	biological_pr	G:9606:CORO1C				CORO1C	Homo sapier Gene Symbol	CORO1C	FALSE	CORO1C
taxon:9606	177252	1902	transferrin re	gene	biological_pr	G:9606:TFRC			KEGG-&-1&-l	TFRC	Homo sapier Gene Symbol	TFRC	FALSE	TFRC
taxon:9606	181348	1902	eukaryotic tr	gene	biological_pr	G:9606:EIF4G1			KEGG-&-1&-l	EIF4G1	Homo sapier Gene Symbol	EIF4G1	FALSE	EIF4G1
taxon:9606	181354	1902	eukaryotic tr	gene	biological_pr	G:9606:EIF4A2			KEGG-&-1&-l	EIF4A2	Homo sapier Gene Symbol	EIF4A2	FALSE	EIF4A2
taxon:9606	181355	1902	eukaryotic tr	gene	biological_pr	G:9606:EIF4A1			KEGG-&-1&-l	EIF4A1	Homo sapier Gene Symbol	EIF4A1	FALSE	EIF4A1
taxon:9606	177257	1902	trefoil factor	gene	biological_pr	G:9606:TFF1			KEGG-&-1&-l	TFF1	Homo sapier Gene Symbol	TFF1	FALSE	TFF1
taxon:9606	160873	1902	NLR family p	gene	biological_pr	G:9606:NLRP10				NLRP10	Homo sapier Gene Symbol	NLRP10	FALSE	NLRP10
taxon:9606	181359	1902	eukaryotic tr	gene	biological_pr	G:9606:EIF2S1			KEGG-&-1&-l	EIF2S1	Homo sapier Gene Symbol	EIF2S1	FALSE	EIF2S1
taxon:9606	164972	1902	NudC domair	gene	biological_pr	G:9606:NUDCD2				NUDCD2	Homo sapier Gene Symbol	NUDCD2	FALSE	NUDCD2
taxon:9606	169069	1902	GUF1 homol	gene	biological_pr	G:9606:GUF1				GUF1	Homo sapier Gene Symbol	GUF1	FALSE	GUF1
taxon:9606	177266	1902	MAF bZIP tra	gene	biological_pr	G:9606:MAFK				MAFK	Homo sapier Gene Symbol	MAFK	FALSE	MAFK
taxon:9606	181361	1902	enoyl-CoA hy	gene	biological_pr	G:9606:EHHADH			KEGG-&-1&-l	EHHADH	Homo sapier Gene Symbol	EHHADH	FALSE	EHHADH
taxon:9606	181367	1902	epidermal gr	gene	biological_pr	G:9606:EGFR			KEGG-&-1&-l	EGFR	Homo sapier Gene Symbol	EGFR	FALSE	EGFR
taxon:9606	177269	1902	aminoacyl tR	gene	biological_pr	G:9606:AIMP2				AIMP2	Homo sapier Gene Symbol	AIMP2	FALSE	AIMP2
taxon:9606	181365	1902	early growth	gene	biological_pr	G:9606:EGR1			KEGG-&-1&-l	EGR1	Homo sapier Gene Symbol	EGR1	FALSE	EGR1
taxon:9606	160891	1902	tripartite mo	gene	biological_pr	G:9606:TRIM59				TRIM59	Homo sapier Gene Symbol	TRIM59	FALSE	TRIM59
taxon:9606	177275	1902	ubiquitin C	gene	biological_pr	G:9606:UBC			KEGG-&-1&-l	UBC	Homo sapier Gene Symbol	UBC	FALSE	UBC
taxon:9606	181371	1902	cadherin EGF	gene	biological_pr	G:9606:CELSR2				CELSR2	Homo sapier Gene Symbol	CELSR2	FALSE	CELSR2
taxon:9606	177272	1902	ubiquitin con	gene	biological_pr	G:9606:UBE2A			KEGG-&-1&-l	UBE2A	Homo sapier Gene Symbol	UBE2A	FALSE	UBE2A
taxon:9606	177278	1902	transcription	gene	biological_pr	G:9606:TFDP1			KEGG-&-1&-l	TFDP1	Homo sapier Gene Symbol	TFDP1	FALSE	TFDP1
taxon:9606	173182	1902	zinc finger pr	gene	biological_pr	G:9606:ZNF346				ZNF346	Homo sapier Gene Symbol	ZNF346	FALSE	ZNF346
taxon:9606	177279	1902	nuclear recep	gene	biological_pr	G:9606:NR2F2				NR2F2	Homo sapier Gene Symbol	NR2F2	FALSE	NR2F2
taxon:9606	173180	1902	peptidyl argi	gene	biological_pr	G:9606:PADI4				PADI4	Homo sapier Gene Symbol	PADI4	FALSE	PADI4
taxon:9606	177282	1902	transcription	gene	biological_pr	G:9606:TFAP4			KEGG-&-1&-l	TFAP4	Homo sapier Gene Symbol	TFAP4	FALSE	TFAP4
taxon:9606	177283	1902	transcription	gene	biological_pr	G:9606:TFAP2C				TFAP2C	Homo sapier Gene Symbol	TFAP2C	FALSE	TFAP2C
taxon:9606	173187	1902	GTP binding p	gene	biological_pr	G:9606:GTPBP4			KEGG-&-1&-l	GTPBP4	Homo sapier Gene Symbol	GTPBP4	FALSE	GTPBP4
taxon:9606	173184	1902	dimethylargi	gene	biological_pr	G:9606:DDAH2				DDAH2	Homo sapier Gene Symbol	DDAH2	FALSE	DDAH2
taxon:9606	181380	1902	AT-rich intera	gene	biological_pr	G:9606:ARID3A				ARID3A	Homo sapier Gene Symbol	ARID3A	FALSE	ARID3A
taxon:9606	177284	1902	transcription	gene	biological_pr	G:9606:TFAP2B				TFAP2B	Homo sapier Gene Symbol	TFAP2B	FALSE	TFAP2B
taxon:9606	177285	1902	transcription	gene	biological_pr	G:9606:TFAP2A				TFAP2A	Homo sapier Gene Symbol	TFAP2A	FALSE	TFAP2A
taxon:9606	173192	1902	tetraspanin 1	gene	biological_pr	G:9606:TSPAN15				TSPAN15	Homo sapier Gene Symbol	TSPAN15	FALSE	TSPAN15
taxon:9606	169097	1902	THO complex	gene	biological_pr	G:9606:THOC2			KEGG-&-1&-l	THOC2	Homo sapier Gene Symbol	THOC2	FALSE	THOC2
taxon:9606	160911	1902	MAGE family	gene	cellular_com	G:9606:MAGEB18				MAGEB18	Homo sapier Gene Symbol	MAGEB18	FALSE	MAGEB18
taxon:9606	177293	1902	telomerase a	gene	biological_pr	G:9606:TEP1				TEP1	Homo sapier Gene Symbol	TEP1	FALSE	TEP1
taxon:9606	177298	1902	tec protein ty	gene	biological_pr	G:9606:TEC			KEGG-&-1&-l	TEC	Homo sapier Gene Symbol	TEC	FALSE	TEC
taxon:9606	169105	1902	zinc finger M	gene	biological_pr	G:9606:ZMIZ1				ZMIZ1	Homo sapier Gene Symbol	ZMIZ1	FALSE	ZMIZ1
taxon:9606	181399	1902	diphthamide	gene	biological_pr	G:9606:DPH1				DPH1	Homo sapier Gene Symbol	DPH1	FALSE	DPH1
taxon:9606	177303	1902	peroxiredoxin	gene	biological_pr	G:9606:PRDX2				PRDX2	Homo sapier Gene Symbol	PRDX2	FALSE	PRDX2
taxon:9606	173204	1902	RNA binding	gene	biological_pr	G:9606:RBFOX2				RBFOX2	Homo sapier Gene Symbol	RBFOX2	FALSE	RBFOX2
taxon:9606	177307	1902	thymine DNA	gene	biological_pr	G:9606:TDG			KEGG-&-1&-l	TDG	Homo sapier Gene Symbol	TDG	FALSE	TDG
taxon:9606	173211	1902	transportin 3	gene	biological_pr	G:9606:TNPO3				TNPO3	Homo sapier Gene Symbol	TNPO3	FALSE	TNPO3
taxon:9606	177308	1902	dynein light c	gene	biological_pr	G:9606:DYNLT1				DYNLT1	Homo sapier Gene Symbol	DYNLT1	FALSE	DYNLT1
taxon:9606	181411	1902	DNA methylt	gene	biological_pr	G:9606:DNMT1			KEGG-&-1&-l	DNMT1	Homo sapier Gene Symbol	DNMT1	FALSE	DNMT1
taxon:9606	181408	1902	DNA methylt	gene	biological_pr	G:9606:DNMT3B			KEGG-&-1&-l	DNMT3B	Homo sapier Gene Symbol	DNMT3B	FALSE	DNMT3B
taxon:9606	169121	1902	SMAD specifi	gene	biological_pr	G:9606:SMURF1			KEGG-&-1&-l	SMURF1	Homo sapier Gene Symbol	SMURF1	FALSE	SMURF1
taxon:9606	173217	1902	ribosomal pr	gene	biological_pr	G:9606:RPL13A			KEGG-&-1&-l	RPL13A	Homo sapier Gene Symbol	RPL13A	FALSE	RPL13A
taxon:9606	173226	1902	SUZ12 polyc	gene	biological_pr	G:9606:SUZ12				SUZ12	Homo sapier Gene Symbol	SUZ12	FALSE	SUZ12
taxon:9606	173227	1902	nucleoporin 1	gene	biological_pr	G:9606:NUP188			KEGG-&-1&-l	NUP188	Homo sapier Gene Symbol	NUP188	FALSE	NUP188
taxon:9606	181416	1902	fibroblast gr	gene	biological_pr	G:9606:FGF10			KEGG-&-1&-l	FGF10	Homo sapier Gene Symbol	FGF10	FALSE	FGF10
taxon:9606	173225	1902	scribbled pla	gene	biological_pr	G:9606:SCRIB			KEGG-&-1&-l	SCRIB	Homo sapier Gene Symbol	SCRIB	FALSE	SCRIB
taxon:9606	181429	1902	FES proto-on	gene	biological_pr	G:9606:FES			KEGG-&-1&-l	FES	Homo sapier Gene Symbol	FES	FALSE	FES
taxon:9606	173237	1902	microtubule-	gene	biological_pr	G:9606:MACF1				MACF1	Homo sapier Gene Symbol	MACF1	FALSE	MACF1
taxon:9606	173240	1902	hes related f	gene	biological_pr	G:9606:HEY2			KEGG-&-1&-l	HEY2	Homo sapier Gene Symbol	HEY2	FALSE	HEY2
taxon:9606	165048	1902	histone clust	gene	biological_pr	G:9606:HIST2H3C			KEGG-&-1&-l	HIST2H3C	Homo sapier Gene Symbol	HIST2H3C	FALSE	HIST2H3C
taxon:9606	169148	1902	golgi associa	gene	biological_pr	G:9606:GOPC				GOPC	Homo sapier Gene Symbol	GOPC	FALSE	GOPC
taxon:9606	173245	1902	pescadillo rib	gene	biological_pr	G:9606:PES1				PES1	Homo sapier Gene Symbol	PES1	FALSE	PES1
taxon:9606	173250	1902	l(3)mbt-like	gene	biological_pr	G:9606:L3MBTL1				L3MBTL1	Homo sapier Gene Symbol	L3MBTL1	FALSE	L3MBTL1
taxon:9606	165063	1902	Src homology	gene	biological_pr	G:9606:SHE				SHE	Homo sapier Gene Symbol	SHE	FALSE	SHE
taxon:9606	173255	1902	triokinase an	gene	biological_pr	G:9606:TKFC			KEGG-&-1&-l	TKFC	Homo sapier Gene Symbol	TKFC	FALSE	TKFC
taxon:9606	177348	1902	t-complex 1	gene	biological_pr	G:9606:TCP1				TCP1	Homo sapier Gene Symbol	TCP1	FALSE	TCP1
taxon:9606	169157	1902	REX4 homolo	gene	biological_pr	G:9606:REXO4				REXO4	Homo sapier Gene Symbol	REXO4	FALSE	REXO4
taxon:9606	173257	1902	golgi reasser	gene	biological_pr	G:9606:GORASP2				GORASP2	Homo sapier Gene Symbol	GORASP2	FALSE	GORASP2
taxon:9606	177356	1902	transcription	gene	biological_pr	G:9606:TCF20				TCF20	Homo sapier Gene Symbol	TCF20	FALSE	TCF20
taxon:9606	177362	1902	zinc finger E-	gene	biological_pr	G:9606:ZEB1			KEGG-&-1&-l	ZEB1	Homo sapier Gene Symbol	ZEB1	FALSE	ZEB1
taxon:9606	181458	1902	dynamin 2	gene	biological_pr	G:9606:DNM2			KEGG-&-1&-l	DNM2	Homo sapier Gene Symbol	DNM2	FALSE	DNM2
taxon:9606	173267	1902	histone H4 tr	gene	biological_pr	G:9606:HINFP				HINFP	Homo sapier Gene Symbol	HINFP	FALSE	HINFP
taxon:9606	177363	1902	transcription	gene	biological_pr	G:9606:TCF7L2			KEGG-&-1&-l	TCF7L2	Homo sapier Gene Symbol	TCF7L2	FALSE	TCF7L2
taxon:9606	181462	1902	dynein cytopl	gene	biological_pr	G:9606:DYNC1I1			KEGG-&-1&-l	DYNC1I1	Homo sapier Gene Symbol	DYNC1I1	FALSE	DYNC1I1
taxon:9606	160982	1902	FRAS1 relate	gene	biological_pr	G:9606:FREM2				FREM2	Homo sapier Gene Symbol	FREM2	FALSE	FREM2
taxon:9606	181463	1902	dynein cytopl	gene	biological_pr	G:9606:DYNC1H1			KEGG-&-1&-l	DYNC1H1	Homo sapier Gene Symbol	DYNC1H1	FALSE	DYNC1H1
taxon:9606	177367	1902	HNF1 homeo	gene	biological_pr	G:9606:HNF1A			KEGG-&-1&-l	HNF1A	Homo sapier Gene Symbol	HNF1A	FALSE	HNF1A
taxon:9606	177365	1902	transcription	gene	biological_pr	G:9606:TCF3			KEGG-&-1&-l	TCF3	Homo sapier Gene Symbol	TCF3	FALSE	TCF3
taxon:9606	177371	1902	elongin B	gene	biological_pr	G:9606:ELOB			KEGG-&-1&-l	ELOB	Homo sapier Gene Symbol	ELOB	FALSE	ELOB
taxon:9606	169176	1902	angiotensin I	gene	biological_pr	G:9606:AGTRAP				AGTRAP	Homo sapier Gene Symbol	AGTRAP	FALSE	AGTRAP
taxon:9606	177369	1902	transcription	gene	biological_pr	G:9606:TCF4				TCF4	Homo sapier Gene Symbol	TCF4	FALSE	TCF4
taxon:9606	177375	1902	transcription	gene	biological_pr	G:9606:TCEA2				TCEA2	Homo sapier Gene Symbol	TCEA2	FALSE	TCEA2
taxon:9606	177373	1902	elongin C	gene	biological_pr	G:9606:ELOC			KEGG-&-1&-l	ELOC	Homo sapier Gene Symbol	ELOC	FALSE	ELOC
taxon:9606	173287	1902	KN motif anc	gene	biological_pr	G:9606:KANK2				KANK2	Homo sapier Gene Symbol	KANK2	FALSE	KANK2
taxon:9606	181477	1902	dynamin 1	gene	biological_pr	G:9606:DNM1			KEGG-&-1&-l	DNM1	Homo sapier Gene Symbol	DNM1	FALSE	DNM1
taxon:9606	177385	1902	TATA-box bir	gene	biological_pr	G:9606:TBP			KEGG-&-1&-l	TBP	Homo sapier Gene Symbol	TBP	FALSE	TBP
taxon:9606	173294	1902	zinc finger pr	gene	biological_pr	G:9606:ZNF385A				ZNF385A	Homo sapier Gene Symbol	ZNF385A	FALSE	ZNF385A
taxon:9606	173297	1902	SIN3 transcri	gene	biological_pr	G:9606:SIN3A			KEGG-&-1&-l	SIN3A	Homo sapier Gene Symbol	SIN3A	FALSE	SIN3A
taxon:9606	177396	1902	threonyl-tRN	gene	biological_pr	G:9606:TARS			KEGG-&-1&-l	TARS	Homo sapier Gene Symbol	TARS	FALSE	TARS
taxon:9606	148734	1902	testis specifi	gene	biological_pr	G:9606:TSPY10				TSPY10	Homo sapier Gene Symbol	TSPY10	FALSE	TSPY10
taxon:9606	177407	1902	TAL bHLH tra	gene	biological_pr	G:9606:TAL1				TAL1	Homo sapier Gene Symbol	TAL1	FALSE	TAL1
taxon:9606	181503	1902	diaphanous r	gene	biological_pr	G:9606:DIAPH1			KEGG-&-1&-l	DIAPH1	Homo sapier Gene Symbol	DIAPH1	FALSE	DIAPH1
taxon:9606	177405	1902	transaldolase	gene	biological_pr	G:9606:TALDO1			KEGG-&-1&-l	TALDO1	Homo sapier Gene Symbol	TALDO1	FALSE	TALDO1
taxon:9606	181504	1902	NAD(P)H qui	gene	biological_pr	G:9606:NQO1			KEGG-&-1&-l	NQO1	Homo sapier Gene Symbol	NQO1	FALSE	NQO1
taxon:9606	177408	1902	mitogen-acti	gene	biological_pr	G:9606:MAP3K7			KEGG-&-1&-l	MAP3K7	Homo sapier Gene Symbol	MAP3K7	FALSE	MAP3K7
taxon:9606	177409	1902	TATA-box bir	gene	biological_pr	G:9606:TAF13			KEGG-&-1&-l	TAF13	Homo sapier Gene Symbol	TAF13	FALSE	TAF13
taxon:9606	181510	1902	24-dehydroch	gene	biological_pr	G:9606:DHCR24			KEGG-&-1&-l	DHCR24	Homo sapier Gene Symbol	DHCR24	FALSE	DHCR24
taxon:9606	177415	1902	TATA-box bir	gene	biological_pr	G:9606:TAF6			KEGG-&-1&-l	TAF6	Homo sapier Gene Symbol	TAF6	FALSE	TAF6
taxon:9606	177412	1902	TATA-box bir	gene	biological_pr	G:9606:TAF10			KEGG-&-1&-l	TAF10	Homo sapier Gene Symbol	TAF10	FALSE	TAF10
taxon:9606	177413	1902	TATA-box bir	gene	biological_pr	G:9606:TAF9			KEGG-&-1&-l	TAF9	Homo sapier Gene Symbol	TAF9	FALSE	TAF9
taxon:9606	181509	1902	dihydrofolate	gene	biological_pr	G:9606:DHFR			KEGG-&-1&-l	DHFR	Homo sapier Gene Symbol	DHFR	FALSE	DHFR
taxon:9606	144651	1902	TNFAIP8L2-S	gene		G:9606:TNFAIP8L2-SCNM1				TNFAIP8L2-S	Homo sapier Gene Symbol	TNFAIP8L2-S	FALSE	TNFAIP8L2-SCNM1
taxon:9606	177416	1902	TATA-box bir	gene	biological_pr	G:9606:TAF5			KEGG-&-1&-l	TAF5	Homo sapier Gene Symbol	TAF5	FALSE	TAF5
taxon:9606	177422	1902	transcription	gene	biological_pr	G:9606:TADA2A				TADA2A	Homo sapier Gene Symbol	TADA2A	FALSE	TADA2A
taxon:9606	165132	1902	ribonucleopr	gene	biological_pr	G:9606:RAVER1				RAVER1	Homo sapier Gene Symbol	RAVER1	FALSE	RAVER1
taxon:9606	177420	1902	TATA-box bir	gene	biological_pr	G:9606:TAF2			KEGG-&-1&-l	TAF2	Homo sapier Gene Symbol	TAF2	FALSE	TAF2
taxon:9606	177421	1902	TATA-box bir	gene	biological_pr	G:9606:TAF1			KEGG-&-1&-l	TAF1	Homo sapier Gene Symbol	TAF1	FALSE	TAF1
taxon:9606	173330	1902	ring finger ar	gene	biological_pr	G:9606:RCHY1			KEGG-&-1&-l	RCHY1	Homo sapier Gene Symbol	RCHY1	FALSE	RCHY1
taxon:9606	169235	1902	ataxin 7 like	gene	biological_pr	G:9606:ATXN7L3				ATXN7L3	Homo sapier Gene Symbol	ATXN7L3	FALSE	ATXN7L3
taxon:9606	177425	1902	ADAM metal	gene	biological_pr	G:9606:ADAM17			KEGG-&-1&-l	ADAM17	Homo sapier Gene Symbol	ADAM17	FALSE	ADAM17
taxon:9606	177433	1902	synaptotagm	gene	biological_pr	G:9606:SYT1			KEGG-&-1&-l	SYT1	Homo sapier Gene Symbol	SYT1	FALSE	SYT1
taxon:9606	177439	1902	spleen assoc	gene	biological_pr	G:9606:SYK			KEGG-&-1&-l	SYK	Homo sapier Gene Symbol	SYK	FALSE	SYK
taxon:9606	169249	1902	SET and MYN	gene	biological_pr	G:9606:SMYD2				SMYD2	Homo sapier Gene Symbol	SMYD2	FALSE	SMYD2
taxon:9606	169254	1902	mitochondria	gene	biological_pr	G:9606:MRPS22				MRPS22	Homo sapier Gene Symbol	MRPS22	FALSE	MRPS22
taxon:9606	177446	1902	suppressor o	gene	biological_pr	G:9606:SUV39H1			KEGG-&-1&-l	SUV39H1	Homo sapier Gene Symbol	SUV39H1	FALSE	SUV39H1
taxon:9606	181540	1902	2,4-dienoyl-C	gene	biological_pr	G:9606:DECR1				DECR1	Homo sapier Gene Symbol	DECR1	FALSE	DECR1
taxon:9606	173348	1902	ribosomal pr	gene	biological_pr	G:9606:RPL36			KEGG-&-1&-l	RPL36	Homo sapier Gene Symbol	RPL36	FALSE	RPL36
taxon:9606	177445	1902	supervillin	gene	biological_pr	G:9606:SVIL				SVIL	Homo sapier Gene Symbol	SVIL	FALSE	SVIL
taxon:9606	181541	1902	DEAH-box he	gene	biological_pr	G:9606:DHX15			KEGG-&-1&-l	DHX15	Homo sapier Gene Symbol	DHX15	FALSE	DHX15
taxon:9606	181545	1902	DExH-box hel	gene	biological_pr	G:9606:DHX9				DHX9	Homo sapier Gene Symbol	DHX9	FALSE	DHX9
taxon:9606	177454	1902	SPT6 homolo	gene	biological_pr	G:9606:SUPT6H				SUPT6H	Homo sapier Gene Symbol	SUPT6H	FALSE	SUPT6H
taxon:9606	181550	1902	DEAD-box he	gene	biological_pr	G:9606:DDX3X			KEGG-&-1&-l	DDX3X	Homo sapier Gene Symbol	DDX3X	FALSE	DDX3X
taxon:9606	169262	1902	SWI/SNF-rel	gene	biological_pr	G:9606:SMARCAD1			KEGG-&-1&-l	SMARCAD1	Homo sapier Gene Symbol	SMARCAD1	FALSE	SMARCAD1
taxon:9606	181551	1902	DEAD-box he	gene	biological_pr	G:9606:DDX1				DDX1	Homo sapier Gene Symbol	DDX1	FALSE	DDX1
taxon:9606	181548	1902	DEAD-box he	gene	biological_pr	G:9606:DDX6			KEGG-&-1&-l	DDX6	Homo sapier Gene Symbol	DDX6	FALSE	DDX6
taxon:9606	181549	1902	DEAD-box he	gene	biological_pr	G:9606:DDX5			KEGG-&-1&-l	DDX5	Homo sapier Gene Symbol	DDX5	FALSE	DDX5
taxon:9606	177462	1902	sterol regula	gene	biological_pr	G:9606:SREBF2				SREBF2	Homo sapier Gene Symbol	SREBF2	FALSE	SREBF2
taxon:9606	177463	1902	sterol regula	gene	biological_pr	G:9606:SREBF1			KEGG-&-1&-l	SREBF1	Homo sapier Gene Symbol	SREBF1	FALSE	SREBF1
taxon:9606	181559	1902	dopa decarb	gene	biological_pr	G:9606:DDC			KEGG-&-1&-l	DDC	Homo sapier Gene Symbol	DDC	FALSE	DDC
taxon:9606	181556	1902	growth arres	gene	biological_pr	G:9606:GADD45A			KEGG-&-1&-l	GADD45A	Homo sapier Gene Symbol	GADD45A	FALSE	GADD45A
taxon:9606	177460	1902	spermidine s	gene	biological_pr	G:9606:SRM			KEGG-&-1&-l	SRM	Homo sapier Gene Symbol	SRM	FALSE	SRM
taxon:9606	177466	1902	sorcin	gene	biological_pr	G:9606:SRI				SRI	Homo sapier Gene Symbol	SRI	FALSE	SRI
taxon:9606	173368	1902	anti-silencing	gene	biological_pr	G:9606:ASF1A				ASF1A	Homo sapier Gene Symbol	ASF1A	FALSE	ASF1A
taxon:9606	181560	1902	damage spec	gene	biological_pr	G:9606:DDB2			KEGG-&-1&-l	DDB2	Homo sapier Gene Symbol	DDB2	FALSE	DDB2
taxon:9606	181561	1902	damage spec	gene	biological_pr	G:9606:DDB1			KEGG-&-1&-l	DDB1	Homo sapier Gene Symbol	DDB1	FALSE	DDB1
taxon:9606	177465	1902	aldo-keto red	gene	biological_pr	G:9606:AKR1D1			KEGG-&-1&-l	AKR1D1	Homo sapier Gene Symbol	AKR1D1	FALSE	AKR1D1
taxon:9606	177469	1902	SRC proto-on	gene	biological_pr	G:9606:SRC			KEGG-&-1&-l	SRC	Homo sapier Gene Symbol	SRC	FALSE	SRC
taxon:9606	181571	1902	DCC netrin 1	gene	biological_pr	G:9606:DCC			KEGG-&-1&-l	DCC	Homo sapier Gene Symbol	DCC	FALSE	DCC
taxon:9606	181568	1902	decorin	gene	biological_pr	G:9606:DCN			KEGG-&-1&-l	DCN	Homo sapier Gene Symbol	DCN	FALSE	DCN
taxon:9606	173377	1902	HECT domair	gene	biological_pr	G:9606:HECTD1				HECTD1	Homo sapier Gene Symbol	HECTD1	FALSE	HECTD1
taxon:9606	181574	1902	drebrin 1	gene	biological_pr	G:9606:DBN1				DBN1	Homo sapier Gene Symbol	DBN1	FALSE	DBN1
taxon:9606	173380	1902	thioredoxin 2	gene	biological_pr	G:9606:TXN2			KEGG-&-1&-l	TXN2	Homo sapier Gene Symbol	TXN2	FALSE	TXN2
taxon:9606	181579	1902	BMP/retinoic	gene	biological_pr	G:9606:BRINP1				BRINP1	Homo sapier Gene Symbol	BRINP1	FALSE	BRINP1
taxon:9606	181582	1902	death domai	gene	biological_pr	G:9606:DAXX			KEGG-&-1&-l	DAXX	Homo sapier Gene Symbol	DAXX	FALSE	DAXX
taxon:9606	177487	1902	secreted pho	gene	biological_pr	G:9606:SPP1			KEGG-&-1&-l	SPP1	Homo sapier Gene Symbol	SPP1	FALSE	SPP1
taxon:9606	181583	1902	aspartyl-tRN	gene	biological_pr	G:9606:DARS			KEGG-&-1&-l	DARS	Homo sapier Gene Symbol	DARS	FALSE	DARS
taxon:9606	169298	1902	UV stimulate	gene	biological_pr	G:9606:UVSSA				UVSSA	Homo sapier Gene Symbol	UVSSA	FALSE	UVSSA
taxon:9606	181584	1902	death associ	gene	biological_pr	G:9606:DAPK3			KEGG-&-1&-l	DAPK3	Homo sapier Gene Symbol	DAPK3	FALSE	DAPK3
taxon:9606	177488	1902	SPARC/osteo	gene	biological_pr	G:9606:SPOCK1				SPOCK1	Homo sapier Gene Symbol	SPOCK1	FALSE	SPOCK1
taxon:9606	181585	1902	death associ	gene	biological_pr	G:9606:DAPK1			KEGG-&-1&-l	DAPK1	Homo sapier Gene Symbol	DAPK1	FALSE	DAPK1
taxon:9606	177495	1902	thyroid horm	gene	biological_pr	G:9606:THRSP				THRSP	Homo sapier Gene Symbol	THRSP	FALSE	THRSP
taxon:9606	177492	1902	TIA1 cytotoxi	gene	biological_pr	G:9606:TIA1				TIA1	Homo sapier Gene Symbol	TIA1	FALSE	TIA1
taxon:9606	177496	1902	thyroid horm	gene	biological_pr	G:9606:THRB			KEGG-&-1&-l	THRB	Homo sapier Gene Symbol	THRB	FALSE	THRB
taxon:9606	181592	1902	dystroglycan	gene	biological_pr	G:9606:DAG1			KEGG-&-1&-l	DAG1	Homo sapier Gene Symbol	DAG1	FALSE	DAG1
taxon:9606	173401	1902	SAM pointed	gene	biological_pr	G:9606:SPDEF				SPDEF	Homo sapier Gene Symbol	SPDEF	FALSE	SPDEF
taxon:9606	177497	1902	thyroid horm	gene	biological_pr	G:9606:THRA			KEGG-&-1&-l	THRA	Homo sapier Gene Symbol	THRA	FALSE	THRA
taxon:9606	169311	1902	E1A binding p	gene	biological_pr	G:9606:EP400				EP400	Homo sapier Gene Symbol	EP400	FALSE	EP400
taxon:9606	181596	1902	DAB2, clathri	gene	biological_pr	G:9606:DAB2			KEGG-&-1&-l	DAB2	Homo sapier Gene Symbol	DAB2	FALSE	DAB2
taxon:9606	177506	1902	secreted prot	gene	biological_pr	G:9606:SPARC				SPARC	Homo sapier Gene Symbol	SPARC	FALSE	SPARC
taxon:9606	173411	1902	bromodomai	gene	biological_pr	G:9606:BRD4				BRD4	Homo sapier Gene Symbol	BRD4	FALSE	BRD4
taxon:9606	173415	1902	translocation	gene	biological_pr	G:9606:TRAM1			KEGG-&-1&-l	TRAM1	Homo sapier Gene Symbol	TRAM1	FALSE	TRAM1
taxon:9606	173413	1902	ETHE1, persu	gene	biological_pr	G:9606:ETHE1			KEGG-&-1&-l	ETHE1	Homo sapier Gene Symbol	ETHE1	FALSE	ETHE1
taxon:9606	177515	1902	Sp1 transcrip	gene	biological_pr	G:9606:SP1			KEGG-&-1&-l	SP1	Homo sapier Gene Symbol	SP1	FALSE	SP1
taxon:9606	169320	1902	zinc finger a	gene	biological_pr	G:9606:ZBTB2				ZBTB2	Homo sapier Gene Symbol	ZBTB2	FALSE	ZBTB2
taxon:9606	173416	1902	PHD finger p	gene	biological_pr	G:9606:PHF3				PHF3	Homo sapier Gene Symbol	PHF3	FALSE	PHF3
taxon:9606	177513	1902	Sp3 transcrip	gene	biological_pr	G:9606:SP3				SP3	Homo sapier Gene Symbol	SP3	FALSE	SP3
taxon:9606	177523	1902	SRY-box 4	gene	biological_pr	G:9606:SOX4			KEGG-&-1&-l	SOX4	Homo sapier Gene Symbol	SOX4	FALSE	SOX4
taxon:9606	169338	1902	WD repeat d	gene	biological_pr	G:9606:WDR48			KEGG-&-1&-l	WDR48	Homo sapier Gene Symbol	WDR48	FALSE	WDR48
taxon:9606	169336	1902	ubiquitin spe	gene	biological_pr	G:9606:USP36				USP36	Homo sapier Gene Symbol	USP36	FALSE	USP36
taxon:9606	177529	1902	sortilin relat	gene	biological_pr	G:9606:SORL1				SORL1	Homo sapier Gene Symbol	SORL1	FALSE	SORL1
taxon:9606	173441	1902	TAR DNA bin	gene	biological_pr	G:9606:TARDBP				TARDBP	Homo sapier Gene Symbol	TARDBP	FALSE	TARDBP
taxon:9606	177543	1902	small nuclea	gene	biological_pr	G:9606:SNRPN				SNRPN	Homo sapier Gene Symbol	SNRPN	FALSE	SNRPN
taxon:9606	173447	1902	RING1 and Y	gene	biological_pr	G:9606:RYBP				RYBP	Homo sapier Gene Symbol	RYBP	FALSE	RYBP
taxon:9606	177547	1902	small nuclea	gene	biological_pr	G:9606:SNRPD3			KEGG-&-1&-l	SNRPD3	Homo sapier Gene Symbol	SNRPD3	FALSE	SNRPD3
taxon:9606	173449	1902	glutamate re	gene	biological_pr	G:9606:GRIP1				GRIP1	Homo sapier Gene Symbol	GRIP1	FALSE	GRIP1
taxon:9606	181644	1902	CD44 molecu	gene	biological_pr	G:9606:CD44			KEGG-&-1&-l	CD44	Homo sapier Gene Symbol	CD44	FALSE	CD44
taxon:9606	177548	1902	small nuclea	gene	biological_pr	G:9606:SNRPD2			KEGG-&-1&-l	SNRPD2	Homo sapier Gene Symbol	SNRPD2	FALSE	SNRPD2
taxon:9606	181645	1902	CD40 ligand	gene	biological_pr	G:9606:CD40LG			KEGG-&-1&-l	CD40LG	Homo sapier Gene Symbol	CD40LG	FALSE	CD40LG
taxon:9606	177549	1902	small nuclea	gene	biological_pr	G:9606:SNRPD1			KEGG-&-1&-l	SNRPD1	Homo sapier Gene Symbol	SNRPD1	FALSE	SNRPD1
taxon:9606	177555	1902	small nuclea	gene	biological_pr	G:9606:SNRNP70			KEGG-&-1&-l	SNRNP70	Homo sapier Gene Symbol	SNRNP70	FALSE	SNRNP70
taxon:9606	177553	1902	small nuclea	gene	biological_pr	G:9606:SNRPA1			KEGG-&-1&-l	SNRPA1	Homo sapier Gene Symbol	SNRPA1	FALSE	SNRPA1
taxon:9606	177556	1902	fascin actin-b	gene	biological_pr	G:9606:FSCN1			KEGG-&-1&-l	FSCN1	Homo sapier Gene Symbol	FSCN1	FALSE	FSCN1
taxon:9606	173461	1902	sirtuin 1	gene	biological_pr	G:9606:SIRT1	SIRT1	TRUE	KEGG-&-1&-l	SIRT1	Homo sapier Gene Symbol	SIRT1	FALSE	SIRT1
taxon:9606	181662	1902	CD86 molecu	gene	biological_pr	G:9606:CD86			KEGG-&-1&-l	CD86	Homo sapier Gene Symbol	CD86	FALSE	CD86
taxon:9606	177567	1902	small ubiquit	gene	biological_pr	G:9606:SUMO2			KEGG-&-1&-l	SUMO2	Homo sapier Gene Symbol	SUMO2	FALSE	SUMO2
taxon:9606	177565	1902	snail family t	gene	biological_pr	G:9606:SNAI1			KEGG-&-1&-l	SNAI1	Homo sapier Gene Symbol	SNAI1	FALSE	SNAI1
taxon:9606	177574	1902	survival of m	gene	biological_pr	G:9606:SMN1			KEGG-&-1&-l	SMN1	Homo sapier Gene Symbol	SMN1	FALSE	SMN1
taxon:9606	177575	1902	SWI/SNF rel	gene	biological_pr	G:9606:SMARCE1			KEGG-&-1&-l	SMARCE1	Homo sapier Gene Symbol	SMARCE1	FALSE	SMARCE1
taxon:9606	177573	1902	survival of m	gene	biological_pr	G:9606:SMN2			KEGG-&-1&-l	SMN2	Homo sapier Gene Symbol	SMN2	FALSE	SMN2
taxon:9606	177578	1902	SWI/SNF rel	gene	biological_pr	G:9606:SMARCD1			KEGG-&-1&-l	SMARCD1	Homo sapier Gene Symbol	SMARCD1	FALSE	SMARCD1
taxon:9606	177577	1902	SWI/SNF rel	gene	biological_pr	G:9606:SMARCD2			KEGG-&-1&-l	SMARCD2	Homo sapier Gene Symbol	SMARCD2	FALSE	SMARCD2
taxon:9606	173486	1902	SMG5, nonse	gene	biological_pr	G:9606:SMG5			KEGG-&-1&-l	SMG5	Homo sapier Gene Symbol	SMG5	FALSE	SMG5
taxon:9606	177582	1902	SWI/SNF rel	gene	biological_pr	G:9606:SMARCA4			KEGG-&-1&-l	SMARCA4	Homo sapier Gene Symbol	SMARCA4	FALSE	SMARCA4
taxon:9606	181679	1902	eukaryotic tr	gene	biological_pr	G:9606:EEF2			KEGG-&-1&-l	EEF2	Homo sapier Gene Symbol	EEF2	FALSE	EEF2
taxon:9606	169391	1902	sortilin relat	gene	biological_pr	G:9606:SORCS2				SORCS2	Homo sapier Gene Symbol	SORCS2	FALSE	SORCS2
taxon:9606	177580	1902	SWI/SNF rel	gene	biological_pr	G:9606:SMARCC1			KEGG-&-1&-l	SMARCC1	Homo sapier Gene Symbol	SMARCC1	FALSE	SMARCC1
taxon:9606	177581	1902	SWI/SNF rel	gene	biological_pr	G:9606:SMARCB1			KEGG-&-1&-l	SMARCB1	Homo sapier Gene Symbol	SMARCB1	FALSE	SMARCB1
taxon:9606	177586	1902	snail family t	gene	biological_pr	G:9606:SNAI2			KEGG-&-1&-l	SNAI2	Homo sapier Gene Symbol	SNAI2	FALSE	SNAI2
taxon:9606	173490	1902	UFM1 specifi	gene	biological_pr	G:9606:UFL1				UFL1	Homo sapier Gene Symbol	UFL1	FALSE	UFL1
taxon:9606	181683	1902	eukaryotic tr	gene	biological_pr	G:9606:EEF1B2				EEF1B2	Homo sapier Gene Symbol	EEF1B2	FALSE	EEF1B2
taxon:9606	181680	1902	eukaryotic tr	gene	biological_pr	G:9606:EEF1G			KEGG-&-1&-l	EEF1G	Homo sapier Gene Symbol	EEF1G	FALSE	EEF1G
taxon:9606	177584	1902	SWI/SNF rel	gene	biological_pr	G:9606:SMARCA2			KEGG-&-1&-l	SMARCA2	Homo sapier Gene Symbol	SMARCA2	FALSE	SMARCA2
taxon:9606	173489	1902	ribosomal RI	gene	biological_pr	G:9606:RRP8				RRP8	Homo sapier Gene Symbol	RRP8	FALSE	RRP8
taxon:9606	177585	1902	SWI/SNF rel	gene	biological_pr	G:9606:SMARCA1				SMARCA1	Homo sapier Gene Symbol	SMARCA1	FALSE	SMARCA1
taxon:9606	181681	1902	eukaryotic tr	gene	biological_pr	G:9606:EEF1D			KEGG-&-1&-l	EEF1D	Homo sapier Gene Symbol	EEF1D	FALSE	EEF1D
taxon:9606	173495	1902	protein phos	gene	biological_pr	G:9606:PPP1R13B				PPP1R13B	Homo sapier Gene Symbol	PPP1R13B	FALSE	PPP1R13B
taxon:9606	181687	1902	eukaryotic tr	gene	biological_pr	G:9606:EEF1A1			KEGG-&-1&-l	EEF1A1	Homo sapier Gene Symbol	EEF1A1	FALSE	EEF1A1
taxon:9606	173499	1902	obscurin like	gene	biological_pr	G:9606:OBSL1				OBSL1	Homo sapier Gene Symbol	OBSL1	FALSE	OBSL1
taxon:9606	169406	1902	HECT, C2 and	gene	biological_pr	G:9606:HECW2				HECW2	Homo sapier Gene Symbol	HECW2	FALSE	HECW2
taxon:9606	181692	1902	endothelin re	gene	biological_pr	G:9606:EDNRB			KEGG-&-1&-l	EDNRB	Homo sapier Gene Symbol	EDNRB	FALSE	EDNRB
taxon:9606	169405	1902	regulatory as	gene	biological_pr	G:9606:RPTOR			KEGG-&-1&-l	RPTOR	Homo sapier Gene Symbol	RPTOR	FALSE	RPTOR
taxon:9606	165314	1902	histone clust	gene	biological_pr	G:9606:HIST4H4			KEGG-&-1&-l	HIST4H4	Homo sapier Gene Symbol	HIST4H4	FALSE	HIST4H4
taxon:9606	173504	1902	ubiquitin spe	gene	biological_pr	G:9606:USP24				USP24	Homo sapier Gene Symbol	USP24	FALSE	USP24
taxon:9606	181701	1902	epithelial cel	gene	biological_pr	G:9606:ECT2				ECT2	Homo sapier Gene Symbol	ECT2	FALSE	ECT2
taxon:9606	173509	1902	ubiquitin pro	gene	biological_pr	G:9606:UBR4			KEGG-&-1&-l	UBR4	Homo sapier Gene Symbol	UBR4	FALSE	UBR4
taxon:9606	169413	1902	exportin 5	gene	biological_pr	G:9606:XPO5			KEGG-&-1&-l	XPO5	Homo sapier Gene Symbol	XPO5	FALSE	XPO5
taxon:9606	169419	1902	metastasis a	gene	biological_pr	G:9606:MTA3				MTA3	Homo sapier Gene Symbol	MTA3	FALSE	MTA3
taxon:9606	173516	1902	extended syn	gene	biological_pr	G:9606:ESYT1				ESYT1	Homo sapier Gene Symbol	ESYT1	FALSE	ESYT1
taxon:9606	181714	1902	E4F transcrip	gene	biological_pr	G:9606:E4F1				E4F1	Homo sapier Gene Symbol	E4F1	FALSE	E4F1
taxon:9606	181717	1902	E2F transcrip	gene	biological_pr	G:9606:E2F4			KEGG-&-1&-l	E2F4	Homo sapier Gene Symbol	E2F4	FALSE	E2F4
taxon:9606	173528	1902	neural precu	gene	biological_pr	G:9606:NEDD4L			KEGG-&-1&-l	NEDD4L	Homo sapier Gene Symbol	NEDD4L	FALSE	NEDD4L
taxon:9606	181721	1902	E2F transcrip	gene	biological_pr	G:9606:E2F1			KEGG-&-1&-l	E2F1	Homo sapier Gene Symbol	E2F1	FALSE	E2F1
taxon:9606	173529	1902	ubiquitin spe	gene	biological_pr	G:9606:USP22				USP22	Homo sapier Gene Symbol	USP22	FALSE	USP22
taxon:9606	181726	1902	dual specifici	gene	biological_pr	G:9606:DYRK1A				DYRK1A	Homo sapier Gene Symbol	DYRK1A	FALSE	DYRK1A
taxon:9606	173532	1902	RPGRIP1 like	gene	biological_pr	G:9606:RPGRIP1L				RPGRIP1L	Homo sapier Gene Symbol	RPGRIP1L	FALSE	RPGRIP1L
taxon:9606	181730	1902	dishevelled s	gene	biological_pr	G:9606:DVL1			KEGG-&-1&-l	DVL1	Homo sapier Gene Symbol	DVL1	FALSE	DVL1
taxon:9606	177635	1902	solute carrier	gene	biological_pr	G:9606:SLC6A8				SLC6A8	Homo sapier Gene Symbol	SLC6A8	FALSE	SLC6A8
taxon:9606	181728	1902	dishevelled s	gene	biological_pr	G:9606:DVL3			KEGG-&-1&-l	DVL3	Homo sapier Gene Symbol	DVL3	FALSE	DVL3
taxon:9606	181729	1902	dishevelled s	gene	biological_pr	G:9606:DVL2			KEGG-&-1&-l	DVL2	Homo sapier Gene Symbol	DVL2	FALSE	DVL2
taxon:9606	173542	1902	SIN3 transcri	gene	biological_pr	G:9606:SIN3B				SIN3B	Homo sapier Gene Symbol	SIN3B	FALSE	SIN3B
taxon:9606	181751	1902	TSC22 domai	gene	biological_pr	G:9606:TSC22D3				TSC22D3	Homo sapier Gene Symbol	TSC22D3	FALSE	TSC22D3
taxon:9606	173556	1902	F-box and W	gene	biological_pr	G:9606:FBXW11			KEGG-&-1&-l	FBXW11	Homo sapier Gene Symbol	FBXW11	FALSE	FBXW11
taxon:9606	177656	1902	solute carrier	gene	biological_pr	G:9606:SLC2A1			KEGG-&-1&-l	SLC2A1	Homo sapier Gene Symbol	SLC2A1	FALSE	SLC2A1
taxon:9606	169465	1902	baculoviral IA	gene	biological_pr	G:9606:BIRC6			KEGG-&-1&-l	BIRC6	Homo sapier Gene Symbol	BIRC6	FALSE	BIRC6
taxon:9606	177667	1902	S-phase kina	gene	biological_pr	G:9606:SKP2			KEGG-&-1&-l	SKP2	Homo sapier Gene Symbol	SKP2	FALSE	SKP2
taxon:9606	177671	1902	SKI proto-on	gene	biological_pr	G:9606:SKI				SKI	Homo sapier Gene Symbol	SKI	FALSE	SKI
taxon:9606	177668	1902	S-phase kina	gene	biological_pr	G:9606:SKP1			KEGG-&-1&-l	SKP1	Homo sapier Gene Symbol	SKP1	FALSE	SKP1
taxon:9606	181770	1902	CD4 molecul	gene	biological_pr	G:9606:CD4			KEGG-&-1&-l	CD4	Homo sapier Gene Symbol	CD4	FALSE	CD4
taxon:9606	144907	1902	RBM14-RBM	gene		G:9606:RBM14-RBM4				RBM14-RBM	Homo sapier Gene Symbol	RBM14-RBM	FALSE	RBM14-RBM4
taxon:9606	181774	1902	CD3d molecu	gene	biological_pr	G:9606:CD3D			KEGG-&-1&-l	CD3D	Homo sapier Gene Symbol	CD3D	FALSE	CD3D
taxon:9606	181772	1902	CD3g molecu	gene	biological_pr	G:9606:CD3G			KEGG-&-1&-l	CD3G	Homo sapier Gene Symbol	CD3G	FALSE	CD3G
taxon:9606	173581	1902	DENN domai	gene	biological_pr	G:9606:DENND5A				DENND5A	Homo sapier Gene Symbol	DENND5A	FALSE	DENND5A
taxon:9606	169491	1902	zinc finger CC	gene	biological_pr	G:9606:ZC3HAV1				ZC3HAV1	Homo sapier Gene Symbol	ZC3HAV1	FALSE	ZC3HAV1
taxon:9606	177687	1902	serine and ar	gene	biological_pr	G:9606:SRSF6			KEGG-&-1&-l	SRSF6	Homo sapier Gene Symbol	SRSF6	FALSE	SRSF6
taxon:9606	181781	1902	chaperonin c	gene	biological_pr	G:9606:CCT6A				CCT6A	Homo sapier Gene Symbol	CCT6A	FALSE	CCT6A
taxon:9606	181787	1902	cyclin G1	gene	biological_pr	G:9606:CCNG1			KEGG-&-1&-l	CCNG1	Homo sapier Gene Symbol	CCNG1	FALSE	CCNG1
taxon:9606	177688	1902	serine and ar	gene	biological_pr	G:9606:SRSF5			KEGG-&-1&-l	SRSF5	Homo sapier Gene Symbol	SRSF5	FALSE	SRSF5
taxon:9606	181785	1902	cyclin H	gene	biological_pr	G:9606:CCNH			KEGG-&-1&-l	CCNH	Homo sapier Gene Symbol	CCNH	FALSE	CCNH
taxon:9606	173598	1902	family with s	gene	biological_pr	G:9606:FAM175B				FAM175B	Homo sapier Gene Symbol	FAM175B	FALSE	FAM175B
taxon:9606	177694	1902	secreted friz	gene	biological_pr	G:9606:SFRP4			KEGG-&-1&-l	SFRP4	Homo sapier Gene Symbol	SFRP4	FALSE	SFRP4
taxon:9606	177692	1902	serine and ar	gene	biological_pr	G:9606:SRSF1			KEGG-&-1&-l	SRSF1	Homo sapier Gene Symbol	SRSF1	FALSE	SRSF1
taxon:9606	181789	1902	cyclin E1	gene	biological_pr	G:9606:CCNE1			KEGG-&-1&-l	CCNE1	Homo sapier Gene Symbol	CCNE1	FALSE	CCNE1
taxon:9606	181794	1902	cyclin C	gene	biological_pr	G:9606:CCNC				CCNC	Homo sapier Gene Symbol	CCNC	FALSE	CCNC
taxon:9606	181795	1902	cyclin B1	gene	biological_pr	G:9606:CCNB1			KEGG-&-1&-l	CCNB1	Homo sapier Gene Symbol	CCNB1	FALSE	CCNB1
taxon:9606	169507	1902	A-kinase inte	gene	biological_pr	G:9606:AKIP1				AKIP1	Homo sapier Gene Symbol	AKIP1	FALSE	AKIP1
taxon:9606	169504	1902	nuclear recep	gene	biological_pr	G:9606:NRIP3				NRIP3	Homo sapier Gene Symbol	NRIP3	FALSE	NRIP3
taxon:9606	177697	1902	splicing facto	gene	biological_pr	G:9606:SFPQ				SFPQ	Homo sapier Gene Symbol	SFPQ	FALSE	SFPQ
taxon:9606	173606	1902	myosin phos	gene	cellular_com	G:9606:MPRIP				MPRIP	Homo sapier Gene Symbol	MPRIP	FALSE	MPRIP
taxon:9606	181796	1902	cyclin A2	gene	biological_pr	G:9606:CCNA2			KEGG-&-1&-l	CCNA2	Homo sapier Gene Symbol	CCNA2	FALSE	CCNA2
taxon:9606	177700	1902	SET nuclear p	gene	biological_pr	G:9606:SET				SET	Homo sapier Gene Symbol	SET	FALSE	SET
taxon:9606	169509	1902	mucin 13, cel	gene	biological_pr	G:9606:MUC13				MUC13	Homo sapier Gene Symbol	MUC13	FALSE	MUC13
taxon:9606	173605	1902	nucleoporin 2	gene	biological_pr	G:9606:NUP205			KEGG-&-1&-l	NUP205	Homo sapier Gene Symbol	NUP205	FALSE	NUP205
taxon:9606	177711	1902	semaphorin 3	gene	biological_pr	G:9606:SEMA3F			KEGG-&-1&-l	SEMA3F	Homo sapier Gene Symbol	SEMA3F	FALSE	SEMA3F
taxon:9606	169516	1902	tripartite mo	gene	biological_pr	G:9606:TRIM39				TRIM39	Homo sapier Gene Symbol	TRIM39	FALSE	TRIM39
taxon:9606	173612	1902	septin 6	gene	biological_pr	G:9606:SEPT6			KEGG-&-1&-l	Sep-06	Homo sapier Gene Symbol	Sep-06	FALSE	Sep-06
taxon:9606	173613	1902	chloride char	gene	biological_pr	G:9606:CLCC1				CLCC1	Homo sapier Gene Symbol	CLCC1	FALSE	CLCC1
taxon:9606	181811	1902	Cbl proto-onc	gene	biological_pr	G:9606:CBL			KEGG-&-1&-l	CBL	Homo sapier Gene Symbol	CBL	FALSE	CBL
taxon:9606	181808	1902	serpin family	gene	biological_pr	G:9606:SERPINH1				SERPINH1	Homo sapier Gene Symbol	SERPINH1	FALSE	SERPINH1
taxon:9606	169525	1902	BRCA2 and C	gene	biological_pr	G:9606:BCCIP				BCCIP	Homo sapier Gene Symbol	BCCIP	FALSE	BCCIP
taxon:9606	181818	1902	runt related t	gene	biological_pr	G:9606:RUNX2			KEGG-&-1&-l	RUNX2	Homo sapier Gene Symbol	RUNX2	FALSE	RUNX2
taxon:9606	181817	1902	runt related t	gene	biological_pr	G:9606:RUNX1			KEGG-&-1&-l	RUNX1	Homo sapier Gene Symbol	RUNX1	FALSE	RUNX1
taxon:9606	165433	1902	ring finger pr	gene	biological_pr	G:9606:RNF166				RNF166	Homo sapier Gene Symbol	RNF166	FALSE	RNF166
taxon:9606	169534	1902	matrix metal	gene	biological_pr	G:9606:MMP26				MMP26	Homo sapier Gene Symbol	MMP26	FALSE	MMP26
taxon:9606	177727	1902	syntaxin 1A	gene	biological_pr	G:9606:STX1A			KEGG-&-1&-l	STX1A	Homo sapier Gene Symbol	STX1A	FALSE	STX1A
taxon:9606	177724	1902	syntaxin 5	gene	biological_pr	G:9606:STX5			KEGG-&-1&-l	STX5	Homo sapier Gene Symbol	STX5	FALSE	STX5
taxon:9606	181821	1902	caveolin 1	gene	biological_pr	G:9606:CAV1			KEGG-&-1&-l	CAV1	Homo sapier Gene Symbol	CAV1	FALSE	CAV1
taxon:9606	173635	1902	erythrocyte n	gene	biological_pr	G:9606:EPB41L3				EPB41L3	Homo sapier Gene Symbol	EPB41L3	FALSE	EPB41L3
taxon:9606	173638	1902	RAD54-like 2	gene	cellular_com	G:9606:RAD54L2				RAD54L2	Homo sapier Gene Symbol	RAD54L2	FALSE	RAD54L2
taxon:9606	181830	1902	caspase 7	gene	biological_pr	G:9606:CASP7			KEGG-&-1&-l	CASP7	Homo sapier Gene Symbol	CASP7	FALSE	CASP7
taxon:9606	173639	1902	G-patch dom	gene	biological_pr	G:9606:GPATCH8				GPATCH8	Homo sapier Gene Symbol	GPATCH8	FALSE	GPATCH8
taxon:9606	177732	1902	serine/threo	gene	biological_pr	G:9606:STK11			KEGG-&-1&-l	STK11	Homo sapier Gene Symbol	STK11	FALSE	STK11
taxon:9606	161348	1902	histone clust	gene	biological_pr	G:9606:HIST2H3A			KEGG-&-1&-l	HIST2H3A	Homo sapier Gene Symbol	HIST2H3A	FALSE	HIST2H3A
taxon:9606	181829	1902	caspase 8	gene	biological_pr	G:9606:CASP8			KEGG-&-1&-l	CASP8	Homo sapier Gene Symbol	CASP8	FALSE	CASP8
taxon:9606	181834	1902	caspase 3	gene	biological_pr	G:9606:CASP3			KEGG-&-1&-l	CASP3	Homo sapier Gene Symbol	CASP3	FALSE	CASP3
taxon:9606	177736	1902	aurora kinas	gene	biological_pr	G:9606:AURKA			KEGG-&-1&-l	AURKA	Homo sapier Gene Symbol	AURKA	FALSE	AURKA
taxon:9606	177737	1902	serine/threo	gene	biological_pr	G:9606:STK4			KEGG-&-1&-l	STK4	Homo sapier Gene Symbol	STK4	FALSE	STK4
taxon:9606	181838	1902	capping actir	gene	biological_pr	G:9606:CAPZB			KEGG-&-1&-l	CAPZB	Homo sapier Gene Symbol	CAPZB	FALSE	CAPZB
taxon:9606	177742	1902	sulfotransfer	gene	biological_pr	G:9606:SULT1E1			KEGG-&-1&-l	SULT1E1	Homo sapier Gene Symbol	SULT1E1	FALSE	SULT1E1
taxon:9606	169551	1902	par-3 family	gene	biological_pr	G:9606:PARD3			KEGG-&-1&-l	PARD3	Homo sapier Gene Symbol	PARD3	FALSE	PARD3
taxon:9606	177743	1902	heat shock p	gene	cellular_com	G:9606:HSPA13				HSPA13	Homo sapier Gene Symbol	HSPA13	FALSE	HSPA13
taxon:9606	181836	1902	caspase 1	gene	biological_pr	G:9606:CASP1			KEGG-&-1&-l	CASP1	Homo sapier Gene Symbol	CASP1	FALSE	CASP1
taxon:9606	181837	1902	cysteinyl-tRN	gene	biological_pr	G:9606:CARS			KEGG-&-1&-l	CARS	Homo sapier Gene Symbol	CARS	FALSE	CARS
taxon:9606	177747	1902	signal transd	gene	biological_pr	G:9606:STAT6			KEGG-&-1&-l	STAT6	Homo sapier Gene Symbol	STAT6	FALSE	STAT6
taxon:9606	173648	1902	TGF-beta act	gene	biological_pr	G:9606:TAB2			KEGG-&-1&-l	TAB2	Homo sapier Gene Symbol	TAB2	FALSE	TAB2
taxon:9606	177745	1902	staufen doub	gene	biological_pr	G:9606:STAU1				STAU1	Homo sapier Gene Symbol	STAU1	FALSE	STAU1
taxon:9606	181841	1902	capping actir	gene	biological_pr	G:9606:CAPZA1			KEGG-&-1&-l	CAPZA1	Homo sapier Gene Symbol	CAPZA1	FALSE	CAPZA1
taxon:9606	165462	1902	musashi RNA	gene	biological_pr	G:9606:MSI2			KEGG-&-1&-l	MSI2	Homo sapier Gene Symbol	MSI2	FALSE	MSI2
taxon:9606	181847	1902	calpain 1	gene	biological_pr	G:9606:CAPN1			KEGG-&-1&-l	CAPN1	Homo sapier Gene Symbol	CAPN1	FALSE	CAPN1
taxon:9606	177751	1902	signal transd	gene	biological_pr	G:9606:STAT3			KEGG-&-1&-l	STAT3	Homo sapier Gene Symbol	STAT3	FALSE	STAT3
taxon:9606	173652	1902	cullin 9	gene	biological_pr	G:9606:CUL9				CUL9	Homo sapier Gene Symbol	CUL9	FALSE	CUL9
taxon:9606	181844	1902	calpain small	gene	biological_pr	G:9606:CAPNS1				CAPNS1	Homo sapier Gene Symbol	CAPNS1	FALSE	CAPNS1
taxon:9606	177748	1902	signal transd	gene	biological_pr	G:9606:STAT5B			KEGG-&-1&-l	STAT5B	Homo sapier Gene Symbol	STAT5B	FALSE	STAT5B
taxon:9606	177749	1902	signal transd	gene	biological_pr	G:9606:STAT5A			KEGG-&-1&-l	STAT5A	Homo sapier Gene Symbol	STAT5A	FALSE	STAT5A
taxon:9606	181845	1902	calpain 3	gene	biological_pr	G:9606:CAPN3				CAPN3	Homo sapier Gene Symbol	CAPN3	FALSE	CAPN3
taxon:9606	169563	1902	methylphosp	gene	biological_pr	G:9606:MEPCE				MEPCE	Homo sapier Gene Symbol	MEPCE	FALSE	MEPCE
taxon:9606	181848	1902	capping actir	gene	biological_pr	G:9606:CAPG				CAPG	Homo sapier Gene Symbol	CAPG	FALSE	CAPG
taxon:9606	173657	1902	mitochondria	gene	biological_pr	G:9606:MRPS27				MRPS27	Homo sapier Gene Symbol	MRPS27	FALSE	MRPS27
taxon:9606	181849	1902	calnexin	gene	biological_pr	G:9606:CANX			KEGG-&-1&-l	CANX	Homo sapier Gene Symbol	CANX	FALSE	CANX
taxon:9606	169566	1902	ring finger pr	gene	biological_pr	G:9606:RNF20				RNF20	Homo sapier Gene Symbol	RNF20	FALSE	RNF20
taxon:9606	177758	1902	suppression	gene	biological_pr	G:9606:ST13				ST13	Homo sapier Gene Symbol	ST13	FALSE	ST13
taxon:9606	181855	1902	calcium/calm	gene	biological_pr	G:9606:CAMK2A			KEGG-&-1&-l	CAMK2A	Homo sapier Gene Symbol	CAMK2A	FALSE	CAMK2A
taxon:9606	173660	1902	MCF.2 cell lir	gene	biological_pr	G:9606:MCF2L2				MCF2L2	Homo sapier Gene Symbol	MCF2L2	FALSE	MCF2L2
taxon:9606	181858	1902	calreticulin	gene	biological_pr	G:9606:CALR			KEGG-&-1&-l	CALR	Homo sapier Gene Symbol	CALR	FALSE	CALR
taxon:9606	173667	1902	tubulin tyrosi	gene	biological_pr	G:9606:TTLL5				TTLL5	Homo sapier Gene Symbol	TTLL5	FALSE	TTLL5
taxon:9606	181857	1902	calumenin	gene	biological_pr	G:9606:CALU				CALU	Homo sapier Gene Symbol	CALU	FALSE	CALU
taxon:9606	173670	1902	zinc finger pr	gene	biological_pr	G:9606:ZNF423				ZNF423	Homo sapier Gene Symbol	ZNF423	FALSE	ZNF423
taxon:9606	165477	1902	nitric oxide s	gene	biological_pr	G:9606:NOSTRIN				NOSTRIN	Homo sapier Gene Symbol	NOSTRIN	FALSE	NOSTRIN
taxon:9606	177770	1902	somatostatin	gene	biological_pr	G:9606:SSTR3			KEGG-&-1&-l	SSTR3	Homo sapier Gene Symbol	SSTR3	FALSE	SSTR3
taxon:9606	181867	1902	caldesmon 1	gene	biological_pr	G:9606:CALD1			KEGG-&-1&-l	CALD1	Homo sapier Gene Symbol	CALD1	FALSE	CALD1
taxon:9606	165486	1902	ubiquitin like	gene	biological_pr	G:9606:UHRF2				UHRF2	Homo sapier Gene Symbol	UHRF2	FALSE	UHRF2
taxon:9606	177774	1902	structure spe	gene	biological_pr	G:9606:SSRP1				SSRP1	Homo sapier Gene Symbol	SSRP1	FALSE	SSRP1
taxon:9606	181875	1902	carbamoyl-ph	gene	biological_pr	G:9606:CAD			KEGG-&-1&-l	CAD	Homo sapier Gene Symbol	CAD	FALSE	CAD
taxon:9606	173683	1902	HECT, C2 and	gene	biological_pr	G:9606:HECW1				HECW1	Homo sapier Gene Symbol	HECW1	FALSE	HECW1
taxon:9606	173680	1902	ribosomal RI	gene	biological_pr	G:9606:RRP1B				RRP1B	Homo sapier Gene Symbol	RRP1B	FALSE	RRP1B
taxon:9606	173681	1902	SWAP switch	gene	biological_pr	G:9606:SWAP70				SWAP70	Homo sapier Gene Symbol	SWAP70	FALSE	SWAP70
taxon:9606	177781	1902	Sjogren synd	gene	biological_pr	G:9606:SSB			KEGG-&-1&-l	SSB	Homo sapier Gene Symbol	SSB	FALSE	SSB
taxon:9606	177785	1902	sex determin	gene	biological_pr	G:9606:SRY				SRY	Homo sapier Gene Symbol	SRY	FALSE	SRY
taxon:9606	169598	1902	protocadheri	gene	biological_pr	G:9606:PCDHA4				PCDHA4	Homo sapier Gene Symbol	PCDHA4	FALSE	PCDHA4
taxon:9606	177788	1902	SRSF protein	gene	biological_pr	G:9606:SRPK1			KEGG-&-1&-l	SRPK1	Homo sapier Gene Symbol	SRPK1	FALSE	SRPK1
taxon:9606	173696	1902	nuclear recep	gene	biological_pr	G:9606:NCOA6				NCOA6	Homo sapier Gene Symbol	NCOA6	FALSE	NCOA6
taxon:9606	181889	1902	calcium voltag	gene	biological_pr	G:9606:CACNA1C			KEGG-&-1&-l	CACNA1C	Homo sapier Gene Symbol	CACNA1C	FALSE	CACNA1C
taxon:9606	169614	1902	protocadheri	gene	biological_pr	G:9606:PCDHB8				PCDHB8	Homo sapier Gene Symbol	PCDHB8	FALSE	PCDHB8
taxon:9606	177808	1902	C-X-C motif c	gene	biological_pr	G:9606:CXCL12			KEGG-&-1&-l	CXCL12	Homo sapier Gene Symbol	CXCL12	FALSE	CXCL12
taxon:9606	177809	1902	syndecan bin	gene	biological_pr	G:9606:SDCBP				SDCBP	Homo sapier Gene Symbol	SDCBP	FALSE	SDCBP
taxon:9606	181910	1902	formin like 1	gene	biological_pr	G:9606:FMNL1				FMNL1	Homo sapier Gene Symbol	FMNL1	FALSE	FMNL1
taxon:9606	173719	1902	lysine demet	gene	biological_pr	G:9606:KDM1A			KEGG-&-1&-l	KDM1A	Homo sapier Gene Symbol	KDM1A	FALSE	KDM1A
taxon:9606	181908	1902	pituitary tum	gene	biological_pr	G:9606:PTTG1IP				PTTG1IP	Homo sapier Gene Symbol	PTTG1IP	FALSE	PTTG1IP
taxon:9606	173716	1902	microtubule a	gene	biological_pr	G:9606:MAST3				MAST3	Homo sapier Gene Symbol	MAST3	FALSE	MAST3
taxon:9606	177819	1902	C-X-C motif c	gene	biological_pr	G:9606:CXCL6			KEGG-&-1&-l	CXCL6	Homo sapier Gene Symbol	CXCL6	FALSE	CXCL6
taxon:9606	177817	1902	C-X-C motif c	gene	biological_pr	G:9606:CXCL5			KEGG-&-1&-l	CXCL5	Homo sapier Gene Symbol	CXCL5	FALSE	CXCL5
taxon:9606	177823	1902	C-C motif ch	gene	biological_pr	G:9606:CCL22			KEGG-&-1&-l	CCL22	Homo sapier Gene Symbol	CCL22	FALSE	CCL22
taxon:9606	165533	1902	oxysterol bin	gene	biological_pr	G:9606:OSBPL10				OSBPL10	Homo sapier Gene Symbol	OSBPL10	FALSE	OSBPL10
taxon:9606	173725	1902	small nuclea	gene	biological_pr	G:9606:SNRNP200			KEGG-&-1&-l	SNRNP200	Homo sapier Gene Symbol	SNRNP200	FALSE	SNRNP200
taxon:9606	173730	1902	F-box protein	gene	biological_pr	G:9606:FBXO21				FBXO21	Homo sapier Gene Symbol	FBXO21	FALSE	FBXO21
taxon:9606	173728	1902	exosome com	gene	biological_pr	G:9606:EXOSC7			KEGG-&-1&-l	EXOSC7	Homo sapier Gene Symbol	EXOSC7	FALSE	EXOSC7
taxon:9606	177828	1902	C-C motif ch	gene	biological_pr	G:9606:CCL18			KEGG-&-1&-l	CCL18	Homo sapier Gene Symbol	CCL18	FALSE	CCL18
taxon:9606	177829	1902	C-C motif ch	gene	biological_pr	G:9606:CCL17			KEGG-&-1&-l	CCL17	Homo sapier Gene Symbol	CCL17	FALSE	CCL17
taxon:9606	177835	1902	C-C motif ch	gene	biological_pr	G:9606:CCL8			KEGG-&-1&-l	CCL8	Homo sapier Gene Symbol	CCL8	FALSE	CCL8
taxon:9606	177833	1902	C-C motif ch	gene	biological_pr	G:9606:CCL13			KEGG-&-1&-l	CCL13	Homo sapier Gene Symbol	CCL13	FALSE	CCL13
taxon:9606	173743	1902	centrosomal	gene	biological_pr	G:9606:CEP152				CEP152	Homo sapier Gene Symbol	CEP152	FALSE	CEP152
taxon:9606	177836	1902	C-C motif ch	gene	biological_pr	G:9606:CCL7			KEGG-&-1&-l	CCL7	Homo sapier Gene Symbol	CCL7	FALSE	CCL7
taxon:9606	177841	1902	C-C motif ch	gene	biological_pr	G:9606:CCL2			KEGG-&-1&-l	CCL2	Homo sapier Gene Symbol	CCL2	FALSE	CCL2
taxon:9606	181946	1902	BCL2, apopto	gene	biological_pr	G:9606:BCL2			KEGG-&-1&-l	BCL2	Homo sapier Gene Symbol	BCL2	FALSE	BCL2
taxon:9606	181947	1902	cyclin D1	gene	biological_pr	G:9606:CCND1			KEGG-&-1&-l	CCND1	Homo sapier Gene Symbol	CCND1	FALSE	CCND1
taxon:9606	173755	1902	ninein like	gene	biological_pr	G:9606:NINL				NINL	Homo sapier Gene Symbol	NINL	FALSE	NINL
taxon:9606	181944	1902	BCL2 like 1	gene	biological_pr	G:9606:BCL2L1			KEGG-&-1&-l	BCL2L1	Homo sapier Gene Symbol	BCL2L1	FALSE	BCL2L1
taxon:9606	173753	1902	microtubule a	gene	biological_pr	G:9606:MAST1				MAST1	Homo sapier Gene Symbol	MAST1	FALSE	MAST1
taxon:9606	169663	1902	BAI1 associa	gene	biological_pr	G:9606:BAIAP2L1				BAIAP2L1	Homo sapier Gene Symbol	BAIAP2L1	FALSE	BAIAP2L1
taxon:9606	173760	1902	PAX interacti	gene	biological_pr	G:9606:PAXIP1				PAXIP1	Homo sapier Gene Symbol	PAXIP1	FALSE	PAXIP1
taxon:9606	173767	1902	tripartite mo	gene	biological_pr	G:9606:TRIM32			KEGG-&-1&-l	TRIM32	Homo sapier Gene Symbol	TRIM32	FALSE	TRIM32
taxon:9606	181959	1902	BCL2 antago	gene	biological_pr	G:9606:BAK1			KEGG-&-1&-l	BAK1	Homo sapier Gene Symbol	BAK1	FALSE	BAK1
taxon:9606	161479	1902	histone clust	gene	biological_pr	G:9606:HIST2H2AB			KEGG-&-1&-l	HIST2H2AB	Homo sapier Gene Symbol	HIST2H2AB	FALSE	HIST2H2AB
taxon:9606	181956	1902	BCL2 associa	gene	biological_pr	G:9606:BAX			KEGG-&-1&-l	BAX	Homo sapier Gene Symbol	BAX	FALSE	BAX
taxon:9606	181957	1902	BRCA1 assoc	gene	biological_pr	G:9606:BARD1			KEGG-&-1&-l	BARD1	Homo sapier Gene Symbol	BARD1	FALSE	BARD1
taxon:9606	169669	1902	PDZ binding k	gene	biological_pr	G:9606:PBK				PBK	Homo sapier Gene Symbol	PBK	FALSE	PBK
taxon:9606	177867	1902	serpin family	gene	biological_pr	G:9606:SERPINB4			KEGG-&-1&-l	SERPINB4	Homo sapier Gene Symbol	SERPINB4	FALSE	SERPINB4
taxon:9606	169672	1902	histone deac	gene	biological_pr	G:9606:HDAC8			KEGG-&-1&-l	HDAC8	Homo sapier Gene Symbol	HDAC8	FALSE	HDAC8
taxon:9606	181966	1902	BTB domain	gene	biological_pr	G:9606:BACH1				BACH1	Homo sapier Gene Symbol	BACH1	FALSE	BACH1
taxon:9606	173772	1902	chaperonin c	gene	biological_pr	G:9606:CCT5				CCT5	Homo sapier Gene Symbol	CCT5	FALSE	CCT5
taxon:9606	181964	1902	BCL2 associa	gene	biological_pr	G:9606:BAG1			KEGG-&-1&-l	BAG1	Homo sapier Gene Symbol	BAG1	FALSE	BAG1
taxon:9606	173778	1902	SNW domain	gene	biological_pr	G:9606:SNW1			KEGG-&-1&-l	SNW1	Homo sapier Gene Symbol	SNW1	FALSE	SNW1
taxon:9606	161489	1902	dehydrogena	gene	biological_pr	G:9606:DHRS4L2			KEGG-&-1&-l	DHRS4L2	Homo sapier Gene Symbol	DHRS4L2	FALSE	DHRS4L2
taxon:9606	177878	1902	spermidine/s	gene	biological_pr	G:9606:SAT1			KEGG-&-1&-l	SAT1	Homo sapier Gene Symbol	SAT1	FALSE	SAT1
taxon:9606	173782	1902	sirtuin 2	gene	biological_pr	G:9606:SIRT2				SIRT2	Homo sapier Gene Symbol	SIRT2	FALSE	SIRT2
taxon:9606	177877	1902	SATB homeo	gene	biological_pr	G:9606:SATB1				SATB1	Homo sapier Gene Symbol	SATB1	FALSE	SATB1
taxon:9606	173784	1902	RAB18, mem	gene	biological_pr	G:9606:RAB18				RAB18	Homo sapier Gene Symbol	RAB18	FALSE	RAB18
taxon:9606	181977	1902	CYLD lysine 6	gene	biological_pr	G:9606:CYLD			KEGG-&-1&-l	CYLD	Homo sapier Gene Symbol	CYLD	FALSE	CYLD
taxon:9606	177887	1902	scaffold atta	gene	biological_pr	G:9606:SAFB				SAFB	Homo sapier Gene Symbol	SAFB	FALSE	SAFB
taxon:9606	173788	1902	hyaluronan b	gene	biological_pr	G:9606:HABP4				HABP4	Homo sapier Gene Symbol	HABP4	FALSE	HABP4
taxon:9606	173794	1902	microtubule a	gene	biological_pr	G:9606:MAPRE1				MAPRE1	Homo sapier Gene Symbol	MAPRE1	FALSE	MAPRE1
taxon:9606	173793	1902	kinesin assoc	gene	biological_pr	G:9606:KIFAP3				KIFAP3	Homo sapier Gene Symbol	KIFAP3	FALSE	KIFAP3
taxon:9606	177895	1902	S100 calcium	gene	biological_pr	G:9606:S100B				S100B	Homo sapier Gene Symbol	S100B	FALSE	S100B
taxon:9606	169701	1902	ubiquitin spe	gene	biological_pr	G:9606:USP29				USP29	Homo sapier Gene Symbol	USP29	FALSE	USP29
taxon:9606	161512	1902	glutamate ri	gene	molecular_fu	G:9606:ERICH2				ERICH2	Homo sapier Gene Symbol	ERICH2	FALSE	ERICH2
taxon:9606	169704	1902	prostate tran	gene	biological_pr	G:9606:PMEPA1				PMEPA1	Homo sapier Gene Symbol	PMEPA1	FALSE	PMEPA1
taxon:9606	173800	1902	RALY hetero	gene	biological_pr	G:9606:RALY				RALY	Homo sapier Gene Symbol	RALY	FALSE	RALY
taxon:9606	177902	1902	S100 calcium	gene	biological_pr	G:9606:S100A7			KEGG-&-1&-l	S100A7	Homo sapier Gene Symbol	S100A7	FALSE	S100A7
taxon:9606	177903	1902	S100 calcium	gene	biological_pr	G:9606:S100A6				S100A6	Homo sapier Gene Symbol	S100A6	FALSE	S100A6
taxon:9606	177900	1902	S100 calcium	gene	biological_pr	G:9606:S100A9			KEGG-&-1&-l	S100A9	Homo sapier Gene Symbol	S100A9	FALSE	S100A9
taxon:9606	177901	1902	S100 calcium	gene	biological_pr	G:9606:S100A8			KEGG-&-1&-l	S100A8	Homo sapier Gene Symbol	S100A8	FALSE	S100A8
taxon:9606	169714	1902	p21 (RAC1) a	gene	biological_pr	G:9606:PAK6			KEGG-&-1&-l	PAK6	Homo sapier Gene Symbol	PAK6	FALSE	PAK6
taxon:9606	182002	1902	cathepsin G	gene	biological_pr	G:9606:CTSG			KEGG-&-1&-l	CTSG	Homo sapier Gene Symbol	CTSG	FALSE	CTSG
taxon:9606	177907	1902	S100 calcium	gene	biological_pr	G:9606:S100A2				S100A2	Homo sapier Gene Symbol	S100A2	FALSE	S100A2
taxon:9606	169712	1902	nicalin	gene	biological_pr	G:9606:NCLN				NCLN	Homo sapier Gene Symbol	NCLN	FALSE	NCLN
taxon:9606	177905	1902	S100 calcium	gene	biological_pr	G:9606:S100A4				S100A4	Homo sapier Gene Symbol	S100A4	FALSE	S100A4
taxon:9606	177908	1902	sortilin 1	gene	biological_pr	G:9606:SORT1			KEGG-&-1&-l	SORT1	Homo sapier Gene Symbol	SORT1	FALSE	SORT1
taxon:9606	177909	1902	S100 calcium	gene	biological_pr	G:9606:S100A1				S100A1	Homo sapier Gene Symbol	S100A1	FALSE	S100A1
taxon:9606	182005	1902	cathepsin B	gene	biological_pr	G:9606:CTSB			KEGG-&-1&-l	CTSB	Homo sapier Gene Symbol	CTSB	FALSE	CTSB
taxon:9606	177914	1902	ryanodine re	gene	biological_pr	G:9606:RYR2			KEGG-&-1&-l	RYR2	Homo sapier Gene Symbol	RYR2	FALSE	RYR2
taxon:9606	182011	1902	catenin beta	gene	biological_pr	G:9606:CTNNB1			KEGG-&-1&-l	CTNNB1	Homo sapier Gene Symbol	CTNNB1	FALSE	CTNNB1
taxon:9606	169720	1902	cullin associa	gene	biological_pr	G:9606:CAND1				CAND1	Homo sapier Gene Symbol	CAND1	FALSE	CAND1
taxon:9606	177913	1902	ryanodine re	gene	biological_pr	G:9606:RYR3			KEGG-&-1&-l	RYR3	Homo sapier Gene Symbol	RYR3	FALSE	RYR3
taxon:9606	173817	1902	ataxin 2 like	gene	biological_pr	G:9606:ATXN2L				ATXN2L	Homo sapier Gene Symbol	ATXN2L	FALSE	ATXN2L
taxon:9606	165631	1902	target of EGI	gene	biological_pr	G:9606:TOE1				TOE1	Homo sapier Gene Symbol	TOE1	FALSE	TOE1
taxon:9606	177919	1902	retinoid X re	gene	biological_pr	G:9606:RXRB			KEGG-&-1&-l	RXRB	Homo sapier Gene Symbol	RXRB	FALSE	RXRB
taxon:9606	173820	1902	DEAD-box he	gene	biological_pr	G:9606:DDX19B				DDX19B	Homo sapier Gene Symbol	DDX19B	FALSE	DDX19B
taxon:9606	169724	1902	DDB1 and CU	gene	biological_pr	G:9606:DCAF6				DCAF6	Homo sapier Gene Symbol	DCAF6	FALSE	DCAF6
taxon:9606	177917	1902	receptor-like	gene	biological_pr	G:9606:RYK			KEGG-&-1&-l	RYK	Homo sapier Gene Symbol	RYK	FALSE	RYK
taxon:9606	169730	1902	lysine demet	gene	biological_pr	G:9606:KDM3A			KEGG-&-1&-l	KDM3A	Homo sapier Gene Symbol	KDM3A	FALSE	KDM3A
taxon:9606	182018	1902	connective ti	gene	biological_pr	G:9606:CTGF			KEGG-&-1&-l	CTGF	Homo sapier Gene Symbol	CTGF	FALSE	CTGF
taxon:9606	177920	1902	retinoid X re	gene	biological_pr	G:9606:RXRA			KEGG-&-1&-l	RXRA	Homo sapier Gene Symbol	RXRA	FALSE	RXRA
taxon:9606	177924	1902	CAP-Gly dom	gene	biological_pr	G:9606:CLIP1			KEGG-&-1&-l	CLIP1	Homo sapier Gene Symbol	CLIP1	FALSE	CLIP1
taxon:9606	182021	1902	C-terminal bi	gene	biological_pr	G:9606:CTBP1			KEGG-&-1&-l	CTBP1	Homo sapier Gene Symbol	CTBP1	FALSE	CTBP1
taxon:9606	177930	1902	ribonucleotid	gene	biological_pr	G:9606:RRM2			KEGG-&-1&-l	RRM2	Homo sapier Gene Symbol	RRM2	FALSE	RRM2
taxon:9606	169737	1902	forkhead box	gene	biological_pr	G:9606:FOXJ2				FOXJ2	Homo sapier Gene Symbol	FOXJ2	FALSE	FOXJ2
taxon:9606	177932	1902	ras responsi	gene	biological_pr	G:9606:RREB1				RREB1	Homo sapier Gene Symbol	RREB1	FALSE	RREB1
taxon:9606	177938	1902	ribosomal pr	gene	biological_pr	G:9606:RPS27A			KEGG-&-1&-l	RPS27A	Homo sapier Gene Symbol	RPS27A	FALSE	RPS27A
taxon:9606	177939	1902	ribosomal pr	gene	biological_pr	G:9606:RPS27			KEGG-&-1&-l	RPS27	Homo sapier Gene Symbol	RPS27	FALSE	RPS27
taxon:9606	173840	1902	zinc fingers a	gene	biological_pr	G:9606:ZHX1				ZHX1	Homo sapier Gene Symbol	ZHX1	FALSE	ZHX1
taxon:9606	182032	1902	cystatin B	gene	biological_pr	G:9606:CSTB				CSTB	Homo sapier Gene Symbol	CSTB	FALSE	CSTB
taxon:9606	177937	1902	ribosomal pr	gene	biological_pr	G:9606:RPS28			KEGG-&-1&-l	RPS28	Homo sapier Gene Symbol	RPS28	FALSE	RPS28
taxon:9606	177942	1902	ribosomal pr	gene	biological_pr	G:9606:RPS24			KEGG-&-1&-l	RPS24	Homo sapier Gene Symbol	RPS24	FALSE	RPS24
taxon:9606	173846	1902	programmed	gene	biological_pr	G:9606:PDCD10				PDCD10	Homo sapier Gene Symbol	PDCD10	FALSE	PDCD10
taxon:9606	177940	1902	ribosomal pr	gene	biological_pr	G:9606:RPS26			KEGG-&-1&-l	RPS26	Homo sapier Gene Symbol	RPS26	FALSE	RPS26
taxon:9606	177941	1902	ribosomal pr	gene	biological_pr	G:9606:RPS25			KEGG-&-1&-l	RPS25	Homo sapier Gene Symbol	RPS25	FALSE	RPS25
taxon:9606	177947	1902	ribosomal pr	gene	biological_pr	G:9606:RPS20			KEGG-&-1&-l	RPS20	Homo sapier Gene Symbol	RPS20	FALSE	RPS20
taxon:9606	169752	1902	ligand depen	gene	biological_pr	G:9606:LRIF1				LRIF1	Homo sapier Gene Symbol	LRIF1	FALSE	LRIF1
taxon:9606	177944	1902	ribosomal pr	gene	biological_pr	G:9606:RPS21			KEGG-&-1&-l	RPS21	Homo sapier Gene Symbol	RPS21	FALSE	RPS21
taxon:9606	182046	1902	versican	gene	biological_pr	G:9606:VCAN			KEGG-&-1&-l	VCAN	Homo sapier Gene Symbol	VCAN	FALSE	VCAN
taxon:9606	182047	1902	casein kinase	gene	biological_pr	G:9606:CSNK2B			KEGG-&-1&-l	CSNK2B	Homo sapier Gene Symbol	CSNK2B	FALSE	CSNK2B
taxon:9606	177951	1902	ribosomal pr	gene	biological_pr	G:9606:RPS17			KEGG-&-1&-l	RPS17	Homo sapier Gene Symbol	RPS17	FALSE	RPS17
taxon:9606	177948	1902	ribosomal pr	gene	biological_pr	G:9606:RPS19			KEGG-&-1&-l	RPS19	Homo sapier Gene Symbol	RPS19	FALSE	RPS19
taxon:9606	177949	1902	ribosomal pr	gene	biological_pr	G:9606:RPS18			KEGG-&-1&-l	RPS18	Homo sapier Gene Symbol	RPS18	FALSE	RPS18
taxon:9606	177954	1902	ribosomal pr	gene	biological_pr	G:9606:RPS15			KEGG-&-1&-l	RPS15	Homo sapier Gene Symbol	RPS15	FALSE	RPS15
taxon:9606	177955	1902	ribosomal pr	gene	biological_pr	G:9606:RPS14			KEGG-&-1&-l	RPS14	Homo sapier Gene Symbol	RPS14	FALSE	RPS14
taxon:9606	177952	1902	ribosomal pr	gene	biological_pr	G:9606:RPS16			KEGG-&-1&-l	RPS16	Homo sapier Gene Symbol	RPS16	FALSE	RPS16
taxon:9606	182048	1902	casein kinase	gene	biological_pr	G:9606:CSNK2A2			KEGG-&-1&-l	CSNK2A2	Homo sapier Gene Symbol	CSNK2A2	FALSE	CSNK2A2
taxon:9606	182049	1902	casein kinase	gene	biological_pr	G:9606:CSNK2A1			KEGG-&-1&-l	CSNK2A1	Homo sapier Gene Symbol	CSNK2A1	FALSE	CSNK2A1
taxon:9606	169761	1902	RIO kinase 2	gene	biological_pr	G:9606:RIOK2			KEGG-&-1&-l	RIOK2	Homo sapier Gene Symbol	RIOK2	FALSE	RIOK2
taxon:9606	177953	1902	ribosomal pr	gene	biological_pr	G:9606:RPS15A			KEGG-&-1&-l	RPS15A	Homo sapier Gene Symbol	RPS15A	FALSE	RPS15A
taxon:9606	182054	1902	casein kinase	gene	biological_pr	G:9606:CSNK1A1			KEGG-&-1&-l	CSNK1A1	Homo sapier Gene Symbol	CSNK1A1	FALSE	CSNK1A1
taxon:9606	177958	1902	ribosomal pr	gene	biological_pr	G:9606:RPS11			KEGG-&-1&-l	RPS11	Homo sapier Gene Symbol	RPS11	FALSE	RPS11
taxon:9606	177959	1902	ribosomal pr	gene	biological_pr	G:9606:RPS10			KEGG-&-1&-l	RPS10	Homo sapier Gene Symbol	RPS10	FALSE	RPS10

taxon:9606	177956	1902	ribosomal pr	gene	biological_pr	G:9606:RPS13			KEGG-&-1&-	RPS13	Homo sapier	Gene Symbol	RPS13	FALSE	RPS13
taxon:9606	182052	1902	casein kinase	gene	biological_pr	G:9606:CSNK1E			KEGG-&-1&-	CSNK1E	Homo sapier	Gene Symbol	CSNK1E	FALSE	CSNK1E
taxon:9606	182053	1902	casein kinase	gene	biological_pr	G:9606:CSNK1D			KEGG-&-1&-	CSNK1D	Homo sapier	Gene Symbol	CSNK1D	FALSE	CSNK1D
taxon:9606	177957	1902	ribosomal pr	gene	biological_pr	G:9606:RPS12			KEGG-&-1&-	RPS12	Homo sapier	Gene Symbol	RPS12	FALSE	RPS12
taxon:9606	177962	1902	ribosomal pr	gene	biological_pr	G:9606:RPS7			KEGG-&-1&-	RPS7	Homo sapier	Gene Symbol	RPS7	FALSE	RPS7
taxon:9606	177960	1902	ribosomal pr	gene	biological_pr	G:9606:RPS9			KEGG-&-1&-	RPS9	Homo sapier	Gene Symbol	RPS9	FALSE	RPS9
taxon:9606	177961	1902	ribosomal pr	gene	biological_pr	G:9606:RPS8			KEGG-&-1&-	RPS8	Homo sapier	Gene Symbol	RPS8	FALSE	RPS8
taxon:9606	165677	1902	TP53 regulati	gene	biological_pr	G:9606:TP53RK				TP53RK	Homo sapier	Gene Symbol	TP53RK	FALSE	TP53RK
taxon:9606	177970	1902	ribosomal pr	gene	biological_pr	G:9606:RPS5			KEGG-&-1&-	RPS5	Homo sapier	Gene Symbol	RPS5	FALSE	RPS5
taxon:9606	173873	1902	proline rich c	gene	biological_pr	G:9606:PRRC2C				PRRC2C	Homo sapier	Gene Symbol	PRRC2C	FALSE	PRRC2C
taxon:9606	177969	1902	ribosomal pr	gene	biological_pr	G:9606:RPS6			KEGG-&-1&-	RPS6	Homo sapier	Gene Symbol	RPS6	FALSE	RPS6
taxon:9606	177974	1902	ribosomal pr	gene	biological_pr	G:9606:RPS3			KEGG-&-1&-	RPS3	Homo sapier	Gene Symbol	RPS3	FALSE	RPS3
taxon:9606	173878	1902	arginine dem	gene	biological_pr	G:9606:JMJD6				JMJD6	Homo sapier	Gene Symbol	JMJD6	FALSE	JMJD6
taxon:9606	182071	1902	mitogen-acti	gene	biological_pr	G:9606:MAPK14			KEGG-&-1&-	MAPK14	Homo sapier	Gene Symbol	MAPK14	FALSE	MAPK14
taxon:9606	177975	1902	ribosomal pr	gene	biological_pr	G:9606:RPS2			KEGG-&-1&-	RPS2	Homo sapier	Gene Symbol	RPS2	FALSE	RPS2
taxon:9606	177972	1902	ribosomal pr	gene	biological_pr	G:9606:RPS4X			KEGG-&-1&-	RPS4X	Homo sapier	Gene Symbol	RPS4X	FALSE	RPS4X
taxon:9606	182068	1902	colony stimul	gene	biological_pr	G:9606:CSF1			KEGG-&-1&-	CSF1	Homo sapier	Gene Symbol	CSF1	FALSE	CSF1
taxon:9606	177973	1902	ribosomal pr	gene	biological_pr	G:9606:RPS3A			KEGG-&-1&-	RPS3A	Homo sapier	Gene Symbol	RPS3A	FALSE	RPS3A
taxon:9606	182069	1902	chromosome	gene	biological_pr	G:9606:CSE1L				CSE1L	Homo sapier	Gene Symbol	CSE1L	FALSE	CSE1L
taxon:9606	169787	1902	septin 11	gene	biological_pr	G:9606:SEPT11			KEGG-&-1&-	Sep-11	Homo sapier	Gene Symbol	Sep-11	FALSE	Sep-11
taxon:9606	177977	1902	ribophorin I	gene	biological_pr	G:9606:RPN1			KEGG-&-1&-	RPN1	Homo sapier	Gene Symbol	RPN1	FALSE	RPN1
taxon:9606	169790	1902	cell division c	gene	biological_pr	G:9606:CCAR1				CCAR1	Homo sapier	Gene Symbol	CCAR1	FALSE	CCAR1
taxon:9606	169791	1902	CNDP dipepti	gene	biological_pr	G:9606:CNDP2			KEGG-&-1&-	CNDP2	Homo sapier	Gene Symbol	CNDP2	FALSE	CNDP2
taxon:9606	165698	1902	chromosome	gene	cellular_com	G:9606:C10orf90				C10orf90	Homo sapier	Gene Symbol	C10orf90	FALSE	C10orf90
taxon:9606	173890	1902	family with s	gene	cellular_com	G:9606:FAM120A				FAM120A	Homo sapier	Gene Symbol	FAM120A	FALSE	FAM120A
taxon:9606	173891	1902	midasin AAA	gene	biological_pr	G:9606:MDN1			KEGG-&-1&-	MDN1	Homo sapier	Gene Symbol	MDN1	FALSE	MDN1
taxon:9606	169795	1902	checkpoint w	gene	biological_pr	G:9606:CHFR				CHFR	Homo sapier	Gene Symbol	CHFR	FALSE	CHFR
taxon:9606	177984	1902	ring finger pr	gene	biological_pr	G:9606:RING1				RING1	Homo sapier	Gene Symbol	RING1	FALSE	RING1
taxon:9606	169798	1902	enabled hom	gene	biological_pr	G:9606:ENAH			KEGG-&-1&-	ENAH	Homo sapier	Gene Symbol	ENAH	FALSE	ENAH
taxon:9606	177990	1902	Ras homolog	gene	biological_pr	G:9606:RHEB			KEGG-&-1&-	RHEB	Homo sapier	Gene Symbol	RHEB	FALSE	RHEB
taxon:9606	182090	1902	crystallin alp	gene	biological_pr	G:9606:CRYAB			KEGG-&-1&-	CRYAB	Homo sapier	Gene Symbol	CRYAB	FALSE	CRYAB
taxon:9606	169801	1902	VPS35, retro	gene	biological_pr	G:9606:VPS35			KEGG-&-1&-	VPS35	Homo sapier	Gene Symbol	VPS35	FALSE	VPS35
taxon:9606	182095	1902	hyaluronan a	gene	biological_pr	G:9606:HAPLN1				HAPLN1	Homo sapier	Gene Symbol	HAPLN1	FALSE	HAPLN1
taxon:9606	169805	1902	chromosome	gene		G:9606:C1orf112				C1orf112	Homo sapier	Gene Symbol	C1orf112	FALSE	C1orf112
taxon:9606	173906	1902	DNA polyme	gene	biological_pr	G:9606:POLG2				POLG2	Homo sapier	Gene Symbol	POLG2	FALSE	POLG2
taxon:9606	182099	1902	CRK proto-on	gene	biological_pr	G:9606:CRK			KEGG-&-1&-	CRK	Homo sapier	Gene Symbol	CRK	FALSE	CRK
taxon:9606	165719	1902	ring finger ar	gene	biological_pr	G:9606:RFFL				RFFL	Homo sapier	Gene Symbol	RFFL	FALSE	RFFL
taxon:9606	145236	1902	COMMD3-BN	gene		G:9606:COMMD3-BMI1			KEGG-&-1&-	COMMD3-BN	Homo sapier	Gene Symbol	COMMD3-BN	FALSE	COMMD3-BMI1
taxon:9606	173912	1902	ribosomal pr	gene	biological_pr	G:9606:RPL35			KEGG-&-1&-	RPL35	Homo sapier	Gene Symbol	RPL35	FALSE	RPL35
taxon:9606	165721	1902	twist family	gene	biological_pr	G:9606:TWIST2			KEGG-&-1&-	TWIST2	Homo sapier	Gene Symbol	TWIST2	FALSE	TWIST2
taxon:9606	182110	1902	activating tra	gene	biological_pr	G:9606:ATF2			KEGG-&-1&-	ATF2	Homo sapier	Gene Symbol	ATF2	FALSE	ATF2
taxon:9606	182111	1902	cAMP respon	gene	biological_pr	G:9606:CREB1			KEGG-&-1&-	CREB1	Homo sapier	Gene Symbol	CREB1	FALSE	CREB1
taxon:9606	178015	1902	siah E3 ubiq	gene	biological_pr	G:9606:SIAH2				SIAH2	Homo sapier	Gene Symbol	SIAH2	FALSE	SIAH2
taxon:9606	182109	1902	CREB binding	gene	biological_pr	G:9606:CREBBP			KEGG-&-1&-	CREBBP	Homo sapier	Gene Symbol	CREBBP	FALSE	CREBBP
taxon:9606	169826	1902	importin 9	gene	biological_pr	G:9606:IPO9				IPO9	Homo sapier	Gene Symbol	IPO9	FALSE	IPO9
taxon:9606	182115	1902	complement	gene	biological_pr	G:9606:CR2			KEGG-&-1&-	CR2	Homo sapier	Gene Symbol	CR2	FALSE	CR2
taxon:9606	173921	1902	A-kinase anc	gene	biological_pr	G:9606:AKAP13			KEGG-&-1&-	AKAP13	Homo sapier	Gene Symbol	AKAP13	FALSE	AKAP13
taxon:9606	173930	1902	SPT16 homol	gene	biological_pr	G:9606:SUPT16H				SUPT16H	Homo sapier	Gene Symbol	SUPT16H	FALSE	SUPT16H
taxon:9606	178027	1902	SHC adaptor	gene	biological_pr	G:9606:SHC1			KEGG-&-1&-	SHC1	Homo sapier	Gene Symbol	SHC1	FALSE	SHC1
taxon:9606	173928	1902	checkpoint ki	gene	biological_pr	G:9606:CHEK2			KEGG-&-1&-	CHEK2	Homo sapier	Gene Symbol	CHEK2	FALSE	CHEK2
taxon:9606	178030	1902	SH2 domain	gene	biological_pr	G:9606:SHB				SHB	Homo sapier	Gene Symbol	SHB	FALSE	SHB
taxon:9606	169837	1902	lysine demet	gene	biological_pr	G:9606:KDM4D				KDM4D	Homo sapier	Gene Symbol	KDM4D	FALSE	KDM4D
taxon:9606	182130	1902	carboxypepti	gene	biological_pr	G:9606:CPD				CPD	Homo sapier	Gene Symbol	CPD	FALSE	CPD
taxon:9606	173939	1902	nischarin	gene	biological_pr	G:9606:NISCH				NISCH	Homo sapier	Gene Symbol	NISCH	FALSE	NISCH
taxon:9606	165744	1902	SSX family m	gene	biological_pr	G:9606:SSX2IP			KEGG-&-1&-	SSX2IP	Homo sapier	Gene Symbol	SSX2IP	FALSE	SSX2IP
taxon:9606	161648	1902	kallikrein rela	gene	biological_pr	G:9606:KLK9				KLK9	Homo sapier	Gene Symbol	KLK9	FALSE	KLK9
taxon:9606	182129	1902	carboxypepti	gene	biological_pr	G:9606:CPE			KEGG-&-1&-	CPE	Homo sapier	Gene Symbol	CPE	FALSE	CPE
taxon:9606	169844	1902	RAB, membe	gene	biological_pr	G:9606:RABL6				RABL6	Homo sapier	Gene Symbol	RABL6	FALSE	RABL6
taxon:9606	165748	1902	SH2 domain	gene	biological_pr	G:9606:SH2D1B			KEGG-&-1&-	SH2D1B	Homo sapier	Gene Symbol	SH2D1B	FALSE	SH2D1B
taxon:9606	165755	1902	transcription	gene	biological_pr	G:9606:TADA1				TADA1	Homo sapier	Gene Symbol	TADA1	FALSE	TADA1
taxon:9606	178040	1902	small glutam	gene	biological_pr	G:9606:SGTA				SGTA	Homo sapier	Gene Symbol	SGTA	FALSE	SGTA
taxon:9606	169852	1902	protein phos	gene	cellular_com	G:9606:PPP4R3A			KEGG-&-1&-	PPP4R3A	Homo sapier	Gene Symbol	PPP4R3A	FALSE	PPP4R3A
taxon:9606	173955	1902	serine/threo	gene	biological_pr	G:9606:STRAP			KEGG-&-1&-	STRAP	Homo sapier	Gene Symbol	STRAP	FALSE	STRAP
taxon:9606	165760	1902	ArfGAP with	gene	biological_pr	G:9606:ARAP1			KEGG-&-1&-	ARAP1	Homo sapier	Gene Symbol	ARAP1	FALSE	ARAP1
taxon:9606	178049	1902	tripartite mo	gene	biological_pr	G:9606:TRIM27				TRIM27	Homo sapier	Gene Symbol	TRIM27	FALSE	TRIM27
taxon:9606	178055	1902	replication fa	gene	biological_pr	G:9606:RFC1			KEGG-&-1&-	RFC1	Homo sapier	Gene Symbol	RFC1	FALSE	RFC1
taxon:9606	182151	1902	chloride volta	gene	biological_pr	G:9606:CLCN3				CLCN3	Homo sapier	Gene Symbol	CLCN3	FALSE	CLCN3
taxon:9606	178052	1902	replication fa	gene	biological_pr	G:9606:RFC4			KEGG-&-1&-	RFC4	Homo sapier	Gene Symbol	RFC4	FALSE	RFC4
taxon:9606	178053	1902	replication fa	gene	biological_pr	G:9606:RFC3			KEGG-&-1&-	RFC3	Homo sapier	Gene Symbol	RFC3	FALSE	RFC3
taxon:9606	165770	1902	mediator com	gene	biological_pr	G:9606:MED12L			KEGG-&-1&-	MED12L	Homo sapier	Gene Symbol	MED12L	FALSE	MED12L
taxon:9606	182154	1902	chloride chan	gene	biological_pr	G:9606:CLCA1			KEGG-&-1&-	CLCA1	Homo sapier	Gene Symbol	CLCA1	FALSE	CLCA1
taxon:9606	173966	1902	ER lipid raft a	gene	biological_pr	G:9606:ERLIN2				ERLIN2	Homo sapier	Gene Symbol	ERLIN2	FALSE	ERLIN2
taxon:9606	182159	1902	adaptor relat	gene	biological_pr	G:9606:AP2M1			KEGG-&-1&-	AP2M1	Homo sapier	Gene Symbol	AP2M1	FALSE	AP2M1
taxon:9606	178060	1902	UPF1, RNA h	gene	biological_pr	G:9606:UPF1			KEGG-&-1&-	UPF1	Homo sapier	Gene Symbol	UPF1	FALSE	UPF1
taxon:9606	182157	1902	adaptor relat	gene	biological_pr	G:9606:AP2S1			KEGG-&-1&-	AP2S1	Homo sapier	Gene Symbol	AP2S1	FALSE	AP2S1
taxon:9606	178065	1902	RELA proto-o	gene	biological_pr	G:9606:RELA			KEGG-&-1&-	RELA	Homo sapier	Gene Symbol	RELA	FALSE	RELA
taxon:9606	178070	1902	RecQ like hel	gene	biological_pr	G:9606:RECQL				RECQL	Homo sapier	Gene Symbol	RECQL	FALSE	RECQL
taxon:9606	182164	1902	ERCC excisio	gene	biological_pr	G:9606:ERCC8			KEGG-&-1&-	ERCC8	Homo sapier	Gene Symbol	ERCC8	FALSE	ERCC8
taxon:9606	169876	1902	O-sialoglycop	gene	biological_pr	G:9606:OSGEP				OSGEP	Homo sapier	Gene Symbol	OSGEP	FALSE	OSGEP
taxon:9606	169877	1902	BTB domain	gene	biological_pr	G:9606:BTBD2				BTBD2	Homo sapier	Gene Symbol	BTBD2	FALSE	BTBD2
taxon:9606	178069	1902	REL proto-on	gene	biological_pr	G:9606:REL			KEGG-&-1&-	REL	Homo sapier	Gene Symbol	REL	FALSE	REL
taxon:9606	169880	1902	chromodoma	gene	biological_pr	G:9606:CHD7				CHD7	Homo sapier	Gene Symbol	CHD7	FALSE	CHD7
taxon:9606	173982	1902	lysine acetylt	gene	biological_pr	G:9606:KAT7				KAT7	Homo sapier	Gene Symbol	KAT7	FALSE	KAT7
taxon:9606	178078	1902	reticulocalbi	gene	biological_pr	G:9606:RCN1				RCN1	Homo sapier	Gene Symbol	RCN1	FALSE	RCN1
taxon:9606	182175	1902	conserved he	gene	biological_pr	G:9606:CHUK			KEGG-&-1&-	CHUK	Homo sapier	Gene Symbol	CHUK	FALSE	CHUK
taxon:9606	169887	1902	proline rich n	gene	biological_pr	G:9606:PNRC2				PNRC2	Homo sapier	Gene Symbol	PNRC2	FALSE	PNRC2
taxon:9606	178077	1902	reticulocalbi	gene	cellular_com	G:9606:RCN2				RCN2	Homo sapier	Gene Symbol	RCN2	FALSE	RCN2
taxon:9606	182173	1902	creatine kina	gene	biological_pr	G:9606:CKB			KEGG-&-1&-	CKB	Homo sapier	Gene Symbol	CKB	FALSE	CKB
taxon:9606	178083	1902	retinol bindi	gene	biological_pr	G:9606:RBP1				RBP1	Homo sapier	Gene Symbol	RBP1	FALSE	RBP1
taxon:9606	178080	1902	retinol bindi	gene	biological_pr	G:9606:RBP4	RBP4	TRUE		RBP4	Homo sapier	Gene Symbol	RBP4	FALSE	RBP4
taxon:9606	173984	1902	interleukin 1	gene	biological_pr	G:9606:IL1RAPL1				IL1RAPL1	Homo sapier	Gene Symbol	IL1RAPL1	FALSE	IL1RAPL1
taxon:9606	173985	1902	cell division c	gene	biological_pr	G:9606:CDC37			KEGG-&-1&-	CDC37	Homo sapier	Gene Symbol	CDC37	FALSE	CDC37
taxon:9606	165796	1902	topoisomera	gene	biological_pr	G:9606:TOP1MT				TOP1MT	Homo sapier	Gene Symbol	TOP1MT	FALSE	TOP1MT
taxon:9606	178091	1902	RNA binding	gene	biological_pr	G:9606:RBM4				RBM4	Homo sapier	Gene Symbol	RBM4	FALSE	RBM4
taxon:9606	173993	1902	ZW10 intera	gene	biological_pr	G:9606:ZWINT				ZWINT	Homo sapier	Gene Symbol	ZWINT	FALSE	ZWINT
taxon:9606	178094	1902	RB transcript	gene	biological_pr	G:9606:RBL1			KEGG-&-1&-	RBL1	Homo sapier	Gene Symbol	RBL1	FALSE	RBL1
taxon:9606	178092	1902	RNA binding	gene	biological_pr	G:9606:RBM3				RBM3	Homo sapier	Gene Symbol	RBM3	FALSE	RBM3
taxon:9606	173996	1902	kinesin famil	gene	biological_pr	G:9606:KIF3A			KEGG-&-1&-	KIF3A	Homo sapier	Gene Symbol	KIF3A	FALSE	KIF3A
taxon:9606	165805	1902	solute carrie	gene	biological_pr	G:9606:SLC26A8				SLC26A8	Homo sapier	Gene Symbol	SLC26A8	FALSE	SLC26A8
taxon:9606	178098	1902	RB binding p	gene	biological_pr	G:9606:RBBP5			KEGG-&-1&-	RBBP5	Homo sapier	Gene Symbol	RBBP5	FALSE	RBBP5
taxon:9606	178099	1902	RB binding p	gene	biological_pr	G:9606:RBBP4			KEGG-&-1&-	RBBP4	Homo sapier	Gene Symbol	RBBP4	FALSE	RBBP4
taxon:9606	178096	1902	RB binding p	gene	biological_pr	G:9606:RBBP7				RBBP7	Homo sapier	Gene Symbol	RBBP7	FALSE	RBBP7
taxon:9606	178097	1902	RB binding p	gene	biological_pr	G:9606:RBBP6				RBBP6	Homo sapier	Gene Symbol	RBBP6	FALSE	RBBP6
taxon:9606	178102	1902	RB transcript	gene	biological_pr	G:9606:RB1			KEGG-&-1&-	RB1	Homo sapier	Gene Symbol	RB1	FALSE	RB1
taxon:9606	178100	1902	lysine demet	gene	biological_pr	G:9606:KDM5A				KDM5A	Homo sapier	Gene Symbol	KDM5A	FALSE	KDM5A
taxon:9606	174005	1902	FGFR1 oncog	gene	biological_pr	G:9606:FGFR1OP				FGFR1OP	Homo sapier	Gene Symbol	FGFR1OP	FALSE	FGFR1OP
taxon:9606	178106	1902	RAS p21 prot	gene	biological_pr	G:9606:RASA1			KEGG-&-1&-	RASA1	Homo sapier	Gene Symbol	RASA1	FALSE	RASA1
taxon:9606	178104	1902	Ras protein s	gene	biological_pr	G:9606:RASGRF1			KEGG-&-1&-	RASGRF1	Homo sapier	Gene Symbol	RASGRF1	FALSE	RASGRF1
taxon:9606	169913	1902	sphingomyel	gene	biological_pr	G:9606:SMPD3			KEGG-&-1&-	SMPD3	Homo sapier	Gene Symbol	SMPD3	FALSE	SMPD3
taxon:9606	165822	1902	retinol dehyd	gene	biological_pr	G:9606:RDH13				RDH13	Homo sapier	Gene Symbol	RDH13	FALSE	RDH13
taxon:9606	178110	1902	arginyl-tRNA	gene	biological_pr	G:9606:RARS			KEGG-&-1&-	RARS	Homo sapier	Gene Symbol	RARS	FALSE	RARS
taxon:9606	174015	1902	heterogeneo	gene	biological_pr	G:9606:HNRNPUL1			KEGG-&-1&-	HNRNPUL1	Homo sapier	Gene Symbol	HNRNPUL1	FALSE	HNRNPUL1
taxon:9606	182210	1902	chromodoma	gene	biological_pr	G:9606:CHD4			KEGG-&-1&-	CHD4	Homo sapier	Gene Symbol	CHD4	FALSE	CHD4
taxon:9606	182211	1902	chromodoma	gene	biological_pr	G:9606:CHD3				CHD3	Homo sapier	Gene Symbol	CHD3	FALSE	CHD3
taxon:9606	174019	1902	ADAM metal	gene	biological_pr	G:9606:ADAMTS5				ADAMTS5	Homo sapier	Gene Symbol	ADAMTS5	FALSE	ADAMTS5
taxon:9606	178115	1902	RAP2B, mem	gene	biological_pr	G:9606:RAP2B				RAP2B	Homo sapier	Gene Symbol	RAP2B	FALSE	RAP2B
taxon:9606	182208	1902	checkpoint ki	gene	biological_pr	G:9606:CHEK1			KEGG-&-1&-	CHEK1	Homo sapier	Gene Symbol	CHEK1	FALSE	CHEK1
taxon:9606	178113	1902	retinoic acid	gene	biological_pr	G:9606:RARA			KEGG-&-1&-	RARA	Homo sapier	Gene Symbol	RARA	FALSE	RARA
taxon:9606	182214	1902	regulator of c	gene	biological_pr	G:9606:RCC1				RCC1	Homo sapier	Gene Symbol	RCC1	FALSE	RCC1
taxon:9606	178119	1902	RAP1B, mem	gene	biological_pr	G:9606:RAP1B			KEGG-&-1&-	RAP1B	Homo sapier	Gene Symbol	RAP1B	FALSE	RAP1B
taxon:9606	178123	1902	RAN binding	gene	biological_pr	G:9606:RANBP2			KEGG-&-1&-	RANBP2	Homo sapier	Gene Symbol	RANBP2	FALSE	RANBP2
taxon:9606	174024	1902	WD repeat d	gene	biological_pr	G:9606:WDR5			KEGG-&-1&-	WDR5	Homo sapier	Gene Symbol	WDR5	FALSE	WDR5
taxon:9606	178121	1902	RAP1A, mem	gene	biological_pr	G:9606:RAP1A			KEGG-&-1&-	RAP1A	Homo sapier	Gene Symbol	RAP1A	FALSE	RAP1A
taxon:9606	178124	1902	RAN binding	gene	biological_pr	G:9606:RANBP1			KEGG-&-1&-	RANBP1	Homo sapier	Gene Symbol	RANBP1	FALSE	RANBP1
taxon:9606	178125	1902	RAN, membe	gene	biological_pr	G:9606:RAN			KEGG-&-1&-	RAN	Homo sapier	Gene Symbol	RAN	FALSE	RAN
taxon:9606	178131	1902	Raf-1 proto-c	gene	biological_pr	G:9606:RAF1			KEGG-&-1&-	RAF1	Homo sapier	Gene Symbol	RAF1	FALSE	RAF1
taxon:9606	178139	1902	RAD23 homo	gene	biological_pr	G:9606:RAD23A			KEGG-&-1&-	RAD23A	Homo sapier	Gene Symbol	RAD23A	FALSE	RAD23A
taxon:9606	178137	1902	RAD51 recom	gene	biological_pr	G:9606:RAD51			KEGG-&-1&-	RAD51	Homo sapier	Gene Symbol	RAD51	FALSE	RAD51
taxon:9606	178142	1902	RAD9 checkp	gene	biological_pr	G:9606:RAD9A			KEGG-&-1&-	RAD9A	Homo sapier	Gene Symbol	RAD9A	FALSE	RAD9A
taxon:9606	178143	1902	ras-related C	gene	biological_pr	G:9606:RAC3			KEGG-&-1&-	RAC3	Homo sapier	Gene Symbol	RAC3	FALSE	RAC3
taxon:9606	182236	1902	cystic fibrosi	gene	biological_pr	G:9606:CFTR	CFTR	TRUE	KEGG-&-1&-	CFTR	Homo sapier	Gene Symbol	CFTR	FALSE	CFTR
taxon:9606	165858	1902	tumor protei	gene	biological_pr	G:9606:TP53INP1				TP53INP1	Homo sapier	Gene Symbol	TP53INP1	FALSE	TP53INP1
taxon:9606	174050	1902	WW domain	gene	biological_pr	G:9606:WWP1			KEGG-&-1&-	WWP1	Homo sapier	Gene Symbol	WWP1	FALSE	WWP1
taxon:9606	178146	1902	RAB5C, mem	gene	biological_pr	G:9606:RAB5C			KEGG-&-1&-	RAB5C	Homo sapier	Gene Symbol	RAB5C	FALSE	RAB5C
taxon:9606	182243	1902	centrin 2	gene	biological_pr	G:9606:CETN2			KEGG-&-1&-	CETN2	Homo sapier	Gene Symbol	CETN2	FALSE	CETN2
taxon:9606	182240	1902	cofilin 1	gene	biological_pr	G:9606:CFL1			KEGG-&-1&-	CFL1	Homo sapier	Gene Symbol	CFL1	FALSE	CFL1
taxon:9606	169952	1902	erbb2 intera	gene	biological_pr	G:9606:ERBIN			KEGG-&-1&-	ERBIN	Homo sapier	Gene Symbol	ERBIN	FALSE	ERBIN
taxon:9606	178144	1902	ras-related C	gene	biological_pr	G:9606:RAC2			KEGG-&-1&-	RAC2	Homo sapier	Gene Symbol	RAC2	FALSE	RAC2
taxon:9606	178145	1902	ras-related C	gene	biological_pr	G:9606:RAC1			KEGG-&-1&-	RAC1	Homo sapier	Gene Symbol	RAC1	FALSE	RAC1
taxon:9606	169959	1902	lysine methyl	gene	biological_pr	G:9606:KMT2E			KEGG-&-1&-	KMT2E	Homo sapier	Gene Symbol	KMT2E	FALSE	KMT2E
taxon:9606	165863	1902	forkhead box	gene	biological_pr	G:9606:FOXQ1				FOXQ1	Homo sapier	Gene Symbol	FOXQ1	FALSE	FOXQ1
taxon:9606	182244	1902	centrin 1	gene	biological_pr	G:9606:CETN1				CETN1	Homo sapier	Gene Symbol	CETN1	FALSE	CETN1
taxon:9606	169962	1902	zinc finger pr	gene	biological_pr	G:9606:ZNF302				ZNF302	Homo sapier	Gene Symbol	ZNF302	FALSE	ZNF302
taxon:9606	182250	1902	centromere	gene	cellular_com	G:9606:CENPB				CENPB	Homo sapier	Gene Symbol	CENPB	FALSE	CENPB
taxon:9606	182251	1902	centromere	gene	biological_pr	G:9606:CENPA				CENPA	Homo sapier	Gene Symbol	CENPA	FALSE	CENPA
taxon:9606	174056	1902	nudix hydrola	gene	biological_pr	G:9606:NUDT21			KEGG-&-1&-	NUDT21	Homo sapier	Gene Symbol	NUDT21	FALSE	NUDT21
taxon:9606	178156	1902	RAB5A, mem	gene	biological_pr	G:9606:RAB5A			KEGG-&-1&-	RAB5A	Homo sapier	Gene Symbol	RAB5A	FALSE	RAB5A
taxon:9606	178157	1902	RAB4A, mem	gene	biological_pr	G:9606:RAB4A			KEGG-&-1&-	RAB4A	Homo sapier	Gene Symbol	RAB4A	FALSE	RAB4A
taxon:9606	182258	1902	CCAAT/enha	gene	biological_pr	G:9606:CEBPB			KEGG-&-1&-	CEBPB	Homo sapier	Gene Symbol	CEBPB	FALSE	CEBPB
taxon:9606	174066	1902	glucuronidas	gene		G:9606:SMA4				SMA4	Homo sapier	Gene Symbol	SMA4	FALSE	SMA4
taxon:9606	182259	1902	CCAAT/enha	gene	biological_pr	G:9606:CEBPA			KEGG-&-1&-	CEBPA	Homo sapier	Gene Symbol	CEBPA	FALSE	CEBPA
taxon:9606	178166	1902	PZP, alpha-2-	gene	biological_pr	G:9606:PZP				PZP	Homo sapier	Gene Symbol	PZP	FALSE	PZP
taxon:9606	178174	1902	paxillin	gene	biological_pr	G:9606:PXN			KEGG-&-1&-	PXN	Homo sapier	Gene Symbol	PXN	FALSE	PXN
taxon:9606	182275	1902	cyclin depen	gene	biological_pr	G:9606:CDKN2A			KEGG-&-1&-	CDKN2A	Homo sapier	Gene Symbol	CDKN2A	FALSE	CDKN2A
taxon:9606	182273	1902	cyclin depen	gene	biological_pr	G:9606:CDKN2C			KEGG-&-1&-	CDKN2C	Homo sapier	Gene Symbol	CDKN2C	FALSE	CDKN2C
taxon:9606	169985	1902	minichromos	gene	biological_pr	G:9606:MCM10				MCM10	Homo sapier	Gene Symbol	MCM10	FALSE	MCM10
taxon:9606	182278	1902	cyclin depen	gene	biological_pr	G:9606:CDKN1A			KEGG-&-1&-	CDKN1A	Homo sapier	Gene Symbol	CDKN1A	FALSE	CDKN1A
taxon:9606	182279	1902	cyclin depen	gene	biological_pr	G:9606:CDK9			KEGG-&-1&-	CDK9	Homo sapier	Gene Symbol	CDK9	FALSE	CDK9
taxon:9606	165892	1902	sideroflexin 5	gene	biological_pr	G:9606:SFXN5				SFXN5	Homo sapier	Gene Symbol	SFXN5	FALSE	SFXN5
taxon:9606	182282	1902	cyclin depen	gene	biological_pr	G:9606:CDK7			KEGG-&-1&-	CDK7	Homo sapier	Gene Symbol	CDK7	FALSE	CDK7
taxon:9606	182283	1902	cyclin depen	gene	biological_pr	G:9606:CDK6			KEGG-&-1&-	CDK6	Homo sapier	Gene Symbol	CDK6	FALSE	CDK6
taxon:9606	182280	1902	cyclin depen	gene	biological_pr	G:9606:CDK8				CDK8	Homo sapier	Gene Symbol	CDK8	FALSE	CDK8
taxon:9606	174088	1902	RuvB like AA	gene	biological_pr	G:9606:RUVBL2				RUVBL2	Homo sapier	Gene Symbol	RUVBL2	FALSE	RUVBL2
taxon:9606	169998	1902	phosphatidyl	gene	biological_pr	G:9606:PI4K2A			KEGG-&-1&-	PI4K2A	Homo sapier	Gene Symbol	PI4K2A	FALSE	PI4K2A
taxon:9606	182287	1902	cyclin depen	gene	biological_pr	G:9606:CDK2			KEGG-&-1&-	CDK2	Homo sapier	Gene Symbol	CDK2	FALSE	CDK2
taxon:9606	182284	1902	cyclin depen	gene	biological_pr	G:9606:CDK5			KEGG-&-1&-	CDK5	Homo sapier	Gene Symbol	CDK5	FALSE	CDK5
taxon:9606	174092	1902	protein phos	gene	biological_pr	G:9606:PPP1R13L				PPP1R13L	Homo sapier	Gene Symbol	PPP1R13L	FALSE	PPP1R13L
taxon:9606	174093	1902	Snf2 related	gene	biological_pr	G:9606:SRCAP				SRCAP	Homo sapier	Gene Symbol	SRCAP	FALSE	SRCAP
taxon:9606	178192	1902	protein tyros	gene	biological_pr	G:9606:PTPRZ1			KEGG-&-1&-	PTPRZ1	Homo sapier	Gene Symbol	PTPRZ1	FALSE	PTPRZ1
taxon:9606	178193	1902	protein tyros	gene	biological_pr	G:9606:PTPRS				PTPRS	Homo sapier	Gene Symbol	PTPRS	FALSE	PTPRS
taxon:9606	178196	1902	protein tyros	gene	biological_pr	G:9606:PTPRN2			KEGG-&-1&-	PTPRN2	Homo sapier	Gene Symbol	PTPRN2	FALSE	PTPRN2
taxon:9606	182306	1902	cell division c	gene	biological_pr	G:9606:CDC42			KEGG-&-1&-	CDC42	Homo sapier	Gene Symbol	CDC42	FALSE	CDC42
taxon:9606	170017	1902	WD repeat d	gene	biological_pr	G:9606:WDR33			KEGG-&-1&-	WDR33	Homo sapier	Gene Symbol	WDR33	FALSE	WDR33
taxon:9606	182305	1902	cadherin 1	gene	biological_pr	G:9606:CDH1			KEGG-&-1&-	CDH1	Homo sapier	Gene Symbol	CDH1	FALSE	CDH1
taxon:9606	182310	1902	cell division c	gene	biological_pr	G:9606:CDC25B			KEGG-&-1&-	CDC25B	Homo sapier	Gene Symbol	CDC25B	FALSE	CDC25B
taxon:9606	182311	1902	cell division c	gene	biological_pr	G:9606:CDC25A			KEGG-&-1&-	CDC25A	Homo sapier	Gene Symbol	CDC25A	FALSE	CDC25A
taxon:9606	182309	1902	cell division c	gene	biological_pr	G:9606:CDC25C			KEGG-&-1&-	CDC25C	Homo sapier	Gene Symbol	CDC25C	FALSE	CDC25C
taxon:9606	178213	1902	protein tyros	gene	biological_pr	G:9606:PTPN11			KEGG-&-1&-	PTPN11	Homo sapier	Gene Symbol	PTPN11	FALSE	PTPN11
taxon:9606	182315	1902	cell division c	gene	biological_pr	G:9606:CDC5L			KEGG-&-1&-	CDC5L	Homo sapier	Gene Symbol	CDC5L	FALSE	CDC5L
taxon:9606	182318	1902	cyclin depen	gene	biological_pr	G:9606:CDK1			KEGG-&-1&-	CDK1	Homo sapier	Gene Symbol	CDK1	FALSE	CDK1
taxon:9606	174126	1902	zinc finger ar	gene	biological_pr	G:9606:ZBTB1				ZBTB1	Homo sapier	Gene Symbol	ZBTB1	FALSE	ZBTB1
taxon:9606	165932	1902	calcium depe	gene	biological_pr	G:9606:CADPS2				CADPS2	Homo sapier	Gene Symbol	CADPS2	FALSE	CADPS2
taxon:9606	170029	1902	lin-7 homolo	gene	biological_pr	G:9606:LIN7C				LIN7C	Homo sapier	Gene Symbol	LIN7C	FALSE	LIN7C
taxon:9606	178226	1902	parathymosi	gene	biological_pr	G:9606:PTMS				PTMS	Homo sapier	Gene Symbol	PTMS	FALSE	PTMS
taxon:9606	174129	1902	forkhead box	gene	biological_pr	G:9606:FOXI3				FOXI3	Homo sapier	Gene Symbol	FOXI3	FALSE	FOXI3
taxon:9606	178231	1902	phosphoryla	gene	biological_pr	G:9606:PHKG2			KEGG-&-1&-	PHKG2	Homo sapier	Gene Symbol	PHKG2	FALSE	PHKG2
taxon:9606	165943	1902	transcription	gene	biological_pr	G:9606:TADA2B				TADA2B	Homo sapier	Gene Symbol	TADA2B	FALSE	TADA2B
taxon:9606	178235	1902	phosphoryla	gene	biological_pr	G:9606:PHKB			KEGG-&-1&-	PHKB	Homo sapier	Gene Symbol	PHKB	FALSE	PHKB
taxon:9606	178239	1902	PHD finger p	gene	biological_pr	G:9606:PHF1				PHF1	Homo sapier	Gene Symbol	PHF1	FALSE	PHF1
taxon:9606	174143	1902	SEC31 homol	gene	biological_pr	G:9606:SEC31A			KEGG-&-1&-	SEC31A	Homo sapier	Gene Symbol	SEC31A	FALSE	SEC31A
taxon:9606	178236	1902	phosphoryla	gene	biological_pr	G:9606:PHKA2			KEGG-&-1&-	PHKA2	Homo sapier	Gene Symbol	PHKA2	FALSE	PHKA2
taxon:9606	182333	1902	CD59 molecu	gene	biological_pr	G:9606:CD59			KEGG-&-1&-	CD59	Homo sapier	Gene Symbol	CD59	FALSE	CD59
taxon:9606	174141	1902	pleckstrin ho	gene		G:9606:PLEKHA6				PLEKHA6	Homo sapier	Gene Symbol	PLEKHA6	FALSE	PLEKHA6
taxon:9606	182338	1902	kinesin famil	gene	biological_pr	G:9606:KIF1A				KIF1A	Homo sapier	Gene Symbol	KIF1A	FALSE	KIF1A
taxon:9606	182339	1902	ATRX, chrom	gene	biological_pr	G:9606:ATRX				ATRX	Homo sapier	Gene Symbol	ATRX	FALSE	ATRX
taxon:9606	178243	1902	prohibitin	gene	biological_pr	G:9606:PHB				PHB	Homo sapier	Gene Symbol	PHB	FALSE	PHB
taxon:9606	178246	1902	progesterone	gene	biological_pr	G:9606:PGR			KEGG-&-1&-	PGR	Homo sapier	Gene Symbol	PGR	FALSE	PGR
taxon:9606	182340	1902	ATR serine/t	gene	biological_pr	G:9606:ATR			KEGG-&-1&-	ATR	Homo sapier	Gene Symbol	ATR	FALSE	ATR
taxon:9606	182346	1902	ATP synthase	gene	biological_pr	G:9606:ATP5O			KEGG-&-1&-	ATP5O	Homo sapier	Gene Symbol	ATP5O	FALSE	ATP5O
taxon:9606	174155	1902	intestinal cell	gene	biological_pr	G:9606:ICK				ICK	Homo sapier	Gene Symbol	ICK	FALSE	ICK
taxon:9606	178255	1902	placental gro	gene	biological_pr	G:9606:PGF			KEGG-&-1&-	PGF	Homo sapier	Gene Symbol	PGF	FALSE	PGF
taxon:9606	170060	1902	ras homolog	gene	biological_pr	G:9606:RHOT1			KEGG-&-1&-	RHOT1	Homo sapier	Gene Symbol	RHOT1	FALSE	RHOT1
taxon:9606	178263	1902	profilin 1	gene	biological_pr	G:9606:PFN1			KEGG-&-1&-	PFN1	Homo sapier	Gene Symbol	PFN1	FALSE	PFN1
taxon:9606	182359	1902	ATP synthase	gene	biological_pr	G:9606:ATP5I			KEGG-&-1&-	ATP5I	Homo sapier	Gene Symbol	ATP5I	FALSE	ATP5I
taxon:9606	178266	1902	ribosomal pr	gene	biological_pr	G:9606:RPLP2			KEGG-&-1&-	RPLP2	Homo sapier	Gene Symbol	RPLP2	FALSE	RPLP2
taxon:9606	170078	1902	paraspeckle	gene	biological_pr	G:9606:PSPC1				PSPC1	Homo sapier	Gene Symbol	PSPC1	FALSE	PSPC1
taxon:9606	182367	1902	ATP synthase	gene	biological_pr	G:9606:ATP5C1			KEGG-&-1&-	ATP5C1	Homo sapier	Gene Symbol	ATP5C1	FALSE	ATP5C1
taxon:9606	178268	1902	ribosomal pr	gene	biological_pr	G:9606:RPLP0			KEGG-&-1&-	RPLP0	Homo sapier	Gene Symbol	RPLP0	FALSE	RPLP0
taxon:9606	178274	1902	ribosomal pr	gene	biological_pr	G:9606:RPL38			KEGG-&-1&-	RPL38	Homo sapier	Gene Symbol	RPL38	FALSE	RPL38
taxon:9606	178275	1902	ribosomal pr	gene	biological_pr	G:9606:RPL37A			KEGG-&-1&-	RPL37A	Homo sapier	Gene Symbol	RPL37A	FALSE	RPL37A
taxon:9606	182369	1902	ATP synthase	gene	biological_pr	G:9606:ATP5B			KEGG-&-1&-	ATP5B	Homo sapier	Gene Symbol	ATP5B	FALSE	ATP5B
taxon:9606	182375	1902	ATPase plas	gene	biological_pr	G:9606:ATP2B4			KEGG-&-1&-	ATP2B4	Homo sapier	Gene Symbol	ATP2B4	FALSE	ATP2B4
taxon:9606	182372	1902	ATP synthase	gene	biological_pr	G:9606:ATP5A1			KEGG-&-1&-	ATP5A1	Homo sapier	Gene Symbol	ATP5A1	FALSE	ATP5A1
taxon:9606	178277	1902	ribosomal pr	gene	biological_pr	G:9606:RPL36AL			KEGG-&-1&-	RPL36AL	Homo sapier	Gene Symbol	RPL36AL	FALSE	RPL36AL
taxon:9606	178282	1902	ribosomal pr	gene	biological_pr	G:9606:RPL31			KEGG-&-1&-	RPL31	Homo sapier	Gene Symbol	RPL31	FALSE	RPL31
taxon:9606	182376	1902	ATPase plas	gene	biological_pr	G:9606:ATP2B3			KEGG-&-1&-	ATP2B3	Homo sapier	Gene Symbol	ATP2B3	FALSE	ATP2B3
taxon:9606	178286	1902	ribosomal pr	gene	biological_pr	G:9606:RPL30			KEGG-&-1&-	RPL30	Homo sapier	Gene Symbol	RPL30	FALSE	RPL30
taxon:9606	157806	1902	BH3-like mot	gene	biological_pr	G:9606:BLID				BLID	Homo sapier	Gene Symbol	BLID	FALSE	BLID
taxon:9606	178287	1902	ribosomal pr	gene	biological_pr	G:9606:RPL27			KEGG-&-1&-	RPL27	Homo sapier	Gene Symbol	RPL27	FALSE	RPL27
taxon:9606	178284	1902	ribosomal pr	gene	biological_pr	G:9606:RPL28			KEGG-&-1&-	RPL28	Homo sapier	Gene Symbol	RPL28	FALSE	RPL28
taxon:9606	182380	1902	ATPase sarc	gene	biological_pr	G:9606:ATP2A2			KEGG-&-1&-	ATP2A2	Homo sapier	Gene Symbol	ATP2A2	FALSE	ATP2A2
taxon:9606	182381	1902	ATPase sarc	gene	biological_pr	G:9606:ATP2A1			KEGG-&-1&-	ATP2A1	Homo sapier	Gene Symbol	ATP2A1	FALSE	ATP2A1
taxon:9606	178285	1902	ribosomal pr	gene	biological_pr	G:9606:RPL27A			KEGG-&-1&-	RPL27A	Homo sapier	Gene Symbol	RPL27A	FALSE	RPL27A
taxon:9606	182387	1902	ATPase Na+/	gene	biological_pr	G:9606:ATP1B1			KEGG-&-1&-	ATP1B1	Homo sapier	Gene Symbol	ATP1B1	FALSE	ATP1B1
taxon:9606	170096	1902	nei like DNA	gene	biological_pr	G:9606:NEIL3			KEGG-&-1&-	NEIL3	Homo sapier	Gene Symbol	NEIL3	FALSE	NEIL3
taxon:9606	178288	1902	ribosomal pr	gene	biological_pr	G:9606:RPL26			KEGG-&-1&-	RPL26	Homo sapier	Gene Symbol	RPL26	FALSE	RPL26
taxon:9606	178289	1902	ribosomal pr	gene	biological_pr	G:9606:RPL24			KEGG-&-1&-	RPL24	Homo sapier	Gene Symbol	RPL24	FALSE	RPL24
taxon:9606	178295	1902	ribosomal pr	gene	biological_pr	G:9606:RPL21			KEGG-&-1&-	RPL21	Homo sapier	Gene Symbol	RPL21	FALSE	RPL21
taxon:9606	178292	1902	ribosomal pr	gene	biological_pr	G:9606:RPL23A			KEGG-&-1&-	RPL23A	Homo sapier	Gene Symbol	RPL23A	FALSE	RPL23A
taxon:9606	182389	1902	ATPase H+/K	gene	biological_pr	G:9606:ATP12A			KEGG-&-1&-	ATP12A	Homo sapier	Gene Symbol	ATP12A	FALSE	ATP12A
taxon:9606	178293	1902	ribosomal pr	gene	biological_pr	G:9606:RPL22			KEGG-&-1&-	RPL22	Homo sapier	Gene Symbol	RPL22	FALSE	RPL22
taxon:9606	178298	1902	ribosomal pr	gene	biological_pr	G:9606:RPL18			KEGG-&-1&-	RPL18	Homo sapier	Gene Symbol	RPL18	FALSE	RPL18
taxon:9606	166011	1902	RAS like fam	gene	biological_pr	G:9606:RASL10B				RASL10B	Homo sapier	Gene Symbol	RASL10B	FALSE	RASL10B
taxon:9606	178299	1902	ribosomal pr	gene	biological_pr	G:9606:RPL17			KEGG-&-1&-	RPL17	Homo sapier	Gene Symbol	RPL17	FALSE	RPL17
taxon:9606	178296	1902	ribosomal pr	gene	biological_pr	G:9606:RPL19			KEGG-&-1&-	RPL19	Homo sapier	Gene Symbol	RPL19	FALSE	RPL19
taxon:9606	178297	1902	ribosomal pr	gene	biological_pr	G:9606:RPL18A			KEGG-&-1&-	RPL18A	Homo sapier	Gene Symbol	RPL18A	FALSE	RPL18A
taxon:9606	178302	1902	ribosomal pr	gene	biological_pr	G:9606:RPL12			KEGG-&-1&-	RPL12	Homo sapier	Gene Symbol	RPL12	FALSE	RPL12
taxon:9606	178303	1902	ribosomal pr	gene	biological_pr	G:9606:RPL11			KEGG-&-1&-	RPL11	Homo sapier	Gene Symbol	RPL11	FALSE	RPL11
taxon:9606	166015	1902	ribosomal pr	gene	cellular_com	G:9606:RPS19BP1				RPS19BP1	Homo sapier	Gene Symbol	RPS19BP1	FALSE	RPS19BP1
taxon:9606	182396	1902	ATM serine/t	gene	biological_pr	G:9606:ATM			KEGG-&-1&-	ATM	Homo sapier	Gene Symbol	ATM	FALSE	ATM
taxon:9606	178300	1902	ribosomal pr	gene	biological_pr	G:9606:RPL15			KEGG-&-1&-	RPL15	Homo sapier	Gene Symbol	RPL15	FALSE	RPL15
taxon:9606	178301	1902	ribosomal pr	gene	biological_pr	G:9606:RPL13			KEGG-&-1&-	RPL13	Homo sapier	Gene Symbol	RPL13	FALSE	RPL13
taxon:9606	174205	1902	cancer susce	gene	biological_pr	G:9606:CASC3			KEGG-&-1&-	CASC3	Homo sapier	Gene Symbol	CASC3	FALSE	CASC3
taxon:9606	161922	1902	casein kinase	gene		G:9606:CSNK2A3			KEGG-&-1&-	CSNK2A3	Homo sapier	Gene Symbol	CSNK2A3	FALSE	CSNK2A3
taxon:9606	178306	1902	ribosomal pr	gene	biological_pr	G:9606:RPL8			KEGG-&-1&-	RPL8	Homo sapier	Gene Symbol	RPL8	FALSE	RPL8
taxon:9606	178307	1902	ribosomal pr	gene	biological_pr	G:9606:RPL7A			KEGG-&-1&-	RPL7A	Homo sapier	Gene Symbol	RPL7A	FALSE	RPL7A
taxon:9606	178304	1902	ribosomal pr	gene	biological_pr	G:9606:RPL10			KEGG-&-1&-	RPL10	Homo sapier	Gene Symbol	RPL10	FALSE	RPL10
taxon:9606	170113	1902	N-acetyltrans	gene	biological_pr	G:9606:NAT10			KEGG-&-1&-	NAT10	Homo sapier	Gene Symbol	NAT10	FALSE	NAT10
taxon:9606	182401	1902	activating tra	gene	biological_pr	G:9606:ATF3				ATF3	Homo sapier	Gene Symbol	ATF3	FALSE	ATF3
taxon:9606	178305	1902	ribosomal pr	gene	biological_pr	G:9606:RPL9			KEGG-&-1&-	RPL9	Homo sapier	Gene Symbol	RPL9	FALSE	RPL9
taxon:9606	178311	1902	ribosomal pr	gene	biological_pr	G:9606:RPL5			KEGG-&-1&-	RPL5	Homo sapier	Gene Symbol	RPL5	FALSE	RPL5
taxon:9606	178308	1902	ribosomal pr	gene	biological_pr	G:9606:RPL7			KEGG-&-1&-	RPL7	Homo sapier	Gene Symbol	RPL7	FALSE	RPL7
taxon:9606	174212	1902	exosome com	gene	biological_pr	G:9606:EXOSC8			KEGG-&-1&-	EXOSC8	Homo sapier	Gene Symbol	EXOSC8	FALSE	EXOSC8
taxon:9606	178309	1902	ribosomal pr	gene	biological_pr	G:9606:RPL6			KEGG-&-1&-	RPL6	Homo sapier	Gene Symbol	RPL6	FALSE	RPL6
taxon:9606	178314	1902	ribosomal pr	gene	biological_pr	G:9606:RPL3			KEGG-&-1&-	RPL3	Homo sapier	Gene Symbol	RPL3	FALSE	RPL3
taxon:9606	178315	1902	RPE65, retin	gene	biological_pr	G:9606:RPE65			KEGG-&-1&-	RPE65	Homo sapier	Gene Symbol	RPE65	FALSE	RPE65
taxon:9606	178312	1902	ribosomal pr	gene	biological_pr	G:9606:RPL4			KEGG-&-1&-	RPL4	Homo sapier	Gene Symbol	RPL4	FALSE	RPL4
taxon:9606	178318	1902	replication p	gene	biological_pr	G:9606:RPA2			KEGG-&-1&-	RPA2	Homo sapier	Gene Symbol	RPA2	FALSE	RPA2
taxon:9606	178319	1902	replication p	gene	biological_pr	G:9606:RPA1			KEGG-&-1&-	RPA1	Homo sapier	Gene Symbol	RPA1	FALSE	RPA1
taxon:9606	166028	1902	ring finger pr	gene	biological_pr	G:9606:RNF185			KEGG-&-1&-	RNF185	Homo sapier	Gene Symbol	RNF185	FALSE	RNF185
taxon:9606	178317	1902	replication p	gene	biological_pr	G:9606:RPA3			KEGG-&-1&-	RPA3	Homo sapier	Gene Symbol	RPA3	FALSE	RPA3
taxon:9606	174221	1902	prohibitin 2	gene	biological_pr	G:9606:PHB2				PHB2	Homo sapier	Gene Symbol	PHB2	FALSE	PHB2
taxon:9606	182419	1902	asparagine s	gene	biological_pr	G:9606:ASNS			KEGG-&-1&-	ASNS	Homo sapier	Gene Symbol	ASNS	FALSE	ASNS
taxon:9606	170128	1902	ATPase famil	gene	biological_pr	G:9606:ATAD3A				ATAD3A	Homo sapier	Gene Symbol	ATAD3A	FALSE	ATAD3A
taxon:9606	174230	1902	ecdysoneless	gene	biological_pr	G:9606:ECD				ECD	Homo sapier	Gene Symbol	ECD	FALSE	ECD
taxon:9606	174234	1902	Parkinsonism	gene	biological_pr	G:9606:PARK7			KEGG-&-1&-	PARK7	Homo sapier	Gene Symbol	PARK7	FALSE	PARK7
taxon:9606	170140	1902	regulation of	gene	biological_pr	G:9606:RPRD1A				RPRD1A	Homo sapier	Gene Symbol	RPRD1A	FALSE	RPRD1A
taxon:9606	174236	1902	lysophosphol	gene	biological_pr	G:9606:LYPLA2			KEGG-&-1&-	LYPLA2	Homo sapier	Gene Symbol	LYPLA2	FALSE	LYPLA2
taxon:9606	174240	1902	polynucleotic	gene	biological_pr	G:9606:PNKP				PNKP	Homo sapier	Gene Symbol	PNKP	FALSE	PNKP
taxon:9606	166055	1902	transmembr	gene	biological_pr	G:9606:TMEM259				TMEM259	Homo sapier	Gene Symbol	TMEM259	FALSE	TMEM259
taxon:9606	174245	1902	Kruppel like f	gene	biological_pr	G:9606:KLF8				KLF8	Homo sapier	Gene Symbol	KLF8	FALSE	KLF8
taxon:9606	174251	1902	UTP14A sma	gene	biological_pr	G:9606:UTP14A			KEGG-&-1&-	UTP14A	Homo sapier	Gene Symbol	UTP14A	FALSE	UTP14A
taxon:9606	182446	1902	arrestin beta	gene	biological_pr	G:9606:ARRB1			KEGG-&-1&-	ARRB1	Homo sapier	Gene Symbol	ARRB1	FALSE	ARRB1
taxon:9606	174255	1902	heat shock p	gene	biological_pr	G:9606:HSPH1			KEGG-&-1&-	HSPH1	Homo sapier	Gene Symbol	HSPH1	FALSE	HSPH1
taxon:9606	182445	1902	arrestin beta	gene	biological_pr	G:9606:ARRB2			KEGG-&-1&-	ARRB2	Homo sapier	Gene Symbol	ARRB2	FALSE	ARRB2
taxon:9606	182450	1902	ADP ribosylat	gene	biological_pr	G:9606:ARL3				ARL3	Homo sapier	Gene Symbol	ARL3	FALSE	ARL3
taxon:9606	182448	1902	aryl hydrocar	gene	biological_pr	G:9606:ARNTL			KEGG-&-1&-	ARNTL	Homo sapier	Gene Symbol	ARNTL	FALSE	ARNTL
taxon:9606	174261	1902	septin 9	gene	biological_pr	G:9606:SEPT9			KEGG-&-1&-	Sep-09	Homo sapier	Gene Symbol	Sep-09	FALSE	Sep-09
taxon:9606	178360	1902	ATP binding c	gene	biological_pr	G:9606:ABCE1				ABCE1	Homo sapier	Gene Symbol	ABCE1	FALSE	ABCE1
taxon:9606	178367	1902	ribonuclease,	gene	biological_pr	G:9606:RNH1				RNH1	Homo sapier	Gene Symbol	RNH1	FALSE	RNH1
taxon:9606	178370	1902	ring finger pr	gene	biological_pr	G:9606:RNF4				RNF4	Homo sapier	Gene Symbol	RNF4	FALSE	RNF4
taxon:9606	178371	1902	bromodomai	gene	biological_pr	G:9606:BRD2				BRD2	Homo sapier	Gene Symbol	BRD2	FALSE	BRD2
taxon:9606	178368	1902	ring finger pr	gene	biological_pr	G:9606:RNF6				RNF6	Homo sapier	Gene Symbol	RNF6	FALSE	RNF6
taxon:9606	178369	1902	ring finger pr	gene	biological_pr	G:9606:RNF5			KEGG-&-1&-	RNF5	Homo sapier	Gene Symbol	RNF5	FALSE	RNF5
taxon:9606	178375	1902	ribonuclease	gene	biological_pr	G:9606:RNASEL			KEGG-&-1&-	RNASEL	Homo sapier	Gene Symbol	RNASEL	FALSE	RNASEL
taxon:9606	178372	1902	ring finger pr	gene	biological_pr	G:9606:RNF2				RNF2	Homo sapier	Gene Symbol	RNF2	FALSE	RNF2
taxon:9606	182475	1902	ADP ribosylat	gene	biological_pr	G:9606:ARF1			KEGG-&-1&-	ARF1	Homo sapier	Gene Symbol	ARF1	FALSE	ARF1
taxon:9606	174287	1902	zinc finger M	gene	biological_pr	G:9606:ZMYND11				ZMYND11	Homo sapier	Gene Symbol	ZMYND11	FALSE	ZMYND11
taxon:9606	170191	1902	OTU deubiqu	gene	biological_pr	G:9606:OTUB1				OTUB1	Homo sapier	Gene Symbol	OTUB1	FALSE	OTUB1
taxon:9606	182476	1902	amphiregulin	gene	biological_pr	G:9606:AREG			KEGG-&-1&-	AREG	Homo sapier	Gene Symbol	AREG	FALSE	AREG
taxon:9606	182481	1902	androgen rec	gene	biological_pr	G:9606:AR	AR	TRUE	KEGG-&-1&-	AR	Homo sapier	Gene Symbol	AR	FALSE	AR
taxon:9606	174292	1902	lysine demet	gene	biological_pr	G:9606:KDM5B				KDM5B	Homo sapier	Gene Symbol	KDM5B	FALSE	KDM5B
taxon:9606	170204	1902	OTU deubiqu	gene	biological_pr	G:9606:OTUD5			KEGG-&-1&-	OTUD5	Homo sapier	Gene Symbol	OTUD5	FALSE	OTUD5
taxon:9606	182492	1902	Fas cell surfa	gene	biological_pr	G:9606:FAS			KEGG-&-1&-	FAS	Homo sapier	Gene Symbol	FAS	FALSE	FAS
taxon:9606	170210	1902	ubiquitin con	gene	biological_pr	G:9606:UBE2Q1			KEGG-&-1&-	UBE2Q1	Homo sapier	Gene Symbol	UBE2Q1	FALSE	UBE2Q1
taxon:9606	182497	1902	adenylate cyc	gene	biological_pr	G:9606:ADCY8			KEGG-&-1&-	ADCY8	Homo sapier	Gene Symbol	ADCY8	FALSE	ADCY8
taxon:9606	162025	1902	MAGE family	gene	biological_pr	G:9606:MAGEA2B				MAGEA2B	Homo sapier	Gene Symbol	MAGEA2B	FALSE	MAGEA2B
taxon:9606	182510	1902	adenosine de	gene	biological_pr	G:9606:ADA			KEGG-&-1&-	ADA	Homo sapier	Gene Symbol	ADA	FALSE	ADA
taxon:9606	178415	1902	peptidase D	gene	biological_pr	G:9606:PEPD				PEPD	Homo sapier	Gene Symbol	PEPD	FALSE	PEPD
taxon:9606	182508	1902	ADAM metal	gene	biological_pr	G:9606:ADAM10			KEGG-&-1&-	ADAM10	Homo sapier	Gene Symbol	ADAM10	FALSE	ADAM10
taxon:9606	174321	1902	prostaglandi	gene	biological_pr	G:9606:PTGES3			KEGG-&-1&-	PTGES3	Homo sapier	Gene Symbol	PTGES3	FALSE	PTGES3
taxon:9606	178423	1902	PDZ domain c	gene	biological_pr	G:9606:PDZK1				PDZK1	Homo sapier	Gene Symbol	PDZK1	FALSE	PDZK1
taxon:9606	182522	1902	actin like 6A	gene	biological_pr	G:9606:ACTL6A			KEGG-&-1&-	ACTL6A	Homo sapier	Gene Symbol	ACTL6A	FALSE	ACTL6A
taxon:9606	182520	1902	actinin alpha	gene	biological_pr	G:9606:ACTN2			KEGG-&-1&-	ACTN2	Homo sapier	Gene Symbol	ACTN2	FALSE	ACTN2
taxon:9606	170233	1902	BTG3 associa	gene	biological_pr	G:9606:BANP				BANP	Homo sapier	Gene Symbol	BANP	FALSE	BANP
taxon:9606	174333	1902	ubiquitin spe	gene	biological_pr	G:9606:USP39			KEGG-&-1&-	USP39	Homo sapier	Gene Symbol	USP39	FALSE	USP39
taxon:9606	166141	1902	GADD45G int	gene	biological_pr	G:9606:GADD45GIP1				GADD45GIP1	Homo sapier	Gene Symbol	GADD45GIP1	FALSE	GADD45GIP1
taxon:9606	182525	1902	actinin alpha	gene	biological_pr	G:9606:ACTN4			KEGG-&-1&-	ACTN4	Homo sapier	Gene Symbol	ACTN4	FALSE	ACTN4
taxon:9606	174337	1902	chaperonin c	gene	biological_pr	G:9606:CCT8				CCT8	Homo sapier	Gene Symbol	CCT8	FALSE	CCT8
taxon:9606	182542	1902	actin beta	gene	biological_pr	G:9606:ACTB			KEGG-&-1&-	ACTB	Homo sapier	Gene Symbol	ACTB	FALSE	ACTB
taxon:9606	182543	1902	actin, alpha 2	gene	biological_pr	G:9606:ACTA2			KEGG-&-1&-	ACTA2	Homo sapier	Gene Symbol	ACTA2	FALSE	ACTA2
taxon:9606	182544	1902	actin, alpha 1	gene	biological_pr	G:9606:ACTA1				ACTA1	Homo sapier	Gene Symbol	ACTA1	FALSE	ACTA1
taxon:9606	178448	1902	phosphodiest	gene	biological_pr	G:9606:PDE6H			KEGG-&-1&-	PDE6H	Homo sapier	Gene Symbol	PDE6H	FALSE	PDE6H
taxon:9606	170260	1902	ring finger pr	gene	biological_pr	G:9606:RNF125			KEGG-&-1&-	RNF125	Homo sapier	Gene Symbol	RNF125	FALSE	RNF125
taxon:9606	182548	1902	acid phospha	gene	biological_pr	G:9606:ACP2			KEGG-&-1&-	ACP2	Homo sapier	Gene Symbol	ACP2	FALSE	ACP2
taxon:9606	182549	1902	acid phospha	gene	biological_pr	G:9606:ACP1			KEGG-&-1&-	ACP1	Homo sapier	Gene Symbol	ACP1	FALSE	ACP1
taxon:9606	157977	1902	H3 histone, f	gene		G:9606:H3F3AP4				H3F3AP4	Homo sapier	Gene Symbol	H3F3AP4	FALSE	H3F3AP4
taxon:9606	174361	1902	CCCTC-bindin	gene	biological_pr	G:9606:CTCF				CTCF	Homo sapier	Gene Symbol	CTCF	FALSE	CTCF
taxon:9606	170270	1902	tRNA methyl	gene	biological_pr	G:9606:TRMT10C				TRMT10C	Homo sapier	Gene Symbol	TRMT10C	FALSE	TRMT10C
taxon:9606	166173	1902	OTU deubiqu	gene	biological_pr	G:9606:OTULIN				OTULIN	Homo sapier	Gene Symbol	OTULIN	FALSE	OTULIN
taxon:9606	182564	1902	cytochrome c	gene	biological_pr	G:9606:COX5B			KEGG-&-1&-	COX5B	Homo sapier	Gene Symbol	COX5B	FALSE	COX5B
taxon:9606	166186	1902	heparan sulfi	gene	biological_pr	G:9606:HS6ST2			KEGG-&-1&-	HS6ST2	Homo sapier	Gene Symbol	HS6ST2	FALSE	HS6ST2
taxon:9606	174379	1902	insulin like g	gene	biological_pr	G:9606:IGF2BP3				IGF2BP3	Homo sapier	Gene Symbol	IGF2BP3	FALSE	IGF2BP3
taxon:9606	178478	1902	proliferating	gene	biological_pr	G:9606:PCNA			KEGG-&-1&-	PCNA	Homo sapier	Gene Symbol	PCNA	FALSE	PCNA
taxon:9606	182574	1902	coatomer pr	gene	biological_pr	G:9606:COPA				COPA	Homo sapier	Gene Symbol	COPA	FALSE	COPA
taxon:9606	178479	1902	protein-L-iso	gene	biological_pr	G:9606:PCMT1				PCMT1	Homo sapier	Gene Symbol	PCMT1	FALSE	PCMT1
taxon:9606	170287	1902	semaphorin	gene	biological_pr	G:9606:SEMA4C			KEGG-&-1&-	SEMA4C	Homo sapier	Gene Symbol	SEMA4C	FALSE	SEMA4C
taxon:9606	182572	1902	Kruppel like f	gene	biological_pr	G:9606:KLF6				KLF6	Homo sapier	Gene Symbol	KLF6	FALSE	KLF6
taxon:9606	170284	1902	YTH N6-meth	gene	biological_pr	G:9606:YTHDF1				YTHDF1	Homo sapier	Gene Symbol	YTHDF1	FALSE	YTHDF1
taxon:9606	174380	1902	insulin like g	gene	biological_pr	G:9606:IGF2BP1			KEGG-&-1&-	IGF2BP1	Homo sapier	Gene Symbol	IGF2BP1	FALSE	IGF2BP1
taxon:9606	182573	1902	coatomer pr	gene	biological_pr	G:9606:COPB1				COPB1	Homo sapier	Gene Symbol	COPB1	FALSE	COPB1
taxon:9606	170288	1902	spindle appa	gene	biological_pr	G:9606:SPDL1				SPDL1	Homo sapier	Gene Symbol	SPDL1	FALSE	SPDL1
taxon:9606	178486	1902	protocadheri	gene	biological_pr	G:9606:PCDH7				PCDH7	Homo sapier	Gene Symbol	PCDH7	FALSE	PCDH7
taxon:9606	174394	1902	thioredoxin i	gene	biological_pr	G:9606:TXNIP			KEGG-&-1&-	TXNIP	Homo sapier	Gene Symbol	TXNIP	FALSE	TXNIP
taxon:9606	170298	1902	castor zinc fi	gene	biological_pr	G:9606:CASZ1				CASZ1	Homo sapier	Gene Symbol	CASZ1	FALSE	CASZ1
taxon:9606	178491	1902	poly(rC) bind	gene	biological_pr	G:9606:PCBP2			KEGG-&-1&-	PCBP2	Homo sapier	Gene Symbol	PCBP2	FALSE	PCBP2
taxon:9606	182591	1902	collagen type	gene	biological_pr	G:9606:COL7A1			KEGG-&-1&-	COL7A1	Homo sapier	Gene Symbol	COL7A1	FALSE	COL7A1
taxon:9606	170300	1902	ring finger pr	gene	biological_pr	G:9606:RNF43			KEGG-&-1&-	RNF43	Homo sapier	Gene Symbol	RNF43	FALSE	RNF43
taxon:9606	178492	1902	poly(rC) bind	gene	biological_pr	G:9606:PCBP1			KEGG-&-1&-	PCBP1	Homo sapier	Gene Symbol	PCBP1	FALSE	PCBP1
taxon:9606	174397	1902	influenza vir	gene	biological_pr	G:9606:IVNS1ABP			KEGG-&-1&-	IVNS1ABP	Homo sapier	Gene Symbol	IVNS1ABP	FALSE	IVNS1ABP
taxon:9606	178498	1902	PBX homeob	gene	biological_pr	G:9606:PBX1			KEGG-&-1&-	PBX1	Homo sapier	Gene Symbol	PBX1	FALSE	PBX1
taxon:9606	182592	1902	collagen type	gene	biological_pr	G:9606:COL6A3			KEGG-&-1&-	COL6A3	Homo sapier	Gene Symbol	COL6A3	FALSE	COL6A3
taxon:9606	170305	1902	NOP2/Sun RI	gene	biological_pr	G:9606:NSUN2				NSUN2	Homo sapier	Gene Symbol	NSUN2	FALSE	NSUN2
taxon:9606	174406	1902	hexamethyle	gene	biological_pr	G:9606:HEXIM1				HEXIM1	Homo sapier	Gene Symbol	HEXIM1	FALSE	HEXIM1
taxon:9606	182598	1902	collagen type	gene	biological_pr	G:9606:COL4A5			KEGG-&-1&-	COL4A5	Homo sapier	Gene Symbol	COL4A5	FALSE	COL4A5
taxon:9606	178503	1902	paired box 6	gene	biological_pr	G:9606:PAX6			KEGG-&-1&-	PAX6	Homo sapier	Gene Symbol	PAX6	FALSE	PAX6
taxon:9606	182599	1902	collagen type	gene	biological_pr	G:9606:COL4A4			KEGG-&-1&-	COL4A4	Homo sapier	Gene Symbol	COL4A4	FALSE	COL4A4
taxon:9606	174407	1902	ER lipid raft a	gene	biological_pr	G:9606:ERLIN1				ERLIN1	Homo sapier	Gene Symbol	ERLIN1	FALSE	ERLIN1
taxon:9606	174404	1902	RANBP2-type	gene	biological_pr	G:9606:RBCK1			KEGG-&-1&-	RBCK1	Homo sapier	Gene Symbol	RBCK1	FALSE	RBCK1
taxon:9606	182597	1902	collagen type	gene	biological_pr	G:9606:COL4A6			KEGG-&-1&-	COL4A6	Homo sapier	Gene Symbol	COL4A6	FALSE	COL4A6
taxon:9606	182603	1902	collagen type	gene	biological_pr	G:9606:COL4A1			KEGG-&-1&-	COL4A1	Homo sapier	Gene Symbol	COL4A1	FALSE	COL4A1
taxon:9606	182600	1902	collagen type	gene	biological_pr	G:9606:COL4A3			KEGG-&-1&-	COL4A3	Homo sapier	Gene Symbol	COL4A3	FALSE	COL4A3
taxon:9606	182601	1902	collagen type	gene	biological_pr	G:9606:COL4A2			KEGG-&-1&-	COL4A2	Homo sapier	Gene Symbol	COL4A2	FALSE	COL4A2
taxon:9606	182607	1902	collagen type	gene	biological_pr	G:9606:COL1A2			KEGG-&-1&-	COL1A2	Homo sapier	Gene Symbol	COL1A2	FALSE	COL1A2
taxon:9606	182605	1902	collagen type	gene	biological_pr	G:9606:COL2A1			KEGG-&-1&-	COL2A1	Homo sapier	Gene Symbol	COL2A1	FALSE	COL2A1
taxon:9606	182608	1902	collagen type	gene	biological_pr	G:9606:COL1A1			KEGG-&-1&-	COL1A1	Homo sapier	Gene Symbol	COL1A1	FALSE	COL1A1
taxon:9606	174423	1902	pre-mRNA p	gene	biological_pr	G:9606:PRPF8			KEGG-&-1&-	PRPF8	Homo sapier	Gene Symbol	PRPF8	FALSE	PRPF8
taxon:9606	174420	1902	activator of h	gene	biological_pr	G:9606:AHSA1				AHSA1	Homo sapier	Gene Symbol	AHSA1	FALSE	AHSA1
taxon:9606	178522	1902	serpin family	gene	biological_pr	G:9606:SERPINE1			KEGG-&-1&-	SERPINE1	Homo sapier	Gene Symbol	SERPINE1	FALSE	SERPINE1
taxon:9606	182619	1902	polo like kina	gene	biological_pr	G:9606:PLK3			KEGG-&-1&-	PLK3	Homo sapier	Gene Symbol	PLK3	FALSE	PLK3
taxon:9606	178526	1902	platelet activ	gene	biological_pr	G:9606:PAFAH1B3			KEGG-&-1&-	PAFAH1B3	Homo sapier	Gene Symbol	PAFAH1B3	FALSE	PAFAH1B3
taxon:9606	178524	1902	peroxiredoxi	gene	biological_pr	G:9606:PRDX1			KEGG-&-1&-	PRDX1	Homo sapier	Gene Symbol	PRDX1	FALSE	PRDX1
taxon:9606	178538	1902	prolyl 4-hydr	gene	biological_pr	G:9606:P4HA1			KEGG-&-1&-	P4HA1	Homo sapier	Gene Symbol	P4HA1	FALSE	P4HA1
taxon:9606	174443	1902	LanC like 1	gene	biological_pr	G:9606:LANCL1				LANCL1	Homo sapier	Gene Symbol	LANCL1	FALSE	LANCL1
taxon:9606	174440	1902	TNFAIP3 inte	gene	biological_pr	G:9606:TNIP1				TNIP1	Homo sapier	Gene Symbol	TNIP1	FALSE	TNIP1
taxon:9606	178536	1902	proliferation	gene	biological_pr	G:9606:PA2G4				PA2G4	Homo sapier	Gene Symbol	PA2G4	FALSE	PA2G4
taxon:9606	170349	1902	aprataxin	gene	biological_pr	G:9606:APTX				APTX	Homo sapier	Gene Symbol	APTX	FALSE	APTX
taxon:9606	178550	1902	oxytocin/neu	gene	biological_pr	G:9606:OXT			KEGG-&-1&-	OXT	Homo sapier	Gene Symbol	OXT	FALSE	OXT
taxon:9606	182647	1902	clathrin heav	gene	biological_pr	G:9606:CLTC			KEGG-&-1&-	CLTC	Homo sapier	Gene Symbol	CLTC	FALSE	CLTC
taxon:9606	182648	1902	clathrin light	gene	biological_pr	G:9606:CLTB			KEGG-&-1&-	CLTB	Homo sapier	Gene Symbol	CLTB	FALSE	CLTB
taxon:9606	182649	1902	clathrin light	gene	biological_pr	G:9606:CLTA			KEGG-&-1&-	CLTA	Homo sapier	Gene Symbol	CLTA	FALSE	CLTA
taxon:9606	170366	1902	zinc finger C	gene	molecular_fu	G:9606:ZCCHC10				ZCCHC10	Homo sapier	Gene Symbol	ZCCHC10	FALSE	ZCCHC10
taxon:9606	182654	1902	ceroid-lipofu	gene	biological_pr	G:9606:CLN5			KEGG-&-1&-	CLN5	Homo sapier	Gene Symbol	CLN5	FALSE	CLN5
taxon:9606	174462	1902	splicing fact	gene	biological_pr	G:9606:SF3A1			KEGG-&-1&-	SF3A1	Homo sapier	Gene Symbol	SF3A1	FALSE	SF3A1
taxon:9606	174460	1902	DnaJ heat sh	gene	biological_pr	G:9606:DNAJA2			KEGG-&-1&-	DNAJA2	Homo sapier	Gene Symbol	DNAJA2	FALSE	DNAJA2
taxon:9606	170365	1902	nudE neurod	gene	biological_pr	G:9606:NDE1				NDE1	Homo sapier	Gene Symbol	NDE1	FALSE	NDE1
taxon:9606	182657	1902	CDC like kina	gene	biological_pr	G:9606:CLK3				CLK3	Homo sapier	Gene Symbol	CLK3	FALSE	CLK3
taxon:9606	182662	1902	chloride intra	gene	biological_pr	G:9606:CLIC1				CLIC1	Homo sapier	Gene Symbol	CLIC1	FALSE	CLIC1
taxon:9606	182663	1902	clusterin	gene	biological_pr	G:9606:CLU			KEGG-&-1&-	CLU	Homo sapier	Gene Symbol	CLU	FALSE	CLU
taxon:9606	174469	1902	interleukin 2	gene	biological_pr	G:9606:IL24			KEGG-&-1&-	IL24	Homo sapier	Gene Symbol	IL24	FALSE	IL24
taxon:9606	182666	1902	bystin like	gene	biological_pr	G:9606:BYSL				BYSL	Homo sapier	Gene Symbol	BYSL	FALSE	BYSL
taxon:9606	182667	1902	BUB1 mitoti	gene	biological_pr	G:9606:BUB1B			KEGG-&-1&-	BUB1B	Homo sapier	Gene Symbol	BUB1B	FALSE	BUB1B
taxon:9606	166280	1902	translocase	gene	biological_pr	G:9606:TIMM50				TIMM50	Homo sapier	Gene Symbol	TIMM50	FALSE	TIMM50
taxon:9606	174483	1902	inner membr	gene	biological_pr	G:9606:IMMT				IMMT	Homo sapier	Gene Symbol	IMMT	FALSE	IMMT
taxon:9606	174480	1902	splicing fact	gene	biological_pr	G:9606:SF3B2			KEGG-&-1&-	SF3B2	Homo sapier	Gene Symbol	SF3B2	FALSE	SF3B2
taxon:9606	182672	1902	Bruton tyros	gene	biological_pr	G:9606:BTK			KEGG-&-1&-	BTK	Homo sapier	Gene Symbol	BTK	FALSE	BTK
taxon:9606	174487	1902	GCN1, eIF2 a	gene	biological_pr	G:9606:GCN1				GCN1	Homo sapier	Gene Symbol	GCN1	FALSE	GCN1
taxon:9606	174485	1902	COP9 signalo	gene	biological_pr	G:9606:COPS5				COPS5	Homo sapier	Gene Symbol	COPS5	FALSE	COPS5
taxon:9606	182677	1902	Kruppel like f	gene	biological_pr	G:9606:KLF5				KLF5	Homo sapier	Gene Symbol	KLF5	FALSE	KLF5
taxon:9606	182680	1902	betacellulin	gene	biological_pr	G:9606:BTC			KEGG-&-1&-	BTC	Homo sapier	Gene Symbol	BTC	FALSE	BTC
taxon:9606	174492	1902	COP9 signalo	gene	biological_pr	G:9606:COPS6				COPS6	Homo sapier	Gene Symbol	COPS6	FALSE	COPS6
taxon:9606	182690	1902	BRCA1, DNA	gene	biological_pr	G:9606:BRCA1			KEGG-&-1&-	BRCA1	Homo sapier	Gene Symbol	BRCA1	FALSE	BRCA1
taxon:9606	174499	1902	tyrosine 3-m	gene	biological_pr	G:9606:YWHAQ			KEGG-&-1&-	YWHAQ	Homo sapier	Gene Symbol	YWHAQ	FALSE	YWHAQ
taxon:9606	182688	1902	BRCA2, DNA	gene	biological_pr	G:9606:BRCA2			KEGG-&-1&-	BRCA2	Homo sapier	Gene Symbol	BRCA2	FALSE	BRCA2
taxon:9606	182694	1902	forkhead box	gene	biological_pr	G:9606:FOXL2				FOXL2	Homo sapier	Gene Symbol	FOXL2	FALSE	FOXL2
taxon:9606	174500	1902	cytoskeleton	gene	biological_pr	G:9606:CKAP4			KEGG-&-1&-	CKAP4	Homo sapier	Gene Symbol	CKAP4	FALSE	CKAP4
taxon:9606	182692	1902	biphenyl hyd	gene	biological_pr	G:9606:BPHL				BPHL	Homo sapier	Gene Symbol	BPHL	FALSE	BPHL
taxon:9606	174501	1902	EBNA1 bindi	gene	biological_pr	G:9606:EBNA1BP2				EBNA1BP2	Homo sapier	Gene Symbol	EBNA1BP2	FALSE	EBNA1BP2
taxon:9606	174505	1902	stress induce	gene	biological_pr	G:9606:STIP1			KEGG-&-1&-	STIP1	Homo sapier	Gene Symbol	STIP1	FALSE	STIP1
taxon:9606	182702	1902	BMX non-rec	gene	biological_pr	G:9606:BMX				BMX	Homo sapier	Gene Symbol	BMX	FALSE	BMX
taxon:9606	174510	1902	proline rich n	gene	biological_pr	G:9606:PNRC1				PNRC1	Homo sapier	Gene Symbol	PNRC1	FALSE	PNRC1
taxon:9606	178607	1902	protein tyros	gene	biological_pr	G:9606:PTK6				PTK6	Homo sapier	Gene Symbol	PTK6	FALSE	PTK6
taxon:9606	170418	1902	FXYD domai	gene	biological_pr	G:9606:FXYD6				FXYD6	Homo sapier	Gene Symbol	FXYD6	FALSE	FXYD6
taxon:9606	174514	1902	translocase	gene	biological_pr	G:9606:TOMM34				TOMM34	Homo sapier	Gene Symbol	TOMM34	FALSE	TOMM34
taxon:9606	178608	1902	protein tyros	gene	biological_pr	G:9606:PTK2			KEGG-&-1&-	PTK2	Homo sapier	Gene Symbol	PTK2	FALSE	PTK2
taxon:9606	174518	1902	heterogeneo	gene	biological_pr	G:9606:HNRNPA0				HNRNPA0	Homo sapier	Gene Symbol	HNRNPA0	FALSE	HNRNPA0
taxon:9606	178612	1902	prostaglandi	gene	biological_pr	G:9606:PTGS2			KEGG-&-1&-	PTGS2	Homo sapier	Gene Symbol	PTGS2	FALSE	PTGS2
taxon:9606	182714	1902	BMI1 proto-	gene	biological_pr	G:9606:BMI1			KEGG-&-1&-	BMI1	Homo sapier	Gene Symbol	BMI1	FALSE	BMI1
taxon:9606	182713	1902	bone morph	gene	biological_pr	G:9606:BMP1				BMP1	Homo sapier	Gene Symbol	BMP1	FALSE	BMP1
taxon:9606	174527	1902	POP1 homol	gene	biological_pr	G:9606:POP1			KEGG-&-1&-	POP1	Homo sapier	Gene Symbol	POP1	FALSE	POP1
taxon:9606	178627	1902	phosphatase	gene	biological_pr	G:9606:PTEN			KEGG-&-1&-	PTEN	Homo sapier	Gene Symbol	PTEN	FALSE	PTEN
taxon:9606	174531	1902	peroxiredoxi	gene	biological_pr	G:9606:PRDX3				PRDX3	Homo sapier	Gene Symbol	PRDX3	FALSE	PRDX3
taxon:9606	182720	1902	Bloom syndr	gene	biological_pr	G:9606:BLM			KEGG-&-1&-	BLM	Homo sapier	Gene Symbol	BLM	FALSE	BLM
taxon:9606	182721	1902	BLK proto-on	gene	biological_pr	G:9606:BLK				BLK	Homo sapier	Gene Symbol	BLK	FALSE	BLK
taxon:9606	178630	1902	polypyrimidi	gene	biological_pr	G:9606:PTBP1				PTBP1	Homo sapier	Gene Symbol	PTBP1	FALSE	PTBP1
taxon:9606	170439	1902	SHC adaptor	gene	biological_pr	G:9606:SHC3			KEGG-&-1&-	SHC3	Homo sapier	Gene Symbol	SHC3	FALSE	SHC3
taxon:9606	174536	1902	ralA binding	gene	biological_pr	G:9606:RALBP1			KEGG-&-1&-	RALBP1	Homo sapier	Gene Symbol	RALBP1	FALSE	RALBP1
taxon:9606	182728	1902	biglycan	gene	biological_pr	G:9606:BGN				BGN	Homo sapier	Gene Symbol	BGN	FALSE	BGN
taxon:9606	178633	1902	proteasome	gene	biological_pr	G:9606:PSME2			KEGG-&-1&-	PSME2	Homo sapier	Gene Symbol	PSME2	FALSE	PSME2
taxon:9606	178638	1902	proteasome	gene	biological_pr	G:9606:PSMD10				PSMD10	Homo sapier	Gene Symbol	PSMD10	FALSE	PSMD10
taxon:9606	182734	1902	bradykinin re	gene	biological_pr	G:9606:BDKRB2			KEGG-&-1&-	BDKRB2	Homo sapier	Gene Symbol	BDKRB2	FALSE	BDKRB2
taxon:9606	174543	1902	COP9 signalo	gene	biological_pr	G:9606:COPS8				COPS8	Homo sapier	Gene Symbol	COPS8	FALSE	COPS8
taxon:9606	166348	1902	tetratricopep	gene	biological_pr	G:9606:TTC5				TTC5	Homo sapier	Gene Symbol	TTC5	FALSE	TTC5
taxon:9606	182733	1902	brain derived	gene	biological_pr	G:9606:BDNF	BDNF	TRUE	KEGG-&-1&-	BDNF	Homo sapier	Gene Symbol	BDNF	FALSE	BDNF
taxon:9606	178637	1902	proteasome	gene	biological_pr	G:9606:PSMD11			KEGG-&-1&-	PSMD11	Homo sapier	Gene Symbol	PSMD11	FALSE	PSMD11
taxon:9606	174546	1902	MAGE family	gene	biological_pr	G:9606:MAGED2				MAGED2	Homo sapier	Gene Symbol	MAGED2	FALSE	MAGED2
taxon:9606	178643	1902	proteasome	gene	biological_pr	G:9606:PSMD4			KEGG-&-1&-	PSMD4	Homo sapier	Gene Symbol	PSMD4	FALSE	PSMD4
taxon:9606	174544	1902	euchromatic	gene	biological_pr	G:9606:EHMT2			KEGG-&-1&-	EHMT2	Homo sapier	Gene Symbol	EHMT2	FALSE	EHMT2
taxon:9606	182736	1902	3-hydroxybut	gene	biological_pr	G:9606:BDH1			KEGG-&-1&-	BDH1	Homo sapier	Gene Symbol	BDH1	FALSE	BDH1
taxon:9606	166353	1902	WD repeat d	gene	biological_pr	G:9606:WDR20				WDR20	Homo sapier	Gene Symbol	WDR20	FALSE	WDR20
taxon:9606	182742	1902	BCR, RhoGEF	gene	biological_pr	G:9606:BCR			KEGG-&-1&-	BCR	Homo sapier	Gene Symbol	BCR	FALSE	BCR
taxon:9606	166359	1902	Cdk5 and Abl	gene	biological_pr	G:9606:CABLES1				CABLES1	Homo sapier	Gene Symbol	CABLES1	FALSE	CABLES1
taxon:9606	174548	1902	poly(A) polym	gene	biological_pr	G:9606:PAPOLA			KEGG-&-1&-	PAPOLA	Homo sapier	Gene Symbol	PAPOLA	FALSE	PAPOLA
taxon:9606	178644	1902	proteasome	gene	biological_pr	G:9606:PSMD3			KEGG-&-1&-	PSMD3	Homo sapier	Gene Symbol	PSMD3	FALSE	PSMD3
taxon:9606	178645	1902	proteasome	gene	biological_pr	G:9606:PSMD2			KEGG-&-1&-	PSMD2	Homo sapier	Gene Symbol	PSMD2	FALSE	PSMD2
taxon:9606	170453	1902	Sec61 transl	gene	biological_pr	G:9606:SEC61A2			KEGG-&-1&-	SEC61A2	Homo sapier	Gene Symbol	SEC61A2	FALSE	SEC61A2
taxon:9606	178651	1902	proteasome	gene	biological_pr	G:9606:PSMC3			KEGG-&-1&-	PSMC3	Homo sapier	Gene Symbol	PSMC3	FALSE	PSMC3
taxon:9606	178648	1902	proteasome	gene	biological_pr	G:9606:PSMC5			KEGG-&-1&-	PSMC5	Homo sapier	Gene Symbol	PSMC5	FALSE	PSMC5
taxon:9606	178649	1902	proteasome	gene	biological_pr	G:9606:PSMC4			KEGG-&-1&-	PSMC4	Homo sapier	Gene Symbol	PSMC4	FALSE	PSMC4
taxon:9606	170463	1902	centrosomal	gene	biological_pr	G:9606:CEP55				CEP55	Homo sapier	Gene Symbol	CEP55	FALSE	CEP55
taxon:9606	174559	1902	bromodomai	gene	biological_pr	G:9606:BRD8				BRD8	Homo sapier	Gene Symbol	BRD8	FALSE	BRD8
taxon:9606	170461	1902	male-specific	gene	biological_pr	G:9606:MSL2				MSL2	Homo sapier	Gene Symbol	MSL2	FALSE	MSL2
taxon:9606	182749	1902	B-cell CLL/ly	gene	biological_pr	G:9606:BCL6			KEGG-&-1&-	BCL6	Homo sapier	Gene Symbol	BCL6	FALSE	BCL6
taxon:9606	182754	1902	BCL2 like 2	gene	biological_pr	G:9606:BCL2L2			KEGG-&-1&-	BCL2L2	Homo sapier	Gene Symbol	BCL2L2	FALSE	BCL2L2
taxon:9606	182755	1902	kallikrein rela	gene	biological_pr	G:9606:KLK3			KEGG-&-1&-	KLK3	Homo sapier	Gene Symbol	KLK3	FALSE	KLK3
taxon:9606	178659	1902	proteasome	gene	biological_pr	G:9606:PSMB6			KEGG-&-1&-	PSMB6	Homo sapier	Gene Symbol	PSMB6	FALSE	PSMB6
taxon:9606	174560	1902	dehydrogena	gene	biological_pr	G:9606:DHRS4			KEGG-&-1&-	DHRS4	Homo sapier	Gene Symbol	DHRS4	FALSE	DHRS4
taxon:9606	182758	1902	apolipoprote	gene	biological_pr	G:9606:APOH			KEGG-&-1&-	APOH	Homo sapier	Gene Symbol	APOH	FALSE	APOH
taxon:9606	178662	1902	proteasome	gene	biological_pr	G:9606:PSMB3			KEGG-&-1&-	PSMB3	Homo sapier	Gene Symbol	PSMB3	FALSE	PSMB3
taxon:9606	182759	1902	apolipoprote	gene	biological_pr	G:9606:APOE			KEGG-&-1&-	APOE	Homo sapier	Gene Symbol	APOE	FALSE	APOE
taxon:9606	174567	1902	lymphatic ve	gene	biological_pr	G:9606:LYVE1				LYVE1	Homo sapier	Gene Symbol	LYVE1	FALSE	LYVE1
taxon:9606	178663	1902	proteasome	gene	biological_pr	G:9606:PSMB2			KEGG-&-1&-	PSMB2	Homo sapier	Gene Symbol	PSMB2	FALSE	PSMB2
taxon:9606	170468	1902	ring finger ar	gene	biological_pr	G:9606:RFWD3				RFWD3	Homo sapier	Gene Symbol	RFWD3	FALSE	RFWD3
taxon:9606	182756	1902	adenine phos	gene	biological_pr	G:9606:APRT			KEGG-&-1&-	APRT	Homo sapier	Gene Symbol	APRT	FALSE	APRT
taxon:9606	178661	1902	proteasome	gene	biological_pr	G:9606:PSMB4			KEGG-&-1&-	PSMB4	Homo sapier	Gene Symbol	PSMB4	FALSE	PSMB4
taxon:9606	182757	1902	amyloid beta	gene	biological_pr	G:9606:APP			KEGG-&-1&-	APP	Homo sapier	Gene Symbol	APP	FALSE	APP
taxon:9606	174570	1902	PPARG coact	gene	biological_pr	G:9606:PPARGC1A			KEGG-&-1&-	PPARGC1A	Homo sapier	Gene Symbol	PPARGC1A	FALSE	PPARGC1A
taxon:9606	178666	1902	proteasome	gene	biological_pr	G:9606:PSMA6			KEGG-&-1&-	PSMA6	Homo sapier	Gene Symbol	PSMA6	FALSE	PSMA6
taxon:9606	178667	1902	proteasome	gene	biological_pr	G:9606:PSMA5			KEGG-&-1&-	PSMA5	Homo sapier	Gene Symbol	PSMA5	FALSE	PSMA5
taxon:9606	178664	1902	proteasome	gene	biological_pr	G:9606:PSMB1			KEGG-&-1&-	PSMB1	Homo sapier	Gene Symbol	PSMB1	FALSE	PSMB1
taxon:9606	178665	1902	proteasome	gene	biological_pr	G:9606:PSMA7			KEGG-&-1&-	PSMA7	Homo sapier	Gene Symbol	PSMA7	FALSE	PSMA7
taxon:9606	178669	1902	proteasome	gene	biological_pr	G:9606:PSMA3			KEGG-&-1&-	PSMA3	Homo sapier	Gene Symbol	PSMA3	FALSE	PSMA3
taxon:9606	182778	1902	apurinic/apy	gene	biological_pr	G:9606:APEX1			KEGG-&-1&-	APEX1	Homo sapier	Gene Symbol	APEX1	FALSE	APEX1
taxon:9606	178682	1902	presenilin 2	gene	biological_pr	G:9606:PSEN2			KEGG-&-1&-	PSEN2	Homo sapier	Gene Symbol	PSEN2	FALSE	PSEN2
taxon:9606	178683	1902	presenilin 1	gene	biological_pr	G:9606:PSEN1			KEGG-&-1&-	PSEN1	Homo sapier	Gene Symbol	PSEN1	FALSE	PSEN1
taxon:9606	182777	1902	baculoviral IA	gene	biological_pr	G:9606:BIRC2			KEGG-&-1&-	BIRC2	Homo sapier	Gene Symbol	BIRC2	FALSE	BIRC2
taxon:9606	170494	1902	tripartite mo	gene	biological_pr	G:9606:TRIM68				TRIM68	Homo sapier	Gene Symbol	TRIM68	FALSE	TRIM68
taxon:9606	182782	1902	APC, WNT si	gene	biological_pr	G:9606:APC			KEGG-&-1&-	APC	Homo sapier	Gene Symbol	APC	FALSE	APC
taxon:9606	178686	1902	prosaposin	gene	biological_pr	G:9606:PSAP			KEGG-&-1&-	PSAP	Homo sapier	Gene Symbol	PSAP	FALSE	PSAP
taxon:9606	178687	1902	proteinase 3	gene	biological_pr	G:9606:PRTN3				PRTN3	Homo sapier	Gene Symbol	PRTN3	FALSE	PRTN3
taxon:9606	170495	1902	HEAT repeat	gene	biological_pr	G:9606:HEATR1			KEGG-&-1&-	HEATR1	Homo sapier	Gene Symbol	HEATR1	FALSE	HEATR1
taxon:9606	182780	1902	autoimmune	gene	biological_pr	G:9606:AIRE			KEGG-&-1&-	AIRE	Homo sapier	Gene Symbol	AIRE	FALSE	AIRE
taxon:9606	174589	1902	ubiquitin spe	gene	biological_pr	G:9606:USP19				USP19	Homo sapier	Gene Symbol	USP19	FALSE	USP19
taxon:9606	158210	1902	YY2 transcrip	gene	biological_pr	G:9606:YY2				YY2	Homo sapier	Gene Symbol	YY2	FALSE	YY2
taxon:9606	170498	1902	akirin 2	gene	biological_pr	G:9606:AKIRIN2				AKIRIN2	Homo sapier	Gene Symbol	AKIRIN2	FALSE	AKIRIN2
taxon:9606	174593	1902	AT-rich inter	gene	biological_pr	G:9606:ARID5A				ARID5A	Homo sapier	Gene Symbol	ARID5A	FALSE	ARID5A
taxon:9606	178689	1902	HtrA serine p	gene	biological_pr	G:9606:HTRA1				HTRA1	Homo sapier	Gene Symbol	HTRA1	FALSE	HTRA1
taxon:9606	182795	1902	annexin A7	gene	biological_pr	G:9606:ANXA7				ANXA7	Homo sapier	Gene Symbol	ANXA7	FALSE	ANXA7
taxon:9606	174603	1902	BCAS2, pre-m	gene	biological_pr	G:9606:BCAS2			KEGG-&-1&-	BCAS2	Homo sapier	Gene Symbol	BCAS2	FALSE	BCAS2
taxon:9606	178697	1902	protease, ser	gene	biological_pr	G:9606:PRSS2			KEGG-&-1&-	PRSS2	Homo sapier	Gene Symbol	PRSS2	FALSE	PRSS2
taxon:9606	182799	1902	annexin A3	gene	biological_pr	G:9606:ANXA3				ANXA3	Homo sapier	Gene Symbol	ANXA3	FALSE	ANXA3
taxon:9606	170511	1902	anoctamin 1	gene	biological_pr	G:9606:ANO1				ANO1	Homo sapier	Gene Symbol	ANO1	FALSE	ANO1
taxon:9606	182803	1902	annexin A2	gene	biological_pr	G:9606:ANXA2				ANXA2	Homo sapier	Gene Symbol	ANXA2	FALSE	ANXA2
taxon:9606	174609	1902	sigma non-o	gene	biological_pr	G:9606:SIGMAR1				SIGMAR1	Homo sapier	Gene Symbol	SIGMAR1	FALSE	SIGMAR1
taxon:9606	182806	1902	solute carrie	gene	biological_pr	G:9606:SLC25A5			KEGG-&-1&-	SLC25A5	Homo sapier	Gene Symbol	SLC25A5	FALSE	SLC25A5
taxon:9606	174615	1902	STIP1 homol	gene	biological_pr	G:9606:STUB1			KEGG-&-1&-	STUB1	Homo sapier	Gene Symbol	STUB1	FALSE	STUB1
taxon:9606	182807	1902	solute carrie	gene	biological_pr	G:9606:SLC25A4			KEGG-&-1&-	SLC25A4	Homo sapier	Gene Symbol	SLC25A4	FALSE	SLC25A4
taxon:9606	174612	1902	ubiquitinatio	gene	biological_pr	G:9606:UBE4B			KEGG-&-1&-	UBE4B	Homo sapier	Gene Symbol	UBE4B	FALSE	UBE4B
taxon:9606	182804	1902	annexin A1	gene	biological_pr	G:9606:ANXA1				ANXA1	Homo sapier	Gene Symbol	ANXA1	FALSE	ANXA1

taxon:9606	170517	1902	WD repeat d	gene	molecular_fu	G:9606:WDR70				WDR70	Homo sapier Gene Symbol	WDR70	FALSE	WDR70
taxon:9606	182811	1902	ankyrin 2	gene	biological_pr	G:9606:ANK2			KEGG-&-1&-	ANK2	Homo sapier Gene Symbol	ANK2	FALSE	ANK2
taxon:9606	166427	1902	dedicator of	gene	biological_pr	G:9606:DOCK7				DOCK7	Homo sapier Gene Symbol	DOCK7	FALSE	DOCK7
taxon:9606	162328	1902	mitogen-acti	gene	biological_pr	G:9606:MAPK15			KEGG-&-1&-	MAPK15	Homo sapier Gene Symbol	MAPK15	FALSE	MAPK15
taxon:9606	182815	1902	angiogenin	gene	biological_pr	G:9606:ANG				ANG	Homo sapier Gene Symbol	ANG	FALSE	ANG
taxon:9606	166428	1902	stonin 2	gene	biological_pr	G:9606:STON2				STON2	Homo sapier Gene Symbol	STON2	FALSE	STON2
taxon:9606	174627	1902	ATP binding	gene	biological_pr	G:9606:ABCC4			KEGG-&-1&-	ABCC4	Homo sapier Gene Symbol	ABCC4	FALSE	ABCC4
taxon:9606	178726	1902	eukaryotic tr	gene	biological_pr	G:9606:EIF2AK2			KEGG-&-1&-	EIF2AK2	Homo sapier Gene Symbol	EIF2AK2	FALSE	EIF2AK2
taxon:9606	166438	1902	DnaJ heat sh	gene	biological_pr	G:9606:DNAJC14				DNAJC14	Homo sapier Gene Symbol	DNAJC14	FALSE	DNAJC14
taxon:9606	162343	1902	signal peptid	gene	biological_pr	G:9606:SCUBE3				SCUBE3	Homo sapier Gene Symbol	SCUBE3	FALSE	SCUBE3
taxon:9606	178724	1902	THAP domair	gene	biological_pr	G:9606:THAP12				THAP12	Homo sapier Gene Symbol	THAP12	FALSE	THAP12
taxon:9606	178731	1902	mitogen-acti	gene	biological_pr	G:9606:MAP2K2			KEGG-&-1&-	MAP2K2	Homo sapier Gene Symbol	MAP2K2	FALSE	MAP2K2
taxon:9606	170537	1902	ring finger pr	gene	biological_pr	G:9606:RNF31			KEGG-&-1&-	RNF31	Homo sapier Gene Symbol	RNF31	FALSE	RNF31
taxon:9606	178734	1902	mitogen-acti	gene	biological_pr	G:9606:MAPK10			KEGG-&-1&-	MAPK10	Homo sapier Gene Symbol	MAPK10	FALSE	MAPK10
taxon:9606	178735	1902	mitogen-acti	gene	biological_pr	G:9606:MAPK9			KEGG-&-1&-	MAPK9	Homo sapier Gene Symbol	MAPK9	FALSE	MAPK9
taxon:9606	182831	1902	autocrine m	gene	biological_pr	G:9606:AMFR			KEGG-&-1&-	AMFR	Homo sapier Gene Symbol	AMFR	FALSE	AMFR
taxon:9606	178738	1902	mitogen-acti	gene	biological_pr	G:9606:MAPK7			KEGG-&-1&-	MAPK7	Homo sapier Gene Symbol	MAPK7	FALSE	MAPK7
taxon:9606	170546	1902	WD repeat d	gene	biological_pr	G:9606:WIPI1			KEGG-&-1&-	WIPI1	Homo sapier Gene Symbol	WIPI1	FALSE	WIPI1
taxon:9606	178736	1902	mitogen-acti	gene	biological_pr	G:9606:MAPK11			KEGG-&-1&-	MAPK11	Homo sapier Gene Symbol	MAPK11	FALSE	MAPK11
taxon:9606	162352	1902	regulatory fa	gene	biological_pr	G:9606:RFX6			KEGG-&-1&-	RFX6	Homo sapier Gene Symbol	RFX6	FALSE	RFX6
taxon:9606	178737	1902	mitogen-acti	gene	biological_pr	G:9606:MAPK8			KEGG-&-1&-	MAPK8	Homo sapier Gene Symbol	MAPK8	FALSE	MAPK8
taxon:9606	178742	1902	mitogen-acti	gene	biological_pr	G:9606:MAPK1			KEGG-&-1&-	MAPK1	Homo sapier Gene Symbol	MAPK1	FALSE	MAPK1
taxon:9606	174646	1902	DDB1 and CU	gene	biological_pr	G:9606:DCAF7				DCAF7	Homo sapier Gene Symbol	DCAF7	FALSE	DCAF7
taxon:9606	166455	1902	myosin light	gene	biological_pr	G:9606:MYLK2			KEGG-&-1&-	MYLK2	Homo sapier Gene Symbol	MYLK2	FALSE	MYLK2
taxon:9606	174644	1902	mitochondria	gene	biological_pr	G:9606:MRPS31				MRPS31	Homo sapier Gene Symbol	MRPS31	FALSE	MRPS31
taxon:9606	178741	1902	mitogen-acti	gene	biological_pr	G:9606:MAPK3			KEGG-&-1&-	MAPK3	Homo sapier Gene Symbol	MAPK3	FALSE	MAPK3
taxon:9606	178744	1902	protein kinas	gene	biological_pr	G:9606:PRKG1			KEGG-&-1&-	PRKG1	Homo sapier Gene Symbol	PRKG1	FALSE	PRKG1
taxon:9606	174648	1902	heterogeneo	gene	biological_pr	G:9606:HNRNPR				HNRNPR	Homo sapier Gene Symbol	HNRNPR	FALSE	HNRNPR
taxon:9606	178745	1902	protein kinas	gene	biological_pr	G:9606:PRKDC			KEGG-&-1&-	PRKDC	Homo sapier Gene Symbol	PRKDC	FALSE	PRKDC
taxon:9606	178749	1902	protein kinas	gene	biological_pr	G:9606:PRKD1			KEGG-&-1&-	PRKD1	Homo sapier Gene Symbol	PRKD1	FALSE	PRKD1
taxon:9606	178755	1902	protein kinas	gene	biological_pr	G:9606:PRKCE			KEGG-&-1&-	PRKCE	Homo sapier Gene Symbol	PRKCE	FALSE	PRKCE
taxon:9606	170560	1902	pentatricope	gene	biological_pr	G:9606:PTCD3				PTCD3	Homo sapier Gene Symbol	PTCD3	FALSE	PTCD3
taxon:9606	178758	1902	protein kinas	gene	biological_pr	G:9606:PRKCA			KEGG-&-1&-	PRKCA	Homo sapier Gene Symbol	PRKCA	FALSE	PRKCA
taxon:9606	178756	1902	protein kinas	gene	biological_pr	G:9606:PRKCD			KEGG-&-1&-	PRKCD	Homo sapier Gene Symbol	PRKCD	FALSE	PRKCD
taxon:9606	178757	1902	protein kinas	gene	biological_pr	G:9606:PRKCB			KEGG-&-1&-	PRKCB	Homo sapier Gene Symbol	PRKCB	FALSE	PRKCB
taxon:9606	178760	1902	protein kinas	gene	biological_pr	G:9606:PRKAR2A			KEGG-&-1&-	PRKAR2A	Homo sapier Gene Symbol	PRKAR2A	FALSE	PRKAR2A
taxon:9606	174671	1902	DExD-box hel	gene	biological_pr	G:9606:DDX39A				DDX39A	Homo sapier Gene Symbol	DDX39A	FALSE	DDX39A
taxon:9606	162380	1902	forkhead box	gene	biological_pr	G:9606:FOXK1				FOXK1	Homo sapier Gene Symbol	FOXK1	FALSE	FOXK1
taxon:9606	182860	1902	aldolase, fru	gene	biological_pr	G:9606:ALDOA			KEGG-&-1&-	ALDOA	Homo sapier Gene Symbol	ALDOA	FALSE	ALDOA
taxon:9606	178771	1902	protein kinas	gene	biological_pr	G:9606:PRKAB1			KEGG-&-1&-	PRKAB1	Homo sapier Gene Symbol	PRKAB1	FALSE	PRKAB1
taxon:9606	174673	1902	TOP1 binding	gene	biological_pr	G:9606:TOPORS				TOPORS	Homo sapier Gene Symbol	TOPORS	FALSE	TOPORS
taxon:9606	178769	1902	protein kinas	gene	biological_pr	G:9606:PRKACA			KEGG-&-1&-	PRKACA	Homo sapier Gene Symbol	PRKACA	FALSE	PRKACA
taxon:9606	178775	1902	primase (DN	gene	biological_pr	G:9606:PRIM1			KEGG-&-1&-	PRIM1	Homo sapier Gene Symbol	PRIM1	FALSE	PRIM1
taxon:9606	178773	1902	protein kinas	gene	biological_pr	G:9606:PRKAA1			KEGG-&-1&-	PRKAA1	Homo sapier Gene Symbol	PRKAA1	FALSE	PRKAA1
taxon:9606	174683	1902	M-phase pho	gene	biological_pr	G:9606:MPHOSPH6			KEGG-&-1&-	MPHOSPH6	Homo sapier Gene Symbol	MPHOSPH6	FALSE	MPHOSPH6
taxon:9606	178779	1902	serglycin	gene	biological_pr	G:9606:SRGN				SRGN	Homo sapier Gene Symbol	SRGN	FALSE	SRGN
taxon:9606	166491	1902	histone clust	gene	biological_pr	G:9606:HIST1H2BK			KEGG-&-1&-	HIST1H2BK	Homo sapier Gene Symbol	HIST1H2BK	FALSE	HIST1H2BK
taxon:9606	182873	1902	albumin	gene	biological_pr	G:9606:ALB			KEGG-&-1&-	ALB	Homo sapier Gene Symbol	ALB	FALSE	ALB
taxon:9606	174686	1902	proteasome	gene	biological_pr	G:9606:PSME3			KEGG-&-1&-	PSME3	Homo sapier Gene Symbol	PSME3	FALSE	PSME3
taxon:9606	182878	1902	AKT serine/t	gene	biological_pr	G:9606:AKT1			KEGG-&-1&-	AKT1	Homo sapier Gene Symbol	AKT1	FALSE	AKT1
taxon:9606	170590	1902	B-cell CLL/ly	gene	biological_pr	G:9606:BCL11A				BCL11A	Homo sapier Gene Symbol	BCL11A	FALSE	BCL11A
taxon:9606	174687	1902	protein argin	gene	biological_pr	G:9606:PRMT3				PRMT3	Homo sapier Gene Symbol	PRMT3	FALSE	PRMT3
taxon:9606	170594	1902	tubulin alpha	gene	biological_pr	G:9606:TUBA8			KEGG-&-1&-	TUBA8	Homo sapier Gene Symbol	TUBA8	FALSE	TUBA8
taxon:9606	166497	1902	RALBP1 asso	gene	biological_pr	G:9606:REPS1				REPS1	Homo sapier Gene Symbol	REPS1	FALSE	REPS1
taxon:9606	182887	1902	aryl hydroca	gene	biological_pr	G:9606:AHR			KEGG-&-1&-	AHR	Homo sapier Gene Symbol	AHR	FALSE	AHR
taxon:9606	174692	1902	Aly/REF exp	gene	biological_pr	G:9606:ALYREF			KEGG-&-1&-	ALYREF	Homo sapier Gene Symbol	ALYREF	FALSE	ALYREF
taxon:9606	174693	1902	tyrosine kina	gene	biological_pr	G:9606:TNK2				TNK2	Homo sapier Gene Symbol	TNK2	FALSE	TNK2
taxon:9606	182890	1902	nuclear rece	gene	biological_pr	G:9606:NR0B1			KEGG-&-1&-	NR0B1	Homo sapier Gene Symbol	NR0B1	FALSE	NR0B1
taxon:9606	178794	1902	protein phos	gene	biological_pr	G:9606:PPP5C			KEGG-&-1&-	PPP5C	Homo sapier Gene Symbol	PPP5C	FALSE	PPP5C
taxon:9606	162410	1902	zinc finger ar	gene	biological_pr	G:9606:ZBTB9				ZBTB9	Homo sapier Gene Symbol	ZBTB9	FALSE	ZBTB9
taxon:9606	182889	1902	adenosylhom	gene	biological_pr	G:9606:AHCY			KEGG-&-1&-	AHCY	Homo sapier Gene Symbol	AHCY	FALSE	AHCY
taxon:9606	182894	1902	angiotensin I	gene	biological_pr	G:9606:AGTR1	AGTR1	TRUE	KEGG-&-1&-	AGTR1	Homo sapier Gene Symbol	AGTR1	FALSE	AGTR1
taxon:9606	170606	1902	inositol poly	gene	biological_pr	G:9606:INPP5K			KEGG-&-1&-	INPP5K	Homo sapier Gene Symbol	INPP5K	FALSE	INPP5K
taxon:9606	182895	1902	angiotensino	gene	biological_pr	G:9606:AGT			KEGG-&-1&-	AGT	Homo sapier Gene Symbol	AGT	FALSE	AGT
taxon:9606	178799	1902	protein phos	gene	biological_pr	G:9606:PPP4C			KEGG-&-1&-	PPP4C	Homo sapier Gene Symbol	PPP4C	FALSE	PPP4C
taxon:9606	178796	1902	protein phos	gene	biological_pr	G:9606:PPP3R1			KEGG-&-1&-	PPP3R1	Homo sapier Gene Symbol	PPP3R1	FALSE	PPP3R1
taxon:9606	178803	1902	protein phos	gene	biological_pr	G:9606:PPP2R5C			KEGG-&-1&-	PPP2R5C	Homo sapier Gene Symbol	PPP2R5C	FALSE	PPP2R5C
taxon:9606	162419	1902	O-acyl-ADP-r	gene	biological_pr	G:9606:OARD1				OARD1	Homo sapier Gene Symbol	OARD1	FALSE	OARD1
taxon:9606	178800	1902	protein phos	gene	biological_pr	G:9606:PPP3CA			KEGG-&-1&-	PPP3CA	Homo sapier Gene Symbol	PPP3CA	FALSE	PPP3CA
taxon:9606	182901	1902	aggrecan	gene	biological_pr	G:9606:ACAN				ACAN	Homo sapier Gene Symbol	ACAN	FALSE	ACAN
taxon:9606	178805	1902	protein phos	gene	biological_pr	G:9606:PPP2R5A			KEGG-&-1&-	PPP2R5A	Homo sapier Gene Symbol	PPP2R5A	FALSE	PPP2R5A
taxon:9606	170618	1902	AT-rich inter	gene	biological_pr	G:9606:ARID4B				ARID4B	Homo sapier Gene Symbol	ARID4B	FALSE	ARID4B
taxon:9606	170619	1902	WW domain	gene	biological_pr	G:9606:WWOX				WWOX	Homo sapier Gene Symbol	WWOX	FALSE	WWOX
taxon:9606	182910	1902	amino-termi	gene	biological_pr	G:9606:AES				AES	Homo sapier Gene Symbol	AES	FALSE	AES
taxon:9606	174722	1902	CCAAT/enha	gene	biological_pr	G:9606:CEBPZ				CEBPZ	Homo sapier Gene Symbol	CEBPZ	FALSE	CEBPZ
taxon:9606	178819	1902	peptidylprol	gene	biological_pr	G:9606:PPIB				PPIB	Homo sapier Gene Symbol	PPIB	FALSE	PPIB
taxon:9606	174720	1902	tripartite mo	gene	biological_pr	G:9606:TRIM28				TRIM28	Homo sapier Gene Symbol	TRIM28	FALSE	TRIM28
taxon:9606	178817	1902	peptidylprol	gene	biological_pr	G:9606:PPID			KEGG-&-1&-	PPID	Homo sapier Gene Symbol	PPID	FALSE	PPID
taxon:9606	182913	1902	adaptor relat	gene	biological_pr	G:9606:AP2B1			KEGG-&-1&-	AP2B1	Homo sapier Gene Symbol	AP2B1	FALSE	AP2B1
taxon:9606	182919	1902	G protein-co	gene	biological_pr	G:9606:GRK3			KEGG-&-1&-	GRK3	Homo sapier Gene Symbol	GRK3	FALSE	GRK3
taxon:9606	182916	1902	adaptor relat	gene	biological_pr	G:9606:AP2A1			KEGG-&-1&-	AP2A1	Homo sapier Gene Symbol	AP2A1	FALSE	AP2A1
taxon:9606	178820	1902	peptidylprol	gene	biological_pr	G:9606:PPIA			KEGG-&-1&-	PPIA	Homo sapier Gene Symbol	PPIA	FALSE	PPIA
taxon:9606	170629	1902	ubiquitin inte	gene	biological_pr	G:9606:UIMC1			KEGG-&-1&-	UIMC1	Homo sapier Gene Symbol	UIMC1	FALSE	UIMC1
taxon:9606	178821	1902	cathepsin A	gene	biological_pr	G:9606:CTSA			KEGG-&-1&-	CTSA	Homo sapier Gene Symbol	CTSA	FALSE	CTSA
taxon:9606	178826	1902	mediator cor	gene	biological_pr	G:9606:MED1			KEGG-&-1&-	MED1	Homo sapier Gene Symbol	MED1	FALSE	MED1
taxon:9606	178827	1902	peroxisome p	gene	biological_pr	G:9606:PPARG			KEGG-&-1&-	PPARG	Homo sapier Gene Symbol	PPARG	FALSE	PPARG
taxon:9606	182920	1902	G protein-co	gene	biological_pr	G:9606:GRK2			KEGG-&-1&-	GRK2	Homo sapier Gene Symbol	GRK2	FALSE	GRK2
taxon:9606	174729	1902	G3BP stress	gene	biological_pr	G:9606:G3BP1				G3BP1	Homo sapier Gene Symbol	G3BP1	FALSE	G3BP1
taxon:9606	178830	1902	pyrophospha	gene	biological_pr	G:9606:PPA1			KEGG-&-1&-	PPA1	Homo sapier Gene Symbol	PPA1	FALSE	PPA1
taxon:9606	166543	1902	tensin 4	gene	biological_pr	G:9606:TNS4				TNS4	Homo sapier Gene Symbol	TNS4	FALSE	TNS4
taxon:9606	178829	1902	peroxisome p	gene	biological_pr	G:9606:PPARA			KEGG-&-1&-	PPARA	Homo sapier Gene Symbol	PPARA	FALSE	PPARA
taxon:9606	170640	1902	nemo like kir	gene	biological_pr	G:9606:NLK			KEGG-&-1&-	NLK	Homo sapier Gene Symbol	NLK	FALSE	NLK
taxon:9606	178833	1902	POU class 5	gene	biological_pr	G:9606:POU5F1			KEGG-&-1&-	POU5F1	Homo sapier Gene Symbol	POU5F1	FALSE	POU5F1
taxon:9606	166551	1902	hematopoiet	gene	biological_pr	G:9606:HSH2D				HSH2D	Homo sapier Gene Symbol	HSH2D	FALSE	HSH2D
taxon:9606	174740	1902	B-cell recept	gene	biological_pr	G:9606:BCAP31			KEGG-&-1&-	BCAP31	Homo sapier Gene Symbol	BCAP31	FALSE	BCAP31
taxon:9606	182933	1902	poly(ADP-rib	gene	biological_pr	G:9606:PARP1	PARP1	TRUE	KEGG-&-1&-	PARP1	Homo sapier Gene Symbol	PARP1	FALSE	PARP1
taxon:9606	174741	1902	optineurin	gene	biological_pr	G:9606:OPTN			KEGG-&-1&-	OPTN	Homo sapier Gene Symbol	OPTN	FALSE	OPTN
taxon:9606	178842	1902	POU class 2	gene	biological_pr	G:9606:POU2F1				POU2F1	Homo sapier Gene Symbol	POU2F1	FALSE	POU2F1
taxon:9606	174745	1902	leucine rich p	gene	biological_pr	G:9606:LRPPRC				LRPPRC	Homo sapier Gene Symbol	LRPPRC	FALSE	LRPPRC
taxon:9606	178841	1902	POU class 2	gene	biological_pr	G:9606:POU2F2				POU2F2	Homo sapier Gene Symbol	POU2F2	FALSE	POU2F2
taxon:9606	170655	1902	negative reg	gene	biological_pr	G:9606:NUB1				NUB1	Homo sapier Gene Symbol	NUB1	FALSE	NUB1
taxon:9606	162460	1902	jumonji dom	gene	biological_pr	G:9606:JMJD1C			KEGG-&-1&-	JMJD1C	Homo sapier Gene Symbol	JMJD1C	FALSE	JMJD1C
taxon:9606	178844	1902	POU class 1	gene	biological_pr	G:9606:POU1F1				POU1F1	Homo sapier Gene Symbol	POU1F1	FALSE	POU1F1
taxon:9606	170653	1902	store-operat	gene	biological_pr	G:9606:SARAF				SARAF	Homo sapier Gene Symbol	SARAF	FALSE	SARAF
taxon:9606	178850	1902	RNA polyme	gene	biological_pr	G:9606:POLRMT				POLRMT	Homo sapier Gene Symbol	POLRMT	FALSE	POLRMT
taxon:9606	182944	1902	alcohol dehy	gene	biological_pr	G:9606:ADH5			KEGG-&-1&-	ADH5	Homo sapier Gene Symbol	ADH5	FALSE	ADH5
taxon:9606	162465	1902	heterogeneo	gene	biological_pr	G:9606:HNRNPA3			KEGG-&-1&-	HNRNPA3	Homo sapier Gene Symbol	HNRNPA3	FALSE	HNRNPA3
taxon:9606	174756	1902	homeodomai	gene	biological_pr	G:9606:HIPK3			KEGG-&-1&-	HIPK3	Homo sapier Gene Symbol	HIPK3	FALSE	HIPK3
taxon:9606	166569	1902	ALG10, alph	gene	biological_pr	G:9606:ALG10			KEGG-&-1&-	ALG10	Homo sapier Gene Symbol	ALG10	FALSE	ALG10
taxon:9606	182953	1902	aconitase 2	gene	biological_pr	G:9606:ACO2			KEGG-&-1&-	ACO2	Homo sapier Gene Symbol	ACO2	FALSE	ACO2
taxon:9606	170671	1902	peptidylprol	gene	biological_pr	G:9606:PPIL1			KEGG-&-1&-	PPIL1	Homo sapier Gene Symbol	PPIL1	FALSE	PPIL1
taxon:9606	170668	1902	mitochondria	gene	biological_pr	G:9606:MRPS23				MRPS23	Homo sapier Gene Symbol	MRPS23	FALSE	MRPS23
taxon:9606	174764	1902	CTD small ph	gene	biological_pr	G:9606:CTDSP2				CTDSP2	Homo sapier Gene Symbol	CTDSP2	FALSE	CTDSP2
taxon:9606	174765	1902	peptidylprol	gene	biological_pr	G:9606:PPIF			KEGG-&-1&-	PPIF	Homo sapier Gene Symbol	PPIF	FALSE	PPIF
taxon:9606	170674	1902	splicing fact	gene	biological_pr	G:9606:SF3B6			KEGG-&-1&-	SF3B6	Homo sapier Gene Symbol	SF3B6	FALSE	SF3B6
taxon:9606	182962	1902	acetyl-CoA a	gene	biological_pr	G:9606:ACAT1			KEGG-&-1&-	ACAT1	Homo sapier Gene Symbol	ACAT1	FALSE	ACAT1
taxon:9606	178870	1902	DNA polyme	gene	biological_pr	G:9606:POLA1			KEGG-&-1&-	POLA1	Homo sapier Gene Symbol	POLA1	FALSE	POLA1
taxon:9606	174775	1902	actin related	gene	biological_pr	G:9606:ARPC3			KEGG-&-1&-	ARPC3	Homo sapier Gene Symbol	ARPC3	FALSE	ARPC3
taxon:9606	174772	1902	ARP2 actin re	gene	biological_pr	G:9606:ACTR2			KEGG-&-1&-	ACTR2	Homo sapier Gene Symbol	ACTR2	FALSE	ACTR2
taxon:9606	178869	1902	DNA polyme	gene	biological_pr	G:9606:POLB			KEGG-&-1&-	POLB	Homo sapier Gene Symbol	POLB	FALSE	POLB
taxon:9606	182971	1902	active BCR-re	gene	biological_pr	G:9606:ABR				ABR	Homo sapier Gene Symbol	ABR	FALSE	ABR
taxon:9606	182969	1902	acetyl-CoA c	gene	biological_pr	G:9606:ACACA			KEGG-&-1&-	ACACA	Homo sapier Gene Symbol	ACACA	FALSE	ACACA
taxon:9606	174777	1902	actin related	gene	biological_pr	G:9606:ARPC5			KEGG-&-1&-	ARPC5	Homo sapier Gene Symbol	ARPC5	FALSE	ARPC5
taxon:9606	170686	1902	TATA-box bir	gene	biological_pr	G:9606:TAF9B			KEGG-&-1&-	TAF9B	Homo sapier Gene Symbol	TAF9B	FALSE	TAF9B
taxon:9606	182975	1902	ABL proto-on	gene	biological_pr	G:9606:ABL1			KEGG-&-1&-	ABL1	Homo sapier Gene Symbol	ABL1	FALSE	ABL1
taxon:9606	182973	1902	ABL proto-on	gene	biological_pr	G:9606:ABL2			KEGG-&-1&-	ABL2	Homo sapier Gene Symbol	ABL2	FALSE	ABL2
taxon:9606	174788	1902	DnaJ heat sh	gene	biological_pr	G:9606:DNAJB6				DNAJB6	Homo sapier Gene Symbol	DNAJB6	FALSE	DNAJB6
taxon:9606	174789	1902	RAN binding	gene	biological_pr	G:9606:RANBP9				RANBP9	Homo sapier Gene Symbol	RANBP9	FALSE	RANBP9
taxon:9606	166600	1902	apoptosis in	gene	biological_pr	G:9606:AIFM2			KEGG-&-1&-	AIFM2	Homo sapier Gene Symbol	AIFM2	FALSE	AIFM2
taxon:9606	182984	1902	alanyl-tRNA	gene	biological_pr	G:9606:AARS			KEGG-&-1&-	AARS	Homo sapier Gene Symbol	AARS	FALSE	AARS
taxon:9606	174793	1902	SH2 domain	gene	biological_pr	G:9606:SH2D3C				SH2D3C	Homo sapier Gene Symbol	SH2D3C	FALSE	SH2D3C
taxon:9606	178894	1902	promyelocyti	gene	biological_pr	G:9606:PML			KEGG-&-1&-	PML	Homo sapier Gene Symbol	PML	FALSE	PML
taxon:9606	174798	1902	poly(ADP-rib	gene	biological_pr	G:9606:PARP2			KEGG-&-1&-	PARP2	Homo sapier Gene Symbol	PARP2	FALSE	PARP2
taxon:9606	170701	1902	protein inhib	gene	biological_pr	G:9606:PIAS4			KEGG-&-1&-	PIAS4	Homo sapier Gene Symbol	PIAS4	FALSE	PIAS4
taxon:9606	174797	1902	poly(ADP-rib	gene	biological_pr	G:9606:PARP3			KEGG-&-1&-	PARP3	Homo sapier Gene Symbol	PARP3	FALSE	PARP3
taxon:9606	170707	1902	La ribonucle	gene	biological_pr	G:9606:LARP7				LARP7	Homo sapier Gene Symbol	LARP7	FALSE	LARP7
taxon:9606	182993	1902	alpha-2-mac	gene	biological_pr	G:9606:A2M			KEGG-&-1&-	A2M	Homo sapier Gene Symbol	A2M	FALSE	A2M
taxon:9606	178902	1902	plexin A2	gene	biological_pr	G:9606:PLXNA2			KEGG-&-1&-	PLXNA2	Homo sapier Gene Symbol	PLXNA2	FALSE	PLXNA2
taxon:9606	174805	1902	mediator cor	gene	biological_pr	G:9606:MED16			KEGG-&-1&-	MED16	Homo sapier Gene Symbol	MED16	FALSE	MED16
taxon:9606	178905	1902	phospholipid	gene	biological_pr	G:9606:PLSCR1				PLSCR1	Homo sapier Gene Symbol	PLSCR1	FALSE	PLSCR1
taxon:9606	170713	1902	histone deac	gene	biological_pr	G:9606:HDAC7			KEGG-&-1&-	HDAC7	Homo sapier Gene Symbol	HDAC7	FALSE	HDAC7
taxon:9606	162521	1902	proprotein c	gene	biological_pr	G:9606:PCSK9			KEGG-&-1&-	PCSK9	Homo sapier Gene Symbol	PCSK9	FALSE	PCSK9
taxon:9606	174814	1902	programmed	gene	biological_pr	G:9606:PDCD6				PDCD6	Homo sapier Gene Symbol	PDCD6	FALSE	PDCD6
taxon:9606	162526	1902	histone clust	gene	biological_pr	G:9606:HIST1H2BA			KEGG-&-1&-	HIST1H2BA	Homo sapier Gene Symbol	HIST1H2BA	FALSE	HIST1H2BA
taxon:9606	174815	1902	programmed	gene	biological_pr	G:9606:PDCD6IP			KEGG-&-1&-	PDCD6IP	Homo sapier Gene Symbol	PDCD6IP	FALSE	PDCD6IP
taxon:9606	170717	1902	5'-nucleotida	gene	biological_pr	G:9606:NT5DC3				NT5DC3	Homo sapier Gene Symbol	NT5DC3	FALSE	NT5DC3
taxon:9606	166630	1902	zinc finger pr	gene	biological_pr	G:9606:ZNF496				ZNF496	Homo sapier Gene Symbol	ZNF496	FALSE	ZNF496
taxon:9606	174823	1902	chaperonin c	gene	biological_pr	G:9606:CCT2				CCT2	Homo sapier Gene Symbol	CCT2	FALSE	CCT2
taxon:9606	170724	1902	sirtuin 7	gene	biological_pr	G:9606:SIRT7				SIRT7	Homo sapier Gene Symbol	SIRT7	FALSE	SIRT7
taxon:9606	178916	1902	polo like kina	gene	biological_pr	G:9606:PLK1			KEGG-&-1&-	PLK1	Homo sapier Gene Symbol	PLK1	FALSE	PLK1
taxon:9606	178922	1902	plasminogen	gene	biological_pr	G:9606:PLG			KEGG-&-1&-	PLG	Homo sapier Gene Symbol	PLG	FALSE	PLG
taxon:9606	174827	1902	SIVA1 apopt	gene	biological_pr	G:9606:SIVA1			KEGG-&-1&-	SIVA1	Homo sapier Gene Symbol	SIVA1	FALSE	SIVA1
taxon:9606	178923	1902	plectin	gene	biological_pr	G:9606:PLEC				PLEC	Homo sapier Gene Symbol	PLEC	FALSE	PLEC
taxon:9606	170731	1902	vesicle traffi	gene	biological_pr	G:9606:VTA1			KEGG-&-1&-	VTA1	Homo sapier Gene Symbol	VTA1	FALSE	VTA1
taxon:9606	174824	1902	chaperonin c	gene	biological_pr	G:9606:CCT4				CCT4	Homo sapier Gene Symbol	CCT4	FALSE	CCT4
taxon:9606	174825	1902	chaperonin c	gene	biological_pr	G:9606:CCT7				CCT7	Homo sapier Gene Symbol	CCT7	FALSE	CCT7
taxon:9606	178926	1902	phospholipas	gene	biological_pr	G:9606:PLCG2			KEGG-&-1&-	PLCG2	Homo sapier Gene Symbol	PLCG2	FALSE	PLCG2
taxon:9606	178927	1902	phospholipas	gene	biological_pr	G:9606:PLCG1			KEGG-&-1&-	PLCG1	Homo sapier Gene Symbol	PLCG1	FALSE	PLCG1
taxon:9606	174829	1902	SLU7 homolo	gene	biological_pr	G:9606:SLU7			KEGG-&-1&-	SLU7	Homo sapier Gene Symbol	SLU7	FALSE	SLU7
taxon:9606	170736	1902	JNK1/MAPK8	gene	biological_pr	G:9606:JKAMP				JKAMP	Homo sapier Gene Symbol	JKAMP	FALSE	JKAMP
taxon:9606	170742	1902	leucyl-tRNA	gene	biological_pr	G:9606:LARS			KEGG-&-1&-	LARS	Homo sapier Gene Symbol	LARS	FALSE	LARS
taxon:9606	170744	1902	denticleless	gene	biological_pr	G:9606:DTL				DTL	Homo sapier Gene Symbol	DTL	FALSE	DTL
taxon:9606	178937	1902	PLAG1 like zi	gene	biological_pr	G:9606:PLAGL1				PLAGL1	Homo sapier Gene Symbol	PLAGL1	FALSE	PLAGL1
taxon:9606	174846	1902	acidic nuclea	gene	biological_pr	G:9606:ANP32B				ANP32B	Homo sapier Gene Symbol	ANP32B	FALSE	ANP32B
taxon:9606	170751	1902	CWC15 splic	gene	biological_pr	G:9606:CWC15			KEGG-&-1&-	CWC15	Homo sapier Gene Symbol	CWC15	FALSE	CWC15
taxon:9606	174845	1902	late endosom	gene	biological_pr	G:9606:LAMTOR5			KEGG-&-1&-	LAMTOR5	Homo sapier Gene Symbol	LAMTOR5	FALSE	LAMTOR5
taxon:9606	174850	1902	ubiquitin D	gene	biological_pr	G:9606:UBD				UBD	Homo sapier Gene Symbol	UBD	FALSE	UBD
taxon:9606	170754	1902	negative elo	gene	biological_pr	G:9606:NELFCD				NELFCD	Homo sapier Gene Symbol	NELFCD	FALSE	NELFCD
taxon:9606	174848	1902	glutaredoxin	gene	biological_pr	G:9606:GLRX3				GLRX3	Homo sapier Gene Symbol	GLRX3	FALSE	GLRX3
taxon:9606	170753	1902	TP53 regulat	gene	biological_pr	G:9606:TRIAP1				TRIAP1	Homo sapier Gene Symbol	TRIAP1	FALSE	TRIAP1
taxon:9606	166657	1902	tubulin alpha	gene	biological_pr	G:9606:TUBA1C			KEGG-&-1&-	TUBA1C	Homo sapier Gene Symbol	TUBA1C	FALSE	TUBA1C
taxon:9606	174854	1902	autophagy re	gene	biological_pr	G:9606:ATG7			KEGG-&-1&-	ATG7	Homo sapier Gene Symbol	ATG7	FALSE	ATG7
taxon:9606	178951	1902	polycystin 1,	gene	biological_pr	G:9606:PKD1				PKD1	Homo sapier Gene Symbol	PKD1	FALSE	PKD1
taxon:9606	170756	1902	3-hydroxyacy	gene	biological_pr	G:9606:HACD3			KEGG-&-1&-	HACD3	Homo sapier Gene Symbol	HACD3	FALSE	HACD3
taxon:9606	174858	1902	importin 7	gene	biological_pr	G:9606:IPO7				IPO7	Homo sapier Gene Symbol	IPO7	FALSE	IPO7
taxon:9606	170760	1902	LIM domain	gene	biological_pr	G:9606:LIMA1				LIMA1	Homo sapier Gene Symbol	LIMA1	FALSE	LIMA1
taxon:9606	166670	1902	UDP-GlcNAc:	gene	biological_pr	G:9606:B3GNT9				B3GNT9	Homo sapier Gene Symbol	B3GNT9	FALSE	B3GNT9
taxon:9606	174861	1902	lysine acetyl	gene	biological_pr	G:9606:KAT5			KEGG-&-1&-	KAT5	Homo sapier Gene Symbol	KAT5	FALSE	KAT5
taxon:9606	178962	1902	phosphatidyl	gene	biological_pr	G:9606:PI4KA			KEGG-&-1&-	PI4KA	Homo sapier Gene Symbol	PI4KA	FALSE	PI4KA
taxon:9606	178963	1902	phosphoinos	gene	biological_pr	G:9606:PIK3R2			KEGG-&-1&-	PIK3R2	Homo sapier Gene Symbol	PIK3R2	FALSE	PIK3R2
taxon:9606	174864	1902	DEAD-box he	gene	biological_pr	G:9606:DDX17				DDX17	Homo sapier Gene Symbol	DDX17	FALSE	DDX17
taxon:9606	178960	1902	peptidylprol	gene	biological_pr	G:9606:PIN1			KEGG-&-1&-	PIN1	Homo sapier Gene Symbol	PIN1	FALSE	PIN1
taxon:9606	174870	1902	MYB binding	gene	biological_pr	G:9606:MYBBP1A				MYBBP1A	Homo sapier Gene Symbol	MYBBP1A	FALSE	MYBBP1A
taxon:9606	174871	1902	amyloid beta	gene	biological_pr	G:9606:APPBP2				APPBP2	Homo sapier Gene Symbol	APPBP2	FALSE	APPBP2
taxon:9606	178964	1902	phosphoinos	gene	biological_pr	G:9606:PIK3R1			KEGG-&-1&-	PIK3R1	Homo sapier Gene Symbol	PIK3R1	FALSE	PIK3R1
taxon:9606	170772	1902	nitric oxide s	gene	biological_pr	G:9606:NOSIP				NOSIP	Homo sapier Gene Symbol	NOSIP	FALSE	NOSIP
taxon:9606	166677	1902	chromobox 2	gene	biological_pr	G:9606:CBX2				CBX2	Homo sapier Gene Symbol	CBX2	FALSE	CBX2
taxon:9606	174878	1902	nuclear rece	gene	biological_pr	G:9606:NCOA2			KEGG-&-1&-	NCOA2	Homo sapier Gene Symbol	NCOA2	FALSE	NCOA2
taxon:9606	174879	1902	coactivator a	gene	biological_pr	G:9606:CARM1			KEGG-&-1&-	CARM1	Homo sapier Gene Symbol	CARM1	FALSE	CARM1
taxon:9606	170780	1902	thioredoxin d	gene	biological_pr	G:9606:TXNDC11				TXNDC11	Homo sapier Gene Symbol	TXNDC11	FALSE	TXNDC11
taxon:9606	174883	1902	vesicle amin	gene	biological_pr	G:9606:VAT1				VAT1	Homo sapier Gene Symbol	VAT1	FALSE	VAT1
taxon:9606	174884	1902	synaptotagm	gene	biological_pr	G:9606:SYNCRIP				SYNCRIP	Homo sapier Gene Symbol	SYNCRIP	FALSE	SYNCRIP
taxon:9606	170794	1902	chymotrypsir	gene	biological_pr	G:9606:CELA2B			KEGG-&-1&-	CELA2B	Homo sapier Gene Symbol	CELA2B	FALSE	CELA2B
taxon:9606	178986	1902	serpin family	gene	biological_pr	G:9606:SERPINB9			KEGG-&-1&-	SERPINB9	Homo sapier Gene Symbol	SERPINB9	FALSE	SERPINB9
taxon:9606	174889	1902	adenylate cy	gene	biological_pr	G:9606:CAP1				CAP1	Homo sapier Gene Symbol	CAP1	FALSE	CAP1
taxon:9606	174897	1902	solute carrier	gene	biological_pr	G:9606:SLC9A6			KEGG-&-1&-	SLC9A6	Homo sapier Gene Symbol	SLC9A6	FALSE	SLC9A6
taxon:9606	174902	1902	transcription	gene	biological_pr	G:9606:TADA3			KEGG-&-1&-	TADA3	Homo sapier Gene Symbol	TADA3	FALSE	TADA3
taxon:9606	170807	1902	pseudouridyl	gene	biological_pr	G:9606:PUS7				PUS7	Homo sapier Gene Symbol	PUS7	FALSE	PUS7
taxon:9606	170810	1902	exosome con	gene	biological_pr	G:9606:EXOSC4			KEGG-&-1&-	EXOSC4	Homo sapier Gene Symbol	EXOSC4	FALSE	EXOSC4
taxon:9606	179002	1902	nucleobindin	gene	biological_pr	G:9606:NUCB1				NUCB1	Homo sapier Gene Symbol	NUCB1	FALSE	NUCB1
taxon:9606	174911	1902	peptidylprol	gene	biological_pr	G:9606:PPIH			KEGG-&-1&-	PPIH	Homo sapier Gene Symbol	PPIH	FALSE	PPIH
taxon:9606	174909	1902	zinc finger H	gene	biological_pr	G:9606:ZNHIT1				ZNHIT1	Homo sapier Gene Symbol	ZNHIT1	FALSE	ZNHIT1
taxon:9606	179010	1902	neurotrophic	gene	biological_pr	G:9606:NTRK2			KEGG-&-1&-	NTRK2	Homo sapier Gene Symbol	NTRK2	FALSE	NTRK2
taxon:9606	179011	1902	neurotrophic	gene	biological_pr	G:9606:NTRK1			KEGG-&-1&-	NTRK1	Homo sapier Gene Symbol	NTRK1	FALSE	NTRK1
taxon:9606	174922	1902	TGF-beta act	gene	biological_pr	G:9606:TAB1			KEGG-&-1&-	TAB1	Homo sapier Gene Symbol	TAB1	FALSE	TAB1
taxon:9606	174920	1902	HCLS1 associ	gene	biological_pr	G:9606:HAX1				HAX1	Homo sapier Gene Symbol	HAX1	FALSE	HAX1
taxon:9606	179016	1902	neurotrophin	gene	biological_pr	G:9606:NTF4			KEGG-&-1&-	NTF4	Homo sapier Gene Symbol	NTF4	FALSE	NTF4
taxon:9606	179017	1902	neurotrophin	gene	biological_pr	G:9606:NTF3			KEGG-&-1&-	NTF3	Homo sapier Gene Symbol	NTF3	FALSE	NTF3
taxon:9606	179020	1902	Y-box binding	gene	biological_pr	G:9606:YBX1				YBX1	Homo sapier Gene Symbol	YBX1	FALSE	YBX1
taxon:9606	174924	1902	vav guanine	gene	biological_pr	G:9606:VAV3			KEGG-&-1&-	VAV3	Homo sapier Gene Symbol	VAV3	FALSE	VAV3
taxon:9606	179024	1902	nuclear respi	gene	biological_pr	G:9606:NRF1			KEGG-&-1&-	NRF1	Homo sapier Gene Symbol	NRF1	FALSE	NRF1
taxon:9606	179025	1902	nardilysin co	gene	biological_pr	G:9606:NRDC				NRDC	Homo sapier Gene Symbol	NRDC	FALSE	NRDC
taxon:9606	162646	1902	zinc finger ar	gene	biological_pr	G:9606:ZBTB38				ZBTB38	Homo sapier Gene Symbol	ZBTB38	FALSE	ZBTB38
taxon:9606	174938	1902	lysophosphol	gene	biological_pr	G:9606:LYPLA1			KEGG-&-1&-	LYPLA1	Homo sapier Gene Symbol	LYPLA1	FALSE	LYPLA1
taxon:9606	162651	1902	RPTOR indep	gene	biological_pr	G:9606:RICTOR			KEGG-&-1&-	RICTOR	Homo sapier Gene Symbol	RICTOR	FALSE	RICTOR
taxon:9606	174939	1902	RNA binding	gene	biological_pr	G:9606:RBM14				RBM14	Homo sapier Gene Symbol	RBM14	FALSE	RBM14
taxon:9606	174944	1902	ariadne RBR	gene	biological_pr	G:9606:ARIH2				ARIH2	Homo sapier Gene Symbol	ARIH2	FALSE	ARIH2
taxon:9606	174950	1902	protein argin	gene	biological_pr	G:9606:PRMT5			KEGG-&-1&-	PRMT5	Homo sapier Gene Symbol	PRMT5	FALSE	PRMT5
taxon:9606	170854	1902	F-box protei	gene	molecular_fu	G:9606:FBXO42				FBXO42	Homo sapier Gene Symbol	FBXO42	FALSE	FBXO42
taxon:9606	179048	1902	nucleophosm	gene	biological_pr	G:9606:NPM1				NPM1	Homo sapier Gene Symbol	NPM1	FALSE	NPM1
taxon:9606	170857	1902	anillin actin	gene	biological_pr	G:9606:ANLN				ANLN	Homo sapier Gene Symbol	ANLN	FALSE	ANLN
taxon:9606	179055	1902	purine nucle	gene	biological_pr	G:9606:PNP			KEGG-&-1&-	PNP	Homo sapier Gene Symbol	PNP	FALSE	PNP
taxon:9606	179059	1902	notch 4	gene	biological_pr	G:9606:NOTCH4			KEGG-&-1&-	NOTCH4	Homo sapier Gene Symbol	NOTCH4	FALSE	NOTCH4
taxon:9606	174967	1902	receptor for	gene	biological_pr	G:9606:RACK1			KEGG-&-1&-	RACK1	Homo sapier Gene Symbol	RACK1	FALSE	RACK1
taxon:9606	179063	1902	notch 1	gene	biological_pr	G:9606:NOTCH1			KEGG-&-1&-	NOTCH1	Homo sapier Gene Symbol	NOTCH1	FALSE	NOTCH1
taxon:9606	174965	1902	protein inhib	gene	biological_pr	G:9606:PIAS3			KEGG-&-1&-	PIAS3	Homo sapier Gene Symbol	PIAS3	FALSE	PIAS3
taxon:9606	170874	1902	TERF2 intera	gene	biological_pr	G:9606:TERF2IP				TERF2IP	Homo sapier Gene Symbol	TERF2IP	FALSE	TERF2IP
taxon:9606	179067	1902	nitric oxide s	gene	biological_pr	G:9606:NOS3	NOS3	TRUE	KEGG-&-1&-	NOS3	Homo sapier Gene Symbol	NOS3	FALSE	NOS3
taxon:9606	174968	1902	myosin light	gene	biological_pr	G:9606:MYL9			KEGG-&-1&-	MYL9	Homo sapier Gene Symbol	MYL9	FALSE	MYL9
taxon:9606	166777	1902	ligand depen	gene	biological_pr	G:9606:LCOR				LCOR	Homo sapier Gene Symbol	LCOR	FALSE	LCOR
taxon:9606	174969	1902	N-myc down	gene	biological_pr	G:9606:NDRG1				NDRG1	Homo sapier Gene Symbol	NDRG1	FALSE	NDRG1
taxon:9606	179070	1902	non-POU dor	gene	biological_pr	G:9606:NONO				NONO	Homo sapier Gene Symbol	NONO	FALSE	NONO
taxon:9606	179074	1902	N-myristoylt	gene	biological_pr	G:9606:NMT1				NMT1	Homo sapier Gene Symbol	NMT1	FALSE	NMT1
taxon:9606	166785	1902	synoviolin 1	gene	biological_pr	G:9606:SYVN1			KEGG-&-1&-	SYVN1	Homo sapier Gene Symbol	SYVN1	FALSE	SYVN1
taxon:9606	174982	1902	tubulin beta	gene	biological_pr	G:9606:TUBB4B			KEGG-&-1&-	TUBB4B	Homo sapier Gene Symbol	TUBB4B	FALSE	TUBB4B
taxon:9606	179076	1902	NME/NM23	gene	biological_pr	G:9606:NME4			KEGG-&-1&-	NME4	Homo sapier Gene Symbol	NME4	FALSE	NME4
taxon:9606	166788	1902	DOT1 like his	gene	biological_pr	G:9606:DOT1L			KEGG-&-1&-	DOT1L	Homo sapier Gene Symbol	DOT1L	FALSE	DOT1L
taxon:9606	174987	1902	tubulin alpha	gene	biological_pr	G:9606:TUBA1B			KEGG-&-1&-	TUBA1B	Homo sapier Gene Symbol	TUBA1B	FALSE	TUBA1B
taxon:9606	170896	1902	mitochondria	gene	biological_pr	G:9606:MRPL39				MRPL39	Homo sapier Gene Symbol	MRPL39	FALSE	MRPL39
taxon:9606	179098	1902	nescient heli	gene	biological_pr	G:9606:NHLH2				NHLH2	Homo sapier Gene Symbol	NHLH2	FALSE	NHLH2
taxon:9606	179096	1902	NHS actin re	gene	biological_pr	G:9606:NHS				NHS	Homo sapier Gene Symbol	NHS	FALSE	NHS
taxon:9606	179097	1902	SNU13 hom	gene	biological_pr	G:9606:SNU13			KEGG-&-1&-	SNU13	Homo sapier Gene Symbol	SNU13	FALSE	SNU13
taxon:9606	175011	1902	TRK-fused ge	gene	biological_pr	G:9606:TFG			KEGG-&-1&-	TFG	Homo sapier Gene Symbol	TFG	FALSE	TFG
taxon:9606	179104	1902	nuclear trans	gene	biological_pr	G:9606:NFYB			KEGG-&-1&-	NFYB	Homo sapier Gene Symbol	NFYB	FALSE	NFYB
taxon:9606	179105	1902	nuclear trans	gene	biological_pr	G:9606:NFYA			KEGG-&-1&-	NFYA	Homo sapier Gene Symbol	NFYA	FALSE	NFYA
taxon:9606	179111	1902	NFKB inhibit	gene	biological_pr	G:9606:NFKBIB			KEGG-&-1&-	NFKBIB	Homo sapier Gene Symbol	NFKBIB	FALSE	NFKBIB
taxon:9606	179114	1902	nuclear facto	gene	biological_pr	G:9606:NFKB1			KEGG-&-1&-	NFKB1	Homo sapier Gene Symbol	NFKB1	FALSE	NFKB1
taxon:9606	179112	1902	NFKB inhibit	gene	biological_pr	G:9606:NFKBIA			KEGG-&-1&-	NFKBIA	Homo sapier Gene Symbol	NFKBIA	FALSE	NFKBIA
taxon:9606	179113	1902	nuclear facto	gene	biological_pr	G:9606:NFKB2			KEGG-&-1&-	NFKB2	Homo sapier Gene Symbol	NFKB2	FALSE	NFKB2
taxon:9606	175023	1902	histone deac	gene	biological_pr	G:9606:HDAC5			KEGG-&-1&-	HDAC5	Homo sapier Gene Symbol	HDAC5	FALSE	HDAC5
taxon:9606	179117	1902	nuclear facto	gene	biological_pr	G:9606:NFIC				NFIC	Homo sapier Gene Symbol	NFIC	FALSE	NFIC
taxon:9606	175027	1902	zinc finger ar	gene	biological_pr	G:9606:ZBTB33				ZBTB33	Homo sapier Gene Symbol	ZBTB33	FALSE	ZBTB33
taxon:9606	175024	1902	histone deac	gene	biological_pr	G:9606:HDAC6			KEGG-&-1&-	HDAC6	Homo sapier Gene Symbol	HDAC6	FALSE	HDAC6
taxon:9606	175025	1902	steroid rece	gene	biological_pr	G:9606:SRA1				SRA1	Homo sapier Gene Symbol	SRA1	FALSE	SRA1
taxon:9606	179126	1902	nuclear facto	gene	biological_pr	G:9606:NFATC1			KEGG-&-1&-	NFATC1	Homo sapier Gene Symbol	NFATC1	FALSE	NFATC1
taxon:9606	179127	1902	neurofibromi	gene	biological_pr	G:9606:NF2			KEGG-&-1&-	NF2	Homo sapier Gene Symbol	NF2	FALSE	NF2
taxon:9606	179125	1902	nuclear facto	gene	biological_pr	G:9606:NFATC2			KEGG-&-1&-	NFATC2	Homo sapier Gene Symbol	NFATC2	FALSE	NFATC2
taxon:9606	175034	1902	nuclear rece	gene	biological_pr	G:9606:NR2E3				NR2E3	Homo sapier Gene Symbol	NR2E3	FALSE	NR2E3
taxon:9606	175035	1902	mediator cor	gene	biological_pr	G:9606:MED6				MED6	Homo sapier Gene Symbol	MED6	FALSE	MED6
taxon:9606	175037	1902	SCO2, cytoch	gene	biological_pr	G:9606:SCO2			KEGG-&-1&-	SCO2	Homo sapier Gene Symbol	SCO2	FALSE	SCO2
taxon:9606	179136	1902	neogenin 1	gene	biological_pr	G:9606:NEO1			KEGG-&-1&-	NEO1	Homo sapier Gene Symbol	NEO1	FALSE	NEO1
taxon:9606	175045	1902	cyclin D bind	gene	biological_pr	G:9606:DMTF1				DMTF1	Homo sapier Gene Symbol	DMTF1	FALSE	DMTF1
taxon:9606	166853	1902	inhibitor of g	gene	biological_pr	G:9606:ING5				ING5	Homo sapier Gene Symbol	ING5	FALSE	ING5
taxon:9606	179141	1902	neurofilame	gene	biological_pr	G:9606:NEFL			KEGG-&-1&-	NEFL	Homo sapier Gene Symbol	NEFL	FALSE	NEFL
taxon:9606	179146	1902	neural precu	gene	biological_pr	G:9606:NEDD8				NEDD8	Homo sapier Gene Symbol	NEDD8	FALSE	NEDD8
taxon:9606	166858	1902	nucleoside-tr	gene	biological_pr	G:9606:NTPCR			KEGG-&-1&-	NTPCR	Homo sapier Gene Symbol	NTPCR	FALSE	NTPCR
taxon:9606	179147	1902	ribosomal pr	gene	biological_pr	G:9606:RPL10A			KEGG-&-1&-	RPL10A	Homo sapier Gene Symbol	RPL10A	FALSE	RPL10A
taxon:9606	179150	1902	development	gene	biological_pr	G:9606:DRG1				DRG1	Homo sapier Gene Symbol	DRG1	FALSE	DRG1
taxon:9606	175052	1902	ring-box 1	gene	biological_pr	G:9606:RBX1			KEGG-&-1&-	RBX1	Homo sapier Gene Symbol	RBX1	FALSE	RBX1
taxon:9606	179149	1902	neural precu	gene	biological_pr	G:9606:NEDD4			KEGG-&-1&-	NEDD4	Homo sapier Gene Symbol	NEDD4	FALSE	NEDD4
taxon:9606	175057	1902	nuclear rece	gene	biological_pr	G:9606:NR1H4			KEGG-&-1&-	NR1H4	Homo sapier Gene Symbol	NR1H4	FALSE	NR1H4
taxon:9606	162769	1902	ubiquitin spe	gene	biological_pr	G:9606:USP12				USP12	Homo sapier Gene Symbol	USP12	FALSE	USP12
taxon:9606	179159	1902	NADH:ubiqui	gene	biological_pr	G:9606:NDUFS3			KEGG-&-1&-	NDUFS3	Homo sapier Gene Symbol	NDUFS3	FALSE	NDUFS3
taxon:9606	175060	1902	mediator cor	gene	biological_pr	G:9606:MED12			KEGG-&-1&-	MED12	Homo sapier Gene Symbol	MED12	FALSE	MED12
taxon:9606	175061	1902	thyroid horm	gene	biological_pr	G:9606:THRAP3				THRAP3	Homo sapier Gene Symbol	THRAP3	FALSE	THRAP3
taxon:9606	175067	1902	major vault	gene	biological_pr	G:9606:MVP				MVP	Homo sapier Gene Symbol	MVP	FALSE	MVP
taxon:9606	179161	1902	NADH:ubiqui	gene	biological_pr	G:9606:NDUFS1			KEGG-&-1&-	NDUFS1	Homo sapier Gene Symbol	NDUFS1	FALSE	NDUFS1
taxon:9606	162793	1902	homeodomai	gene	biological_pr	G:9606:HIPK1			KEGG-&-1&-	HIPK1	Homo sapier Gene Symbol	HIPK1	FALSE	HIPK1
taxon:9606	166894	1902	mediator cor	gene	biological_pr	G:9606:MED10				MED10	Homo sapier Gene Symbol	MED10	FALSE	MED10
taxon:9606	170992	1902	fission, mito	gene	biological_pr	G:9606:FIS1			KEGG-&-1&-	FIS1	Homo sapier Gene Symbol	FIS1	FALSE	FIS1
taxon:9606	171002	1902	exosome con	gene	biological_pr	G:9606:EXOSC1			KEGG-&-1&-	EXOSC1	Homo sapier Gene Symbol	EXOSC1	FALSE	EXOSC1
taxon:9606	175099	1902	PAN2 poly(A)	gene	biological_pr	G:9606:PAN2			KEGG-&-1&-	PAN2	Homo sapier Gene Symbol	PAN2	FALSE	PAN2
taxon:9606	179202	1902	msh homeob	gene	biological_pr	G:9606:MSX2			KEGG-&-1&-	MSX2	Homo sapier Gene Symbol	MSX2	FALSE	MSX2
taxon:9606	179203	1902	msh homeob	gene	biological_pr	G:9606:MSX1			KEGG-&-1&-	MSX1	Homo sapier Gene Symbol	MSX1	FALSE	MSX1
taxon:9606	175104	1902	SEC16 homolo	gene	biological_pr	G:9606:SEC16A				SEC16A	Homo sapier Gene Symbol	SEC16A	FALSE	SEC16A
taxon:9606	179201	1902	metallothion	gene	biological_pr	G:9606:MT1A			KEGG-&-1&-	MT1A	Homo sapier Gene Symbol	MT1A	FALSE	MT1A
taxon:9606	175105	1902	non-SMC con	gene	biological_pr	G:9606:NCAPD2				NCAPD2	Homo sapier Gene Symbol	NCAPD2	FALSE	NCAPD2
taxon:9606	175109	1902	SPT7-like STA	gene	biological_pr	G:9606:SUPT7L				SUPT7L	Homo sapier Gene Symbol	SUPT7L	FALSE	SUPT7L
taxon:9606	175114	1902	G3BP stress	gene	biological_pr	G:9606:G3BP2				G3BP2	Homo sapier Gene Symbol	G3BP2	FALSE	G3BP2
taxon:9606	179209	1902	moesin	gene	biological_pr	G:9606:MSN			KEGG-&-1&-	MSN	Homo sapier Gene Symbol	MSN	FALSE	MSN
taxon:9606	179215	1902	protein phos	gene	biological_pr	G:9606:PPP2R2B			KEGG-&-1&-	PPP2R2B	Homo sapier Gene Symbol	PPP2R2B	FALSE	PPP2R2B
taxon:9606	179218	1902	protein phos	gene	biological_pr	G:9606:PPP2R1A			KEGG-&-1&-	PPP2R1A	Homo sapier Gene Symbol	PPP2R1A	FALSE	PPP2R1A
taxon:9606	179216	1902	protein phos	gene	biological_pr	G:9606:PPP2R2A			KEGG-&-1&-	PPP2R2A	Homo sapier Gene Symbol	PPP2R2A	FALSE	PPP2R2A
taxon:9606	162832	1902	tubulin beta	gene	biological_pr	G:9606:TUBB			KEGG-&-1&-	TUBB	Homo sapier Gene Symbol	TUBB	FALSE	TUBB
taxon:9606	175120	1902	mannose rec	gene	biological_pr	G:9606:MRC2			KEGG-&-1&-	MRC2	Homo sapier Gene Symbol	MRC2	FALSE	MRC2
taxon:9606	179217	1902	protein phos	gene	biological_pr	G:9606:PPP2R1B			KEGG-&-1&-	PPP2R1B	Homo sapier Gene Symbol	PPP2R1B	FALSE	PPP2R1B
taxon:9606	175124	1902	ubiquitin ass	gene	biological_pr	G:9606:UBAP2L				UBAP2L	Homo sapier Gene Symbol	UBAP2L	FALSE	UBAP2L
taxon:9606	179221	1902	protein phos	gene	biological_pr	G:9606:PPP2CA			KEGG-&-1&-	PPP2CA	Homo sapier Gene Symbol	PPP2CA	FALSE	PPP2CA
taxon:9606	175130	1902	NUAK family	gene	biological_pr	G:9606:NUAK1				NUAK1	Homo sapier Gene Symbol	NUAK1	FALSE	NUAK1
taxon:9606	179231	1902	protein phos	gene	biological_pr	G:9606:PPP1CC			KEGG-&-1&-	PPP1CC	Homo sapier Gene Symbol	PPP1CC	FALSE	PPP1CC
taxon:9606	162845	1902	cardiomyopa	gene	cellular_com	G:9606:CMYA5				CMYA5	Homo sapier Gene Symbol	CMYA5	FALSE	CMYA5
taxon:9606	171037	1902	forkhead box	gene	biological_pr	G:9606:FOXP3			KEGG-&-1&-	FOXP3	Homo sapier Gene Symbol	FOXP3	FALSE	FOXP3
taxon:9606	175138	1902	TBC1 domair	gene	biological_pr	G:9606:TBC1D4			KEGG-&-1&-	TBC1D4	Homo sapier Gene Symbol	TBC1D4	FALSE	TBC1D4
taxon:9606	179233	1902	protein phos	gene	biological_pr	G:9606:PPP1CA			KEGG-&-1&-	PPP1CA	Homo sapier Gene Symbol	PPP1CA	FALSE	PPP1CA
taxon:9606	166950	1902	lysine acetyl	gene	biological_pr	G:9606:KAT8				KAT8	Homo sapier Gene Symbol	KAT8	FALSE	KAT8
taxon:9606	171047	1902	SPARC/osteo	gene	biological_pr	G:9606:SPOCK3				SPOCK3	Homo sapier Gene Symbol	SPOCK3	FALSE	SPOCK3
taxon:9606	166953	1902	family with s	gene	biological_pr	G:9606:FAM175A			KEGG-&-1&-	FAM175A	Homo sapier Gene Symbol	FAM175A	FALSE	FAM175A
taxon:9606	171054	1902	F11 receptor	gene	biological_pr	G:9606:F11R			KEGG-&-1&-	F11R	Homo sapier Gene Symbol	F11R	FALSE	F11R
taxon:9606	175155	1902	mediator cor	gene	biological_pr	G:9606:MED24			KEGG-&-1&-	MED24	Homo sapier Gene Symbol	MED24	FALSE	MED24
taxon:9606	166960	1902	ubiquitin spe	gene	biological_pr	G:9606:USP42				USP42	Homo sapier Gene Symbol	USP42	FALSE	USP42
taxon:9606	175158	1902	centrosomal	gene	cellular_com	G:9606:CEP170				CEP170	Homo sapier Gene Symbol	CEP170	FALSE	CEP170
taxon:9606	175156	1902	proteasome	gene	biological_pr	G:9606:PSMD6			KEGG-&-1&-	PSMD6	Homo sapier Gene Symbol	PSMD6	FALSE	PSMD6
taxon:9606	166970	1902	PML-RARA re	gene	biological_pr	G:9606:PRAM1				PRAM1	Homo sapier Gene Symbol	PRAM1	FALSE	PRAM1
taxon:9606	179256	1902	mutS homolo	gene	biological_pr	G:9606:MSH2			KEGG-&-1&-	MSH2	Homo sapier Gene Symbol	MSH2	FALSE	MSH2
taxon:9606	158776	1902	SHC adaptor	gene	biological_pr	G:9606:SHC4			KEGG-&-1&-	SHC4	Homo sapier Gene Symbol	SHC4	FALSE	SHC4
taxon:9606	171070	1902	COP9 signalo	gene	biological_pr	G:9606:COPS7A				COPS7A	Homo sapier Gene Symbol	COPS7A	FALSE	COPS7A
taxon:9606	162879	1902	centromere	gene	biological_pr	G:9606:CENPX			KEGG-&-1&-	CENPX	Homo sapier Gene Symbol	CENPX	FALSE	CENPX
taxon:9606	175185	1902	Rho guanine	gene	biological_pr	G:9606:ARHGEF17				ARHGEF17	Homo sapier Gene Symbol	ARHGEF17	FALSE	ARHGEF17
taxon:9606	175191	1902	RB1 inducibl	gene	biological_pr	G:9606:RB1CC1			KEGG-&-1&-	RB1CC1	Homo sapier Gene Symbol	RB1CC1	FALSE	RB1CC1
taxon:9606	142420	1902	notch homolo	gene		G:9606:LOC100996763				LOC10099676	Homo sapier Gene Symbol	LOC10099676	FALSE	LOC100996763
taxon:9606	175192	1902	cullin 7	gene	biological_pr	G:9606:CUL7			KEGG-&-1&-	CUL7	Homo sapier Gene Symbol	CUL7	FALSE	CUL7
taxon:9606	175202	1902	ring finger pr	gene	biological_pr	G:9606:RNF40				RNF40	Homo sapier Gene Symbol	RNF40	FALSE	RNF40
taxon:9606	175203	1902	KIAA0087 lnc	gene		G:9606:KIAA0087				KIAA0087	Homo sapier Gene Symbol	KIAA0087	FALSE	KIAA0087
taxon:9606	142439	1902	notch homolo	gene		G:9606:LOC100996717				LOC10099671	Homo sapier Gene Symbol	LOC10099671	FALSE	LOC100996717
taxon:9606	175210	1902	histone deac	gene	biological_pr	G:9606:HDAC4			KEGG-&-1&-	HDAC4	Homo sapier Gene Symbol	HDAC4	FALSE	HDAC4
taxon:9606	179311	1902	Mov10 RISC	gene	biological_pr	G:9606:MOV10				MOV10	Homo sapier Gene Symbol	MOV10	FALSE	MOV10
taxon:9606	167023	1902	mitochondria	gene	biological_pr	G:9606:MRPL43				MRPL43	Homo sapier Gene Symbol	MRPL43	FALSE	MRPL43
taxon:9606	171120	1902	ribonucleotid	gene	biological_pr	G:9606:RRM2B			KEGG-&-1&-	RRM2B	Homo sapier Gene Symbol	RRM2B	FALSE	RRM2B
taxon:9606	179318	1902	myeloid cell	gene	biological_pr	G:9606:MNDA				MNDA	Homo sapier Gene Symbol	MNDA	FALSE	MNDA
taxon:9606	179319	1902	MNAT1, CDK	gene	biological_pr	G:9606:MNAT1			KEGG-&-1&-	MNAT1	Homo sapier Gene Symbol	MNAT1	FALSE	MNAT1
taxon:9606	179323	1902	matrix metal	gene	biological_pr	G:9606:MMP17			KEGG-&-1&-	MMP17	Homo sapier Gene Symbol	MMP17	FALSE	MMP17
taxon:9606	179326	1902	matrix metal	gene	biological_pr	G:9606:MMP14			KEGG-&-1&-	MMP14	Homo sapier Gene Symbol	MMP14	FALSE	MMP14
taxon:9606	175230	1902	G protein-co	gene	biological_pr	G:9606:GPRASP1				GPRASP1	Homo sapier Gene Symbol	GPRASP1	FALSE	GPRASP1
taxon:9606	179327	1902	matrix metal	gene	biological_pr	G:9606:MMP13	MMP13	TRUE	KEGG-&-1&-	MMP13	Homo sapier Gene Symbol	MMP13	FALSE	MMP13
taxon:9606	175228	1902	SET domain	gene	biological_pr	G:9606:SETD1A			KEGG-&-1&-	SETD1A	Homo sapier Gene Symbol	SETD1A	FALSE	SETD1A
taxon:9606	171138	1902	deoxynucleot	gene	biological_pr	G:9606:DNTTIP2				DNTTIP2	Homo sapier Gene Symbol	DNTTIP2	FALSE	DNTTIP2
taxon:9606	175234	1902	squamous ce	gene	biological_pr	G:9606:SART3				SART3	Homo sapier Gene Symbol	SART3	FALSE	SART3
taxon:9606	179330	1902	matrix metal	gene	biological_pr	G:9606:MMP10				MMP10	Homo sapier Gene Symbol	MMP10	FALSE	MMP10
taxon:9606	179331	1902	matrix metal	gene	biological_pr	G:9606:MMP9	MMP9	TRUE	KEGG-&-1&-	MMP9	Homo sapier Gene Symbol	MMP9	FALSE	MMP9
taxon:9606	175233	1902	histone deac	gene	biological_pr	G:9606:HDAC9			KEGG-&-1&-	HDAC9	Homo sapier Gene Symbol	HDAC9	FALSE	HDAC9
taxon:9606	179334	1902	matrix metal	gene	biological_pr	G:9606:MMP3	MMP3	TRUE	KEGG-&-1&-	MMP3	Homo sapier Gene Symbol	MMP3	FALSE	MMP3
taxon:9606	175238	1902	KIAA0408	gene	molecular_fu	G:9606:KIAA0408				KIAA0408	Homo sapier Gene Symbol	KIAA0408	FALSE	KIAA0408
taxon:9606	179335	1902	matrix metal	gene	biological_pr	G:9606:MMP2	MMP2	TRUE	KEGG-&-1&-	MMP2	Homo sapier Gene Symbol	MMP2	FALSE	MMP2
taxon:9606	175237	1902	DDB1 and CU	gene	biological_pr	G:9606:DCAF1			KEGG-&-1&-	DCAF1	Homo sapier Gene Symbol	DCAF1	FALSE	DCAF1
taxon:9606	179333	1902	matrix metal	gene	biological_pr	G:9606:MMP7			KEGG-&-1&-	MMP7	Homo sapier Gene Symbol	MMP7	FALSE	MMP7
taxon:9606	171144	1902	CXXC finger	gene	biological_pr	G:9606:CXXC1				CXXC1	Homo sapier Gene Symbol	CXXC1	FALSE	CXXC1
taxon:9606	162953	1902	abnormal spi	gene	biological_pr	G:9606:ASPM				ASPM	Homo sapier Gene Symbol	ASPM	FALSE	ASPM
taxon:9606	175246	1902	serine palmi	gene	biological_pr	G:9606:SPTLC1			KEGG-&-1&-	SPTLC1	Homo sapier Gene Symbol	SPTLC1	FALSE	SPTLC1
taxon:9606	179343	1902	afadin, adhe	gene	biological_pr	G:9606:AFDN			KEGG-&-1&-	AFDN	Homo sapier Gene Symbol	AFDN	FALSE	AFDN
taxon:9606	179340	1902	nuclear rece	gene	biological_pr	G:9606:NR3C2			KEGG-&-1&-	NR3C2	Homo sapier Gene Symbol	NR3C2	FALSE	NR3C2
taxon:9606	175244	1902	solute carrier	gene	biological_pr	G:9606:SLC19A2			KEGG-&-1&-	SLC19A2	Homo sapier Gene Symbol	SLC19A2	FALSE	SLC19A2
taxon:9606	179341	1902	forkhead box	gene	biological_pr	G:9606:FOXO4			KEGG-&-1&-	FOXO4	Homo sapier Gene Symbol	FOXO4	FALSE	FOXO4
taxon:9606	162962	1902	heat shock p	gene	biological_pr	G:9606:HSPA12A				HSPA12A	Homo sapier Gene Symbol	HSPA12A	FALSE	HSPA12A
taxon:9606	179346	1902	MLLT1, supe	gene	biological_pr	G:9606:MLLT1			KEGG-&-1&-	MLLT1	Homo sapier Gene Symbol	MLLT1	FALSE	MLLT1
taxon:9606	179347	1902	lysine methyl	gene	biological_pr	G:9606:KMT2A			KEGG-&-1&-	KMT2A	Homo sapier Gene Symbol	KMT2A	FALSE	KMT2A
taxon:9606	179344	1902	MLLT3, supe	gene	biological_pr	G:9606:MLLT3			KEGG-&-1&-	MLLT3	Homo sapier Gene Symbol	MLLT3	FALSE	MLLT3
taxon:9606	175255	1902	HECT, UBA a	gene	biological_pr	G:9606:HUWE1			KEGG-&-1&-	HUWE1	Homo sapier Gene Symbol	HUWE1	FALSE	HUWE1
taxon:9606	171162	1902	EGF containi	gene	cellular_com	G:9606:EFEMP2				EFEMP2	Homo sapier Gene Symbol	EFEMP2	FALSE	EFEMP2
taxon:9606	167071	1902	ubiquitin spe	gene	biological_pr	G:9606:USP26				USP26	Homo sapier Gene Symbol	USP26	FALSE	USP26
taxon:9606	175264	1902	COX17, cytoc	gene	biological_pr	G:9606:COX17			KEGG-&-1&-	COX17	Homo sapier Gene Symbol	COX17	FALSE	COX17
taxon:9606	179361	1902	macrophage	gene	biological_pr	G:9606:MIF			KEGG-&-1&-	MIF	Homo sapier Gene Symbol	MIF	FALSE	MIF
taxon:9606	175265	1902	nuclear rece	gene	biological_pr	G:9606:NR1H3			KEGG-&-1&-	NR1H3	Homo sapier Gene Symbol	NR1H3	FALSE	NR1H3
taxon:9606	175271	1902	phenylalanyl-	gene	biological_pr	G:9606:FARSB			KEGG-&-1&-	FARSB	Homo sapier Gene Symbol	FARSB	FALSE	FARSB
taxon:9606	175269	1902	ATP binding	gene	biological_pr	G:9606:ABCB6			KEGG-&-1&-	ABCB6	Homo sapier Gene Symbol	ABCB6	FALSE	ABCB6
taxon:9606	167083	1902	glutamate ri	gene	biological_pr	G:9606:GRWD1				GRWD1	Homo sapier Gene Symbol	GRWD1	FALSE	GRWD1
taxon:9606	175272	1902	SUMO1 activ	gene	biological_pr	G:9606:SAE1			KEGG-&-1&-	SAE1	Homo sapier Gene Symbol	SAE1	FALSE	SAE1
taxon:9606	167087	1902	itchy E3 ubiq	gene	biological_pr	G:9606:ITCH			KEGG-&-1&-	ITCH	Homo sapier Gene Symbol	ITCH	FALSE	ITCH
taxon:9606	179372	1902	class II major	gene	biological_pr	G:9606:CIITA			KEGG-&-1&-	CIITA	Homo sapier Gene Symbol	CIITA	FALSE	CIITA
taxon:9606	154797	1902	histone clust	gene	biological_pr	G:9606:HIST2H2AA4			KEGG-&-1&-	HIST2H2AA4	Homo sapier Gene Symbol	HIST2H2AA4	FALSE	HIST2H2AA4
taxon:9606	167098	1902	fermitin fam	gene	biological_pr	G:9606:FERMT3			KEGG-&-1&-	FERMT3	Homo sapier Gene Symbol	FERMT3	FALSE	FERMT3
taxon:9606	171192	1902	DNA methylt	gene	biological_pr	G:9606:DNMT3L				DNMT3L	Homo sapier Gene Symbol	DNMT3L	FALSE	DNMT3L
taxon:9606	171196	1902	peptidyl argi	gene	biological_pr	G:9606:PADI1				PADI1	Homo sapier Gene Symbol	PADI1	FALSE	PADI1
taxon:9606	167100	1902	SH3 domain	gene	cellular_com	G:9606:SH3BGRL2				SH3BGRL2	Homo sapier Gene Symbol	SH3BGRL2	FALSE	SH3BGRL2
taxon:9606	179389	1902	microfibrillar	gene	biological_pr	G:9606:MFAP4				MFAP4	Homo sapier Gene Symbol	MFAP4	FALSE	MFAP4
taxon:9606	171201	1902	replication p	gene	biological_pr	G:9606:RPA4			KEGG-&-1&-	RPA4	Homo sapier Gene Symbol	RPA4	FALSE	RPA4
taxon:9606	179398	1902	meprin A sub	gene	biological_pr	G:9606:MEP1A			KEGG-&-1&-	MEP1A	Homo sapier Gene Symbol	MEP1A	FALSE	MEP1A
taxon:9606	167110	1902	poly(ADP-rib	gene	biological_pr	G:9606:PARP9				PARP9	Homo sapier Gene Symbol	PARP9	FALSE	PARP9
taxon:9606	179399	1902	mesenchyme	gene	biological_pr	G:9606:MEOX2				MEOX2	Homo sapier Gene Symbol	MEOX2	FALSE	MEOX2
taxon:9606	179397	1902	meprin A sub	gene	biological_pr	G:9606:MEP1B			KEGG-&-1&-	MEP1B	Homo sapier Gene Symbol	MEP1B	FALSE	MEP1B
taxon:9606	175304	1902	ER membrane	gene	biological_pr	G:9606:EMC2				EMC2	Homo sapier Gene Symbol	EMC2	FALSE	EMC2
taxon:9606	179401	1902	menin 1	gene	biological_pr	G:9606:MEN1			KEGG-&-1&-	MEN1	Homo sapier Gene Symbol	MEN1	FALSE	MEN1
taxon:9606	179406	1902	mitogen-acti	gene	biological_pr	G:9606:MAP3K1			KEGG-&-1&-	MAP3K1	Homo sapier Gene Symbol	MAP3K1	FALSE	MAP3K1
taxon:9606	175309	1902	nucleoporin	gene	biological_pr	G:9606:NUP93			KEGG-&-1&-	NUP93	Homo sapier Gene Symbol	NUP93	FALSE	NUP93
taxon:9606	175315	1902	lysine demet	gene	biological_pr	G:9606:KDM4A				KDM4A	Homo sapier Gene Symbol	KDM4A	FALSE	KDM4A
taxon:9606	175312	1902	clathrin inter	gene	biological_pr	G:9606:CLINT1				CLINT1	Homo sapier Gene Symbol	CLINT1	FALSE	CLINT1
taxon:9606	167127	1902	zinc finger M	gene	biological_pr	G:9606:ZMIZ2				ZMIZ2	Homo sapier Gene Symbol	ZMIZ2	FALSE	ZMIZ2
taxon:9606	179412	1902	myocyte enh	gene	biological_pr	G:9606:MEF2C			KEGG-&-1&-	MEF2C	Homo sapier Gene Symbol	MEF2C	FALSE	MEF2C
taxon:9606	179422	1902	MDM2 proto	gene	biological_pr	G:9606:MDM2			KEGG-&-1&-	MDM2	Homo sapier Gene Symbol	MDM2	FALSE	MDM2
taxon:9606	175326	1902	eukaryotic tr	gene	biological_pr	G:9606:EIF5B			KEGG-&-1&-	EIF5B	Homo sapier Gene Symbol	EIF5B	FALSE	EIF5B
taxon:9606	179421	1902	MDM4, p53 r	gene	biological_pr	G:9606:MDM4			KEGG-&-1&-	MDM4	Homo sapier Gene Symbol	MDM4	FALSE	MDM4
taxon:9606	175328	1902	scaffold atta	gene	biological_pr	G:9606:SAFB2				SAFB2	Homo sapier Gene Symbol	SAFB2	FALSE	SAFB2
taxon:9606	179424	1902	malate dehy	gene	biological_pr	G:9606:MDH2			KEGG-&-1&-	MDH2	Homo sapier Gene Symbol	MDH2	FALSE	MDH2
taxon:9606	179425	1902	malate dehy	gene	biological_pr	G:9606:MDH1			KEGG-&-1&-	MDH1	Homo sapier Gene Symbol	MDH1	FALSE	MDH1
taxon:9606	175335	1902	IQ motif con	gene	biological_pr	G:9606:IQCB1				IQCB1	Homo sapier Gene Symbol	IQCB1	FALSE	IQCB1
taxon:9606	175332	1902	centrosomal	gene	biological_pr	G:9606:CEP135				CEP135	Homo sapier Gene Symbol	CEP135	FALSE	CEP135
taxon:9606	154853	1902	bolA family r	gene	biological_pr	G:9606:BOLA2B				BOLA2B	Homo sapier Gene Symbol	BOLA2B	FALSE	BOLA2B
taxon:9606	175336	1902	mediator of	gene	biological_pr	G:9606:MDC1				MDC1	Homo sapier Gene Symbol	MDC1	FALSE	MDC1
taxon:9606	179443	1902	galectin 3	gene	biological_pr	G:9606:LGALS3				LGALS3	Homo sapier Gene Symbol	LGALS3	FALSE	LGALS3
taxon:9606	171251	1902	membrane a	gene	biological_pr	G:9606:MARCH2				Mar-02	Homo sapier Gene Symbol	Mar-02	FALSE	Mar-02
taxon:9606	179441	1902	galectin 4	gene	biological_pr	G:9606:LGALS4				LGALS4	Homo sapier Gene Symbol	LGALS4	FALSE	LGALS4
taxon:9606	175350	1902	inhibitor of n	gene	biological_pr	G:9606:IKBKE			KEGG-&-1&-	IKBKE	Homo sapier Gene Symbol	IKBKE	FALSE	IKBKE
taxon:9606	179445	1902	galectin 1	gene	biological_pr	G:9606:LGALS1				LGALS1	Homo sapier Gene Symbol	LGALS1	FALSE	LGALS1
taxon:9606	163064	1902	aprataxin an	gene	biological_pr	G:9606:APLF				APLF	Homo sapier Gene Symbol	APLF	FALSE	APLF
taxon:9606	179454	1902	lactate dehy	gene	biological_pr	G:9606:LDHB			KEGG-&-1&-	LDHB	Homo sapier Gene Symbol	LDHB	FALSE	LDHB
taxon:9606	171261	1902	shisa family	gene	biological_pr	G:9606:SHISA5			KEGG-&-1&-	SHISA5	Homo sapier Gene Symbol	SHISA5	FALSE	SHISA5
taxon:9606	179456	1902	lactate dehy	gene	biological_pr	G:9606:LDHA			KEGG-&-1&-	LDHA	Homo sapier Gene Symbol	LDHA	FALSE	LDHA
taxon:9606	179462	1902	LCK proto-on	gene	biological_pr	G:9606:LCK			KEGG-&-1&-	LCK	Homo sapier Gene Symbol	LCK	FALSE	LCK
taxon:9606	171271	1902	PHD finger p	gene	biological_pr	G:9606:PHF20				PHF20	Homo sapier Gene Symbol	PHF20	FALSE	PHF20
taxon:9606	179460	1902	lipocalin 2	gene	biological_pr	G:9606:LCN2			KEGG-&-1&-	LCN2	Homo sapier Gene Symbol	LCN2	FALSE	LCN2
taxon:9606	163082	1902	CREB regulat	gene	biological_pr	G:9606:CRTC2			KEGG-&-1&-	CRTC2	Homo sapier Gene Symbol	CRTC2	FALSE	CRTC2
taxon:9606	175374	1902	nuclear rece	gene	biological_pr	G:9606:NCOR2			KEGG-&-1&-	NCOR2	Homo sapier Gene Symbol	NCOR2	FALSE	NCOR2
taxon:9606	175375	1902	nuclear rece	gene	biological_pr	G:9606:NCOR1			KEGG-&-1&-	NCOR1	Homo sapier Gene Symbol	NCOR1	FALSE	NCOR1
taxon:9606	179474	1902	necdin, MAG	gene	biological_pr	G:9606:NDN				NDN	Homo sapier Gene Symbol	NDN	FALSE	NDN
taxon:9606	179475	1902	nucleolin	gene	biological_pr	G:9606:NCL			KEGG-&-1&-	NCL	Homo sapier Gene Symbol	NCL	FALSE	NCL
taxon:9606	175380	1902	ring finger pr	gene	biological_pr	G:9606:RNF14				RNF14	Homo sapier Gene Symbol	RNF14	FALSE	RNF14
taxon:9606	179482	1902	nibrin	gene	biological_pr	G:9606:NBN			KEGG-&-1&-	NBN	Homo sapier Gene Symbol	NBN	FALSE	NBN
taxon:9606	171291	1902	ninein	gene	biological_pr	G:9606:NIN				NIN	Homo sapier Gene Symbol	NIN	FALSE	NIN
taxon:9606	167195	1902	Cdk5 and Abl	gene	biological_pr	G:9606:CABLES2				CABLES2	Homo sapier Gene Symbol	CABLES2	FALSE	CABLES2
taxon:9606	179481	1902	neural cell a	gene	biological_pr	G:9606:NCAM1			KEGG-&-1&-	NCAM1	Homo sapier Gene Symbol	NCAM1	FALSE	NCAM1
taxon:9606	175390	1902	peroxiredoxi	gene	biological_pr	G:9606:PRDX6			KEGG-&-1&-	PRDX6	Homo sapier Gene Symbol	PRDX6	FALSE	PRDX6
taxon:9606	175391	1902	MAD2L1 bind	gene	biological_pr	G:9606:MAD2L1BP				MAD2L1BP	Homo sapier Gene Symbol	MAD2L1BP	FALSE	MAD2L1BP
taxon:9606	171295	1902	importin 11	gene	biological_pr	G:9606:IPO11				IPO11	Homo sapier Gene Symbol	IPO11	FALSE	IPO11
taxon:9606	175388	1902	A-kinase anc	gene	biological_pr	G:9606:AKAP12				AKAP12	Homo sapier Gene Symbol	AKAP12	FALSE	AKAP12
taxon:9606	171298	1902	HECT and RLI	gene	biological_pr	G:9606:HERC5				HERC5	Homo sapier Gene Symbol	HERC5	FALSE	HERC5
taxon:9606	175394	1902	RNA binding	gene	biological_pr	G:9606:RBM39				RBM39	Homo sapier Gene Symbol	RBM39	FALSE	RBM39
taxon:9606	179491	1902	nucleosome	gene	biological_pr	G:9606:NAP1L1				NAP1L1	Homo sapier Gene Symbol	NAP1L1	FALSE	NAP1L1
taxon:9606	179493	1902	heterogeneo	gene	biological_pr	G:9606:HNRNPM			KEGG-&-1&-	HNRNPM	Homo sapier Gene Symbol	HNRNPM	FALSE	HNRNPM
taxon:9606	163114	1902	THAP domair	gene	molecular_fu	G:9606:THAP8				THAP8	Homo sapier Gene Symbol	THAP8	FALSE	THAP8
taxon:9606	175402	1902	clock circadia	gene	biological_pr	G:9606:CLOCK			KEGG-&-1&-	CLOCK	Homo sapier Gene Symbol	CLOCK	FALSE	CLOCK
taxon:9606	175400	1902	brain and re	gene	biological_pr	G:9606:BRE			KEGG-&-1&-	BRE	Homo sapier Gene Symbol	BRE	FALSE	BRE
taxon:9606	167215	1902	SHANK assoc	gene	biological_pr	G:9606:SHARPIN			KEGG-&-1&-	SHARPIN	Homo sapier Gene Symbol	SHARPIN	FALSE	SHARPIN
taxon:9606	179501	1902	protein phos	gene	biological_pr	G:9606:PPP1R12A			KEGG-&-1&-	PPP1R12A	Homo sapier Gene Symbol	PPP1R12A	FALSE	PPP1R12A
taxon:9606	179506	1902	myogenic dif	gene	biological_pr	G:9606:MYOD1				MYOD1	Homo sapier Gene Symbol	MYOD1	FALSE	MYOD1
taxon:9606	167218	1902	sideroflexin	gene	biological_pr	G:9606:SFXN3				SFXN3	Homo sapier Gene Symbol	SFXN3	FALSE	SFXN3
taxon:9606	167216	1902	mediator cor	gene	biological_pr	G:9606:MED25				MED25	Homo sapier Gene Symbol	MED25	FALSE	MED25
taxon:9606	175415	1902	chromodoma	gene	biological_pr	G:9606:CHD1L				CHD1L	Homo sapier Gene Symbol	CHD1L	FALSE	CHD1L
taxon:9606	154932	1902	histone clust	gene	biological_pr	G:9606:HIST2H3D			KEGG-&-1&-	HIST2H3D	Homo sapier Gene Symbol	HIST2H3D	FALSE	HIST2H3D
taxon:9606	154936	1902	pleckstrin ho	gene	molecular_fu	G:9606:PHLDB3				PHLDB3	Homo sapier Gene Symbol	PHLDB3	FALSE	PHLDB3
taxon:9606	179513	1902	myosin VI	gene	biological_pr	G:9606:MYO6				MYO6	Homo sapier Gene Symbol	MYO6	FALSE	MYO6
taxon:9606	175417	1902	H2A histone	gene	biological_pr	G:9606:H2AFY			KEGG-&-1&-	H2AFY	Homo sapier Gene Symbol	H2AFY	FALSE	H2AFY
taxon:9606	167225	1902	ring finger pr	gene	biological_pr	G:9606:RNF146				RNF146	Homo sapier Gene Symbol	RNF146	FALSE	RNF146
taxon:9606	179518	1902	myosin IC	gene	biological_pr	G:9606:MYO1C				MYO1C	Homo sapier Gene Symbol	MYO1C	FALSE	MYO1C
taxon:9606	171331	1902	inhibitor of g	gene	biological_pr	G:9606:ING4				ING4	Homo sapier Gene Symbol	ING4	FALSE	ING4
taxon:9606	179523	1902	myosin light	gene	biological_pr	G:9606:MYL4			KEGG-&-1&-	MYL4	Homo sapier Gene Symbol	MYL4	FALSE	MYL4
taxon:9606	179520	1902	myosin light	gene	biological_pr	G:9606:MYLK			KEGG-&-1&-	MYLK	Homo sapier Gene Symbol	MYLK	FALSE	MYLK
taxon:9606	179521	1902	myosin light	gene	biological_pr	G:9606:MYL6			KEGG-&-1&-	MYL6	Homo sapier Gene Symbol	MYL6	FALSE	MYL6
taxon:9606	179526	1902	myosin light	gene	biological_pr	G:9606:MYL1				MYL1	Homo sapier Gene Symbol	MYL1	FALSE	MYL1
taxon:9606	171338	1902	COP9 signalo	gene	biological_pr	G:9606:COPS4				COPS4	Homo sapier Gene Symbol	COPS4	FALSE	COPS4
taxon:9606	171336	1902	coiled-coil-he	gene	biological_pr	G:9606:CHCHD2				CHCHD2	Homo sapier Gene Symbol	CHCHD2	FALSE	CHCHD2
taxon:9606	179528	1902	myosin heav	gene	biological_pr	G:9606:MYH10			KEGG-&-1&-	MYH10	Homo sapier Gene Symbol	MYH10	FALSE	MYH10
taxon:9606	179529	1902	myosin heav	gene	biological_pr	G:9606:MYH9			KEGG-&-1&-	MYH9	Homo sapier Gene Symbol	MYH9	FALSE	MYH9
taxon:9606	175439	1902	BCL2 associa	gene	biological_pr	G:9606:BAG5				BAG5	Homo sapier Gene Symbol	BAG5	FALSE	BAG5
taxon:9606	175436	1902	BCL2 associa	gene	biological_pr	G:9606:BAG2			KEGG-&-1&-	BAG2	Homo sapier Gene Symbol	BAG2	FALSE	BAG2
taxon:9606	175437	1902	BCL2 associa	gene	biological_pr	G:9606:BAG3				BAG3	Homo sapier Gene Symbol	BAG3	FALSE	BAG3
taxon:9606	175447	1902	aminopeptid	gene	biological_pr	G:9606:NPEPPS				NPEPPS	Homo sapier Gene Symbol	NPEPPS	FALSE	NPEPPS
taxon:9606	175444	1902	trans-2,3-en	gene	biological_pr	G:9606:TECR			KEGG-&-1&-	TECR	Homo sapier Gene Symbol	TECR	FALSE	TECR
taxon:9606	175445	1902	secretory car	gene	biological_pr	G:9606:SCAMP1				SCAMP1	Homo sapier Gene Symbol	SCAMP1	FALSE	SCAMP1
taxon:9606	179541	1902	v-myc avian	gene	biological_pr	G:9606:MYCN			KEGG-&-1&-	MYCN	Homo sapier Gene Symbol	MYCN	FALSE	MYCN
taxon:9606	171354	1902	ribosomal pr	gene	biological_pr	G:9606:RPL26L1			KEGG-&-1&-	RPL26L1	Homo sapier Gene Symbol	RPL26L1	FALSE	RPL26L1
taxon:9606	179545	1902	v-myc avian	gene	biological_pr	G:9606:MYC			KEGG-&-1&-	MYC	Homo sapier Gene Symbol	MYC	FALSE	MYC
taxon:9606	171358	1902	mitochondria	gene	biological_pr	G:9606:MRPS2			KEGG-&-1&-	MRPS2	Homo sapier Gene Symbol	MRPS2	FALSE	MRPS2
taxon:9606	179549	1902	MYB proto-o	gene	biological_pr	G:9606:MYBL2			KEGG-&-1&-	MYBL2	Homo sapier Gene Symbol	MYBL2	FALSE	MYBL2
taxon:9606	179552	1902	MYB proto-o	gene	biological_pr	G:9606:MYB			KEGG-&-1&-	MYB	Homo sapier Gene Symbol	MYB	FALSE	MYB
taxon:9606	179557	1902	mevalonate	gene	biological_pr	G:9606:MVD			KEGG-&-1&-	MVD	Homo sapier Gene Symbol	MVD	FALSE	MVD
taxon:9606	175475	1902	SLC9A3 regul	gene	biological_pr	G:9606:SLC9A3R1			KEGG-&-1&-	SLC9A3R1	Homo sapier Gene Symbol	SLC9A3R1	FALSE	SLC9A3R1
taxon:9606	179568	1902	mucin 1, cell	gene	biological_pr	G:9606:MUC1				MUC1	Homo sapier Gene Symbol	MUC1	FALSE	MUC1
taxon:9606	163192	1902	argonaute 3,	gene	biological_pr	G:9606:AGO3				AGO3	Homo sapier Gene Symbol	AGO3	FALSE	AGO3
taxon:9606	171398	1902	Sec61 transk	gene	biological_pr	G:9606:SEC61A1			KEGG-&-1&-	SEC61A1	Homo sapier Gene Symbol	SEC61A1	FALSE	SEC61A1
taxon:9606	167303	1902	coiled-coil d	gene	biological_pr	G:9606:CCDC8				CCDC8	Homo sapier Gene Symbol	CCDC8	FALSE	CCDC8
taxon:9606	175493	1902	IST1, ESCRT-	gene	biological_pr	G:9606:IST1			KEGG-&-1&-	IST1	Homo sapier Gene Symbol	IST1	FALSE	IST1
taxon:9606	171401	1902	epsin 1	gene	biological_pr	G:9606:EPN1			KEGG-&-1&-	EPN1	Homo sapier Gene Symbol	EPN1	FALSE	EPN1
taxon:9606	179598	1902	melatonin re	gene	biological_pr	G:9606:MTNR1A			KEGG-&-1&-	MTNR1A	Homo sapier Gene Symbol	MTNR1A	FALSE	MTNR1A
taxon:9606	171407	1902	host cell fact	gene	biological_pr	G:9606:HCFC2			KEGG-&-1&-	HCFC2	Homo sapier Gene Symbol	HCFC2	FALSE	HCFC2
taxon:9606	171404	1902	pyrroline-5-c	gene	biological_pr	G:9606:PYCR2			KEGG-&-1&-	PYCR2	Homo sapier Gene Symbol	PYCR2	FALSE	PYCR2
taxon:9606	179597	1902	melatonin re	gene	biological_pr	G:9606:MTNR1B			KEGG-&-1&-	MTNR1B	Homo sapier Gene Symbol	MTNR1B	FALSE	MTNR1B
taxon:9606	167312	1902	Fc fragment	gene	biological_pr	G:9606:FCAMR				FCAMR	Homo sapier Gene Symbol	FCAMR	FALSE	FCAMR
taxon:9606	171408	1902	UbiA prenylt	gene	biological_pr	G:9606:UBIAD1				UBIAD1	Homo sapier Gene Symbol	UBIAD1	FALSE	UBIAD1
taxon:9606	171414	1902	coiled-coil d	gene	cellular_com	G:9606:CCDC106				CCDC106	Homo sapier Gene Symbol	CCDC106	FALSE	CCDC106
taxon:9606	175515	1902	eukaryotic tr	gene	biological_pr	G:9606:EIF4A3			KEGG-&-1&-	EIF4A3	Homo sapier Gene Symbol	EIF4A3	FALSE	EIF4A3
taxon:9606	179612	1902	methylenete	gene	biological_pr	G:9606:MTHFD1			KEGG-&-1&-	MTHFD1	Homo sapier Gene Symbol	MTHFD1	FALSE	MTHFD1
taxon:9606	171421	1902	PSMC3 intera	gene	biological_pr	G:9606:PSMC3IP				PSMC3IP	Homo sapier Gene Symbol	PSMC3IP	FALSE	PSMC3IP
taxon:9606	179619	1902	ribosomal pr	gene	biological_pr	G:9606:RPSA			KEGG-&-1&-	RPSA	Homo sapier Gene Symbol	RPSA	FALSE	RPSA
taxon:9606	179616	1902	stathmin 1	gene	biological_pr	G:9606:STMN1			KEGG-&-1&-	STMN1	Homo sapier Gene Symbol	STMN1	FALSE	STMN1
taxon:9606	175527	1902	ribosomal pr	gene	biological_pr	G:9606:RPL23			KEGG-&-1&-	RPL23	Homo sapier Gene Symbol	RPL23	FALSE	RPL23
taxon:9606	179620	1902	lysosomal as	gene	biological_pr	G:9606:LAMP2			KEGG-&-1&-	LAMP2	Homo sapier Gene Symbol	LAMP2	FALSE	LAMP2
taxon:9606	175525	1902	SLC9A3 regul	gene	biological_pr	G:9606:SLC9A3R2			KEGG-&-1&-	SLC9A3R2	Homo sapier Gene Symbol	SLC9A3R2	FALSE	SLC9A3R2
taxon:9606	175530	1902	elongation f	gene	biological_pr	G:9606:EFTUD2			KEGG-&-1&-	EFTUD2	Homo sapier Gene Symbol	EFTUD2	FALSE	EFTUD2
taxon:9606	171435	1902	SUMO1/sent	gene	biological_pr	G:9606:SENP1				SENP1	Homo sapier Gene Symbol	SENP1	FALSE	SENP1
taxon:9606	175531	1902	synaptosome	gene	biological_pr	G:9606:SNAP29			KEGG-&-1&-	SNAP29	Homo sapier Gene Symbol	SNAP29	FALSE	SNAP29

taxon:9606	171432	1902	inducible T-c	gene	biological_pr	G:9606:ICOS	KEGG-&-1&-I	ICOS	Homo sapier Gene Symbol	ICOS	FALSE	ICOS
taxon:9606	175534	1902	transcription	gene	biological_pr	G:9606:TCEAL1		TCEAL1	Homo sapier Gene Symbol	TCEAL1	FALSE	TCEAL1
taxon:9606	179628	1902	laminin subu	gene	biological_pr	G:9606:LAMA4	KEGG-&-1&-I	LAMA4	Homo sapier Gene Symbol	LAMA4	FALSE	LAMA4
taxon:9606	179629	1902	laminin subu	gene	biological_pr	G:9606:LAMA3	KEGG-&-1&-I	LAMA3	Homo sapier Gene Symbol	LAMA3	FALSE	LAMA3
taxon:9606	155053	1902	testis specifi	gene	biological_pr	G:9606:TSPY3		TSPY3	Homo sapier Gene Symbol	TSPY3	FALSE	TSPY3
taxon:9606	175536	1902	beta-1,4-gala	gene	biological_pr	G:9606:B4GALT5	KEGG-&-1&-I	B4GALT5	Homo sapier Gene Symbol	B4GALT5	FALSE	B4GALT5
taxon:9606	175543	1902	zinc finger HI	gene	biological_pr	G:9606:ZNHIT3		ZNHIT3	Homo sapier Gene Symbol	ZNHIT3	FALSE	ZNHIT3
taxon:9606	175540	1902	general trans	gene	biological_pr	G:9606:GTF3C3		GTF3C3	Homo sapier Gene Symbol	GTF3C3	FALSE	GTF3C3
taxon:9606	175541	1902	general trans	gene	biological_pr	G:9606:GTF3C4		GTF3C4	Homo sapier Gene Symbol	GTF3C4	FALSE	GTF3C4
taxon:9606	175544	1902	thyroid horm	gene	biological_pr	G:9606:TRIP4		TRIP4	Homo sapier Gene Symbol	TRIP4	FALSE	TRIP4
taxon:9606	179640	1902	kinectin 1	gene	biological_pr	G:9606:KTN1		KTN1	Homo sapier Gene Symbol	KTN1	FALSE	KTN1
taxon:9606	175550	1902	COP9 signalo	gene	biological_pr	G:9606:COPS2		COPS2	Homo sapier Gene Symbol	COPS2	FALSE	COPS2
taxon:9606	175548	1902	thyroid horm	gene	biological_pr	G:9606:TRIP12	KEGG-&-1&-I	TRIP12	Homo sapier Gene Symbol	TRIP12	FALSE	TRIP12
taxon:9606	175553	1902	Kruppel like f	gene	biological_pr	G:9606:KLF4	KEGG-&-1&-I	KLF4	Homo sapier Gene Symbol	KLF4	FALSE	KLF4
taxon:9606	175559	1902	suppressor o	gene	biological_pr	G:9606:SOCS6	KEGG-&-1&-I	SOCS6	Homo sapier Gene Symbol	SOCS6	FALSE	SOCS6
taxon:9606	179653	1902	keratin 19	gene	biological_pr	G:9606:KRT19	KEGG-&-1&-I	KRT19	Homo sapier Gene Symbol	KRT19	FALSE	KRT19
taxon:9606	171466	1902	ubiquitin like	gene	biological_pr	G:9606:UHRF1		UHRF1	Homo sapier Gene Symbol	UHRF1	FALSE	UHRF1
taxon:9606	179656	1902	keratin 18	gene	biological_pr	G:9606:KRT18	KEGG-&-1&-I	KRT18	Homo sapier Gene Symbol	KRT18	FALSE	KRT18
taxon:9606	171465	1902	B-cell linker	gene	biological_pr	G:9606:BLNK	KEGG-&-1&-I	BLNK	Homo sapier Gene Symbol	BLNK	FALSE	BLNK
taxon:9606	179663	1902	keratin 10	gene	biological_pr	G:9606:KRT10	KEGG-&-1&-I	KRT10	Homo sapier Gene Symbol	KRT10	FALSE	KRT10
taxon:9606	167379	1902	tubulin beta	gene	biological_pr	G:9606:TUBB1	KEGG-&-1&-I	TUBB1	Homo sapier Gene Symbol	TUBB1	FALSE	TUBB1
taxon:9606	171472	1902	protease, ser	gene	biological_pr	G:9606:PRSS50		PRSS50	Homo sapier Gene Symbol	PRSS50	FALSE	PRSS50
taxon:9606	179664	1902	keratin 9	gene	biological_pr	G:9606:KRT9	KEGG-&-1&-I	KRT9	Homo sapier Gene Symbol	KRT9	FALSE	KRT9
taxon:9606	179665	1902	keratin 8	gene	biological_pr	G:9606:KRT8		KRT8	Homo sapier Gene Symbol	KRT8	FALSE	KRT8
taxon:9606	171476	1902	bromodomai	gene	biological_pr	G:9606:BRD7	KEGG-&-1&-I	BRD7	Homo sapier Gene Symbol	BRD7	FALSE	BRD7
taxon:9606	163284	1902	tripartite mo	gene	biological_pr	G:9606:TRIM65		TRIM65	Homo sapier Gene Symbol	TRIM65	FALSE	TRIM65
taxon:9606	175579	1902	mediator cor	gene	biological_pr	G:9606:MED14	KEGG-&-1&-I	MED14	Homo sapier Gene Symbol	MED14	FALSE	MED14
taxon:9606	179672	1902	keratin 2	gene	biological_pr	G:9606:KRT2		KRT2	Homo sapier Gene Symbol	KRT2	FALSE	KRT2
taxon:9606	171481	1902	TANK binding	gene	biological_pr	G:9606:TBK1	KEGG-&-1&-I	TBK1	Homo sapier Gene Symbol	TBK1	FALSE	TBK1
taxon:9606	179673	1902	keratin 1	gene	biological_pr	G:9606:KRT1		KRT1	Homo sapier Gene Symbol	KRT1	FALSE	KRT1
taxon:9606	167391	1902	SET domain c	gene	biological_pr	G:9606:SETD7	KEGG-&-1&-I	SETD7	Homo sapier Gene Symbol	SETD7	FALSE	SETD7
taxon:9606	179677	1902	importin 5	gene	biological_pr	G:9606:IPO5		IPO5	Homo sapier Gene Symbol	IPO5	FALSE	IPO5
taxon:9606	179682	1902	karyopherin s	gene	biological_pr	G:9606:KPNA2	KEGG-&-1&-I	KPNA2	Homo sapier Gene Symbol	KPNA2	FALSE	KPNA2
taxon:9606	179683	1902	karyopherin s	gene	biological_pr	G:9606:KPNB1	KEGG-&-1&-I	KPNB1	Homo sapier Gene Symbol	KPNB1	FALSE	KPNB1
taxon:9606	179680	1902	karyopherin s	gene	biological_pr	G:9606:KPNA4		KPNA4	Homo sapier Gene Symbol	KPNA4	FALSE	KPNA4
taxon:9606	171489	1902	drosha ribon	gene	biological_pr	G:9606:DROSHA	KEGG-&-1&-I	DROSHA	Homo sapier Gene Symbol	DROSHA	FALSE	DROSHA
taxon:9606	175585	1902	piwi like RNA	gene	biological_pr	G:9606:PIWIL1		PIWIL1	Homo sapier Gene Symbol	PIWIL1	FALSE	PIWIL1
taxon:9606	179681	1902	karyopherin s	gene	biological_pr	G:9606:KPNA3		KPNA3	Homo sapier Gene Symbol	KPNA3	FALSE	KPNA3
taxon:9606	175593	1902	PDZ and LIM	gene	biological_pr	G:9606:PDLIM7		PDLIM7	Homo sapier Gene Symbol	PDLIM7	FALSE	PDLIM7
taxon:9606	167407	1902	centrosomal	gene	cellular_com	G:9606:CEP44		CEP44	Homo sapier Gene Symbol	CEP44	FALSE	CEP44
taxon:9606	175599	1902	ribosomal pr	gene	biological_pr	G:9606:RPS6KA5	KEGG-&-1&-I	RPS6KA5	Homo sapier Gene Symbol	RPS6KA5	FALSE	RPS6KA5
taxon:9606	167410	1902	integrator co	gene	biological_pr	G:9606:INTS5		INTS5	Homo sapier Gene Symbol	INTS5	FALSE	INTS5
taxon:9606	167411	1902	collagen type	gene	biological_pr	G:9606:COL18A1	KEGG-&-1&-I	COL18A1	Homo sapier Gene Symbol	COL18A1	FALSE	COL18A1
taxon:9606	179697	1902	kallikrein rel	gene	biological_pr	G:9606:KLK2	KEGG-&-1&-I	KLK2	Homo sapier Gene Symbol	KLK2	FALSE	KLK2
taxon:9606	171510	1902	mediator cor	gene	biological_pr	G:9606:MED4	KEGG-&-1&-I	MED4	Homo sapier Gene Symbol	MED4	FALSE	MED4
taxon:9606	179700	1902	KiSS-1 meta	gene	biological_pr	G:9606:KISS1	KEGG-&-1&-I	KISS1	Homo sapier Gene Symbol	KISS1	FALSE	KISS1
taxon:9606	171514	1902	SET domain c	gene	biological_pr	G:9606:SETD2	KEGG-&-1&-I	SETD2	Homo sapier Gene Symbol	SETD2	FALSE	SETD2
taxon:9606	175612	1902	pituitary tum	gene	biological_pr	G:9606:PTTG1	KEGG-&-1&-I	PTTG1	Homo sapier Gene Symbol	PTTG1	FALSE	PTTG1
taxon:9606	167426	1902	inter-alpha-t	gene	biological_pr	G:9606:ITIH5		ITIH5	Homo sapier Gene Symbol	ITIH5	FALSE	ITIH5
taxon:9606	179714	1902	kinesin famil	gene	biological_pr	G:9606:KIF5B	KEGG-&-1&-I	KIF5B	Homo sapier Gene Symbol	KIF5B	FALSE	KIF5B
taxon:9606	179713	1902	kinesin famil	gene	biological_pr	G:9606:KIF5C	KEGG-&-1&-I	KIF5C	Homo sapier Gene Symbol	KIF5C	FALSE	KIF5C
taxon:9606	175622	1902	VAMP associ	gene	biological_pr	G:9606:VAPA	KEGG-&-1&-I	VAPA	Homo sapier Gene Symbol	VAPA	FALSE	VAPA
taxon:9606	175621	1902	metastasis a	gene	biological_pr	G:9606:MTA2		MTA2	Homo sapier Gene Symbol	MTA2	FALSE	MTA2
taxon:9606	175627	1902	aurora kinase	gene	biological_pr	G:9606:AURKB		AURKB	Homo sapier Gene Symbol	AURKB	FALSE	AURKB
taxon:9606	179720	1902	kinase insert	gene	biological_pr	G:9606:KDR	KEGG-&-1&-I	KDR	Homo sapier Gene Symbol	KDR	FALSE	KDR
taxon:9606	171534	1902	homeodomai	gene	biological_pr	G:9606:HIPK2	KEGG-&-1&-I	HIPK2	Homo sapier Gene Symbol	HIPK2	FALSE	HIPK2
taxon:9606	175630	1902	LRR binding f	gene	biological_pr	G:9606:LRRFIP2		LRRFIP2	Homo sapier Gene Symbol	LRRFIP2	FALSE	LRRFIP2
taxon:9606	171536	1902	MACRO dom	gene	biological_pr	G:9606:MACROD1		MACROD1	Homo sapier Gene Symbol	MACROD1	FALSE	MACROD1
taxon:9606	175638	1902	doublecortin	gene	biological_pr	G:9606:DCLK1		DCLK1	Homo sapier Gene Symbol	DCLK1	FALSE	DCLK1
taxon:9606	175646	1902	DExD-box hel	gene	biological_pr	G:9606:DDX21		DDX21	Homo sapier Gene Symbol	DDX21	FALSE	DDX21
taxon:9606	171552	1902	mitochondria	gene	biological_pr	G:9606:MRPS18B	KEGG-&-1&-I	MRPS18B	Homo sapier Gene Symbol	MRPS18B	FALSE	MRPS18B
taxon:9606	167456	1902	ring finger pr	gene	biological_pr	G:9606:RNF39		RNF39	Homo sapier Gene Symbol	RNF39	FALSE	RNF39
taxon:9606	175648	1902	RALBP1 asso	gene	biological_pr	G:9606:REPS2		REPS2	Homo sapier Gene Symbol	REPS2	FALSE	REPS2
taxon:9606	175649	1902	BUB3, mitoti	gene	biological_pr	G:9606:BUB3	KEGG-&-1&-I	BUB3	Homo sapier Gene Symbol	BUB3	FALSE	BUB3
taxon:9606	179750	1902	potassium vo	gene	biological_pr	G:9606:KCNJ1	KEGG-&-1&-I	KCNJ1	Homo sapier Gene Symbol	KCNJ1	FALSE	KCNJ1
taxon:9606	175659	1902	lysophosphat	gene	biological_pr	G:9606:LPAR2	KEGG-&-1&-I	LPAR2	Homo sapier Gene Symbol	LPAR2	FALSE	LPAR2
taxon:9606	171565	1902	mitochondria	gene	biological_pr	G:9606:MRPS28		MRPS28	Homo sapier Gene Symbol	MRPS28	FALSE	MRPS28
taxon:9606	167469	1902	WD repeat d	gene	biological_pr	G:9606:WDR82	KEGG-&-1&-I	WDR82	Homo sapier Gene Symbol	WDR82	FALSE	WDR82
taxon:9606	138803	1902	LOC400927-C	gene	biological_pr	G:9606:LOC400927-CSNK1E	KEGG-&-1&-I	LOC400927-C	Homo sapier Gene Symbol	LOC400927-C	FALSE	LOC400927-CSNK1E
taxon:9606	167472	1902	DnaJ heat sh	gene	biological_pr	G:9606:DNAJC5	KEGG-&-1&-I	DNAJC5	Homo sapier Gene Symbol	DNAJC5	FALSE	DNAJC5
taxon:9606	179767	1902	potassium vo	gene	biological_pr	G:9606:KCNA5		KCNA5	Homo sapier Gene Symbol	KCNA5	FALSE	KCNA5
taxon:9606	179768	1902	potassium vo	gene	biological_pr	G:9606:KCNA4	KEGG-&-1&-I	KCNA4	Homo sapier Gene Symbol	KCNA4	FALSE	KCNA4
taxon:9606	175676	1902	hepatocyte g	gene	biological_pr	G:9606:HGS	KEGG-&-1&-I	HGS	Homo sapier Gene Symbol	HGS	FALSE	HGS
taxon:9606	179778	1902	JunB proto-o	gene	biological_pr	G:9606:JUNB	KEGG-&-1&-I	JUNB	Homo sapier Gene Symbol	JUNB	FALSE	JUNB
taxon:9606	179779	1902	Jun proto-on	gene	biological_pr	G:9606:JUN	KEGG-&-1&-I	JUN	Homo sapier Gene Symbol	JUN	FALSE	JUN
taxon:9606	175681	1902	programmed	gene	biological_pr	G:9606:PDCD5		PDCD5	Homo sapier Gene Symbol	PDCD5	FALSE	PDCD5
taxon:9606	179777	1902	JunD proto-o	gene	biological_pr	G:9606:JUND	KEGG-&-1&-I	JUND	Homo sapier Gene Symbol	JUND	FALSE	JUND
taxon:9606	179783	1902	Janus kinase	gene	biological_pr	G:9606:JAK2	KEGG-&-1&-I	JAK2	Homo sapier Gene Symbol	JAK2	FALSE	JAK2
taxon:9606	175690	1902	apoptosis in	gene	biological_pr	G:9606:AIFM1	KEGG-&-1&-I	AIFM1	Homo sapier Gene Symbol	AIFM1	FALSE	AIFM1
taxon:9606	179784	1902	Janus kinase	gene	biological_pr	G:9606:JAK1	KEGG-&-1&-I	JAK1	Homo sapier Gene Symbol	JAK1	FALSE	JAK1
taxon:9606	179793	1902	inositol-tetra	gene	biological_pr	G:9606:ITPK1	KEGG-&-1&-I	ITPK1	Homo sapier Gene Symbol	ITPK1	FALSE	ITPK1
taxon:9606	163415	1902	torsin 1A int	gene	biological_pr	G:9606:TOR1AIP2		TOR1AIP2	Homo sapier Gene Symbol	TOR1AIP2	FALSE	TOR1AIP2
taxon:9606	179797	1902	integrin subu	gene	biological_pr	G:9606:ITGA4	KEGG-&-1&-I	ITGA4	Homo sapier Gene Symbol	ITGA4	FALSE	ITGA4
taxon:9606	175707	1902	synaptosome	gene	biological_pr	G:9606:SNAP23	KEGG-&-1&-I	SNAP23	Homo sapier Gene Symbol	SNAP23	FALSE	SNAP23
taxon:9606	179806	1902	interferon re	gene	biological_pr	G:9606:IRF7	KEGG-&-1&-I	IRF7	Homo sapier Gene Symbol	IRF7	FALSE	IRF7
taxon:9606	175711	1902	RAB11A, me	gene	biological_pr	G:9606:RAB11A	KEGG-&-1&-I	RAB11A	Homo sapier Gene Symbol	RAB11A	FALSE	RAB11A
taxon:9606	175708	1902	Fas associat	gene	biological_pr	G:9606:FADD	KEGG-&-1&-I	FADD	Homo sapier Gene Symbol	FADD	FALSE	FADD
taxon:9606	179805	1902	insulin recep	gene	biological_pr	G:9606:IRS1	KEGG-&-1&-I	IRS1	Homo sapier Gene Symbol	IRS1	FALSE	IRS1
taxon:9606	175719	1902	ADAM metal	gene	biological_pr	G:9606:ADAM9		ADAM9	Homo sapier Gene Symbol	ADAM9	FALSE	ADAM9
taxon:9606	171620	1902	zinc finger HI	gene	biological_pr	G:9606:ZNHIT6		ZNHIT6	Homo sapier Gene Symbol	ZNHIT6	FALSE	ZNHIT6
taxon:9606	167531	1902	WD repeat d	gene	biological_pr	G:9606:WDR76		WDR76	Homo sapier Gene Symbol	WDR76	FALSE	WDR76
taxon:9606	175725	1902	tumor necro	gene	biological_pr	G:9606:TNFSF9	KEGG-&-1&-I	TNFSF9	Homo sapier Gene Symbol	TNFSF9	FALSE	TNFSF9
taxon:9606	179821	1902	eukaryotic tr	gene	biological_pr	G:9606:EIF3E	KEGG-&-1&-I	EIF3E	Homo sapier Gene Symbol	EIF3E	FALSE	EIF3E
taxon:9606	179831	1902	minichromos	gene	biological_pr	G:9606:MCM7	KEGG-&-1&-I	MCM7	Homo sapier Gene Symbol	MCM7	FALSE	MCM7
taxon:9606	175732	1902	receptor inte	gene	biological_pr	G:9606:RIPK1	KEGG-&-1&-I	RIPK1	Homo sapier Gene Symbol	RIPK1	FALSE	RIPK1
taxon:9606	179828	1902	inositol polyp	gene	biological_pr	G:9606:INPPL1	KEGG-&-1&-I	INPPL1	Homo sapier Gene Symbol	INPPL1	FALSE	INPPL1
taxon:9606	179834	1902	minichromos	gene	biological_pr	G:9606:MCM4	KEGG-&-1&-I	MCM4	Homo sapier Gene Symbol	MCM4	FALSE	MCM4
taxon:9606	171643	1902	INO80 compl	gene	biological_pr	G:9606:INO80		INO80	Homo sapier Gene Symbol	INO80	FALSE	INO80
taxon:9606	179835	1902	minichromos	gene	biological_pr	G:9606:MCM3	KEGG-&-1&-I	MCM3	Homo sapier Gene Symbol	MCM3	FALSE	MCM3
taxon:9606	179832	1902	minichromos	gene	biological_pr	G:9606:MCM6	KEGG-&-1&-I	MCM6	Homo sapier Gene Symbol	MCM6	FALSE	MCM6
taxon:9606	179833	1902	minichromos	gene	biological_pr	G:9606:MCM5	KEGG-&-1&-I	MCM5	Homo sapier Gene Symbol	MCM5	FALSE	MCM5
taxon:9606	175740	1902	ADAM metal	gene	biological_pr	G:9606:ADAMTS1		ADAMTS1	Homo sapier Gene Symbol	ADAMTS1	FALSE	ADAMTS1
taxon:9606	179836	1902	minichromos	gene	biological_pr	G:9606:MCM2	KEGG-&-1&-I	MCM2	Homo sapier Gene Symbol	MCM2	FALSE	MCM2
taxon:9606	179837	1902	BCL2 family	gene	biological_pr	G:9606:MCL1	KEGG-&-1&-I	MCL1	Homo sapier Gene Symbol	MCL1	FALSE	MCL1
taxon:9606	167558	1902	microtubule	gene	biological_pr	G:9606:MAP1LC3B	KEGG-&-1&-I	MAP1LC3B	Homo sapier Gene Symbol	MAP1LC3B	FALSE	MAP1LC3B
taxon:9606	175750	1902	solute carrier	gene	biological_pr	G:9606:SLC4A7		SLC4A7	Homo sapier Gene Symbol	SLC4A7	FALSE	SLC4A7
taxon:9606	175749	1902	solute carrier	gene	biological_pr	G:9606:SLC4A8		SLC4A8	Homo sapier Gene Symbol	SLC4A8	FALSE	SLC4A8
taxon:9606	179850	1902	muscleblind	gene	biological_pr	G:9606:MBNL1		MBNL1	Homo sapier Gene Symbol	MBNL1	FALSE	MBNL1
taxon:9606	167563	1902	diaphanous r	gene	biological_pr	G:9606:DIAPH3	KEGG-&-1&-I	DIAPH3	Homo sapier Gene Symbol	DIAPH3	FALSE	DIAPH3
taxon:9606	163471	1902	zinc finger pr	gene	biological_pr	G:9606:ZNF483		ZNF483	Homo sapier Gene Symbol	ZNF483	FALSE	ZNF483
taxon:9606	179859	1902	megakaryocy	gene	biological_pr	G:9606:MATK	KEGG-&-1&-I	MATK	Homo sapier Gene Symbol	MATK	FALSE	MATK
taxon:9606	175767	1902	autophagy re	gene	biological_pr	G:9606:ATG5	KEGG-&-1&-I	ATG5	Homo sapier Gene Symbol	ATG5	FALSE	ATG5
taxon:9606	179863	1902	methionyl-tR	gene	biological_pr	G:9606:MARS	KEGG-&-1&-I	MARS	Homo sapier Gene Symbol	MARS	FALSE	MARS
taxon:9606	175764	1902	mediator cor	gene	biological_pr	G:9606:MED20		MED20	Homo sapier Gene Symbol	MED20	FALSE	MED20
taxon:9606	175770	1902	eukaryotic tr	gene	biological_pr	G:9606:EIF4E2	KEGG-&-1&-I	EIF4E2	Homo sapier Gene Symbol	EIF4E2	FALSE	EIF4E2
taxon:9606	179866	1902	microtubule	gene	biological_pr	G:9606:MAPT	KEGG-&-1&-I	MAPT	Homo sapier Gene Symbol	MAPT	FALSE	MAPT
taxon:9606	171675	1902	G protein nu	gene	biological_pr	G:9606:GNL3L	KEGG-&-1&-I	GNL3L	Homo sapier Gene Symbol	GNL3L	FALSE	GNL3L
taxon:9606	175769	1902	A-kinase anc	gene	biological_pr	G:9606:AKAP6		AKAP6	Homo sapier Gene Symbol	AKAP6	FALSE	AKAP6
taxon:9606	179870	1902	microtubule	gene	biological_pr	G:9606:MAP1B		MAP1B	Homo sapier Gene Symbol	MAP1B	FALSE	MAP1B
taxon:9606	167581	1902	tripartite mo	gene	biological_pr	G:9606:TRIM8		TRIM8	Homo sapier Gene Symbol	TRIM8	FALSE	TRIM8
taxon:9606	155298	1902	glucuronidas	gene		G:9606:GUSBP3		GUSBP3	Homo sapier Gene Symbol	GUSBP3	FALSE	GUSBP3
taxon:9606	179881	1902	male germ c	gene	biological_pr	G:9606:MAK		MAK	Homo sapier Gene Symbol	MAK	FALSE	MAK
taxon:9606	171689	1902	exocyst comp	gene	biological_pr	G:9606:EXOC6		EXOC6	Homo sapier Gene Symbol	EXOC6	FALSE	EXOC6
taxon:9606	155314	1902	zinc finger ar	gene	biological_pr	G:9606:ZBTB8A		ZBTB8A	Homo sapier Gene Symbol	ZBTB8A	FALSE	ZBTB8A
taxon:9606	171699	1902	REV1, DNA d	gene	biological_pr	G:9606:REV1	KEGG-&-1&-I	REV1	Homo sapier Gene Symbol	REV1	FALSE	REV1
taxon:9606	179888	1902	MAGE family	gene	cellular_com	G:9606:MAGEA11		MAGEA11	Homo sapier Gene Symbol	MAGEA11	FALSE	MAGEA11
taxon:9606	175793	1902	mediator cor	gene	biological_pr	G:9606:MED7		MED7	Homo sapier Gene Symbol	MED7	FALSE	MED7
taxon:9606	175796	1902	mediator cor	gene	biological_pr	G:9606:MED17	KEGG-&-1&-I	MED17	Homo sapier Gene Symbol	MED17	FALSE	MED17
taxon:9606	175797	1902	mediator cor	gene	biological_pr	G:9606:MED23		MED23	Homo sapier Gene Symbol	MED23	FALSE	MED23
taxon:9606	171707	1902	YTH N6-meth	gene	biological_pr	G:9606:YTHDF2		YTHDF2	Homo sapier Gene Symbol	YTHDF2	FALSE	YTHDF2
taxon:9606	171704	1902	inositol hexa	gene	biological_pr	G:9606:IP6K2	KEGG-&-1&-I	IP6K2	Homo sapier Gene Symbol	IP6K2	FALSE	IP6K2
taxon:9606	179897	1902	MAGE family	gene	biological_pr	G:9606:MAGEA2		MAGEA2	Homo sapier Gene Symbol	MAGEA2	FALSE	MAGEA2
taxon:9606	171710	1902	MAGE family	gene	biological_pr	G:9606:MAGEC2		MAGEC2	Homo sapier Gene Symbol	MAGEC2	FALSE	MAGEC2
taxon:9606	175807	1902	chromodoma	gene	biological_pr	G:9606:CDYL		CDYL	Homo sapier Gene Symbol	CDYL	FALSE	CDYL
taxon:9606	163516	1902	additional se	gene	biological_pr	G:9606:ASXL1		ASXL1	Homo sapier Gene Symbol	ASXL1	FALSE	ASXL1
taxon:9606	179900	1902	MAF bZIP tra	gene	biological_pr	G:9606:MAFG		MAFG	Homo sapier Gene Symbol	MAFG	FALSE	MAFG
taxon:9606	171715	1902	sorting nexin	gene	biological_pr	G:9606:SNX9		SNX9	Homo sapier Gene Symbol	SNX9	FALSE	SNX9
taxon:9606	179905	1902	SMAD family	gene	biological_pr	G:9606:SMAD7	KEGG-&-1&-I	SMAD7	Homo sapier Gene Symbol	SMAD7	FALSE	SMAD7
taxon:9606	179910	1902	SMAD family	gene	biological_pr	G:9606:SMAD2	KEGG-&-1&-I	SMAD2	Homo sapier Gene Symbol	SMAD2	FALSE	SMAD2
taxon:9606	179911	1902	SMAD family	gene	biological_pr	G:9606:SMAD1	KEGG-&-1&-I	SMAD1	Homo sapier Gene Symbol	SMAD1	FALSE	SMAD1
taxon:9606	179908	1902	SMAD family	gene	biological_pr	G:9606:SMAD4	KEGG-&-1&-I	SMAD4	Homo sapier Gene Symbol	SMAD4	FALSE	SMAD4
taxon:9606	179909	1902	SMAD family	gene	biological_pr	G:9606:SMAD3	KEGG-&-1&-I	SMAD3	Homo sapier Gene Symbol	SMAD3	FALSE	SMAD3
taxon:9606	175818	1902	mediator cor	gene	biological_pr	G:9606:MED21		MED21	Homo sapier Gene Symbol	MED21	FALSE	MED21
taxon:9606	175819	1902	Rho GTPase	gene	biological_pr	G:9606:ARHGAP29		ARHGAP29	Homo sapier Gene Symbol	ARHGAP29	FALSE	ARHGAP29
taxon:9606	179912	1902	MAD2 mitoti	gene	biological_pr	G:9606:MAD2L1	KEGG-&-1&-I	MAD2L1	Homo sapier Gene Symbol	MAD2L1	FALSE	MAD2L1
taxon:9606	175816	1902	tight junctior	gene	biological_pr	G:9606:TJP2	KEGG-&-1&-I	TJP2	Homo sapier Gene Symbol	TJP2	FALSE	TJP2
taxon:9606	175826	1902	GRB2-relate	gene	biological_pr	G:9606:GRAP2	KEGG-&-1&-I	GRAP2	Homo sapier Gene Symbol	GRAP2	FALSE	GRAP2
taxon:9606	175827	1902	RecQ like hel	gene	biological_pr	G:9606:RECQL4		RECQL4	Homo sapier Gene Symbol	RECQL4	FALSE	RECQL4
taxon:9606	179921	1902	epithelial cel	gene	biological_pr	G:9606:EPCAM		EPCAM	Homo sapier Gene Symbol	EPCAM	FALSE	EPCAM
taxon:9606	179926	1902	LYN proto-on	gene	biological_pr	G:9606:LYN	KEGG-&-1&-I	LYN	Homo sapier Gene Symbol	LYN	FALSE	LYN
taxon:9606	175831	1902	N-myristoylt	gene	biological_pr	G:9606:NMT2		NMT2	Homo sapier Gene Symbol	NMT2	FALSE	NMT2
taxon:9606	179924	1902	lysozyme	gene	biological_pr	G:9606:LYZ	KEGG-&-1&-I	LYZ	Homo sapier Gene Symbol	LYZ	FALSE	LYZ
taxon:9606	179925	1902	SH2 domain c	gene	biological_pr	G:9606:SH2D1A	KEGG-&-1&-I	SH2D1A	Homo sapier Gene Symbol	SH2D1A	FALSE	SH2D1A
taxon:9606	171733	1902	eukaryotic tr	gene	biological_pr	G:9606:EIF3L		EIF3L	Homo sapier Gene Symbol	EIF3L	FALSE	EIF3L
taxon:9606	175838	1902	embryonic e	gene	biological_pr	G:9606:EED		EED	Homo sapier Gene Symbol	EED	FALSE	EED
taxon:9606	179938	1902	lymphotoxin	gene	biological_pr	G:9606:LTBR	KEGG-&-1&-I	LTBR	Homo sapier Gene Symbol	LTBR	FALSE	LTBR
taxon:9606	175840	1902	sorting nexin	gene	biological_pr	G:9606:SNX3	KEGG-&-1&-I	SNX3	Homo sapier Gene Symbol	SNX3	FALSE	SNX3
taxon:9606	175841	1902	sorting nexin	gene	biological_pr	G:9606:SNX4	KEGG-&-1&-I	SNX4	Homo sapier Gene Symbol	SNX4	FALSE	SNX4
taxon:9606	175846	1902	TNFRSF1A a	gene	biological_pr	G:9606:TRADD	KEGG-&-1&-I	TRADD	Homo sapier Gene Symbol	TRADD	FALSE	TRADD
taxon:9606	171748	1902	ubiquitin pro	gene	biological_pr	G:9606:UBR5	KEGG-&-1&-I	UBR5	Homo sapier Gene Symbol	UBR5	FALSE	UBR5
taxon:9606	175844	1902	membrane b	gene	biological_pr	G:9606:MBTPS1	KEGG-&-1&-I	MBTPS1	Homo sapier Gene Symbol	MBTPS1	FALSE	MBTPS1
taxon:9606	179944	1902	lymphotoxin	gene	biological_pr	G:9606:LTA	KEGG-&-1&-I	LTA	Homo sapier Gene Symbol	LTA	FALSE	LTA
taxon:9606	179945	1902	leukotriene A	gene	biological_pr	G:9606:LTA4H	KEGG-&-1&-I	LTA4H	Homo sapier Gene Symbol	LTA4H	FALSE	LTA4H
taxon:9606	171762	1902	zinc finger ar	gene	biological_pr	G:9606:ZBTB7A		ZBTB7A	Homo sapier Gene Symbol	ZBTB7A	FALSE	ZBTB7A
taxon:9606	179955	1902	LDL receptor	gene	biological_pr	G:9606:LRP1	KEGG-&-1&-I	LRP1	Homo sapier Gene Symbol	LRP1	FALSE	LRP1
taxon:9606	171761	1902	fizzy and cell	gene	biological_pr	G:9606:FZR1	KEGG-&-1&-I	FZR1	Homo sapier Gene Symbol	FZR1	FALSE	FZR1
taxon:9606	171766	1902	thioesterase	gene	cellular_com	G:9606:THEM6		THEM6	Homo sapier Gene Symbol	THEM6	FALSE	THEM6
taxon:9606	179970	1902	leucyl and cy	gene	biological_pr	G:9606:LNPEP	KEGG-&-1&-I	LNPEP	Homo sapier Gene Symbol	LNPEP	FALSE	LNPEP
taxon:9606	175872	1902	serine and ar	gene	biological_pr	G:9606:SRSF9	KEGG-&-1&-I	SRSF9	Homo sapier Gene Symbol	SRSF9	FALSE	SRSF9
taxon:9606	163585	1902	family with s	gene	cellular_com	G:9606:FAM84B		FAM84B	Homo sapier Gene Symbol	FAM84B	FALSE	FAM84B
taxon:9606	179973	1902	LIM domain 7	gene	biological_pr	G:9606:LMO7	KEGG-&-1&-I	LMO7	Homo sapier Gene Symbol	LMO7	FALSE	LMO7
taxon:9606	179978	1902	lamin A/C	gene	biological_pr	G:9606:LMNA	KEGG-&-1&-I	LMNA	Homo sapier Gene Symbol	LMNA	FALSE	LMNA
taxon:9606	175880	1902	vesicle assoc	gene	biological_pr	G:9606:VAMP8	KEGG-&-1&-I	VAMP8	Homo sapier Gene Symbol	VAMP8	FALSE	VAMP8
taxon:9606	179977	1902	lamin B1	gene	cellular_com	G:9606:LMNB1	KEGG-&-1&-I	LMNB1	Homo sapier Gene Symbol	LMNB1	FALSE	LMNB1
taxon:9606	175884	1902	eukaryotic tr	gene	biological_pr	G:9606:EIF3I	KEGG-&-1&-I	EIF3I	Homo sapier Gene Symbol	EIF3I	FALSE	EIF3I
taxon:9606	175890	1902	eukaryotic tr	gene	biological_pr	G:9606:EIF3B	KEGG-&-1&-I	EIF3B	Homo sapier Gene Symbol	EIF3B	FALSE	EIF3B
taxon:9606	175891	1902	eukaryotic tr	gene	biological_pr	G:9606:EIF3A	KEGG-&-1&-I	EIF3A	Homo sapier Gene Symbol	EIF3A	FALSE	EIF3A
taxon:9606	175888	1902	eukaryotic tr	gene	biological_pr	G:9606:EIF3D	KEGG-&-1&-I	EIF3D	Homo sapier Gene Symbol	EIF3D	FALSE	EIF3D
taxon:9606	163606	1902	anterior grad	gene	biological_pr	G:9606:AGR3		AGR3	Homo sapier Gene Symbol	AGR3	FALSE	AGR3
taxon:9606	167702	1902	KIAA0319 lik	gene	cellular_com	G:9606:KIAA0319L		KIAA0319L	Homo sapier Gene Symbol	KIAA0319L	FALSE	KIAA0319L
taxon:9606	175892	1902	insulin recep	gene	biological_pr	G:9606:IRS2	KEGG-&-1&-I	IRS2	Homo sapier Gene Symbol	IRS2	FALSE	IRS2
taxon:9606	175898	1902	suppressor o	gene	biological_pr	G:9606:SOCS1	KEGG-&-1&-I	SOCS1	Homo sapier Gene Symbol	SOCS1	FALSE	SOCS1
taxon:9606	179994	1902	DNA ligase 3	gene	biological_pr	G:9606:LIG3	KEGG-&-1&-I	LIG3	Homo sapier Gene Symbol	LIG3	FALSE	LIG3
taxon:9606	175899	1902	NUMB, endo	gene	biological_pr	G:9606:NUMB	KEGG-&-1&-I	NUMB	Homo sapier Gene Symbol	NUMB	FALSE	NUMB
taxon:9606	167711	1902	transcription	gene	biological_pr	G:9606:TCEAL4		TCEAL4	Homo sapier Gene Symbol	TCEAL4	FALSE	TCEAL4
taxon:9606	175901	1902	nuclear rece	gene	biological_pr	G:9606:NCOA1	KEGG-&-1&-I	NCOA1	Homo sapier Gene Symbol	NCOA1	FALSE	NCOA1
taxon:9606	180006	1902	inhibitor of g	gene	biological_pr	G:9606:ING1		ING1	Homo sapier Gene Symbol	ING1	FALSE	ING1
taxon:9606	180005	1902	inhibitor of g	gene	biological_pr	G:9606:ING2		ING2	Homo sapier Gene Symbol	ING2	FALSE	ING2
taxon:9606	180015	1902	interleukin e	gene	biological_pr	G:9606:ILF3		ILF3	Homo sapier Gene Symbol	ILF3	FALSE	ILF3
taxon:9606	175920	1902	tumor protei	gene	biological_pr	G:9606:TP63	KEGG-&-1&-I	TP63	Homo sapier Gene Symbol	TP63	FALSE	TP63
taxon:9606	163632	1902	vitamin K ep	gene	biological_pr	G:9606:VKORC1L1	KEGG-&-1&-I	VKORC1L1	Homo sapier Gene Symbol	VKORC1L1	FALSE	VKORC1L1
taxon:9606	180016	1902	interleukin e	gene	biological_pr	G:9606:ILF2		ILF2	Homo sapier Gene Symbol	ILF2	FALSE	ILF2
taxon:9606	180017	1902	forkhead box	gene	biological_pr	G:9606:FOXK2		FOXK2	Homo sapier Gene Symbol	FOXK2	FALSE	FOXK2
taxon:9606	180026	1902	interleukin 1	gene	biological_pr	G:9606:IL13	KEGG-&-1&-I	IL13	Homo sapier Gene Symbol	IL13	FALSE	IL13
taxon:9606	167739	1902	microtubule	gene	biological_pr	G:9606:MAP9		MAP9	Homo sapier Gene Symbol	MAP9	FALSE	MAP9
taxon:9606	175928	1902	USO1 vesicle	gene	biological_pr	G:9606:USO1		USO1	Homo sapier Gene Symbol	USO1	FALSE	USO1
taxon:9606	180024	1902	interleukin 1	gene	biological_pr	G:9606:IL13RA2	KEGG-&-1&-I	IL13RA2	Homo sapier Gene Symbol	IL13RA2	FALSE	IL13RA2
taxon:9606	175935	1902	RuvB like AA	gene	biological_pr	G:9606:RUVBL1	KEGG-&-1&-I	RUVBL1	Homo sapier Gene Symbol	RUVBL1	FALSE	RUVBL1
taxon:9606	167746	1902	rabaptin, RA	gene	biological_pr	G:9606:RABEP2		RABEP2	Homo sapier Gene Symbol	RABEP2	FALSE	RABEP2
taxon:9606	175939	1902	NOP14 nucle	gene	biological_pr	G:9606:NOP14		NOP14	Homo sapier Gene Symbol	NOP14	FALSE	NOP14
taxon:9606	163651	1902	membrane b	gene	biological_pr	G:9606:MBOAT1	KEGG-&-1&-I	MBOAT1	Homo sapier Gene Symbol	MBOAT1	FALSE	MBOAT1
taxon:9606	167751	1902	cleavage and	gene	biological_pr	G:9606:CPSF7	KEGG-&-1&-I	CPSF7	Homo sapier Gene Symbol	CPSF7	FALSE	CPSF7
taxon:9606	163653	1902	solute carrier	gene	biological_pr	G:9606:SLC2A12		SLC2A12	Homo sapier Gene Symbol	SLC2A12	FALSE	SLC2A12
taxon:9606	175941	1902	tumor necro	gene	biological_pr	G:9606:TNFSF11	KEGG-&-1&-I	TNFSF11	Homo sapier Gene Symbol	TNFSF11	FALSE	TNFSF11
taxon:9606	180041	1902	C-X-C motif	gene	biological_pr	G:9606:CXCL8	KEGG-&-1&-I	CXCL8	Homo sapier Gene Symbol	CXCL8	FALSE	CXCL8
taxon:9606	155470	1902	POU class 5	gene		G:9606:POU5F1P4		POU5F1P4	Homo sapier Gene Symbol	POU5F1P4	FALSE	POU5F1P4
taxon:9606	180045	1902	interleukin 6	gene	biological_pr	G:9606:IL6ST	KEGG-&-1&-I	IL6ST	Homo sapier Gene Symbol	IL6ST	FALSE	IL6ST
taxon:9606	175958	1902	protein activ	gene	biological_pr	G:9606:PRKRA		PRKRA	Homo sapier Gene Symbol	PRKRA	FALSE	PRKRA
taxon:9606	175962	1902	KH-type splic	gene	biological_pr	G:9606:KHSRP		KHSRP	Homo sapier Gene Symbol	KHSRP	FALSE	KHSRP
taxon:9606	167768	1902	PDZ domain c	gene	biological_pr	G:9606:PDZD3		PDZD3	Homo sapier Gene Symbol	PDZD3	FALSE	PDZD3
taxon:9606	180063	1902	inhibitor of n	gene	biological_pr	G:9606:IKBKB	KEGG-&-1&-I	IKBKB	Homo sapier Gene Symbol	IKBKB	FALSE	IKBKB
taxon:9606	175967	1902	tyrosyl-tRNA	gene	biological_pr	G:9606:YARS	KEGG-&-1&-I	YARS	Homo sapier Gene Symbol	YARS	FALSE	YARS
taxon:9606	167775	1902	zinc finger ar	gene	biological_pr	G:9606:ZBTB3		ZBTB3	Homo sapier Gene Symbol	ZBTB3	FALSE	ZBTB3
taxon:9606	180061	1902	interleukin 1	gene	biological_pr	G:9606:IL1B	KEGG-&-1&-I	IL1B	Homo sapier Gene Symbol	IL1B	FALSE	IL1B
taxon:9606	167776	1902	ATP/GTP bin	gene	biological_pr	G:9606:AGBL2		AGBL2	Homo sapier Gene Symbol	AGBL2	FALSE	AGBL2
taxon:9606	175969	1902	THO comple	gene	biological_pr	G:9606:THOC5	KEGG-&-1&-I	THOC5	Homo sapier Gene Symbol	THOC5	FALSE	THOC5
taxon:9606	175975	1902	cell division c	gene	biological_pr	G:9606:CDC14A	KEGG-&-1&-I	CDC14A	Homo sapier Gene Symbol	CDC14A	FALSE	CDC14A
taxon:9606	175978	1902	basic helix-lo	gene	biological_pr	G:9606:BHLHE40	KEGG-&-1&-I	BHLHE40	Homo sapier Gene Symbol	BHLHE40	FALSE	BHLHE40
taxon:9606	163691	1902	centrosomal	gene	biological_pr	G:9606:CEP120		CEP120	Homo sapier Gene Symbol	CEP120	FALSE	CEP120
taxon:9606	175976	1902	cell division c	gene	biological_pr	G:9606:CDC14B	KEGG-&-1&-I	CDC14B	Homo sapier Gene Symbol	CDC14B	FALSE	CDC14B
taxon:9606	175977	1902	protein inhib	gene	biological_pr	G:9606:PIAS1	KEGG-&-1&-I	PIAS1	Homo sapier Gene Symbol	PIAS1	FALSE	PIAS1
taxon:9606	175981	1902	mitogen-acti	gene	biological_pr	G:9606:MAPKAPK5	KEGG-&-1&-I	MAPKAPK5	Homo sapier Gene Symbol	MAPKAPK5	FALSE	MAPKAPK5
taxon:9606	163697	1902	DAB2 interac	gene	biological_pr	G:9606:DAB2IP	KEGG-&-1&-I	DAB2IP	Homo sapier Gene Symbol	DAB2IP	FALSE	DAB2IP
taxon:9606	167800	1902	euchromatic	gene	biological_pr	G:9606:EHMT1	KEGG-&-1&-I	EHMT1	Homo sapier Gene Symbol	EHMT1	FALSE	EHMT1
taxon:9606	175998	1902	COP9 signalo	gene	biological_pr	G:9606:COPS3		COPS3	Homo sapier Gene Symbol	COPS3	FALSE	COPS3
taxon:9606	175996	1902	chromobox 4	gene	biological_pr	G:9606:CBX4		CBX4	Homo sapier Gene Symbol	CBX4	FALSE	CBX4
taxon:9606	180099	1902	immunoglob	gene	biological_pr	G:9606:IGHG1		IGHG1	Homo sapier Gene Symbol	IGHG1	FALSE	IGHG1
taxon:9606	176000	1902	Y-box binding	gene	biological_pr	G:9606:YBX3	KEGG-&-1&-I	YBX3	Homo sapier Gene Symbol	YBX3	FALSE	YBX3
taxon:9606	176015	1902	potassium vo	gene	biological_pr	G:9606:KCNAB2		KCNAB2	Homo sapier Gene Symbol	KCNAB2	FALSE	KCNAB2
taxon:9606	176012	1902	inhibitor of n	gene	biological_pr	G:9606:IKBKG	KEGG-&-1&-I	IKBKG	Homo sapier Gene Symbol	IKBKG	FALSE	IKBKG
taxon:9606	180109	1902	insulin like g	gene	biological_pr	G:9606:IGFBP7		IGFBP7	Homo sapier Gene Symbol	IGFBP7	FALSE	IGFBP7
taxon:9606	180113	1902	insulin like g	gene	biological_pr	G:9606:IGFBP3	KEGG-&-1&-I	IGFBP3	Homo sapier Gene Symbol	IGFBP3	FALSE	IGFBP3
taxon:9606	176022	1902	histone clust	gene	biological_pr	G:9606:HIST1H4F	KEGG-&-1&-I	HIST1H4F	Homo sapier Gene Symbol	HIST1H4F	FALSE	HIST1H4F
taxon:9606	180119	1902	insulin like g	gene	biological_pr	G:9606:IGF1R	KEGG-&-1&-I	IGF1R	Homo sapier Gene Symbol	IGF1R	FALSE	IGF1R
taxon:9606	176023	1902	histone clust	gene	biological_pr	G:9606:HIST1H4D	KEGG-&-1&-I	HIST1H4D	Homo sapier Gene Symbol	HIST1H4D	FALSE	HIST1H4D
taxon:9606	176020	1902	histone clust	gene	biological_pr	G:9606:HIST1H4J	KEGG-&-1&-I	HIST1H4J	Homo sapier Gene Symbol	HIST1H4J	FALSE	HIST1H4J
taxon:9606	176021	1902	histone clust	gene	biological_pr	G:9606:HIST1H4K	KEGG-&-1&-I	HIST1H4K	Homo sapier Gene Symbol	HIST1H4K	FALSE	HIST1H4K
taxon:9606	176026	1902	histone clust	gene	biological_pr	G:9606:HIST1H3H	KEGG-&-1&-I	HIST1H3H	Homo sapier Gene Symbol	HIST1H3H	FALSE	HIST1H3H
taxon:9606	176027	1902	histone clust	gene	biological_pr	G:9606:HIST1H3J	KEGG-&-1&-I	HIST1H3J	Homo sapier Gene Symbol	HIST1H3J	FALSE	HIST1H3J
taxon:9606	176024	1902	histone clust	gene	biological_pr	G:9606:HIST1H4A	KEGG-&-1&-I	HIST1H4A	Homo sapier Gene Symbol	HIST1H4A	FALSE	HIST1H4A
taxon:9606	176025	1902	histone clust	gene	biological_pr	G:9606:HIST1H3B	KEGG-&-1&-I	HIST1H3B	Homo sapier Gene Symbol	HIST1H3B	FALSE	HIST1H3B
taxon:9606	176030	1902	histone clust	gene	biological_pr	G:9606:HIST1H3E	KEGG-&-1&-I	HIST1H3E	Homo sapier Gene Symbol	HIST1H3E	FALSE	HIST1H3E
taxon:9606	167839	1902	zinc finger pr	gene	biological_pr	G:9606:ZNF668		ZNF668	Homo sapier Gene Symbol	ZNF668	FALSE	ZNF668
taxon:9606	176031	1902	histone clust	gene	biological_pr	G:9606:HIST1H3C	KEGG-&-1&-I	HIST1H3C	Homo sapier Gene Symbol	HIST1H3C	FALSE	HIST1H3C
taxon:9606	176028	1902	histone clust	gene	biological_pr	G:9606:HIST1H3G	KEGG-&-1&-I	HIST1H3G	Homo sapier Gene Symbol	HIST1H3G	FALSE	HIST1H3G
taxon:9606	176029	1902	histone clust	gene	biological_pr	G:9606:HIST1H3I	KEGG-&-1&-I	HIST1H3I	Homo sapier Gene Symbol	HIST1H3I	FALSE	HIST1H3I
taxon:9606	180131	1902	interferon ga	gene	biological_pr	G:9606:IFNG	KEGG-&-1&-I	IFNG	Homo sapier Gene Symbol	IFNG	FALSE	IFNG
taxon:9606	176032	1902	histone clust	gene	biological_pr	G:9606:HIST1H3D	KEGG-&-1&-I	HIST1H3D	Homo sapier Gene Symbol	HIST1H3D	FALSE	HIST1H3D
taxon:9606	176033	1902	histone clust	gene	biological_pr	G:9606:HIST1H3A	KEGG-&-1&-I	HIST1H3A	Homo sapier Gene Symbol	HIST1H3A	FALSE	HIST1H3A
taxon:9606	180129	1902	interferon ga	gene	biological_pr	G:9606:IFNGR2	KEGG-&-1&-I	IFNGR2	Homo sapier Gene Symbol	IFNGR2	FALSE	IFNGR2
taxon:9606	176039	1902	histone clust	gene	biological_pr	G:9606:HIST1H2BE	KEGG-&-1&-I	HIST1H2BE	Homo sapier Gene Symbol	HIST1H2BE	FALSE	HIST1H2BE
taxon:9606	176036	1902	histone clust	gene	biological_pr	G:9606:HIST1H2BC	KEGG-&-1&-I	HIST1H2BC	Homo sapier Gene Symbol	HIST1H2BC	FALSE	HIST1H2BC
taxon:9606	176037	1902	histone clust	gene	biological_pr	G:9606:HIST1H2BI	KEGG-&-1&-I	HIST1H2BI	Homo sapier Gene Symbol	HIST1H2BI	FALSE	HIST1H2BI
taxon:9606	176040	1902	histone clust	gene	biological_pr	G:9606:HIST1H2BF	KEGG-&-1&-I	HIST1H2BF	Homo sapier Gene Symbol	HIST1H2BF	FALSE	HIST1H2BF
taxon:9606	176046	1902	histone clust	gene	biological_pr	G:9606:HIST2H2AA3	KEGG-&-1&-I	HIST2H2AA3	Homo sapier Gene Symbol	HIST2H2AA3	FALSE	HIST2H2AA3
taxon:9606	176044	1902	histone clust	gene	biological_pr	G:9606:HIST1H2BG	KEGG-&-1&-I	HIST1H2BG	Homo sapier Gene Symbol	HIST1H2BG	FALSE	HIST1H2BG
taxon:9606	167859	1902	potassium ch	gene	biological_pr	G:9606:KCTD17		KCTD17	Homo sapier Gene Symbol	KCTD17	FALSE	KCTD17
taxon:9606	176053	1902	metastasis a	gene	biological_pr	G:9606:MTA1		MTA1	Homo sapier Gene Symbol	MTA1	FALSE	MTA1
taxon:9606	176062	1902	ubiquitin spe	gene	biological_pr	G:9606:USP2		USP2	Homo sapier Gene Symbol	USP2	FALSE	USP2
taxon:9606	180156	1902	interferon ga	gene	biological_pr	G:9606:IFI16	KEGG-&-1&-I	IFI16	Homo sapier Gene Symbol	IFI16	FALSE	IFI16
taxon:9606	176061	1902	ubiquitin spe	gene	biological_pr	G:9606:USP10		USP10	Homo sapier Gene Symbol	USP10	FALSE	USP10
taxon:9606	180163	1902	isocitrate de	gene	biological_pr	G:9606:IDH3B	KEGG-&-1&-I	IDH3B	Homo sapier Gene Symbol	IDH3B	FALSE	IDH3B
taxon:9606	167879	1902	HECT domai	gene	biological_pr	G:9606:HECTD3		HECTD3	Homo sapier Gene Symbol	HECTD3	FALSE	HECTD3
taxon:9606	180167	1902	insulin degra	gene	biological_pr	G:9606:IDE	KEGG-&-1&-I	IDE	Homo sapier Gene Symbol	IDE	FALSE	IDE
taxon:9606	176069	1902	squamous ce	gene	biological_pr	G:9606:SART1	KEGG-&-1&-I	SART1	Homo sapier Gene Symbol	SART1	FALSE	SART1
taxon:9606	159691	1902	notch 2 N-te	gene	biological_pr	G:9606:NOTCH2NL		NOTCH2NL	Homo sapier Gene Symbol	NOTCH2NL	FALSE	NOTCH2NL
taxon:9606	176082	1902	claudin 1	gene	biological_pr	G:9606:CLDN1	KEGG-&-1&-I	CLDN1	Homo sapier Gene Symbol	CLDN1	FALSE	CLDN1
taxon:9606	176087	1902	ASH2 like his	gene	biological_pr	G:9606:ASH2L	KEGG-&-1&-I	ASH2L	Homo sapier Gene Symbol	ASH2L	FALSE	ASH2L
taxon:9606	171988	1902	vacuolar prot	gene	biological_pr	G:9606:VPS4A	KEGG-&-1&-I	VPS4A	Homo sapier Gene Symbol	VPS4A	FALSE	VPS4A
taxon:9606	167899	1902	inositol-trisp	gene	biological_pr	G:9606:ITPKC	KEGG-&-1&-I	ITPKC	Homo sapier Gene Symbol	ITPKC	FALSE	ITPKC
taxon:9606	180187	1902	homeobox B	gene	biological_pr	G:9606:HOXB5		HOXB5	Homo sapier Gene Symbol	HOXB5	FALSE	HOXB5
taxon:9606	180185	1902	homeobox B	gene	biological_pr	G:9606:HOXB7		HOXB7	Homo sapier Gene Symbol	HOXB7	FALSE	HOXB7
taxon:9606	180191	1902	homeobox B	gene	biological_pr	G:9606:HOXB1	KEGG-&-1&-I	HOXB1	Homo sapier Gene Symbol	HOXB1	FALSE	HOXB1
taxon:9606	176092	1902	protein inhib	gene	biological_pr	G:9606:PIAS2	KEGG-&-1&-I	PIAS2	Homo sapier Gene Symbol	PIAS2	FALSE	PIAS2
taxon:9606	180194	1902	hippocalcin	gene	biological_pr	G:9606:HPCA		HPCA	Homo sapier Gene Symbol	HPCA	FALSE	HPCA
taxon:9606	172001	1902	argonaute 2,	gene	biological_pr	G:9606:AGO2		AGO2	Homo sapier Gene Symbol	AGO2	FALSE	AGO2
taxon:9606	167910	1902	elongation fa	gene	biological_pr	G:9606:ELL3		ELL3	Homo sapier Gene Symbol	ELL3	FALSE	ELL3
taxon:9606	176106	1902	SH2 domain c	gene	biological_pr	G:9606:SH2D2A	KEGG-&-1&-I	SH2D2A	Homo sapier Gene Symbol	SH2D2A	FALSE	SH2D2A
taxon:9606	180210	1902	heterogeneo	gene	biological_pr	G:9606:HNRNPL		HNRNPL	Homo sapier Gene Symbol	HNRNPL	FALSE	HNRNPL
taxon:9606	176114	1902	huntingtin in	gene	biological_pr	G:9606:HIP1R		HIP1R	Homo sapier Gene Symbol	HIP1R	FALSE	HIP1R
taxon:9606	172019	1902	XIAP associat	gene	biological_pr	G:9606:XAF1		XAF1	Homo sapier Gene Symbol	XAF1	FALSE	XAF1
taxon:9606	176115	1902	ring finger pr	gene	biological_pr	G:9606:RNF8		RNF8	Homo sapier Gene Symbol	RNF8	FALSE	RNF8
taxon:9606	180209	1902	heterogeneo	gene	biological_pr	G:9606:HNRNPU	KEGG-&-1&-I	HNRNPU	Homo sapier Gene Symbol	HNRNPU	FALSE	HNRNPU
taxon:9606	172017	1902	tight junctior	gene	cellular_com	G:9606:TJP3	KEGG-&-1&-I	TJP3	Homo sapier Gene Symbol	TJP3	FALSE	TJP3
taxon:9606	167927	1902	TM2 domain	gene	cellular_com	G:9606:TM2D3		TM2D3	Homo sapier Gene Symbol	TM2D3	FALSE	TM2D3
taxon:9606	176119	1902	suppressor o	gene	biological_pr	G:9606:SOCS3	KEGG-&-1&-I	SOCS3	Homo sapier Gene Symbol	SOCS3	FALSE	SOCS3
taxon:9606	180213	1902	inter-alpha-t	gene	biological_pr	G:9606:ITIH2		ITIH2	Homo sapier Gene Symbol	ITIH2	FALSE	ITIH2
taxon:9606	172021	1902	M-phase pho	gene	biological_pr	G:9606:MPHOSPH8		MPHOSPH8	Homo sapier Gene Symbol	MPHOSPH8	FALSE	MPHOSPH8
taxon:9606	176123	1902	TATA-box bir	gene	biological_pr	G:9606:TAF1A		TAF1A	Homo sapier Gene Symbol	TAF1A	FALSE	TAF1A
taxon:9606	180219	1902	eukaryotic tr	gene	biological_pr	G:9606:EIF6	KEGG-&-1&-I	EIF6	Homo sapier Gene Symbol	EIF6	FALSE	EIF6
taxon:9606	172024	1902	transmembr	gene	biological_pr	G:9606:TMED9		TMED9	Homo sapier Gene Symbol	TMED9	FALSE	TMED9
taxon:9606	180216	1902	integrin subu	gene	biological_pr	G:9606:ITGB7	KEGG-&-1&-I	ITGB7	Homo sapier Gene Symbol	ITGB7	FALSE	ITGB7
taxon:9606	167934	1902	chromodoma	gene	biological_pr	G:9606:CHD9		CHD9	Homo sapier Gene Symbol	CHD9	FALSE	CHD9
taxon:9606	167935	1902	F-box protei	gene	biological_pr	G:9606:FBXO11		FBXO11	Homo sapier Gene Symbol	FBXO11	FALSE	FBXO11
taxon:9606	180223	1902	integrin subu	gene	biological_pr	G:9606:ITGB1	KEGG-&-1&-I	ITGB1	Homo sapier Gene Symbol	ITGB1	FALSE	ITGB1
taxon:9606	176124	1902	TATA-box bir	gene	biological_pr	G:9606:TAF1B		TAF1B	Homo sapier Gene Symbol	TAF1B	FALSE	TAF1B
taxon:9606	176125	1902	TATA-box bir	gene	biological_pr	G:9606:TAF1C		TAF1C	Homo sapier Gene Symbol	TAF1C	FALSE	TAF1C
