# Supplementary material for: Network Pharmacology Approach for Predicting Targets of Zishen Yutai Pills on Premature Ovarian Insufficiency
Source: Evid Based Complement Alternat Med. 2021 Aug 4;2021:8215454. doi: 10.1155/2021/8215454 (PMC8357500; doi:10.1155/2021/8215454)
Supplement: Supplementary Materials — Supplementary Table 1: active compounds of each herb in ZSYTP. Supplementary Table 2: corresponding targets of active compounds: (A) two target genes of SDH; (B) 138 target genes of GQZ; (C) 202 target genes of TSZ; (D) 25 target genes of BJT; (E) 121 target genes of DS; (F) 11 target genes of BZ; (G) 283 target genes of DZ; (H) 13 target genes of SR; (J) 112 target genes of RS; (K) 76 target genes of SJS; (L) 115 target genes of AY; (M) 149 target genes of HSW; (N) 68 target genes of EJ; (P) 46 target genes of LJJ. Supplementary Table 3: POI targets. Supplementary Table 4: compounds and targets attributes of C-T network. Supplementary Table 5: data of compound-target network. Supplementary Table 6: analysed C-T network: (A) active compounds of C-T network and corresponding degrees; (B) targets of C-T network and corresponding degrees. Supplementary Table 7: ZSYTP core PPI network. Supplementary Table 8: gene ontology and KEGG analysis: (A) GO-BP analysis; (B) GO-MF analysis; (C) GO-CC analysis; (D) KEGG pathway enrichment analysis. [file 8215454.f1.zip › 8215454.f1/ST8C GO-CC.pdf]

Table S8C Gene Ontology (GO) Cellular Component Analysis				
GO ID	GO Term	Term P value	No. of Genes	Associated Genes Found
GO:1903561	extracellular vesicle	3.498E-30	97	[ACTB, ACTN1, ACTN4, ALDOA, ALYREF, ATP1A1, ATP5F1A, ATP5F1B, BCR, CCT2, CCT3, CCT4, CCT5, CCT7, CCT8, CFL1, CLTC, CSE1L, CUL4B, DDX5, DNAJB1, E2F1, EEF1A1, EEF1G, EEF2, ENO1, FBL, FLNA, FLNB, GAPDH, H2AC20, H2AX, H2BC21, H4-16, H4C1, H4C11, H4C12, H4C13, H4C14, H4C15, H4C2, H4C3, H4C4, H4C5, H4C6, H4C8, H4C9, HNRNPK, HNRNPM, HSPA6, HSPD1, IQGAP1, JUP, KRT18, LDHA, LDHB, LGALS3BP, MACROH2A1, NCL, NCOA3, P4HB, PDCD6, PHB, PHGDH, PKM, PPIA, PRDX1, PRKCB, PSMA5, PSMA7, RAN, RBMX, RPL10A, RPL11, RPL14, RPL23, RPL5, RPLP0, RPS16, RPS19, RPS2, RPS20, RPS3, RPS4X, RPS8, RPSA, SLC25A3, SPTBN1, SSBP1, TCP1, TLN1, TPI1, TPM3, TUBA1A, TUBA4A, TUBB4B, UBA1]
GO:0005681	spliceosomal complex	3.717E-24	30	[ALYREF, DDX5, DHX15, EFTUD2, HNRNPF, HNRNPH1, HNRNPK, HNRNPM, HNRNPR, NCL, PRPF19, PRPF40A, PRPF8, RBMX, SF1, SF3A1, SF3B1, SF3B2, SF3B3, SNRNP200, SNRNP70, SNRPA1, SNRPB, SRRM1, SRSF1, SRSF2, SYNCRIP, TRA2A, U2AF2, UPF1]
GO:0071013	catalytic step 2 spliceosome	1.157E-21	21	[ALYREF, DDX5, EFTUD2, HNRNPF, HNRNPH1, HNRNPK, HNRNPM, HNRNPR, PRPF19, PRPF8, RBMX, SF3A1, SF3B1, SF3B2, SF3B3, SNRNP200, SNRPA1, SNRPB, SRRM1, SRSF1, SYNCRIP]
GO:0000784	nuclear chromosome, telomeric region	7.341E-20	23	[ALYREF, ATR, H4-16, H4C1, H4C11, H4C12, H4C13, H4C14, H4C15, H4C2, H4C3, H4C4, H4C5, H4C6, H4C8, H4C9, MACROH2A1, MRE11, MSH2, PRKDC, TP53BP1, UPF1, XRCC5]
GO:0000781	chromosome, telomeric region	1.222E-19	25	[ALYREF, ATR, CHEK1, H2AX, H4-16, H4C1, H4C11, H4C12, H4C13, H4C14, H4C15, H4C2, H4C3, H4C4, H4C5, H4C6, H4C8, H4C9, MACROH2A1, MRE11, MSH2, PRKDC, TP53BP1, UPF1, XRCC5]
GO:0022626	cytosolic ribosome	8.657E-19	21	[RPL10A, RPL11, RPL13, RPL14, RPL23, RPL5, RPL6, RPL7, RPL8, RPL9, RPLP0, RPS16, RPS19, RPS2, RPS20, RPS21, RPS3, RPS4X, RPS6, RPS8, RPSA]
GO:0098687	chromosomal region	5.761E-18	32	[ALYREF, ATR, AURKB, CEBPB, CHEK1, CSNK1A1, DAXX, H2AX, H4-16, H4C1, H4C11, H4C12, H4C13, H4C14, H4C15, H4C2, H4C3, H4C4, H4C5, H4C6, H4C8, H4C9, MACROH2A1, MRE11, MSH2, PRKDC, RAD21, SMC1A, SMC3, TP53BP1, UPF1, XRCC5]
GO:0000788	nuclear nucleosome	2.151E-17	14	[H4-16, H4C1, H4C11, H4C12, H4C13, H4C14, H4C15, H4C2, H4C3, H4C4, H4C5, H4C6, H4C8, H4C9]
GO:0000228	nuclear chromosome	1.250E-16	57	[ACTB, ALYREF, ATR, AURKB, CCAR2, CEBPB, CHD4, CHEK1, E2F1, H2AC20, H2AX, H4-16, H4C1, H4C11, H4C12, H4C13, H4C14, H4C15, H4C2, H4C3, H4C4, H4C5, H4C6, H4C8, H4C9, HNRNPD, HNRNPK, KAT2A, MACROH2A1, MRE11, MSH2, MTA2, NCOA3, NCOR1, NCOR2, PRKDC, PRPF19, RAD21, RBBP4, RBBP7, RBMX, SF3B3, SMARCA4, SMARCA5, SMARCB1, SMARCC1, SMARCC2, SMC1A, SMC3, TARDBP, TCP1, TOP1, TP53BP1, TUBG1, UPF1, XRCC5, YY1]
GO:0005925	focal adhesion	6.677E-16	32	[ACTB, ACTN1, ACTN4, CFL1, CLTC, CTNNA1, DAB2, FLNA, FLNB, HNRNPK, IQGAP1, JAK1, JUP, LIMA1, P4HB, PAK1, PPIA, RPL10A, RPL23, RPL5, RPL6, RPL7, RPL8, RPL9, RPLP0, RPS16, RPS19, RPS2, RPS3, RPS4X, RPS8, TLN1]
GO:0000786	nucleosome	1.580E-15	18	[H2AC20, H2AX, H2BC21, H4-16, H4C1, H4C11, H4C12, H4C13, H4C14, H4C15, H4C2, H4C3, H4C4, H4C5, H4C6, H4C8, H4C9, MACROH2A1]
GO:0016604	nuclear body	3.872E-14	41	[ACTN4, ALYREF, ATR, CRY2, CSNK1A1, DAXX, DDX1, DDX17, DHX15, DYRK1A, EFTUD2, FBL, H2AX, HDAC5, HNRNPM, INPPL1, MRE11, NCOR2, NONO, NXF1, PRPF19, PRPF40A, PRPF8, RBM39, SF1, SF3A1, SF3B1, SF3B2, SF3B3, SFPQ, SKI, SNRNP70, SNRPA1, SRRM1, SRSF1, SRSF2, SRSF3, TARDBP, TP53BP1, U2AF2, UBXN7]
GO:0044391	ribosomal subunit	4.611E-14	21	[RPL10A, RPL11, RPL13, RPL14, RPL23, RPL5, RPL6, RPL7, RPL8, RPL9, RPLP0, RPS16, RPS19, RPS2, RPS20, RPS21, RPS3, RPS4X, RPS6, RPS8, RPSA]
GO:0005840	ribosome	5.536E-14	23	[EEF2, RPL10A, RPL11, RPL13, RPL14, RPL23, RPL5, RPL6, RPL7, RPL8, RPL9, RPLP0, RPS16, RPS19, RPS2, RPS20, RPS21, RPS3, RPS4X, RPS6, RPS8, RPSA, SF1]
GO:0016607	nuclear speck	1.114E-13	29	[ALYREF, CRY2, CSNK1A1, DDX17, DHX15, DYRK1A, EFTUD2, H2AX, HDAC5, INPPL1, NONO, NXF1, PRPF19, PRPF40A, PRPF8, RBM39, SF3A1, SF3B1, SF3B2, SF3B3, SFPQ, SNRNP70, SNRPA1, SRRM1, SRSF1, SRSF2, SRSF3, TARDBP, U2AF2]
GO:0005730	nucleolus	4.333E-12	41	[C1QBP, CDKN2A, DAB2, DAXX, DDX17, DDX24, DDX5, DHX15, DNAJB1, EEF1A1, FBL, FLNA, HNRNPM, ILF2, ILF3, KRT18, MACROH2A1, NCL, NONO, NOP2, NUP153, PRKDC, RAN, RPL11, RPL13, RPL23, RPL5, RPL7, RPS19, RPS3, RPS6, SENP3, SF3B3, SKP2, SMARCA4, SMARCA5, SMARCB1, SPTBN1, TOP1, TRA2A, XRCC5]
GO:0005684	U2-type spliceosomal complex	1.396E-11	14	[DHX15, EFTUD2, PRPF19, PRPF40A, PRPF8, SF3A1, SF3B1, SF3B2, SF3B3, SNRNP200, SNRNP70, SNRPA1, SNRPB, U2AF2]
GO:0070161	anchoring junction	7.307E-11	37	[ACTB, ACTN1, ACTN4, ATP1A1, CCND1, CFL1, CLTC, CTNNA1, DAB2, FLNA, FLNB, HNRNPK, ILF3, IQGAP1, JAK1, JUP, KRT18, LIMA1, P4HB, PAK1, PPIA, RPL10A, RPL23, RPL5, RPL6, RPL7, RPL8, RPL9, RPLP0, RPS16, RPS19, RPS2, RPS3, RPS4X, RPS8, TLN1, UBA1]
GO:0043657	host cell	1.048E-10	20	[CCND1, H4-16, H4C1, H4C11, H4C12, H4C13, H4C14, H4C15, H4C2, H4C3, H4C4, H4C5, H4C6, H4C8, H4C9, KPNA1, NUP153, NXF1, RAN, RANBP2]
GO:0022625	cytosolic large ribosomal subunit	2.262E-10	11	[RPL10A, RPL11, RPL13, RPL14, RPL23, RPL5, RPL6, RPL7, RPL8, RPL9, RPLP0]
GO:0042025	host cell nucleus	4.039E-10	17	[CCND1, H4-16, H4C1, H4C11, H4C12, H4C13, H4C14, H4C15, H4C2, H4C3, H4C4, H4C5, H4C6, H4C8, H4C9, KPNA1, NXF1]
GO:0022627	cytosolic small ribosomal subunit	6.598E-10	10	[RPS16, RPS19, RPS2, RPS20, RPS21, RPS3, RPS4X, RPS6, RPS8, RPSA]
GO:0034399	nuclear periphery	2.037E-09	14	[CEBPB, CFL1, HNRNPM, MATR3, NCOR2, NONO, NUP153, PRPF40A, RAD21, SFPQ, SMC1A, SMC3, SRRM1, YY1]
GO:0097525	spliceosomal snRNP complex	6.493E-09	10	[EFTUD2, PRPF40A, PRPF8, SF3A1, SF3B1, SF3B2, SNRNP200, SNRNP70, SNRPA1, SNRPB]
GO:0071005	U2-type precatalytic spliceosome	1.075E-08	9	[EFTUD2, PRPF8, SF3A1, SF3B1, SF3B2, SF3B3, SNRNP200, SNRPA1, SNRPB]
GO:0000118	histone deacetylase complex	4.496E-08	10	[CHD4, HDAC3, HDAC5, HDAC6, MTA2, NCOR1, NCOR2, RBBP4, RBBP7, SF3B3]
GO:0071004	U2-type prespliceosome	1.092E-07	6	[PRPF40A, SF3A1, SF3B1, SNRNP70, SNRPB, U2AF2]
GO:0017053	transcription repressor complex	1.425E-07	10	[CCND1, CHD4, HDAC3, HDGF, MTA2, NCOR1, NCOR2, RBBP4, RBBP7, SKI]
GO:0070603	SWI/SNF superfamily-type complex	1.425E-07	10	[CHD4, MTA2, RBBP4, RBBP7, SMARCA4, SMARCA5, SMARCB1, SMARCC1, SMARCC2, YY1]
GO:0005874	microtubule	7.326E-07	20	[AURKB, CCT2, CCT3, CCT4, CCT5, CCT7, CCT8, CDC27, CLTC, EMD, HDAC6, IQGAP1, TCP1, TUBA1A, TUBA1B, TUBA1C, TUBA4A, TUBB4B, TUBG1, TUBGCP3]
GO:0005844	polysome	1.012E-06	9	[EEF2, FUS, RPL10A, RPL11, RPL6, RPL8, RPS21, RPS3, RPS4X]
GO:0036464	cytoplasmic ribonucleoprotein granule	1.352E-06	14	[ACTB, DDX1, HNRNPK, IQGAP1, NCL, NXF1, RPL6, RPLP0, RPS4X, RPS6, TARDBP, TOP1, TUBA1A, UPF1]
GO:0042788	polysomal ribosome	8.800E-06	6	[EEF2, RPL10A, RPL11, RPL6, RPL8, RPS21]
GO:1904813	ficolin-1-rich granule lumen	9.312E-06	10	[ALDOA, CCT8, EEF1A1, EEF2, HSPA6, ILF2, JUP, PKM, PPIA, PSMA5]
GO:0005686	U2 snRNP	9.486E-06	5	[SF3A1, SF3B1, SF3B2, SNRPA1, SNRPB]
GO:0071564	npBAF complex	1.023E-05	4	[SMARCA4, SMARCB1, SMARCC1, SMARCC2]
GO:0034774	secretory granule lumen	1.096E-05	16	[ACTN1, ACTN4, ALDOA, CCT2, CCT8, EEF1A1, EEF2, HSPA6, ILF2, JUP, LGALS3BP, PKM, PPIA, PSMA5, TUBB4B, XRCC5]
GO:0005689	U12-type spliceosomal complex	1.439E-05	5	[DHX15, SF3B1, SF3B2, SF3B3, SNRPB]
GO:0071565	nBAF complex	2.741E-05	4	[SMARCA4, SMARCB1, SMARCC1, SMARCC2]
GO:0071007	U2-type catalytic step 2 spliceosome	2.995E-05	5	[EFTUD2, PRPF19, PRPF8, SNRPA1, SNRPB]
GO:0045261	proton-transporting ATP synthase complex, catalytic core F(1)	3.579E-05	3	[ATP5F1A, ATP5F1B, ATP5F1C]
GO:0000793	condensed chromosome	3.931E-05	12	[AURKB, CEBPB, CHEK1, CSNK1A1, H2AX, MACROH2A1, RAD21, SMARCA5, SMC1A, SMC3, TP53BP1, TUBG1]
GO:0000793	condensed chromosome	3.931E-05	12	[AURKB, CEBPB, CHEK1, CSNK1A1, H2AX, MACROH2A1, RAD21, SMARCA5, SMC1A, SMC3, TP53BP1, TUBG1]
GO:0005657	replication fork	5.329E-05	7	[CHEK1, H2AX, MRE11, PRPF19, SMARCA5, TOP1, TP53BP1]
GO:0042382	paraspeckles	6.205E-05	3	[HNRNPM, NONO, SFPQ]
GO:0030893	meiotic cohesin complex	6.205E-05	3	[RAD21, SMC1A, SMC3]
GO:0030132	clathrin coat of coated pit	7.487E-05	4	[AP2B1, CLTC, EPS15, PICALM]
GO:0016514	SWI/SNF complex	1.134E-04	4	[SMARCA4, SMARCB1, SMARCC1, SMARCC2]
GO:0016581	NuRD complex	1.373E-04	4	[CHD4, MTA2, RBBP4, RBBP7]
GO:0000794	condensed nuclear chromosome	3.585E-04	7	[AURKB, CHEK1, H2AX, RAD21, SMC1A, SMC3, TUBG1]
GO:0034709	methylosome	3.727E-04	3	[PRMT5, SNRPB, WDR77]
GO:0030017	sarcomere	4.113E-04	10	[ACTN1, ACTN4, ALDOA, ENO1, FLNA, FLNB, JUP, PAK1, SPTBN1, TPM3]
GO:0034708	methyltransferase complex	4.241E-04	7	[HCFC1, PRMT5, RBBP4, RBBP7, SENP3, SNRPB, WDR77]
GO:0071006	U2-type catalytic step 1 spliceosome	4.801E-04	3	[PRPF19, PRPF8, SNRNP200]
GO:0042470	melanosome	4.990E-04	7	[ATP1A1, CCT4, CLTC, P4HB, PRDX1, RAN, RPN1]
GO:0000803	sex chromosome	5.413E-04	4	[H2AX, MACROH2A1, SMARCB1, SMARCC1]
GO:0030120	vesicle coat	6.719E-04	5	[AP2B1, CLTC, EPS15, PDCD6, PICALM]
GO:0046540	U4/U6 x U5 tri-snRNP complex	6.905E-04	4	[EFTUD2, PRPF8, SNRNP200, SNRPB]
GO:0030863	cortical cytoskeleton	7.510E-04	7	[ACTB, ACTN4, CFL1, EEF1A1, FLNA, PRKCB, SPTBN1]
GO:0014704	intercalated disc	7.876E-04	5	[ACTN1, ATP1A1, CTNNA1, JUP, PAK1]
GO:0005876	spindle microtubule	8.506E-04	5	[AURKB, CDC27, CLTC, TUBG1, TUBGCP3]
GO:0000775	chromosome, centromeric region	1.052E-03	9	[AURKB, CEBPB, CSNK1A1, DAXX, MACROH2A1, RAD21, SMC1A, SMC3, TP53BP1]
GO:0005682	U5 snRNP	1.101E-03	3	[PRPF8, SNRNP200, SNRPB]
GO:0019005	SCF ubiquitin ligase complex	1.141E-03	5	[CUL4A, CUL4B, RBX1, SKP1, SKP2]
GO:0030669	clathrin-coated endocytic vesicle membrane	1.892E-03	4	[AP2B1, CLTC, EPS15, PICALM]
GO:0070822	Sin3-type complex	2.076E-03	3	[NCOR1, RBBP4, SF3B3]
GO:0001725	stress fiber	2.184E-03	5	[ACTN1, ACTN4, FLNB, LIMA1, TPM3]
GO:0005753	mitochondrial proton-transporting ATP synthase complex	2.382E-03	3	[ATP5F1A, ATP5F1B, ATP5F1C]
GO:0005685	U1 snRNP	2.382E-03	3	[PRPF40A, SNRNP70, SNRPB]
GO:0031674	I band	3.005E-03	7	[ACTN1, ACTN4, ALDOA, FLNA, FLNB, JUP, PAK1]
GO:0001726	ruffle	3.306E-03	8	[ACTN1, CFL1, EEF1A1, IQGAP1, LIMA1, PAK1, RPS3, TLN1]
GO:0005881	cytoplasmic microtubule	3.417E-03	5	[HDAC6, TUBA1A, TUBA1B, TUBA1C, TUBG1]
GO:0035097	histone methyltransferase complex	3.417E-03	5	[HCFC1, PRMT5, RBBP4, RBBP7, SENP3]
GO:0031430	M band	3.879E-03	3	[ALDOA, ENO1, SPTBN1]
GO:0031461	cullin-RING ubiquitin ligase complex	4.538E-03	7	[CDC27, CUL4A, CUL4B, PDCD6, RBX1, SKP1, SKP2]
GO:0090734	site of DNA damage	5.330E-03	5	[H2AX, MRE11, PRPF19, TP53BP1, XRCC5]
GO:0030864	cortical actin cytoskeleton	2.419E-02	4	[CFL1, EEF1A1, PRKCB, SPTBN1]
