# Supplementary material for: Network Pharmacology Approach for Predicting Targets of Zishen Yutai Pills on Premature Ovarian Insufficiency
Source: Evid Based Complement Alternat Med. 2021 Aug 4;2021:8215454. doi: 10.1155/2021/8215454 (PMC8357500; doi:10.1155/2021/8215454)
Supplement: Supplementary Materials — Supplementary Table 1: active compounds of each herb in ZSYTP. Supplementary Table 2: corresponding targets of active compounds: (A) two target genes of SDH; (B) 138 target genes of GQZ; (C) 202 target genes of TSZ; (D) 25 target genes of BJT; (E) 121 target genes of DS; (F) 11 target genes of BZ; (G) 283 target genes of DZ; (H) 13 target genes of SR; (J) 112 target genes of RS; (K) 76 target genes of SJS; (L) 115 target genes of AY; (M) 149 target genes of HSW; (N) 68 target genes of EJ; (P) 46 target genes of LJJ. Supplementary Table 3: POI targets. Supplementary Table 4: compounds and targets attributes of C-T network. Supplementary Table 5: data of compound-target network. Supplementary Table 6: analysed C-T network: (A) active compounds of C-T network and corresponding degrees; (B) targets of C-T network and corresponding degrees. Supplementary Table 7: ZSYTP core PPI network. Supplementary Table 8: gene ontology and KEGG analysis: (A) GO-BP analysis; (B) GO-MF analysis; (C) GO-CC analysis; (D) KEGG pathway enrichment analysis. [file 8215454.f1.zip › 8215454.f1/ST8D KEGG.pdf]

Table S8D Kyoto Encyclopedia of Genes and Genomes (KEGG) Pathway analysis				
**GO ID**	**GO Term**	**Term P value**	**No. of Genes**	**Associated Genes Found**
KEGG:05203	Viral carcinogenesis	6.19E-17	30	[ACTN1, ACTN4, CCND1, CDKN2A, CHD4, CHEK1, H2BC21, H4-16, H4C1, H4C11, H4C12, H4C13, H4C14, H4C15, H4C2, H4C3, H4C4, H4C5, H4C6, H4C8, H4C9, HDAC3, HDAC5, HDAC6, HNRNPK, JAK1, KAT2A, PIK3R2, PKM, SKP2]
KEGG:03040	Spliceosome	1.28E-15	25	[ALYREF, DDX5, DHX15, EFTUD2, HNRNPK, HNRNPM, HSPA6, NCBP1, PRPF19, PRPF40A, PRPF8, RBMX, SF3A1, SF3B1, SF3B2, SF3B3, SNRNP200, SNRNP70, SNRPA1, SNRPB, SRSF1, SRSF2, SRSF3, TRA2A, U2AF2]
KEGG:05322	Systemic lupus erythematosus	1.53E-12	21	[ACTN1, ACTN4, H2AC20, H2AX, H2BC21, H4-16, H4C1, H4C11, H4C12, H4C13, H4C14, H4C15, H4C2, H4C3, H4C4, H4C5, H4C6, H4C8, H4C9, MACROH2A1, SNRPB]
KEGG:03010	Ribosome	2.98E-11	21	[RPL10A, RPL11, RPL13, RPL14, RPL23, RPL5, RPL6, RPL7, RPL8, RPL9, RPLP0, RPS16, RPS19, RPS2, RPS20, RPS21, RPS3, RPS4X, RPS6, RPS8, RPSA]
KEGG:05034	Alcoholism	7.36E-10	21	[H2AC20, H2AX, H2BC21, H4-16, H4C1, H4C11, H4C12, H4C13, H4C14, H4C15, H4C2, H4C3, H4C4, H4C5, H4C6, H4C8, H4C9, HDAC3, HDAC5, HDAC6, MACROH2A1]
KEGG:04110	Cell cycle	5.43E-07	14	[ATR, CCND1, CDC27, CDK7, CDKN2A, CHEK1, E2F1, PRKDC, RAD21, RBX1, SKP1, SKP2, SMC1A, SMC3]
KEGG:04066	HIF-1 signaling pathway	2.73E-05	11	[ALDOA, ENO1, GAPDH, IGF1R, LDHA, LDHB, PIK3R2, PRKCB, RBX1, RPS6, RPS6KB2]
KEGG:05225	Hepatocellular carcinoma	8.62E-05	13	[ACTB, CCND1, CDKN2A, CSNK1A1, E2F1, IGF1R, PIK3R2, PRKCB, RPS6KB2, SMARCA4, SMARCB1, SMARCC1, SMARCC2]
KEGG:00010	Glycolysis / Gluconeogenesis	7.28E-04	7	[ALDOA, ENO1, GAPDH, LDHA, LDHB, PKM, TPI1]
