# Supplementary material for: Network Pharmacology Approach for Predicting Targets of Zishen Yutai Pills on Premature Ovarian Insufficiency
Source: Evid Based Complement Alternat Med. 2021 Aug 4;2021:8215454. doi: 10.1155/2021/8215454 (PMC8357500; doi:10.1155/2021/8215454)
Supplement: Supplementary Materials — Supplementary Table 1: active compounds of each herb in ZSYTP. Supplementary Table 2: corresponding targets of active compounds: (A) two target genes of SDH; (B) 138 target genes of GQZ; (C) 202 target genes of TSZ; (D) 25 target genes of BJT; (E) 121 target genes of DS; (F) 11 target genes of BZ; (G) 283 target genes of DZ; (H) 13 target genes of SR; (J) 112 target genes of RS; (K) 76 target genes of SJS; (L) 115 target genes of AY; (M) 149 target genes of HSW; (N) 68 target genes of EJ; (P) 46 target genes of LJJ. Supplementary Table 3: POI targets. Supplementary Table 4: compounds and targets attributes of C-T network. Supplementary Table 5: data of compound-target network. Supplementary Table 6: analysed C-T network: (A) active compounds of C-T network and corresponding degrees; (B) targets of C-T network and corresponding degrees. Supplementary Table 7: ZSYTP core PPI network. Supplementary Table 8: gene ontology and KEGG analysis: (A) GO-BP analysis; (B) GO-MF analysis; (C) GO-CC analysis; (D) KEGG pathway enrichment analysis. [file ST1 Active compounds of each herb in ZSYT.pdf › 8215454.f1/ST1 Active compounds of each herb in ZSYT.pdf]

Supplementary Table S1. Active compounds of each herb in ZSYT

Herb Name	Mol ID	Chemical Name	OB	DL	InCHIkey	Canonical SMILES	Source
Rehmanniae Radix Praeparata (Shu Di Huang, SDH)	MOL000359	Sitosterol	36.914	0.7512	KZJWDPNRJALLNS-ZFVHJZABSA-N	CCC(CCC(C)C1CCC2C1(CCC3C2CC=C4C3(CCC(C4)O)C)C)C(C)C	TCMSP
Rehmanniae Radix Praeparata (Shu Di Huang, SDH)	MOL000449	Stigmasterol	43.830	0.75665	HCXVJBMSMIARIN-PHZDYDNGSA-N	CCC(C=CC(C)C1CCC2C1(CCC3C2CC=C4C3(CCC(C4)O)C)C)C(C)C	TCMSP
Lycii Fructus (Gou Qi Zi, GQZ)	MOL010234	delta-Carotene	31.801	0.546	WGIYGODPCLMGQH-GOXCNPTKSA-N	CC1=CCCC(C1C=CC(=CC=CC(=CC=CC=C(C)C=CC=C(C)C=CC=C(C)CCC=C(C)C	TCMSP
Lycii Fructus (Gou Qi Zi, GQZ)	MOL009678	lanost-8-enol	34.226	0.742	MBZYKEVPFYHDOH-ZSLNGYDASA-N		TCMSP
Lycii Fructus (Gou Qi Zi, GQZ)	MOL009677	lanost-8-en-3beta-ol	34.226	0.740	MBZYKEVPFYHDOH-BQNIITSRSA-N	CC(C)CCCC(C)C1CCC2(C1(CCC3=C2CCC4C3(CCC(C4(C)C)O)C)C)C	TCMSP
Lycii Fructus (Gou Qi Zi, GQZ)	MOL009604	14b-pregnane	34.779	0.337	JWMFYGXQPXQEEM-MTUNSFAGSA-N	CCC1CCC2C1(CCC3C2CCC4C3(CCCC4)C)C	TCMSP
Lycii Fructus (Gou Qi Zi, GQZ)	MOL000358	beta-sitosterol	36.914	0.751	KZJWDPNRJALLNS-VJSFXXLFSA-N	CCC(CCC(C)C1CCC2C1(CCC3C2CC=C4C3(CCC(C4)O)C)C)C(C)C	TCMSP
Lycii Fructus (Gou Qi Zi, GQZ)	MOL008173	daucosterol_qt	36.914	0.753	KZJWDPNRJALLNS-ZBNQVYKFSA-N		TCMSP
Lycii Fructus (Gou Qi Zi, GQZ)	MOL009617	24-ethylcholest-22-enol	37.095	0.751	CSVWWLUMXNHWSU-YASJBQHGSA-N	CCC(C=CC(C)C1CCC2C1(CCC3C2CCC4C3(CCCC4)C)C)C(C)CO	TCMSP
Lycii Fructus (Gou Qi Zi, GQZ)	MOL009615	24-Methylenecycloartan-3beta,	37.317	0.798	FJXNINQGUTYPNE-DLJSKDEVSA-N	CC(C)C(=C)CCC(CO)C1CCC2(C1(CCC34C2CCC5C3(C4)CCC(C5(C)C)O)C)C	TCMSP
Lycii Fructus (Gou Qi Zi, GQZ)	MOL005438	campesterol	37.577	0.715	SGNBVLSWZMBQTH-KAASKNFBSA-N		TCMSP
Lycii Fructus (Gou Qi Zi, GQZ)	MOL009635	4,24-methyllophenol	37.835	0.750	AOQRDALGACAKHI-AHSCMWHKSA-N		TCMSP
Lycii Fructus (Gou Qi Zi, GQZ)	MOL000953	CLR	37.874	0.677	HVYWMOMLDIMFJA-DPAQBDIFSA-N	CC(C)CCCC(C)C1CCC2C1(CCC3C2CC=C4C3(CCC(C4)O)C)C	TCMSP
Lycii Fructus (Gou Qi Zi, GQZ)	MOL009620	24-methyl-31-norlanost-9(11)-	38.000	0.751	NWPXYRKVJLBSQU-NTJPQSOTSA-N		TCMSP
Lycii Fructus (Gou Qi Zi, GQZ)	MOL009639	Lophenol	38.129	0.714	LMYZQUNLYGJIHI-SPONXPENSA-N	CC1C(CCC2(C1CC=C3C2CCC4(C3CCC4C(C)CCCC(C)C)C)C)O	TCMSP
Lycii Fructus (Gou Qi Zi, GQZ)	MOL009633	31-norlanost-9(11)-enol	38.354	0.725	SZCKXGWHINUNKB-SNKUZYNWSA-N		TCMSP
Lycii Fructus (Gou Qi Zi, GQZ)	MOL009662	Lantadene A	38.679	0.574	KCLIRHUTOPOHKJ-DMKBDCOSSA-N		TCMSP
Lycii Fructus (Gou Qi Zi, GQZ)	MOL009631	31-Norcyclolaudenol	38.682	0.814	OSKBBWPZDJRQMS-TVCYAWOFSA-N		TCMSP
Lycii Fructus (Gou Qi Zi, GQZ)	MOL003578	Cycloartenol	38.686	0.781	ONQRKEUAIJMULO-YBXTVTTCSA-N	CC(CCC=C(C)C)C1CCC2(C1(CCC34C2CCC5C3(C4)CCC(C5(C)C)O)C)C	TCMSP
Lycii Fructus (Gou Qi Zi, GQZ)	MOL009640	4alpha,14alpha,24-trimethylch	38.910	0.758	GCZFZGBLBXBCJP-VXRRTDEQSA-N		TCMSP
Lycii Fructus (Gou Qi Zi, GQZ)	MOL009660	methyl (1R,4aS,7R,7aS)-4a,7-	39.428	0.466	RWMXKBUPLSNIJL-VALIWVFLSA-N	CC1(CCC2(C1C(OC=C2C(=O)OC)OC3C(C(C(C(O3)CO)O)O)O)O)O	TCMSP
Lycii Fructus (Gou Qi Zi, GQZ)	MOL009653	Cycloeucalenol	39.726	0.794	HUNLTIZKNQDZEI-PGFZVWMDSA-N	CC1C2CCC3C4(CCC(C4(CCC35C2(C5)CCC1O)C)C(C)CCC(=C)C(C)C)C	TCMSP
Lycii Fructus (Gou Qi Zi, GQZ)	MOL009646	7-O-Methylluteolin-6-C-beta-g	40.774	0.305	PHGRMBKBKPAXKQ-LLVKDONJSA-N		TCMSP
Lycii Fructus (Gou Qi Zi, GQZ)	MOL001494	Mandenol	41.996	0.193	FMMOOAYVCKXGMF-MURFETPASA-N	CCCCCC=CCC=CCCCCCCCC(=O)OCC	TCMSP
Lycii Fructus (Gou Qi Zi, GQZ)	MOL009656	(E,E)-1-ethyl octadeca-3,13-dic	41.996	0.194	WDXJYOKJLIZASF-RSIFQWQVSA-N		TCMSP
Lycii Fructus (Gou Qi Zi, GQZ)	MOL001979	LAN	42.119	0.748	CAHGCLMLTWQZNJ-BQNIITSRSA-N	CC(CCC=C(C)C)C1CCC2(C1(CCC3=C2CCC4C3(CCC(C4(C)C)O)C)C)C	TCMSP
Lycii Fructus (Gou Qi Zi, GQZ)	MOL009650	Atropine	42.159	0.193	RKUNBYITZUJHSG-JJXSEGSLSA-N	CN1C2CCC1CC(C2)OC(=O)C(CO)C3=CC=CC=C3	TCMSP
Lycii Fructus (Gou Qi Zi, GQZ)	MOL009634	31-norlanosterol	42.205	0.730	KLZWTHGLLDRKHD-MGAQDEEHSA-N		TCMSP
Lycii Fructus (Gou Qi Zi, GQZ)	MOL009642	4alpha-methyl-24-ethylcholest	42.295	0.783	HIIFUGOBGVCVLO-MTFRKTCUSA-N	CCC(=C(C)C)CCC(C)C1CCC2C1(CCC3C2=CCC4C3(CCC(C4C)O)C)C	TCMSP
Lycii Fructus (Gou Qi Zi, GQZ)	MOL009621	24-methylenelanost-8-enol	42.368	0.768	XJLZCPIILZRCPS-MCOFMKOSSA-N		TCMSP
Lycii Fructus (Gou Qi Zi, GQZ)	MOL009681	Obtusifoliol	42.552	0.757	MMNYKQIDRZNIKT-VSADUBDNSA-N	CC1C2CCC3=C(C2(CCC1O)C)CCC4(C3(CCC4C(C)CCC(=C)C(C)C)C)C	TCMSP
Lycii Fructus (Gou Qi Zi, GQZ)	MOL009641	4alpha,24-dimethylcholesta-7,	42.653	0.753	KPIRFXVTLHBVFL-AAGDOFLISA-N	CC1C(CCC2(C1CC=C3C2CCC4(C3CCC4C(C)CCC(=C(C)C)C)C)C)O	TCMSP
Lycii Fructus (Gou Qi Zi, GQZ)	MOL001323	Sitosterol alpha1	43.281	0.784	LPZCCMIISIBREI-JXMPMKKESA-N	CC=C(CCC(C)C1CCC2C1(CCC3C2=CCC4C3(CCC(C4C)O)C)C)C(C)C	TCMSP
Lycii Fructus (Gou Qi Zi, GQZ)	MOL009644	6-Fluoroindole-7-Dehydrochol	43.726	0.722	HMEVHMIHKDZWLI-WXBCRLQESA-N	CC(C)CCCC(C)C1CCC2C1(CCC3C2=CC(=C4C3(CCC(C4)O)C)F)C	TCMSP
Lycii Fructus (Gou Qi Zi, GQZ)	MOL009622	Fucosterol	43.776	0.757	OSELKOCHBMDKEJ-JUGJNGJRSA-N	CC=C(CCC(C)C1CCC2C1(CCC3C2CC=C4C3(CCC(C4)O)C)C)C(C)C	TCMSP
Lycii Fructus (Gou Qi Zi, GQZ)	MOL000449	Stigmasterol	43.830	0.757	HCXVJBMSMIARIN-PHZDYDNGSA-N	CC=C(CCC(C)C1CCC2C1(CCC3C2CC=C4C3(CCC(C4)O)C)C)C(C)C	TCMSP
Lycii Fructus (Gou Qi Zi, GQZ)	MOL009618	24-ethylcholesta-5,22-dienol	43.830	0.756	HCXVJBMSMIARIN-KRUSZOTOSA-N	CCC(C=CC(C)C1CCC2C1(CCC3C2CC=C4C3(CCC(C4)O)C)C)C(C)C	TCMSP
Lycii Fructus (Gou Qi Zi, GQZ)	MOL009665	Physcion-8-O-beta-D-gentiobic	43.904	0.624	LHWONDXFTUKXDH-BFTLVBKUSA-N	CC1=CC2=C(C(=C1)OC3C(C(C(C(O3)COC4C(C(C(C(O4)CO)O)O)O)O)O)O)C(=O)C5=	TCMSP
Lycii Fructus (Gou Qi Zi, GQZ)	MOL007449	24-methylidenelophenol	44.193	0.753	RSMKYRDCCSNYFM-AAGDOFLISA-N	CC1C(CCC2(C1CC=C3C2CCC4(C3CCC4C(C)CCC(=C)C(C)C)C)C)O	TCMSP
Lycii Fructus (Gou Qi Zi, GQZ)	MOL005406	Atropine	45.971	0.193	RKUNBYITZUJHSG-QKPAOTATSA-N		TCMSP
Lycii Fructus (Gou Qi Zi, GQZ)	MOL001495	Ethyl linolenate	46.101	0.197	JYYFMIOPGOFNPK-XSHSMGBESA-N	CCC=CCC=CCC=CCCCCCCCC(=O)OCC	TCMSP
Lycii Fructus (Gou Qi Zi, GQZ)	MOL009612	(24R)-4alpha-Methyl-24-ethylc	46.357	0.840	YXSNMSCMEXMDCO-ZDOZIUNCSA-N		TCMSP
Lycii Fructus (Gou Qi Zi, GQZ)	MOL000098	Quercetin	46.433	0.275	REFJWTPEDVJJIY-UHFFFAOYSA-N	C1=CC(=C(C=C1C2=C(C(=O)C3=C(C=C(C=C3O2)O)O)O)O)O	TCMSP
Lycii Fructus (Gou Qi Zi, GQZ)	MOL009651	Cryptoxanthin monoepoxide	46.954	0.561	CMOLUFWHADIFGS-WZIUPQIASA-N		TCMSP
Lycii Fructus (Gou Qi Zi, GQZ)	MOL006209	Cyanin	47.421	0.759	ZNWVDIROEWRDJT-DARPEHSRSA-N		TCMSP
Lycii Fructus (Gou Qi Zi, GQZ)	MOL008400	Glycitein	50.479	0.238	DXYUAIFZCFRPTH-UHFFFAOYSA-N	COC1=C(C=C2C(=C1)C(=O)C(=CO2)C3=CC=C(C=C3)O)O	TCMSP
Lycii Fructus (Gou Qi Zi, GQZ)	MOL009664	Physalin A	91.706	0.272	VELDODQHYQSJOF-RLGRCWQRSA-N		TCMSP
Cuscutae Semen (Tu Si Zi, TSZ)	MOL000358	beta-sitosterol	36.914	0.751	KZJWDPNRJALLNS-VJSFXXLFSA-N	CCC(CCC(C)C1CCC2C1(CCC3C2CC=C4C3(CCC(C4)O)C)C)C(C)C	TCMSP
Cuscutae Semen (Tu Si Zi, TSZ)	MOL005043	campest-5-en-3beta-ol	37.577	0.715	SGNBVLSWZMBQTH-PODYLUTMSA-N	CC(C)C(C)CCC(C)C1CCC2C1(CCC3C2CC=C4C3(CCC(C4)O)C)C	TCMSP
Cuscutae Semen (Tu Si Zi, TSZ)	MOL000953	CLR	37.874	0.677	HVYWMOMLDIMFJA-DPAQBDIFSA-N	CC(C)CCCC(C)C1CCC2C1(CCC3C2CC=C4C3(CCC(C4)O)C)C	TCMSP
Cuscutae Semen (Tu Si Zi, TSZ)	MOL000184	NSC63551	39.254	0.759	RTLUSWHIKFIQFU-DALQDKCESA-N	CCC(C=CC(C)C1CCC2C1(CCC3C2CCC4C3(CCC(=O)C4)C)C)C(C)C	TCMSP
Cuscutae Semen (Tu Si Zi, TSZ)	MOL000422	Kaempferol	41.882	0.241	IYRMWMYZSQPJKC-UHFFFAOYSA-N	C1=CC(=CC=C1C2=C(C(=O)C3=C(C=C(C=C3O2)O)O)O)O	TCMSP
Cuscutae Semen (Tu Si Zi, TSZ)	MOL005440	Isofucosterol	43.776	0.758	OSELKOCHBMDKEJ-WGMIZEQOSA-N	CC=C(CCC(C)C1CCC2C1(CCC3C2CC=C4C3(CCC(C4)O)C)C)C(C)C	TCMSP
Cuscutae Semen (Tu Si Zi, TSZ)	MOL000098	Quercetin	46.433	0.275	REFJWTPEDVJJIY-UHFFFAOYSA-N	C1=CC(=C(C=C1C2=C(C(=O)C3=C(C=C(C=C3O2)O)O)O)O)O	TCMSP
Cuscutae Semen (Tu Si Zi, TSZ)	MOL000354	Isorhamnetin	49.604	0.306	IZQSVPBOUDKVDZ-UHFFFAOYSA-N	COC1=C(C=CC(=C1)C2=C(C(=O)C3=C(C=C(C=C3O2)O)O)O)O	TCMSP
Cuscutae Semen (Tu Si Zi, TSZ)	MOL006649	sophranol	55.417	0.282	VQYBAEAOOJBSTR-RJZRQDKASA-N		TCMSP
Cuscutae Semen (Tu Si Zi, TSZ)	MOL001558	Sesamin	56.547	0.827	PEYUIKBAABKQKQ-AFHBHXEDSA-N	C1C2C(COC2C3=CC4=C(C=C3)OCO4)C(O1)C5=CC6=C(C=C5)OCO6	TCMSP
Cuscutae Semen (Tu Si Zi, TSZ)	MOL005944	Matrine	63.775	0.249	ZSBXGIUJOOQZMP-JLNYLFASSA-N	C1CC2C3CCCN4C3C(CCC4)CN2C(=O)C1	TCMSP
Morindae Officinalis Radix (Ba Ji Tian, BJT)	MOL002883	Ethyl oleate (NF)	32.397	0.191	LVGKNOAMLMIIKO-QXMHVHEDSA-N	CCCCCCCCC=CCCCCCCCC(=O)OCC	TCMSP
Morindae Officinalis Radix (Ba Ji Tian, BJT)	MOL006147	Alizarin-2-methylether	32.809	0.210	BYQWRZGQEZAOPQ-UHFFFAOYSA-N	COC1=C(C2=C(C=C1)C(=O)C3=CC=CC=C3C2=O)O	TCMSP
Morindae Officinalis Radix (Ba Ji Tian, BJT)	MOL001506	Supraene	33.546	0.422	YYGNTYWPHWGJRM-AAJYLUCBSA-N	CC(=CCCC(=CCCC(=CCCC=C(C)CCC=C(C)CCC=C(C)C)C)C)C	TCMSP
Morindae Officinalis Radix (Ba Ji Tian, BJT)	MOL009525	3beta-24S(R)-butyl-5-alkenyl-c	35.352	0.822	JTNJOYSWTWFIJO-STRJLHQDSA-N		TCMSP
Morindae Officinalis Radix (Ba Ji Tian, BJT)	MOL000359	sitosterol	36.914	0.751	KZJWDPNRJALLNS-ZFVHJZABSA-N	CCC(CCC(C)C1CCC2C1(CCC3C2CC=C4C3(CCC(C4)O)C)C)C(C)C	TCMSP
Morindae Officinalis Radix (Ba Ji Tian, BJT)	MOL000358	beta-sitosterol	36.914	0.751	KZJWDPNRJALLNS-VJSFXXLFSA-N	CCC(CCC(C)C1CCC2C1(CCC3C2CC=C4C3(CCC(C4)O)C)C)C(C)C	TCMSP
Morindae Officinalis Radix (Ba Ji Tian, BJT)	MOL009524	3beta,20(R),5-alkenyl-stigmast	36.914	0.751	KZJWDPNRJALLNS-OSBPDXFISA-N	CCC(CCC(C)C1CCC2C1(CCC3C2CC=C4C3(CCC(C4)O)C)C)C(C)C	TCMSP
Morindae Officinalis Radix (Ba Ji Tian, BJT)	MOL009562	Ohioensin-A	38.135	0.758	DBGJQYIYUBGFLT-GQIHDASDSA-N	C1C2C3C(C4=C(C=CC(=C4)O)C5=C3C(=C(C=C5O)O)C1=O)OC6=CC=CC=C26	TCMSP
Morindae Officinalis Radix (Ba Ji Tian, BJT)	MOL002879	Diop	43.593	0.392	IJFPVINAQGWBRJ-UHFFFAOYSA-N	CC(C)CCCCCOC(=O)C1=CC=CC=C1C(=O)OCCCCCC(C)C	TCMSP
Morindae Officinalis Radix (Ba Ji Tian, BJT)	MOL009541	Asperuloside tetraacetate	45.473	0.816	PNJMLKLECQXAHQ-GGHIHTNXSA-N	CC(=O)OCC1C(C(C(C(O1)OC2C3C4C(C=C3COC(=O)C)OC(=O)C4=CO2)OC(=O)C)O(	TCMSP
Morindae Officinalis Radix (Ba Ji Tian, BJT)	MOL009537	americanin A	46.706	0.349	NTXXGPYGMQQSML-MNSDHWDNSA-N		TCMSP
Morindae Officinalis Radix (Ba Ji Tian, BJT)	MOL009551	isoprincepin	49.121	0.774	RRXDUTVDPLTNBK-LTJUSVHVSA-N		TCMSP
Morindae Officinalis Radix (Ba Ji Tian, BJT)	MOL009558	2-hydroxyethyl 5-hydroxy-2-(2	62.316	0.258	BRYATOWTSDMLNN-UHFFFAOYSA-N	C1=CC=C(C(=C1)C(=O)C2=C(C=C(C(=C2)CO)O)C(=O)OCCO)O	TCMSP
Morindae Officinalis Radix (Ba Ji Tian, BJT)	MOL009519	(2R,3S)-(+)-3',5-Dihydroxy-4	77.238	0.335	SVPNMFZMHPLGRR-IAGOWNOFSA-N	COC1=C(C=C(C=C1)C2C(C(=O)C3=C(C=C(C=C3O2)OC)O)O)O	TCMSP
Morindae Officinalis Radix (Ba Ji Tian, BJT)	MOL009496	1,5,7-trihydroxy-6-methoxy-2-	80.423	0.378	WQUIDUAYYISUHB-UHFFFAOYSA-N	COCC1=C(C2=C(C=C1)C(=O)C3=C(C(=C(C=C3C2=O)O)OC)O)O	TCMSP
Morindae Officinalis Radix (Ba Ji Tian, BJT)	MOL009504	1-hydroxy-6-hydroxymethylan	81.765	0.212	DGGHVFUGMGGLON-UHFFFAOYSA-N	C1=CC2=C(C(=C1)O)C(=O)C3=C(C2=O)C=C(C=C3)CO	TCMSP
Morindae Officinalis Radix (Ba Ji Tian, BJT)	MOL009495	2-hydroxy-1,5-dimethoxy-6-(m	95.852	0.372	HEVJQUHAJKODII-UHFFFAOYSA-N	COCC1=C(C2=C(C=C1)C(=O)C3=C(C2=O)C=CC(=C3OC)O)OC	TCMSP
Morindae Officinalis Radix (Ba Ji Tian, BJT)	MOL009503	1-hydroxy-3-methoxy-9,10-ant	104.325	0.209	LRCKMFKYRNMGRL-UHFFFAOYSA-N	COC1=CC2=C(C(=C1)O)C(=O)C3=CC=CC=C3C2=O	TCMSP
Morindae Officinalis Radix (Ba Ji Tian, BJT)	MOL009500	1,6-dihydroxy-5-methoxy-2-(m	104.539	0.339	KKHLOCIPXRSWKM-UHFFFAOYSA-N	COCC1=C(C2=C(C=C1)C(=O)C3=C(C2=O)C=CC(=C3OC)O)O	TCMSP
Morindae Officinalis Radix (Ba Ji Tian, BJT)	MOL009513	2-hydroxy-1,8-dimethoxy-7-m	112.303	0.372	ZAHPVPZTAQYVKO-UHFFFAOYSA-N		TCMSP
Codonopsis Radix (Dang Shen, DS)	MOL007059	3-beta-Hydroxymethyllenetans	32.161	0.409	RUJKJFRMCYQMLH-ZDUSSCGKSA-N	CC1=COC2=C1C(=O)C(=O)C3=C2C=CC4=C3CCC(C4=C)O	TCMSP
Codonopsis Radix (Dang Shen, DS)	MOL008391	5alpha-Stigmastan-3,6-dione	33.115	0.790	HMMVBUVVQLUGQA-CFLIYZTGSA-N		TCMSP
Codonopsis Radix (Dang Shen, DS)	MOL000006	Luteolin	36.163	0.246	IQPNAANSBPBGFQ-UHFFFAOYSA-N	C1=CC(=C(C=C1C2=CC(=O)C3=C(C=C(C=C3O2)O)O)O)O	TCMSP
Codonopsis Radix (Dang Shen, DS)	MOL006774	stigmast-7-enol	37.423	0.751	YSKVBPGQYRAUQO-UBCZCFGNSA-N	CCC(CCC(C)C1CCC2C1(CCC3C2=CCC4C3(CCC(C4)O)C)C)C(C)C	TCMSP
Codonopsis Radix (Dang Shen, DS)	MOL008393	7-(beta-Xylosyl)cephalomannir	38.327	0.286	YVFWOWYGCXXKFY-IICLVWGGSA-N		TCMSP
Codonopsis Radix (Dang Shen, DS)	MOL006554	Taraxerol	38.403	0.767	GGGUGZHBAOMSFJ-GADYQYKKSA-N	CC1(CCC2(CC=C3C4(CCC5C(C(CCC5(C4CCC3(C2C1C)C)C)O)(C)C)C)C)C	TCMSP
Codonopsis Radix (Dang Shen, DS)	MOL004492	Chrysanthemaxanthin	38.724	0.584	JRHJXXLCNATYLS-HYHFCDFPSA-N		TCMSP
Codonopsis Radix (Dang Shen, DS)	MOL007514	methyl icosa-11,14-dienoate	39.667	0.229	GWJCFAOQCNNFAM-ZDVGBALWSA-N	CCCCCC=CCC=CCCCCCCCCCC(=O)OC	TCMSP
Codonopsis Radix (Dang Shen, DS)	MOL008406	Spinoside A	39.967	0.403	KNKOUUNHYIXOAK-OCCINUARSA-N		TCMSP
Codonopsis Radix (Dang Shen, DS)	MOL008411	11-Hydroxyrankinidine	40.003	0.662	FALAMCOLIJTCTR-XAVCKHEUSA-N		TCMSP
Codonopsis Radix (Dang Shen, DS)	MOL003896	7-Methoxy-2-methyl isoflavon	42.565	0.199	XRGWZIGGCNSFRY-UHFFFAOYSA-N	CC1=C(C(=O)C2=C(O1)C=C(C=C2)OC)C3=CC=CC=C3	TCMSP
Codonopsis Radix (Dang Shen, DS)	MOL001006	poriferasta-7,22E-dien-3beta-ol	42.979	0.756	JZVFJDZBLUFKCA-INYURWPISA-N	CCC(C=CC(C)C1CCC2C1(CCC3C2=CCC4C3(CCC(C4)O)C)C)C(C)C	TCMSP
Codonopsis Radix (Dang Shen, DS)	MOL004355	Spinasterol	42.979	0.755	JZVFJDZBLUFKCA-FXIAWGAOSA-N	CCC(C=CC(C)C1CCC2C1(CCC3C2=CCC4C3(CCC(C4)O)C)C)C(C)C	TCMSP
Codonopsis Radix (Dang Shen, DS)	MOL002879	Diop	43.593	0.392	IJFPVINAQGWBRJ-UHFFFAOYSA-N	CC(C)CCCCCOC(=O)C1=CC=CC=C1C(=O)OCCCCCC(C)C	TCMSP
Codonopsis Radix (Dang Shen, DS)	MOL003036	ZINC03978781	43.830	0.756	HCXVJBMSMIARIN-NKMAIEQZSA-N	CCC(C=CC(C)C1CCC2C1(CCC3C2CC=C4C3(CCC(C4)O)C)C)C(C)C	TCMSP
Codonopsis Radix (Dang Shen, DS)	MOL000449	Stigmasterol	43.830	0.757	HCXVJBMSMIARIN-PHZDYDNGSA-N	CCC(C=CC(C)C1CCC2C1(CCC3C2CC=C4C3(CCC(C4)O)C)C)C(C)C	TCMSP
Codonopsis Radix (Dang Shen, DS)	MOL008407	(8S,9S,10R,13R,14S,17R)-17	45.405	0.762	XWDAKKDQJHVMKG-LPJPOILFSA-N	CCC(C=CC(C)C1CCC2C1(CCC3C2CC=C4C3(CCC(=O)C4)C)C)C(C)C	TCMSP
Codonopsis Radix (Dang Shen, DS)	MOL008397	Daturilin	50.365	0.768	QUGZOXCXHGEACS-ZFEQYVDASA-N		TCMSP
Codonopsis Radix (Dang Shen, DS)	MOL008400	glycitein	50.479	0.238	DXYUAIFZCFRPTH-UHFFFAOYSA-N	COC1=C(C=C2C(=C1)C(=O)C(=CO2)C3=CC=C(C=C3)O)O	TCMSP
Codonopsis Radix (Dang Shen, DS)	MOL005321	Frutinone A	65.904	0.342	RFWULRHBGYKEEZ-UHFFFAOYSA-N	C1=CC=C2C(=C1)C3=C(C(=O)C4=CC=CC=C4O3)C(=O)O2	TCMSP
Codonopsis Radix (Dang Shen, DS)	MOL002140	Perlolyrine	65.948	0.275	KFUCYPGCMLPUMT-UHFFFAOYSA-N	C1=CC=C2C(=C1)C3=C(N2)C(=NC=C3)C4=CC=C(O4)CO	TCMSP
Atractylodes Macrocephala Koidz. (Bai Zhu, BZ)	MOL000020	12-senecioyl-2E,8E,10E-atract	62.396	0.223	UJHAKGDWVZIMIT-PKPRIEKHSA-N		TCMSP
Atractylodes Macrocephala Koidz. (Bai Zhu, BZ)	MOL000021	14-acetyl-12-senecioyl-2E,8E,	60.313	0.305	CQDVFBMTEZFKKY-JAPNHWDDSA-N		TCMSP
Atractylodes Macrocephala Koidz. (Bai Zhu, BZ)	MOL000022	14-acetyl-12-senecioyl-2E,8Z,	63.371	0.300	CQDVFBMTEZFKKY-PUYRJQRLSA-N		TCMSP
Atractylodes Macrocephala Koidz. (Bai Zhu, BZ)	MOL000028	α-Amyrin	39.512	0.763	FSLPMRQHCOLESF-FRCIEAHOSA-N		TCMSP
Atractylodes Macrocephala Koidz. (Bai Zhu, BZ)	MOL000033	(3S,8S,9S,10R,13R,14S,17R)	36.228	0.783	KLEXDBGYSOIREE-UIFQYPGESA-N	CCCC(CCC(C)C1CCC2C1(CCC3C2CC=C4C3(CCC(C4)O)C)C)C(C)C	TCMSP
Atractylodes Macrocephala Koidz. (Bai Zhu, BZ)	MOL000049	3β-acetoxyatractylone	54.067	0.219	YYGOPZCCRBWMBK-ZOBUZTSGSA-N		TCMSP
Atractylodes Macrocephala Koidz. (Bai Zhu, BZ)	MOL000072	8beta-ethoxy atractylenolide II	35.951	0.211	IKNWKYVCOWHPTC-BLAYRMRBSA-N	CCOC12CC3(CCCC(=C)C3(CC1=C(C(=O)O2)C)C)C	TCMSP
Atractylodes Macrocephala Koidz. (Bai Zhu, BZ)	MOL003152	Gentisin	64.062	0.213	XOXYHGOIRWABTC-UHFFFAOYSA-N	COC1=CC(=C2C(=C1)OC3=C(C2=O)C=C(C=C3)O)O	TCMSP
Atractylodes Macrocephala Koidz. (Bai Zhu, BZ)	MOL000358	beta-sitosterol	36.914	0.751	KZJWDPNRJALLNS-VJSFXXLFSA-N	CCC(CCC(C)C1CCC2C1(CCC3C2CC=C4C3(CCC(C4)O)C)C)C(C)C	TCMSP
Dipsaci Radix (Xu Duan, XD)	MOL000359	sitosterol	36.914	0.751	KZJWDPNRJALLNS-ZFVHJZABSA-N	CCC(CCC(C)C1CCC2C1(CCC3C2CC=C4C3(CCC(C4)O)C)C)C(C)C	TCMSP
Dipsaci Radix (Xu Duan, XD)	MOL009312	(E,E)-3,5-Di-O-caffeoylquinic	48.145	0.684	KRZBCHWVBQOTNZ-ZURXXRFOSA-N		TCMSP
Dipsaci Radix (Xu Duan, XD)	MOL009317	Cauloside A_qt	43.317	0.810	CPPAMFUQVBDPTG-XDGYBBFPSA-N		TCMSP
Dipsaci Radix (Xu Duan, XD)	MOL008188	Japonine	44.107	0.246	XTBMLWZKNWXUHV-UHFFFAOYSA-N	CN1C2=C(C=C(C=C2)OC)C(=O)C(=C1C3=CC=CC=C3)OC	TCMSP
Dipsaci Radix (Xu Duan, XD)	MOL009322	Sylvestroside III	48.018	0.527	MALYQXLMMWQXME-YWSLBQKTSA-N		TCMSP
Dipsaci Radix (Xu Duan, XD)	MOL009323	Sylvestroside III_qt	56.468	0.426	HPPQDJKDCBOQBR-HORHJMKNSA-N		TCMSP
Eucommiae Cortex (Du Zhong, DZ)	MOL002058	40957-99-1	57.204	0.619	VJOBNGRIBLNUKN-BMHXQBNDSA-N	COC1=CC(=CC(=C1O)OC)C2C3COC(C3CO2)C4=CC(=C(C=C4)O)OC	TCMSP
Eucommiae Cortex (Du Zhong, DZ)	MOL000211	Mairin	55.377	0.776	QGJZLNKBHJESQX-FZFNOLFKSA-N	CC(=C)C1CCC2(C1C3CCC4C5(CCC(C(C5CCC4(C3(CC2)C)C)(C)C)O)C)C(=O)O	TCMSP
Eucommiae Cortex (Du Zhong, DZ)	MOL000358	beta-sitosterol	36.914	0.751	KZJWDPNRJALLNS-VJSFXXLFSA-N	CCC(CCC(C)C1CCC2C1(CCC3C2CC=C4C3(CCC(C4)O)C)C)C(C)C	TCMSP
Eucommiae Cortex (Du Zhong, DZ)	MOL000422	kaempferol	41.882	0.241	IYRMWMYZSQPJKC-UHFFFAOYSA-N	C1=CC(=CC=C1C2=C(C(=O)C3=C(C=C(C=C3O2)O)O)O)O	TCMSP
Eucommiae Cortex (Du Zhong, DZ)	MOL004367	olivil	62.229	0.406	BVHIKUCXNBQDEM-XMCHAPAWSA-N		TCMSP
Eucommiae Cortex (Du Zhong, DZ)	MOL000443	Erythraline	49.177	0.550	TVOFUERNMZTYRM-KBXCAEBGSA-N	COC1CC23C(=CCN2CCC4=CC5=C(C=C34)OCO5)C=C1	TCMSP
Eucommiae Cortex (Du Zhong, DZ)	MOL005922	Acanthoside B	43.353	0.767	WEKCEGQSIIQPAQ-IRBNZIFYSA-N	COC1=CC(=CC(=C1O)OC)C2C3COC(C3CO2)C4=CC(=C(C(=C4)OC)OC5C(C(C(O5	TCMSP
Eucommiae Cortex (Du Zhong, DZ)	MOL006709	AIDS214634	92.427	0.549	CICMVLOHBZPXIT-WNISUXOKSA-N	COC1=C(C=CC(=C1)C2C3COC(C3(CO2)O)C4=CC(=C(C=C4)O)OC)O	TCMSP
Eucommiae Cortex (Du Zhong, DZ)	MOL007059	3-beta-Hydroxymethyllenetans	32.161	0.409	RUJKJFRMCYQMLH-ZDUSSCGKSA-N	CC1=COC2=C1C(=O)C(=O)C3=C2C=CC4=C3CCC(C4=C)O	TCMSP
Eucommiae Cortex (Du Zhong, DZ)	MOL000073	ent-Epicatechin	48.960	0.242	PFTAWBLQPZVEMU-ZFWWWQNUSA-N	C1C(C(OC2=CC(=CC(=C21)O)O)C3=CC(=C(C=C3)O)O)O	TCMSP
Eucommiae Cortex (Du Zhong, DZ)	MOL007563	Yangambin	57.525	0.808	HRLFUIXSXUASEX-RZTYQLBFSA-N	COC1=CC(=CC(=C1OC)OC)C2C3COC(C3CO2)C4=CC(=C(C(=C4)OC)OC)OC	TCMSP
Eucommiae Cortex (Du Zhong, DZ)	MOL009007	Eucommin A	30.513	0.848	GLGVEKKQPFRBAS-UOVCOODASA-N	COC1=CC(=CC(=C1OC2C(C(C(C(O2)CO)O)O)O)OC)C3C4COC(C4CO3)C5=CC(=C(C	TCMSP
Eucommiae Cortex (Du Zhong, DZ)	MOL009009	(+)-medioresinol	87.189	0.619	VJOBNGRIBLNUKN-HANNOOJCSA-N	COC1=CC(=CC(=C1O)OC)C2C3COC(C3CO2)C4=CC(=C(C=C4)O)OC	TCMSP
Eucommiae Cortex (Du Zhong, DZ)	MOL009015	(-)-Tabernemontanine	58.669	0.607	FFVRRQMGGGTQRH-MSPRAJMNSA-N	CCC1CN(C2CC3=C(C(=O)CC1C2C(=O)OC)NC4=CC=CC=C34)C	TCMSP
Eucommiae Cortex (Du Zhong, DZ)	MOL009027	Cyclopamine	55.422	0.821	QASFUMOKHFSJGL-LAFRSMQTSA-N	CC1CC2C(C(C3(O2)CCC4C5CC=C6CC(CCC6(C5CC4=C3C)C)O)C)NC1	TCMSP
Eucommiae Cortex (Du Zhong, DZ)	MOL009029	Dehydrodiconiferyl alcohol 4,g	51.442	0.395	GQNBCFQSSDOEHP-LNFBDUAVSA-N		TCMSP
Eucommiae Cortex (Du Zhong, DZ)	MOL009030	Dehydrodieugenol	30.103	0.239	KETPSFSOGFKJJY-UHFFFAOYSA-N	COC1=CC(=CC(=C1O)C2=C(C(=CC(=C2)CC=C)OC)O)CC=C	TCMSP
Eucommiae Cortex (Du Zhong, DZ)	MOL009031	Cinchonan-9-al, 6'-methoxy-, (	68.215	0.401	LOUPRKONTZGTKE-AFHBHXEDSA-N	COC1=CC2=C(C=CN=C2C=C1)C(C3CC4CCN3CC4C=C)O	TCMSP
Eucommiae Cortex (Du Zhong, DZ)	MOL009038	GBGB	45.577	0.827	FYZYXYLPBWLLGI-AUOPOVQUSA-N	COC(=O)C1=COC(C2C1CC=C2CO)OC3C(C(C(C(O3)COC4C(C(C(C(O4)CO)O)O)O)O)	TCMSP
Eucommiae Cortex (Du Zhong, DZ)	MOL009042	Helenalin	77.011	0.190	ZVLOPMNVFLSSAA-XEPQRQSNSA-N	CC1CC2C(C(C3(C1C=CC3=O)C)O)C(=C)C(=O)O2	TCMSP
Eucommiae Cortex (Du Zhong, DZ)	MOL009047	(+)-Eudesmin	33.287	0.620	PEUUVVGQIVMSAW-RZTYQLBFSA-N	COC1=C(C=C(C=C1)C2C3COC(C3CO2)C4=CC(=C(C=C4)OC)OC)OC	TCMSP
Eucommiae Cortex (Du Zhong, DZ)	MOL009053	4-[(2S,3R)-5-[(E)-3-hydroxypr	50.755	0.395	KUSXBOZNRPQEON-LNFBDUAVSA-N	COC1=CC(=CC2=C1OC(C2CO)C3=CC(=C(C=C3)O)OC)C=CCO	TCMSP
Eucommiae Cortex (Du Zhong, DZ)	MOL009055	hirsutin_qt	49.815	0.372	UIZYKATWALCYCD-UHFFFAOYSA-N		TCMSP
Eucommiae Cortex (Du Zhong, DZ)	MOL009057	liriodendrin_qt	53.137	0.800	KJHRHHLEZJFLDH-OZCSAQGGSA-N		TCMSP
Eucommiae Cortex (Du Zhong, DZ)	MOL000098	quercetin	46.433	0.275	REFJWTPEDVJJIY-UHFFFAOYSA-N	C1=CC(=C(C=C1C2=C(C(=O)C3=C(C=C(C=C3O2)O)O)O)O)O	TCMSP
Eucommiae Cortex (Du Zhong, DZ)	MOL002773	beta-carotene	37.184	0.584	OENHQHLEOONYIE-JLTXGRSLSA-N	CC1=C(C(CCC1)(C)C)C=CC(=CC=CC(=CC=CC=C(C)C=CC=C(C)C=CC2=C(CCCC2	TCMSP
Eucommiae Cortex (Du Zhong, DZ)	MOL008240	(E)-3-[4-[(1R,2R)-2-hydroxy-2	56.317	0.361	LWLOALZBDOVWAE-FMEUAVTJSA-N	COC1=C(C=CC(=C1)C=CC=O)OC(CO)C(C2=CC(=C(C=C2)O)OC)O	TCMSP
Eucommiae Cortex (Du Zhong, DZ)	MOL011604	Syringetin	36.822	0.374	UZMAPBJVXOGOFT-UHFFFAOYSA-N	COC1=CC(=CC(=C1O)OC)C2=C(C(=O)C3=C(C=C(C=C3O2)O)O)O	TCMSP
Amomum Aurantiacum (Sha Ren, SR)	MOL000358	beta-sitosterol	36.910	0.750	KZJWDPNRJALLNS-VJSFXXLFSA-N	CCC(CCC(C)C1CCC2C1(CCC3C2CC=C4C3(CCC(C4)O)C)C)C(C)C	TCMSP
Amomum Aurantiacum (Sha Ren, SR)	MOL000449	Stigmasterol	43.830	0.757	HCXVJBMSMIARIN-PHZDYDNGSA-N	CCC(C=CC(C)C1CCC2C1(CCC3C2CC=C4C3(CCC(C4)O)C)C)C(C)C	TCMSP
Amomum Aurantiacum (Sha Ren, SR)	MOL001755	24-Ethylcholest-4-en-3-one	36.084	0.757	RUVUHIUYGJBLGI-XJZKHKOHSA-N	CCC(CCC(C)C1CCC2C1(CCC3C2CCC4=CC(=O)CCC34C)C)C(C)C	TCMSP
Amomum Aurantiacum (Sha Ren, SR)	MOL001771	poriferast-5-en-3beta-ol	36.914	0.750	KZJWDPNRJALLNS-FBZNIEFRSA-N	CCC(CCC(C)C1CCC2C1(CCC3C2CC=C4C3(CCC(C4)O)C)C)C(C)C	TCMSP
Amomum Aurantiacum (Sha Ren, SR)	MOL001973	Sitosteryl acetate	40.390	0.851	PBWOIPCULUXTNY-LBKBYZTLSA-N	CCC(CCC(C)C1CCC2C1(CCC3C2CC=C4C3(CCC(C4)OC(=O)C)C)C)C(C)C	TCMSP
Amomum Aurantiacum (Sha Ren, SR)	MOL003975	icosa-11,14,17-trienoic acid m	44.814	0.234	XQAVRBUXEPJVRC-JSIPCRQOSA-N	CCC=CCC=CCC=CCCCCCCCCCC(=O)OC	TCMSP
Amomum Aurantiacum (Sha Ren, SR)	MOL007180	vitamin-e	32.286	0.696	WIGIZIANZCJQQY-RUCARUNLSA-N		TCMSP
Amomum Aurantiacum (Sha Ren, SR)	MOL007514	methyl icosa-11,14-dienoate	39.667	0.229	GWJCFAOQCNNFAM-ZDVGBALWSA-N	CCCCCC=CCC=CCCCCCCCCCC(=O)OC	TCMSP
Amomum Aurantiacum (Sha Ren, SR)	MOL007535	(5S,8S,9S,10R,13R,14S,17R)	33.115	0.788	HMMVBUVVQLUGQA-UMQMBAGDSA-N	CCC(CCC(C)C1CCC2C1(CCC3C2CC(=O)C4C3(CCC(=O)C4)C)C)C(C)C	TCMSP
Amomum Aurantiacum (Sha Ren, SR)	MOL007536	Stigmasta-5,22-dien-3-beta-yl	46.442	0.857	IZEUIYYDWBKERE-ZRODXFKISA-N	CCC(C=CC(C)C1CCC2C1(CCC3C2CC=C4C3(CCC(C4)OC(=O)C)C)C)C(C)C	TCMSP
Panax Ginseng (Ren Shen, RS)	MOL005314	Celabenzine	101.883	0.488	LSYKFBZWBDMZLQ-OAQYLSRUSA-N		TCMSP
Panax Ginseng (Ren Shen, RS)	MOL005308	Aposiopolamine	66.647	0.220	UPWMWFSEBOFTNA-SBAKFKMJSA-N		TCMSP
Panax Ginseng (Ren Shen, RS)	MOL005321	Frutinone A	65.904	0.342	RFWULRHBGYKEEZ-UHFFFAOYSA-N	C1=CC=C2C(=C1)C3=C(C(=O)C4=CC=CC=C4O3)C(=O)O2	TCMSP
Panax Ginseng (Ren Shen, RS)	MOL003648	Inermin	65.831	0.538	HUKSJTUUSUGIDC-BDJLRTHQSA-N	C1C2C(C3=C(O1)C=C(C=C3)O)OC4=CC5=C(C=C24)OCO5	TCMSP
Panax Ginseng (Ren Shen, RS)	MOL005356	Girinimbin	61.215	0.315	GAEQWKVGMHUUKO-UHFFFAOYSA-N	CC1=CC2=C(C3=C1OC(C=C3)(C)C)NC4=CC=CC=C42	TCMSP
Panax Ginseng (Ren Shen, RS)	MOL000787	Fumarine	59.263	0.827	GPTFURBXHJWNHR-UHFFFAOYSA-N	CN1CCC2=CC3=C(C=C2C(=O)CC4=C(C1)C5=C(C=C4)OCO5)OCO3	TCMSP
Panax Ginseng (Ren Shen, RS)	MOL005360	malkangunin	57.714	0.626	DTMIMKTZETWDJV-DUUKBJRLSA-N		TCMSP
Panax Ginseng (Ren Shen, RS)	MOL005384	suchilactone	57.519	0.556	GVNUFBXIXQNOCF-CRNMQVKPSA-N	COC1=C(C=C(C=C1)CC2COC(=O)C2=CC3=CC4=C(C=C3)OCO4)OC	TCMSP
Panax Ginseng (Ren Shen, RS)	MOL005320	Arachidonate	45.573	0.205	YZXBAPSDXZZRGB-CGRWFSSPSA-N	CCCCCC=CCC=CCC=CCC=CCCCC(=O)O	TCMSP
Panax Ginseng (Ren Shen, RS)	MOL000449	Stigmasterol	43.830	0.757	HCXVJBMSMIARIN-PHZDYDNGSA-N	CCC(C=CC(C)C1CCC2C1(CCC3C2CC=C4C3(CCC(C4)O)C)C)C(C)C	TCMSP
Panax Ginseng (Ren Shen, RS)	MOL002879	Diop	43.593	0.392	IJFPVINAQGWBRJ-UHFFFAOYSA-N	CC(C)CCCCCOC(=O)C1=CC=CC=C1C(=O)OCCCCCC(C)C	TCMSP
Panax Ginseng (Ren Shen, RS)	MOL000422	kaempferol	41.882	0.241	IYRMWMYZSQPJKC-UHFFFAOYSA-N	C1=CC(=CC=C1C2=C(C(=O)C3=C(C=C(C=C3O2)O)O)O)O	TCMSP
Panax Ginseng (Ren Shen, RS)	MOL005318	Dianthramine	40.446	0.197	SVZLTRRSGWXPBL-UHFFFAOYSA-N	C1=CC(=C(C=C1O)NC2=C(C=CC(=C2)O)C(=O)O)C(=O)O	TCMSP
Panax Ginseng (Ren Shen, RS)	MOL005401	ginsenoside Rg5_qt	39.563	0.785	FCMJTYFZRUKOPU-FFFKMGBISA-N		TCMSP
Panax Ginseng (Ren Shen, RS)	MOL005317	Deoxyharringtonine	39.274	0.812	WRCBXHDQHPUVHW-CDRKEARJSA-N		TCMSP
Panax Ginseng (Ren Shen, RS)	MOL004492	Chrysanthemaxanthin	38.724	0.584	JRHJXXLCNATYLS-HYHFCDFPSA-N		TCMSP
Panax Ginseng (Ren Shen, RS)	MOL000358	beta-sitosterol	36.914	0.751	KZJWDPNRJALLNS-VJSFXXLFSA-N	CCC(CCC(C)C1CCC2C1(CCC3C2CC=C4C3(CCC(C4)O)C)C)C(C)C	TCMSP
Panax Ginseng (Ren Shen, RS)	MOL005399	alexandrin_qt	36.914	0.753	KZJWDPNRJALLNS-HEEOYUHISA-N		TCMSP
Panax Ginseng (Ren Shen, RS)	MOL005344	Ginsenoside rh2	36.320	0.559	CKUVNOCSBYYHIS-IRFFNABBSA-N	CC(=CCCC(C)(C1CCC2(C1C(CC3C2(CCC4C3(CCC(C4(C)C)OC5C(C(C(C(O5)CO)O)(	TCMSP
Panax Ginseng (Ren Shen, RS)	MOL005376	Panaxadiol	33.088	0.794	PVLHOJXLNBFHDX-BMTJSWHCSA-N		TCMSP
Panax Ginseng (Ren Shen, RS)	MOL005357	Gomisin B	31.990	0.829	XDVOVYYAPHHHBE-YGIMCSHQSA-N	CC=C(C)C(=O)OC1C2=CC(=C(C(=C2C3C(=CC(C1(C)O)C)C=C4C(=C3OC)OCO4)OC	TCMSP
Panax Ginseng (Ren Shen, RS)	MOL005348	Ginsenoside-Rh4_qt	31.112	0.778	JKPOYAJYRYOGBN-ARWCPIPWSA-N		TCMSP
Herba Taxili (Sang Ji Sheng, SJS)	MOL000359	sitosterol	36.914	0.751	KZJWDPNRJALLNS-ZFVHJZABSA-N	CCC(CCC(C)C1CCC2C1(CCC3C2CC=C4C3(CCC(C4)O)C)C)C(C)C	TCMSP
Herba Taxili (Sang Ji Sheng, SJS)	MOL000098	quercetin	46.433	0.275	REFJWTPEDVJJIY-UHFFFAOYSA-N	C1=CC(=C(C=C1C2=C(C(=O)C3=C(C=C(C=C3O2)O)O)O)O)O	TCMSP
Folium Artemisiae Argyi (Ai Ye, AY)	MOL002883	Ethyl oleate (NF)	32.397	0.191	LVGKNOAMLMIIKO-QXMHVHEDSA-N	CCCCCCCCC=CCCCCCCCC(=O)OCC	TCMSP
Folium Artemisiae Argyi (Ai Ye, AY)	MOL000358	beta-sitosterol	36.914	0.751	KZJWDPNRJALLNS-VJSFXXLFSA-N	CCC(CCC(C)C1CCC2C1(CCC3C2CC=C4C3(CCC(C4)O)C)C)C(C)C	TCMSP
Folium Artemisiae Argyi (Ai Ye, AY)	MOL005741	cycloartenol acetate	41.107	0.803	PQNTWKDHNSWVPU-GUULDYRZSA-N		TCMSP
Folium Artemisiae Argyi (Ai Ye, AY)	MOL005720	24-methylenecycloartanone	41.114	0.794	AEAWOMODYBIREN-ZHKFOJSISA-N		TCMSP
Folium Artemisiae Argyi (Ai Ye, AY)	MOL001494	Mandenol	41.996	0.193	FMMOOAYVCKXGMF-MURFETPASA-N	CCCCCC=CCC=CCCCCCCCC(=O)OCC	TCMSP
Folium Artemisiae Argyi (Ai Ye, AY)	MOL001040	(2R)-5,7-dihydroxy-2-(4-hydro	42.363	0.211	FTVWIRXFELQLPI-CYBMUJFWSA-N	C1C(OC2=CC(=CC(=C2C1=O)O)O)C3=CC=C(C=C3)O	TCMSP
Folium Artemisiae Argyi (Ai Ye, AY)	MOL000449	Stigmasterol	43.830	0.757	HCXVJBMSMIARIN-PHZDYDNGSA-N	CCC(C=CC(C)C1CCC2C1(CCC3C2CC=C4C3(CCC(C4)O)C)C)C(C)C	TCMSP
Folium Artemisiae Argyi (Ai Ye, AY)	MOL005735	dammaradienyl acetate	44.833	0.835	DPNWLADVDGGIIN-QCUIPRAOSA-N	CC(C)CCCC(C)(C1CCC2(C1CCC3C2CCC4C3(CCC(C4(C)C)OC(=O)C)C)C)C	TCMSP
Folium Artemisiae Argyi (Ai Ye, AY)	MOL000098	quercetin	46.433	0.275	REFJWTPEDVJJIY-UHFFFAOYSA-N	C1=CC(=C(C=C1C2=C(C(=O)C3=C(C=C(C=C3O2)O)O)O)O)O	TCMSP
Polygonum Multiflorum (He Shou Wu, HSW)	MOL000358	beta-sitosterol	36.914	0.751	KZJWDPNRJALLNS-VJSFXXLFSA-N	CCC(CCC(C)C1CCC2C1(CCC3C2CC=C4C3(CCC(C4)O)C)C)C(C)C	TCMSP
Polygonum Multiflorum (He Shou Wu, HSW)	MOL000702	Guajavarin	29.650	0.703	PZZRDJXEMZMZFD-IEGSVRCHSA-N	C1C(C(C(O1)OC2=C(OC3=CC(=CC(=C3C2=O)O)O)C4=CC(=C(C=C4)O)O)O)O	TCMSP
Polygonum Multiflorum (He Shou Wu, HSW)	MOL001729	Crysophanol			LQGUBLBATBMXHT-UHFFFAOYSA-N	CC1=CC2=C(C(=C1)O)C(=O)C3=C(C2=O)C=CC=C3O	Taiwan@Database
Polygonum Multiflorum (He Shou Wu, HSW)	MOL000472	Emodin			RHMXXJGYXNZAPX-UHFFFAOYSA-N	CC1=CC2=C(C(=C1)O)C(=O)C3=C(C2=O)C=C(C=C3O)O	Taiwan@Database
Polygonum Multiflorum (He Shou Wu, HSW)	MOL008647	N-trans-Feruloyltyramine			NPNNKDMSXVRADT-WEVVVXLNSA-N	COC1=C(C=CC(=C1)C=CC(=O)NCCC2=CC=C(C=C2)O)O	Taiwan@Database
Polygonum Multiflorum (He Shou Wu, HSW)	MOL000476	Physcion			FFWOKTFYGVYKIR-UHFFFAOYSA-N	CC1=CC2=C(C(=C1)O)C(=O)C3=C(C2=O)C=C(C=C3O)OC	Taiwan@Database
Polygonum Multiflorum (He Shou Wu, HSW)	MOL013289	Piceid			HSTZMXCBWJGKHG-CUYWLFDKSA-N	C1=CC(=CC=C1C=CC2=CC(=CC(=C2)OC3C(C(C(C(O3)CO)O)O)O)O)O	Taiwan@Database
Polygonum Multiflorum (He Shou Wu, HSW)		Procyanidin B1 3'-O-gallate			BXWABJPTCUDBMM-NNJCKQBNSA-N	C1C(C(OC2=C1C(=CC(=C2C3C(C(OC4=CC(=CC(=C34)O)O)C5=CC(=C(C=C5)O)O)O	Taiwan@Database
Polygonum Multiflorum (He Shou Wu, HSW)	MOL006488	Questin			UUNPIWCQMVNINR-UHFFFAOYSA-N	CC1=CC2=C(C(=C1)O)C(=O)C3=C(C2=O)C=C(C=C3OC)O	Taiwan@Database
Polygonum Multiflorum (He Shou Wu, HSW)	MOL002268	Rhien			FCDLCPWAQCPTKC-UHFFFAOYSA-N	C1=CC2=C(C(=C1)O)C(=O)C3=C(C2=O)C=C(C=C3O)C(=O)O	Taiwan@Database
Polygonum Multiflorum (He Shou Wu, HSW)		1,3-Dihydroxy-6,7-dimethylxanthone-1-O-beta-D-glucoside			WMAOOSUVFZELAH-CMWLGVBASA-N	CC1=CC2=C(C=C1C)OC3=CC(=CC(=C3C2=O)OC4C(C(C(C(O4)CO)O)O)O)O	Taiwan@Database
Polygonum Multiflorum (He Shou Wu, HSW)		3,5-Dihydroxy-4'-methoxystilbene			NFDKQDQXIWHPF-UHFFFAOYSA-N	COC1=CC=C(C=C1)C=CC2=CC(=CC(=C2)O)O	Taiwan@Database
Colla Corii Asini (EJiao, EJ)	MOL000054	Arginine	47.642	0.032	ODKSFYDXXFIFQN-BYPYZUCNSA-N	C(CC(C(=O)O)N)CN=C(N)N	Martin S, Desai K. The effects of oral arginine on its metabolic pathways in Sprague-Dawley rats [J]. Br J Nutr, 2020, 123(2): 135-148.
Colla Corii Asini (EJiao, EJ)	MOL000071	Histidine	53.175	0.029	HNDVDQJCIGZPNO-YFKPBYRVSA-N	C1=C(NC=N1)CC(C(=O)O)N	FengRN,NiuYC,SunXW,etal.Histidine supplementation improves insulin resistance through suppressed inflammation in obese women with the metabolic syndrome: A randomised controlled trial [J]. Diabetologia, 2013, 56(5): 985-994.
Colla Corii Asini (EJiao, EJ)	MOL000055	Lysine	29.333	0.018	KDXKERNSBIXSRK-YFKPBYRVSA-N	C(CCN)CC(C(=O)O)N	Sato T, Ito Y, Nagasawa T. Regulation of skeletal muscle protein degradation and synthesis by oral administration of lysine in rats [J]. J Nutr Sci Vitaminol (Tokyo), 2013, 59(5): 412-419.
Colla Corii Asini (EJiao, EJ)	MOL003971	Threonine	73.516	0.013	AYFVYJQAPQTCCC-GBXIJSLDSA-N	CC(C(C(=O)O)N)O	Malinovsky A V. Reason for indispensability of threonine in humans and other mammals in comparative aspect [J]. Biochemistry (Mosc), 2017, 82(9): 1055-1060.<br>BATMAN-TCM
Colla Cornus Cervi (Lu Jiao Jiao, LJJ)		Calcium Phosphate			QORWJWZARLRLPR-UHFFFAOYSA-H	[O-]P(=O)([O-])[O-].[O-]P(=O)([O-])[O-].[Ca+2].[Ca+2].[Ca+2]	Wang, Y., Xu, C., Wang, P., Sun, S., Chen, J., Li, J., Chen, T. and Wang, J., 2011. Analysis and identification of different animal horns by a three-stage infrared spectroscopy. Spectrochimica Acta Part A: Molecular and Biomolecular Spectroscopy, 83(1), pp.265-270.
Colla Cornus Cervi (Lu Jiao Jiao, LJJ)		Calcium Carbonate			VTYYLEPIZMXCLO-UHFFFAOYSA-L	C(=O)([O-])[O-].[Ca+2]	Carter, O., Xu, Y. and Sadler, P., 2021. Minerals in biology and medicine. RSC Advances, 11(4), pp.1939-1951.<br>BATMAN-TCM
