# Supplementary material for: Network Pharmacology Approach for Predicting Targets of Zishen Yutai Pills on Premature Ovarian Insufficiency
Source: Evid Based Complement Alternat Med. 2021 Aug 4;2021:8215454. doi: 10.1155/2021/8215454 (PMC8357500; doi:10.1155/2021/8215454)
Supplement: Supplementary Materials — Supplementary Table 1: active compounds of each herb in ZSYTP. Supplementary Table 2: corresponding targets of active compounds: (A) two target genes of SDH; (B) 138 target genes of GQZ; (C) 202 target genes of TSZ; (D) 25 target genes of BJT; (E) 121 target genes of DS; (F) 11 target genes of BZ; (G) 283 target genes of DZ; (H) 13 target genes of SR; (J) 112 target genes of RS; (K) 76 target genes of SJS; (L) 115 target genes of AY; (M) 149 target genes of HSW; (N) 68 target genes of EJ; (P) 46 target genes of LJJ. Supplementary Table 3: POI targets. Supplementary Table 4: compounds and targets attributes of C-T network. Supplementary Table 5: data of compound-target network. Supplementary Table 6: analysed C-T network: (A) active compounds of C-T network and corresponding degrees; (B) targets of C-T network and corresponding degrees. Supplementary Table 7: ZSYTP core PPI network. Supplementary Table 8: gene ontology and KEGG analysis: (A) GO-BP analysis; (B) GO-MF analysis; (C) GO-CC analysis; (D) KEGG pathway enrichment analysis. [file ST2C TSZ Herb Targets.pdf › 8215454.f1/ST2C TSZ Herb Targets.pdf]

Supplementary Table S2C. Corresponding Targets of TSZ

Herb Name	Mol ID	Chemical Name	Protein Name	Symbol		Sources	
Cuscutae Semen (Tu Si Zi, TSZ)	MOL000358	Beta-sitosterol	DNA polymerase Beta	POLB	PubChem		
Cuscutae Semen (Tu Si Zi, TSZ)	MOL000358	Beta-sitosterol	glycogen synthase kinase 3 Beta	GSK3B	PubChem		
Cuscutae Semen (Tu Si Zi, TSZ)	MOL000358	Beta-sitosterol	protein kinase AMP-activated non-catalytic subun	PRKAB2	PubChem		
Cuscutae Semen (Tu Si Zi, TSZ)	MOL000358	Beta-sitosterol	protein kinase AMP-activated non-catalytic subun	PRKAG3	PubChem		
Cuscutae Semen (Tu Si Zi, TSZ)	MOL000358	Beta-sitosterol	protein kinase AMP-activated non-catalytic subun	PRKAG1	PubChem		
Cuscutae Semen (Tu Si Zi, TSZ)	MOL000358	Beta-sitosterol	protein kinase AMP-activated catalytic subunit alp	PRKAA1	PubChem		
Cuscutae Semen (Tu Si Zi, TSZ)	MOL000358	Beta-sitosterol	protein kinase AMP-activated catalytic subunit alp	PRKAA2	PubChem		
Cuscutae Semen (Tu Si Zi, TSZ)	MOL000358	Beta-sitosterol	protein kinase AMP-activated non-catalytic subun	PRKAB1	PubChem		
Cuscutae Semen (Tu Si Zi, TSZ)	MOL000358	Beta-sitosterol	protein kinase AMP-activated non-catalytic subun	PRKAG2	PubChem		
Cuscutae Semen (Tu Si Zi, TSZ)	MOL000358	Beta-sitosterol	dipeptidyl peptidase 4	DPP4	PubChem		
Cuscutae Semen (Tu Si Zi, TSZ)	MOL000358	Beta-sitosterol	coagulation factor II, thrombin	F2	PubChem		
Cuscutae Semen (Tu Si Zi, TSZ)	MOL000953	CLR	nuclear factor, erythroid 2 like 2	NFE2L2	PubChem		
Cuscutae Semen (Tu Si Zi, TSZ)	MOL000953	CLR	ATP binding cassette subfamily B member 1	ABCB1	PubChem		
Cuscutae Semen (Tu Si Zi, TSZ)	MOL000953	CLR	RAR related orphan receptor A	RORA	PubChem		
Cuscutae Semen (Tu Si Zi, TSZ)	MOL000953	CLR	RAR related orphan receptor C	RORC	PubChem		
Cuscutae Semen (Tu Si Zi, TSZ)	MOL000422	Kaempferol	cyclin-dependent kinase 1	CDK1	STITCH		
Cuscutae Semen (Tu Si Zi, TSZ)	MOL000422	Kaempferol	nuclear receptor subfamily 1, group I, member 2	NR1I2	STITCH		
Cuscutae Semen (Tu Si Zi, TSZ)	MOL000422	Kaempferol	UDP glycosyltransferase 3 family, polypeptide A1	UGT3A1	STITCH		
Cuscutae Semen (Tu Si Zi, TSZ)	MOL000422	Kaempferol	cytochrome P450, family 1, subfamily B, polypepti	CYP1B1	STITCH	Swiss Target Prediction	
Cuscutae Semen (Tu Si Zi, TSZ)	MOL000422	Kaempferol	ribosomal protein S6 kinase, 90kDa, polypeptide 3	RPS6KA3	STITCH		
Cuscutae Semen (Tu Si Zi, TSZ)	MOL000422	Kaempferol	aryl hydrocarbon receptor	AHR	STITCH	Swiss Target Prediction	
Cuscutae Semen (Tu Si Zi, TSZ)	MOL000422	Kaempferol	UDP glucuronosyltransferase 1 family, polypeptide	UGT1A3	STITCH		
Cuscutae Semen (Tu Si Zi, TSZ)	MOL000422	Kaempferol	UDP glucuronosyltransferase 1 family, polypeptide	UGT1A8	STITCH		
Cuscutae Semen (Tu Si Zi, TSZ)	MOL000422	Kaempferol	UDP glucuronosyltransferase 1 family, polypeptide	UGT1A7	STITCH		
Cuscutae Semen (Tu Si Zi, TSZ)	MOL000422	Kaempferol	UDP glucuronosyltransferase 1 family, polypeptide	UGT1A9	STITCH		
Cuscutae Semen (Tu Si Zi, TSZ)	MOL000422	Kaempferol	cytochrome P450 family 1 subfamily A member 2	CYP1A2	PubChem		
Cuscutae Semen (Tu Si Zi, TSZ)	MOL000422	Kaempferol	CDC like kinase 1	CLK1	PubChem		
Cuscutae Semen (Tu Si Zi, TSZ)	MOL000422	Kaempferol	serine/threonine kinase 16	STK16	PubChem		
Cuscutae Semen (Tu Si Zi, TSZ)	MOL000422	Kaempferol	Pim-2 proto-oncogene, serine/threonine kinase	PIM2	PubChem		
Cuscutae Semen (Tu Si Zi, TSZ)	MOL000422	Kaempferol	microtubule associated protein tau	MAPT	PubChem		
Cuscutae Semen (Tu Si Zi, TSZ)	MOL000422	Kaempferol	cytochrome P450 family 2 subfamily D member 6	CYP2D6	PubChem		
Cuscutae Semen (Tu Si Zi, TSZ)	MOL000422	Kaempferol	cytochrome P450 family 3 subfamily A member 4	CYP3A4	PubChem		
Cuscutae Semen (Tu Si Zi, TSZ)	MOL000422	Kaempferol	cytochrome P450 family 2 subfamily C member 9	CYP2C9	PubChem		
Cuscutae Semen (Tu Si Zi, TSZ)	MOL000422	Kaempferol	15-hydroxyprostaglandin dehydrogenase	HPGD	PubChem		
Cuscutae Semen (Tu Si Zi, TSZ)	MOL000422	Kaempferol	hypoxia inducible factor 1 subunit alpha	HIF1A	PubChem		
Cuscutae Semen (Tu Si Zi, TSZ)	MOL000422	Kaempferol	Cytochrome P450 2C19	CYP2C19	PubChem		
Cuscutae Semen (Tu Si Zi, TSZ)	MOL000422	Kaempferol	glycogen synthase kinase 3 alpha	GSK3A	PubChem		
Cuscutae Semen (Tu Si Zi, TSZ)	MOL000422	Kaempferol	glycogen synthase kinase 3 Beta	GSK3B	PubChem		
Cuscutae Semen (Tu Si Zi, TSZ)	MOL000422	Kaempferol	cyclin B2	CCNB2	PubChem		
Cuscutae Semen (Tu Si Zi, TSZ)	MOL000422	Kaempferol	cyclin B1	CCNB1	PubChem		
Cuscutae Semen (Tu Si Zi, TSZ)	MOL000422	Kaempferol	cyclin B3	CCNB3	PubChem		
Cuscutae Semen (Tu Si Zi, TSZ)	MOL000422	Kaempferol	cyclin dependent kinase 6	CDK6	PubChem		
Cuscutae Semen (Tu Si Zi, TSZ)	MOL000422	Kaempferol	arachidonate 5-lipoxygenase	ALOX5	PubChem	Swiss Target Prediction	
Cuscutae Semen (Tu Si Zi, TSZ)	MOL000422	Kaempferol	glyoxalase I	GLO1	PubChem		
Cuscutae Semen (Tu Si Zi, TSZ)	MOL000422	Kaempferol	xanthine dehydrogenase	XDH	PubChem	Swiss Target Prediction	
Cuscutae Semen (Tu Si Zi, TSZ)	MOL000422	Kaempferol	hydroxysteroid 17-Beta dehydrogenase 1	HSD17B1	PubChem		
Cuscutae Semen (Tu Si Zi, TSZ)	MOL000422	Kaempferol	hydroxysteroid 17-Beta dehydrogenase 2	HSD17B2	PubChem	Swiss Target Prediction	
Cuscutae Semen (Tu Si Zi, TSZ)	MOL000422	Kaempferol	ATP binding cassette subfamily C member 1	ABCC1	PubChem		
Cuscutae Semen (Tu Si Zi, TSZ)	MOL000422	Kaempferol	androgen receptor	AR	PubChem		
Cuscutae Semen (Tu Si Zi, TSZ)	MOL000422	Kaempferol	butyrylcholinesterase	BCHE	PubChem		
Cuscutae Semen (Tu Si Zi, TSZ)	MOL000422	Kaempferol	platelet activating factor acetylhydrolase 1b cataly	PAFAH1B3	PubChem		
Cuscutae Semen (Tu Si Zi, TSZ)	MOL000422	Kaempferol	CTD small phosphatase 1	CTDSP1	PubChem		
Cuscutae Semen (Tu Si Zi, TSZ)	MOL000422	Kaempferol	cytochrome P450 family 1 subfamily A member 1	CYP1A1	PubChem		
Cuscutae Semen (Tu Si Zi, TSZ)	MOL000422	Kaempferol	NADPH oxidase 4	NOX4	PubChem	Swiss Target Prediction	
Cuscutae Semen (Tu Si Zi, TSZ)	MOL000422	Kaempferol	ATP binding cassette subfamily G member 2 (Junic	ABCG2	PubChem		
Cuscutae Semen (Tu Si Zi, TSZ)	MOL000422	Kaempferol	estrogen receptor 1	ESR1	PubChem		
Cuscutae Semen (Tu Si Zi, TSZ)	MOL000422	Kaempferol	DNA polymerase iota	POLI	PubChem		
Cuscutae Semen (Tu Si Zi, TSZ)	MOL000422	Kaempferol	DNA polymerase eta	POLH	PubChem		
Cuscutae Semen (Tu Si Zi, TSZ)	MOL000422	Kaempferol	cholinergic receptor muscarinic 1	CHRM	PubChem		
Cuscutae Semen (Tu Si Zi, TSZ)	MOL000422	Kaempferol	ATP binding cassette subfamily B member 1	ABCB1	PubChem	Swiss Target Prediction	
Cuscutae Semen (Tu Si Zi, TSZ)	MOL000422	Kaempferol	Rac GTPase activating protein 1	RACGAP1	PubChem		
Cuscutae Semen (Tu Si Zi, TSZ)	MOL000422	Kaempferol	aldo-keto reductase family 1 member B	AKR1B1	PubChem	Swiss Target Prediction	
Cuscutae Semen (Tu Si Zi, TSZ)	MOL000422	Kaempferol	nuclear factor, erythroid 2 like 2	NFE2L2	PubChem		
Cuscutae Semen (Tu Si Zi, TSZ)	MOL000422	Kaempferol	phosphatidylinositol-5-phosphate 4-kinase type 2	PIP4K2A	PubChem		
Cuscutae Semen (Tu Si Zi, TSZ)	MOL000422	Kaempferol	ADAM metallopeptidase domain 10	ADAM10	PubChem		
Cuscutae Semen (Tu Si Zi, TSZ)	MOL000422	Kaempferol	ADAM metallopeptidase domain 17	ADAM17	PubChem		
Cuscutae Semen (Tu Si Zi, TSZ)	MOL000422	Kaempferol	nuclear receptor subfamily 3 group C member 1	NR3C1	PubChem		
Cuscutae Semen (Tu Si Zi, TSZ)	MOL000422	Kaempferol	fms related tyrosine kinase 3	FLT3	PubChem	Swiss Target Prediction	
Cuscutae Semen (Tu Si Zi, TSZ)	MOL000422	Kaempferol	peroxisome proliferator activated receptor gamma	PPARG	PubChem		
Cuscutae Semen (Tu Si Zi, TSZ)	MOL000422	Kaempferol	cytochrome P450 family 19 subfamily A member 1	CYP19A1	PubChem		
Cuscutae Semen (Tu Si Zi, TSZ)	MOL000422	Kaempferol	estrogen related receptor alpha	ESRRA	PubChem	Swiss Target Prediction	
Cuscutae Semen (Tu Si Zi, TSZ)	MOL000422	Kaempferol	sialic acid acetylesterase	SIAE	PubChem		
Cuscutae Semen (Tu Si Zi, TSZ)	MOL000422	Kaempferol	Beta-secretase 1	BACE1	PubChem		
Cuscutae Semen (Tu Si Zi, TSZ)	MOL000422	Kaempferol	retinoic acid receptor alpha	RARA	PubChem		
Cuscutae Semen (Tu Si Zi, TSZ)	MOL000422	Kaempferol	nuclear receptor subfamily 1 group I member 3	NR1I3	PubChem		
Cuscutae Semen (Tu Si Zi, TSZ)	MOL000422	Kaempferol	nuclear factor kappa B subunit 1	NFKB1	PubChem		
Cuscutae Semen (Tu Si Zi, TSZ)	MOL000422	Kaempferol	nuclear factor kappa B subunit 2	NFKB2	PubChem		
Cuscutae Semen (Tu Si Zi, TSZ)	MOL000422	Kaempferol	RELA proto-oncogene, NF-kB subunit	RELA	PubChem		
Cuscutae Semen (Tu Si Zi, TSZ)	MOL000422	Kaempferol	casein kinase 2 alpha 2	CSNK2A2	PubChem		
Cuscutae Semen (Tu Si Zi, TSZ)	MOL000422	Kaempferol	casein kinase 2 Beta	CSNK2B	PubChem		
Cuscutae Semen (Tu Si Zi, TSZ)	MOL000422	Kaempferol	casein kinase 2 alpha 1	CSNK2A1	PubChem		
Cuscutae Semen (Tu Si Zi, TSZ)	MOL000422	Kaempferol	death associated protein kinase 1	DAPK1	PubChem		
Cuscutae Semen (Tu Si Zi, TSZ)	MOL000422	Kaempferol	carbonic anhydrase 2	CA2	PubChem	Swiss Target Prediction	
Cuscutae Semen (Tu Si Zi, TSZ)	MOL000422	Kaempferol	carbonic anhydrase 7	CA7	PubChem	Swiss Target Prediction	
Cuscutae Semen (Tu Si Zi, TSZ)	MOL000422	Kaempferol	carbonic anhydrase 12	CA12	PubChem	Swiss Target Prediction	
Cuscutae Semen (Tu Si Zi, TSZ)	MOL000422	Kaempferol	histone deacetylase 9	HDAC9	PubChem		
Cuscutae Semen (Tu Si Zi, TSZ)	MOL000422	Kaempferol	GLI family zinc finger 3	GLI3	PubChem		
Cuscutae Semen (Tu Si Zi, TSZ)	MOL000422	Kaempferol	estrogen receptor 2	ESR2	PubChem		
Cuscutae Semen (Tu Si Zi, TSZ)	MOL000422	Kaempferol	amyloid Beta precursor protein	APP	PubChem		
Cuscutae Semen (Tu Si Zi, TSZ)	MOL000422	Kaempferol	CDGSH iron sulfur domain 1	CISD1	PubChem		
Cuscutae Semen (Tu Si Zi, TSZ)	MOL000422	Kaempferol	Tyrosinase	TYR	PubChem	Swiss Target Prediction	
Cuscutae Semen (Tu Si Zi, TSZ)	MOL000422	Kaempferol	Progesterone receptor	PGR	PubChem		
Cuscutae Semen (Tu Si Zi, TSZ)	MOL000422	Kaempferol	SMAD family member 2	SMAD2	PubChem		
Cuscutae Semen (Tu Si Zi, TSZ)	MOL000422	Kaempferol	SMAD family member 3	SMAD3	PubChem		
Cuscutae Semen (Tu Si Zi, TSZ)	MOL000422	Kaempferol	solute carrier family 2 member 1	SLC2A1	PubChem		
Cuscutae Semen (Tu Si Zi, TSZ)	MOL000422	Kaempferol	dipeptidyl peptidase 4	DPP4	PubChem		
Cuscutae Semen (Tu Si Zi, TSZ)	MOL000422	Kaempferol	myeloperoxidase	MPO	PubChem		
Cuscutae Semen (Tu Si Zi, TSZ)	MOL000422	Kaempferol	Adenosine A1 receptor (by homology)	ADORA1	Swiss Target Prediction		
Cuscutae Semen (Tu Si Zi, TSZ)	MOL000422	Kaempferol	Carbonic anhydrase IV	CA4	Swiss Target Prediction		
Cuscutae Semen (Tu Si Zi, TSZ)	MOL000098	Quercetin	Induced myeloid leukemia cell differentiation prot	MCL1	STITCH		
Cuscutae Semen (Tu Si Zi, TSZ)	MOL000098	Quercetin	Tyrosine-protein kinase HCK	HCK	STITCH		
Cuscutae Semen (Tu Si Zi, TSZ)	MOL000098	Quercetin	Solute carrier family 2, facilitated glucose transport	SLC2A2	STITCH		
Cuscutae Semen (Tu Si Zi, TSZ)	MOL000098	Quercetin	Cytochrome P450 2C8	CYP2C8	STITCH		
Cuscutae Semen (Tu Si Zi, TSZ)	MOL000098	Quercetin	ATP synthase subunit Beta, mitochondrial	ATP5B	STITCH		
Cuscutae Semen (Tu Si Zi, TSZ)	MOL000098	Quercetin	3-hydroxyisobutyryl-CoA hydrolase, mitochondrial	HIBCH	STITCH		
Cuscutae Semen (Tu Si Zi, TSZ)	MOL000098	Quercetin	serine/threonine kinase 17b	STK17B	STITCH		
Cuscutae Semen (Tu Si Zi, TSZ)	MOL000098	Quercetin	NADPH oxidase 4	NOX4	Swiss Target Prediction		
Cuscutae Semen (Tu Si Zi, TSZ)	MOL000098	Quercetin	Vasopressin V2 receptor	AVPR2	Swiss Target Prediction		
Cuscutae Semen (Tu Si Zi, TSZ)	MOL000098	Quercetin	Aldose reductase	AKR1B1	Swiss Target Prediction		
Cuscutae Semen (Tu Si Zi, TSZ)	MOL000098	Quercetin	Xanthine dehydrogenase	XDH	Swiss Target Prediction		
Cuscutae Semen (Tu Si Zi, TSZ)	MOL000098	Quercetin	Monoamine oxidase A	MAOA	Swiss Target Prediction		
Cuscutae Semen (Tu Si Zi, TSZ)	MOL000098	Quercetin	Insulin-like growth factor I receptor	IGF1R	Swiss Target Prediction		
Cuscutae Semen (Tu Si Zi, TSZ)	MOL000098	Quercetin	Tyrosine-protein kinase receptor FLT3	FLT3	Swiss Target Prediction		
Cuscutae Semen (Tu Si Zi, TSZ)	MOL000098	Quercetin	Cytochrome P450 19A1	CYP19A1	Swiss Target Prediction		
Cuscutae Semen (Tu Si Zi, TSZ)	MOL000098	Quercetin	Epidermal growth factor receptor erbB1	EGFR	Swiss Target Prediction		
Cuscutae Semen (Tu Si Zi, TSZ)	MOL000098	Quercetin	Thrombin	F2	Swiss Target Prediction		
Cuscutae Semen (Tu Si Zi, TSZ)	MOL000098	Quercetin	Carbonic anhydrase II	CA2	Swiss Target Prediction		
Cuscutae Semen (Tu Si Zi, TSZ)	MOL000098	Quercetin	Serine/threonine-protein kinase PIM1	PIM1	Swiss Target Prediction	STITCH	
Cuscutae Semen (Tu Si Zi, TSZ)	MOL000098	Quercetin	Arachidonate 5-lipoxygenase	ALOX5	Swiss Target Prediction		
Cuscutae Semen (Tu Si Zi, TSZ)	MOL000098	Quercetin	Serine/threonine-protein kinase Aurora-B	AURKB	Swiss Target Prediction		
Cuscutae Semen (Tu Si Zi, TSZ)	MOL000098	Quercetin	Dopamine D4 receptor	DRD4	Swiss Target Prediction		
Cuscutae Semen (Tu Si Zi, TSZ)	MOL000098	Quercetin	Adenosine A1 receptor (by homology)	ADORA1	Swiss Target Prediction		
Cuscutae Semen (Tu Si Zi, TSZ)	MOL000098	Quercetin	Carbonic anhydrase VII	CA7	Swiss Target Prediction		
Cuscutae Semen (Tu Si Zi, TSZ)	MOL000098	Quercetin	Glyoxalase I	GLO1	Swiss Target Prediction		
Cuscutae Semen (Tu Si Zi, TSZ)	MOL000098	Quercetin	Myeloperoxidase	MPO	Swiss Target Prediction		
Cuscutae Semen (Tu Si Zi, TSZ)	MOL000098	Quercetin	PI3-kinase p85-alpha subunit	PIK3R1	Swiss Target Prediction		
Cuscutae Semen (Tu Si Zi, TSZ)	MOL000098	Quercetin	Adenosine A2a receptor (by homology)	ADORA2A	Swiss Target Prediction		
Cuscutae Semen (Tu Si Zi, TSZ)	MOL000098	Quercetin	Death-associated protein kinase 1	DAPK1	Swiss Target Prediction		
Cuscutae Semen (Tu Si Zi, TSZ)	MOL000098	Quercetin	Liver glycogen phosphorylase	PYGL	Swiss Target Prediction		
Cuscutae Semen (Tu Si Zi, TSZ)	MOL000098	Quercetin	Carbonic anhydrase I	CA1	Swiss Target Prediction		
Cuscutae Semen (Tu Si Zi, TSZ)	MOL000098	Quercetin	Glycogen synthase kinase-3 Beta	GSK3B	Swiss Target Prediction		
Cuscutae Semen (Tu Si Zi, TSZ)	MOL000098	Quercetin	Tyrosine-protein kinase SRC	SRC	Swiss Target Prediction		
Cuscutae Semen (Tu Si Zi, TSZ)	MOL000098	Quercetin	Focal adhesion kinase 1	PTK2	Swiss Target Prediction		
Cuscutae Semen (Tu Si Zi, TSZ)	MOL000098	Quercetin	Estradiol 17-Beta-dehydrogenase 2	HSD17B2	Swiss Target Prediction		
Cuscutae Semen (Tu Si Zi, TSZ)	MOL000098	Quercetin	Vascular endothelial growth factor receptor 2	KDR	Swiss Target Prediction		
Cuscutae Semen (Tu Si Zi, TSZ)	MOL000098	Quercetin	Matrix metalloproteinase 13	MMP13	Swiss Target Prediction		
Cuscutae Semen (Tu Si Zi, TSZ)	MOL000098	Quercetin	Matrix metalloproteinase 3	MMP3	Swiss Target Prediction		
Cuscutae Semen (Tu Si Zi, TSZ)	MOL000098	Quercetin	Carbonic anhydrase III	CA3	Swiss Target Prediction		
Cuscutae Semen (Tu Si Zi, TSZ)	MOL000098	Quercetin	Arachidonate 15-lipoxygenase	ALOX15	Swiss Target Prediction		
Cuscutae Semen (Tu Si Zi, TSZ)	MOL000098	Quercetin	Multidrug resistance-associated protein 1	ABCC1	Swiss Target Prediction		
Cuscutae Semen (Tu Si Zi, TSZ)	MOL000098	Quercetin	Serine/threonine-protein kinase PLK1	PLK1	Swiss Target Prediction		
Cuscutae Semen (Tu Si Zi, TSZ)	MOL000098	Quercetin	Carbonic anhydrase VI	CA6	Swiss Target Prediction		
Cuscutae Semen (Tu Si Zi, TSZ)	MOL000098	Quercetin	Cyclin-dependent kinase 1	CDK1	Swiss Target Prediction		
Cuscutae Semen (Tu Si Zi, TSZ)	MOL000098	Quercetin	Matrix metalloproteinase 9	MMP9	Swiss Target Prediction		
Cuscutae Semen (Tu Si Zi, TSZ)	MOL000098	Quercetin	Carbonic anhydrase XII	CA12	Swiss Target Prediction		
Cuscutae Semen (Tu Si Zi, TSZ)	MOL000098	Quercetin	Matrix metalloproteinase 2	MMP2	Swiss Target Prediction		
Cuscutae Semen (Tu Si Zi, TSZ)	MOL000098	Quercetin	Protein kinase N1	PKN1	Swiss Target Prediction		
Cuscutae Semen (Tu Si Zi, TSZ)	MOL000098	Quercetin	Carbonic anhydrase XIV	CA14	Swiss Target Prediction		
Cuscutae Semen (Tu Si Zi, TSZ)	MOL000098	Quercetin	Carbonic anhydrase IX	CA9	Swiss Target Prediction		
Cuscutae Semen (Tu Si Zi, TSZ)	MOL000098	Quercetin	Casein kinase II alpha	CSNK2A1	Swiss Target Prediction		
Cuscutae Semen (Tu Si Zi, TSZ)	MOL000098	Quercetin	Arachidonate 12-lipoxygenase	ALOX12	Swiss Target Prediction		
Cuscutae Semen (Tu Si Zi, TSZ)	MOL000098	Quercetin	Hepatocyte growth factor receptor	MET	Swiss Target Prediction		
Cuscutae Semen (Tu Si Zi, TSZ)	MOL000098	Quercetin	Carbonic anhydrase IV	CA4	Swiss Target Prediction		
Cuscutae Semen (Tu Si Zi, TSZ)	MOL000098	Quercetin	Serine/threonine-protein kinase NEK2	NEK2	Swiss Target Prediction		
Cuscutae Semen (Tu Si Zi, TSZ)	MOL000098	Quercetin	Interleukin-8 receptor A	CXCR1	Swiss Target Prediction		
Cuscutae Semen (Tu Si Zi, TSZ)	MOL000098	Quercetin	CaM kinase II Beta	CAMK2B	Swiss Target Prediction		
Cuscutae Semen (Tu Si Zi, TSZ)	MOL000098	Quercetin	ALK tyrosine kinase receptor	ALK	Swiss Target Prediction		
Cuscutae Semen (Tu Si Zi, TSZ)	MOL000098	Quercetin	Serine/threonine-protein kinase AKT	AKT1	Swiss Target Prediction		
Cuscutae Semen (Tu Si Zi, TSZ)	MOL000098	Quercetin	P-glycoprotein 1	ABCB1	Swiss Target Prediction		
Cuscutae Semen (Tu Si Zi, TSZ)	MOL000098	Quercetin	Serine/threonine-protein kinase NEK6	NEK6	Swiss Target Prediction		
Cuscutae Semen (Tu Si Zi, TSZ)	MOL000098	Quercetin	Phospholipase A2 group 1B	PLA2G1B	Swiss Target Prediction		
Cuscutae Semen (Tu Si Zi, TSZ)	MOL000098	Quercetin	Carbonic anhydrase VA	CA5A	Swiss Target Prediction		
Cuscutae Semen (Tu Si Zi, TSZ)	MOL000098	Quercetin	Beta-secretase 1	BACE1	Swiss Target Prediction		
Cuscutae Semen (Tu Si Zi, TSZ)	MOL000098	Quercetin	Cytochrome P450 1B1	CYP1B1	Swiss Target Prediction	STITCH	
Cuscutae Semen (Tu Si Zi, TSZ)	MOL000098	Quercetin	Tyrosine-protein kinase receptor UFO	AXL	Swiss Target Prediction		
Cuscutae Semen (Tu Si Zi, TSZ)	MOL000098	Quercetin	ATP-binding cassette sub-family G member 2	ABCG2	Swiss Target Prediction		
Cuscutae Semen (Tu Si Zi, TSZ)	MOL000098	Quercetin	NUAK family SNF1-like kinase 1	NUAK1	Swiss Target Prediction		
Cuscutae Semen (Tu Si Zi, TSZ)	MOL000098	Quercetin	Aldo-keto reductase family 1 member C2 (by hon	AKR1C2	Swiss Target Prediction		
Cuscutae Semen (Tu Si Zi, TSZ)	MOL000098	Quercetin	Aldo-keto reductase family 1 member C1 (by hon	AKR1C1	Swiss Target Prediction		
Cuscutae Semen (Tu Si Zi, TSZ)	MOL000098	Quercetin	Aldo-keto-reductase family 1 member C3 (by hon	AKR1C3	Swiss Target Prediction		
Cuscutae Semen (Tu Si Zi, TSZ)	MOL000098	Quercetin	Aldo-keto reductase family 1 member C4 (by hon	AKR1C4	Swiss Target Prediction		
Cuscutae Semen (Tu Si Zi, TSZ)	MOL000098	Quercetin	Carbonic anhydrase XIII (by homology)	CA13	Swiss Target Prediction		
Cuscutae Semen (Tu Si Zi, TSZ)	MOL000098	Quercetin	Aldehyde reductase (by homology)	AKR1A1	Swiss Target Prediction		
Cuscutae Semen (Tu Si Zi, TSZ)	MOL000098	Quercetin	G-protein coupled receptor 35	GPR35	Swiss Target Prediction		
Cuscutae Semen (Tu Si Zi, TSZ)	MOL000098	Quercetin	Tyrosine-protein kinase SYK	SYK	Swiss Target Prediction		
Cuscutae Semen (Tu Si Zi, TSZ)	MOL000354	Isorhamnetin	cytochrome P450, family 1, subfamily B, polypepti	CYP1B1	STITCH	PubChem	iss Target Prediction
Cuscutae Semen (Tu Si Zi, TSZ)	MOL000354	Isorhamnetin	cytochrome P450, family 1, subfamily A, polypepti	CYP1A1	STITCH	PubChem	
Cuscutae Semen (Tu Si Zi, TSZ)	MOL000354	Isorhamnetin	mitogen-activated protein kinase 8	MAPK8	STITCH		
Cuscutae Semen (Tu Si Zi, TSZ)	MOL000354	Isorhamnetin	v-akt murine thymoma viral oncogene homolog 1	AKT1	STITCH		
Cuscutae Semen (Tu Si Zi, TSZ)	MOL000354	Isorhamnetin	paraoxonase 2 (354 aa)	PON2	STITCH		
Cuscutae Semen (Tu Si Zi, TSZ)	MOL000354	Isorhamnetin	heme oxygenase (decycling) 1	HMOX1	STITCH		
Cuscutae Semen (Tu Si Zi, TSZ)	MOL000354	Isorhamnetin	mitogen-activated protein kinase 9	MAPK9	STITCH		
Cuscutae Semen (Tu Si Zi, TSZ)	MOL000354	Isorhamnetin	nitric oxide synthase 2, inducible (1153 aa)	NOS2	STITCH		
Cuscutae Semen (Tu Si Zi, TSZ)	MOL000354	Isorhamnetin	ATP-binding cassette, sub-family C (CFTR/MRP), m	ABCC1	STITCH		
Cuscutae Semen (Tu Si Zi, TSZ)	MOL000354	Isorhamnetin	protein kinase C, delta;	PRKCD	STITCH		
Cuscutae Semen (Tu Si Zi, TSZ)	MOL000354	Isorhamnetin	xanthine dehydrogenase	XDH	PubChem	Swiss Target Prediction	
Cuscutae Semen (Tu Si Zi, TSZ)	MOL000354	Isorhamnetin	Cytochrome P450 1A2	CYP1A2	PubChem		
Cuscutae Semen (Tu Si Zi, TSZ)	MOL000354	Isorhamnetin	Nuclear factor NF-kappa-B p105 subunit	NFKB1	PubChem		
Cuscutae Semen (Tu Si Zi, TSZ)	MOL000354	Isorhamnetin	Nuclear Factor Kappa B Subunit 2	NFKB2	PubChem		
Cuscutae Semen (Tu Si Zi, TSZ)	MOL000354	Isorhamnetin	v-rel reticuloendotheliosis viral oncogene homolog	RELA	PubChem		
Cuscutae Semen (Tu Si Zi, TSZ)	MOL000354	Isorhamnetin	Carbonic anhydrase 2	CA2	PubChem	Swiss Target Prediction	
Cuscutae Semen (Tu Si Zi, TSZ)	MOL000354	Isorhamnetin	Carbonic anhydrase 7	CA7	PubChem	Swiss Target Prediction	
Cuscutae Semen (Tu Si Zi, TSZ)	MOL000354	Isorhamnetin	Carbonic anhydrase IV	CA4	PubChem	Swiss Target Prediction	
Cuscutae Semen (Tu Si Zi, TSZ)	MOL000354	Isorhamnetin	Carbonic anhydrase 12	CA12	PubChem	Swiss Target Prediction	
Cuscutae Semen (Tu Si Zi, TSZ)	MOL001558	Sesamin	TAR DNA binding protein	TARDBP	PubChem		
Cuscutae Semen (Tu Si Zi, TSZ)	MOL001558	Sesamin	cytochrome P450 family 2 subfamily C member 9	CYP2C9	PubChem		
Cuscutae Semen (Tu Si Zi, TSZ)	MOL001558	Sesamin	cytochrome P450 family 1 subfamily A member 2	CYP1A2	PubChem		
Cuscutae Semen (Tu Si Zi, TSZ)	MOL001558	Sesamin	Induced myeloid leukemia cell differentiation prot	MCL1	Swiss Target Prediction		
Cuscutae Semen (Tu Si Zi, TSZ)	MOL005944	Matrine	phosphatase and tensin homolog	PTEN	STITCH		
Cuscutae Semen (Tu Si Zi, TSZ)	MOL005944	Matrine	potassium voltage-gated channel, subfamily H (ea	KCNH2	STITCH		
Cuscutae Semen (Tu Si Zi, TSZ)	MOL005944	Matrine	matrix metallopeptidase 9 (gelatinase B, 92kDa ge	MMP9	STITCH		
Cuscutae Semen (Tu Si Zi, TSZ)	MOL005944	Matrine	cyclin D1	CCND1	STITCH		
