# Supplementary material for: Network Pharmacology Approach for Predicting Targets of Zishen Yutai Pills on Premature Ovarian Insufficiency
Source: Evid Based Complement Alternat Med. 2021 Aug 4;2021:8215454. doi: 10.1155/2021/8215454 (PMC8357500; doi:10.1155/2021/8215454)
Supplement: Supplementary Materials — Supplementary Table 1: active compounds of each herb in ZSYTP. Supplementary Table 2: corresponding targets of active compounds: (A) two target genes of SDH; (B) 138 target genes of GQZ; (C) 202 target genes of TSZ; (D) 25 target genes of BJT; (E) 121 target genes of DS; (F) 11 target genes of BZ; (G) 283 target genes of DZ; (H) 13 target genes of SR; (J) 112 target genes of RS; (K) 76 target genes of SJS; (L) 115 target genes of AY; (M) 149 target genes of HSW; (N) 68 target genes of EJ; (P) 46 target genes of LJJ. Supplementary Table 3: POI targets. Supplementary Table 4: compounds and targets attributes of C-T network. Supplementary Table 5: data of compound-target network. Supplementary Table 6: analysed C-T network: (A) active compounds of C-T network and corresponding degrees; (B) targets of C-T network and corresponding degrees. Supplementary Table 7: ZSYTP core PPI network. Supplementary Table 8: gene ontology and KEGG analysis: (A) GO-BP analysis; (B) GO-MF analysis; (C) GO-CC analysis; (D) KEGG pathway enrichment analysis. [file ST2G DZ Herb Targets.pdf › 8215454.f1/ST2G DZ Herb Targets.pdf]

Supplementary Table S2G. Corresponding Targets of DS

Herb Name	Mol ID	Chemical Name	Protein Name	Symbol	Sources	
Eucommiae Cortex (Du Zhong, DZ)	MOL000211	Mairin	ubiquitin like modifier activating enzyme 2	UBA2	Pubchem	
Eucommiae Cortex (Du Zhong, DZ)	MOL000211	Mairin	SUMO1 activating enzyme subunit 1	SAE1	Pubchem	
Eucommiae Cortex (Du Zhong, DZ)	MOL000211	Mairin	ubiquitin conjugating enzyme E2 I	UBE2I	Pubchem	
Eucommiae Cortex (Du Zhong, DZ)	MOL000211	Mairin	serine/threonine kinase 33	STK33	Pubchem	
Eucommiae Cortex (Du Zhong, DZ)	MOL000211	Mairin	DNA polymerase beta	POLB	Pubchem	
Eucommiae Cortex (Du Zhong, DZ)	MOL000211	Mairin	nuclear receptor subfamily 1 group H member 3	NR1H3	Pubchem	
Eucommiae Cortex (Du Zhong, DZ)	MOL000211	Mairin	Glycogen synthase kinase-3 beta	GSK3B	Pubchem	
Eucommiae Cortex (Du Zhong, DZ)	MOL000211	Mairin	G protein-coupled bile acid receptor 1	GPBAR1	Pubchem	
Eucommiae Cortex (Du Zhong, DZ)	MOL000211	Mairin	protein kinase AMP-activated non-catalytic subunit b	PRKAB2	Pubchem	
Eucommiae Cortex (Du Zhong, DZ)	MOL000211	Mairin	protein kinase AMP-activated non-catalytic subunit g	PRKAG3	Pubchem	
Eucommiae Cortex (Du Zhong, DZ)	MOL000211	Mairin	protein kinase AMP-activated non-catalytic subunit g	PRKAG1	Pubchem	
Eucommiae Cortex (Du Zhong, DZ)	MOL000211	Mairin	protein kinase AMP-activated catalytic subunit alpha	PRKAA1	Pubchem	
Eucommiae Cortex (Du Zhong, DZ)	MOL000211	Mairin	protein kinase AMP-activated catalytic subunit alpha	PRKAA2	Pubchem	
Eucommiae Cortex (Du Zhong, DZ)	MOL000211	Mairin	protein kinase AMP-activated non-catalytic subunit b	PRKAB1	Pubchem	
Eucommiae Cortex (Du Zhong, DZ)	MOL000211	Mairin	protein kinase AMP-activated non-catalytic subunit g	PRKAG2	Pubchem	
Eucommiae Cortex (Du Zhong, DZ)	MOL000211	Mairin	MDM2 proto-oncogene	MDM2	Pubchem	
Eucommiae Cortex (Du Zhong, DZ)	MOL000211	Mairin	MDM4 regulator of p53	MDM4	Pubchem	
Eucommiae Cortex (Du Zhong, DZ)	MOL000211	Mairin	dopamine receptor D2	DRD2	Pubchem	
Eucommiae Cortex (Du Zhong, DZ)	MOL000211	Mairin	DNA (cytosine-5-)-methyltransferase 1	DNMT1	Pubchem	
Eucommiae Cortex (Du Zhong, DZ)	MOL000211	Mairin	cystic fibrosis transmembrane conductance regulator	CFTR	Pubchem	
Eucommiae Cortex (Du Zhong, DZ)	MOL000211	Mairin	tyrosyl-DNA phosphodiesterase 1	TDP1	Pubchem	
Eucommiae Cortex (Du Zhong, DZ)	MOL000211	Mairin	aldo-keto reductase family 1 member B10	AKR1B10	Pubchem	
Eucommiae Cortex (Du Zhong, DZ)	MOL000211	Mairin	aldo-keto reductase family 1 member B	AKR1B1	Pubchem	
Eucommiae Cortex (Du Zhong, DZ)	MOL000211	Mairin	albumin	ALB	Pubchem	
Eucommiae Cortex (Du Zhong, DZ)	MOL000211	Mairin	interferon gamma	IFNG	Pubchem	
Eucommiae Cortex (Du Zhong, DZ)	MOL000211	Mairin	DNA polymerase beta (by homology)	POLB	Swiss Target Prediction	
Eucommiae Cortex (Du Zhong, DZ)	MOL000211	Mairin	Aldo-keto reductase family 1 member B10	AKR1B10	Swiss Target Prediction	
Eucommiae Cortex (Du Zhong, DZ)	MOL000211	Mairin	SUMO-activating enzyme	SAE1 UBA2	Swiss Target Prediction	
Eucommiae Cortex (Du Zhong, DZ)	MOL000358	Beta-sitosterol	DNA polymerase beta	POLB	PubChem	
Eucommiae Cortex (Du Zhong, DZ)	MOL000358	Beta-sitosterol	glycogen synthase kinase 3 beta	GSK3B	PubChem	
Eucommiae Cortex (Du Zhong, DZ)	MOL000358	Beta-sitosterol	protein kinase AMP-activated non-catalytic subunit b	PRKAB2	PubChem	
Eucommiae Cortex (Du Zhong, DZ)	MOL000358	Beta-sitosterol	protein kinase AMP-activated non-catalytic subunit g	PRKAG3	PubChem	
Eucommiae Cortex (Du Zhong, DZ)	MOL000358	Beta-sitosterol	protein kinase AMP-activated non-catalytic subunit g	PRKAG1	PubChem	
Eucommiae Cortex (Du Zhong, DZ)	MOL000358	Beta-sitosterol	protein kinase AMP-activated catalytic subunit alpha	PRKAA1	PubChem	
Eucommiae Cortex (Du Zhong, DZ)	MOL000358	Beta-sitosterol	protein kinase AMP-activated catalytic subunit alpha	PRKAA2	PubChem	
Eucommiae Cortex (Du Zhong, DZ)	MOL000358	Beta-sitosterol	protein kinase AMP-activated non-catalytic subunit b	PRKAB1	PubChem	
Eucommiae Cortex (Du Zhong, DZ)	MOL000358	Beta-sitosterol	protein kinase AMP-activated non-catalytic subunit g	PRKAG2	PubChem	
Eucommiae Cortex (Du Zhong, DZ)	MOL000358	Beta-sitosterol	dipeptidyl peptidase 4	DPP4	PubChem	
Eucommiae Cortex (Du Zhong, DZ)	MOL000358	Beta-sitosterol	coagulation factor II, thrombin	F2	PubChem	
Eucommiae Cortex (Du Zhong, DZ)	MOL000422	Kaempferol	cyclin-dependent kinase 1	CDK1	STITCH	
Eucommiae Cortex (Du Zhong, DZ)	MOL000422	Kaempferol	nuclear receptor subfamily 1, group I, member 2	NR1I2	STITCH	
Eucommiae Cortex (Du Zhong, DZ)	MOL000422	Kaempferol	UDP glycosyltransferase 3 family, polypeptide A1	UGT3A1	STITCH	
Eucommiae Cortex (Du Zhong, DZ)	MOL000422	Kaempferol	cytochrome P450, family 1, subfamily B, polypeptide	CYP1B1	STITCH	iss Target Prediction
Eucommiae Cortex (Du Zhong, DZ)	MOL000422	Kaempferol	ribosomal protein S6 kinase, 90kDa, polypeptide 3;	RPS6KA3	STITCH	
Eucommiae Cortex (Du Zhong, DZ)	MOL000422	Kaempferol	aryl hydrocarbon receptor	AHR	STITCH	iss Target Prediction
Eucommiae Cortex (Du Zhong, DZ)	MOL000422	Kaempferol	UDP glucuronosyltransferase 1 family, polypeptide A	UGT1A3	STITCH	
Eucommiae Cortex (Du Zhong, DZ)	MOL000422	Kaempferol	UDP glucuronosyltransferase 1 family, polypeptide A	UGT1A8	STITCH	
Eucommiae Cortex (Du Zhong, DZ)	MOL000422	Kaempferol	UDP glucuronosyltransferase 1 family, polypeptide A	UGT1A7	STITCH	
Eucommiae Cortex (Du Zhong, DZ)	MOL000422	Kaempferol	UDP glucuronosyltransferase 1 family, polypeptide A	UGT1A9	STITCH	
Eucommiae Cortex (Du Zhong, DZ)	MOL000422	Kaempferol	cytochrome P450 family 1 subfamily A member 2	CYP1A2	PubChem	
Eucommiae Cortex (Du Zhong, DZ)	MOL000422	Kaempferol	CDC like kinase 1	CLK1	PubChem	
Eucommiae Cortex (Du Zhong, DZ)	MOL000422	Kaempferol	serine/threonine kinase 16	STK16	PubChem	
Eucommiae Cortex (Du Zhong, DZ)	MOL000422	Kaempferol	Pim-2 proto-oncogene, serine/threonine kinase	PIM2	PubChem	
Eucommiae Cortex (Du Zhong, DZ)	MOL000422	Kaempferol	microtubule associated protein tau	MAPT	PubChem	
Eucommiae Cortex (Du Zhong, DZ)	MOL000422	Kaempferol	cytochrome P450 family 2 subfamily D member 6	CYP2D6	PubChem	
Eucommiae Cortex (Du Zhong, DZ)	MOL000422	Kaempferol	cytochrome P450 family 3 subfamily A member 4	CYP3A4	PubChem	
Eucommiae Cortex (Du Zhong, DZ)	MOL000422	Kaempferol	cytochrome P450 family 2 subfamily C member 9	CYP2C9	PubChem	
Eucommiae Cortex (Du Zhong, DZ)	MOL000422	Kaempferol	15-hydroxyprostaglandin dehydrogenase	HPGD	PubChem	
Eucommiae Cortex (Du Zhong, DZ)	MOL000422	Kaempferol	hypoxia inducible factor 1 subunit alpha	HIF1A	PubChem	
Eucommiae Cortex (Du Zhong, DZ)	MOL000422	Kaempferol	Cytochrome P450 2C19	CYP2C19	PubChem	
Eucommiae Cortex (Du Zhong, DZ)	MOL000422	Kaempferol	glycogen synthase kinase 3 alpha	GSK3A	PubChem	
Eucommiae Cortex (Du Zhong, DZ)	MOL000422	Kaempferol	glycogen synthase kinase 3 Beta	GSK3B	PubChem	
Eucommiae Cortex (Du Zhong, DZ)	MOL000422	Kaempferol	cyclin B2	CCNB2	PubChem	
Eucommiae Cortex (Du Zhong, DZ)	MOL000422	Kaempferol	cyclin B1	CCNB1	PubChem	
Eucommiae Cortex (Du Zhong, DZ)	MOL000422	Kaempferol	cyclin B3	CCNB3	PubChem	
Eucommiae Cortex (Du Zhong, DZ)	MOL000422	Kaempferol	cyclin dependent kinase 6	CDK6	PubChem	
Eucommiae Cortex (Du Zhong, DZ)	MOL000422	Kaempferol	arachidonate 5-lipoxygenase	ALOX5	PubChem	iss Target Prediction
Eucommiae Cortex (Du Zhong, DZ)	MOL000422	Kaempferol	glyoxalase I	GLO1	PubChem	
Eucommiae Cortex (Du Zhong, DZ)	MOL000422	Kaempferol	xanthine dehydrogenase	XDH	PubChem	iss Target Prediction
Eucommiae Cortex (Du Zhong, DZ)	MOL000422	Kaempferol	hydroxysteroid 17-Beta dehydrogenase 1	HSD17B1	PubChem	
Eucommiae Cortex (Du Zhong, DZ)	MOL000422	Kaempferol	hydroxysteroid 17-Beta dehydrogenase 2	HSD17B2	PubChem	iss Target Prediction
Eucommiae Cortex (Du Zhong, DZ)	MOL000422	Kaempferol	ATP binding cassette subfamily C member 1	ABCC1	PubChem	
Eucommiae Cortex (Du Zhong, DZ)	MOL000422	Kaempferol	androgen receptor	AR	PubChem	
Eucommiae Cortex (Du Zhong, DZ)	MOL000422	Kaempferol	butyrylcholinesterase	BCHE	PubChem	
Eucommiae Cortex (Du Zhong, DZ)	MOL000422	Kaempferol	platelet activating factor acetylhydrolase 1b catalytic	PAFAH1B3	PubChem	
Eucommiae Cortex (Du Zhong, DZ)	MOL000422	Kaempferol	CTD small phosphatase 1	CTDSP1	PubChem	
Eucommiae Cortex (Du Zhong, DZ)	MOL000422	Kaempferol	cytochrome P450 family 1 subfamily A member 1	CYP1A1	PubChem	
Eucommiae Cortex (Du Zhong, DZ)	MOL000422	Kaempferol	NADPH oxidase 4	NOX4	PubChem	iss Target Prediction
Eucommiae Cortex (Du Zhong, DZ)	MOL000422	Kaempferol	ATP binding cassette subfamily G member 2 (Junior b	ABCG2	PubChem	
Eucommiae Cortex (Du Zhong, DZ)	MOL000422	Kaempferol	estrogen receptor 1	ESR1	PubChem	
Eucommiae Cortex (Du Zhong, DZ)	MOL000422	Kaempferol	DNA polymerase iota	POLI	PubChem	
Eucommiae Cortex (Du Zhong, DZ)	MOL000422	Kaempferol	DNA polymerase eta	POLH	PubChem	
Eucommiae Cortex (Du Zhong, DZ)	MOL000422	Kaempferol	cholinergic receptor muscarinic 1	CHRM	PubChem	
Eucommiae Cortex (Du Zhong, DZ)	MOL000422	Kaempferol	ATP binding cassette subfamily B member 1	ABCB1	PubChem	iss Target Prediction
Eucommiae Cortex (Du Zhong, DZ)	MOL000422	Kaempferol	Rac GTPase activating protein 1	RACGAP1	PubChem	
Eucommiae Cortex (Du Zhong, DZ)	MOL000422	Kaempferol	aldo-keto reductase family 1 member B	AKR1B1	PubChem	iss Target Prediction
Eucommiae Cortex (Du Zhong, DZ)	MOL000422	Kaempferol	nuclear factor, erythroid 2 like 2	NFE2L2	PubChem	
Eucommiae Cortex (Du Zhong, DZ)	MOL000422	Kaempferol	phosphatidylinositol-5-phosphate 4-kinase type 2 alp	PIP4K2A	PubChem	
Eucommiae Cortex (Du Zhong, DZ)	MOL000422	Kaempferol	ADAM metallopeptidase domain 10	ADAM10	PubChem	
Eucommiae Cortex (Du Zhong, DZ)	MOL000422	Kaempferol	ADAM metallopeptidase domain 17	ADAM17	PubChem	
Eucommiae Cortex (Du Zhong, DZ)	MOL000422	Kaempferol	nuclear receptor subfamily 3 group C member 1	NR3C1	PubChem	
Eucommiae Cortex (Du Zhong, DZ)	MOL000422	Kaempferol	fms related tyrosine kinase 3	FLT3	PubChem	iss Target Prediction
Eucommiae Cortex (Du Zhong, DZ)	MOL000422	Kaempferol	peroxisome proliferator activated receptor gamma	PPARG	PubChem	
Eucommiae Cortex (Du Zhong, DZ)	MOL000422	Kaempferol	cytochrome P450 family 19 subfamily A member 1	CYP19A1	PubChem	
Eucommiae Cortex (Du Zhong, DZ)	MOL000422	Kaempferol	estrogen related receptor alpha	ESRRA	PubChem	iss Target Prediction
Eucommiae Cortex (Du Zhong, DZ)	MOL000422	Kaempferol	sialic acid acetylesterase	SIAE	PubChem	
Eucommiae Cortex (Du Zhong, DZ)	MOL000422	Kaempferol	Beta-secretase 1	BACE1	PubChem	
Eucommiae Cortex (Du Zhong, DZ)	MOL000422	Kaempferol	retinoic acid receptor alpha	RARA	PubChem	
Eucommiae Cortex (Du Zhong, DZ)	MOL000422	Kaempferol	nuclear receptor subfamily 1 group I member 3	NR1I3	PubChem	
Eucommiae Cortex (Du Zhong, DZ)	MOL000422	Kaempferol	nuclear factor kappa B subunit 1	NFKB1	PubChem	
Eucommiae Cortex (Du Zhong, DZ)	MOL000422	Kaempferol	nuclear factor kappa B subunit 2	NFKB2	PubChem	
Eucommiae Cortex (Du Zhong, DZ)	MOL000422	Kaempferol	RELA proto-oncogene, NF-kB subunit	RELA	PubChem	
Eucommiae Cortex (Du Zhong, DZ)	MOL000422	Kaempferol	casein kinase 2 alpha 2	CSNK2A2	PubChem	
Eucommiae Cortex (Du Zhong, DZ)	MOL000422	Kaempferol	casein kinase 2 Beta	CSNK2B	PubChem	
Eucommiae Cortex (Du Zhong, DZ)	MOL000422	Kaempferol	casein kinase 2 alpha 1	CSNK2A1	PubChem	
Eucommiae Cortex (Du Zhong, DZ)	MOL000422	Kaempferol	death associated protein kinase 1	DAPK1	PubChem	
Eucommiae Cortex (Du Zhong, DZ)	MOL000422	Kaempferol	carbonic anhydrase 2	CA2	PubChem	iss Target Prediction
Eucommiae Cortex (Du Zhong, DZ)	MOL000422	Kaempferol	carbonic anhydrase 7	CA7	PubChem	iss Target Prediction
Eucommiae Cortex (Du Zhong, DZ)	MOL000422	Kaempferol	carbonic anhydrase 12	CA12	PubChem	iss Target Prediction
Eucommiae Cortex (Du Zhong, DZ)	MOL000422	Kaempferol	histone deacetylase 9	HDAC9	PubChem	
Eucommiae Cortex (Du Zhong, DZ)	MOL000422	Kaempferol	GLI family zinc finger 3	GLI3	PubChem	
Eucommiae Cortex (Du Zhong, DZ)	MOL000422	Kaempferol	estrogen receptor 2	ESR2	PubChem	
Eucommiae Cortex (Du Zhong, DZ)	MOL000422	Kaempferol	amyloid Beta precursor protein	APP	PubChem	
Eucommiae Cortex (Du Zhong, DZ)	MOL000422	Kaempferol	CDGSH iron sulfur domain 1	CISD1	PubChem	
Eucommiae Cortex (Du Zhong, DZ)	MOL000422	Kaempferol	Tyrosinase	TYR	PubChem	iss Target Prediction
Eucommiae Cortex (Du Zhong, DZ)	MOL000422	Kaempferol	Progesterone receptor	PGR	PubChem	
Eucommiae Cortex (Du Zhong, DZ)	MOL000422	Kaempferol	SMAD family member 2	SMAD2	PubChem	
Eucommiae Cortex (Du Zhong, DZ)	MOL000422	Kaempferol	SMAD family member 3	SMAD3	PubChem	
Eucommiae Cortex (Du Zhong, DZ)	MOL000422	Kaempferol	solute carrier family 2 member 1	SLC2A1	PubChem	
Eucommiae Cortex (Du Zhong, DZ)	MOL000422	Kaempferol	dipeptidyl peptidase 4	DPP4	PubChem	
Eucommiae Cortex (Du Zhong, DZ)	MOL000422	Kaempferol	myeloperoxidase	MPO	PubChem	
Eucommiae Cortex (Du Zhong, DZ)	MOL000422	Kaempferol	Adenosine A1 receptor (by homology)	ADORA1	Swiss Target Prediction	
Eucommiae Cortex (Du Zhong, DZ)	MOL000422	Kaempferol	Carbonic anhydrase IV	CA4	Swiss Target Prediction	
Eucommiae Cortex (Du Zhong, DZ)	MOL000073	ent-Epicatechin	heme oxygenase (decycling) 1	HMOX1	STITCH	
Eucommiae Cortex (Du Zhong, DZ)	MOL000073	ent-Epicatechin	DNA (cytosine-5-)-methyltransferase 1	DNMT1	STITCH	
Eucommiae Cortex (Du Zhong, DZ)	MOL000073	ent-Epicatechin	prostaglandin-endoperoxide synthase 2 (prostagland	PTGS2	STITCH	
Eucommiae Cortex (Du Zhong, DZ)	MOL000073	ent-Epicatechin	solute carrier family 47, member 1	SLC47A1	STITCH	
Eucommiae Cortex (Du Zhong, DZ)	MOL000073	ent-Epicatechin	apolipoprotein B (including Ag(x) antigen) (4563 aa)	APOB	STITCH	
Eucommiae Cortex (Du Zhong, DZ)	MOL000073	ent-Epicatechin	paraoxonase 1	PON1	STITCH	
Eucommiae Cortex (Du Zhong, DZ)	MOL000073	ent-Epicatechin	beta-site APP-cleaving enzyme 1	BACE1	STITCH	PubChem
Eucommiae Cortex (Du Zhong, DZ)	MOL000073	ent-Epicatechin	solute carrier family 22 (organic anion/urate transpor	SLC22A11	STITCH	
Eucommiae Cortex (Du Zhong, DZ)	MOL000073	ent-Epicatechin	colony stimulating factor 2 (granulocyte-macrophage	CSF2	STITCH	
Eucommiae Cortex (Du Zhong, DZ)	MOL000073	ent-Epicatechin	beta-secretase 1 isoform E (401 aa)	KIAA1149	STITCH	
Eucommiae Cortex (Du Zhong, DZ)	MOL000073	ent-Epicatechin	opioid receptor kappa 1	OPRK1	Pubchem	
Eucommiae Cortex (Du Zhong, DZ)	MOL000073	ent-Epicatechin	opioid receptor mu 1	OPRM1	Pubchem	
Eucommiae Cortex (Du Zhong, DZ)	MOL000073	ent-Epicatechin	opioid receptor delta 1	OPRD1	Pubchem	
Eucommiae Cortex (Du Zhong, DZ)	MOL007563	Yangambin	Protein-tyrosine phosphatase 1B	PTPN1	Pubchem	
Eucommiae Cortex (Du Zhong, DZ)	MOL009015	(-)-Tabernemontanine	polycystic kidney disease 1 (autosomal dominant)	PKD1	STITCH	
Eucommiae Cortex (Du Zhong, DZ)	MOL009027	Cyclopamine	smoothened, frizzled class receptor	SMO	Pubchem	
Eucommiae Cortex (Du Zhong, DZ)	MOL009027	Cyclopamine	sonic hedgehog signaling molecule	SHH	Pubchem	
Eucommiae Cortex (Du Zhong, DZ)	MOL009027	Cyclopamine	nuclear receptor subfamily 1 group H member 4	NR1H4	Pubchem	
Eucommiae Cortex (Du Zhong, DZ)	MOL009027	Cyclopamine	estrogen related receptor alpha	ESRRA	Pubchem	
Eucommiae Cortex (Du Zhong, DZ)	MOL009027	Cyclopamine	GLI family zinc finger 3	GLI3	Pubchem	
Eucommiae Cortex (Du Zhong, DZ)	MOL009031	chonan-9-al, 6'-methoxy-, (9	Muscarinic acetylcholine receptor M2	CHRM2	STITCH	
Eucommiae Cortex (Du Zhong, DZ)	MOL009031	chonan-9-al, 6'-methoxy-, (9	adrenoceptor beta 3	ADRB3	STITCH	
Eucommiae Cortex (Du Zhong, DZ)	MOL009031	chonan-9-al, 6'-methoxy-, (9	Butyrylcholinesterase	BCHE	Swiss Target Prediction	
Eucommiae Cortex (Du Zhong, DZ)	MOL009031	chonan-9-al, 6'-methoxy-, (9	Sodium channel protein type V alpha subunit	SCN5A	Swiss Target Prediction	
Eucommiae Cortex (Du Zhong, DZ)	MOL009031	chonan-9-al, 6'-methoxy-, (9	HERG	KCNH2	Swiss Target Prediction	
Eucommiae Cortex (Du Zhong, DZ)	MOL009031	chonan-9-al, 6'-methoxy-, (9	P-glycoprotein 1	ABCB1	Swiss Target Prediction	
Eucommiae Cortex (Du Zhong, DZ)	MOL009031	chonan-9-al, 6'-methoxy-, (9	Voltage-gated potassium channel subunit Kv1.5	KCNA5	Swiss Target Prediction	
Eucommiae Cortex (Du Zhong, DZ)	MOL009042	Helenalin	v-rel reticuloendotheliosis viral oncogene homolog A	RELA	STITCH	PubChem
Eucommiae Cortex (Du Zhong, DZ)	MOL009042	Helenalin	nuclear factor of kappa light polypeptide gene enhar	NFKBIA	STITCH	
Eucommiae Cortex (Du Zhong, DZ)	MOL009042	Helenalin	mitogen-activated protein kinase 8	MAPK8	STITCH	
Eucommiae Cortex (Du Zhong, DZ)	MOL009042	Helenalin	autophagy related 12	ATG12	STITCH	
Eucommiae Cortex (Du Zhong, DZ)	MOL009042	Helenalin	TANK-binding kinase 1	TBK1	STITCH	
Eucommiae Cortex (Du Zhong, DZ)	MOL009042	Helenalin	heat shock 70kDa protein 6 (HSP70B')	HSPA6	STITCH	
Eucommiae Cortex (Du Zhong, DZ)	MOL009042	Helenalin	vascular cell adhesion molecule 1	VCAM1	STITCH	
Eucommiae Cortex (Du Zhong, DZ)	MOL009042	Helenalin	steroidogenic acute regulatory protein	STAR	STITCH	
Eucommiae Cortex (Du Zhong, DZ)	MOL009042	Helenalin	caspase 9	CASP9	STITCH	
Eucommiae Cortex (Du Zhong, DZ)	MOL009042	Helenalin	MDM2 proto-oncogene	MDM2	Pubchem	
Eucommiae Cortex (Du Zhong, DZ)	MOL009042	Helenalin	TNF superfamily member 10	TNFSF10	Pubchem	
Eucommiae Cortex (Du Zhong, DZ)	MOL009042	Helenalin	microtubule associated protein tau	MAPT	Pubchem	
Eucommiae Cortex (Du Zhong, DZ)	MOL009042	Helenalin	huntingtin	HTT	Pubchem	
Eucommiae Cortex (Du Zhong, DZ)	MOL009042	Helenalin	tumor necrosis factor	TNF	Pubchem	
Eucommiae Cortex (Du Zhong, DZ)	MOL009042	Helenalin	G protein-coupled receptor 55	GPR55	Pubchem	
Eucommiae Cortex (Du Zhong, DZ)	MOL009042	Helenalin	apelin receptor	APLNR	Pubchem	
Eucommiae Cortex (Du Zhong, DZ)	MOL009042	Helenalin	nuclear factor kappa B subunit 1	NFKB1	Pubchem	
Eucommiae Cortex (Du Zhong, DZ)	MOL009042	Helenalin	nuclear factor kappa B subunit 2	NFKB2	Pubchem	
Eucommiae Cortex (Du Zhong, DZ)	MOL009042	Helenalin	regulator of G protein signaling 4	RGS4	Pubchem	
Eucommiae Cortex (Du Zhong, DZ)	MOL009042	Helenalin	angiotensin II receptor type 1	AGTR1	Pubchem	
Eucommiae Cortex (Du Zhong, DZ)	MOL009042	Helenalin	melanocyte inducing transcription factor	MITF	Pubchem	
Eucommiae Cortex (Du Zhong, DZ)	MOL009042	Helenalin	MDM4 regulator of p53	MDM4	Pubchem	
Eucommiae Cortex (Du Zhong, DZ)	MOL009042	Helenalin	X-box binding protein 1	XBP1	Pubchem	
Eucommiae Cortex (Du Zhong, DZ)	MOL009042	Helenalin	nuclear factor, erythroid 2 like 2	NFE2L2	Pubchem	
Eucommiae Cortex (Du Zhong, DZ)	MOL009042	Helenalin	ATPase family AAA domain containing 5	ATAD5	Pubchem	
Eucommiae Cortex (Du Zhong, DZ)	MOL009042	Helenalin	BRCA1 DNA repair associated	BRCA1	Pubchem	
Eucommiae Cortex (Du Zhong, DZ)	MOL009042	Helenalin	BRCA1 associated RING domain 1	BARD1	Pubchem	
Eucommiae Cortex (Du Zhong, DZ)	MOL009042	Helenalin	cystic fibrosis transmembrane conductance regulator	CFTR	Pubchem	
Eucommiae Cortex (Du Zhong, DZ)	MOL009042	Helenalin	SMAD family member 3	SMAD3	Pubchem	
Eucommiae Cortex (Du Zhong, DZ)	MOL009042	Helenalin	nuclear receptor subfamily 2 group E member 3	NR2E3	Pubchem	
Eucommiae Cortex (Du Zhong, DZ)	MOL009042	Helenalin	perilipin 5	PLIN5	Pubchem	
Eucommiae Cortex (Du Zhong, DZ)	MOL009042	Helenalin	abhydrolase domain containing 5	ABHD5	Pubchem	
Eucommiae Cortex (Du Zhong, DZ)	MOL009042	Helenalin	geminin DNA replication inhibitor	GMNN	Pubchem	
Eucommiae Cortex (Du Zhong, DZ)	MOL009042	Helenalin	endothelial PAS domain protein 1	EPAS1	Pubchem	
Eucommiae Cortex (Du Zhong, DZ)	MOL009042	Helenalin	TNF receptor superfamily member 10b	TNFRSF10B	Pubchem	
Eucommiae Cortex (Du Zhong, DZ)	MOL009042	Helenalin	glucagon like peptide 1 receptor	GLP1R	Pubchem	
Eucommiae Cortex (Du Zhong, DZ)	MOL009042	Helenalin	G protein-coupled receptor 183	GPR183	Pubchem	
Eucommiae Cortex (Du Zhong, DZ)	MOL009042	Helenalin	ataxin 2	ATXN2	Pubchem	
Eucommiae Cortex (Du Zhong, DZ)	MOL009042	Helenalin	hepatocyte nuclear factor 4 alpha	HNF4A	Pubchem	
Eucommiae Cortex (Du Zhong, DZ)	MOL009042	Helenalin	nuclear receptor coactivator 2	NCOA2	Pubchem	
Eucommiae Cortex (Du Zhong, DZ)	MOL009042	Helenalin	dopamine receptor D3	DRD3	Pubchem	
Eucommiae Cortex (Du Zhong, DZ)	MOL009042	Helenalin	paired box 8	PAX8	Pubchem	
Eucommiae Cortex (Du Zhong, DZ)	MOL009042	Helenalin	nuclear receptor subfamily 2 group F member 2	NR2F3	Pubchem	
Eucommiae Cortex (Du Zhong, DZ)	MOL009042	Helenalin	isocitrate dehydrogenase (NADP(+)) 1, cytosolic	IDH1	Pubchem	
Eucommiae Cortex (Du Zhong, DZ)	MOL009042	Helenalin	prion protein	PRNP	Pubchem	
Eucommiae Cortex (Du Zhong, DZ)	MOL009042	Helenalin	interleukin 1 beta	IL1B	Pubchem	
Eucommiae Cortex (Du Zhong, DZ)	MOL009042	Helenalin	telomerase reverse transcriptase	TERT	Pubchem	
Eucommiae Cortex (Du Zhong, DZ)	MOL000098	Quercetin	Induced myeloid leukemia cell differentiation protein	MCL1	STITCH	
Eucommiae Cortex (Du Zhong, DZ)	MOL000098	Quercetin	Tyrosine-protein kinase HCK	HCK	STITCH	
Eucommiae Cortex (Du Zhong, DZ)	MOL000098	Quercetin	Solute carrier family 2, facilitated glucose transporter	SLC2A2	STITCH	
Eucommiae Cortex (Du Zhong, DZ)	MOL000098	Quercetin	Cytochrome P450 2C8	CYP2C8	STITCH	
Eucommiae Cortex (Du Zhong, DZ)	MOL000098	Quercetin	ATP synthase subunit Beta, mitochondrial	ATP5B	STITCH	
Eucommiae Cortex (Du Zhong, DZ)	MOL000098	Quercetin	3-hydroxyisobutyryl-CoA hydrolase, mitochondrial	HIBCH	STITCH	
Eucommiae Cortex (Du Zhong, DZ)	MOL000098	Quercetin	serine/threonine kinase 17b	STK17B	STITCH	
Eucommiae Cortex (Du Zhong, DZ)	MOL000098	Quercetin	NADPH oxidase 4	NOX4	Swiss Target Prediction	
Eucommiae Cortex (Du Zhong, DZ)	MOL000098	Quercetin	Vasopressin V2 receptor	AVPR2	Swiss Target Prediction	
Eucommiae Cortex (Du Zhong, DZ)	MOL000098	Quercetin	Aldose reductase	AKR1B1	Swiss Target Prediction	
Eucommiae Cortex (Du Zhong, DZ)	MOL000098	Quercetin	Xanthine dehydrogenase	XDH	Swiss Target Prediction	
Eucommiae Cortex (Du Zhong, DZ)	MOL000098	Quercetin	Monoamine oxidase A	MAOA	Swiss Target Prediction	
Eucommiae Cortex (Du Zhong, DZ)	MOL000098	Quercetin	Insulin-like growth factor I receptor	IGF1R	Swiss Target Prediction	
Eucommiae Cortex (Du Zhong, DZ)	MOL000098	Quercetin	Tyrosine-protein kinase receptor FLT3	FLT3	Swiss Target Prediction	
Eucommiae Cortex (Du Zhong, DZ)	MOL000098	Quercetin	Cytochrome P450 19A1	CYP19A1	Swiss Target Prediction	
Eucommiae Cortex (Du Zhong, DZ)	MOL000098	Quercetin	Epidermal growth factor receptor erbB1	EGFR	Swiss Target Prediction	
Eucommiae Cortex (Du Zhong, DZ)	MOL000098	Quercetin	Thrombin	F2	Swiss Target Prediction	
Eucommiae Cortex (Du Zhong, DZ)	MOL000098	Quercetin	Carbonic anhydrase II	CA2	Swiss Target Prediction	
Eucommiae Cortex (Du Zhong, DZ)	MOL000098	Quercetin	Serine/threonine-protein kinase PIM1	PIM1	Swiss Target Prediction	STITCH
Eucommiae Cortex (Du Zhong, DZ)	MOL000098	Quercetin	Arachidonate 5-lipoxygenase	ALOX5	Swiss Target Prediction	
Eucommiae Cortex (Du Zhong, DZ)	MOL000098	Quercetin	Serine/threonine-protein kinase Aurora-B	AURKB	Swiss Target Prediction	
Eucommiae Cortex (Du Zhong, DZ)	MOL000098	Quercetin	Dopamine D4 receptor	DRD4	Swiss Target Prediction	
Eucommiae Cortex (Du Zhong, DZ)	MOL000098	Quercetin	Adenosine A1 receptor (by homology)	ADORA1	Swiss Target Prediction	
Eucommiae Cortex (Du Zhong, DZ)	MOL000098	Quercetin	Carbonic anhydrase VII	CA7	Swiss Target Prediction	
Eucommiae Cortex (Du Zhong, DZ)	MOL000098	Quercetin	Glyoxalase I	GLO1	Swiss Target Prediction	
Eucommiae Cortex (Du Zhong, DZ)	MOL000098	Quercetin	Myeloperoxidase	MPO	Swiss Target Prediction	
Eucommiae Cortex (Du Zhong, DZ)	MOL000098	Quercetin	PI3-kinase p85-alpha subunit	PIK3R1	Swiss Target Prediction	
Eucommiae Cortex (Du Zhong, DZ)	MOL000098	Quercetin	Adenosine A2a receptor (by homology)	ADORA2A	Swiss Target Prediction	
Eucommiae Cortex (Du Zhong, DZ)	MOL000098	Quercetin	Death-associated protein kinase 1	DAPK1	Swiss Target Prediction	
Eucommiae Cortex (Du Zhong, DZ)	MOL000098	Quercetin	Liver glycogen phosphorylase	PYGL	Swiss Target Prediction	
Eucommiae Cortex (Du Zhong, DZ)	MOL000098	Quercetin	Carbonic anhydrase I	CA1	Swiss Target Prediction	
Eucommiae Cortex (Du Zhong, DZ)	MOL000098	Quercetin	Glycogen synthase kinase-3 Beta	GSK3B	Swiss Target Prediction	
Eucommiae Cortex (Du Zhong, DZ)	MOL000098	Quercetin	Tyrosine-protein kinase SRC	SRC	Swiss Target Prediction	
Eucommiae Cortex (Du Zhong, DZ)	MOL000098	Quercetin	Focal adhesion kinase 1	PTK2	Swiss Target Prediction	
Eucommiae Cortex (Du Zhong, DZ)	MOL000098	Quercetin	Estradiol 17-Beta-dehydrogenase 2	HSD17B2	Swiss Target Prediction	
Eucommiae Cortex (Du Zhong, DZ)	MOL000098	Quercetin	Vascular endothelial growth factor receptor 2	KDR	Swiss Target Prediction	
Eucommiae Cortex (Du Zhong, DZ)	MOL000098	Quercetin	Matrix metalloproteinase 13	MMP13	Swiss Target Prediction	
Eucommiae Cortex (Du Zhong, DZ)	MOL000098	Quercetin	Matrix metalloproteinase 3	MMP3	Swiss Target Prediction	
Eucommiae Cortex (Du Zhong, DZ)	MOL000098	Quercetin	Carbonic anhydrase III	CA3	Swiss Target Prediction	
Eucommiae Cortex (Du Zhong, DZ)	MOL000098	Quercetin	Arachidonate 15-lipoxygenase	ALOX15	Swiss Target Prediction	
Eucommiae Cortex (Du Zhong, DZ)	MOL000098	Quercetin	Multidrug resistance-associated protein 1	ABCC1	Swiss Target Prediction	
Eucommiae Cortex (Du Zhong, DZ)	MOL000098	Quercetin	Serine/threonine-protein kinase PLK1	PLK1	Swiss Target Prediction	
Eucommiae Cortex (Du Zhong, DZ)	MOL000098	Quercetin	Carbonic anhydrase VI	CA6	Swiss Target Prediction	
Eucommiae Cortex (Du Zhong, DZ)	MOL000098	Quercetin	Cyclin-dependent kinase 1	CDK1	Swiss Target Prediction	
Eucommiae Cortex (Du Zhong, DZ)	MOL000098	Quercetin	Matrix metalloproteinase 9	MMP9	Swiss Target Prediction	
Eucommiae Cortex (Du Zhong, DZ)	MOL000098	Quercetin	Carbonic anhydrase XII	CA12	Swiss Target Prediction	
Eucommiae Cortex (Du Zhong, DZ)	MOL000098	Quercetin	Matrix metalloproteinase 2	MMP2	Swiss Target Prediction	
Eucommiae Cortex (Du Zhong, DZ)	MOL000098	Quercetin	Protein kinase N1	PKN1	Swiss Target Prediction	
Eucommiae Cortex (Du Zhong, DZ)	MOL000098	Quercetin	Carbonic anhydrase XIV	CA14	Swiss Target Prediction	
Eucommiae Cortex (Du Zhong, DZ)	MOL000098	Quercetin	Carbonic anhydrase IX	CA9	Swiss Target Prediction	
Eucommiae Cortex (Du Zhong, DZ)	MOL000098	Quercetin	Casein kinase II alpha	CSNK2A1	Swiss Target Prediction	
Eucommiae Cortex (Du Zhong, DZ)	MOL000098	Quercetin	Arachidonate 12-lipoxygenase	ALOX12	Swiss Target Prediction	
Eucommiae Cortex (Du Zhong, DZ)	MOL000098	Quercetin	Hepatocyte growth factor receptor	MET	Swiss Target Prediction	
Eucommiae Cortex (Du Zhong, DZ)	MOL000098	Quercetin	Carbonic anhydrase IV	CA4	Swiss Target Prediction	
Eucommiae Cortex (Du Zhong, DZ)	MOL000098	Quercetin	Serine/threonine-protein kinase NEK2	NEK2	Swiss Target Prediction	
Eucommiae Cortex (Du Zhong, DZ)	MOL000098	Quercetin	Interleukin-8 receptor A	CXCR1	Swiss Target Prediction	
Eucommiae Cortex (Du Zhong, DZ)	MOL000098	Quercetin	CaM kinase II Beta	CAMK2B	Swiss Target Prediction	
Eucommiae Cortex (Du Zhong, DZ)	MOL000098	Quercetin	ALK tyrosine kinase receptor	ALK	Swiss Target Prediction	
Eucommiae Cortex (Du Zhong, DZ)	MOL000098	Quercetin	Serine/threonine-protein kinase AKT	AKT1	Swiss Target Prediction	
Eucommiae Cortex (Du Zhong, DZ)	MOL000098	Quercetin	P-glycoprotein 1	ABCB1	Swiss Target Prediction	
Eucommiae Cortex (Du Zhong, DZ)	MOL000098	Quercetin	Serine/threonine-protein kinase NEK6	NEK6	Swiss Target Prediction	
Eucommiae Cortex (Du Zhong, DZ)	MOL000098	Quercetin	Phospholipase A2 group 1B	PLA2G1B	Swiss Target Prediction	
Eucommiae Cortex (Du Zhong, DZ)	MOL000098	Quercetin	Carbonic anhydrase VA	CA5A	Swiss Target Prediction	
Eucommiae Cortex (Du Zhong, DZ)	MOL000098	Quercetin	Beta-secretase 1	BACE1	Swiss Target Prediction	
Eucommiae Cortex (Du Zhong, DZ)	MOL000098	Quercetin	Cytochrome P450 1B1	CYP1B1	Swiss Target Prediction	STITCH
Eucommiae Cortex (Du Zhong, DZ)	MOL000098	Quercetin	Tyrosine-protein kinase receptor UFO	AXL	Swiss Target Prediction	
Eucommiae Cortex (Du Zhong, DZ)	MOL000098	Quercetin	ATP-binding cassette sub-family G member 2	ABCG2	Swiss Target Prediction	
Eucommiae Cortex (Du Zhong, DZ)	MOL000098	Quercetin	NUAK family SNF1-like kinase 1	NUAK1	Swiss Target Prediction	
Eucommiae Cortex (Du Zhong, DZ)	MOL000098	Quercetin	Aldo-keto reductase family 1 member C2 (by homo	AKR1C2	Swiss Target Prediction	
Eucommiae Cortex (Du Zhong, DZ)	MOL000098	Quercetin	Aldo-keto reductase family 1 member C1 (by homo	AKR1C1	Swiss Target Prediction	
Eucommiae Cortex (Du Zhong, DZ)	MOL000098	Quercetin	Aldo-keto-reductase family 1 member C3 (by homo	AKR1C3	Swiss Target Prediction	
Eucommiae Cortex (Du Zhong, DZ)	MOL000098	Quercetin	Aldo-keto reductase family 1 member C4 (by homo	AKR1C4	Swiss Target Prediction	
Eucommiae Cortex (Du Zhong, DZ)	MOL000098	Quercetin	Carbonic anhydrase XIII (by homology)	CA13	Swiss Target Prediction	
Eucommiae Cortex (Du Zhong, DZ)	MOL000098	Quercetin	Aldehyde reductase (by homology)	AKR1A1	Swiss Target Prediction	
Eucommiae Cortex (Du Zhong, DZ)	MOL000098	Quercetin	G-protein coupled receptor 35	GPR35	Swiss Target Prediction	
Eucommiae Cortex (Du Zhong, DZ)	MOL000098	Quercetin	Tyrosine-protein kinase SYK	SYK	Swiss Target Prediction	
Eucommiae Cortex (Du Zhong, DZ)	MOL002773	Beta-Carotene	solute carrier organic anion transporter family memb	SLCO1B1	Pubchem	
Eucommiae Cortex (Du Zhong, DZ)	MOL002773	Beta-Carotene	solute carrier organic anion transporter family memb	SLCO1B3	Pubchem	
Eucommiae Cortex (Du Zhong, DZ)	MOL002773	Beta-Carotene	ATP binding cassette subfamily B member 11	ABCB11	Pubchem	
Eucommiae Cortex (Du Zhong, DZ)	MOL002773	Beta-Carotene	Very-low-density lipoprotein particle receptor activity	VLDLR	DrugBank	
Eucommiae Cortex (Du Zhong, DZ)	MOL002773	Beta-Carotene	Retinol-binding protein 1	RBP1	DrugBank	
Eucommiae Cortex (Du Zhong, DZ)	MOL002773	Beta-Carotene	Retinol-binding protein 2	RBP2	DrugBank	
Eucommiae Cortex (Du Zhong, DZ)	MOL002773	Beta-Carotene	Retinol-binding protein 3	RBP3	DrugBank	
Eucommiae Cortex (Du Zhong, DZ)	MOL002773	Beta-Carotene	Retinol-binding protein 4	RBP4	DrugBank	
Eucommiae Cortex (Du Zhong, DZ)	MOL002773	Beta-Carotene	Retinol-binding protein 5	RBP5	DrugBank	
Eucommiae Cortex (Du Zhong, DZ)	MOL011604	Syringetin	bone morphogenetic protein 2	BMP2	STITCH	
