# Supplementary material for: Network Pharmacology Approach for Predicting Targets of Zishen Yutai Pills on Premature Ovarian Insufficiency
Source: Evid Based Complement Alternat Med. 2021 Aug 4;2021:8215454. doi: 10.1155/2021/8215454 (PMC8357500; doi:10.1155/2021/8215454)
Supplement: Supplementary Materials — Supplementary Table 1: active compounds of each herb in ZSYTP. Supplementary Table 2: corresponding targets of active compounds: (A) two target genes of SDH; (B) 138 target genes of GQZ; (C) 202 target genes of TSZ; (D) 25 target genes of BJT; (E) 121 target genes of DS; (F) 11 target genes of BZ; (G) 283 target genes of DZ; (H) 13 target genes of SR; (J) 112 target genes of RS; (K) 76 target genes of SJS; (L) 115 target genes of AY; (M) 149 target genes of HSW; (N) 68 target genes of EJ; (P) 46 target genes of LJJ. Supplementary Table 3: POI targets. Supplementary Table 4: compounds and targets attributes of C-T network. Supplementary Table 5: data of compound-target network. Supplementary Table 6: analysed C-T network: (A) active compounds of C-T network and corresponding degrees; (B) targets of C-T network and corresponding degrees. Supplementary Table 7: ZSYTP core PPI network. Supplementary Table 8: gene ontology and KEGG analysis: (A) GO-BP analysis; (B) GO-MF analysis; (C) GO-CC analysis; (D) KEGG pathway enrichment analysis. [file ST8B GO-MF.pdf › 8215454.f1/ST8B GO-MF.pdf]

## Table S8B Gene Ontology (GO) Molecular Function Analysis

| GO ID | GO Term | Term P value | No. of Genes | Associated Genes Found |
|---|---|---|---|---|
| GO:0003723 | RNA binding | 5.500E-75 | 132 | [ACTN1, ACTN4, ALDOA, ALYREF, ATP5F1A, ATP5F1C, C1QBP, CARM1, CCAR2, CCT3, CCT4, CCT5, CDKN2A, CLTC, CTNNA1, DDX1, DDX17, DDX24, DDX5, DHX15, EEF1A1, EEF1G, EEF2, EFTUD2, ENO1, EPRS1, FBL, FLNA, FLNB, FUS, H4-16, H4C1, H4C11, H4C12, H4C13, H4C14, H4C15, H4C2, H4C3, H4C4, H4C5, H4C6, H4C8, H4C9, HDGF, HNRNPAB, HNRNPD, HNRNPF, HNRNPH1, HNRNPH2, HNRNPK, HNRNPM, HNRNPR, HNRNPUL1, HSPD1, ILF2, ILF3, KHDRBS1, KRT18, MATR3, NCBP1, NCL, NONO, NOP2, NXF1, P4HB, PKM, PPIA, PRDX1, PRKDC, PRPF40A, PRPF8, RAN, RANBP2, RBBP7, RBM39, RBMX, RPL10A, RPL11, RPL13, RPL14, RPL23, RPL5, RPL6, RPL7, RPL8, RPL9, RPLP0, RPN1, RPS16, RPS19, RPS2, RPS20, RPS21, RPS3, RPS4X, RPS6, RPS8, RPSA, SF1, SF3A1, SF3B1, SF3B2, SFPQ, SLC25A5, SMARCA4, SMC1A, SNRNP200, SNRNP70, SNRPA1, SNRPB, SPTBN1, SRRM1, SRSF1, SRSF2, SRSF3, SSBP1, SUPT16H, SUPT5H, SYNCRIP, TARDBP, TCP1, TOP1, TRA2A, TUBA1B, TUBB4B, U2AF2, UBA1, UBR5, UPF1, XRCC5, YY1] |
| GO:0003729 | mRNA binding | 5.710E-17 | 29 | [C1QBP, CARM1, CCT5, DDX5, FUS, HNRNPAB, HNRNPD, HNRNPK, HNRNPM, HNRNPR, ILF3, KHDRBS1, MATR3, NCBP1, NCL, NXF1, RBMX, RPL5, RPL7, RPS2, RPS3, SF1, SF3B1, SNRNP70, SRSF1, SRSF3, SUPT5H, SYNCRIP, TARDBP] |
| GO:0008134 | transcription factor binding | 8.736E-15 | 40 | [ACTB, ACTN4, C1QBP, CCND1, CDK7, CDKN2A, CEBPB, CHD4, CRY1, CRY2, DAXX, DDX5, E2F1, FAF1, FBL, FLNA, FUS, HCFC1, HDAC3, HDAC5, HDGF, HNRNPD, HNRNPF, JUP, KAT2A, MTA2, NCOA3, NCOR1, NCOR2, PRKCB, PRKDC, RAN, RBX1, RPL23, RPS3, SKI, SMARCA4, SMARCB1, TP53BP1, UBXN7] |
| GO:0045296 | cadherin binding | 1.964E-14 | 28 | [ALDOA, CCT8, CTNNA1, DNAJB1, EEF1G, EEF2, EMD, ENO1, EPS15, FLNA, FLNB, HCFC1, HNRNPK, IQGAP1, JUP, KRT18, LDHA, LIMA1, MRE11, PICALM, PKM, PRDX1, RAN, RPL14, RPL6, RPS2, SPTBN1, TLN1] |
| GO:0019904 | protein domain specific binding | 4.143E-12 | 37 | [ATP1A1, CDKN2A, CHEK1, CLTC, DDX5, EPS15, FAF1, GAPDH, H4-16, H4C1, H4C11, H4C12, H4C13, H4C14, H4C15, H4C2, H4C3, H4C4, H4C5, H4C6, H4C8, H4C9, HNRNPK, HNRNPM, INPPL1, IQGAP1, IRS1, KHDRBS1, NCOA3, PRKDC, RBMX, SKI, SKP1, TLN1, TOP1, TUBA1A, U2AF2] |
| GO:0050839 | cell adhesion molecule binding | 6.552E-12 | 31 | [ACTN1, ACTN4, ALDOA, CCT8, CTNNA1, DNAJB1, EEF1G, EEF2, EMD, ENO1, EPS15, FLNA, FLNB, HCFC1, HNRNPK, IQGAP1, JUP, KRT18, LDHA, LIMA1, MRE11, P4HB, PICALM, PKM, PRDX1, RAN, RPL14, RPL6, RPS2, SPTBN1, TLN1] |
| GO:0140297 | DNA-binding transcription factor binding | 6.766E-12 | 26 | [ACTB, ACTN4, CDK7, CDKN2A, CEBPB, CHD4, CRY1, CRY2, DAXX, DDX5, FAF1, FUS, HCFC1, HDAC3, HDAC5, JUP, MTA2, NCOA3, NCOR1, NCOR2, PRKCB, RAN, SKI, SMARCA4, SMARCB1, TP53BP1] |
| GO:0061629 | RNA polymerase II-specific DNA-binding transcription factor binding | 1.095E-11 | 23 | [ACTB, ACTN4, CDK7, CDKN2A, CEBPB, CHD4, CRY1, CRY2, DAXX, DDX5, FAF1, FUS, HDAC3, JUP, MTA2, NCOA3, NCOR1, NCOR2, PRKCB, RAN, SMARCA4, SMARCB1, TP53BP1] |
| GO:0019900 | kinase binding | 7.048E-10 | 34 | [ACTB, ATP1A1, AURKB, C1QBP, CCND1, CDKN2A, CEBPB, CLTC, CRY1, CRY2, DAXX, E2F1, EEF1A1, EEF2, FAF1, FLNA, HDAC5, IQGAP1, IRS1, JUP, KHDRBS1, LDHA, MACROH2A1, MSH2, NCOA3, PAK1, PIK3R2, PRKCB, RPS19, RPS3, RPS6, SKI, SRSF2, TUBA4A] |
| GO:0031491 | nucleosome binding | 1.941E-09 | 11 | [ACTB, CHD4, MACROH2A1, MTA2, RBBP4, SMARCA4, SMARCA5, SMARCB1, SMARCC1, SMARCC2, SUPT16H] |
| GO:0044389 | ubiquitin-like protein ligase binding | 2.545E-09 | 21 | [CCT2, CEBPB, CUL4A, CUL4B, DAXX, FAF1, HDAC6, HSPA6, HSPD1, JAK1, RBX1, RPL11, RPL23, RPL5, SKI, SLC25A5, TCP1, TPI1, TUBA1B, UBXN7, XRCC5] |
| GO:0019901 | protein kinase binding | 7.369E-09 | 30 | [ACTB, ATP1A1, C1QBP, CCND1, CDKN2A, CLTC, CRY1, CRY2, DAXX, E2F1, EEF1A1, EEF2, FAF1, HDAC5, IQGAP1, IRS1, JUP, KHDRBS1, MACROH2A1, MSH2, NCOA3, PAK1, PIK3R2, PRKCB, RPS19, RPS3, RPS6, SKI, SRSF2, TUBA4A] |
| GO:0051427 | hormone receptor binding | 7.608E-09 | 16 | [ACTN4, CDK7, CEBPB, CRY1, CRY2, DAXX, DDX5, FUS, JAK1, JUP, NCOA3, NCOR1, NCOR2, PRKCB, RAN, SMARCA4] |
| GO:0003713 | transcription coactivator activity | 1.654E-08 | 21 | [ACTN1, ACTN4, CARM1, CCAR2, CDK7, DAXX, DDX17, DYRK1A, FUS, HCFC1, JUP, KAT2A, MTA2, NCOA3, PRKCB, SMARCA4, SMARCB1, SMARCC1, SMARCC2, WDR77, YY1] |
| GO:0004386 | helicase activity | 1.165E-07 | 14 | [CHD4, DDX1, DDX17, DDX24, DDX5, DHX15, MRE11, MSH2, SMARCA4, SMARCA5, SNRNP200, SSBP1, UPF1, XRCC5] |
| GO:0016887 | ATPase activity | 1.560E-07 | 24 | [ATP5F1A, ATP5F1B, ATP5F1C, CCT8, CDK7, CHD4, DDX1, DDX17, DDX24, DDX5, DHX15, DNAJB1, HSPA6, HSPD1, MRE11, MSH2, RBBP4, SMARCA4, SMARCA5, SNRNP200, SNRNP70, SSBP1, UPF1, XRCC5] |
| GO:0019843 | rRNA binding | 4.498E-07 | 9 | [EEF2, RPL11, RPL23, RPL5, RPL8, RPL9, RPLP0, RPS3, RPS4X] |
| GO:0035258 | steroid hormone receptor binding | 5.091E-07 | 10 | [CDK7, CEBPB, DAXX, DDX5, FUS, NCOA3, NCOR2, PRKCB, RAN, SMARCA4] |
| GO:0003725 | double-stranded RNA binding | 7.143E-07 | 9 | [ACTN1, CLTC, DDX1, DHX15, HSPD1, ILF2, ILF3, TUBA1B, TUBB4B] |
| GO:0031490 | chromatin DNA binding | 8.192E-07 | 10 | [ACTB, ACTN4, CHD4, MACROH2A1, MTA2, RBBP4, SMARCA4, SMARCB1, SMARCC1, SMARCC2] |
| GO:1990948 | ubiquitin ligase inhibitor activity | 1.915E-06 | 4 | [CDKN2A, RPL11, RPL23, RPL5] |
| GO:0050681 | androgen receptor binding | 2.073E-06 | 7 | [CDK7, DAXX, DDX5, NCOA3, PRKCB, RAN, SMARCA4] |
| GO:0016922 | nuclear receptor binding | 2.932E-06 | 10 | [CDK7, CEBPB, DAXX, DDX5, FUS, NCOA3, NCOR2, PRKCB, RAN, SMARCA4] |
| GO:0051973 | positive regulation of telomerase activity | 7.767E-06 | 6 | [AURKB, CCT2, CCT4, HNRNPD, TCP1, XRCC5] |
| GO:0036002 | pre-mRNA binding | 9.123E-06 | 6 | [DDX5, PRPF8, SF1, SRSF2, TARDBP, U2AF2] |
| GO:0003727 | single-stranded RNA binding | 1.823E-05 | 8 | [DDX1, HNRNPF, HNRNPH1, ILF3, KHDRBS1, RBMX, SYNCRIP, U2AF2] |
| GO:0003730 | mRNA 3'-UTR binding | 3.467E-05 | 8 | [CCT5, DDX5, FUS, HNRNPD, HNRNPR, ILF3, RPL5, TARDBP] |
| GO:0008094 | DNA-dependent ATPase activity | 5.041E-05 | 9 | [CDK7, CHD4, MRE11, MSH2, RBBP4, SMARCA4, SMARCA5, SSBP1, XRCC5] |
| GO:0003724 | RNA helicase activity | 7.788E-05 | 7 | [DDX1, DDX17, DDX24, DDX5, DHX15, SNRNP200, UPF1] |
| GO:0030374 | nuclear receptor transcription coactivator activity | 8.441E-05 | 7 | [ACTN1, ACTN4, CARM1, CCAR2, NCOA3, PRKCB, WDR77] |
| GO:0003720 | telomerase activity | 9.749E-05 | 6 | [AURKB, CCT2, CCT4, HNRNPD, TCP1, XRCC5] |
| GO:0051438 | regulation of ubiquitin-protein transferase activity | 1.564E-04 | 6 | [CDKN2A, RPL11, RPL23, RPL5, RPS2, SKP1] |
| GO:0004407 | histone deacetylase activity | 2.101E-04 | 5 | [HDAC3, HDAC5, HDAC6, MTA2, SKI] |
| GO:0032041 | NAD-dependent histone deacetylase activity (H3-K14 specific) | 2.476E-04 | 3 | [HDAC3, HDAC5, HDAC6] |
| GO:0030957 | Tat protein binding | 3.371E-04 | 3 | [ACTB, SMARCA4, SMARCB1] |
| GO:0008097 | 5S rRNA binding | 4.452E-04 | 3 | [EEF2, RPL11, RPL5] |
| GO:0048027 | mRNA 5'-UTR binding | 4.560E-04 | 4 | [CCT5, NCL, RPL5, SYNCRIP] |
| GO:0005080 | protein kinase C binding | 8.821E-04 | 5 | [C1QBP, HDAC5, IRS1, PRKCB, SRSF2] |
| GO:0140030 | modification-dependent protein binding | 1.233E-03 | 8 | [CARM1, DAXX, EPS15, HDAC6, HSPD1, PRPF8, SMARCA4, TP53BP1] |
| GO:0097718 | disordered domain specific binding | 1.801E-03 | 4 | [CDKN2A, CLTC, GAPDH, NCOA3] |
| GO:0008143 | poly(A) binding | 2.135E-03 | 3 | [DDX1, KHDRBS1, SYNCRIP] |
| GO:0046933 | proton-transporting ATP synthase activity, rotational mechanism | 3.223E-03 | 3 | [ATP5F1A, ATP5F1B, ATP5F1C] |
| GO:0017069 | snRNA binding | 3.534E-03 | 4 | [EFTUD2, PRPF8, SNRNP70, SNRPA1] |
| GO:0042288 | MHC class I protein binding | 3.648E-03 | 3 | [ATP5F1A, ATP5F1B, TUBB4B] |
| GO:0043022 | ribosome binding | 4.945E-02 | 3 | [C1QBP, EEF2, RPSA] |
